# Pan-European maps and models of current and future tree species distributions and their growth potential

**DOI:** 10.1016/j.dib.2026.113027

**Published:** 2026-06-27

**Authors:** Anna Wöhlbrandt, Anabel Onay, Ute Bachmann-Gigl, Wolfgang Falk, Christian Temperli, Samuel Aspalter, Debojyoti Chakraborty, Silvio Schüler, Johannes Breidenbach, Jonas Fridman, Miriam Isaac-Renton, Vladimír Šebeň, Mitja Skudnik, Tzvetan Zlatanov, Dominik Thom, Eric A. Thurm

**Affiliations:** a Landesforstanstalt Mecklenburg-Vorpommern, Department of Forest Planning/Forest Research/Information Systems, Research Unit Silviculture and Forest Growth, Zeppelinstrasse 3, 19061 Schwerin, Germany; b Chair of Silviculture, Institute of Silviculture and Forest Protection, TUD Dresden University of Technology, Pienner Strasse 8, 01737 Tharandt, Germany; c TUM Technical University of Munich, Alte Akademie 8, 85354 Freising, Germany; d LWF Bavarian State Institute of Forestry, Hans-von-Carlowitz-Platz 1, 85354 Freising, Germany; e Swiss Federal Institute for Forest, Snow and Landscape Research WSL, Zürcherstrasse 111, 8903 Birmensdorf, Switzerland; f BFW Austrian Research Centre for Forests, Seckendorff-Gudent-Weg 8, A-1131 Wien, Austria; g NIBIO Norwegian Institute of Bioeconomy Research, Division of Forest and Forest Resources, Høgskolevegen 8, 1431 ÅS, Norway; h SLU Swedish University of Agricultural Sciences, Department of Forest resource management, SE-901 83 Umeå, Sweden; i Pacific Forestry Centre, Canadian Forest Service, Natural Resources Canada, 506 Burnside Road West, Victoria, Canada; j National Forest Centre, T.G.Masaryka 22, 96001, Zvolen, Slovakia; k University of Ljubljana, Biotechnical faculty, Jamnikarjeva 101, 1000 Ljubljana, Slovenia; l Slovenian Forestry Institute, Vecna pot 2, 1000 Ljubljana, Slovenia; m Institute of Biodiversity and Ecosystem Research, Bulgarian Academy of Sciences, 2 Gagarin Street, 1113 Sofia, Bulgaria

**Keywords:** Site index model, Species distribution model, Top height, Climate suitability, Climate change, Europe, Reforestation, Tree species selection

## Abstract

Amid increasing temperatures and extended drought periods, forest managers require comprehensive information regarding the suitability of various tree species under changing climatic conditions. To address this need, we assembled a unique dataset spanning Europe, incorporating multiple data sources such as national forest inventories, forest management plans, and data from ICP Forests. Our database ultimately included over six million individual trees across 860,000 forest plots throughout Europe. Using this extensive dataset, we developed Species Distribution Models (SDM) for 30 and Site Index Models (SIM) for 25 European tree species, the latter limited by data availability. Both model types were used to generate predictions at a spatial resolution of 1 × 1 km for the periods 2011–2040, 2041–2070, and 2071–2100 under climate change scenarios RCP2.6, RCP4.5 and RCP8.5. The model predictions aim to estimate the top height and assess climate suitability across Europe under future climate conditions. One potential application of these models is in a decision support system (DSS) to inform tree species selection and management strategies in the context of climate change. Provided are the models, prediction outputs, and supporting information, as the underlying database is restricted by data use agreements.

Specifications Table

**Table:** 

Subject	Forestry
Specific subject area	Predictions of Site index models (SIM) for 25 tree species and 30 Species distribution models (SDM), respectively, under climate change on European scale.
Type of data	Models (.RData) Maps (.tif) Data tables (.excel and .csv) R-script (.R) Supplementary files (.pdf)
How data were acquired	We developed site index models (SIMs) and species distribution models (SDMs) to predict tree growth and species distributions across Europe under reference climate conditions (1981–2010) and three climate change scenarios (RCP2.6, RCP4.5, and RCP8.5). Model calibration was based on an extensive dataset compiled from national forest inventories of 35 European countries, complemented by forest management plans, the International Co-operative Programme on Assessment and Monitoring of Air Pollution Effects on Forests (ICP Forests) network, smaller presence–absence datasets, and provenance trial data. Due to time-limited data use agreements, the calibration dataset itself cannot be shared. Instead, we provide the trained models, prediction outputs, and supporting information. Instruments: open-source statistical program “R”
Data format	Analyzed (Models, Model predictions and supporting information)
Parameters for data collection	NA
Description of data collection	NA
Data source location	Primary data sources: see Table 1
Data accessibility	Repository name: ZENODO.org Data identification number: 10.5281/zenodo.15863683 Direct URL to data: https://doi.org/10.5281/zenodo.15863683
Related research article	Wöhlbrandt, Anna; Onay, Anabel; Bachmann-Gigl, Ute; Falk, Wolfgang; Temperli, Christian; Aspalter, Samuel; Chakraborty, Debojyoti; Schüler, Silvio; Breidenbach, Johannes; Fridman, Jonas; Isaac-Renton, Miriam; Šebeň, Vladimír; Skudnik, Mitja; Zlatanov, Tzvetan; Thurm, Eric A. (2026): Dual modelling approach reveals: Need for assisted migration to mitigate loss of forest productivity in Europe. Journal of environmental management, 404, 129,142. https://doi.org/10.1016/j.jenvman.2026.129142.

## 1 Value of the Data

• The extensive collection of models and predictions for over 25 tree species facilitates the estimation of species-specific productivity and climate suitability under changing climatic conditions.
• Researchers and data analysts in forestry may employ this dataset alongside the provided R-script to determine maximum top height and spatial distribution of species. Possible applications of the dataset include: climate-smart forest management planning, assisted migration strategies, reforestation and afforestation planning, risk assessment of climate-induced productivity losses, input for decision support systems (DSS), educational and methodological benchmarking resource.
• Model outputs, available as .tif files, can serve as direct informational resources or be integrated into further analyses and decision support systems.
• These predictions were generated at a 1 × 1 km spatial resolution across Europe, covering the reference period (1981–2010) and future climate scenarios RCP2.6, RCP4.5, and RCP8.5 for the periods 2011–2040, 2041–2070, and 2071–2100.
• Site index model (SIM) predictions are provided for 25 tree species to estimate top height and productivity, while species distribution model (SDM) outputs are available for 30 species to project future distributions and climate suitability.
• Both SDM and SIM outputs can be adapted to project outcomes under alternative climate scenarios and at different spatial resolutions.

## 2 Data Description

### 2.1 Site index models (SIM)

#### 2.1.1 zip-file “Site index models”

The .zip-file “Site index models” contains the SIM predictions, as well as the models, from 25 tree species across Europe, provided at a 1 × 1 km resolution. Possible applications include the assessment of spatial patterns of forest productivity, the comparison of species-specific growth potential under different climate scenarios, and support of climate-adapted species selection in forest management and planning. This raster dataset spans a geographic extent from −25° to 35° longitude and from 34° to 72° latitude, referenced in the WGS 84 coordinate system (EPSG:4326). The predictions cover the reference period (1981–2010) and future climate change scenarios RCP2.6, RCP4.5 and RCP8.5 (EURO—CORDEX, model MPI-ESM CLM) for the time periods 2011–2040, 2041–2070 and 2071–2100 (see section 3.1.2). The dimensionless Site index (SI) ranges from negative values to 1. Generally, smaller numbers suggest lower top height (m) or production potential on the site. To recalculate the SI_rel_ into top height (m), the height of SI curves are needed in a certain age and the chosen Age, for example Age = 100 (Eq. (4)). We have not converted the SI in top height but provided a formula to do so, as the user should be able to choose between different Ages, for example 50 or 100. This option can be interesting under climate change, because trees may not reach the Age of 100 years at all sites. The raster files can be directly imported into GIS software or R (please also refer to the “analysis.R” script described below) to analyse spatial patterns of site productivity or to compare scenarios across time periods.

#### 2.1.2 Nlrq-parameters table

The parameters for the non-linear quantile regression (nlrq)-table (.xlsx and .csv) hold the parameters of the SI curves as produced by the nlrq-function. They enable users to derive site-specific top height estimates for different ages, facilitating flexible productivity assessments and integration into growth modelling or decision support workflows. Using them in combination of (1), (2) or R script “analysis.R”, one can transform the SI_rel_ into top height at a specified age for a defined species.

#### 2.1.3 pdf-file “Supp. material 1: site index models”

The .pdf-file shows the site index curves, model statistics, and predictions, as well as a table of the nlrq-parameters to build the Site index curves from every model (i.e. SIM) provided within the .zip-file “Site index models”. Furthermore, the R packages used for the analysis are listed at the beginning of the pdf-file.

### 2.2 Species distribution models (SDM)

#### 2.2.1 zip-file “Species distribution models”

The .zip-file “Species distribution models” can be used in GIS or R to analyse current and future species distributions, assess climate suitability across Europe, and support reforestation planning and assisted migration strategies. It contains SDM predictions and models for 30 tree species at the same resolution and extent as the SIM predictions. The SDMs and predictions include the reference period (1981–2010) and climate change scenarios RCP2.6, RCP4.5, and RCP8.5 for the time periods 2011–2040, 2041–2070, and 2071–2100. The SDM output is the occurrence probability (OP), ranging from 0 to 1. An OP near 0 can be understood as a site on which a tree has a low probability to survive, whereas an OP near 1 symbolizes a high probability to survive.

#### 2.2.2 Table SDM thresholds

To bring the OP into practice and outline areas of climate suitability for each species, we used a data driven approach (see Section 2.2) to calculate thresholds and classify the OP. The table of the SDM thresholds, included as stand-alone excel-sheet, as well as integrated into the .pdf-file “Supp. Material 2: Species distribution models”, contains the calculated thresholds. The OP will be transferred by those thresholds into categories which we term “climate risk classes”: High climatic risk (OP value ≤ SDM_threshold_Q.05), medium climatic risk (SDM_threshold_Q.05 < OP value ≤ SDM_threshold_Q.3) and low climatic risk (SDM_threshold_Q.3 < OP value ≤ 1). Low climatic risk refers to an area where the majority of presences, or occurrences, are found today within the reference climate, corresponding to the current species distribution. The other regions are categorized as “medium climatic risk” and “high climatic risk”, indicating regions where evidence on survival of the species under the given climate remains low.

#### 2.2.3 pdf-file “Supp. material 2: species distribution models”

The .pdf-file “Supp. Material 2: Species distribution models” provides a detailed report for every SDM in a similar format like the .pdf-file “Supp. Material 1: Site index models”. For every tree species, we present model statistics, response curves, and model predictions, including the figures with the climate risk classes. At the beginning the table “SDM thresholds” is printed.

### 2.3 R-script

The R script “analysis.R” provides a reproducible workflow, i.e. functions, for applying the models to new climate data nd for post-processing outputs. Specifically, it enables users to (i) generate predictions from the SIM and SDM using user-provided climate data, (ii) convert predicted site index values into top height for a defined reference age, and (iii) classify SDM occurrence probabilities into climate suitability classes based on predefined or user-defined thresholds. This facilitates the transferability of the models to new study areas and supports further analyses and visualization.

## 3 Materials and Methods

### 3.1 Data

#### 3.1.1 Presence-absence and tree growth information

We collected presence-absence and tree growth information throughout Europe, harmonised the data sets, and built a database called 'BaRis' by combining the following data sources: 1) national forest inventory data (NFI), 2) forest management plan data, 3) the International Co-operative Programme on Assessment and Monitoring of Air pollution Effects on Forests (ICP Forests) network, 4) small presence-absence data sets, and 5) provenance trial data. Most of the data originates from NFIs and has been supplemented by other data sources such as provenance datasets, ICP Forests or forest management plans to improve data coverage. Some datasets were not publicly available and could only be accessed after signing legally binding data use agreements defining the scope of use, duration of access, authorized users, and data-sharing restrictions (Table 1).

**Table 1:** Table data providers. Given are the geographical area of the data, the type of data (NFI = national forest inventory, FMP = forest management plans, PTD = provenance trial data, PA = presences and absences, FC = data about forest conditions harmonised across Europe), data volume, period covered, the institution responsible for data acquisition, and information whether the dataset is subject to a Data use agreement.

Inventory	data	Trees	Plots	time period	institution	Data use agreement (DUA)
*or country*	*Type*	*per inventory*	*per inventory*	*start - end*	*of the data provider or publication*	*data subject to DUA*
Germany	NFI	419,745	54,593	2012	Thünen-Institut für Waldökosysteme	no
Italy	NFI	230,874	7188		Corpo Forestale dello Stato, CREA Unità di ricerca per il Monitoraggio e la Pianificazione Forestale	no
Slovenia	NFI	12,310	759		Slovenian Forestry Institute, Department for Forest and Landscape Planning and Monitoring,	yes
Sweden	NFI	497,209	32,569		Department of Forest Resource Management, Swedish University of Agricultural Sciences	yes
Netherlands	NFI	149,759	7169	2012–2013	Schelhaas M.J. et al [1]	no
Slovakia	NFI	32,682	1388		Národné lesnícke centrum	no
Norway	NFI	276,474	12,885		Breidenbach et al [2] 10.1186/s40663–020–00,261-0	yes
Spain	NFI	1,234,650	78,991	1997–2007	Tercer Inventario Forestal Nacional (IFN3) (1997–2007), Ministerio para la Transición Ecológica y el Reto Demográfico	no
Austria	NFI	65,006	10,488	2016–2021	Bundesforschungszentrum für Wald, Fachinstitut Waldinventur	yes
Austria	NFI, PA	62,258	10,488	2016–2021	Bundesforschungszentrum für Wald, Fachinstitut Waldinventur	yes
Finland	NFI	0	45,022	2014–2018	Natural Resources Institut Finland (LUKE)	yes
Bulgaria	FMP	655,518	501,534	2005–2015	Institute of Biodiversity and Ecosystem Research, Bulgarian Academy of Sciences	no
Europe (SUSTREE)	PTD	35,156	573	1963–2017	SUSTREE project, https://programme2014–20.interreg-central.eu/Content.Node/SUSTREE.html	no
Germany and Austria - Douglas fir	PTD	133,377	60		Chakraborty et al [3] 10.1371/journal.pone.0136357	no
Austria - wild fruits	PA	1930	0	2021–2023	WILDOBST project, https://www.bfw.gv.at/anbau-wildobst-foerderung/	no
Europe - Douglas fir	PTD	2708	119		Isaac-Renton et al [4] 10.1111/gcb.12604	no
Europe - Black pine	PTD	194,642	5	1971–2016	Vizcaíno-Palomar et al [5] 10.1007/s13595–019–0867–2	no
France	NFI	93,269	1,283,793	2009–2014	Institut national de l'information géographique et forestière (IGN)	no
ICP Forest - Level I	FC	494,399	20,173	2005 - 2008	ICP Forests Programme Coordinating Centre [6] http://icp-forests.net/	yes
ICP Forest - Level II	FC	1,136,531	12,166	1984–2017	ICP Forests Programme Coordinating Centre [7] http://icp-forests.net/	yes

**Fig. 1:**
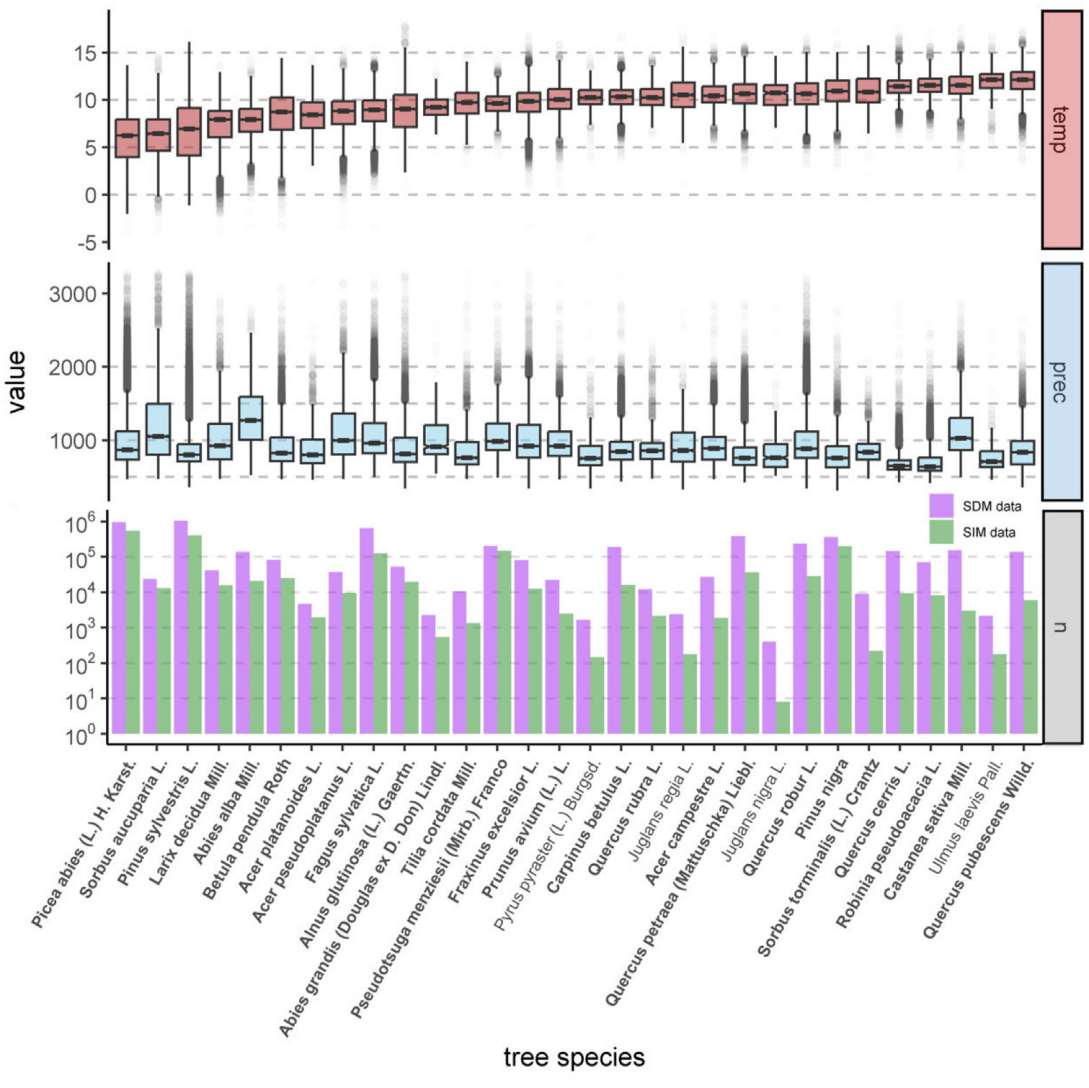
Covered tree species with their respective mean annual temperature [°C] (red) and precipitation sum [mm] (light blue) ranges, as well as the number of observations in the calibration datasets of the species distribution models (SDM) (purple bars) and the site index models (SIM) (green bars). Temperature and precipitation values were determined at the plot coordinates of the presences (CHELSA dataset, 1981 to 2010)[9]. SDMs were built for all species (30 in total). Due to insufficient data, we were able to create SIMs only for 25 species (absent labelled with normal font, all other in bold).

**Fig. 2:**
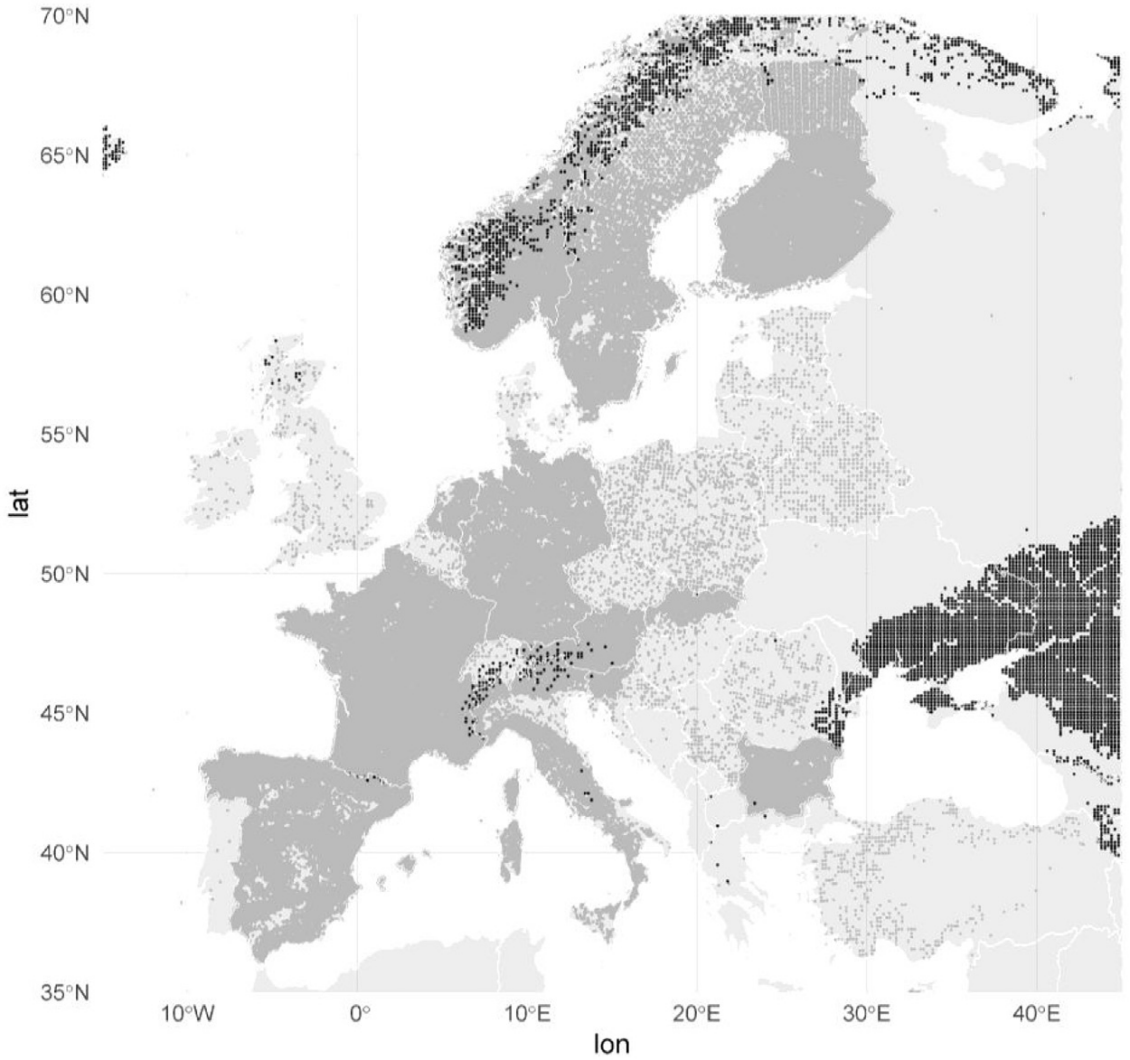
Distribution of inventory points (grey) in the BaRis database (v.7.2) and pseudo-absences (black) across Europe. Pseudo-absences were derived from Arctic tundras and alpine vegetation, as well as Steppes across Europe according to Bohn et al [8].

In the end, the database comprises a total of 2734 tree species and shrubs with 6,261,686 individual trees, including 5,882,519 individuals with growth information and 683,968 aggregated data sets (forest management plan data or mean values of individual plots), on 860,095 plots in 35 countries (Fig. 1). The database has the highest data density in central and northern Europe, while it decreases slightly in eastern and south-eastern Europe (Fig. 1). Furthermore, we determined coordinates for pseudo-absences from alpine vegetation, arctic tundras, and steppe regions across Europe according to Bohn et al [8] (Fig. 2).

#### 3.1.2 Climate data

We used climate data with a resolution of 1 × 1 km from CHELSA (v2.1) for the reference period and from EURO—CORDEX (model: MPI-ESM CLM) for three future time frames (2011–2040, 2041–2070, 2071–2100) [10,9]. The calculation of the variables was based on the CHELSA bioclim variables and was done for the reference period (1981–2010), as well as two different climate scenarios (RCP4.5 and RCP8.5).

## 4 Modelling

Model calibration was based on a harmonized dataset derived from multiple national and regional forest inventories, which were subjected to a consistent preprocessing workflow (see 4.1.1 and 4.1.2). Furthermore, we have chosen an initial set of 30 tree species for our modelling study since the species are already economically important European tree species or promising options for European forests under climate change (Fig. 1).

### 4.1 Site index models

#### 4.1.1 Data preparation

To calculate top height, we selected the tallest 20% of trees per plot and species, effectively focusing on dominant and codominant individuals. This approach excludes trees whose growth may be limited by light availability, ensuring that measured heights reflect site productivity rather than competition effects. In addition to the data harmonization, special treatments were necessary for three inventories: (a) the Finnish inventory was aggregated per sample point and tree species; therefore, the aforementioned filter was not possible. Furthermore, (b) we filtered the Dutch inventory sample points for the 20 percent of the thickest individuals after diameter at breast height and (c) rasterised the Bulgarian inventory (4 random points per 4 km grid cell). This approach aimed to sample the forest management plan data in a manner analogous to the German NFI system, thereby reducing spatial imbalances. Dead or damaged trees (if such parameters were available) were also removed.

**Fig. 3:**
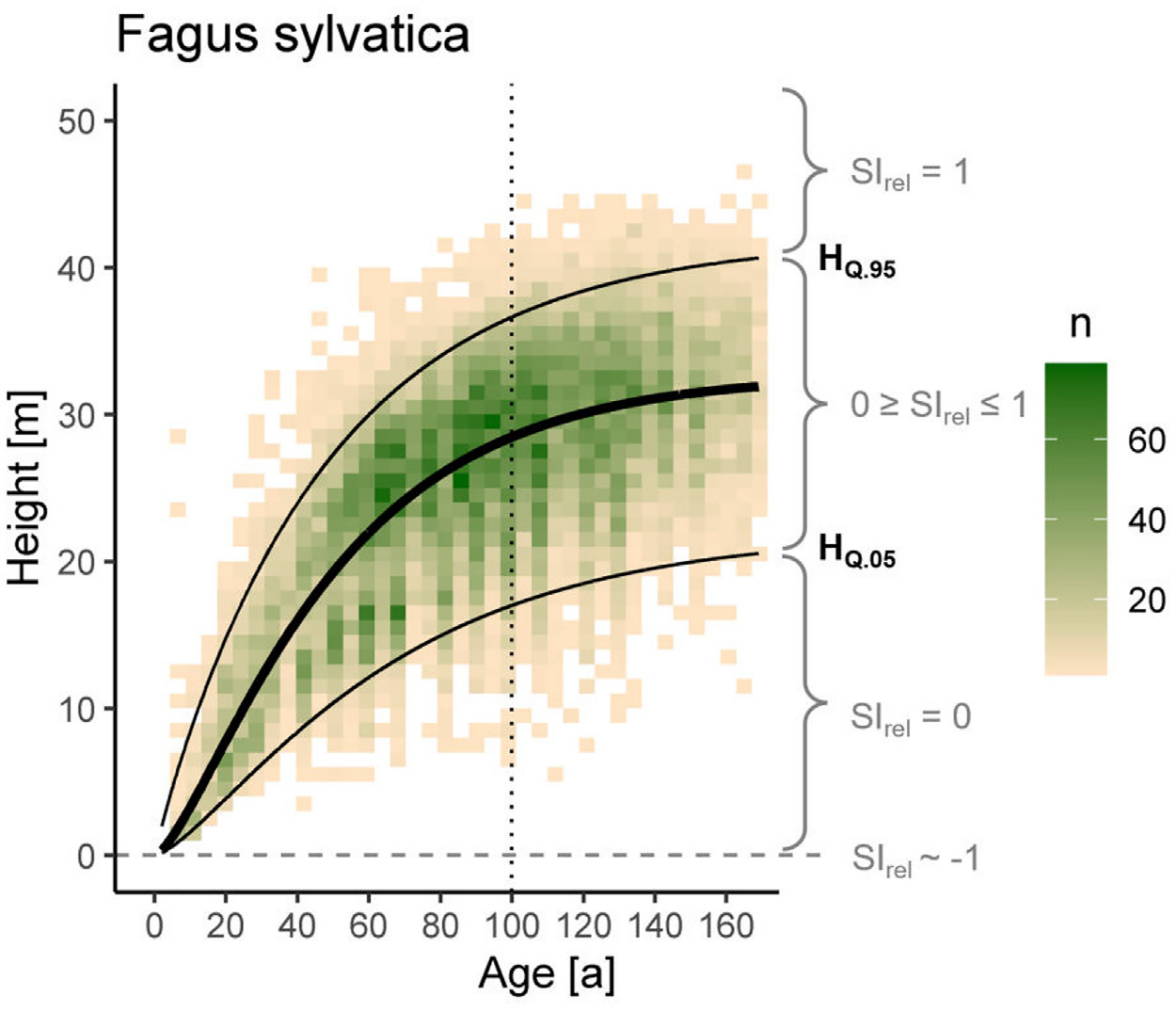
Site index curves of European beech (Fagus sylvatica L.) created with nonlinear quantile regressions based on the algorithm of Koenker and Park (1992). The site index (SI_rel_) was created by setting all points on the 95th percentile (upper line) and above to one (SI_rel_ = 1) and all on the 5th percentile (lower line) and below to zero (SIrel = 0). The points between the percentile boundaries were assigned a site index between zero and one according to the ratio of their position between the percentile boundaries (0 ≥ SI_rel_ ≤ 1). We set selected absences SI_GA_ (see Section 4.1.3) on Height = 0 m (at Age 100), which results, depending on the site index curves, for each tree species in a SI_rel_ near −1.

Furthermore, we excluded the highest age classes (e.g. older than 120 years), as these individuals can no longer be assigned a reliable age. We also trees exceeding the 95^th^ percentile of species specific age distributions. This step reduces potential bias in the upper range of the height-age relationship, where highly productive trees are often removed through thinning, leaving less productive indivuals in older age classes. To improve the visualisation of height over age figures, ages reported in classes were randomly distributed across the corresponding age interval (20 years) (Supp. Material 1). At this point, 630,948 trees or, in case of Finland and Bulgaria, mean values per inventory plot were left across the 30 initially selected tree species (Fig. 3).

#### 4.1.2 Site index curves and site index

After this selection procedure, we used the age and height data to create SI curves for the 5^th^ and 95^th^ percentiles using non-linear quantile regressions based on the algorithm by Koenker and Park [11] adjusted with the growth function from Chapman Richards [12]. Eq. (1) has three parameters *A, k,* and *p* [13]. A represents the maximum age of the 95^th^ percentile of all trees of a species, while k and p define the curvature of the function.

(1) $$H_{Q_{i}}=A\;{\left(1-e^{-k\;\times \;age_{j}}\right)}^{p}$$

**Fig. 4:**
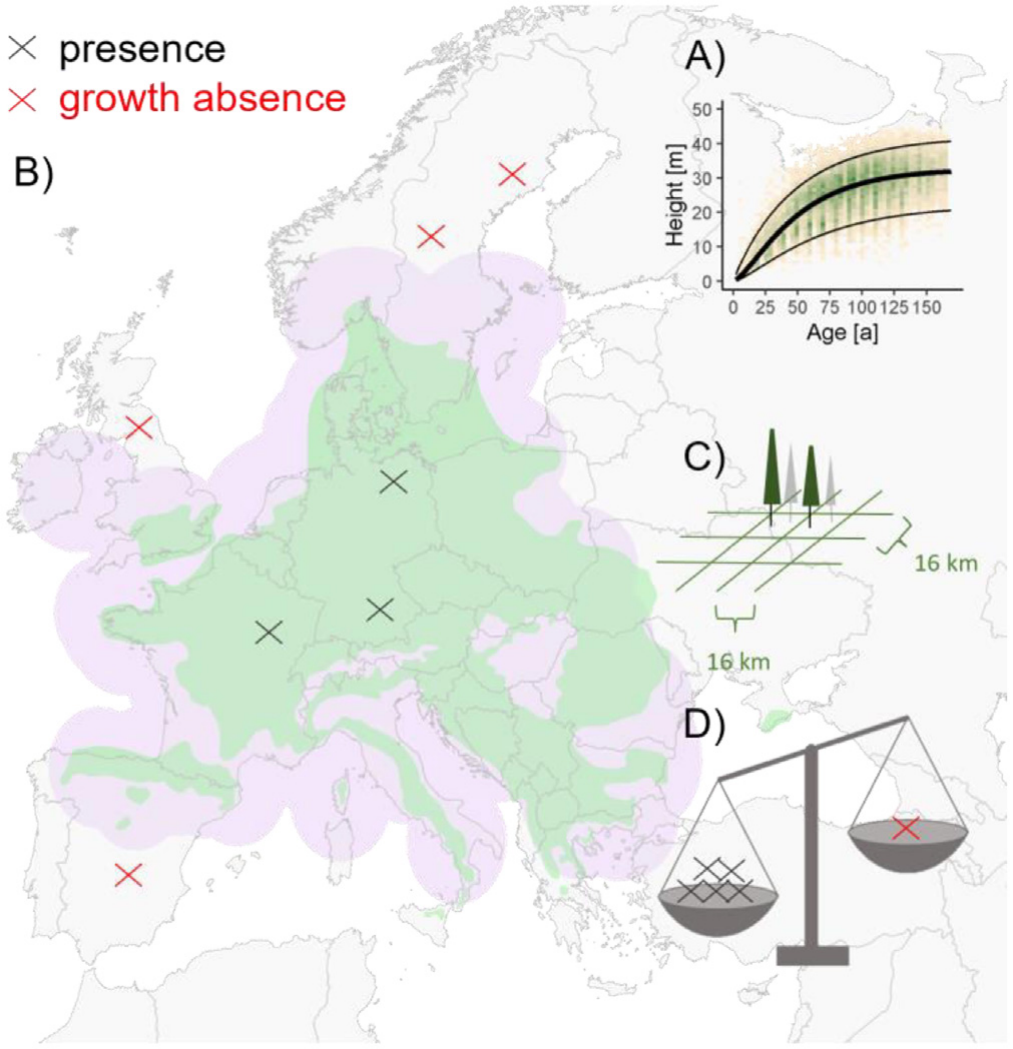
Graphic synopsis of the data preparation of the site index models (SIMs): For each tree species, we A) built site index curves of European growth information (presences, black crosses) and translated the height (m) into a Europe-wide site index, B) added growth absences (red crosses) outside their natural distribution range by Caudullo et al [14] (light green area) and outside a 200 km buffer around the presences (purple area) to represent areas that are currently not inhabited by a tree species, C) picked the highest site index value per 16 km raster cell over Europe, and D) weighted presences to absences five to one.

Since the function can only successfully fit the curve with certain sets of k and p, we built several matrices, calibrated the function with them, and visually checked and selected successful combinations (Fig. 4). Finally, we were able to create SI curves *H_Q_* for 25 species (Supp. Material 1). For the other five species, data was insufficient (Fig. 3). To convert the top height of each tree into a European-wide relative site index (*SI_rel_*), we first averaged the mean height and age values for each tree species and each inventory point. This aggregation yielded in a single mean top height *H_j_* per plot and species, reducing noise from individual tree variability and measurement errors to enhance the model’s ability to discern underlying trends. Second, the height of the 5th percentile was subtracted from the height (in meters) of a *j*-year old tree and divided by the distance from 95^th^ to 5^th^ percentile *H_Q_* (see Eq. (2)).

(2) $$SI_{rel}=\left(H_{j}-H_{Q.05_{j}}\right)÷\left(H_{Q.95_{j}}-\;H_{Q.05_{j}}\right)$$

This produces a SI_rel_ that stretches from negative values to over one, though most cluster between zero and one. All SI_rel_ height values < 0 were set to 0, and all values greater than 1 were set to 1 (Fig. 4). This procedure has two advantages: measurement errors that have not previously been corrected are normalised, and the extreme values are still included in the calibration data set for the models.

#### 4.1.3 Growth absences and pseudo-absences

To represent areas not inhabited by a tree species and avoid overshooting, we added absences to the calibration dataset. Therefore, our assumption is that growth can only occur where the environmental conditions are suitable enough to survive, aligning with the concept of the “fundamental niche” [15]. All plots where a species was not observed were designated as absences, with the absolute growth set to zero meters at age 100. Furthermore, we used pseudo-absences derived from Arctic tundras and alpine vegetation, as well as Steppes across Europe according to Bohn et al [8]. These pseudo-absences help to better define the model behavior at the edges of tree distributions. We took those coordinates and, similarly to the absences, set growth to zero meters for each species. The SI_rel_ for the growth absences (SI_GA_) was calculated using Eq. (3) and amounts for each species slightly different (around −1) depending on the Site index curves.

(3) $$SI_{GA_{100}}=H_{Q.05_{100}}÷\left(H_{Q.95_{100}}-H_{Q.05_{100}}\right)\times \;-1$$

To ensure that the points with growth absences are truly non-habitable for the species, we have built in three safety mechanisms: (a) we took absences and pseudo-absences from the database. However, it is occasionally criticized that absences within inventory plots cannot be regarded as “true” absences, since only presences are looked at in surveys (e.g. Elith et al [16]). To take this into account, absences and pseudo-absences were removed that occurred (b) within the natural distribution area according to Caudullo et al [14]. Thirdly, data points within a 250 km buffer zone surrounding known presences were omitted, as this distance approximates the maximum dispersal range for most tree species over a century (Fig. 4) [[17], [18], [19]]. The resulting SIM predictions show similarities to SDMs, which is in line with our assumption (Supp. Material 1).

#### 4.1.4 Rasterization and weighting

We selected the highest SI_rel_ values in a 16 km grid (projection WGS84, EPSG 4326), following recommendations from SDM studies to reduce spatial sampling bias [[17], [18], [19]]. The highest SI_rel_ was used to get the climatic potential of each tree species, while being constrained by the 95^th^ percentile of the Site index curves (Fig. 3 and Section 2.1.2 and Section 4.1.2). Therefore, this procedure isolates the growth potential, while limiting the influence of exceptionally favorable microsites (e.g., wind protected mountain gorges). To address the common issue of class imbalance—many more absences than presences in presence/absence modeling—we subsampled absences randomly at a 1:5 ratio, a strategy supported by literature showing that balancing class proportions enhances model sensitivity and overall performance [20]. In addition, this approach increases the relative influence of presences in model calibration while retaining a representative set of absences (Fig. 4).

#### 4.1.5 Variable selection

To identify the best predictor variables for the site index, we performed a variable selection procedure. Only climate variables from the CHELSA dataset (reference period: 1981 to 2010) and derivatives were used in the variable selection procedure described below [9]. These comprise the annual average temperature (bio1) and annual precipitation sum (bio12), as well as aggregated mean temperature values and precipitation sums for spring (March to May), summer (June to August), and winter (December to February). We deliberately omitted other bio-variables, as the interpretability is hindered by different precipitation maxima over the course of the year within Europe. For each of the 25 species, univariate generalized additive models (GAMs) were fitted and variables ranked by explanatory power (adjusted R²). We retained the variable with the highest R² and discarded highly correlated variables (ρ ≥ 0.7, Spearman). The resulting sets of two to four predictors per species were subsequently checked for interpretability and biological relevance.

#### 4.1.6 Modelling and prediction

Using the species-specific selected variables, we created multivariate GAMs for each tree species. *SI_rel_* was set as response variable. We limited the number of basis functions to three in order to obtain biologically plausible curves for each variable. For other parameters, such as family and method, the default settings of the “mgcv” package were used [21]. We have focused with the SIMs primarily on the ability to extrapolate to other geographical areas and times. Given the utilization of climate-growth relationships principles, traditional train-test validation was deemed less relevant. Model credibility was instead assessed via correlation matrices, response curves, residual distributions, and predict-over-observed plots (see Supp. Material 1). Predictions were made in the resolution of 1 × 1 km for the reference period (1981–2010) and the future (2011–2040, 2041–2070, 2071–2100) under three different climate scenarios (RCP2.6, RCP4.5, and RCP8.5). The spatial coverage of the rasters extend longitudinally from −25° to 35° and latitudinally from 34° to 72°. Since the target variable of the model is the *SI_rel_*, which ranges from 0 to 1 and is therefore not intuitively interpretable and does not allow any comparisons between the tree species, we converted the *SI_rel_* into *top height* at age 100 (Eq. (4)).

(4) $$top\;height=\;\left(H_{Q.95_{100}}-H_{Q.05_{100}}\right)\times SI_{rel}+\;H_{Q.05_{100}}$$

This SI at age 100 could be understood to mean that a tree species X planted 100 years ago would have reached the predicted top height given the climatic conditions [13].

### 4.2 Species Distribution Models

To ensure that the conditions for growth are given, we used estimations of the species distribution area of each species as a mask for the respective SIM prediction. Since SDMs improve with increasing data coverage and given the extensive coverage in the collated database, we developed independent estimates of species distributions to enhance coverage and resolution beyond that available from existing models. Due to the aforementioned database and the easier acquisition of presence-absence information, we were able to create SDMs for five additional tree species (30 in total, see Fig. 3).

#### 4.2.1 Data preparation

Species occurrence data, i.e., presences and absences, for model calibration were obtained from the BaRis database (Section 2.1.1). These data were supplemented with presence data from the EU-Forest dataset where possible [22]. Furthermore, we added the pseudo-absences according to Bohn et al [8] (Section 4.1.3). After compiling the database, a species-specific sampling was performed as described in Thurm et al [23] to harmonize the data sets and reduce autocorrelation. On a raster scale of 16 km only one inventory plot was selected randomly with preference for presences. In addition, the pine (*Pinus sylvestris*) data sets in Scandinavia (Norway, Sweden, and Finland) were reduced to 10 percent in order to compensate for an imbalance in the geographical distribution of the data.

#### 4.2.2 Variable set and modelling

The variable set for 26 species consisted of winter temperature (December-February), summer temperature (June-August), and summer precipitation (June-August). For the other four tree species black pine (*Pinus nigra*), downy oak (*Quercus pubescens*), Turkey oak (*Quercus cerris*) and chestnut (*Castanea sativa*), the climate variables consisted of winter temperature (December-February), summer temperature (June-August) and annual precipitation to consider a different precipitation pattern in the Mediterranean compared to central or northern Europe. The SDMs were fitted using GAMs with a binomial distribution and a logit link function, as well as three basis functions.

#### 4.2.3 Model performance

SDM performance was assessed using four statistical parameters: the area under the receiver operating characteristic curve (AUC), the true skill statistic (TSS), sensitivity (probability of the model to correctly predict a true presence), and specificity (probability of the model to correctly predict a true absence) [24]. AUC values below 0.7 were considered insufficient, values in the range of <0.8 to <0.9 were considered good, and values above 0.9 were considered excellent [25]. TSS is calculated as sensitivity + specificity – 1 and ranges between −1 and +1 [26]. Models with values above 0.5 were considered to have good performance [27] (Table 2).

**Table 2:** Model statistics and variables for the 25 site index models (SIMs) and the 30 species distribution models (SDMs). Given are for the SIMs the goodness of fit (R2), the correlation coefficient according to Pearson between the predicted and observed site index (cor.pre) and the heights (m) of the site index curves at age 100 for the lower quantile (SI_Q.05) and the upper quantile (SI_Q.95). Variable acronyms: Bio.1 = Mean annual temperature [°C], Bio.12 = Annual precipitation sum [mm/m2], sp_p = Sum of precipitation [mm/m2] within months 3 to 5, su_p = Sum of precipitation [mm/m2] within months 6 to 8, wi_p = Sum of precipitation [mm/m2] within months 12,1,2, sp_t = Mean temperature [°C] within months 3 to 5, su_t = Mean temperature [°C] within months 6 to 8, wi_t = Mean temperature [°C] within months 12,1,2. For the SDMs no extra variable column is necessary since the variable set stays the same (the variable set for 26 species consisted of wi_t, su_t and su_p. For the other 4 species black pine, downy oak, Turkey oak and chestnut, the climate variables consisted of wi_t, su_t and Bio.12). In addition, the performance parameters such as area under the curve (AUC), sensitivity (sens), specificity (spec), and true skill statistics (TSS) are provided.

species	SIMs					SDMs			
	R^2^	cor.pre	SI_Q.05	SI_Q.95	variables	AUC	sens	spec	TSS
*Abies alba*	0.63	0.18	23.0	37.7	wi_t, sp_p, su_t	0.86	0.83	0.71	0.55
*Abies grandis*	0.46	−0.01	29.0	41.3	Bio.1, Bio.12	0.92	0.94	0.80	0.74
*Acer campestre*	0.46	0.25	10.7	23.7	sp_p, Bio.1	0.90	0.90	0.76	0.66
*Acer platanoides*	0.66	0.17	18.7	33.7	su_t, wi_t, su_p, sp_p	0.83	0.89	0.68	0.57
*Acer pseudoplatanus*	0.62	0.24	20.3	35.5	wi_t, su_t, su_p	0.87	0.91	0.73	0.64
*Alnus glutinosa*	0.68	0.31	11.7	31.6	su_t, wi_t, su_p	0.83	0.88	0.68	0.56
*Betula pendula*	0.65	0.35	16.8	32.8	su_t, su_p	0.84	0.89	0.68	0.56
*Carpinus betulus*	0.59	0.30	12.0	29.8	wi_t, su_t, su_p	0.92	0.89	0.81	0.70
*Castanea sativa*	0.58	0.33	13.0	33.2	su_t, wi_t, sp_p, su_p	0.94	0.91	0.85	0.76
*Fagus sylvatica*	0.56	0.17	17.0	36.6	su_t, wi_t, sp_p	0.91	0.92	0.78	0.70
*Fraxinus excelsior*	0.69	0.33	20.4	38.4	wi_t, su_t, sp_p	0.88	0.86	0.74	0.60
*Juglans nigra*						0.91	0.89	0.80	0.69
*Juglans regia*						0.90	0.88	0.76	0.63
*Larix decidua*	0.36	0.31	17.3	38.6	Bio.1, Bio.12	0.83	0.90	0.68	0.58
*Picea abies*	0.63	0.66	6.4	37.8	su_t, su_p, wi_t	0.91	0.90	0.76	0.65
*Pinus nigra*	0.54	0.26	13.7	29.9	wi_t, sp_p, su_p	0.87	0.90	0.72	0.62
*Pinus sylvestris*	0.62	0.62	6.9	30.8	su_t, wi_p, su_p	0.82	0.85	0.67	0.53
*Prunus avium*	0.66	0.17	17.0	34.1	su_t, wi_t, sp_p	0.89	0.90	0.74	0.64
*Pseudotsuga menziesii*	0.69	0.21	26.7	48.1	su_t, wi_t, sp_p, su_p	0.92	0.93	0.79	0.72
*Pyrus pyraster*						0.88	0.88	0.75	0.63
*Quercus cerris*	0.58	0.20	10.7	23.7	su_t, wi_p, wi_t	0.92	0.91	0.78	0.69
*Quercus petraea*	0.57	0.25	14.6	31.7	wi_t, su_t, su_p	0.91	0.91	0.78	0.69
*Quercus pubescens*	0.45	0.40	6.0	24.3	wi_t, su_p, sp_p	0.92	0.91	0.79	0.71
*Quercus robur*	0.66	0.25	16.8	31.8	su_t, wi_t, su_p	0.90	0.88	0.77	0.66
*Quercus rubra*	0.62	0.10	23.1	37.6	su_t, su_p, wi_t	0.90	0.93	0.75	0.68
*Robinia pseudocacia*	0.51	0.24	11.1	30.9	wi_t, su_p, wi_p	0.94	0.93	0.83	0.75
*Sorbus aucuparia*	0.53	0.30	3.5	14.7	su_t, wi_t, su_p	0.86	0.86	0.70	0.56
*Sorbus torminalis*						0.91	0.92	0.77	0.69
*Tilia cordata*	0.55	0.36	14.6	29.2	Bio.1, su_p	0.86	0.86	0.72	0.57
*Ulmus laevis*						0.88	0.88	0.77	0.64

#### 4.2.4 Classification and thresholds

After model selection, we made predictions on the same geographic extent, the same periods, and climate scenarios as the SIMs. Following the selection of the model, predictions were made across the entirety of Europe at a spatial resolution of 1 × 1 km (EPSG 4326). Given that the continuous probability of occurrence (ranging from 0 to 1) is not directly suitable for application, it was discretized into three categorical classes: low, medium, and high climatic risk. This approach employs an inverse logic: a high predicted occurrence probability corresponds to low climatic risk, and vice versa, since the models are solely climate-dependent. Our objective was to utilize these classes to delineate the possible distributional ranges of each species. Thresholds were established to facilitate the categorization of these classes. We calculated thresholds according to the principle of sensitivity, which indicates the success rate of correctly predicting an observed occurrence [28]. Thus, SDM predictions were (a) created using only the coordinates of the presences, and (b) the quantiles 0.05 and 0.3 of this number of prediction points were calculated separately for each species (see Supp. Material 2 for the table of thresholds). This ensures that the same approach can be applied to all species, while still allowing the margins of the classes to adapt according to the individual distribution ranges. Quantile selection was based on a visual comparison between real occurrences and SDM thresholds in reference time (1981–2010). For this, we visually checked that the chosen set of quantile thresholds showed the majority of presence points located within the area classified as “low climatic risk” (Supp. Material 2).

## Declaration of Generative AI and AI-Assisted Technologies in the Writing Process

During the preparation of this work the authors used DeepSeek-V3 (https://chat.deepseek.com/) and PaperDigest – TextRewriter (https://www.paperdigest.org/rewriter/) in order to improve the readability and language of the manuscript. After using this services, the authors reviewed and edited the content as needed and take full responsibility for the content of the published article.

## CRediT Author Statement

**AW:** Writing – original draft, Analysis, Visualization, Methodology, Conceptualization, Data preparation, Validation. **AO:** SDM Modelling, Data preparation, Data validation, Review, and editing. **UB:** Project coordination, Data preparation, Data validation, Review, and editing. **SA:** Data preparation, Data validation, Review, and editing. **WF:** Funding acquisition, Project administration, Supervision, Review, and editing. **DC and SS:** Data provision, Funding acquisition, Review, and editing. **ET:** Conceptualization, Methodology, Supervision, Funding acquisition, Validation, Review, and editing. Others: Data provision, Review and editing.

## Data Availability

ZENODOPan-European maps and models of current and future tree species distributions and their growth potential (Original data).

## Appendix Supplementary materials

Image, application 1

Image, application 2
