# Supplementary material for: Pan-European maps and models of current and future tree species distributions and their growth potential
Source: Data Brief. 2026 Jun 27;67:113027. doi: 10.1016/j.dib.2026.113027 (PMC13355197; doi:10.1016/j.dib.2026.113027)

Supplementary material 1: Site index models

Wöhlbrandt et al. (2026)

Contents

Software 11

Table of nlrq-parameters 13

Abies alba 14

Site index curves . . . 14

Model statistics and evaluation . . . 15

Summary . . . 15

Variance inflation factor (VIF) . . . 15

Correlation matrix . . . 16

Response curves . . . 17

Response maps . . . 18

Residual distribution . . . 19

Correlation between predict and observed site index . . . 20

Predictions and forecasts . . . 21

Predict . . . 21

Forecast . . . 22

Abies grandis 23

Site index curves . . . 23

Model statistics and evaluation . . . 24

Summary . . . 24

Variance inflation factor (VIF) . . . 24

Correlation matrix . . . 25

Response curves . . . 26

Response maps . . . 27

Residual distribution . . . 28

Correlation between predict and observed site index . . . 29

Predictions and forecasts . . . 30

Predict . . . 30

Forecast . . . 31

Acer campestre 32
Site index curves 32
Model statistics and evaluation 33
Summary 33
Variance inflation factor (VIF) 33
Correlation matrix 34
Response curves 35
Response maps 36
Residual distribution 37
Correlation between predict and observed site index 38
Predictions and forecasts 39
Predict 39
Forecast 40

Acer platanoides 41
Site index curves 41
Model statistics and evaluation 42
Summary 42
Variance inflation factor (VIF) 42
Correlation matrix 43
Response curves 44
Response maps 45
Residual distribution 46
Correlation between predict and observed site index 47
Predictions and forecasts 48
Predict 48
Forecast 49

Acer pseudoplatanus 50
Site index curves 50
Model statistics and evaluation 51
Summary 51
Variance inflation factor (VIF) 51
Correlation matrix 52
Response curves 53
Response maps 54
Residual distribution 55

Correlation between predict and observed site index . . . 56
Predictions and forecasts . . . 57
Predict . . . 57
Forecast . . . 58

Alnus glutinosa **59**
Site index curves . . . 59
Model statistics and evaluation . . . 60
Summary . . . 60
Variance inflation factor (VIF) . . . 60
Correlation matrix . . . 61
Response curves . . . 62
Response maps . . . 63
Residual distribution . . . 64
Correlation between predict and observed site index . . . 65
Predictions and forecasts . . . 66
Predict . . . 66
Forecast . . . 67

Betula pendula **68**
Site index curves . . . 68
Model statistics and evaluation . . . 69
Summary . . . 69
Variance inflation factor (VIF) . . . 69
Correlation matrix . . . 70
Response curves . . . 71
Response maps . . . 72
Residual distribution . . . 73
Correlation between predict and observed site index . . . 74
Predictions and forecasts . . . 75
Predict . . . 75
Forecast . . . 76

Carpinus betulus **77**
Site index curves . . . 77
Model statistics and evaluation . . . 78
Summary . . . 78
Variance inflation factor (VIF) . . . 78

Correlation matrix . . . 79
Response curves . . . 80
Response maps . . . 81
Residual distribution . . . 82
Correlation between predict and observed site index . . . 83
Predictions and forecasts . . . 84
Predict . . . 84
Forecast . . . 85

Castanea sativa **86**
Site index curves . . . 86
Model statistics and evaluation . . . 87
Summary . . . 87
Variance inflation factor (VIF) . . . 87
Correlation matrix . . . 88
Response curves . . . 89
Response maps . . . 90
Residual distribution . . . 91
Correlation between predict and observed site index . . . 92
Predictions and forecasts . . . 93
Predict . . . 93
Forecast . . . 94

Fagus sylvatica **95**
Site index curves . . . 95
Model statistics and evaluation . . . 96
Summary . . . 96
Variance inflation factor (VIF) . . . 96
Correlation matrix . . . 97
Response curves . . . 98
Response maps . . . 99
Residual distribution . . . 100
Correlation between predict and observed site index . . . 101
Predictions and forecasts . . . 102
Predict . . . 102
Forecast . . . 103

Fraxinus excelsior **104**
Site index curves . . . 104
Model statistics and evaluation . . . 105
Summary . . . 105
Variance inflation factor (VIF) . . . 105
Correlation matrix . . . 106
Response curves . . . 107
Response maps . . . 108
Residual distribution . . . 109
Correlation between predict and observed site index . . . 110
Predictions and forecasts . . . 111
Predict . . . 111
Forecast . . . 112

Larix decidua **113**
Site index curves . . . 113
Model statistics and evaluation . . . 114
Summary . . . 114
Variance inflation factor (VIF) . . . 114
Correlation matrix . . . 115
Response curves . . . 116
Response maps . . . 117
Residual distribution . . . 118
Correlation between predict and observed site index . . . 119
Predictions and forecasts . . . 120
Predict . . . 120
Forecast . . . 121

Picea abies **122**
Site index curves . . . 122
Model statistics and evaluation . . . 123
Summary . . . 123
Variance inflation factor (VIF) . . . 123
Correlation matrix . . . 124
Response curves . . . 125
Response maps . . . 126
Residual distribution . . . 127

Correlation between predict and observed site index . . . 128
Predictions and forecasts . . . 129
Predict . . . 129
Forecast . . . 130

Pinus nigra **131**
Site index curves . . . 131
Model statistics and evaluation . . . 132
Summary . . . 132
Variance inflation factor (VIF) . . . 132
Correlation matrix . . . 133
Response curves . . . 134
Response maps . . . 135
Residual distribution . . . 136
Correlation between predict and observed site index . . . 137
Predictions and forecasts . . . 138
Predict . . . 138
Forecast . . . 139

Pinus sylvestris **140**
Site index curves . . . 140
Model statistics and evaluation . . . 141
Summary . . . 141
Variance inflation factor (VIF) . . . 141
Correlation matrix . . . 142
Response curves . . . 143
Response maps . . . 144
Residual distribution . . . 145
Correlation between predict and observed site index . . . 146
Predictions and forecasts . . . 147
Predict . . . 147
Forecast . . . 148

Prunus avium **149**
Site index curves . . . 149
Model statistics and evaluation . . . 150
Summary . . . 150
Variance inflation factor (VIF) . . . 150

Correlation matrix 151
Response curves 152
Response maps 153
Residual distribution 154
Correlation between predict and observed site index 155
Predictions and forecasts 156
Predict 156
Forecast 157

Pseudotsuga menziesii **158**
Site index curves 158
Model statistics and evaluation 159
Summary 159
Variance inflation factor (VIF) 159
Correlation matrix 160
Response curves 161
Response maps 162
Residual distribution 163
Correlation between predict and observed site index 164
Predictions and forecasts 165
Predict 165
Forecast 166

Quercus cerris **167**
Site index curves 167
Model statistics and evaluation 168
Summary 168
Variance inflation factor (VIF) 168
Correlation matrix 169
Response curves 170
Response maps 171
Residual distribution 172
Correlation between predict and observed site index 173
Predictions and forecasts 174
Predict 174
Forecast 175

Quercus petraea **176**
Site index curves . . . 176
Model statistics and evaluation . . . 177
Summary . . . 177
Variance inflation factor (VIF) . . . 177
Correlation matrix . . . 178
Response curves . . . 179
Response maps . . . 180
Residual distribution . . . 181
Correlation between predict and observed site index . . . 182
Predictions and forecasts . . . 183
Predict . . . 183
Forecast . . . 184

Quercus pubescens **185**
Site index curves . . . 185
Model statistics and evaluation . . . 186
Summary . . . 186
Variance inflation factor (VIF) . . . 186
Correlation matrix . . . 187
Response curves . . . 188
Response maps . . . 189
Residual distribution . . . 190
Correlation between predict and observed site index . . . 191
Predictions and forecasts . . . 192
Predict . . . 192
Forecast . . . 193

Quercus robur **194**
Site index curves . . . 194
Model statistics and evaluation . . . 195
Summary . . . 195
Variance inflation factor (VIF) . . . 195
Correlation matrix . . . 196
Response curves . . . 197
Response maps . . . 198
Residual distribution . . . 199

Correlation between predict and observed site index . . . 200
Predictions and forecasts . . . 201
Predict . . . 201
Forecast . . . 202

Quercus rubra **203**
Site index curves . . . 203
Model statistics and evaluation . . . 204
Summary . . . 204
Variance inflation factor (VIF) . . . 204
Correlation matrix . . . 205
Response curves . . . 206
Response maps . . . 207
Residual distribution . . . 208
Correlation between predict and observed site index . . . 209
Predictions and forecasts . . . 210
Predict . . . 210
Forecast . . . 211

Robinia pseudoacacia **212**
Site index curves . . . 212
Model statistics and evaluation . . . 213
Summary . . . 213
Variance inflation factor (VIF) . . . 213
Correlation matrix . . . 214
Response curves . . . 215
Response maps . . . 216
Residual distribution . . . 217
Correlation between predict and observed site index . . . 218
Predictions and forecasts . . . 219
Predict . . . 219
Forecast . . . 220

Sorbus aucuparia **221**
Site index curves . . . 221
Model statistics and evaluation . . . 222
Summary . . . 222
Variance inflation factor (VIF) . . . 222

Correlation matrix 223
Response curves 224
Response maps 225
Residual distribution 226
Correlation between predict and observed site index 227
Predictions and forecasts 228
Predict 228
Forecast 229

Tilia cordata **230**

Site index curves 230
Model statistics and evaluation 231
Summary 231
Variance inflation factor (VIF) 231
Correlation matrix 232
Response curves 233
Response maps 234
Residual distribution 235
Correlation between predict and observed site index 236
Predictions and forecasts 237
Predict 237
Forecast 238

Software

Table of additional R-packages. Overview of add-on packages used for the open-source statistical software "R" (version 4.2.3), their versions, short descriptions and references

id	packages	version	description	reference
1	DBI	1.1.3	*R Database Interface*	R-SIG-DB (2024)
2	circlize	0.4.15	*Circular Visualization*	Gu (2014)
3	dplyr	1.1.0	*A Grammar of Data Manipulation*	Wickham et al. (2023)
4	egg	0.4.5	*Extensions for 'ggplot2'*	Auguie (2017)
5	ggnewscale	0.4.10	*Multiple Fill and Colour Scales in 'ggplot2'*	Campitelli
6	ggplot2	3.4.0	*Create Elegant Data Visualisations Using the Grammar of Graphics*	Wickham et al. (2016)
7	ggpubr	0.6.0	*'ggplot2' Based Publication Ready Plots*	Kassambara (2023)
8	ggspatial	1.1.7	*Spatial Data Framework for ggplot2*	Dunnigton et al. (2023)
9	ggrepel	0.9.3	*Automatically Position Non-Overlapping Text Labels with 'ggplot2'*	Slowikowski (2024)
10	lwgeom	0.2-14	*Bindings to Selected 'liblwgeom' Functions for Simple Features*	Pebesma (2024)
11	mgcv	1.8.42	*Mixed GAM Computation Vehicle with Automatic Smoothness Estimation*	Wood (2011)
12	plyr	1.8.8	*Tools for Splitting, Applying and Combining Data*	Wickham (2011)
13	quantreg	5.94	*Quantile Regression*	Koenker et al. (2002)
14	RColorBrewer	1.1.3	*ColorBrewer Palettes*	Neuwirth (2022)
15	raster	3.6.14	*Geographic Data Analysis and Modeling*	Hijmans (2010)
16	readxl	1.4.1	*Read Excel Files*	Wickham and Bryan (2023)
17	rmarkdown	2.21	*Dynamic documents for R*	Allaire et al. (2023)
18	RSQLite	2.2.20	*SQLite Interface for R*	MÃ¼ller et al. (2022)
19	sf	1.0.9	*Simple Features for R*	Pebesma and Bivand (2018)
20	sfheaders	0.4.0	*Converts Between R Objects and Simple Feature Objects*	Cooley and Summer (2023)
21	terra	1.7.3	*Spatial Data Analysis*	Hijmans et al. (2024)
22	tidyterra	0.4.0	*'tidyverse' Methods and 'ggplot2' Helpers for 'terra' Objects*	HernangÃ³mez (2023)
23	writexl	1.4.2	*Export Data Frames to Excel 'xlsx' Format*	Ooms (2024)

References

Allaire J; Xie Y; Dervieux C; McPherson J; Luraschi J; Ushey K et al. (2023): Dynamic Documents for R. package 'rmarkdown'. Version 2.21. for open-source statistical software 'R'. Available online at https://github.com/rstudio/rmarkdown.

Auguie, Baptiste (2017): Extensions for 'ggplot2': Custom Geom, Custom Themes, Plot Alignment, Labelled Panels, Symmetric Scales, and Fixed Panel Size. package 'egg'. Version 0.4.5. for open-source statistical software 'R'. Available online at https://cran.r-project.org/package=egg.

Campitelli, Elio (2024): Multiple Fill and Colour Scales in 'ggplot2'. package 'ggnewscale'. Version 0.4.10. for open-source statistical software 'R'. Available online at https://cran.r-project.org/package=ggnewscale.

Cooley, David; Summer, Michael (2023): Converts Between R Objects and Simple Feature Objects. package 'sfheaders'. Version 0.4.0. for open-source statistical software 'R'. Available online at https://cran.r-project.org/package=sfheaders.

Dunnigton, Dewey; Thorne, Brent; Hernangómez, Diego (2023): Spatial Data Framework for ggplot2. package 'ggspatial'. Version 1.1.7. for open-source statistical software 'R'. Available online at https://cran.r-project.org/package=ggspatial.

Gu, Zuguang (2014): Circular Visualization. package 'circlize'. Version 0.4.15. for open-source statistical software 'R'. Available online at https://cran.r-project.org/package=circlize.

Hernangómez, Diego (2023): 'tidyverse' Methods and 'ggplot2' Helpers for 'terra' Objects. package 'tidyterra'. Version 0.4.0. Available online at https://cran.r-project.org/package=tidyterra.

Hijmans, Robert J. (2010): Geographic Data Analysis and Modeling. package 'raster'. Version 3.6.14. for open-source statistical software 'R'. Available online at https://cran.r-project.org/package=raster.

Hijmans, Robert J.; Bivand, Roger; Pebesma, Edzer; Sumner, Michael D. (2024): Spatial Data Analysis. package 'terra'. Version 1.7.3. for open-source statistical software 'R'. Available online at https://cran.r-project.org/package=terra.

Kassambara, Alboukadel (2023): ‘ggplot2’ Based Publication Ready Plots. package ‘ggpubr’. Version 0.6.0. for open-source statistical software ‘R’. Available online at https://cran.r-project.org/package=ggpubr.

Koenker, Roger; Grosjean, Philippe; Koenker, Maintainer Roger (2002): Quantile Regression. package ‘quantreg’. Version 5.94. for open-source statistical software ‘R’. Available online at https://cran.r-project.org/package=quantreg.

Müller, Kirill; Wickham, Hadley; James, David A.; Falcon, Seth (2022): SQLite Interface for R. package ‘RSQLite’. Version 2.2.20. for open-source statistical software ‘R’. Available online at https://cran.r-project.org/package=RSQLite.

Neuwirth, Erich (2022): ColorBrewer Palettes. package ‘RColorBrewer’. Version 1.1.3. for open-source statistical software ‘R’. Available online at https://cran.r-project.org/package=RColorBrewer.

Ooms, Jeroen (2024): Export Data Frames to Excel ‘xlsx’ Format. package ‘writexl’. Version 1.4.2. for open-source statistical software ‘R’. Available online at https://cran.r-project.org/package=writexl.

Pebesma, Edzer (2024): Bindings to Selected ‘liblwgeom’ Functions for Simple Features. package ‘lwgeom’. Version 0.2-14. for open-source statistical software ‘R’. Available online at https://cran.r-project.org/package=lwgeom.

Posit team (2023): RStudio. Integrated Development Environment for R. Version 2023.03.0+386. Boston, MA: Posit Software, PBC. Available online at http://www.posit.co/.

R Core Team (2023): R. A Language and Environment for Statistical Computing. Version 4.2.3 (2023-03-15 ucrt). Vienna, Austria: R Foundation for Statistical Computing. Available online at https://www.R-project.org/.

R Special Interest Group on Databases; Wickham, Hadley; Müller, Kirill; R Consortium (2024): R Database Interface. package ‘DBI’. Version 1.1.3. for open-source statistical software ‘R’. Available online at https://cran.r-project.org/package=DBI.

Slowikowski, Kamil (2024): Automatically Position Non-Overlapping Text Labels with ‘ggplot2’. package ‘ggrepel’. Version 0.9.3. for open-source statistical software ‘R’. Available online at https://cran.r-project.org/package=ggrepel.

Wickham, Hadley (2011): Tools for Splitting, Applying and Combining Data. package ‘plyr’. Version 1.8.8. for open-source statistical software ‘R’. Available online at https://cran.r-project.org/package=plyr.

Wickham, Hadley; Bryan, Jennifer (2023): Read Excel Files. package ‘readxl’. Version 1.4.1. for open-source statistical software ‘R’. Available online at https://cran.r-project.org/package=readxl.

Wickham, Hadley; Chang, Winston; Henry, Lionel; Pedersen, Thomas Lin; Takahashi, Kohske; Wilke, Claus et al. (2016): Create Elegant Data Visualisations Using the Grammar of Graphics. package ‘ggplot2’. Version 3.4.0. for open-source statistical software ‘R’: Springer-Verlag New York. Available online at https://cran.r-project.org/package=ggplot2.

Wickham, Hadley; Francois, Romain; Henry, Lionel; Müller, Kirill; Vaughan, Davis; Posit Software (2023): A Grammar of Data Manipulation. package ‘dplyr’. Version 1.1.0. for open-source statistical software ‘R’. Available online at https://cran.r-project.org/package=dplyr.

Wood, Simon (2011): Mixed GAM Computation Vehicle with Automatic Smoothness Estimation. package ‘mgcv’. Version 1.8.42. for open-source statistical software ‘R’. Available online at https://cran.r-project.org/package=mgcv.

Table of nlrq-parameters

Table with parameters (nlrq-function). Parameter for the non-linear quantile regression based on the algorithm by Koenker and Park (1992) adjusted with the growth function from Chapman Richards (Richards 1959). The table includes start parameter (k and p value), the age limits, which we filtered for beforehand, and the coefficients (A, k, p) that defines the curvature of the site index curves of the 5 percent quantile and the 95 percent quantile.

	start parameter		coefficients						age (range)	
species	k	p	AQ0.05	kQ0.05	pQ0.05	AQ0.95	kQ0.95	pQ0.95	agemin	agemax
Abies alba	0.012	0.652	24.424	0.036	2.156	42.255	0.019	0.725	0	165
Abies grandis	0.012	0.652	30.037	0.044	2.806	42.532	0.037	1.139	0	54
Acer campestre	0.012	0.652	28.914	0.001	0.431	30.000	0.012	0.652	0	80
Acer platanoides	0.012	0.600	21.764	0.018	0.814	41.350	0.009	0.395	0	134
Acer pseudoplatanus	0.015	0.300	21.728	0.027	1.039	39.477	0.019	0.674	0	125
Alnus glutinosa	0.012	0.300	12.041	0.038	1.383	34.474	0.017	0.457	0	100
Betula pendula	0.012	0.600	16.938	0.059	2.107	40.700	0.012	0.600	0	92
Carpinus betulus	0.012	0.300	13.770	0.018	0.742	42.308	0.006	0.432	0	130
Castanea sativa	0.025	0.652	13.052	0.045	0.728	37.754	0.016	0.542	0	126
Fagus sylvatica	0.012	0.652	21.990	0.018	1.470	41.983	0.020	0.943	0	170
Fraxinus excelsior	0.015	0.300	22.599	0.024	1.082	43.446	0.017	0.631	0	130
Larix decidua	0.012	0.200	17.518	0.060	4.060	40.850	0.029	0.970	0	130.5
Picea abies	0.012	0.300	6.405	0.088	3.988	45.872	0.012	0.535	0	140
Pinus nigra	0.015	0.300	14.042	0.041	1.449	30.202	0.047	1.078	0	105
Pinus sylvestris	0.012	0.652	6.864	0.141	7.970	37.550	0.012	0.538	0	148
Prunus avium	0.025	2.000	29.862	0.004	0.505	36.646	0.023	0.699	0	94
Pseudotsuga menziesii	0.012	0.652	26.821	0.067	3.468	52.659	0.024	0.928	0	86
Quercus cerris	0.012	0.652	28.914	0.001	0.431	30.000	0.012	0.652	0	60
Quercus petraea	0.005	1.200	44.700	0.005	1.200	39.528	0.013	0.735	0	175
Quercus pubescens	0.020	1.700	7.102	0.011	0.415	33.988	0.008	0.544	0	118
Quercus robur	0.012	0.652	19.887	0.022	1.449	37.074	0.016	0.697	0	181
Quercus rubra	0.020	0.260	26.481	0.018	0.791	41.557	0.019	0.654	0	109
Robinia pseudoacacia	0.012	0.652	11.996	0.019	0.464	32.747	0.026	0.742	0	70
Sorbus aucuparia	0.100	0.652	3.468	0.104	0.221	14.708	0.104	0.638	0	114
Tilia cordata	0.040	2.710	15.269	0.036	1.579	29.878	0.038	0.988	0	100.2

Abies alba

Site index curves

Site index curves of Abies alba created with non-linear quantile regressions based on the algorithm of Koenker and Park (1992). The site index (SI) was created by setting all points on the 95 percent quantile (upper line) and above to one (SI = 1) and all on the 5 percent quantile (lower line) and below to zero (SI = 0). The points between the quantile boundaries were assigned a site index between zero and one according to the ratio of their position between the quantile boundaries. We set selected absences (see chapter 2.1.3) on Height = 0 m (at age 100), which means, depending on the site index curves, for each tree species a SI near -1 (red line).

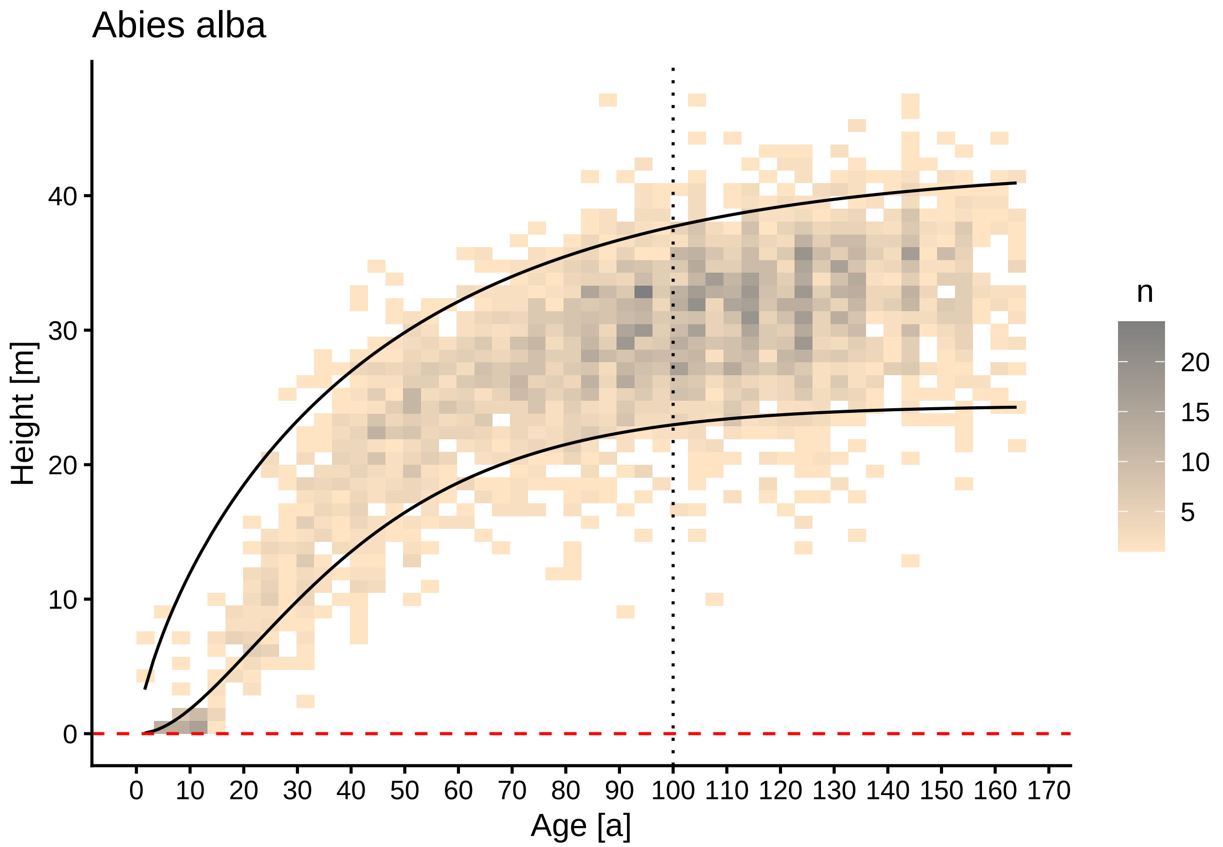

Model statistics and evaluation

Summary

Predictor acronyms: Bio.1 = Mean annual temperature [°C], Bio.12 = Annual precipitation sum [mm/m2], sp_p = Sum of precipitation [mm/m2] within months 3 to 5, su_p = Sum of precipitation [mm/m2] within months 6 to 8, wi_p = Sum of precipitation [mm/m2] within months 12,1,2, sp_t = Mean temperature [°C] within months 3 to 5, su_t = Mean temperature [°C] within months 6 to 8, wi_t = Mean temperature [°C] within months 12,1,2.

```
##
## Family: gaussian
## Link function: identity
##
## Formula:
## H03 ~ s(reference_19812010_wi_t, k = 3) + s(reference_19812010_sp_p,
##     k = 3) + s(reference_19812010_su_t, k = 3)
##
## Parametric coefficients:
##             Estimate Std. Error t value Pr(>|t|)
## (Intercept)  0.21492    0.01715   12.53   <2e-16 ***
## ---
## Signif. codes:  0 '***' 0.001 '**' 0.01 '*' 0.05 '.' 0.1 ' ' 1
##
## Approximate significance of smooth terms:
##                              edf Ref.df     F p-value
## s(reference_19812010_wi_t) 1.993      2 87.13  <2e-16 ***
## s(reference_19812010_sp_p) 1.991      2 91.23  <2e-16 ***
## s(reference_19812010_su_t) 1.988      2 47.61  <2e-16 ***
## ---
## Signif. codes:  0 '***' 0.001 '**' 0.01 '*' 0.05 '.' 0.1 ' ' 1
##
## R-sq.(adj) =  0.634   Deviance explained = 63.7%
## -REML = 682.54  Scale est. = 0.26141   n = 889
```

Variance inflation factor (VIF)

Predictor acronyms: Bio.1 = Mean annual temperature [°C], Bio.12 = Annual precipitation sum [mm/m2], sp_p = Sum of precipitation [mm/m2] within months 3 to 5, su_p = Sum of precipitation [mm/m2] within months 6 to 8, wi_p = Sum of precipitation [mm/m2] within months 12,1,2, sp_t = Mean temperature [°C] within months 3 to 5, su_t = Mean temperature [°C] within months 6 to 8, wi_t = Mean temperature [°C] within months 12,1,2.

```
##                  Variables      VIF
## 1 reference_19812010_wi_t 2.098076
## 2 reference_19812010_sp_p 1.406082
## 3 reference_19812010_su_t 2.397632
```

Correlation matrix

Correlation matrix between the predictor variables and the target variable in the model. Correlation coefficient according to PEARSON. Predictor acronyms: Bio.1 = Mean annual temperature [°C], Bio.12 = Annual precipitation sum [mm/m2], sp__p = Sum of precipitation [mm/m2] within months 3 to 5, su__p = Sum of precipitation [mm/m2] within months 6 to 8, wi__p = Sum of precipitation [mm/m2] within months 12,1,2, sp__t = Mean temperature [°C] within months 3 to 5, su__t = Mean temperature [°C] within months 6 to 8, wi__t = Mean temperature [°C] within months 12,1,2.

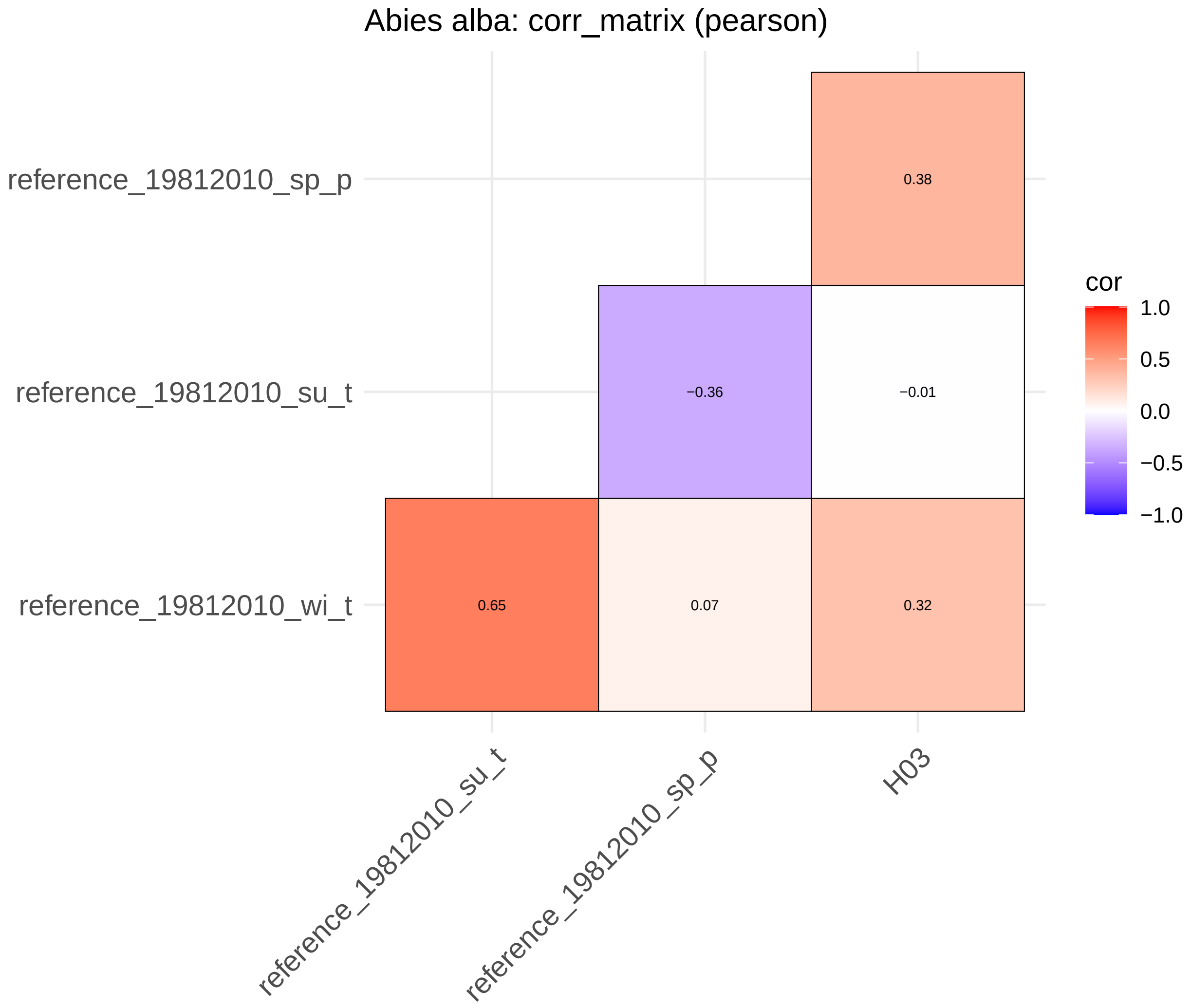

Response curves

Response curves (also known as effect curves) show how each predictor variable affects the target variable (H03 = european Site index, SIrel). H03 values below zero represent 'Growth absences'. Predictor acronyms: Bio.1 = Mean annual temperature [°C], Bio.12 = Annual precipitation sum [mm/m2], sp_p = Sum of precipitation [mm/m2] within months 3 to 5, su_p = Sum of precipitation [mm/m2] within months 6 to 8, wi_p = Sum of precipitation [mm/m2] within months 12,1,2, sp_t = Mean temperature [°C] within months 3 to 5, su_t = Mean temperature [°C] within months 6 to 8, wi_t = Mean temperature [°C] within months 12,1,2.

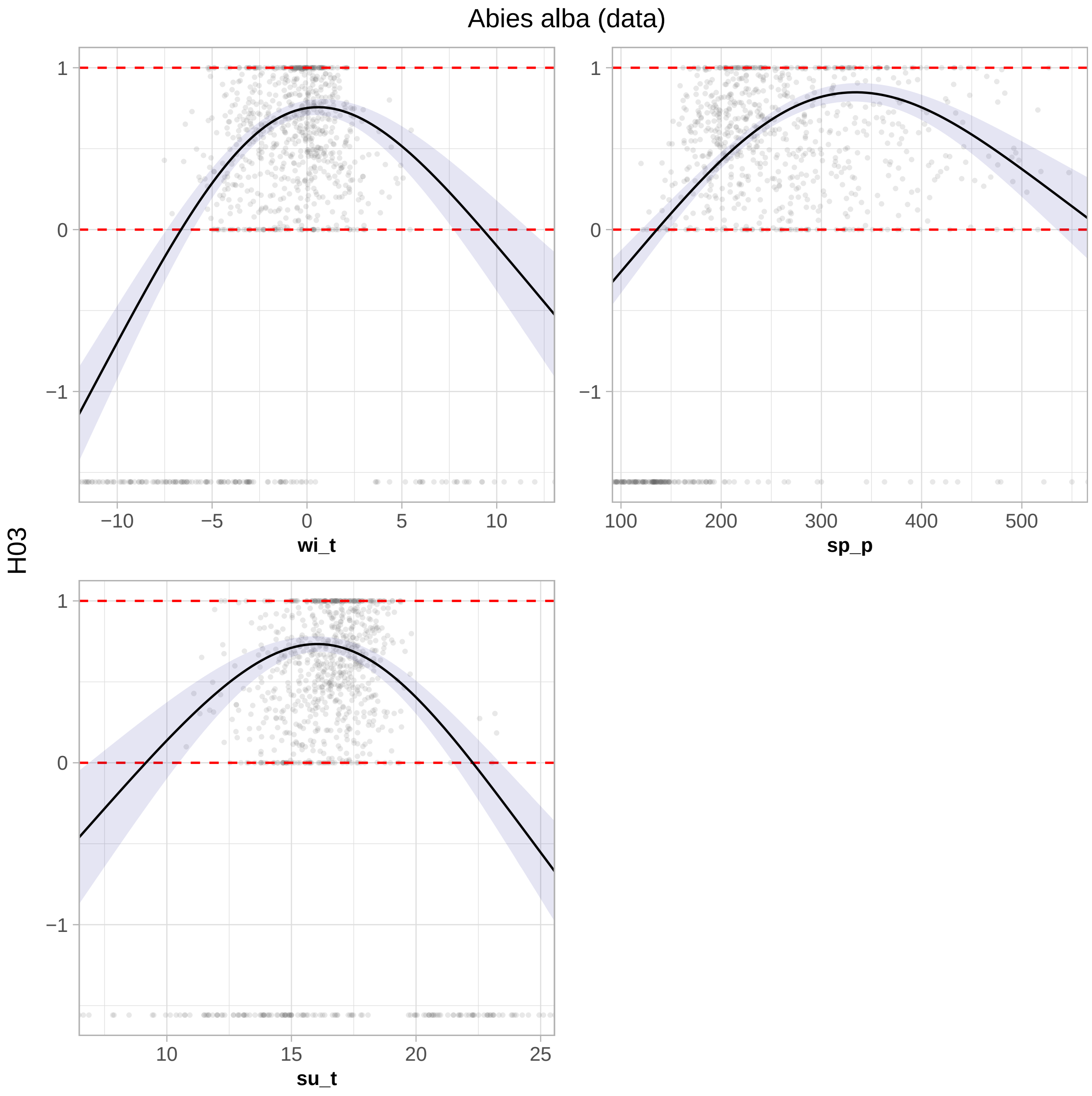

Response maps

Response maps (also referred as partial effect maps). Each map visualizes how a predictor affect the target variable (top height [m] at Age 100). Technically their work like response curves in a geographical area, that is setting all predictor variables except the one shown in the figure on their mean, and mapping the prediction. Predictor acronyms: Bio.1 = Mean annual temperature [°C], Bio.12 = Annual precipitation sum [mm/m2], sp_p = Sum of precipitation [mm/m2] within months 3 to 5, su_p = Sum of precipitation [mm/m2] within months 6 to 8, wi_p = Sum of precipitation [mm/m2] within months 12,1,2, sp_t = Mean temperature [°C] within months 3 to 5, su_t = Mean temperature [°C] within months 6 to 8, wi_t = Mean temperature [°C] within months 12,1,2.

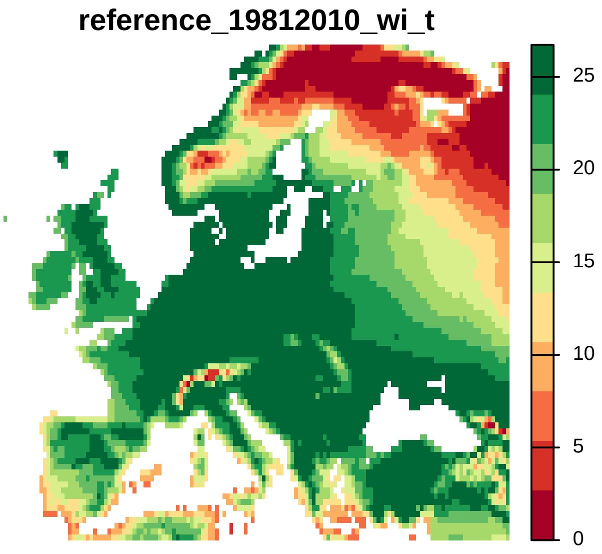

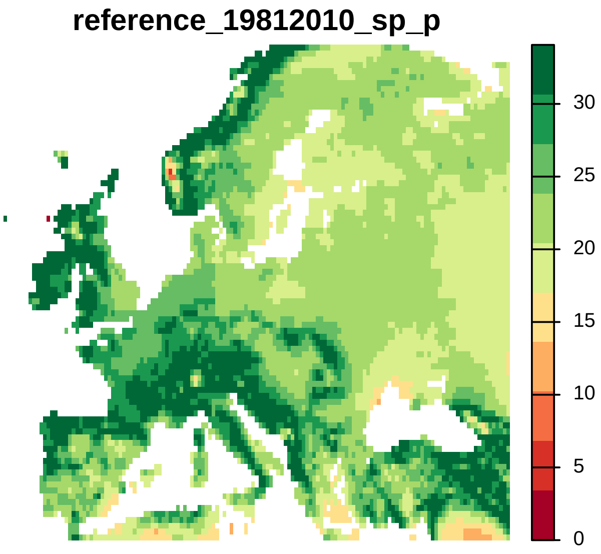

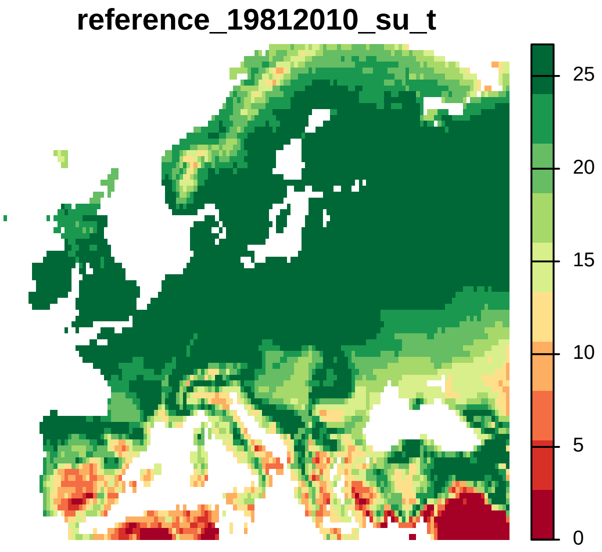

Residual distribution

The multi-panel plot includes a histogram of the residuals (top left), residuals over fitted values (top right), a histogram of observed and predicted values (bottom left) and boxplot diagram of observed and predicted values (bottom right). Observed values are shown in light green, while predicted ones are depicted in light red.

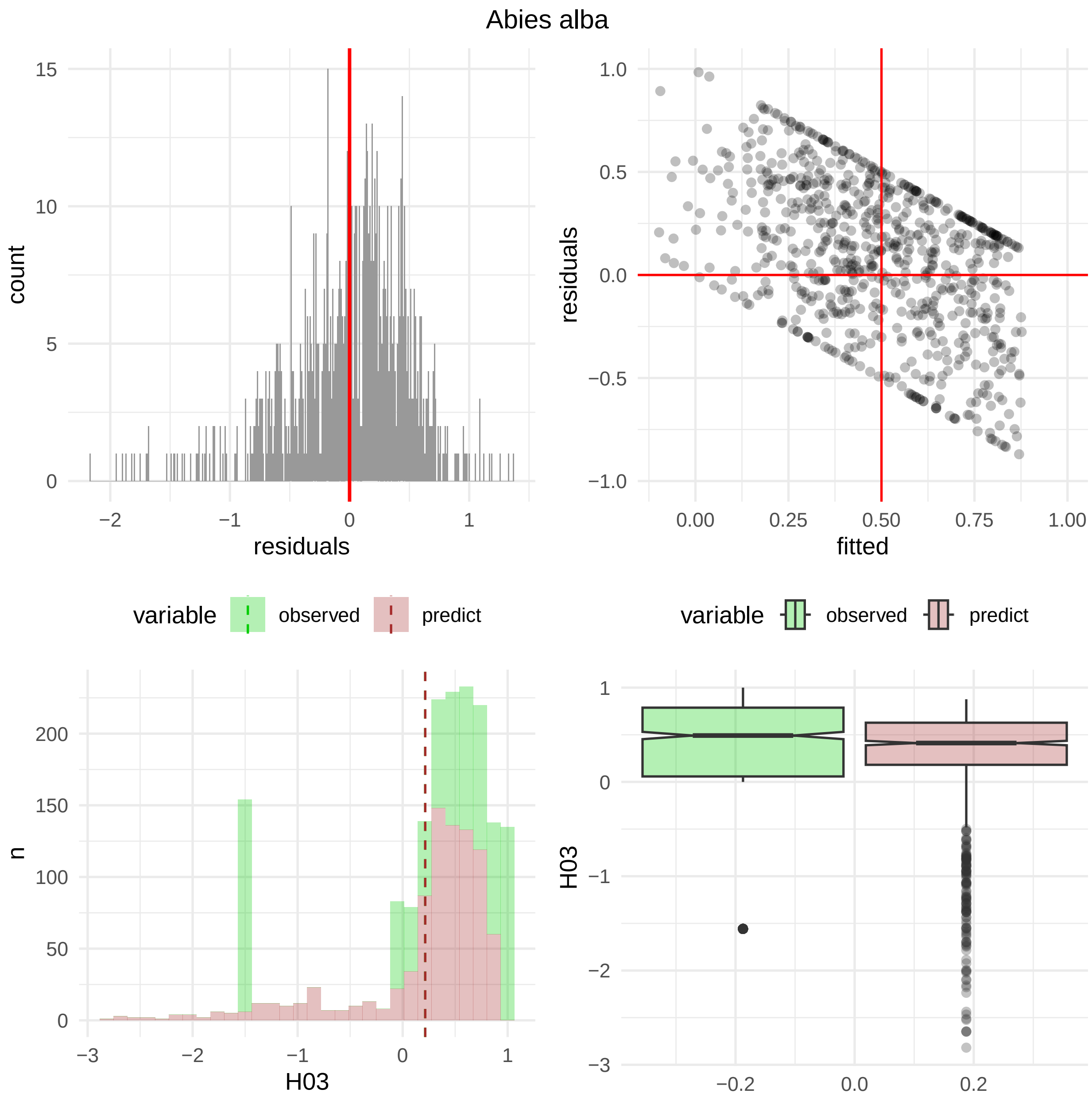

Correlation between predict and observed site index

Relationship between predicted and observed site index (density cloud), as well as linear regressions of presences and absences (= 'growth absences') (red line) and presences only (magenta line). The formulas, significance, R2 and number of observations are displayed below for both regressions. Ideally, both the point cloud and the regression lines lie close to the dashed line. For presences only we additionally calculated the correlation coefficient according to PEARSON (cor.pre) in the bottom right corner.

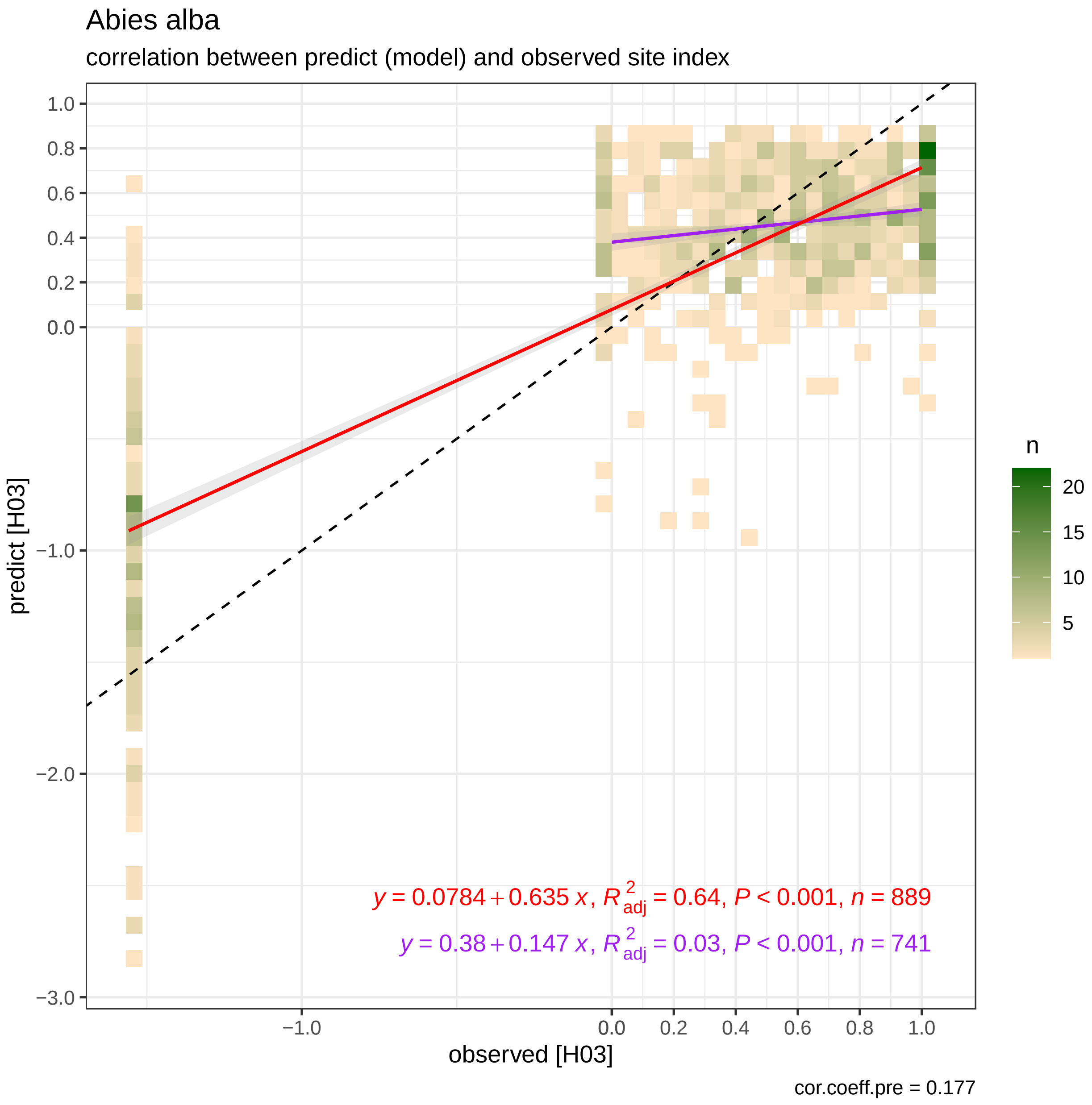

Predictions and forecasts

Predict

European predict for the reference period (1981 to 2010). Dark green symbolizes a high site index (tree height in meters at age 100), orange a lower site index and red no growth. Magenta-coloured dots represent inventory points with growth information, light blue dots are absences (= 'growth absences'). Results were aggregated on 25 km x 25 km scale.

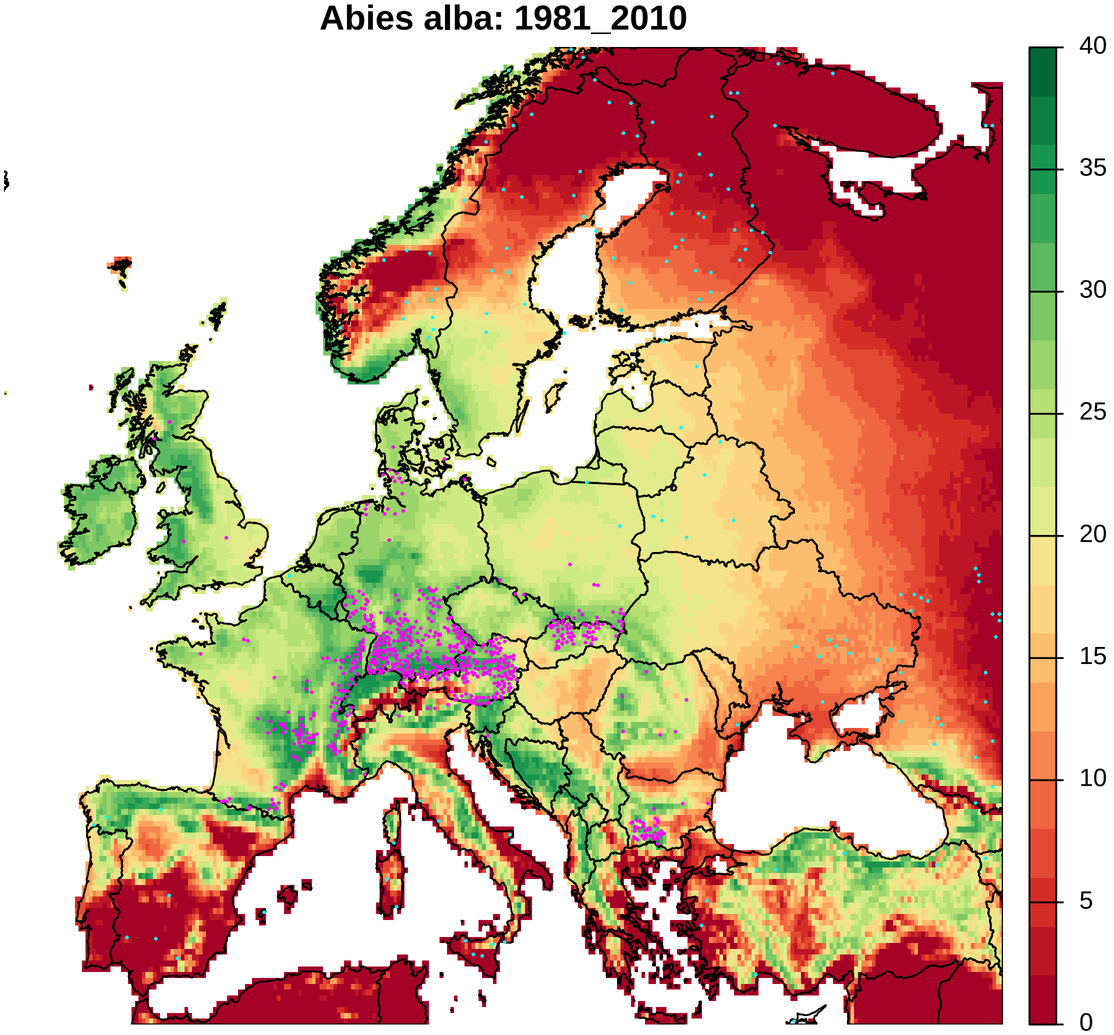

Forecast

Prediction for the reference period (1981 to 2010), as well as forecasts to 2071 to 2100 under szenario RCP4.5 and RCP8.5. Dark green symbolizes a high site index (tree height in m at age 100), orange a lower site index and red no growth. Results were aggregated on 25 km x 25 km scale.

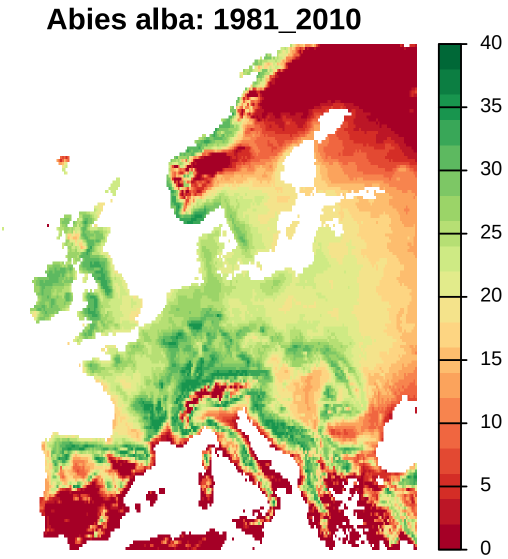

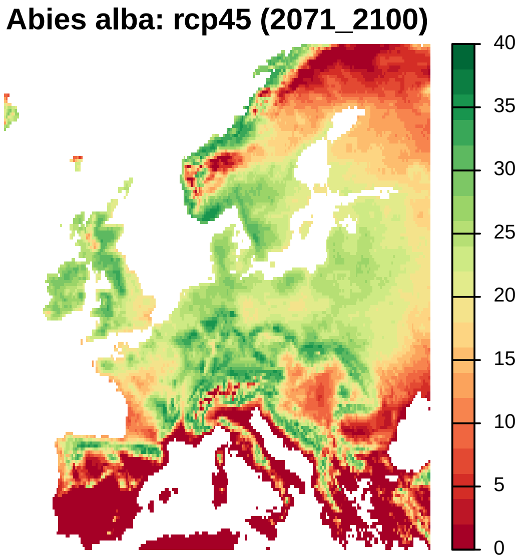

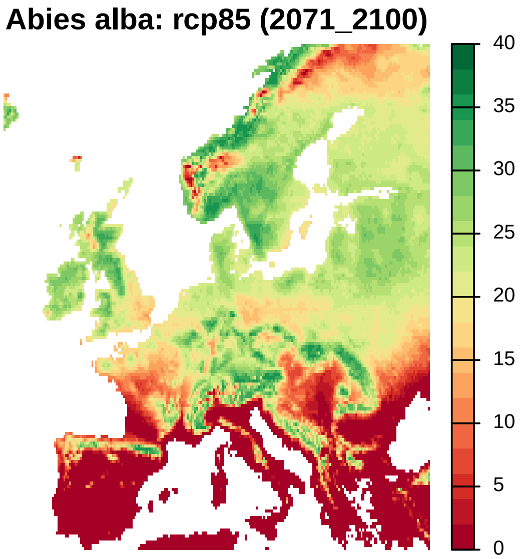

Abies grandis

Site index curves

Site index curves of Abies grandis created with non-linear quantile regressions based on the algorithm of Koenker and Park (1992). The site index (SI) was created by setting all points on the 95 percent quantile (upper line) and above to one (SI = 1) and all on the 5 percent quantile (lower line) and below to zero (SI = 0). The points between the quantile boundaries were assigned a site index between zero and one according to the ratio of their position between the quantile boundaries. We set selected absences (see chapter 2.1.3) on Height = 0 m (at age 100), which means, depending on the site index curves, for each tree species a SI near -1 (red line).

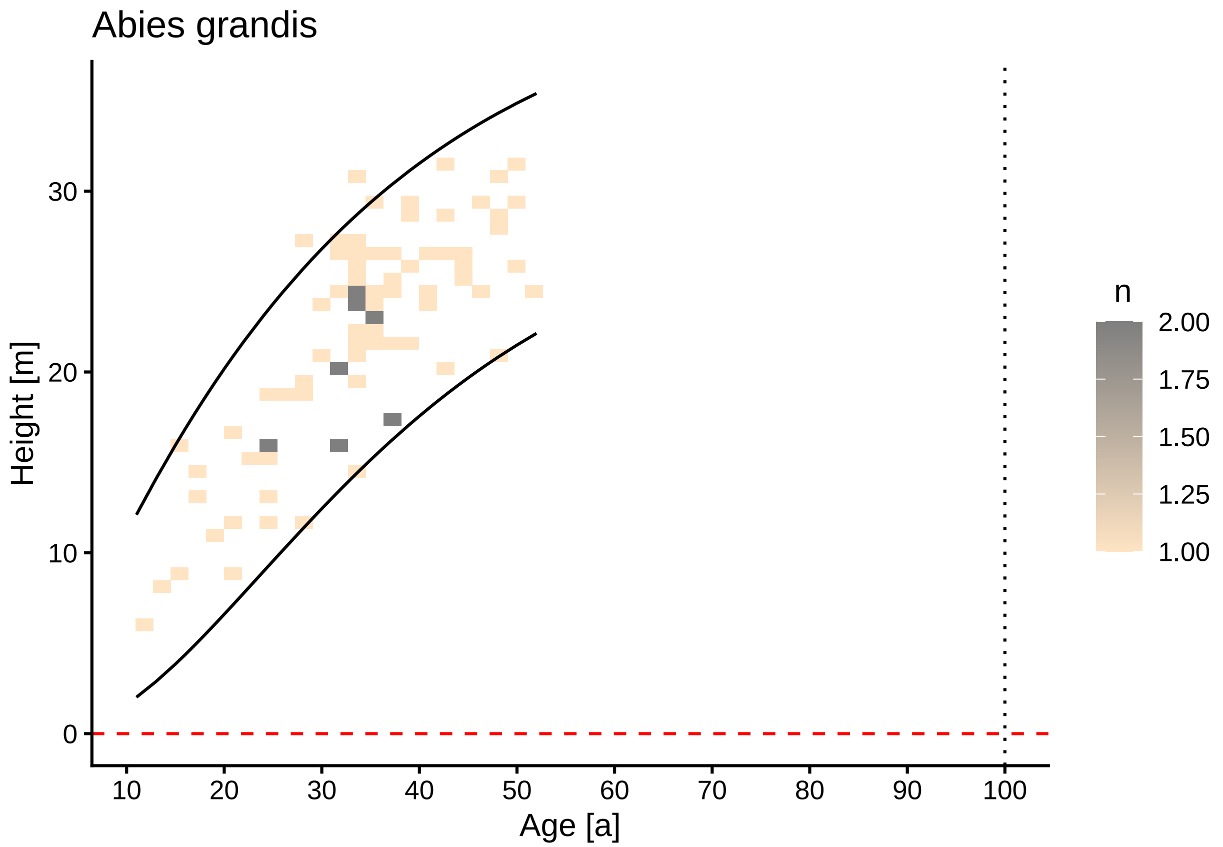

Model statistics and evaluation

Summary

Predictor acronyms: Bio.1 = Mean annual temperature [°C], Bio.12 = Annual precipitation sum [mm/m2], sp__p = Sum of precipitation [mm/m2] within months 3 to 5, su__p = Sum of precipitation [mm/m2] within months 6 to 8, wi__p = Sum of precipitation [mm/m2] within months 12,1,2, sp__t = Mean temperature [°C] within months 3 to 5, su__t = Mean temperature [°C] within months 6 to 8, wi__t = Mean temperature [°C] within months 12,1,2.

```
##
## Family: gaussian
## Link function: identity
##
## Formula:
## H03 ~ s(reference_19812010_su_t, k = 3) + s(reference_19812010_wi_p,
##     k = 3)
##
## Parametric coefficients:
##             Estimate Std. Error t value Pr(>|t|)
## (Intercept)  0.07412    0.08843   0.838    0.404
##
## Approximate significance of smooth terms:
##                              edf Ref.df      F  p-value
## s(reference_19812010_su_t) 1.970  1.998 16.484 1.65e-06 ***
## s(reference_19812010_wi_p) 1.879  1.984  4.179   0.0224 *
## ---
## Signif. codes:  0 '***' 0.001 '**' 0.01 '*' 0.05 '.' 0.1 ' ' 1
##
## R-sq.(adj) =  0.457   Deviance explained = 48.2%
## -REML = 109.15  Scale est. = 0.6647    n = 85
```

Variance inflation factor (VIF)

Predictor acronyms: Bio.1 = Mean annual temperature [°C], Bio.12 = Annual precipitation sum [mm/m2], sp__p = Sum of precipitation [mm/m2] within months 3 to 5, su__p = Sum of precipitation [mm/m2] within months 6 to 8, wi__p = Sum of precipitation [mm/m2] within months 12,1,2, sp__t = Mean temperature [°C] within months 3 to 5, su__t = Mean temperature [°C] within months 6 to 8, wi__t = Mean temperature [°C] within months 12,1,2.

```
##                  Variables      VIF
## 1 reference_19812010_su_t 1.126028
## 2 reference_19812010_wi_p 1.126028
```

Correlation matrix

Correlation matrix between the predictor variables and the target variable in the model. Correlation coefficient according to PEARSON. Predictor acronyms: Bio.1 = Mean annual temperature [°C], Bio.12 = Annual precipitation sum [mm/m2], sp_p = Sum of precipitation [mm/m2] within months 3 to 5, su_p = Sum of precipitation [mm/m2] within months 6 to 8, wi_p = Sum of precipitation [mm/m2] within months 12,1,2, sp_t = Mean temperature [°C] within months 3 to 5, su_t = Mean temperature [°C] within months 6 to 8, wi_t = Mean temperature [°C] within months 12,1,2.

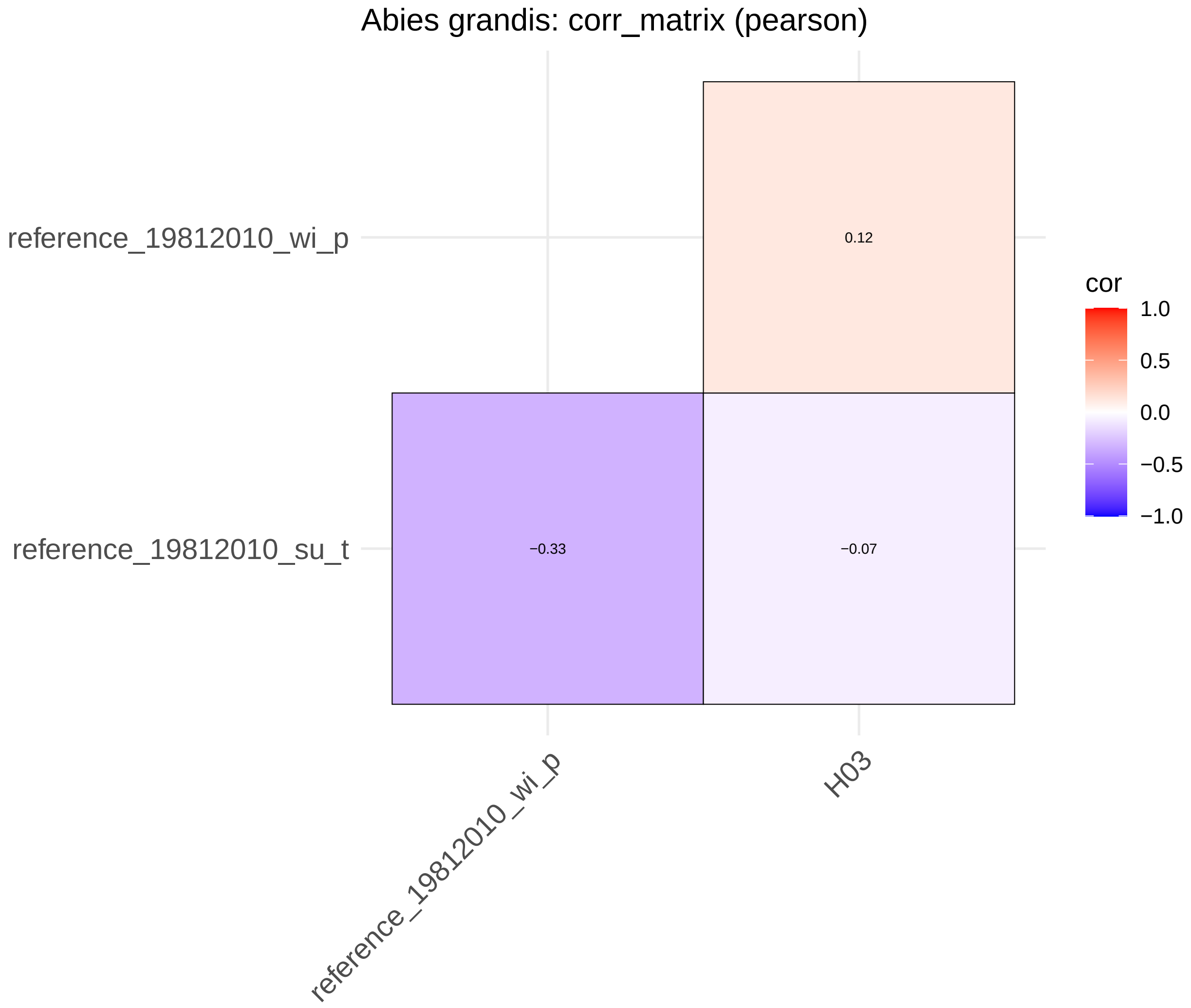

Response curves

Response curves (also known as effect curves) show how each predictor variable affects the target variable (H03 = european Site index, SIrel). H03 values below zero represent 'Growth absences'. Predictor acronyms: Bio.1 = Mean annual temperature [°C], Bio.12 = Annual precipitation sum [mm/m2], sp_p = Sum of precipitation [mm/m2] within months 3 to 5, su_p = Sum of precipitation [mm/m2] within months 6 to 8, wi_p = Sum of precipitation [mm/m2] within months 12,1,2, sp_t = Mean temperature [°C] within months 3 to 5, su_t = Mean temperature [°C] within months 6 to 8, wi_t = Mean temperature [°C] within months 12,1,2.

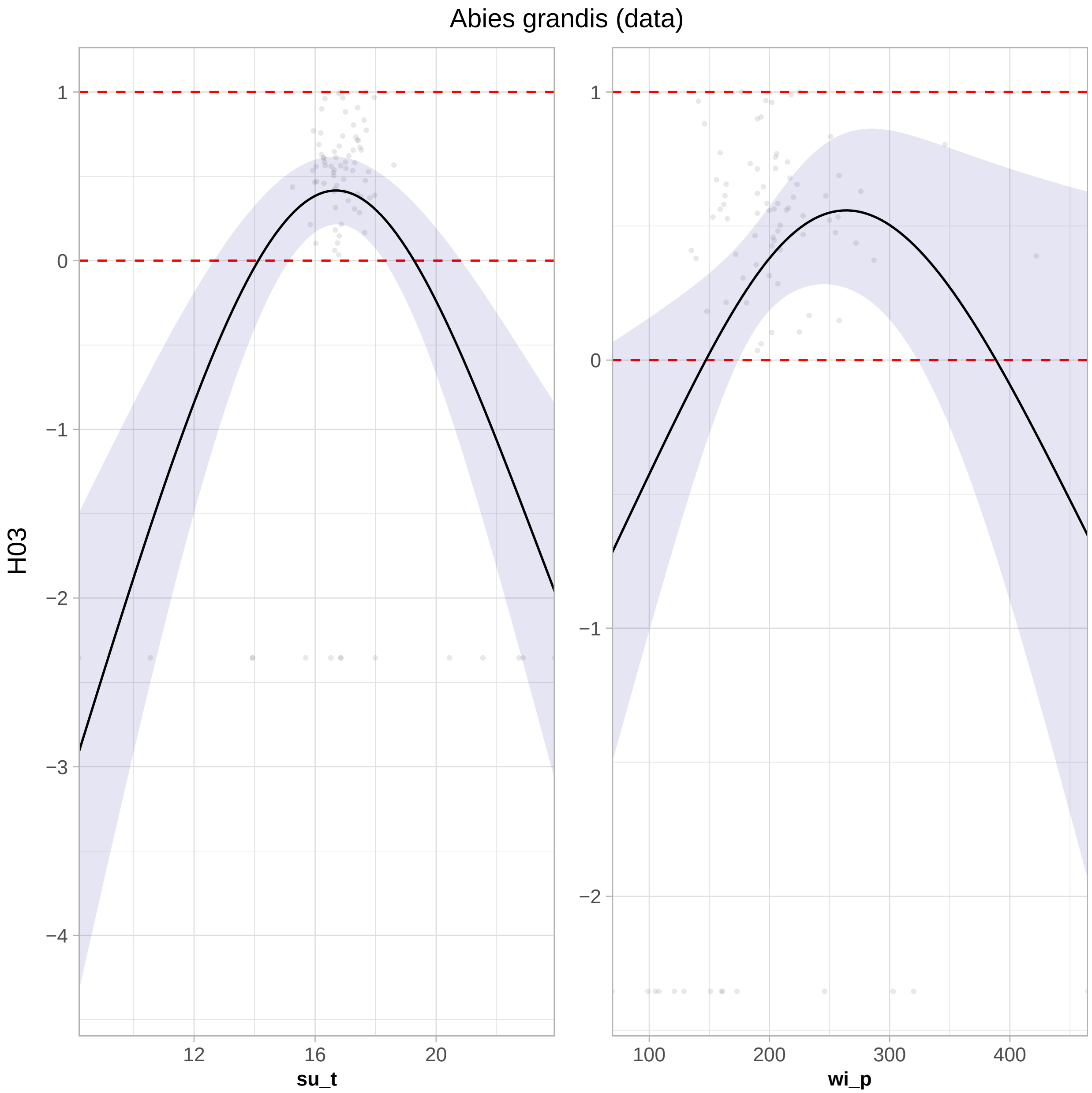

Response maps

Response maps (also referred as partial effect maps). Each map visualizes how a predictor affect the target variable (top height [m] at Age 100). Technically their work like response curves in a geographical area, that is setting all predictor variables except the one shown in the figure on their mean, and mapping the prediction. Predictor acronyms: Bio.1 = Mean annual temperature [°C], Bio.12 = Annual precipitation sum [mm/m2], sp_p = Sum of precipitation [mm/m2] within months 3 to 5, su_p = Sum of precipitation [mm/m2] within months 6 to 8, wi_p = Sum of precipitation [mm/m2] within months 12,1,2, sp_t = Mean temperature [°C] within months 3 to 5, su_t = Mean temperature [°C] within months 6 to 8, wi_t = Mean temperature [°C] within months 12,1,2.

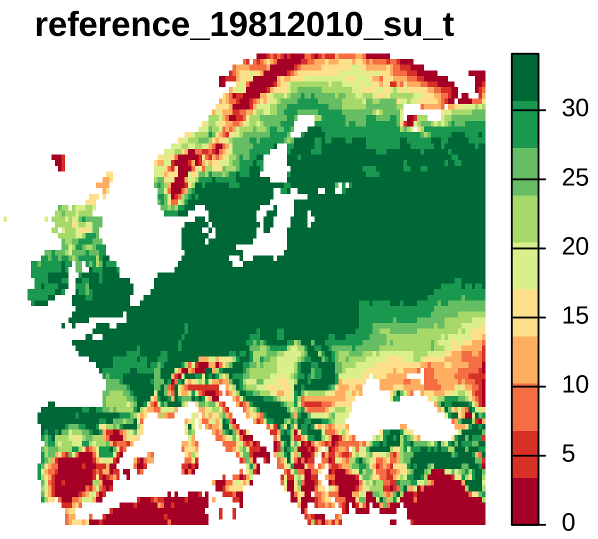

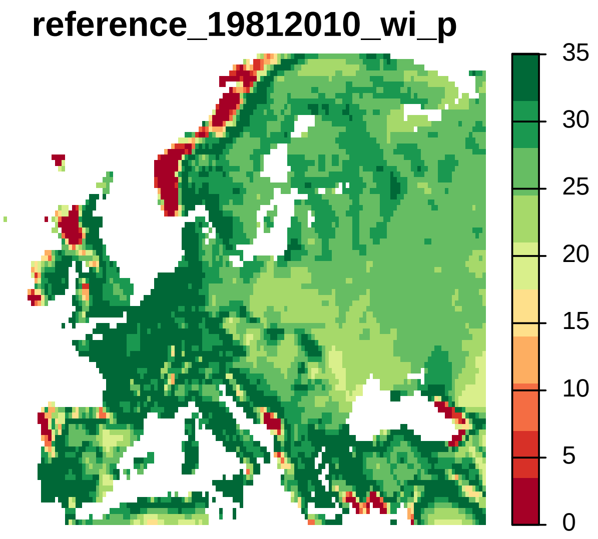

Residual distribution

The multi-panel plot includes a histogram of the residuals (top left), residuals over fitted values (top right), a histogram of observed and predicted values (bottom left) and boxplot diagram of observed and predicted values (bottom right). Observed values are shown in light green, while predicted ones are depicted in light red.

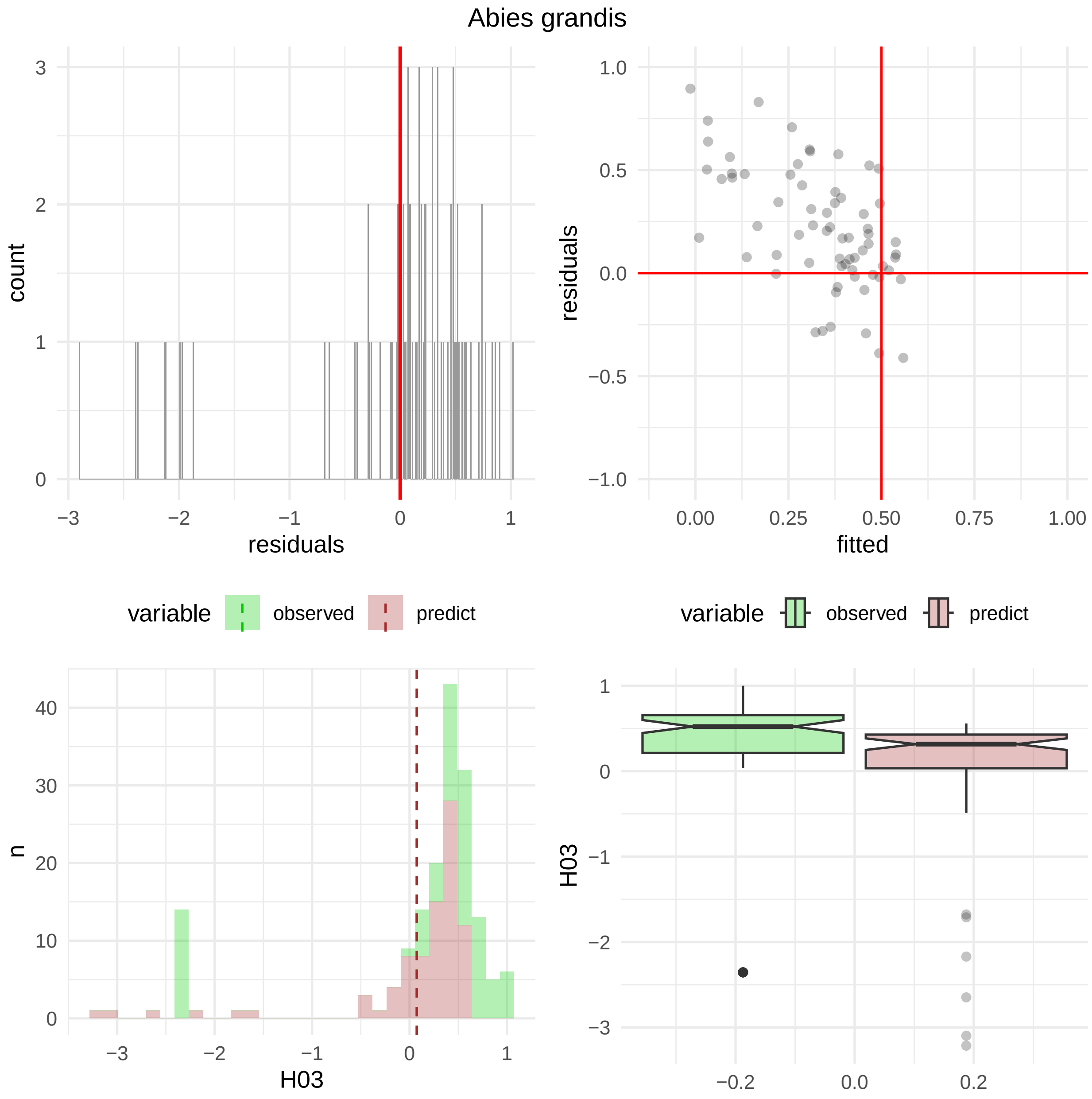

Correlation between predict and observed site index

Relationship between predicted and observed site index (density cloud), as well as linear regressions of presences and absences (= 'growth absences') (red line) and presences only (magenta line). The formulas, significance, R2 and number of observations are displayed below for both regressions. Ideally, both the point cloud and the regression lines lie close to the dashed line. For presences only we additionally calculated the correlation coefficient according to PEARSON (cor.pre) in the bottom right corner.

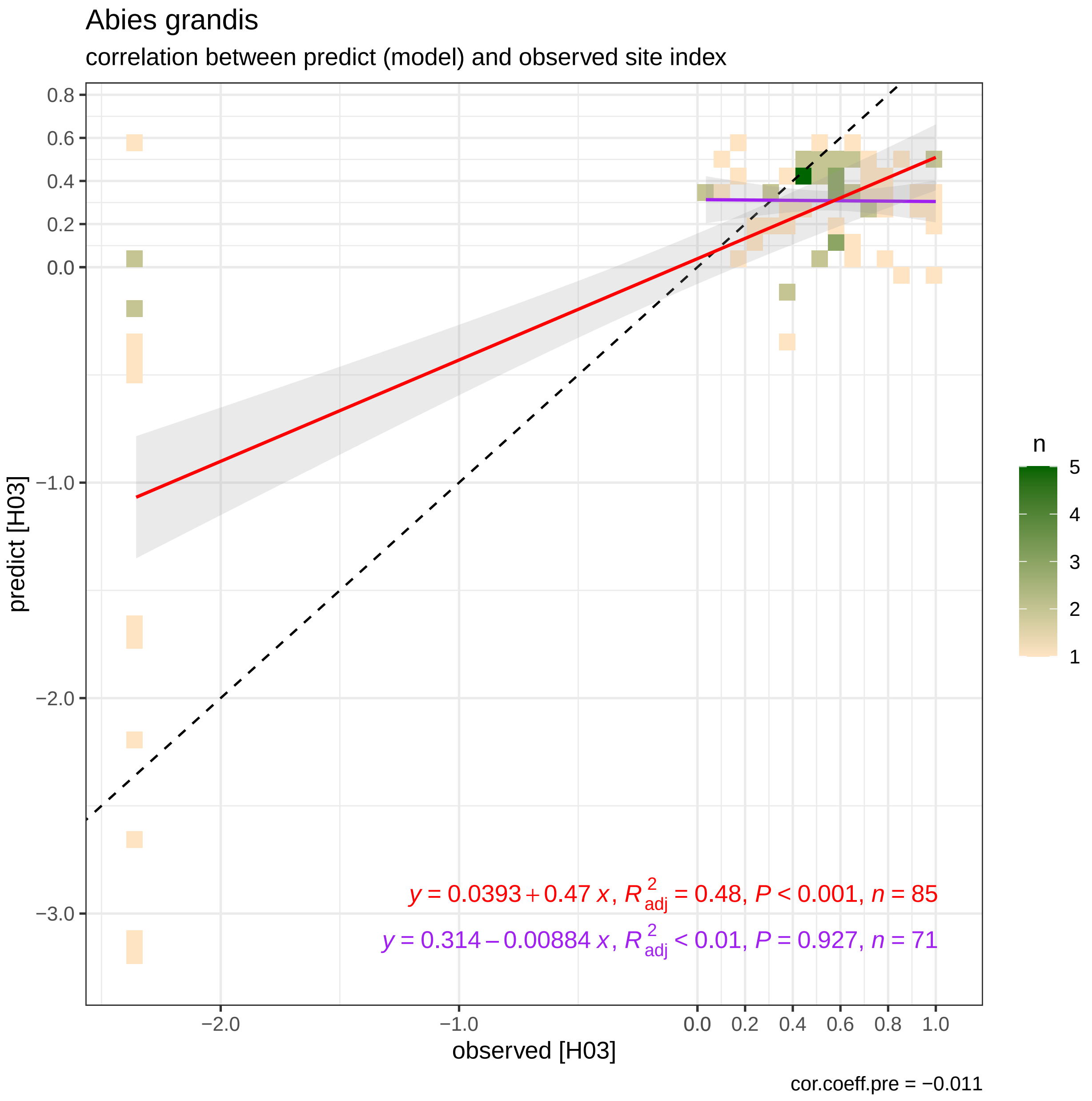

Predictions and forecasts

Predict

European predict for the reference period (1981 to 2010). Dark green symbolizes a high site index (tree height in meters at age 100), orange a lower site index and red no growth. Magenta-coloured dots represent inventory points with growth information, light blue dots are absences (= ‘growth absences’). Results were aggregated on 25 km x 25 km scale.

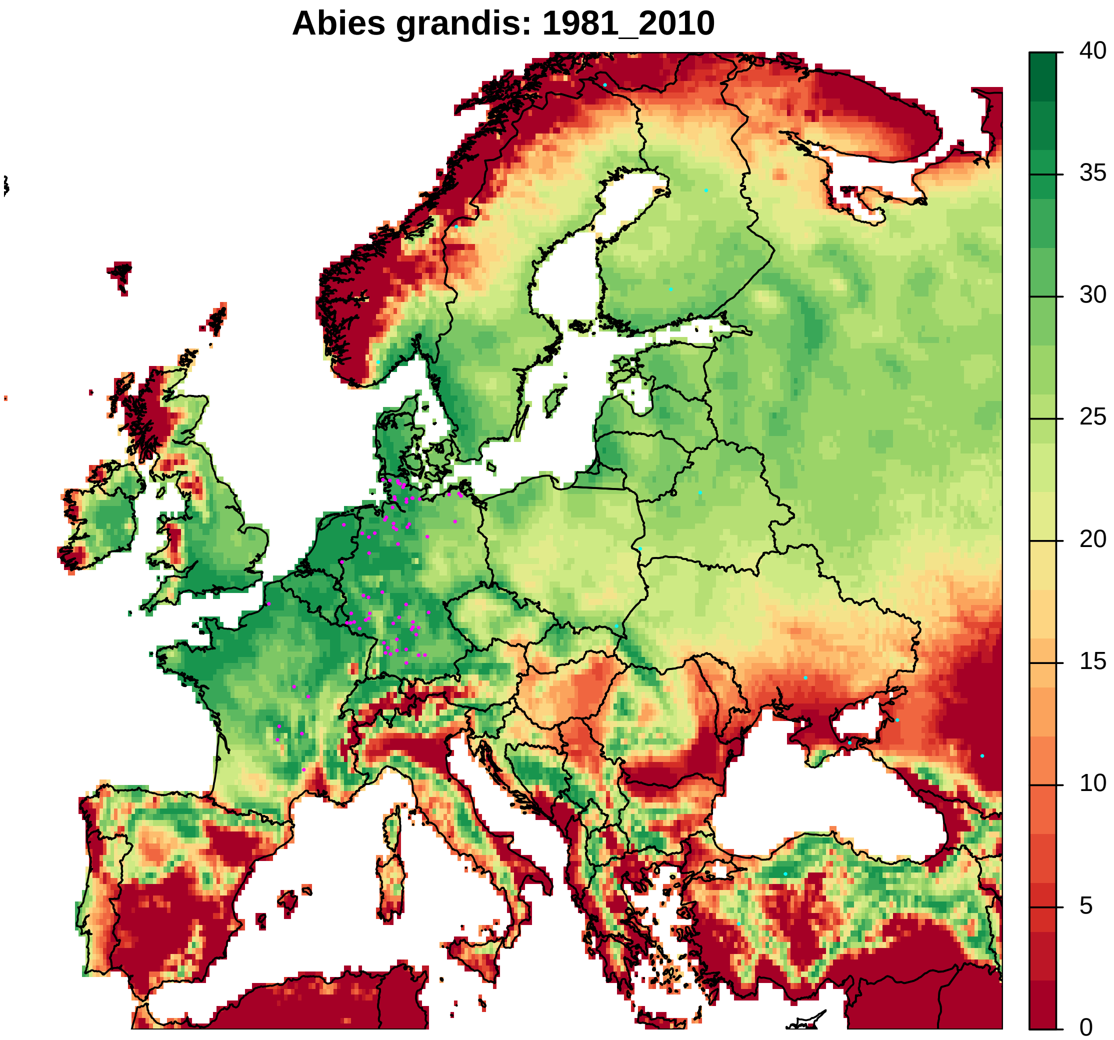

Forecast

Prediction for the reference period (1981 to 2010), as well as forecasts to 2071 to 2100 under szenario RCP4.5 and RCP8.5. Dark green symbolizes a high site index (tree height in m at age 100), orange a lower site index and red no growth. Results were aggregated on 25 km x 25 km scale.

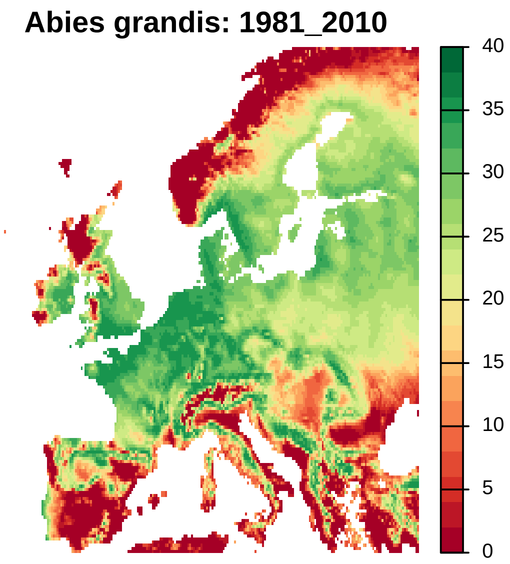

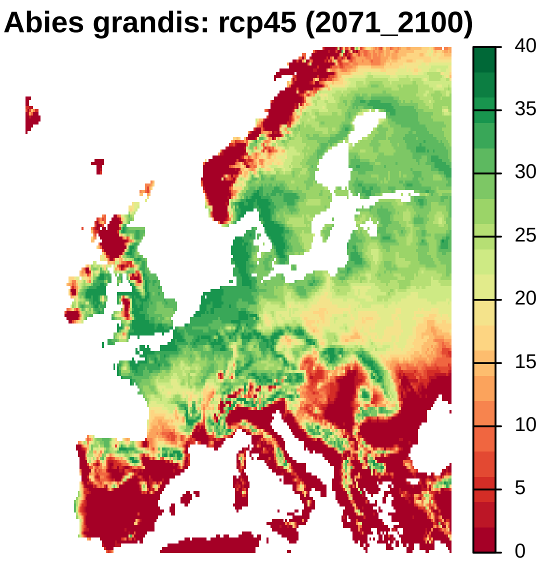

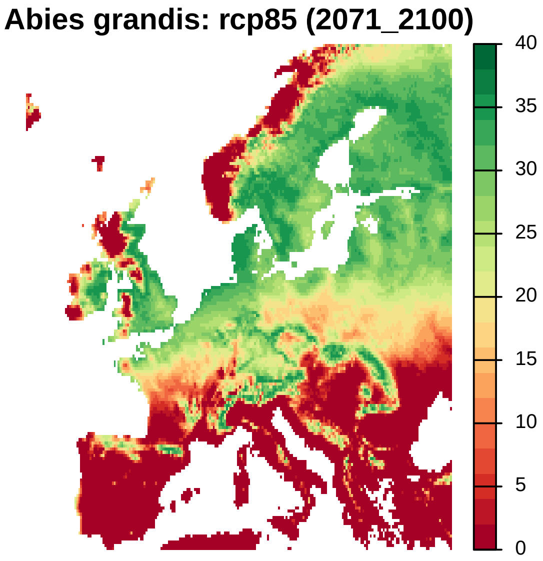

Acer campestre

Site index curves

Site index curves of Acer campestre created with non-linear quantile regressions based on the algorithm of Koenker and Park (1992). The site index (SI) was created by setting all points on the 95 percent quantile (upper line) and above to one (SI = 1) and all on the 5 percent quantile (lower line) and below to zero (SI = 0). The points between the quantile boundaries were assigned a site index between zero and one according to the ratio of their position between the quantile boundaries. We set selected absences (see chapter 2.1.3) on Height = 0 m (at age 100), which means, depending on the site index curves, for each tree species a SI near -1 (red line).

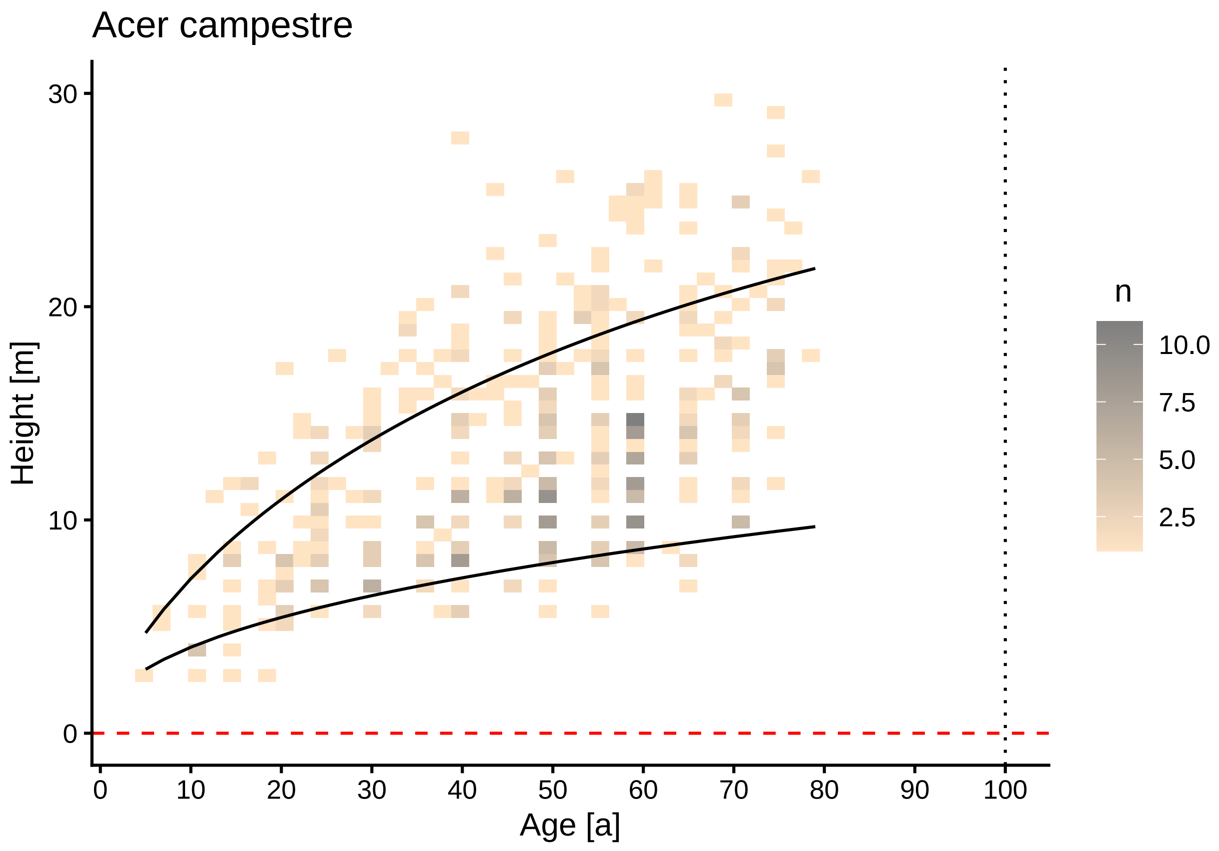

Model statistics and evaluation

Summary

Predictor acronyms: Bio.1 = Mean annual temperature [°C], Bio.12 = Annual precipitation sum [mm/m2], sp_p = Sum of precipitation [mm/m2] within months 3 to 5, su_p = Sum of precipitation [mm/m2] within months 6 to 8, wi_p = Sum of precipitation [mm/m2] within months 12,1,2, sp_t = Mean temperature [°C] within months 3 to 5, su_t = Mean temperature [°C] within months 6 to 8, wi_t = Mean temperature [°C] within months 12,1,2.

```
##
## Family: gaussian
## Link function: identity
##
## Formula:
## H03 ~ s(reference_19812010_sp_p, k = 3) + s(reference_19812010_Bio.1,
##     k = 3)
##
## Parametric coefficients:
##             Estimate Std. Error t value Pr(>|t|)
## (Intercept)  0.47449    0.02864   16.57   <2e-16 ***
## ---
## Signif. codes:  0 '***' 0.001 '**' 0.01 '*' 0.05 '.' 0.1 ' ' 1
##
## Approximate significance of smooth terms:
##                               edf Ref.df     F p-value
## s(reference_19812010_sp_p)  1.987  2.000 39.10  <2e-16 ***
## s(reference_19812010_Bio.1) 1.973  1.999 66.69  <2e-16 ***
## ---
## Signif. codes:  0 '***' 0.001 '**' 0.01 '*' 0.05 '.' 0.1 ' ' 1
##
## R-sq.(adj) =  0.458   Deviance explained = 46.5%
## -REML = 195.79  Scale est. = 0.22549   n = 275
```

Variance inflation factor (VIF)

Predictor acronyms: Bio.1 = Mean annual temperature [°C], Bio.12 = Annual precipitation sum [mm/m2], sp_p = Sum of precipitation [mm/m2] within months 3 to 5, su_p = Sum of precipitation [mm/m2] within months 6 to 8, wi_p = Sum of precipitation [mm/m2] within months 12,1,2, sp_t = Mean temperature [°C] within months 3 to 5, su_t = Mean temperature [°C] within months 6 to 8, wi_t = Mean temperature [°C] within months 12,1,2.

```
##                   Variables      VIF
## 1  reference_19812010_sp_p 1.003757
## 2 reference_19812010_Bio.1 1.003757
```

Correlation matrix

Correlation matrix between the predictor variables and the target variable in the model. Correlation coefficient according to PEARSON. Predictor acronyms: Bio.1 = Mean annual temperature [°C], Bio.12 = Annual precipitation sum [mm/m2], sp_p = Sum of precipitation [mm/m2] within months 3 to 5, su_p = Sum of precipitation [mm/m2] within months 6 to 8, wi_p = Sum of precipitation [mm/m2] within months 12,1,2, sp_t = Mean temperature [°C] within months 3 to 5, su_t = Mean temperature [°C] within months 6 to 8, wi_t = Mean temperature [°C] within months 12,1,2.

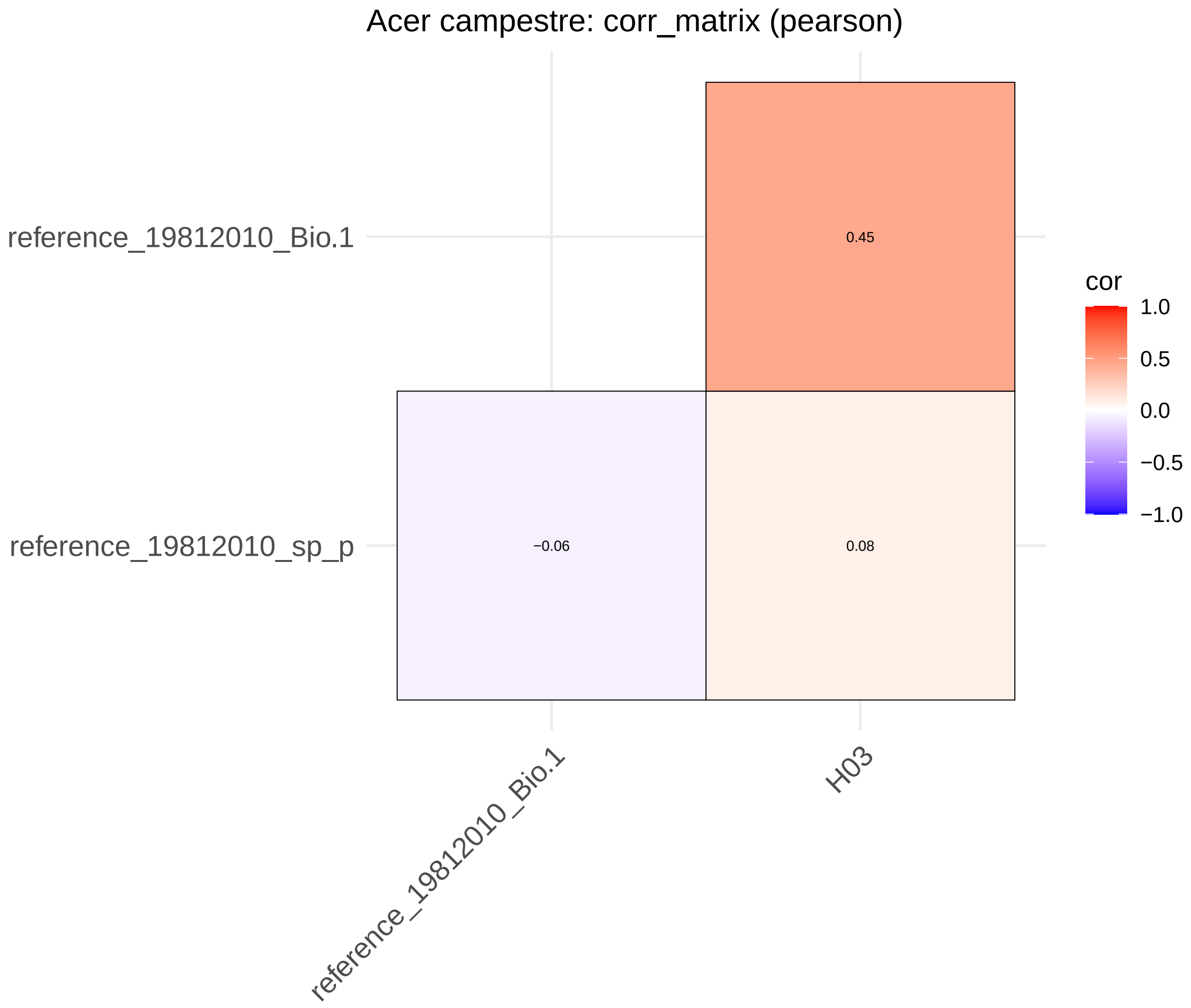

Response curves

Response curves (also known as effect curves) show how each predictor variable affects the target variable (H03 = european Site index, SIrel). H03 values below zero represent 'Growth absences'. Predictor acronyms: Bio.1 = Mean annual temperature [°C], Bio.12 = Annual precipitation sum [mm/m2], sp_p = Sum of precipitation [mm/m2] within months 3 to 5, su_p = Sum of precipitation [mm/m2] within months 6 to 8, wi_p = Sum of precipitation [mm/m2] within months 12,1,2, sp_t = Mean temperature [°C] within months 3 to 5, su_t = Mean temperature [°C] within months 6 to 8, wi_t = Mean temperature [°C] within months 12,1,2.

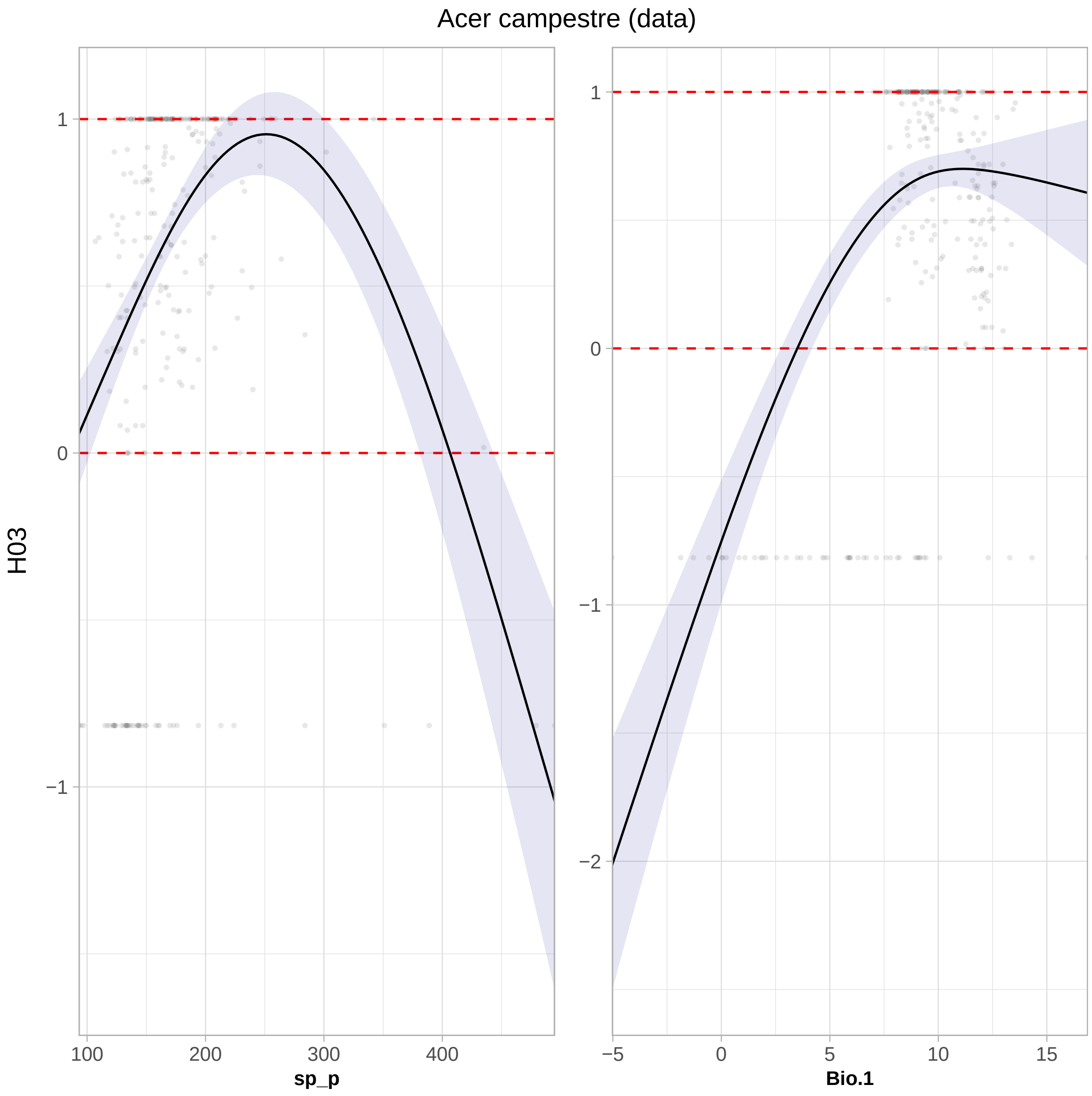

Response maps

Response maps (also referred as partial effect maps). Each map visualizes how a predictor affect the target variable (top height [m] at Age 100). Technically their work like response curves in a geographical area, that is setting all predictor variables except the one shown in the figure on their mean, and mapping the prediction. Predictor acronyms: Bio.1 = Mean annual temperature [°C], Bio.12 = Annual precipitation sum [mm/m2], sp_p = Sum of precipitation [mm/m2] within months 3 to 5, su_p = Sum of precipitation [mm/m2] within months 6 to 8, wi_p = Sum of precipitation [mm/m2] within months 12,1,2, sp_t = Mean temperature [°C] within months 3 to 5, su_t = Mean temperature [°C] within months 6 to 8, wi_t = Mean temperature [°C] within months 12,1,2.

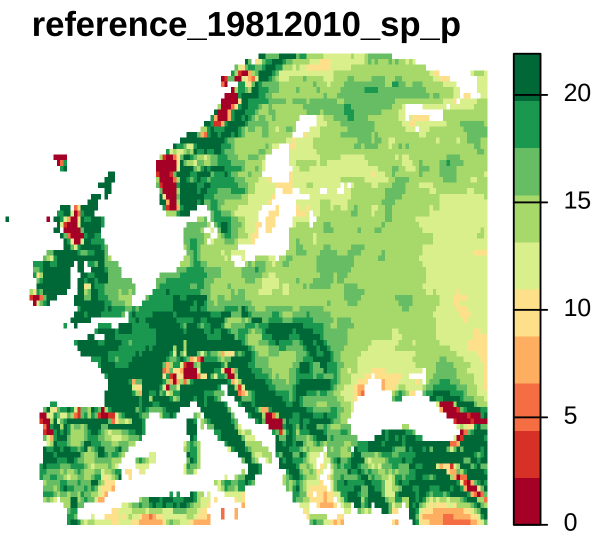

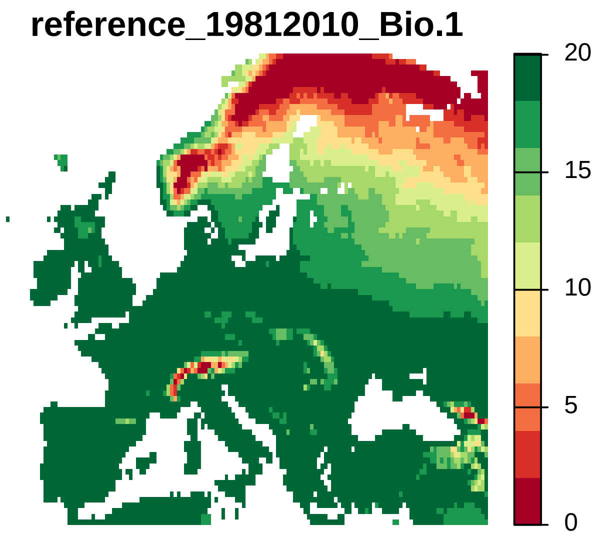

Residual distribution

The multi-panel plot includes a histogram of the residuals (top left), residuals over fitted values (top right), a histogram of observed and predicted values (bottom left) and boxplot diagram of observed and predicted values (bottom right). Observed values are shown in light green, while predicted ones are depicted in light red.

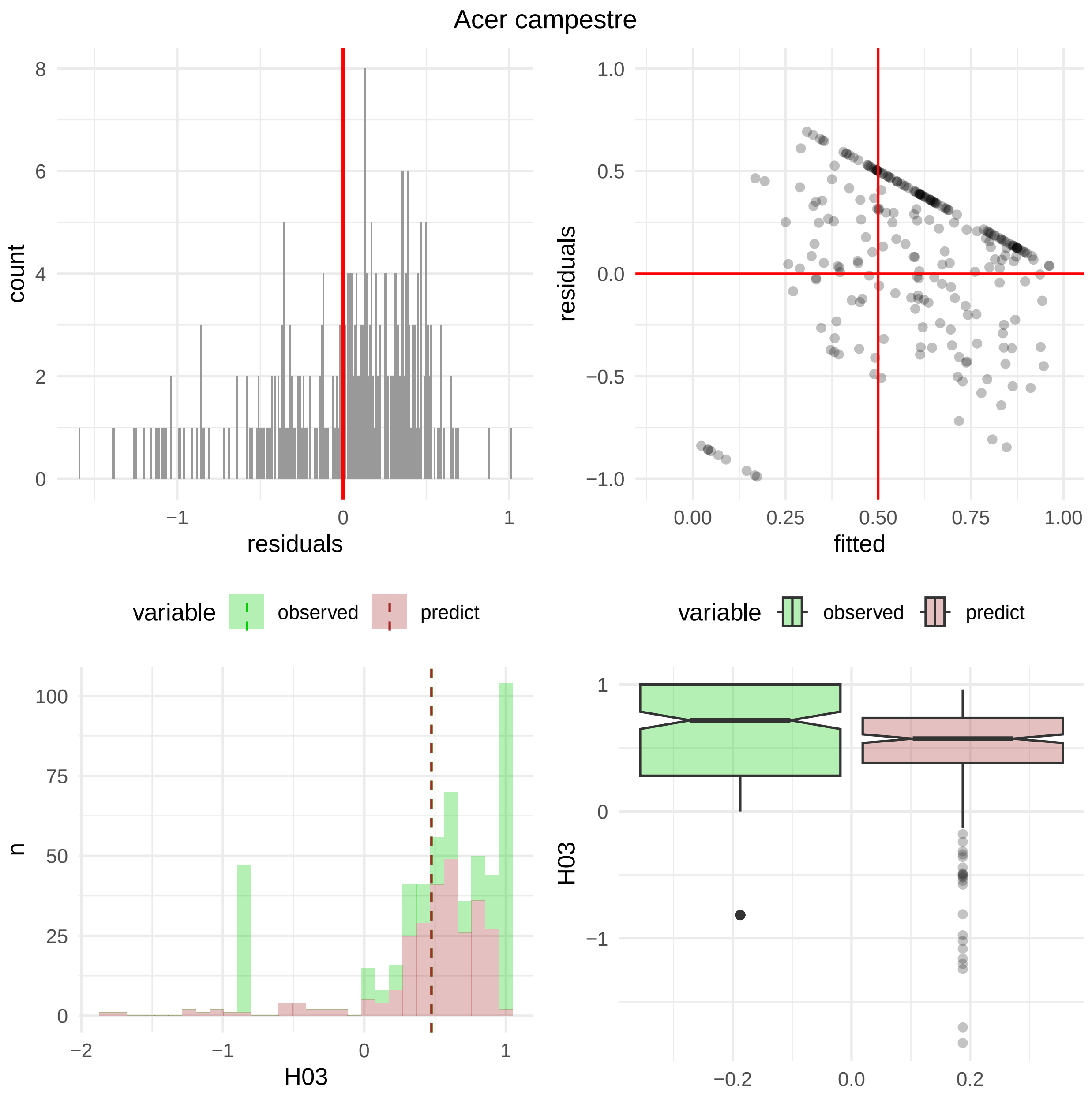

Correlation between predict and observed site index

Relationship between predicted and observed site index (density cloud), as well as linear regressions of presences and absences (= 'growth absences') (red line) and presences only (magenta line). The formulas, significance, R2 and number of observations are displayed below for both regressions. Ideally, both the point cloud and the regression lines lie close to the dashed line. For presences only we additionally calculated the correlation coefficient according to PEARSON (cor.pre) in the bottom right corner.

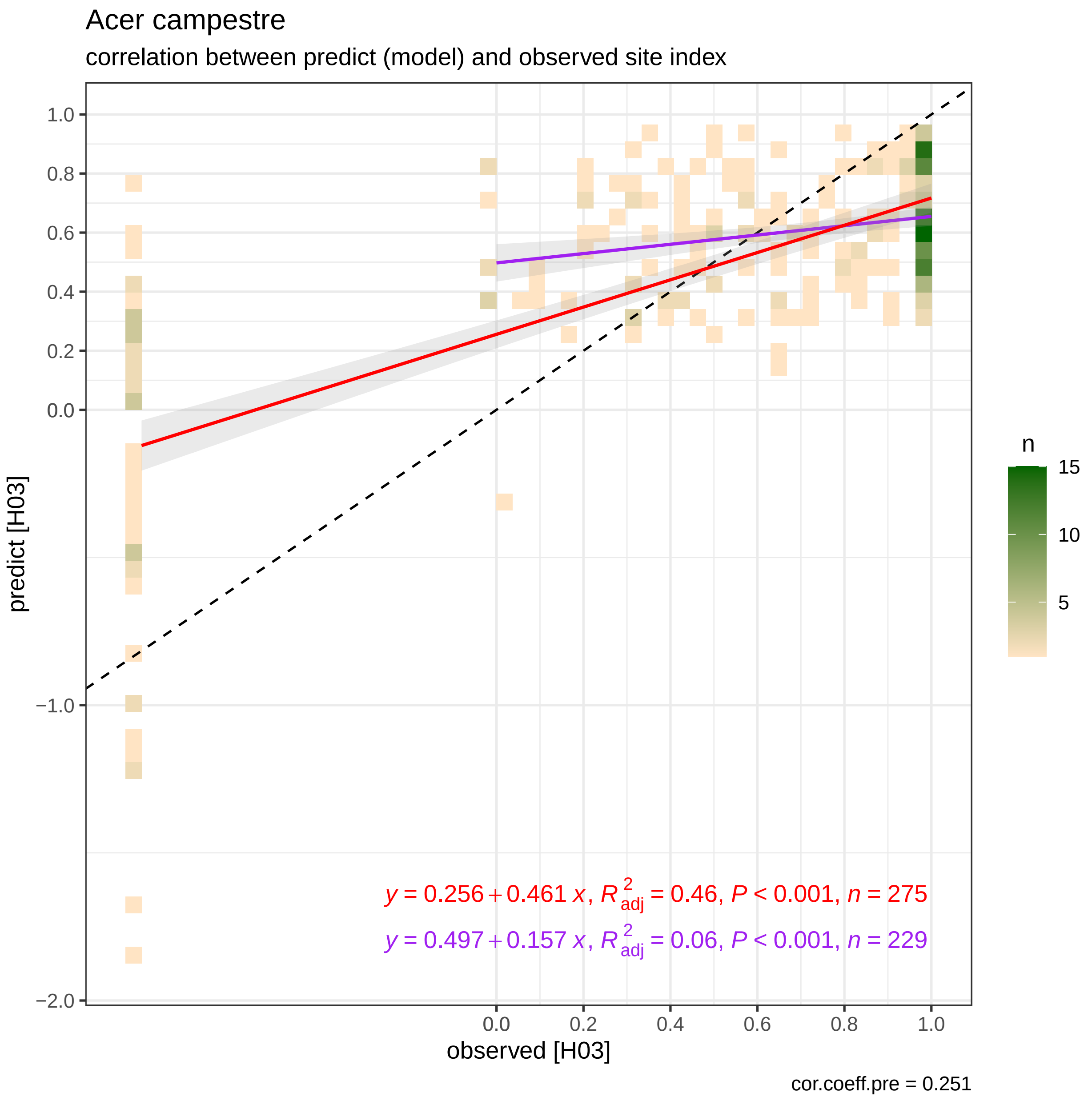

Predictions and forecasts

Predict

European predict for the reference period (1981 to 2010). Dark green symbolizes a high site index (tree height in meters at age 100), orange a lower site index and red no growth. Magenta-coloured dots represent inventory points with growth information, light blue dots are absences (= 'growth absences'). Results were aggregated on 25 km x 25 km scale.

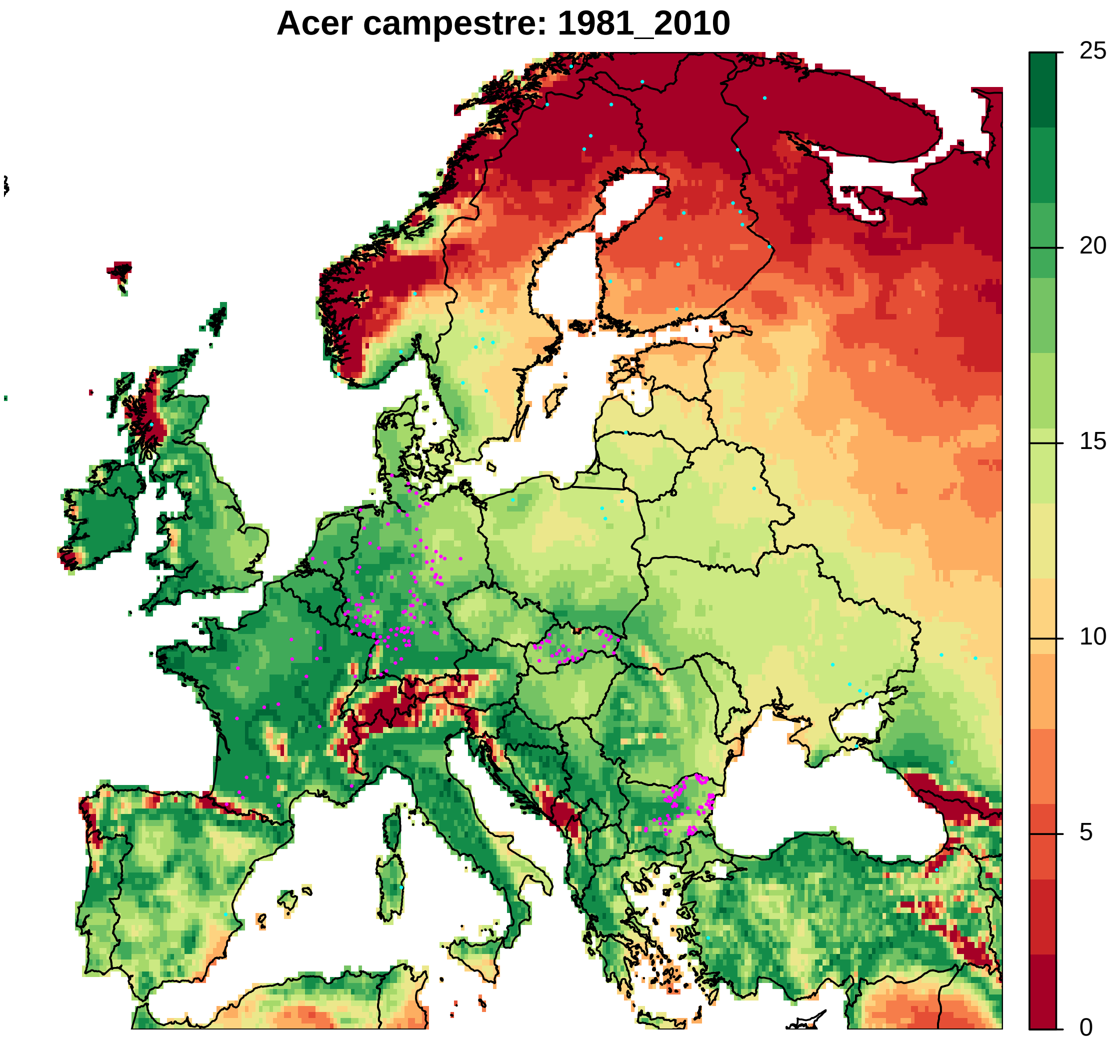

Forecast

Prediction for the reference period (1981 to 2010), as well as forecasts to 2071 to 2100 under szenario RCP4.5 and RCP8.5. Dark green symbolizes a high site index (tree height in m at age 100), orange a lower site index and red no growth. Results were aggregated on 25 km x 25 km scale.

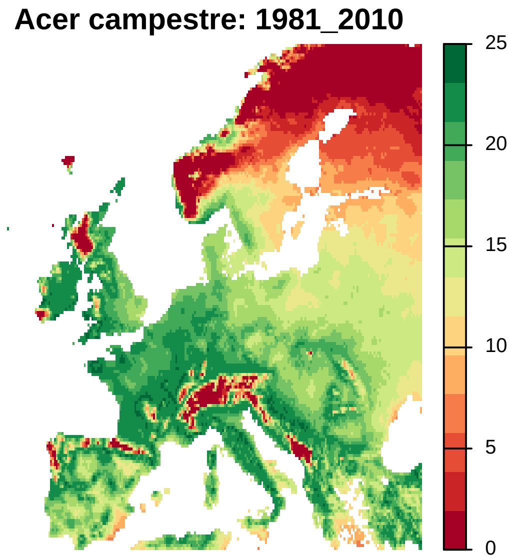

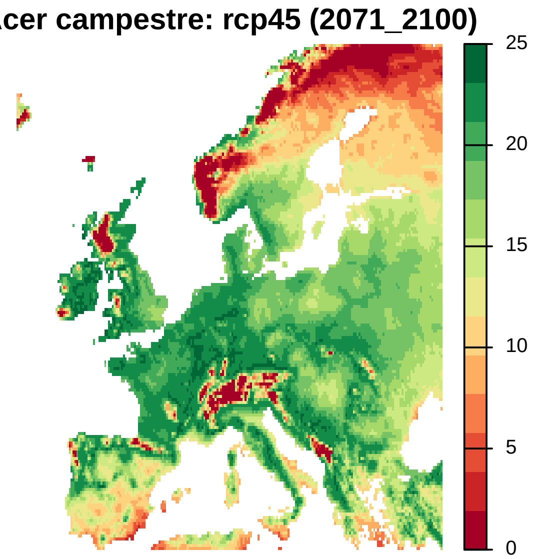

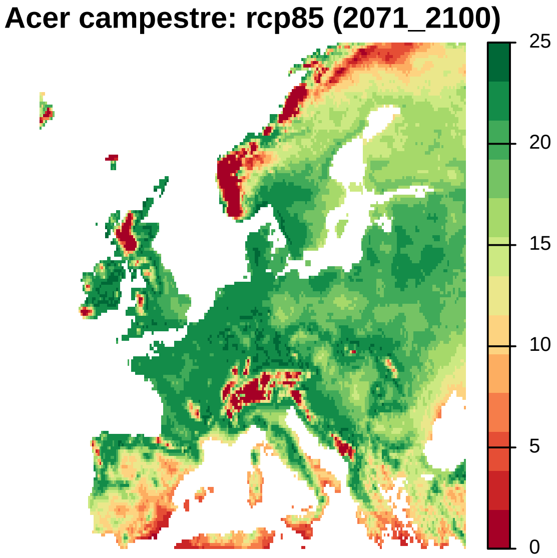

Acer platanoides

Site index curves

Site index curves of Acer platanoides created with non-linear quantile regressions based on the algorithm of Koenker and Park (1992). The site index (SI) was created by setting all points on the 95 percent quantile (upper line) and above to one (SI = 1) and all on the 5 percent quantile (lower line) and below to zero (SI = 0). The points between the quantile boundaries were assigned a site index between zero and one according to the ratio of their position between the quantile boundaries. We set selected absences (see chapter 2.1.3) on Height = 0 m (at age 100), which means, depending on the site index curves, for each tree species a SI near -1 (red line).

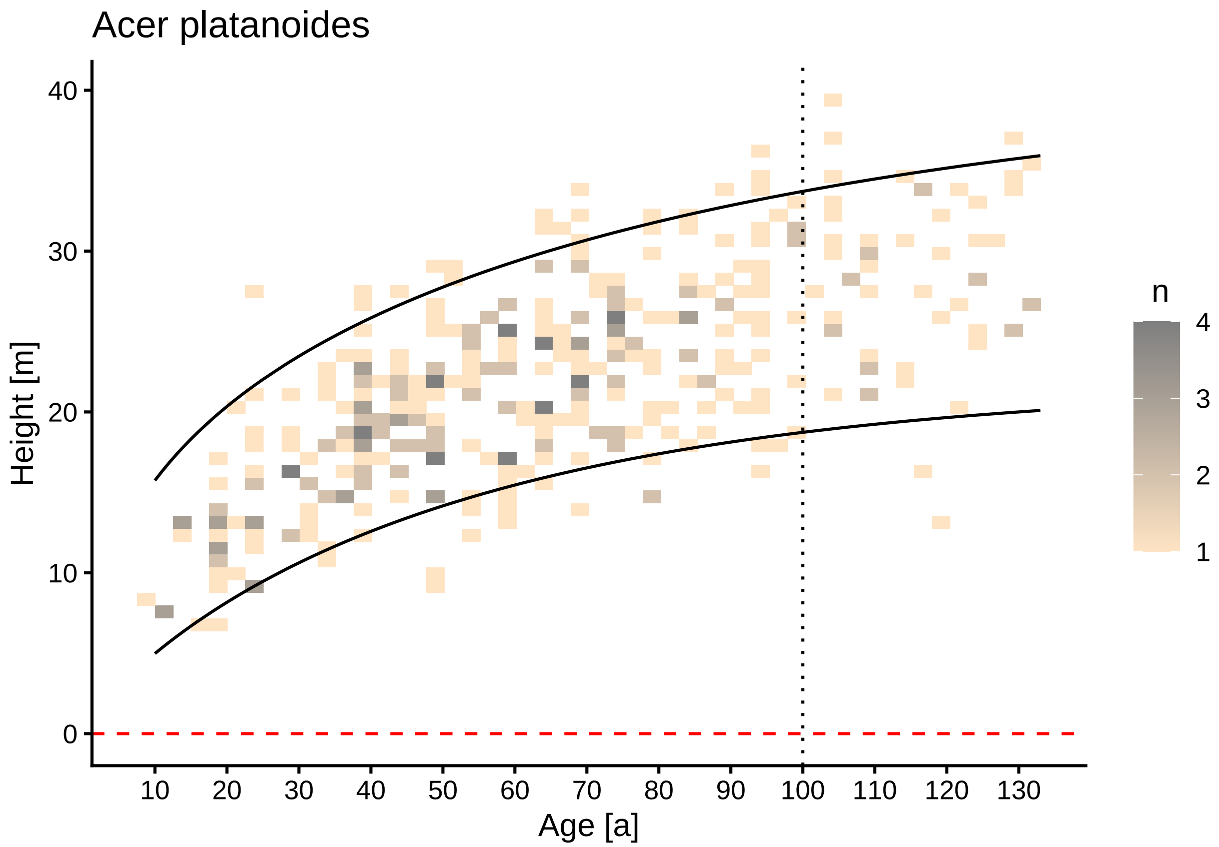

Model statistics and evaluation

Summary

Predictor acronyms: Bio.1 = Mean annual temperature [°C], Bio.12 = Annual precipitation sum [mm/m2], sp_p = Sum of precipitation [mm/m2] within months 3 to 5, su_p = Sum of precipitation [mm/m2] within months 6 to 8, wi_p = Sum of precipitation [mm/m2] within months 12,1,2, sp_t = Mean temperature [°C] within months 3 to 5, su_t = Mean temperature [°C] within months 6 to 8, wi_t = Mean temperature [°C] within months 12,1,2.

```
##
## Family: gaussian
## Link function: identity
##
## Formula:
## H03 ~ s(reference_19812010_su_t, k = 3) + s(reference_19812010_wi_t,
##     k = 3) + s(reference_19812010_su_p, k = 3) + s(reference_19812010_sp_p,
##     k = 3)
##
## Parametric coefficients:
##             Estimate Std. Error t value Pr(>|t|)
## (Intercept)   0.2054     0.0213   9.643   <2e-16 ***
## ---
## Signif. codes:  0 '***' 0.001 '**' 0.01 '*' 0.05 '.' 0.1 ' ' 1
##
## Approximate significance of smooth terms:
##                              edf Ref.df     F p-value
## s(reference_19812010_su_t) 1.965  1.998 23.88  <2e-16 ***
## s(reference_19812010_wi_t) 1.976  1.999 34.28  <2e-16 ***
## s(reference_19812010_su_p) 1.778  1.949 39.93  <2e-16 ***
## s(reference_19812010_sp_p) 1.957  1.997 29.10  <2e-16 ***
## ---
## Signif. codes:  0 '***' 0.001 '**' 0.01 '*' 0.05 '.' 0.1 ' ' 1
##
## R-sq.(adj) =  0.661   Deviance explained = 66.8%
## -REML = 214.49  Scale est. = 0.16874   n = 372
```

Variance inflation factor (VIF)

Predictor acronyms: Bio.1 = Mean annual temperature [°C], Bio.12 = Annual precipitation sum [mm/m2], sp_p = Sum of precipitation [mm/m2] within months 3 to 5, su_p = Sum of precipitation [mm/m2] within months 6 to 8, wi_p = Sum of precipitation [mm/m2] within months 12,1,2, sp_t = Mean temperature [°C] within months 3 to 5, su_t = Mean temperature [°C] within months 6 to 8, wi_t = Mean temperature [°C] within months 12,1,2.

```
##                 Variables      VIF
## 1 reference_19812010_su_t 2.099878
## 2 reference_19812010_wi_t 2.567395
## 3 reference_19812010_su_p 3.229286
## 4 reference_19812010_sp_p 2.441017
```

Correlation matrix

Correlation matrix between the predictor variables and the target variable in the model. Correlation coefficient according to PEARSON. Predictor acronyms: Bio.1 = Mean annual temperature [°C], Bio.12 = Annual precipitation sum [mm/m2], sp_p = Sum of precipitation [mm/m2] within months 3 to 5, su_p = Sum of precipitation [mm/m2] within months 6 to 8, wi_p = Sum of precipitation [mm/m2] within months 12,1,2, sp_t = Mean temperature [°C] within months 3 to 5, su_t = Mean temperature [°C] within months 6 to 8, wi_t = Mean temperature [°C] within months 12,1,2.

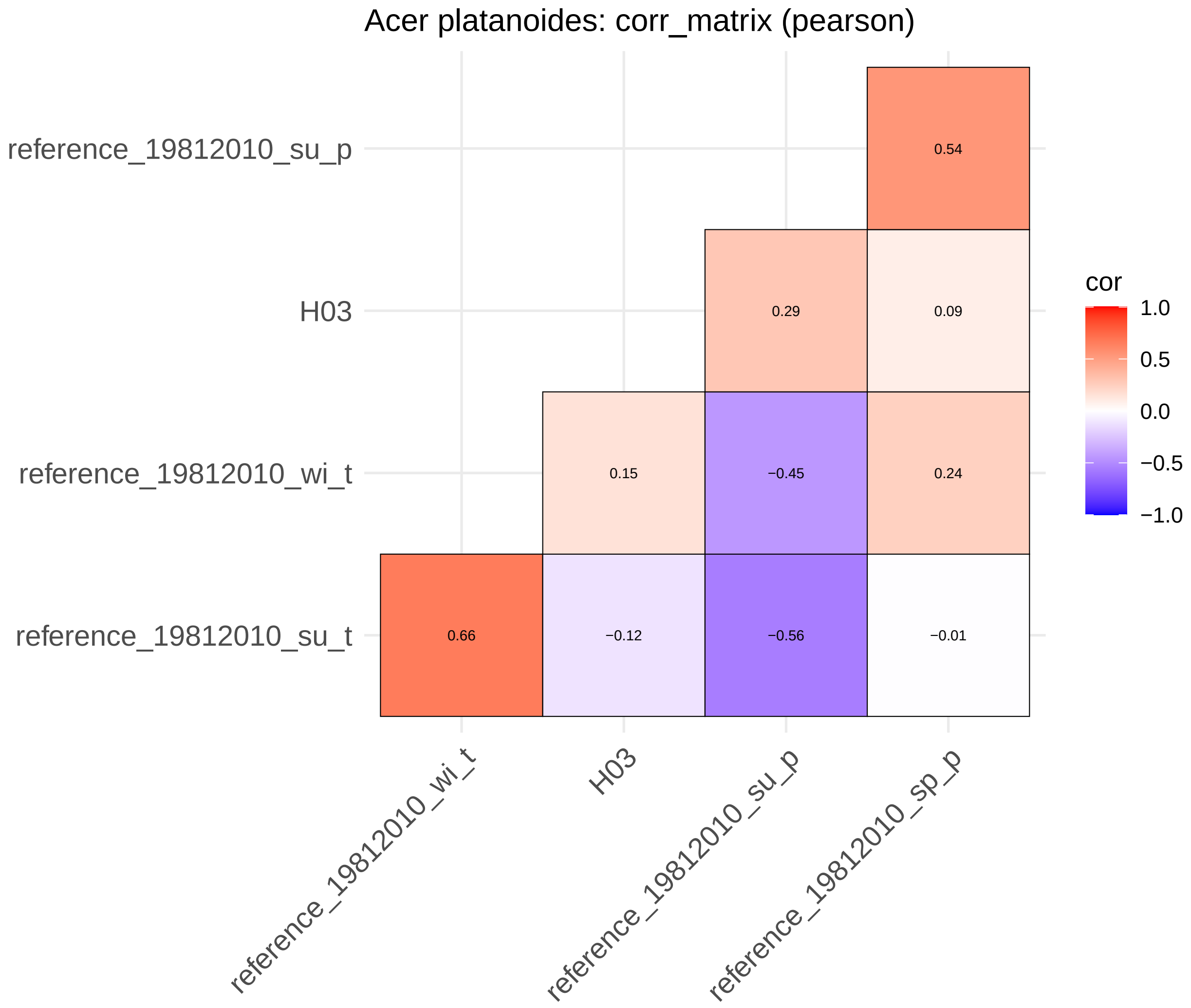

Response curves

Response curves (also known as effect curves) show how each predictor variable affects the target variable (H03 = european Site index, SIrel). H03 values below zero represent ‘Growth absences’. Predictor acronyms: Bio.1 = Mean annual temperature [°C], Bio.12 = Annual precipitation sum [mm/m2], sp_p = Sum of precipitation [mm/m2] within months 3 to 5, su_p = Sum of precipitation [mm/m2] within months 6 to 8, wi_p = Sum of precipitation [mm/m2] within months 12,1,2, sp_t = Mean temperature [°C] within months 3 to 5, su_t = Mean temperature [°C] within months 6 to 8, wi_t = Mean temperature [°C] within months 12,1,2.

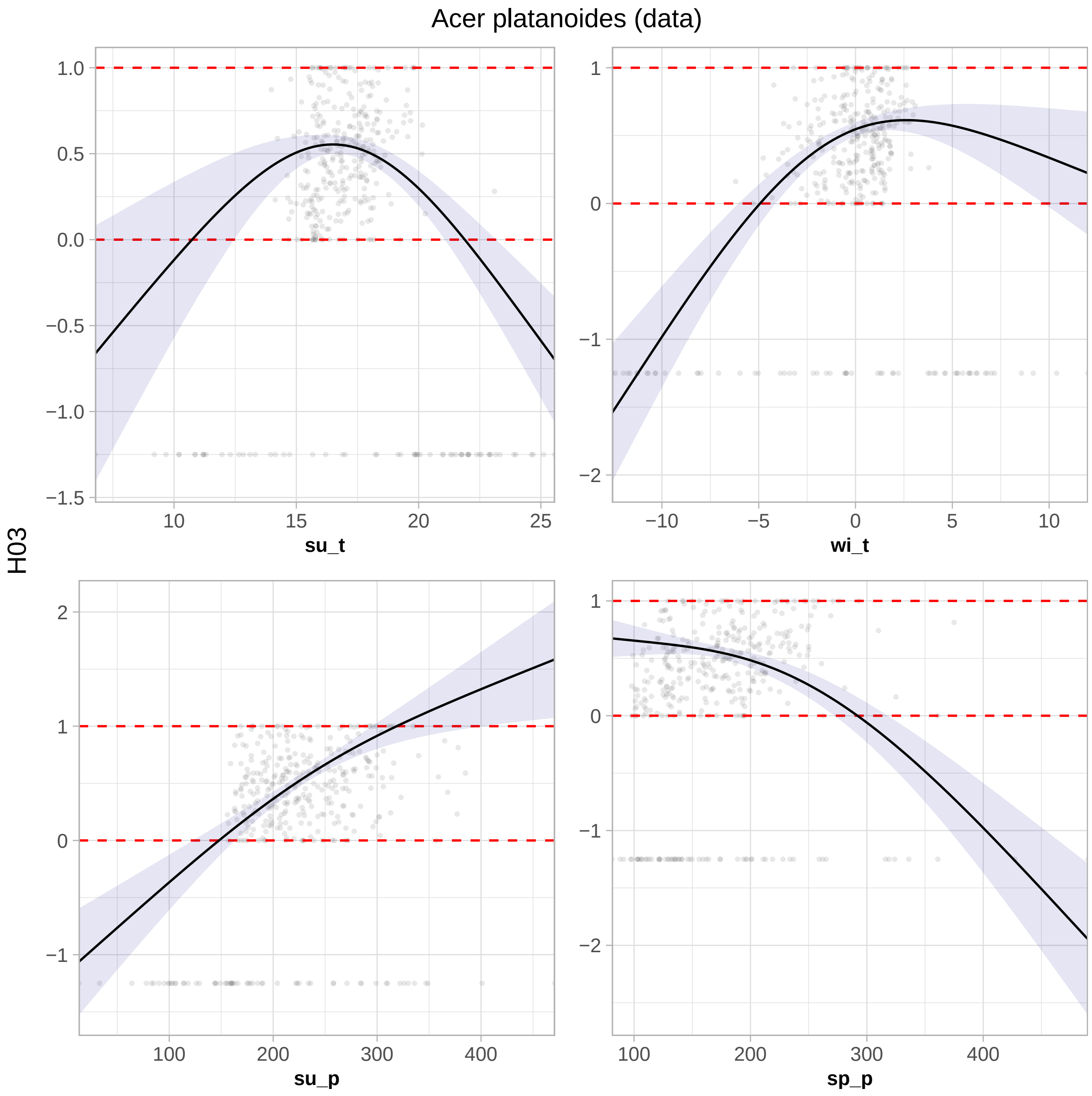

Response maps

Response maps (also referred as partial effect maps). Each map visualizes how a predictor affect the target variable (top height [m] at Age 100). Technically their work like response curves in a geographical area, that is setting all predictor variables except the one shown in the figure on their mean, and mapping the prediction. Predictor acronyms: Bio.1 = Mean annual temperature [°C], Bio.12 = Annual precipitation sum [mm/m2], sp_p = Sum of precipitation [mm/m2] within months 3 to 5, su_p = Sum of precipitation [mm/m2] within months 6 to 8, wi_p = Sum of precipitation [mm/m2] within months 12,1,2, sp_t = Mean temperature [°C] within months 3 to 5, su_t = Mean temperature [°C] within months 6 to 8, wi_t = Mean temperature [°C] within months 12,1,2.

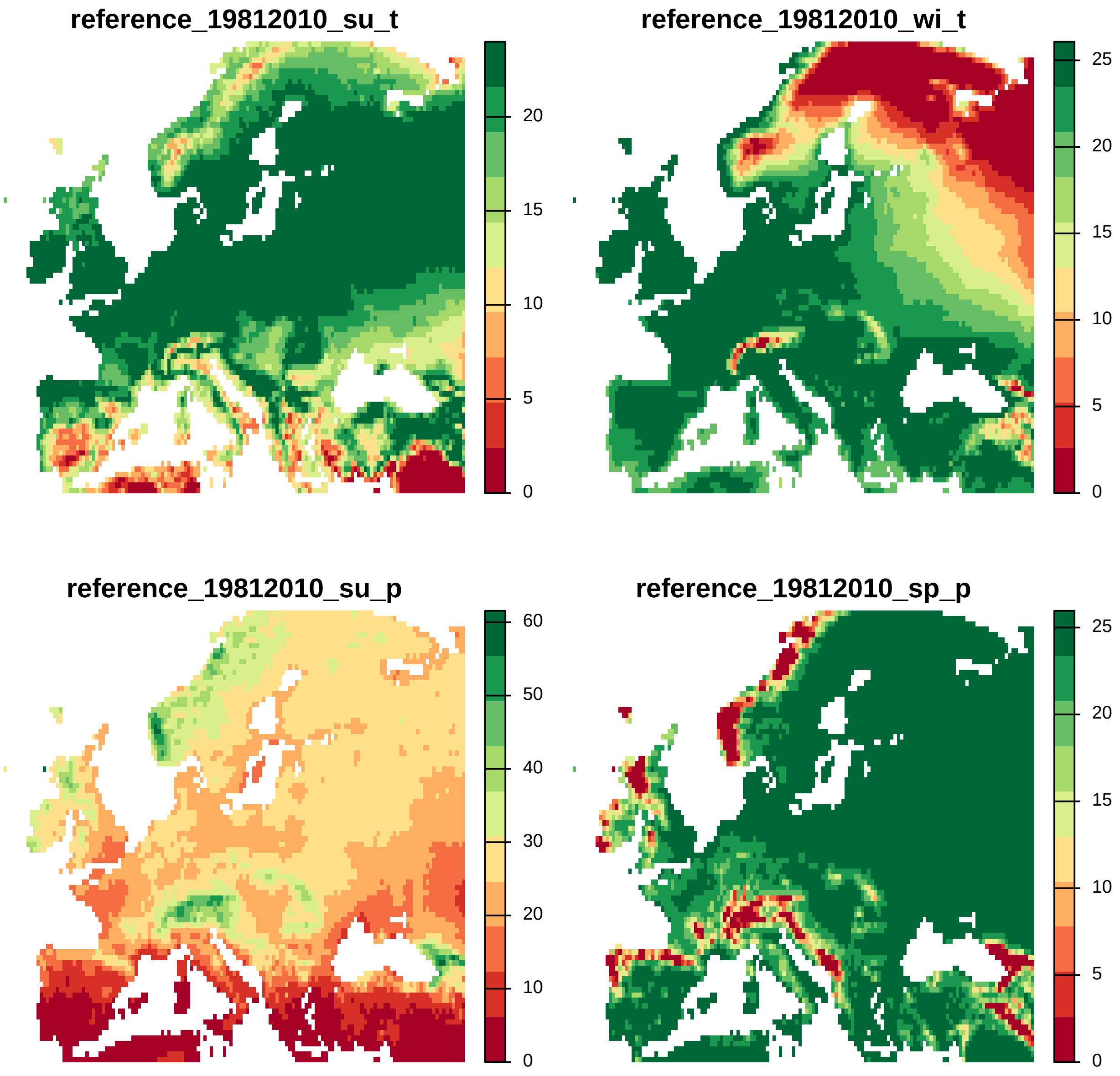

Residual distribution

The multi-panel plot includes a histogram of the residuals (top left), residuals over fitted values (top right), a histogram of observed and predicted values (bottom left) and boxplot diagram of observed and predicted values (bottom right). Observed values are shown in light green, while predicted ones are depicted in light red.

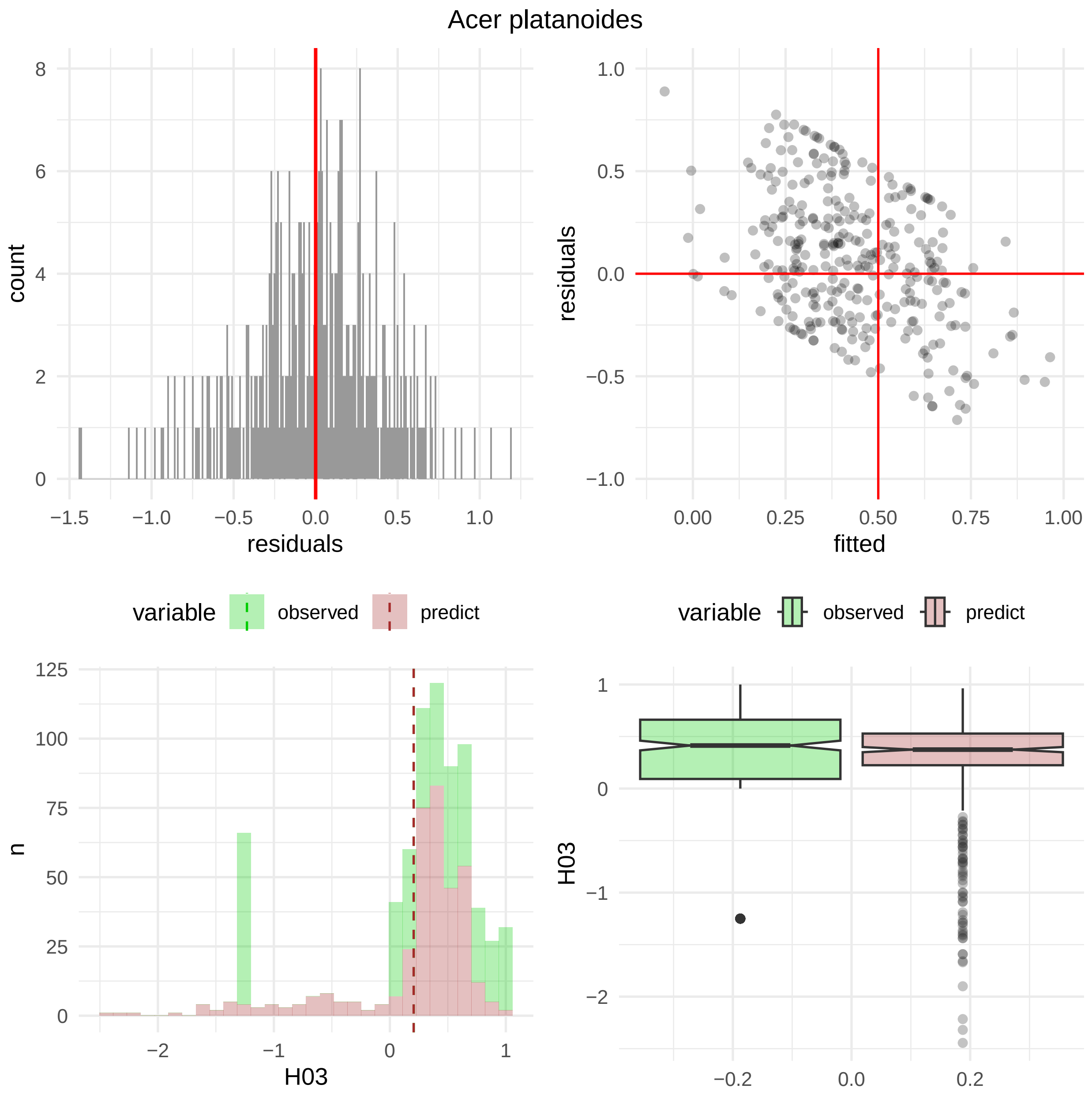

Correlation between predict and observed site index

Relationship between predicted and observed site index (density cloud), as well as linear regressions of presences and absences (= ‘growth absences’) (red line) and presences only (magenta line). The formulas, significance, R2 and number of observations are displayed below for both regressions. Ideally, both the point cloud and the regression lines lie close to the dashed line. For presences only we additionally calculated the correlation coefficient according to PEARSON (cor.pre) in the bottom right corner.

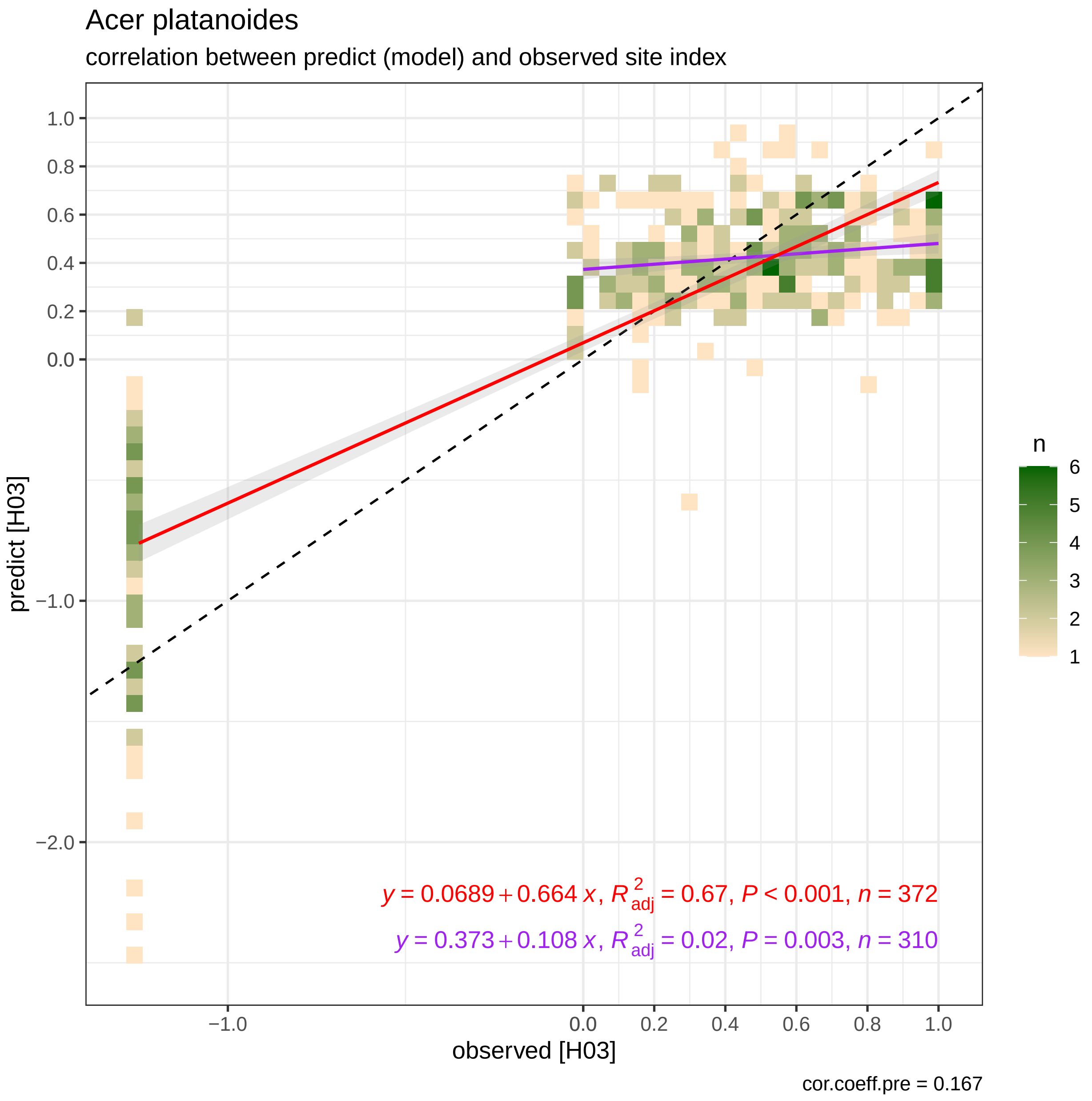

Predictions and forecasts

Predict

European predict for the reference period (1981 to 2010). Dark green symbolizes a high site index (tree height in meters at age 100), orange a lower site index and red no growth. Magenta-coloured dots represent inventory points with growth information, light blue dots are absences (= ‘growth absences’). Results were aggregated on 25 km x 25 km scale.

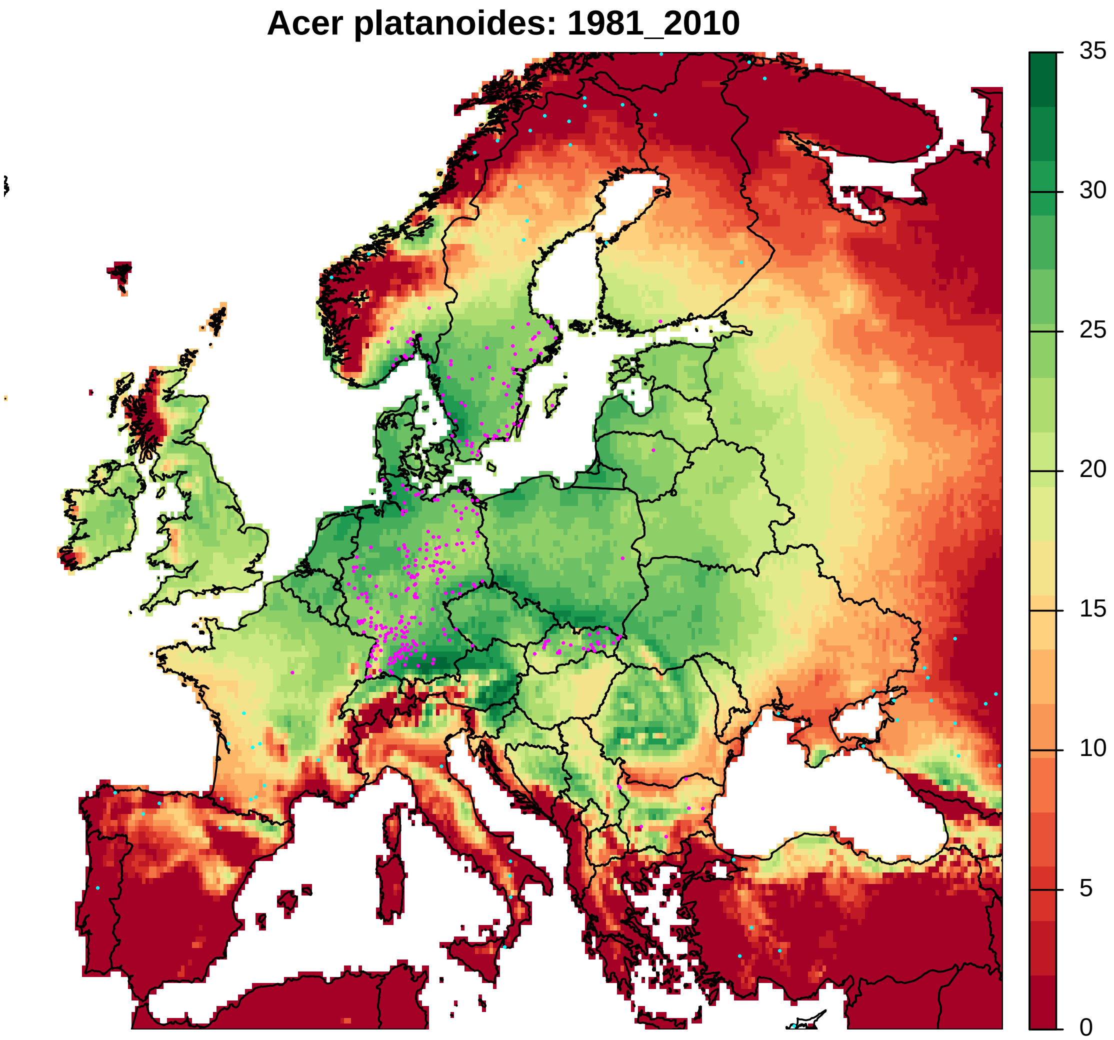

Forecast

Prediction for the reference period (1981 to 2010), as well as forecasts to 2071 to 2100 under szenario RCP4.5 and RCP8.5. Dark green symbolizes a high site index (tree height in m at age 100), orange a lower site index and red no growth. Results were aggregated on 25 km x 25 km scale.

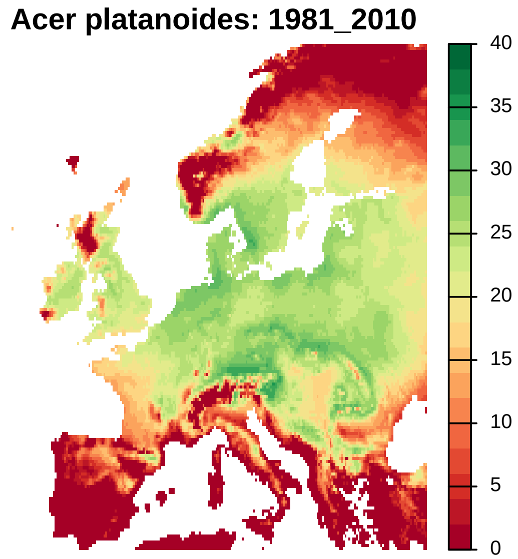

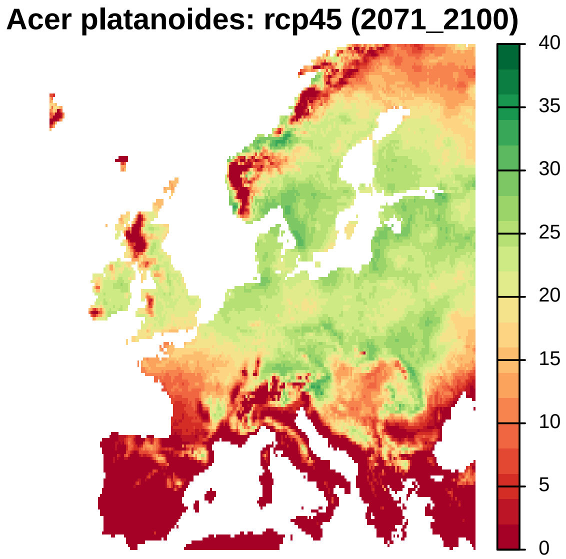

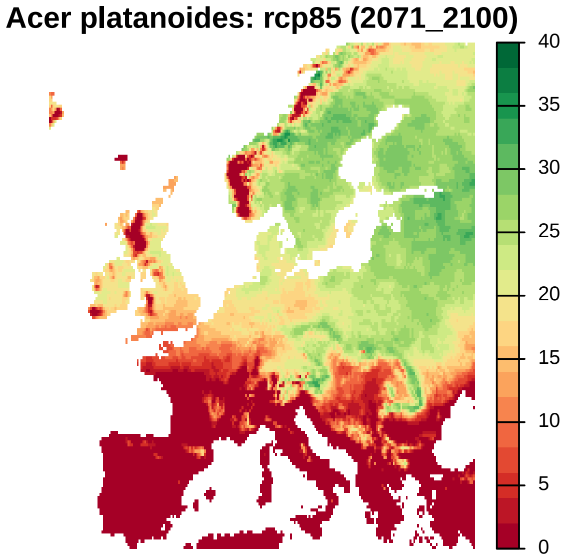

Acer pseudoplatanus

Site index curves

Site index curves of Acer pseudoplatanus created with non-linear quantile regressions based on the algorithm of Koenker and Park (1992). The site index (SI) was created by setting all points on the 95 percent quantile (upper line) and above to one (SI = 1) and all on the 5 percent quantile (lower line) and below to zero (SI = 0). The points between the quantile boundaries were assigned a site index between zero and one according to the ratio of their position between the quantile boundaries. We set selected absences (see chapter 2.1.3) on Height = 0 m (at age 100), which means, depending on the site index curves, for each tree species a SI near -1 (red line).

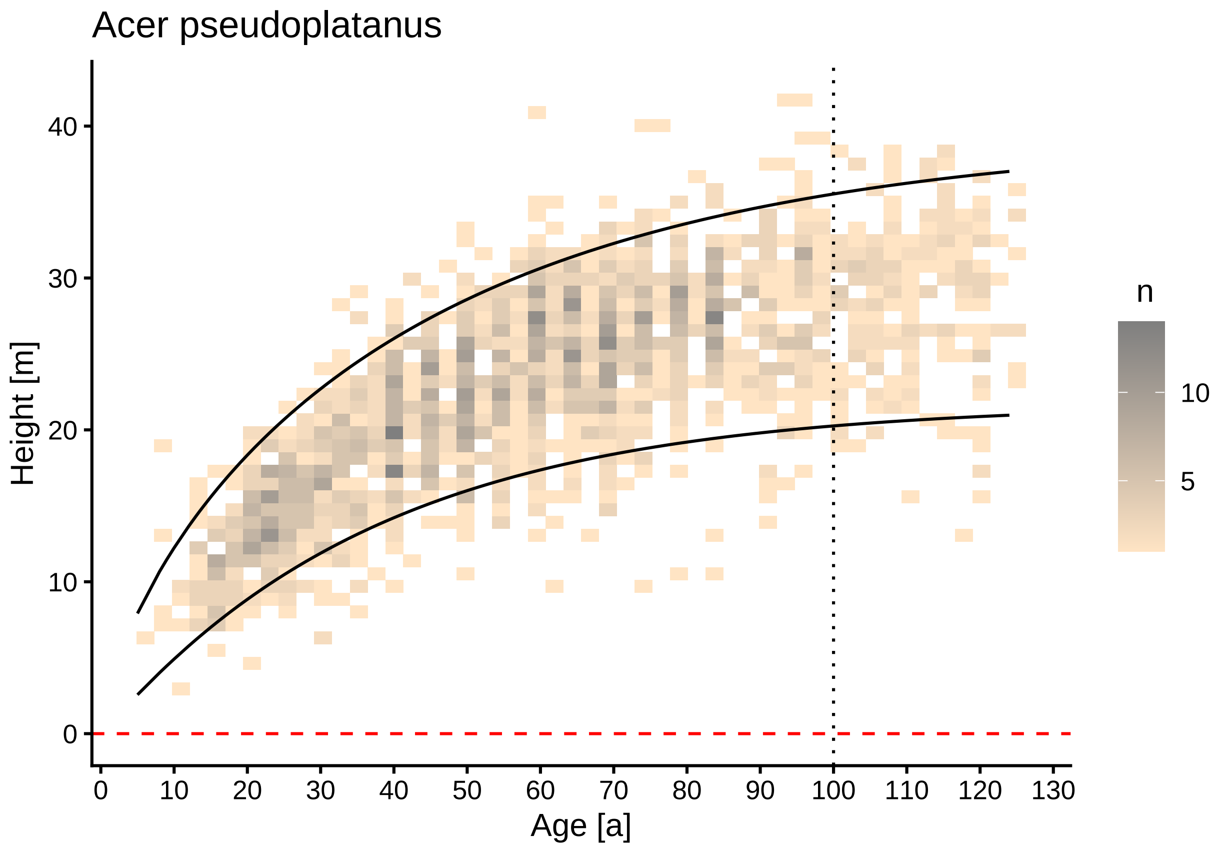

Model statistics and evaluation

Summary

Predictor acronyms: Bio.1 = Mean annual temperature [°C], Bio.12 = Annual precipitation sum [mm/m2], sp_p = Sum of precipitation [mm/m2] within months 3 to 5, su_p = Sum of precipitation [mm/m2] within months 6 to 8, wi_p = Sum of precipitation [mm/m2] within months 12,1,2, sp_t = Mean temperature [°C] within months 3 to 5, su_t = Mean temperature [°C] within months 6 to 8, wi_t = Mean temperature [°C] within months 12,1,2.

```
##
## Family: gaussian
## Link function: identity
##
## Formula:
## H03 ~ s(reference_19812010_wi_t, k = 3) + s(reference_19812010_su_t,
##     k = 3) + s(reference_19812010_su_p, k = 3)
##
## Parametric coefficients:
##             Estimate Std. Error t value Pr(>|t|)
## (Intercept)  0.29053    0.01508   19.27   <2e-16 ***
## ---
## Signif. codes:  0 '***' 0.001 '**' 0.01 '*' 0.05 '.' 0.1 ' ' 1
##
## Approximate significance of smooth terms:
##                              edf Ref.df      F p-value
## s(reference_19812010_wi_t) 1.995  2.000 239.73  <2e-16 ***
## s(reference_19812010_su_t) 1.966  1.999  52.86  <2e-16 ***
## s(reference_19812010_su_p) 1.965  1.999  45.66  <2e-16 ***
## ---
## Signif. codes:  0 '***' 0.001 '**' 0.01 '*' 0.05 '.' 0.1 ' ' 1
##
## R-sq.(adj) =  0.616   Deviance explained = 61.9%
## -REML = 693.04  Scale est. = 0.22688   n = 998
```

Variance inflation factor (VIF)

Predictor acronyms: Bio.1 = Mean annual temperature [°C], Bio.12 = Annual precipitation sum [mm/m2], sp_p = Sum of precipitation [mm/m2] within months 3 to 5, su_p = Sum of precipitation [mm/m2] within months 6 to 8, wi_p = Sum of precipitation [mm/m2] within months 12,1,2, sp_t = Mean temperature [°C] within months 3 to 5, su_t = Mean temperature [°C] within months 6 to 8, wi_t = Mean temperature [°C] within months 12,1,2.

```
##                  Variables      VIF
## 1 reference_19812010_wi_t 1.521449
## 2 reference_19812010_su_t 1.781658
## 3 reference_19812010_su_p 1.356859
```

Correlation matrix

Correlation matrix between the predictor variables and the target variable in the model. Correlation coefficient according to PEARSON. Predictor acronyms: Bio.1 = Mean annual temperature [°C], Bio.12 = Annual precipitation sum [mm/m2], sp_p = Sum of precipitation [mm/m2] within months 3 to 5, su_p = Sum of precipitation [mm/m2] within months 6 to 8, wi_p = Sum of precipitation [mm/m2] within months 12,1,2, sp_t = Mean temperature [°C] within months 3 to 5, su_t = Mean temperature [°C] within months 6 to 8, wi_t = Mean temperature [°C] within months 12,1,2.

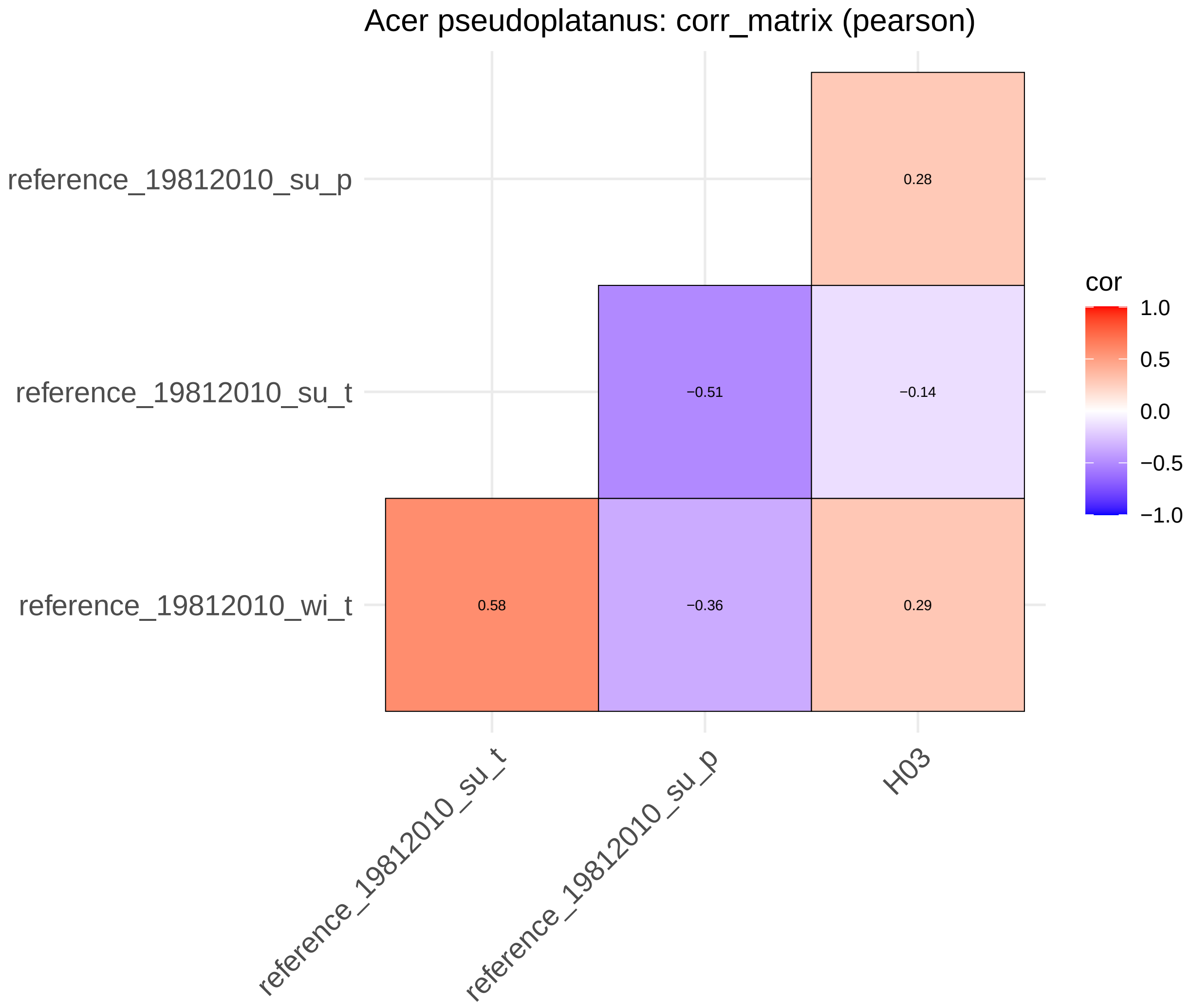

Response curves

Response curves (also known as effect curves) show how each predictor variable affects the target variable (H03 = european Site index, SIrel). H03 values below zero represent 'Growth absences'. Predictor acronyms: Bio.1 = Mean annual temperature [°C], Bio.12 = Annual precipitation sum [mm/m2], sp_p = Sum of precipitation [mm/m2] within months 3 to 5, su_p = Sum of precipitation [mm/m2] within months 6 to 8, wi_p = Sum of precipitation [mm/m2] within months 12,1,2, sp_t = Mean temperature [°C] within months 3 to 5, su_t = Mean temperature [°C] within months 6 to 8, wi_t = Mean temperature [°C] within months 12,1,2.

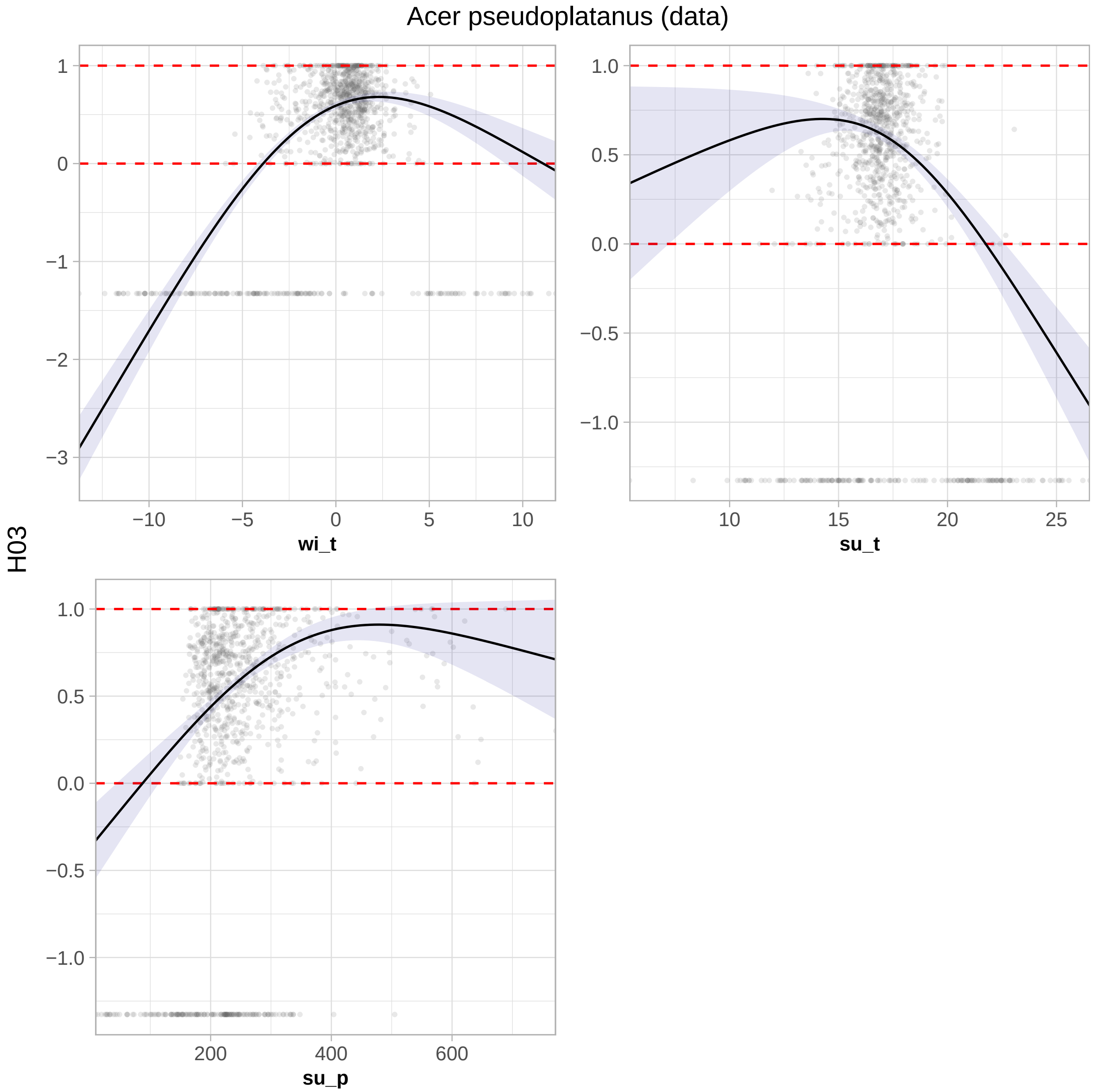

Response maps

Response maps (also referred as partial effect maps). Each map visualizes how a predictor affect the target variable (top height [m] at Age 100). Technically their work like response curves in a geographical area, that is setting all predictor variables except the one shown in the figure on their mean, and mapping the prediction. Predictor acronyms: Bio.1 = Mean annual temperature [°C], Bio.12 = Annual precipitation sum [mm/m2], sp_p = Sum of precipitation [mm/m2] within months 3 to 5, su_p = Sum of precipitation [mm/m2] within months 6 to 8, wi_p = Sum of precipitation [mm/m2] within months 12,1,2, sp_t = Mean temperature [°C] within months 3 to 5, su_t = Mean temperature [°C] within months 6 to 8, wi_t = Mean temperature [°C] within months 12,1,2.

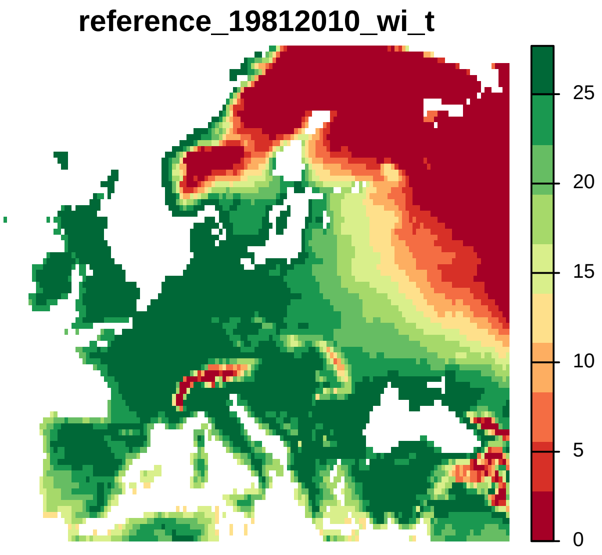

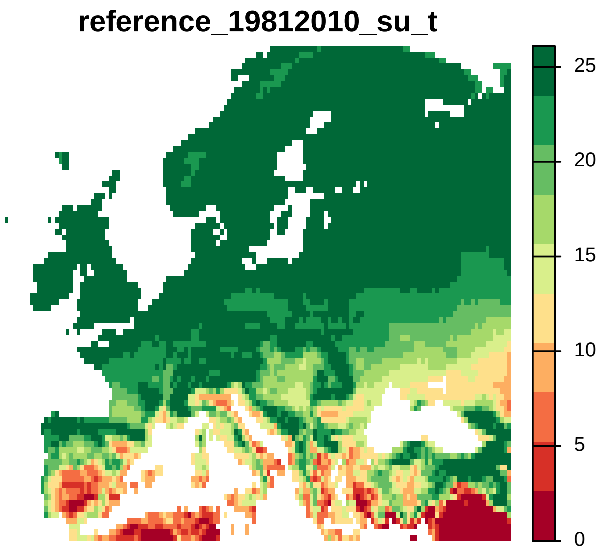

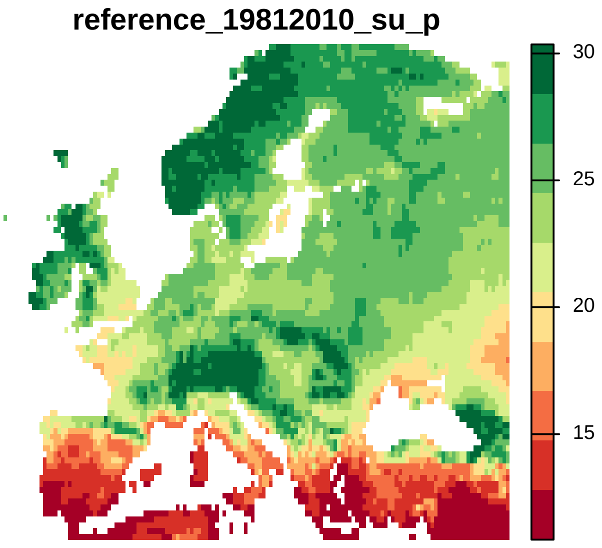

Residual distribution

The multi-panel plot includes a histogram of the residuals (top left), residuals over fitted values (top right), a histogram of observed and predicted values (bottom left) and boxplot diagram of observed and predicted values (bottom right). Observed values are shown in light green, while predicted ones are depicted in light red.

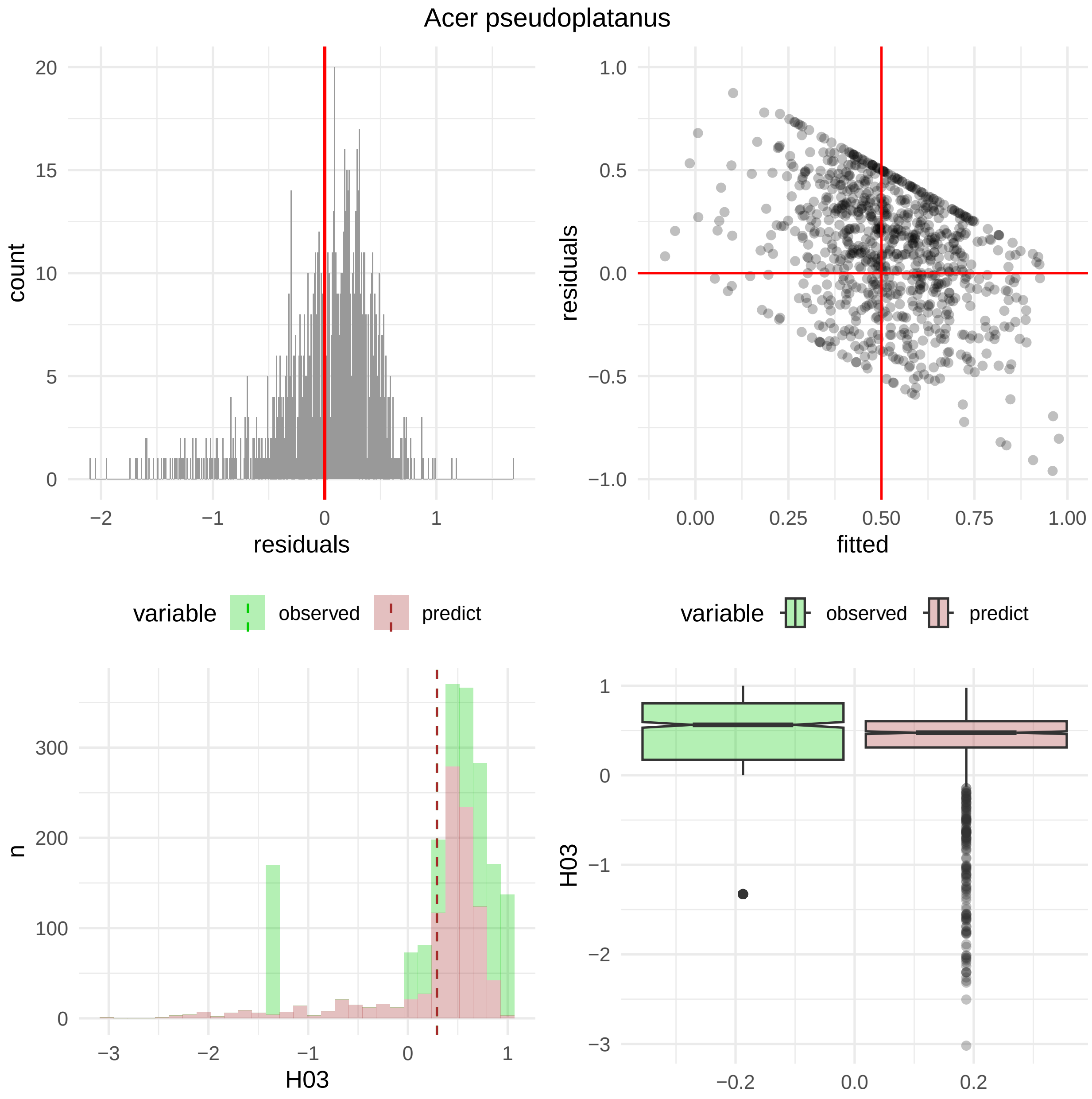

Correlation between predict and observed site index

Relationship between predicted and observed site index (density cloud), as well as linear regressions of presences and absences (= 'growth absences') (red line) and presences only (magenta line). The formulas, significance, R2 and number of observations are displayed below for both regressions. Ideally, both the point cloud and the regression lines lie close to the dashed line. For presences only we additionally calculated the correlation coefficient according to PEARSON (cor.pre) in the bottom right corner.

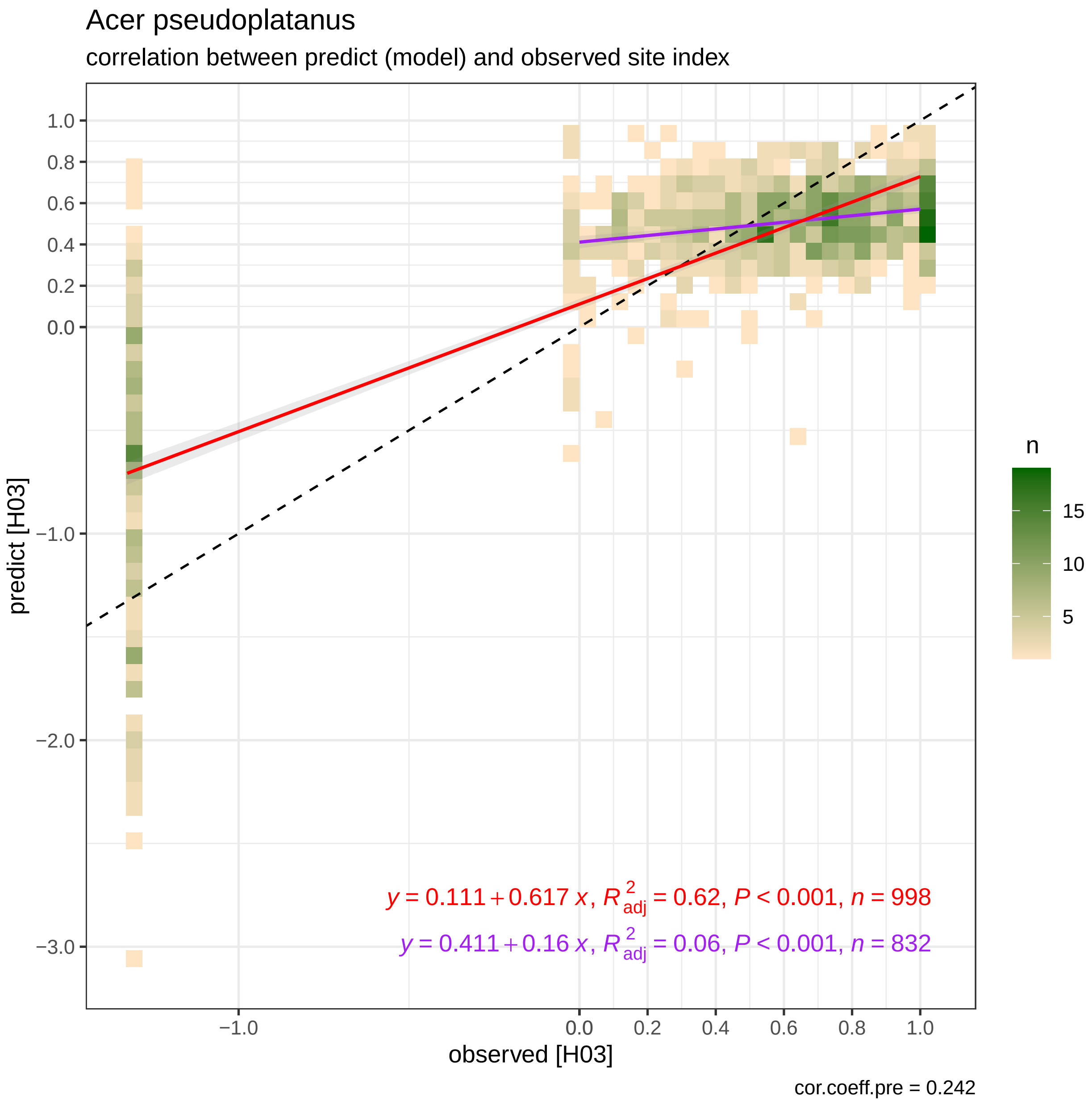

Predictions and forecasts

Predict

European predict for the reference period (1981 to 2010). Dark green symbolizes a high site index (tree height in meters at age 100), orange a lower site index and red no growth. Magenta-coloured dots represent inventory points with growth information, light blue dots are absences (= 'growth absences'). Results were aggregated on 25 km x 25 km scale.

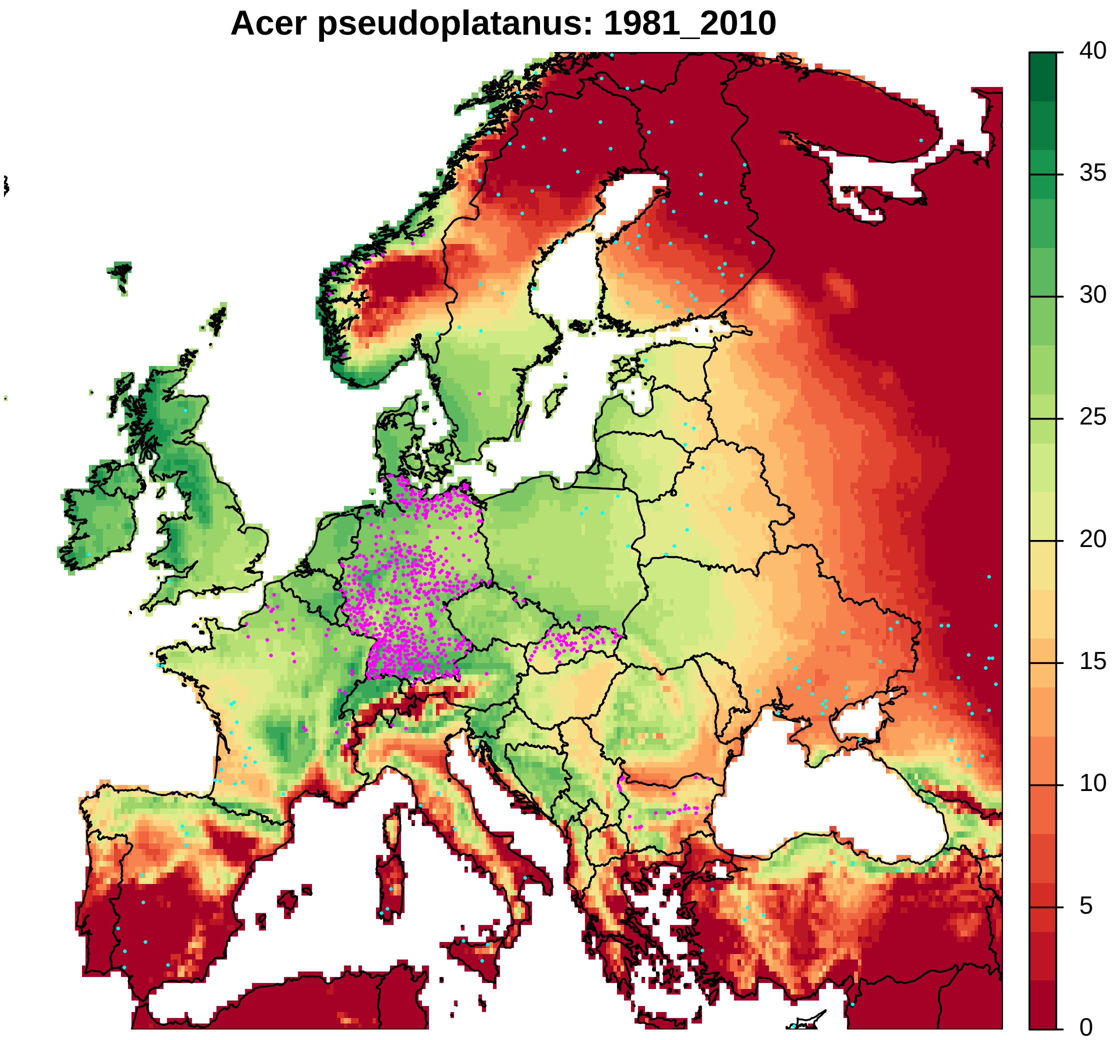

Forecast

Prediction for the reference period (1981 to 2010), as well as forecasts to 2071 to 2100 under szenario RCP4.5 and RCP8.5. Dark green symbolizes a high site index (tree height in m at age 100), orange a lower site index and red no growth. Results were aggregated on 25 km x 25 km scale.

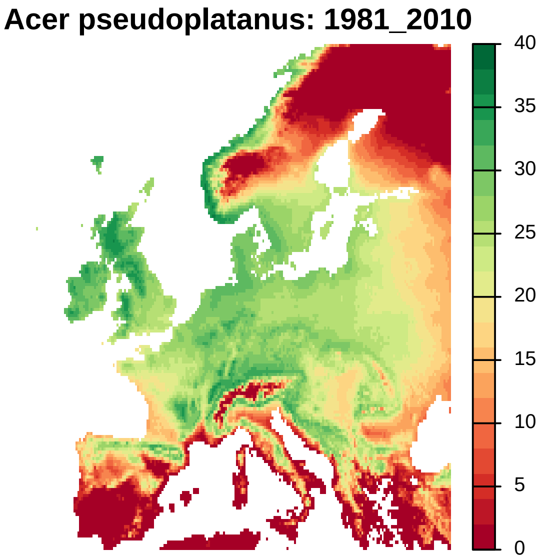

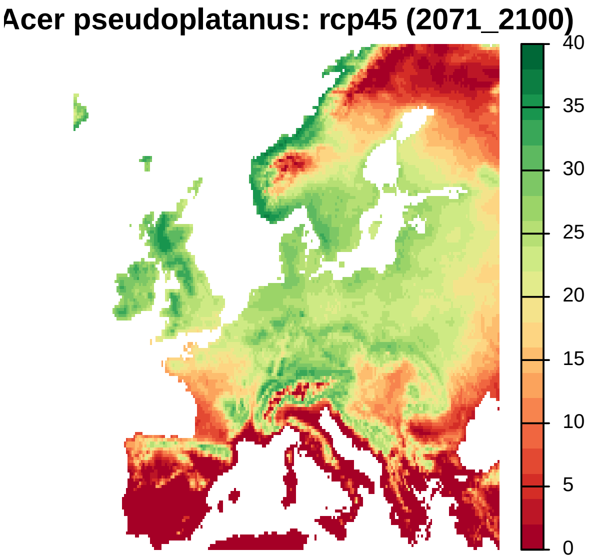

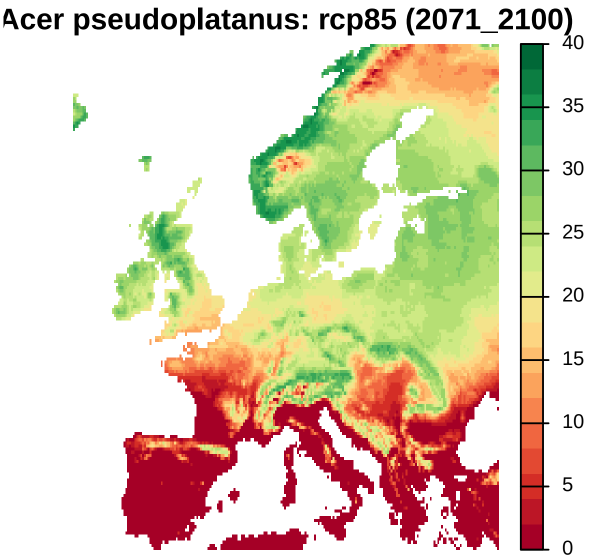

Alnus glutinosa

Site index curves

Site index curves of Alnus glutinosa created with non-linear quantile regressions based on the algorithm of Koenker and Park (1992). The site index (SI) was created by setting all points on the 95 percent quantile (upper line) and above to one (SI = 1) and all on the 5 percent quantile (lower line) and below to zero (SI = 0). The points between the quantile boundaries were assigned a site index between zero and one according to the ratio of their position between the quantile boundaries. We set selected absences (see chapter 2.1.3) on Height = 0 m (at age 100), which means, depending on the site index curves, for each tree species a SI near -1 (red line).

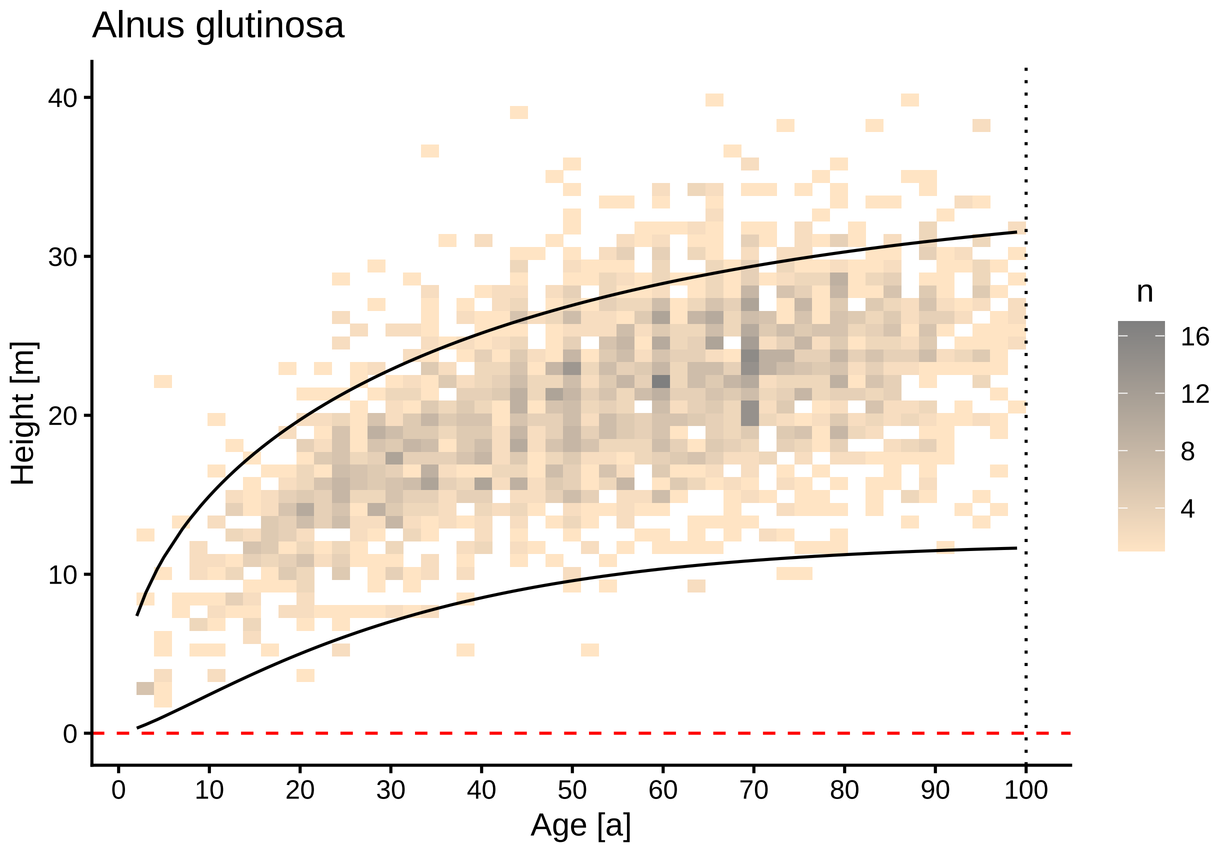

Model statistics and evaluation

Summary

Predictor acronyms: Bio.1 = Mean annual temperature [°C], Bio.12 = Annual precipitation sum [mm/m2], sp_p = Sum of precipitation [mm/m2] within months 3 to 5, su_p = Sum of precipitation [mm/m2] within months 6 to 8, wi_p = Sum of precipitation [mm/m2] within months 12,1,2, sp_t = Mean temperature [°C] within months 3 to 5, su_t = Mean temperature [°C] within months 6 to 8, wi_t = Mean temperature [°C] within months 12,1,2.

```
##
## Family: gaussian
## Link function: identity
##
## Formula:
## H03 ~ s(reference_19812010_su_t, k = 3) + s(reference_19812010_wi_t,
##     k = 3) + s(reference_19812010_su_p, k = 3)
##
## Parametric coefficients:
##             Estimate Std. Error t value Pr(>|t|)
## (Intercept) 0.472939   0.007353   64.32   <2e-16 ***
## ---
## Signif. codes:  0 '***' 0.001 '**' 0.01 '*' 0.05 '.' 0.1 ' ' 1
##
## Approximate significance of smooth terms:
##                              edf Ref.df     F p-value
## s(reference_19812010_su_t) 1.998  2.000 402.8  <2e-16 ***
## s(reference_19812010_wi_t) 1.943  1.997 120.1  <2e-16 ***
## s(reference_19812010_su_p) 1.993  2.000 109.1  <2e-16 ***
## ---
## Signif. codes:  0 '***' 0.001 '**' 0.01 '*' 0.05 '.' 0.1 ' ' 1
##
## R-sq.(adj) =  0.685   Deviance explained = 68.6%
## -REML = 318.87  Scale est. = 0.085377  n = 1579
```

Variance inflation factor (VIF)

Predictor acronyms: Bio.1 = Mean annual temperature [°C], Bio.12 = Annual precipitation sum [mm/m2], sp_p = Sum of precipitation [mm/m2] within months 3 to 5, su_p = Sum of precipitation [mm/m2] within months 6 to 8, wi_p = Sum of precipitation [mm/m2] within months 12,1,2, sp_t = Mean temperature [°C] within months 3 to 5, su_t = Mean temperature [°C] within months 6 to 8, wi_t = Mean temperature [°C] within months 12,1,2.

```
##                  Variables      VIF
## 1 reference_19812010_su_t 1.706754
## 2 reference_19812010_wi_t 1.442740
## 3 reference_19812010_su_p 1.295518
```

Correlation matrix

Correlation matrix between the predictor variables and the target variable in the model. Correlation coefficient according to PEARSON. Predictor acronyms: Bio.1 = Mean annual temperature [°C], Bio.12 = Annual precipitation sum [mm/m2], sp_p = Sum of precipitation [mm/m2] within months 3 to 5, su_p = Sum of precipitation [mm/m2] within months 6 to 8, wi_p = Sum of precipitation [mm/m2] within months 12,1,2, sp_t = Mean temperature [°C] within months 3 to 5, su_t = Mean temperature [°C] within months 6 to 8, wi_t = Mean temperature [°C] within months 12,1,2.

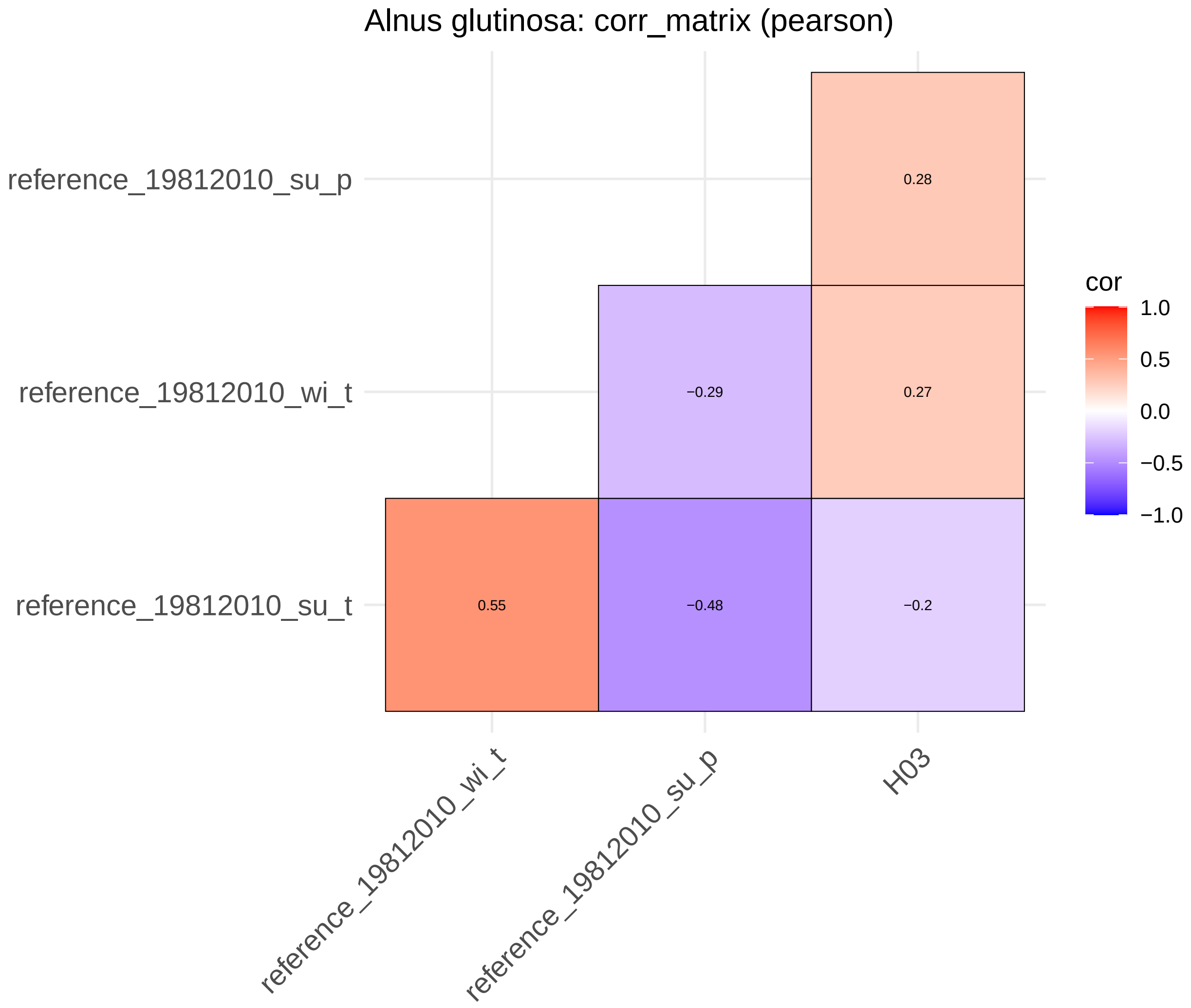

Response curves

Response curves (also known as effect curves) show how each predictor variable affects the target variable (H03 = european Site index, SIrel). H03 values below zero represent 'Growth absences'. Predictor acronyms: Bio.1 = Mean annual temperature [°C], Bio.12 = Annual precipitation sum [mm/m2], sp_p = Sum of precipitation [mm/m2] within months 3 to 5, su_p = Sum of precipitation [mm/m2] within months 6 to 8, wi_p = Sum of precipitation [mm/m2] within months 12,1,2, sp_t = Mean temperature [°C] within months 3 to 5, su_t = Mean temperature [°C] within months 6 to 8, wi_t = Mean temperature [°C] within months 12,1,2.

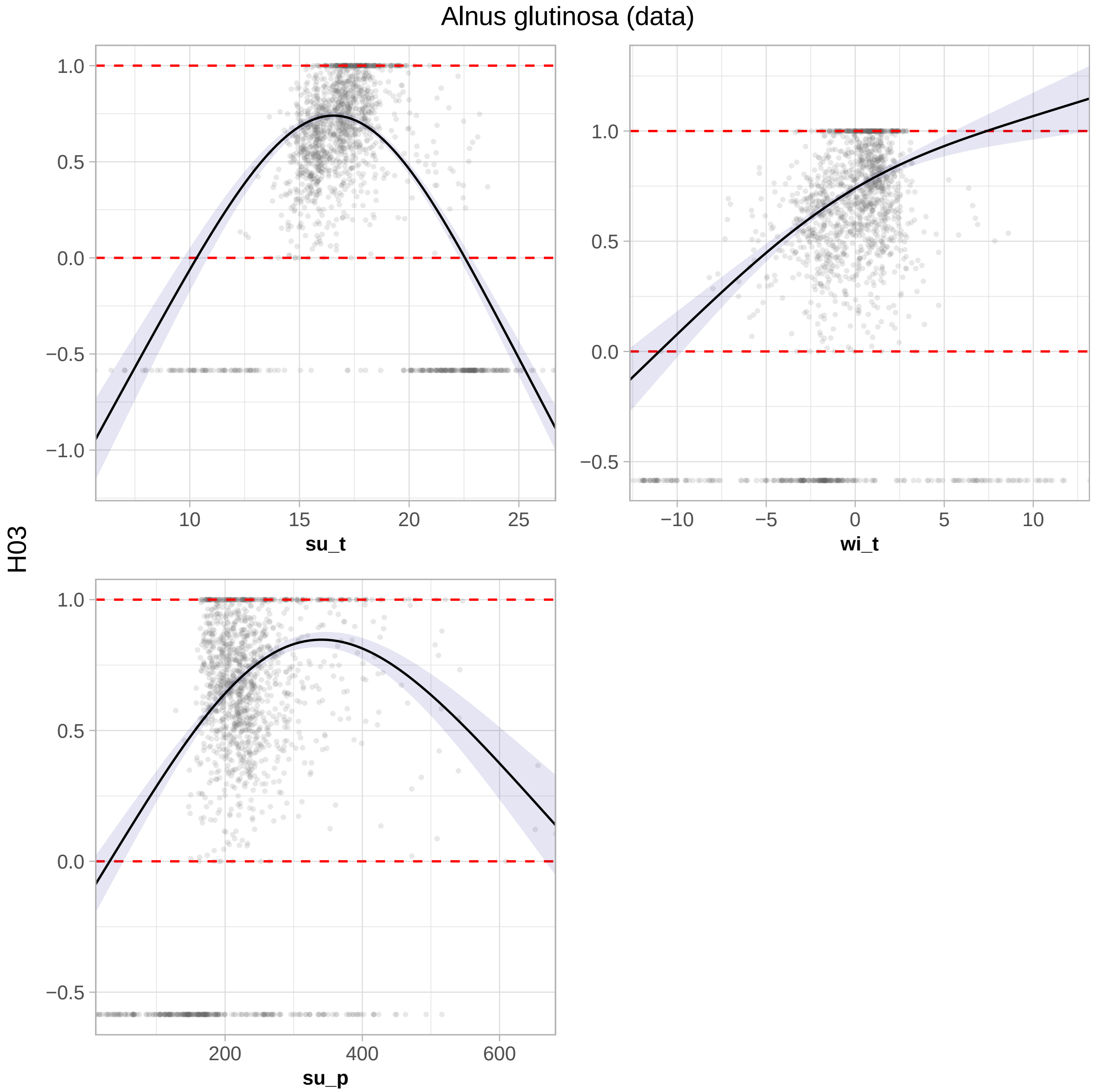

Response maps

Response maps (also referred as partial effect maps). Each map visualizes how a predictor affect the target variable (top height [m] at Age 100). Technically their work like response curves in a geographical area, that is setting all predictor variables except the one shown in the figure on their mean, and mapping the prediction. Predictor acronyms: Bio.1 = Mean annual temperature [°C], Bio.12 = Annual precipitation sum [mm/m2], sp_p = Sum of precipitation [mm/m2] within months 3 to 5, su_p = Sum of precipitation [mm/m2] within months 6 to 8, wi_p = Sum of precipitation [mm/m2] within months 12,1,2, sp_t = Mean temperature [°C] within months 3 to 5, su_t = Mean temperature [°C] within months 6 to 8, wi_t = Mean temperature [°C] within months 12,1,2.

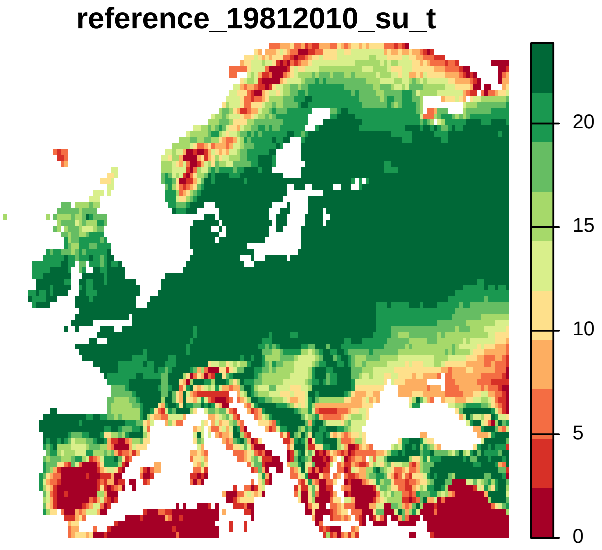

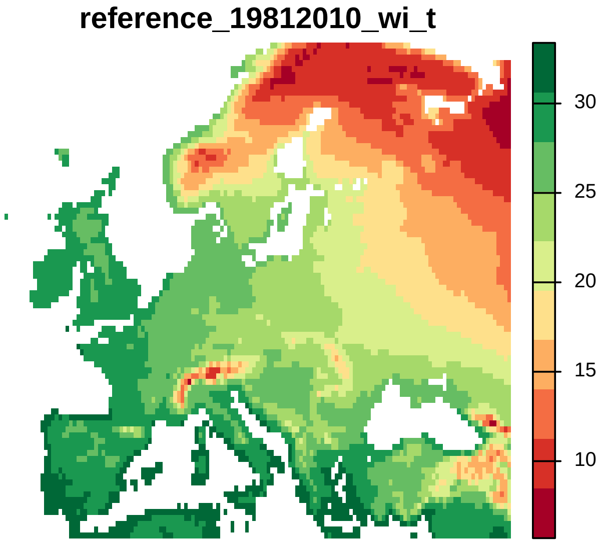

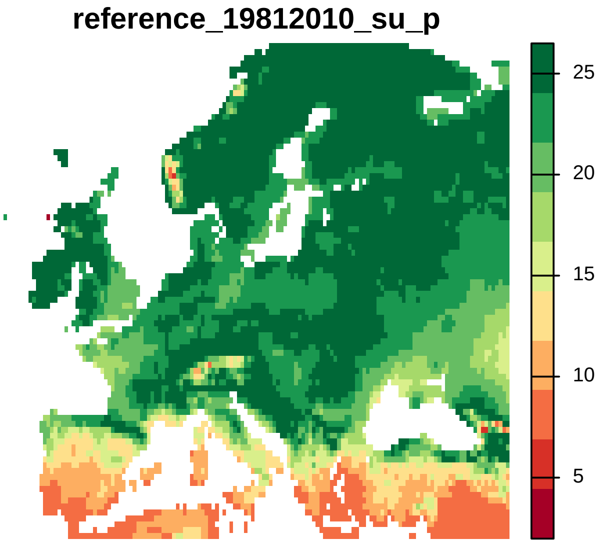

Residual distribution

The multi-panel plot includes a histogram of the residuals (top left), residuals over fitted values (top right), a histogram of observed and predicted values (bottom left) and boxplot diagram of observed and predicted values (bottom right). Observed values are shown in light green, while predicted ones are depicted in light red.

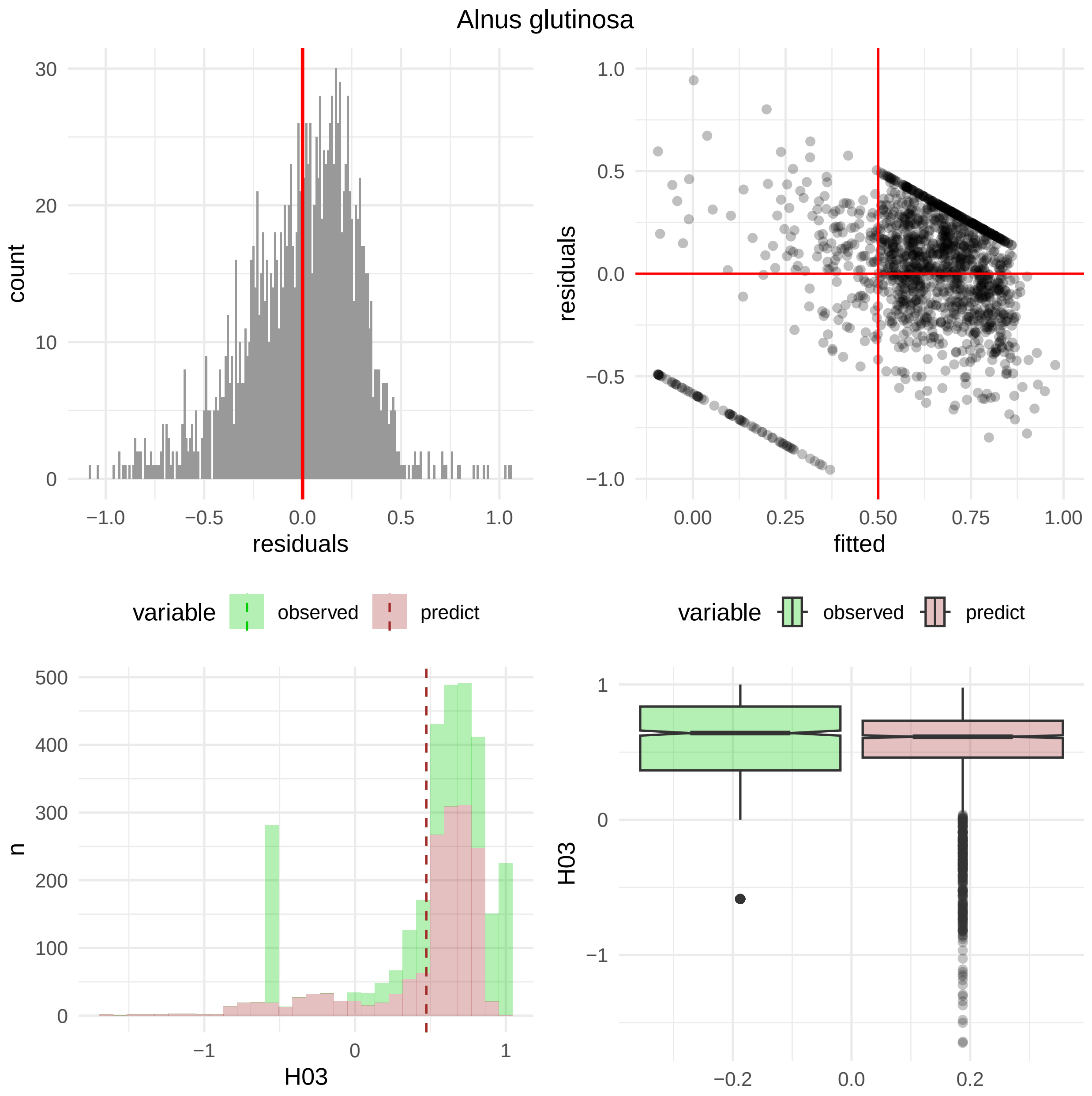

Correlation between predict and observed site index

Relationship between predicted and observed site index (density cloud), as well as linear regressions of presences and absences (= ‘growth absences’) (red line) and presences only (magenta line). The formulas, significance, R2 and number of observations are displayed below for both regressions. Ideally, both the point cloud and the regression lines lie close to the dashed line. For presences only we additionally calculated the correlation coefficient according to PEARSON (cor.pre) in the bottom right corner.

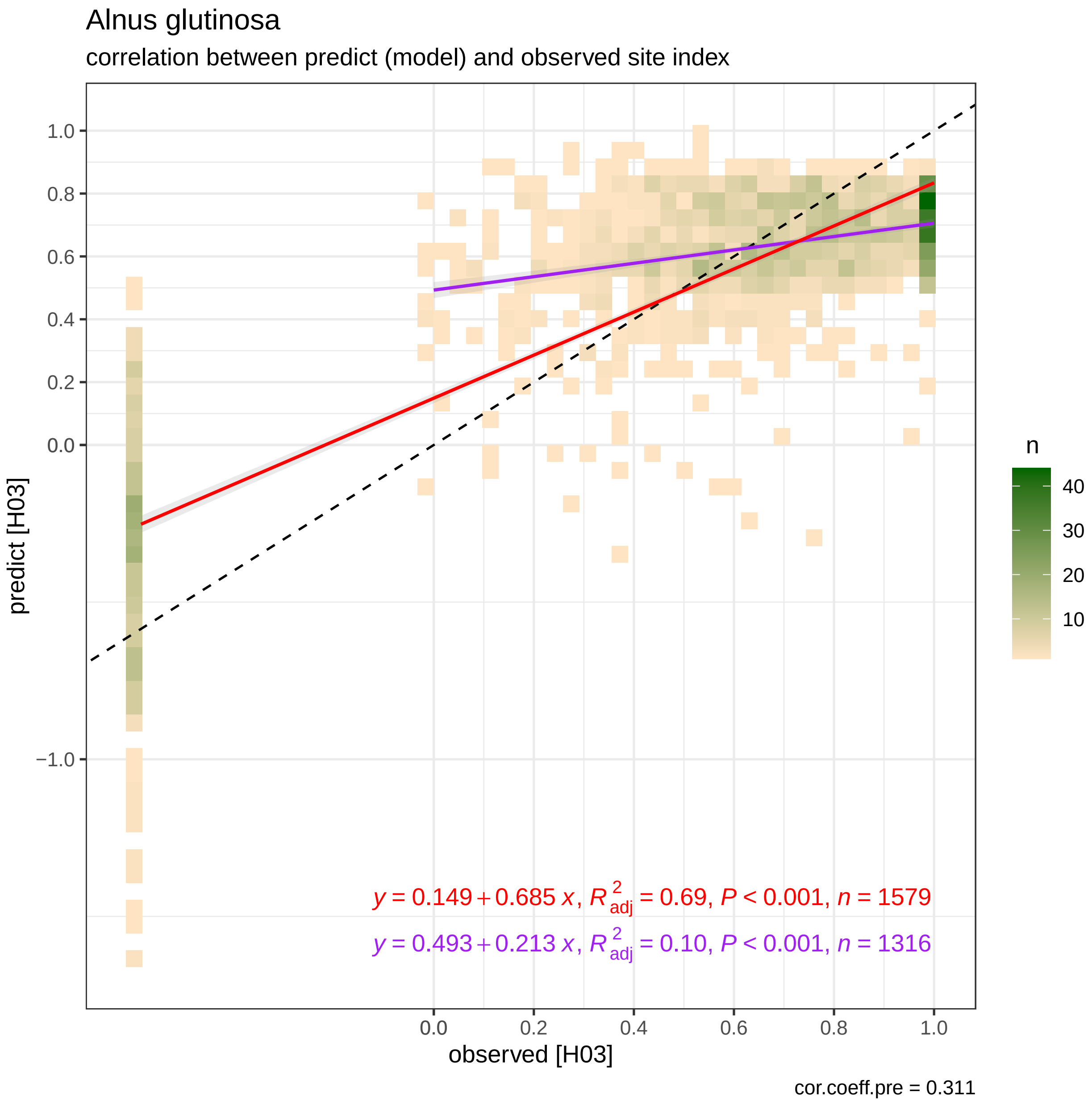

Predictions and forecasts

Predict

European predict for the reference period (1981 to 2010). Dark green symbolizes a high site index (tree height in meters at age 100), orange a lower site index and red no growth. Magenta-coloured dots represent inventory points with growth information, light blue dots are absences (= ‘growth absences’). Results were aggregated on 25 km x 25 km scale.

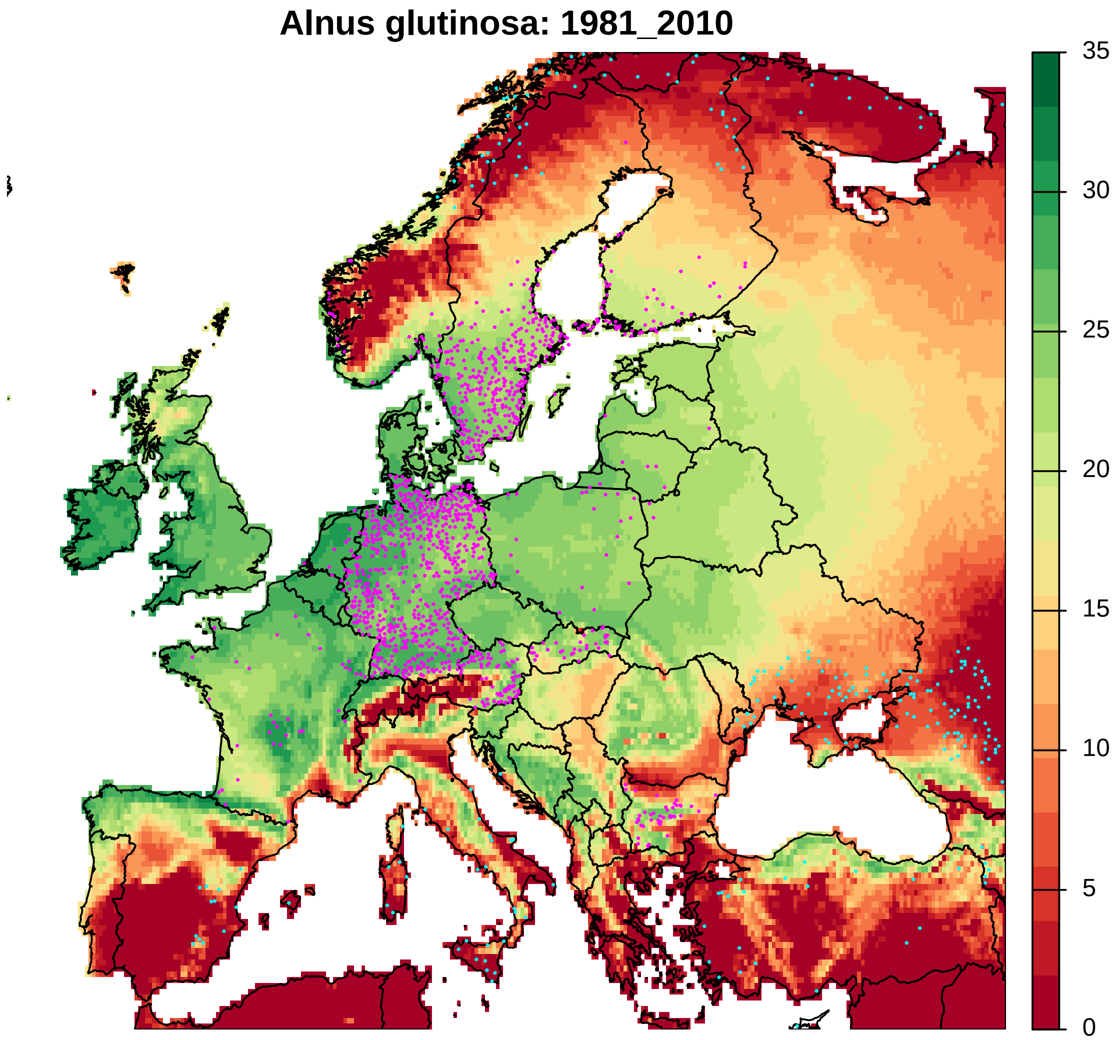

Forecast

Prediction for the reference period (1981 to 2010), as well as forecasts to 2071 to 2100 under szenario RCP4.5 and RCP8.5. Dark green symbolizes a high site index (tree height in m at age 100), orange a lower site index and red no growth. Results were aggregated on 25 km x 25 km scale.

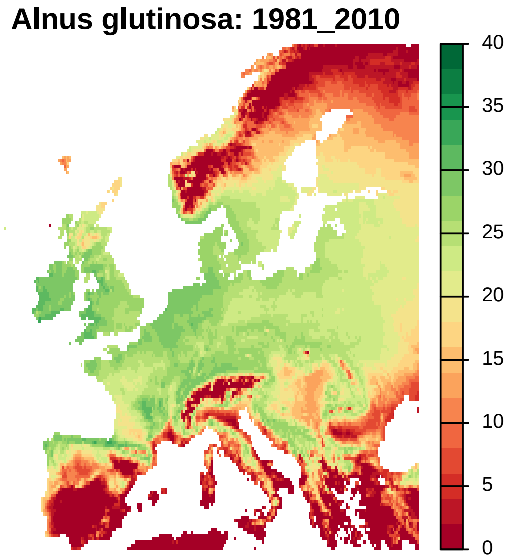

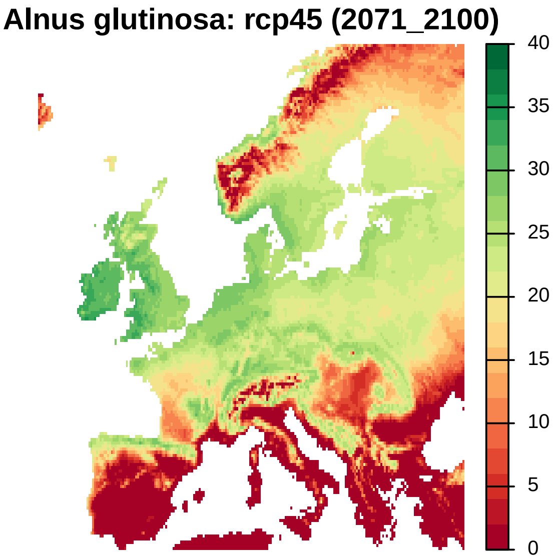

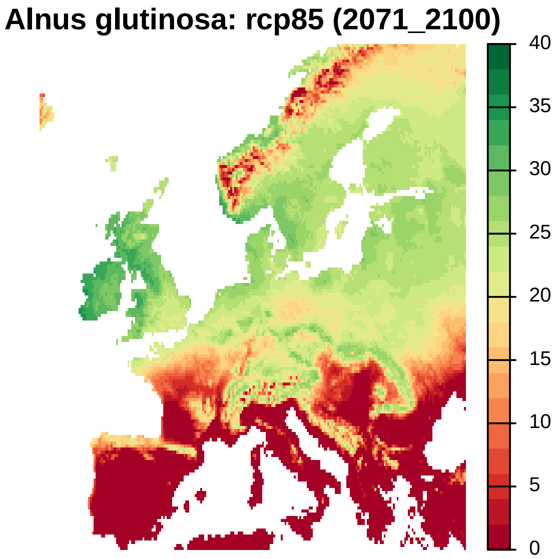

Betula pendula

Site index curves

Site index curves of Betula pendula created with non-linear quantile regressions based on the algorithm of Koenker and Park (1992). The site index (SI) was created by setting all points on the 95 percent quantile (upper line) and above to one (SI = 1) and all on the 5 percent quantile (lower line) and below to zero (SI = 0). The points between the quantile boundaries were assigned a site index between zero and one according to the ratio of their position between the quantile boundaries. We set selected absences (see chapter 2.1.3) on Height = 0 m (at age 100), which means, depending on the site index curves, for each tree species a SI near -1 (red line).

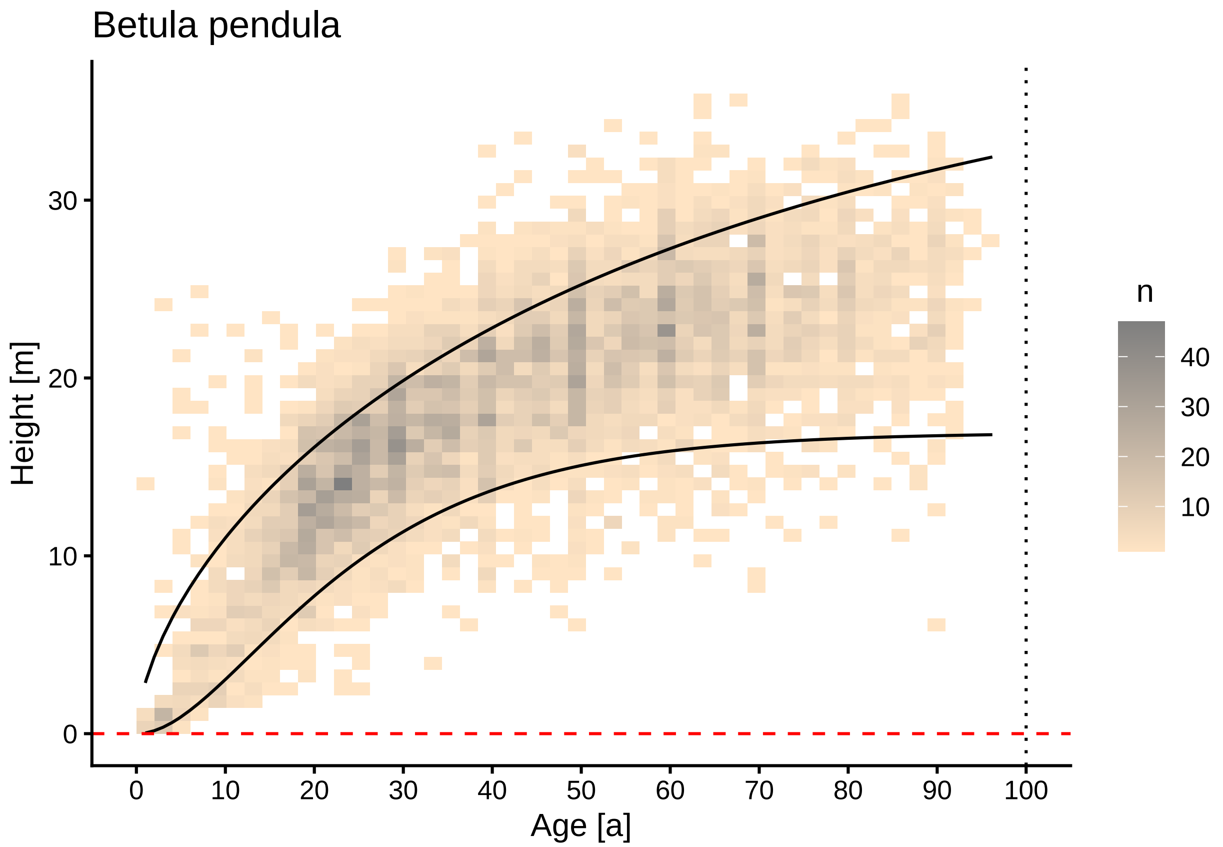

Model statistics and evaluation

Summary

Predictor acronyms: Bio.1 = Mean annual temperature [°C], Bio.12 = Annual precipitation sum [mm/m2], sp_p = Sum of precipitation [mm/m2] within months 3 to 5, su_p = Sum of precipitation [mm/m2] within months 6 to 8, wi_p = Sum of precipitation [mm/m2] within months 12,1,2, sp_t = Mean temperature [°C] within months 3 to 5, su_t = Mean temperature [°C] within months 6 to 8, wi_t = Mean temperature [°C] within months 12,1,2.

```
##
## Family: gaussian
## Link function: identity
##
## Formula:
## H03 ~ s(reference_19812010_su_t, k = 3) + s(reference_19812010_su_p,
##     k = 3)
##
## Parametric coefficients:
##             Estimate Std. Error t value Pr(>|t|)
## (Intercept)  0.39838    0.00737   54.05   <2e-16 ***
## ---
## Signif. codes:  0 '***' 0.001 '**' 0.01 '*' 0.05 '.' 0.1 ' ' 1
##
## Approximate significance of smooth terms:
##                              edf Ref.df      F p-value
## s(reference_19812010_su_t) 2.000      2 1245.4  <2e-16 ***
## s(reference_19812010_su_p) 1.988      2   61.8  <2e-16 ***
## ---
## Signif. codes:  0 '***' 0.001 '**' 0.01 '*' 0.05 '.' 0.1 ' ' 1
##
## R-sq.(adj) =  0.647   Deviance explained = 64.7%
## -REML = 1805.7  Scale est. = 0.17632   n = 3246
```

Variance inflation factor (VIF)

Predictor acronyms: Bio.1 = Mean annual temperature [°C], Bio.12 = Annual precipitation sum [mm/m2], sp_p = Sum of precipitation [mm/m2] within months 3 to 5, su_p = Sum of precipitation [mm/m2] within months 6 to 8, wi_p = Sum of precipitation [mm/m2] within months 12,1,2, sp_t = Mean temperature [°C] within months 3 to 5, su_t = Mean temperature [°C] within months 6 to 8, wi_t = Mean temperature [°C] within months 12,1,2.

```
##                  Variables      VIF
## 1 reference_19812010_su_t 1.687227
## 2 reference_19812010_su_p 1.687227
```

Correlation matrix

Correlation matrix between the predictor variables and the target variable in the model. Correlation coefficient according to PEARSON. Predictor acronyms: Bio.1 = Mean annual temperature [°C], Bio.12 = Annual precipitation sum [mm/m2], sp_p = Sum of precipitation [mm/m2] within months 3 to 5, su_p = Sum of precipitation [mm/m2] within months 6 to 8, wi_p = Sum of precipitation [mm/m2] within months 12,1,2, sp_t = Mean temperature [°C] within months 3 to 5, su_t = Mean temperature [°C] within months 6 to 8, wi_t = Mean temperature [°C] within months 12,1,2.

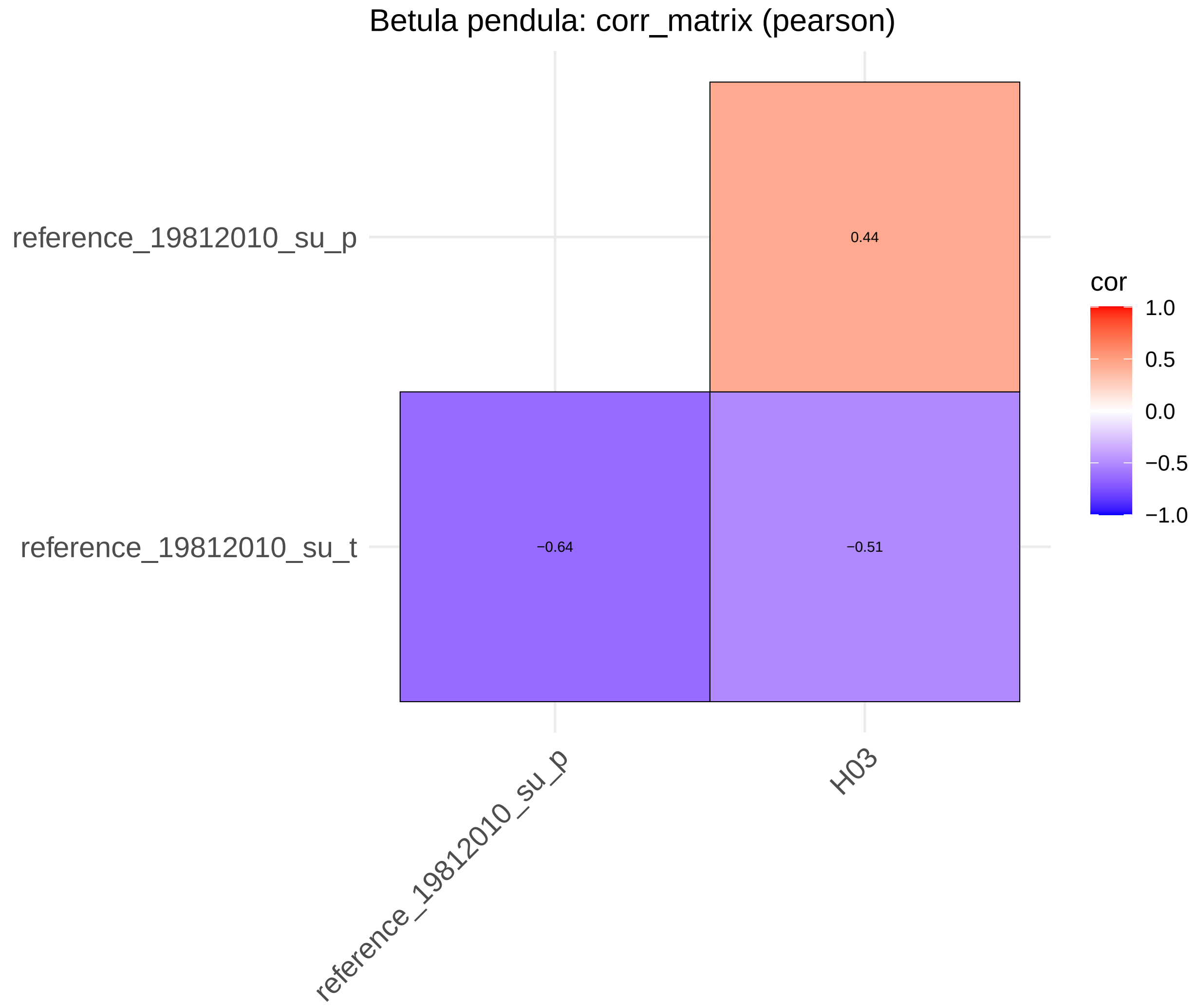

Response curves

Response curves (also known as effect curves) show how each predictor variable affects the target variable (H03 = european Site index, SIrel). H03 values below zero represent 'Growth absences'. Predictor acronyms: Bio.1 = Mean annual temperature [°C], Bio.12 = Annual precipitation sum [mm/m2], sp_p = Sum of precipitation [mm/m2] within months 3 to 5, su_p = Sum of precipitation [mm/m2] within months 6 to 8, wi_p = Sum of precipitation [mm/m2] within months 12,1,2, sp_t = Mean temperature [°C] within months 3 to 5, su_t = Mean temperature [°C] within months 6 to 8, wi_t = Mean temperature [°C] within months 12,1,2.

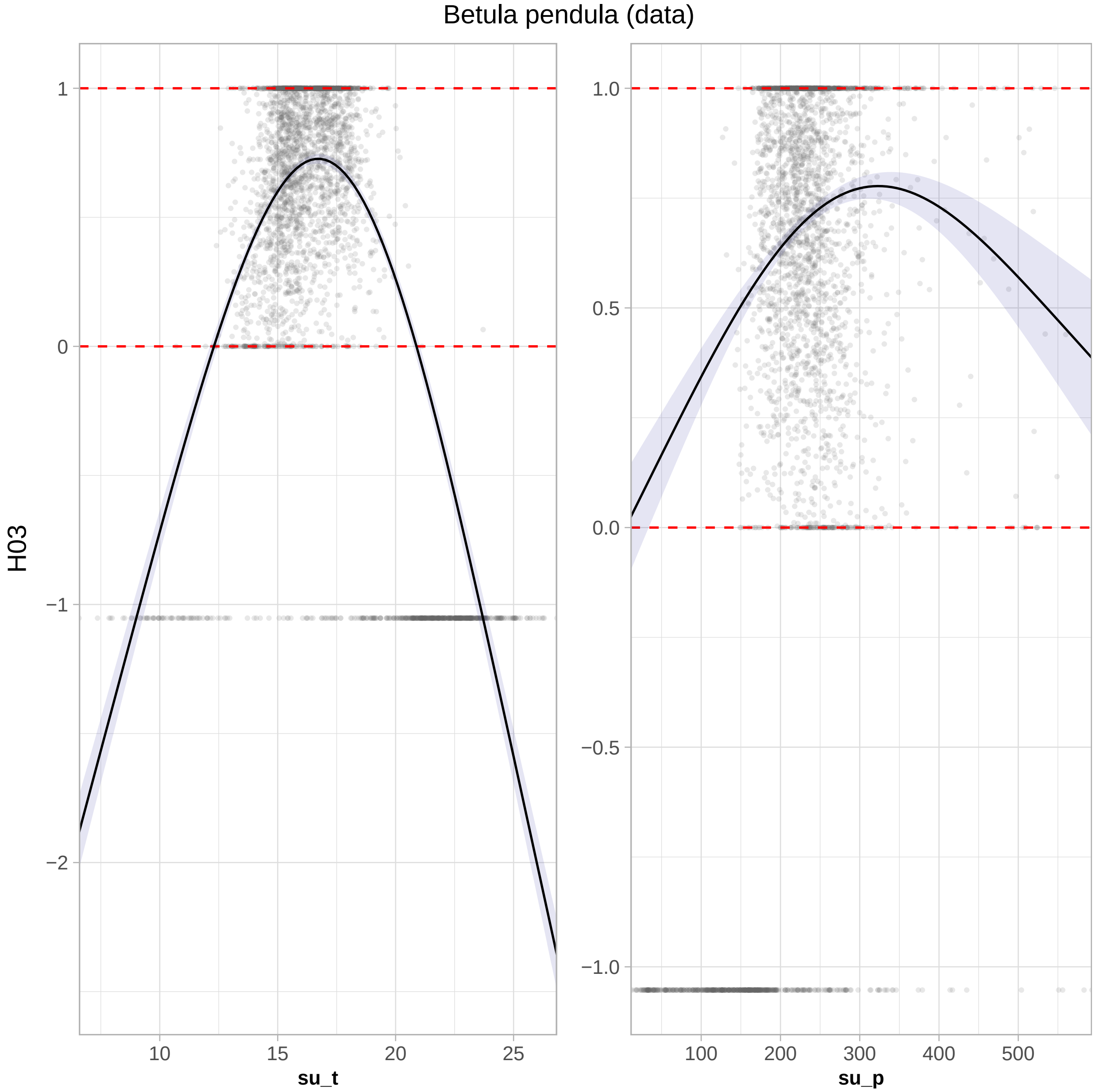

Response maps

Response maps (also referred as partial effect maps). Each map visualizes how a predictor affect the target variable (top height [m] at Age 100). Technically their work like response curves in a geographical area, that is setting all predictor variables except the one shown in the figure on their mean, and mapping the prediction. Predictor acronyms: Bio.1 = Mean annual temperature [°C], Bio.12 = Annual precipitation sum [mm/m2], sp_p = Sum of precipitation [mm/m2] within months 3 to 5, su_p = Sum of precipitation [mm/m2] within months 6 to 8, wi_p = Sum of precipitation [mm/m2] within months 12,1,2, sp_t = Mean temperature [°C] within months 3 to 5, su_t = Mean temperature [°C] within months 6 to 8, wi_t = Mean temperature [°C] within months 12,1,2.

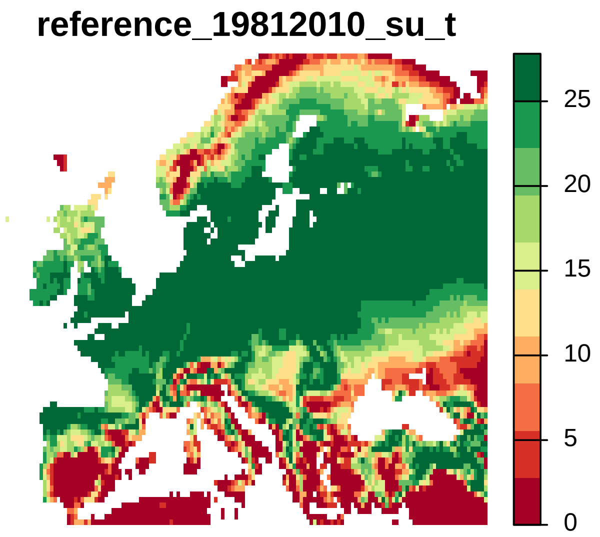

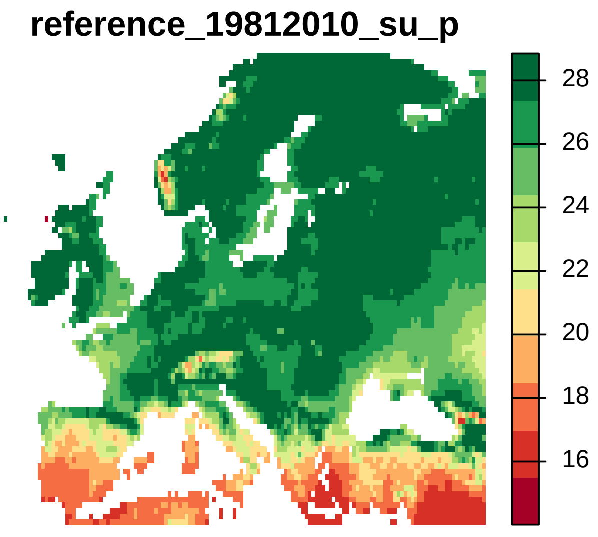

Residual distribution

The multi-panel plot includes a histogram of the residuals (top left), residuals over fitted values (top right), a histogram of observed and predicted values (bottom left) and boxplot diagram of observed and predicted values (bottom right). Observed values are shown in light green, while predicted ones are depicted in light red.

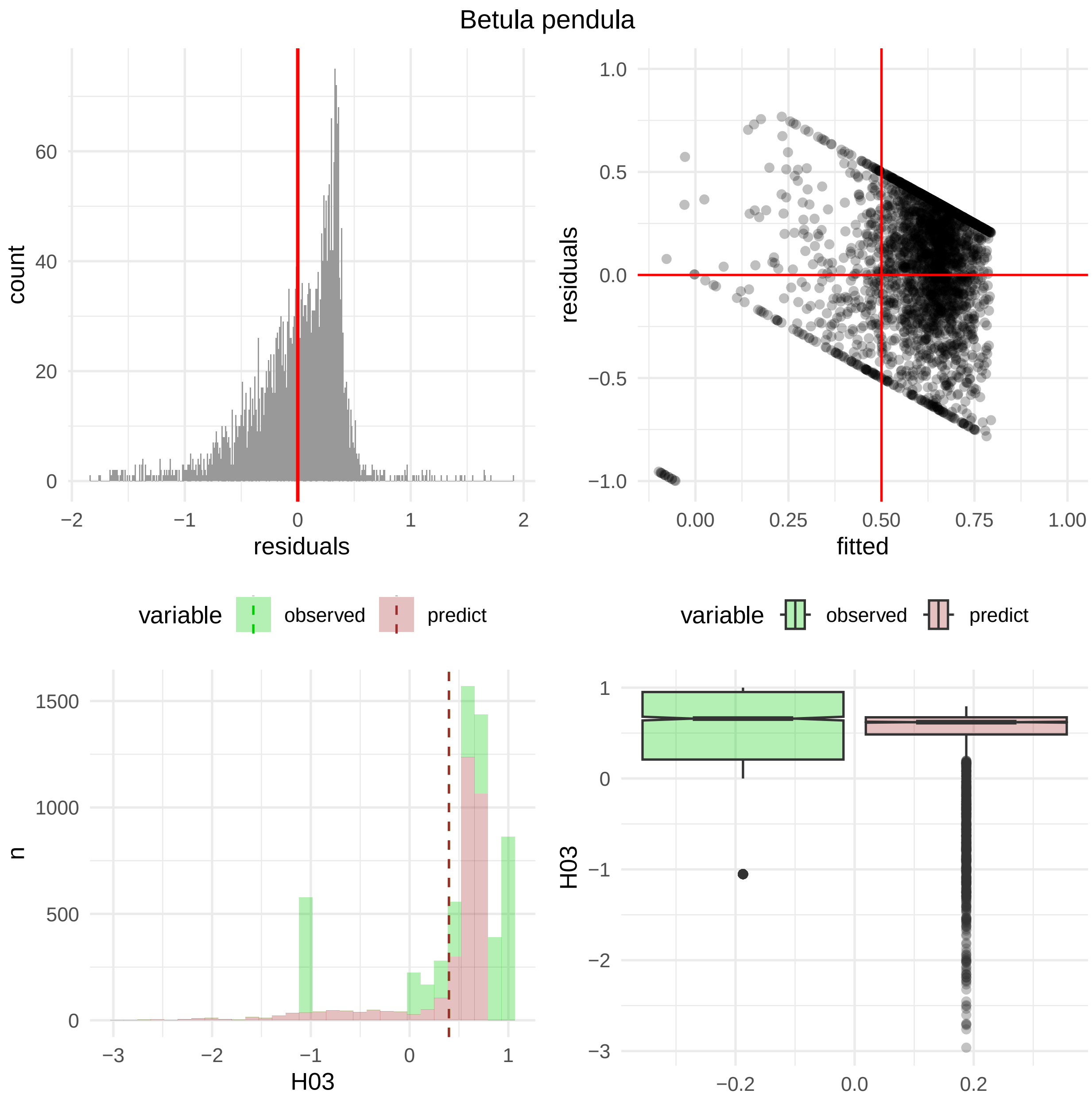

Correlation between predict and observed site index

Relationship between predicted and observed site index (density cloud), as well as linear regressions of presences and absences (= 'growth absences') (red line) and presences only (magenta line). The formulas, significance, R2 and number of observations are displayed below for both regressions. Ideally, both the point cloud and the regression lines lie close to the dashed line. For presences only we additionally calculated the correlation coefficient according to PEARSON (cor.pre) in the bottom right corner.

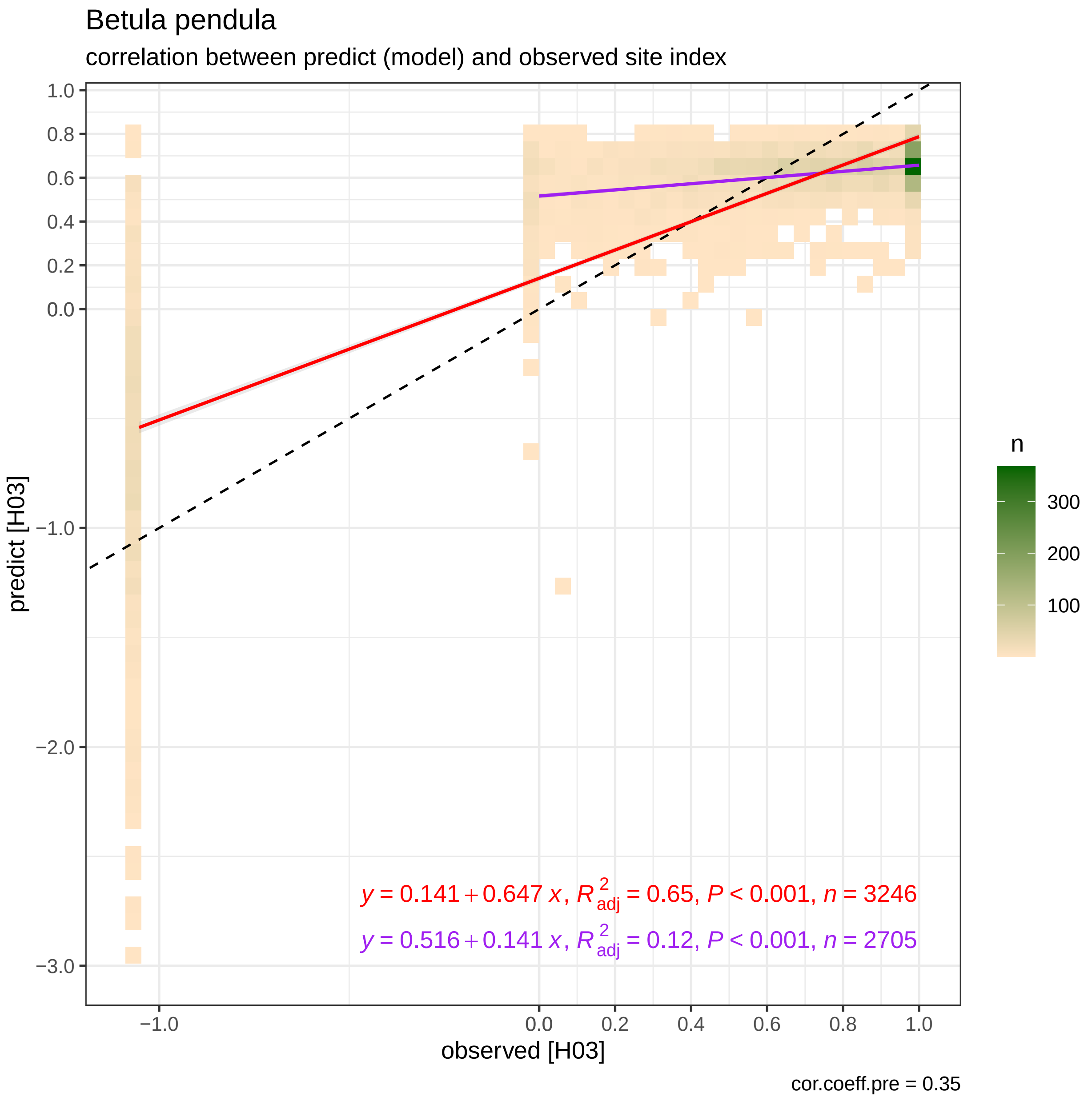

Predictions and forecasts

Predict

European predict for the reference period (1981 to 2010). Dark green symbolizes a high site index (tree height in meters at age 100), orange a lower site index and red no growth. Magenta-coloured dots represent inventory points with growth information, light blue dots are absences (= 'growth absences'). Results were aggregated on 25 km x 25 km scale.

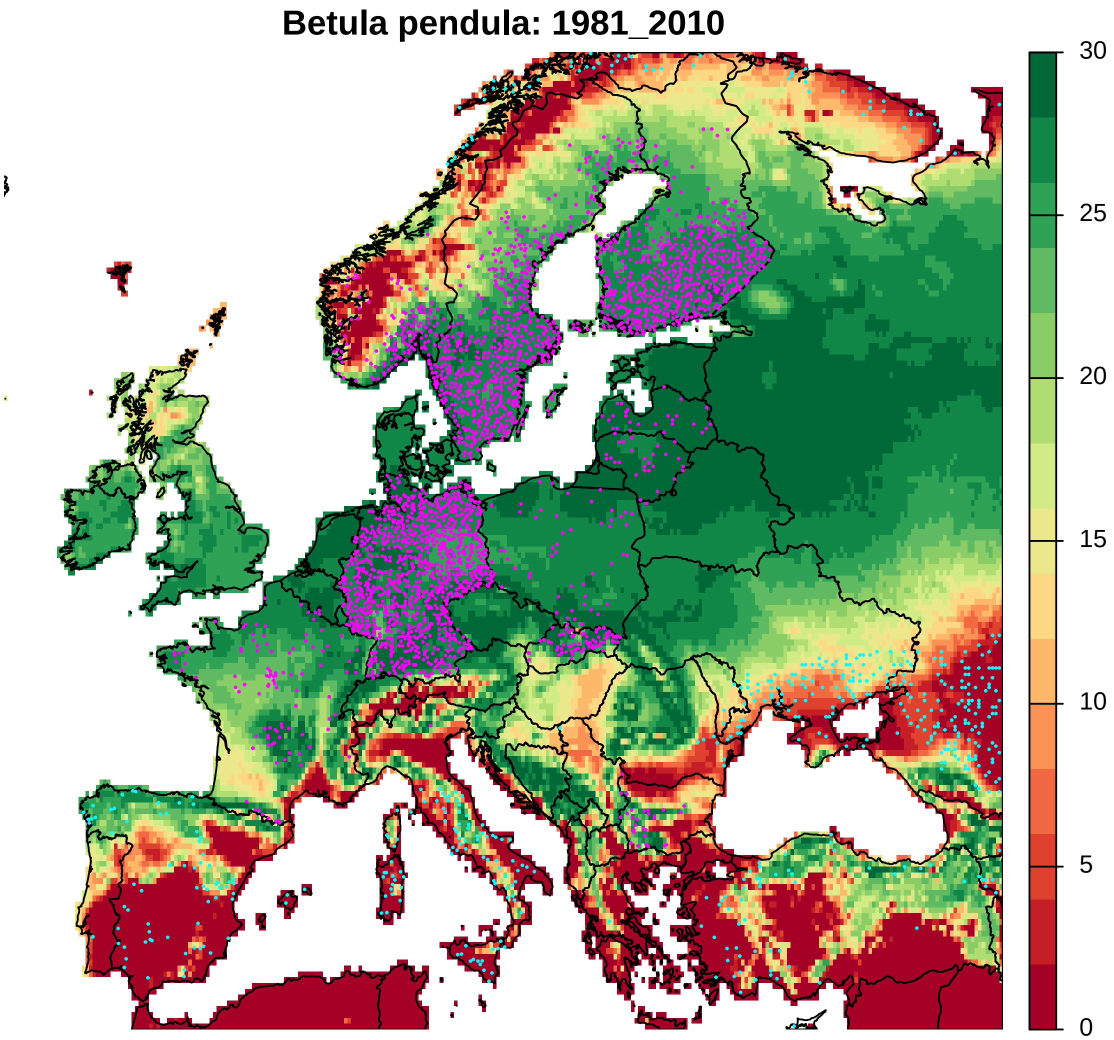

Forecast

Prediction for the reference period (1981 to 2010), as well as forecasts to 2071 to 2100 under szenario RCP4.5 and RCP8.5. Dark green symbolizes a high site index (tree height in m at age 100), orange a lower site index and red no growth. Results were aggregated on 25 km x 25 km scale.

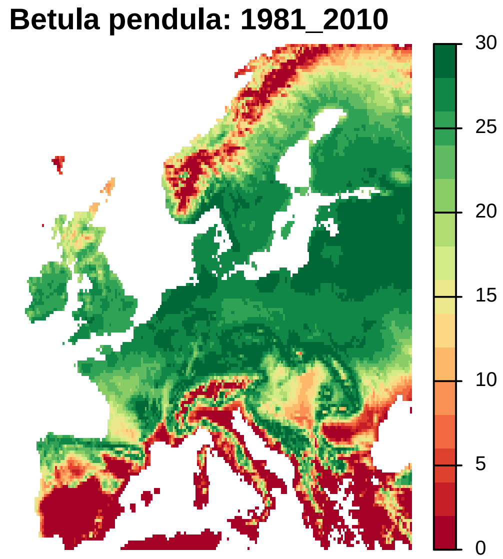

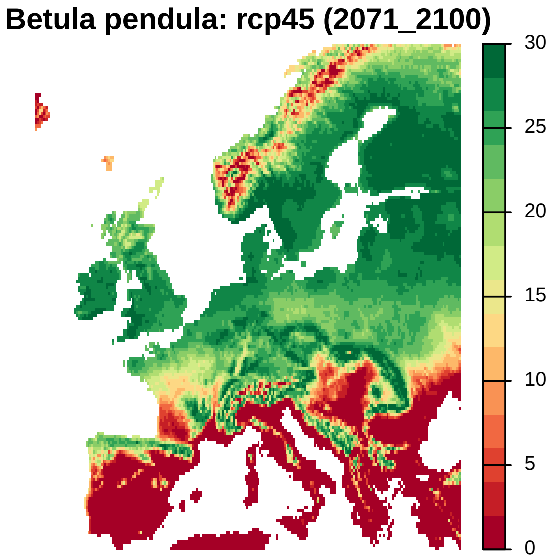

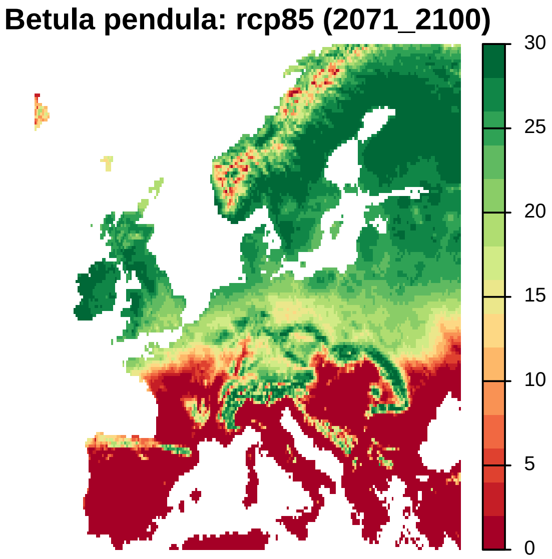

Carpinus betulus

Site index curves

Site index curves of Carpinus betulus created with non-linear quantile regressions based on the algorithm of Koenker and Park (1992). The site index (SI) was created by setting all points on the 95 percent quantile (upper line) and above to one (SI = 1) and all on the 5 percent quantile (lower line) and below to zero (SI = 0). The points between the quantile boundaries were assigned a site index between zero and one according to the ratio of their position between the quantile boundaries. We set selected absences (see chapter 2.1.3) on Height = 0 m (at age 100), which means, depending on the site index curves, for each tree species a SI near -1 (red line).

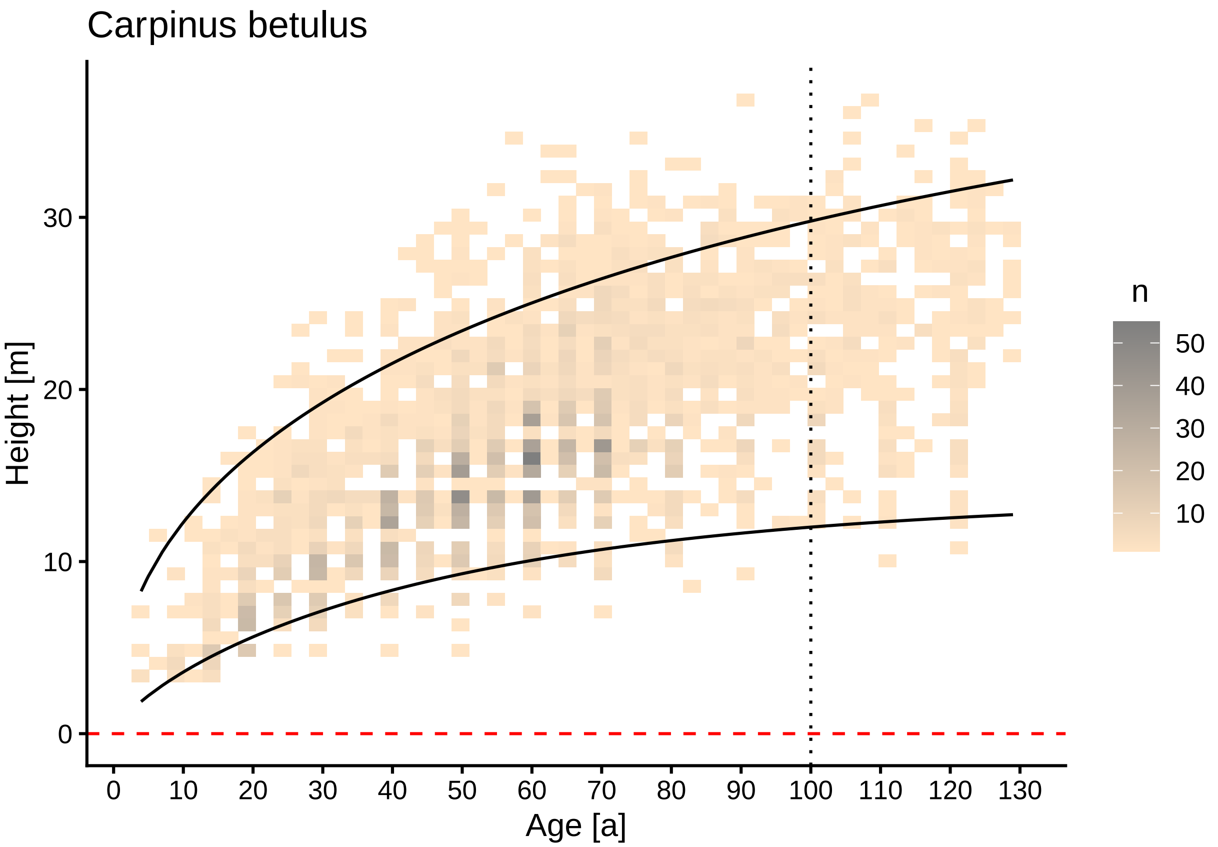

Model statistics and evaluation

Summary

Predictor acronyms: Bio.1 = Mean annual temperature [°C], Bio.12 = Annual precipitation sum [mm/m2], sp_p = Sum of precipitation [mm/m2] within months 3 to 5, su_p = Sum of precipitation [mm/m2] within months 6 to 8, wi_p = Sum of precipitation [mm/m2] within months 12,1,2, sp_t = Mean temperature [°C] within months 3 to 5, su_t = Mean temperature [°C] within months 6 to 8, wi_t = Mean temperature [°C] within months 12,1,2.

```
##
## Family: gaussian
## Link function: identity
##
## Formula:
## H03 ~ s(reference_19812010_wi_t, k = 3) + s(reference_19812010_su_t,
##     k = 3) + s(reference_19812010_su_p, k = 3)
##
## Parametric coefficients:
##             Estimate Std. Error t value Pr(>|t|)
## (Intercept)  0.45172    0.01002   45.09   <2e-16 ***
## ---
## Signif. codes:  0 '***' 0.001 '**' 0.01 '*' 0.05 '.' 0.1 ' ' 1
##
## Approximate significance of smooth terms:
##                              edf Ref.df      F p-value
## s(reference_19812010_wi_t) 1.989      2 217.91  <2e-16 ***
## s(reference_19812010_su_t) 1.990      2  55.69  <2e-16 ***
## s(reference_19812010_su_p) 1.992      2  77.45  <2e-16 ***
## ---
## Signif. codes:  0 '***' 0.001 '**' 0.01 '*' 0.05 '.' 0.1 ' ' 1
##
## R-sq.(adj) =  0.591   Deviance explained = 59.3%
## -REML = 509.16  Scale est. = 0.12696   n = 1265
```

Variance inflation factor (VIF)

Predictor acronyms: Bio.1 = Mean annual temperature [°C], Bio.12 = Annual precipitation sum [mm/m2], sp_p = Sum of precipitation [mm/m2] within months 3 to 5, su_p = Sum of precipitation [mm/m2] within months 6 to 8, wi_p = Sum of precipitation [mm/m2] within months 12,1,2, sp_t = Mean temperature [°C] within months 3 to 5, su_t = Mean temperature [°C] within months 6 to 8, wi_t = Mean temperature [°C] within months 12,1,2.

```
##                   Variables      VIF
## 1 reference_19812010_wi_t 1.461001
## 2 reference_19812010_su_t 1.732879
## 3 reference_19812010_su_p 1.412391
```

Correlation matrix

Correlation matrix between the predictor variables and the target variable in the model. Correlation coefficient according to PEARSON. Predictor acronyms: Bio.1 = Mean annual temperature [°C], Bio.12 = Annual precipitation sum [mm/m2], sp_p = Sum of precipitation [mm/m2] within months 3 to 5, su_p = Sum of precipitation [mm/m2] within months 6 to 8, wi_p = Sum of precipitation [mm/m2] within months 12,1,2, sp_t = Mean temperature [°C] within months 3 to 5, su_t = Mean temperature [°C] within months 6 to 8, wi_t = Mean temperature [°C] within months 12,1,2.

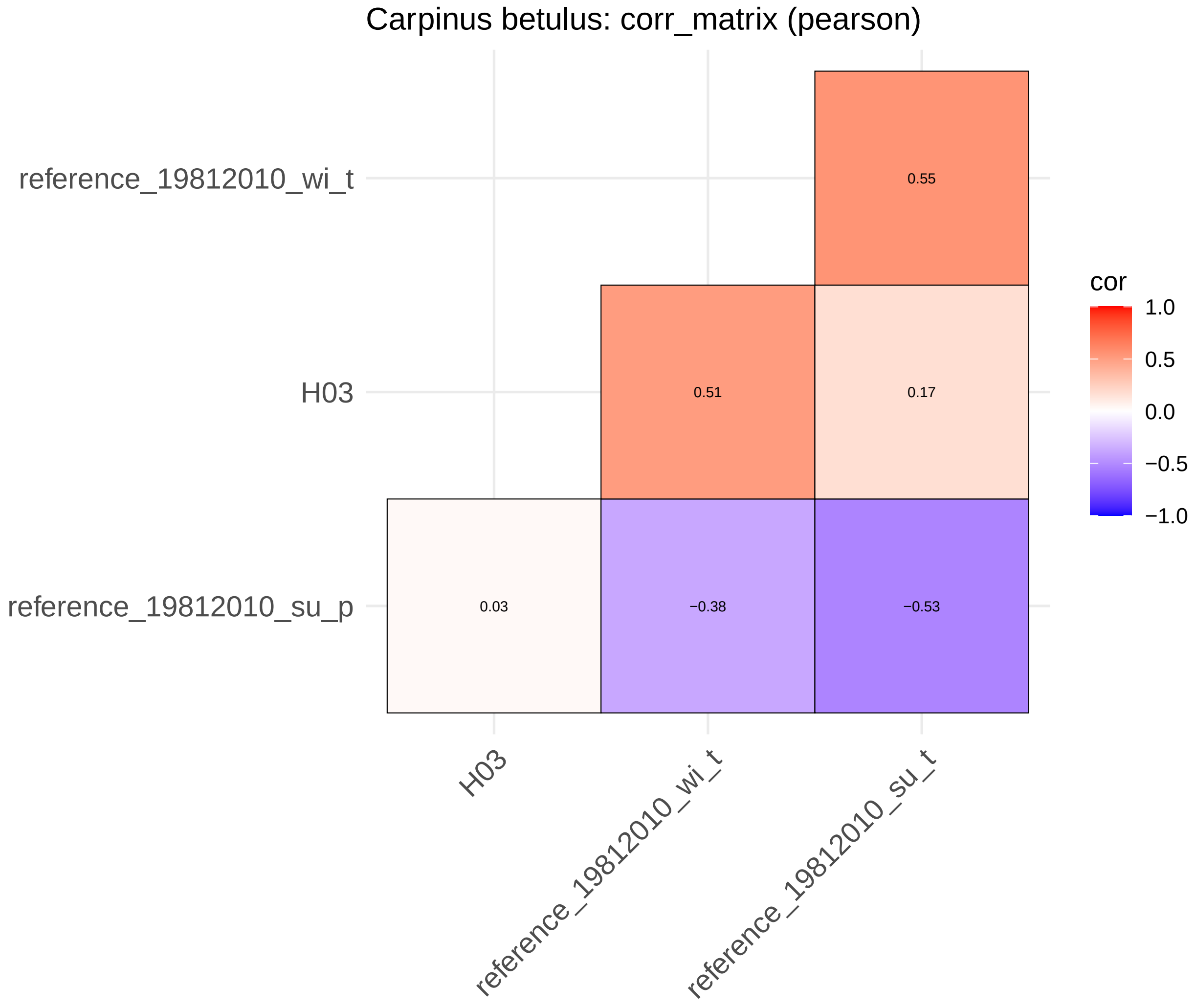

Response curves

Response curves (also known as effect curves) show how each predictor variable affects the target variable (H03 = european Site index, SIrel). H03 values below zero represent 'Growth absences'. Predictor acronyms: Bio.1 = Mean annual temperature [°C], Bio.12 = Annual precipitation sum [mm/m2], sp_p = Sum of precipitation [mm/m2] within months 3 to 5, su_p = Sum of precipitation [mm/m2] within months 6 to 8, wi_p = Sum of precipitation [mm/m2] within months 12,1,2, sp_t = Mean temperature [°C] within months 3 to 5, su_t = Mean temperature [°C] within months 6 to 8, wi_t = Mean temperature [°C] within months 12,1,2.

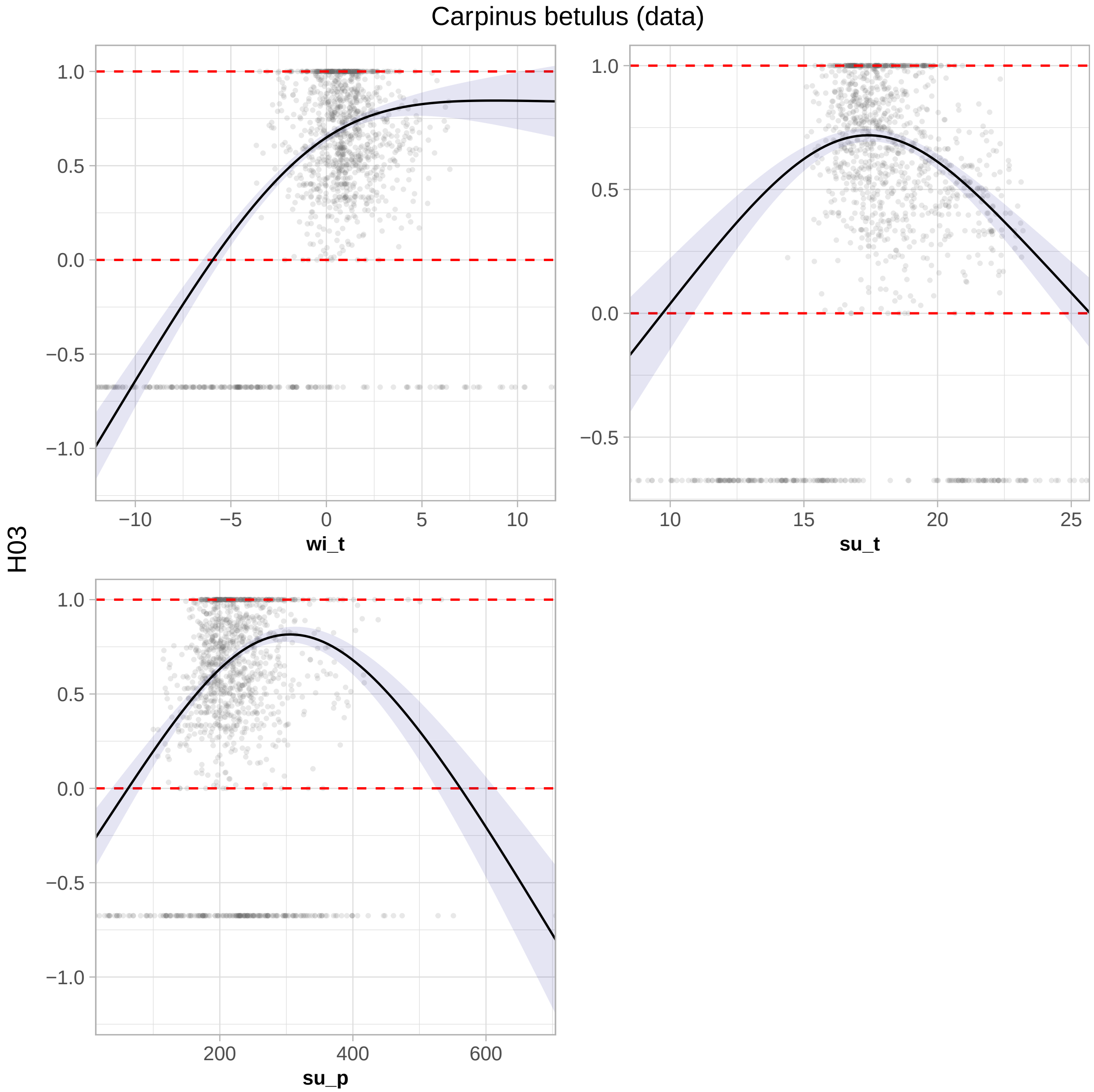

Response maps

Response maps (also referred as partial effect maps). Each map visualizes how a predictor affect the target variable (top height [m] at Age 100). Technically their work like response curves in a geographical area, that is setting all predictor variables except the one shown in the figure on their mean, and mapping the prediction. Predictor acronyms: Bio.1 = Mean annual temperature [°C], Bio.12 = Annual precipitation sum [mm/m2], sp_p = Sum of precipitation [mm/m2] within months 3 to 5, su_p = Sum of precipitation [mm/m2] within months 6 to 8, wi_p = Sum of precipitation [mm/m2] within months 12,1,2, sp_t = Mean temperature [°C] within months 3 to 5, su_t = Mean temperature [°C] within months 6 to 8, wi_t = Mean temperature [°C] within months 12,1,2.

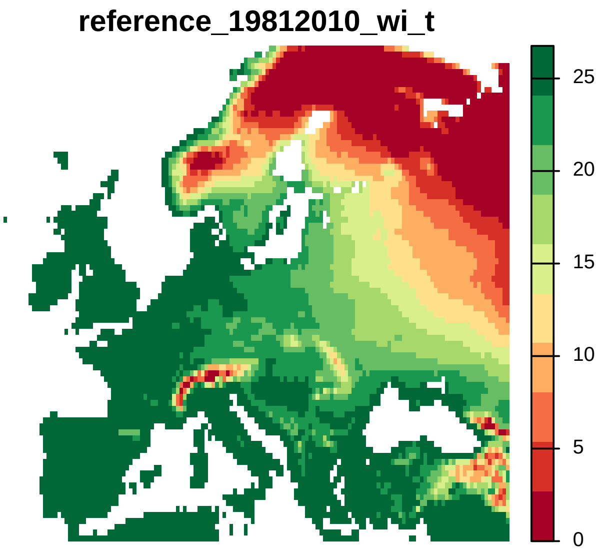

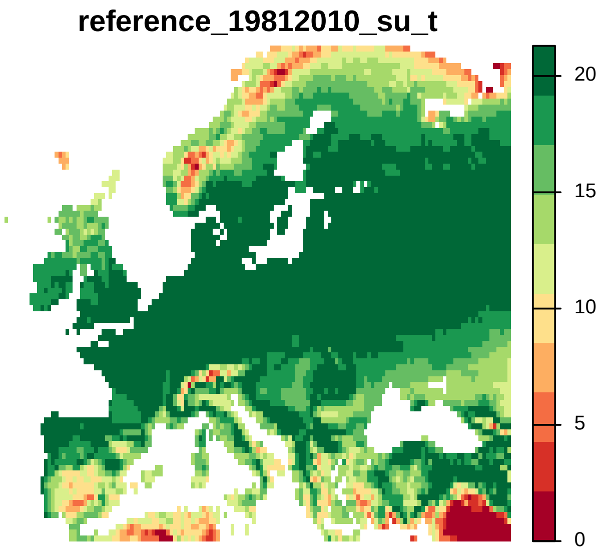

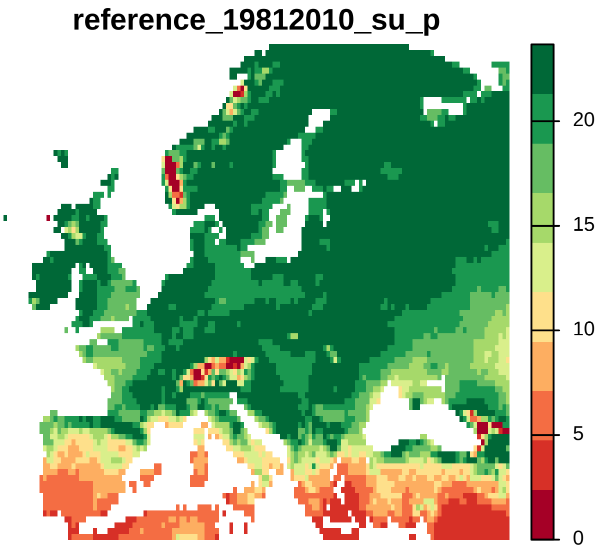

Residual distribution

The multi-panel plot includes a histogram of the residuals (top left), residuals over fitted values (top right), a histogram of observed and predicted values (bottom left) and boxplot diagram of observed and predicted values (bottom right). Observed values are shown in light green, while predicted ones are depicted in light red.

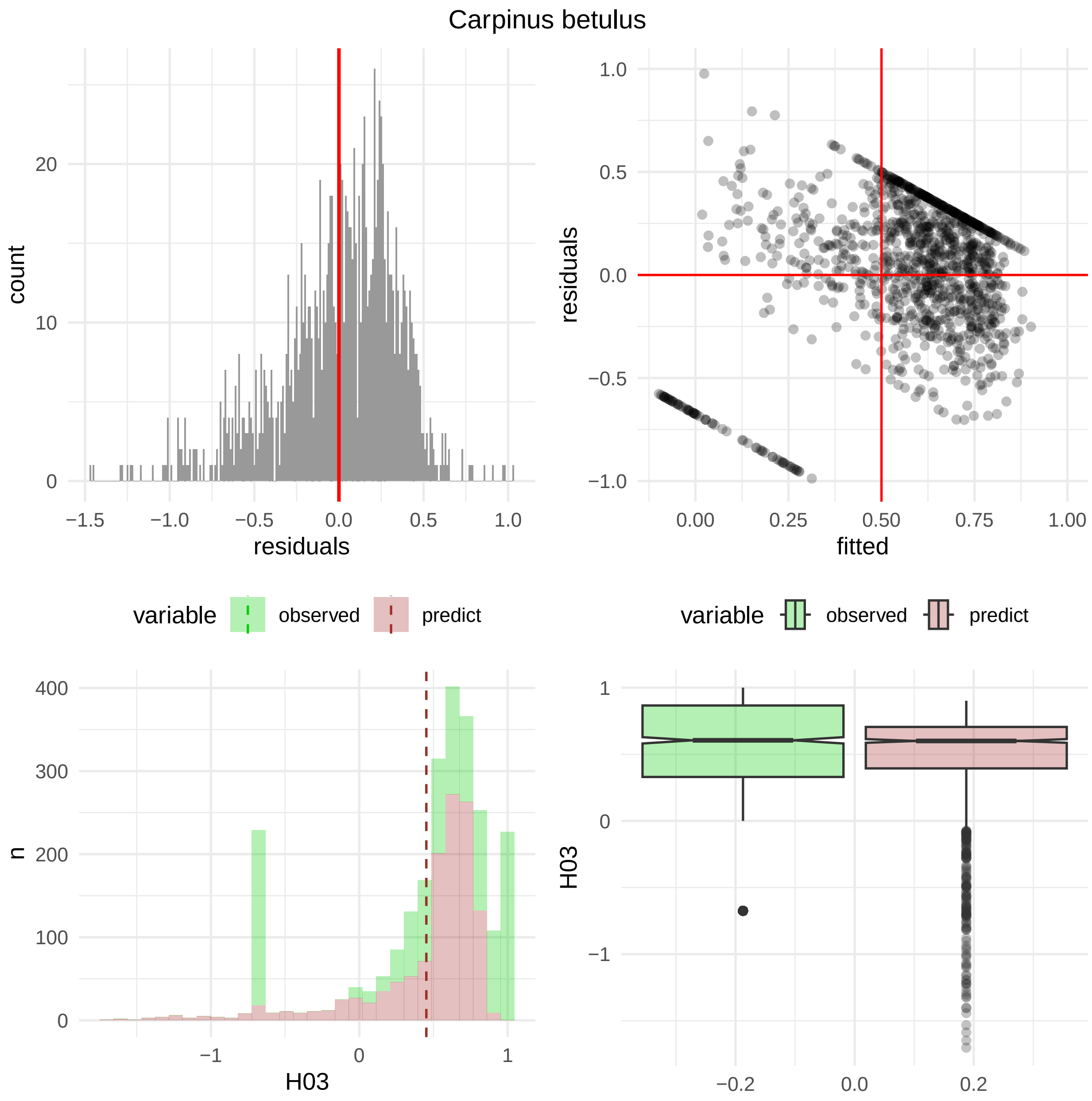

Correlation between predict and observed site index

Relationship between predicted and observed site index (density cloud), as well as linear regressions of presences and absences (= 'growth absences') (red line) and presences only (magenta line). The formulas, significance, R2 and number of observations are displayed below for both regressions. Ideally, both the point cloud and the regression lines lie close to the dashed line. For presences only we additionally calculated the correlation coefficient according to PEARSON (cor.pre) in the bottom right corner.

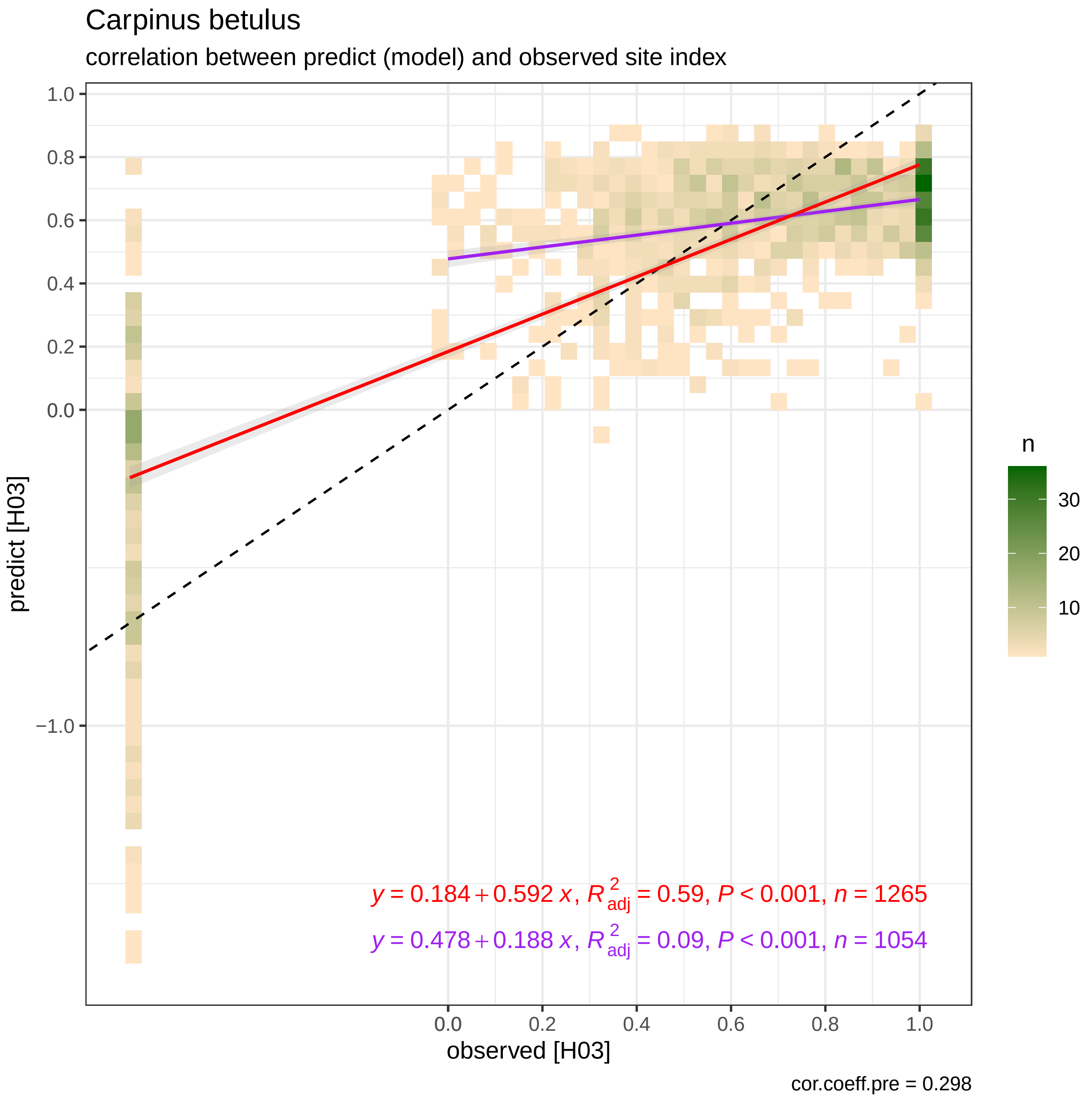

Predictions and forecasts

Predict

European predict for the reference period (1981 to 2010). Dark green symbolizes a high site index (tree height in meters at age 100), orange a lower site index and red no growth. Magenta-coloured dots represent inventory points with growth information, light blue dots are absences (= 'growth absences'). Results were aggregated on 25 km x 25 km scale.

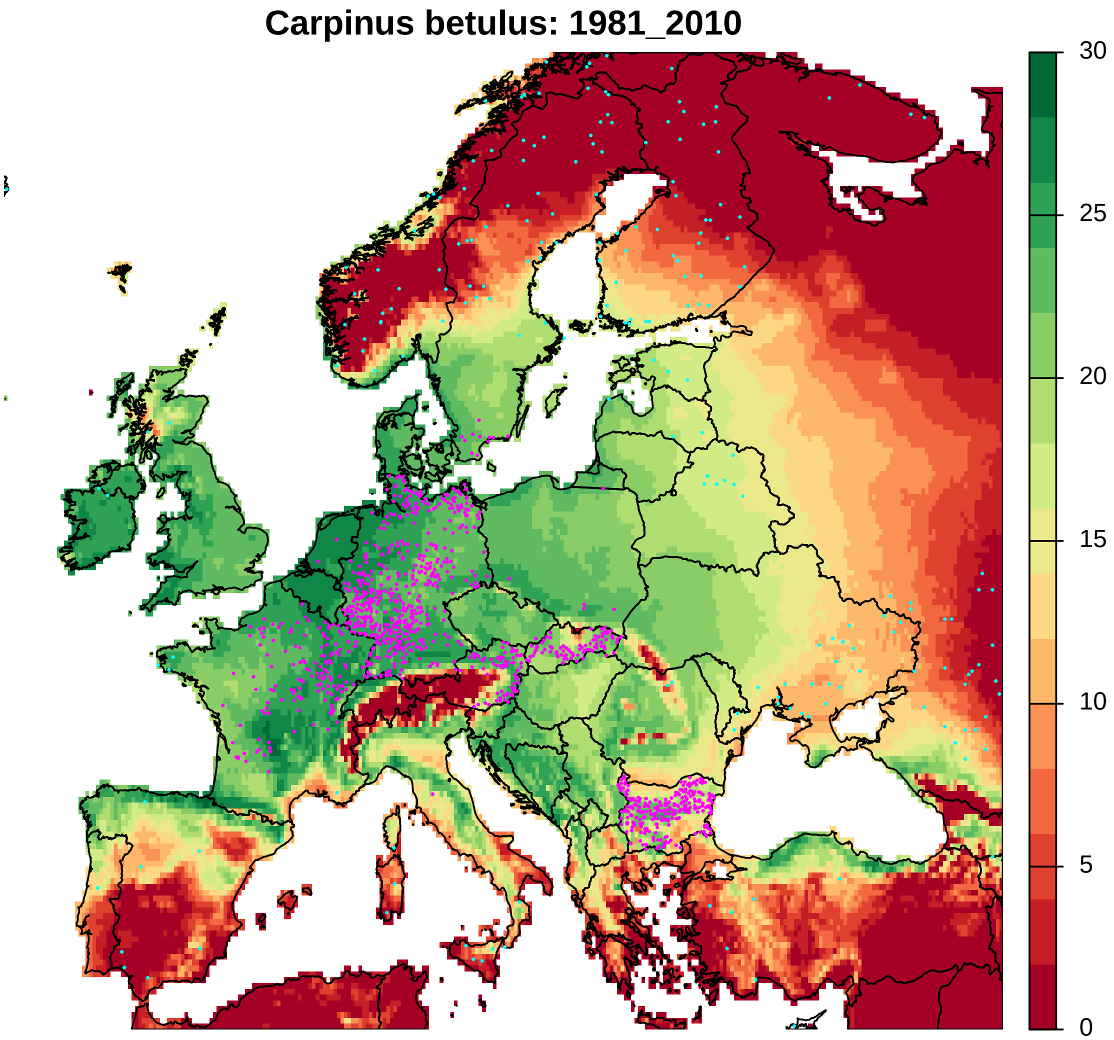

Forecast

Prediction for the reference period (1981 to 2010), as well as forecasts to 2071 to 2100 under szenario RCP4.5 and RCP8.5. Dark green symbolizes a high site index (tree height in m at age 100), orange a lower site index and red no growth. Results were aggregated on 25 km x 25 km scale.

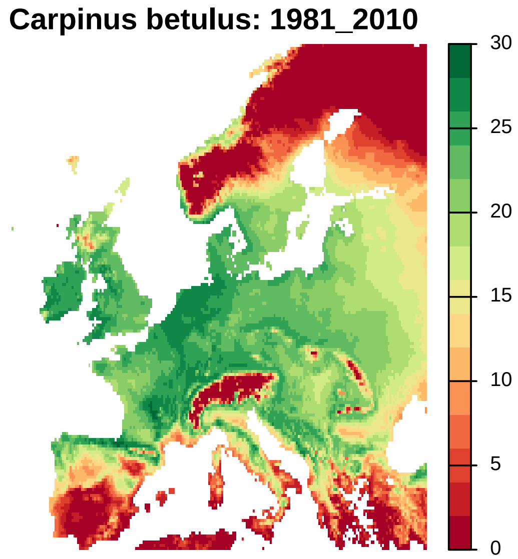

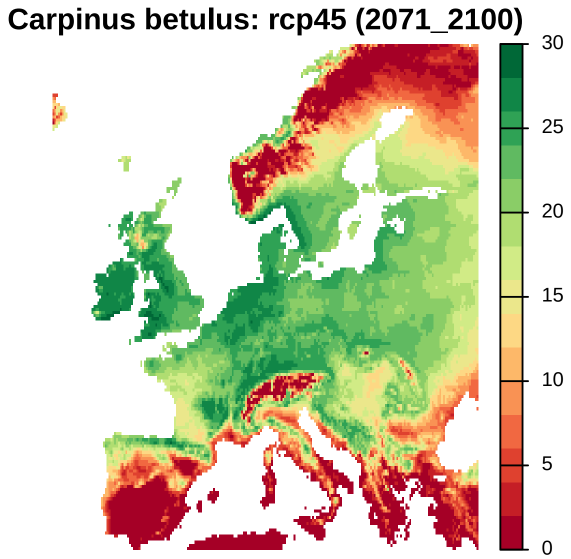

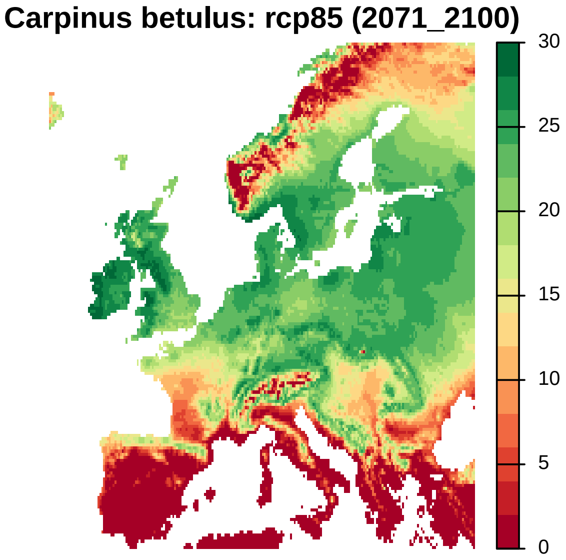

Castanea sativa

Site index curves

Site index curves of Castanea sativa created with non-linear quantile regressions based on the algorithm of Koenker and Park (1992). The site index (SI) was created by setting all points on the 95 percent quantile (upper line) and above to one (SI = 1) and all on the 5 percent quantile (lower line) and below to zero (SI = 0). The points between the quantile boundaries were assigned a site index between zero and one according to the ratio of their position between the quantile boundaries. We set selected absences (see chapter 2.1.3) on Height = 0 m (at age 100), which means, depending on the site index curves, for each tree species a SI near -1 (red line).

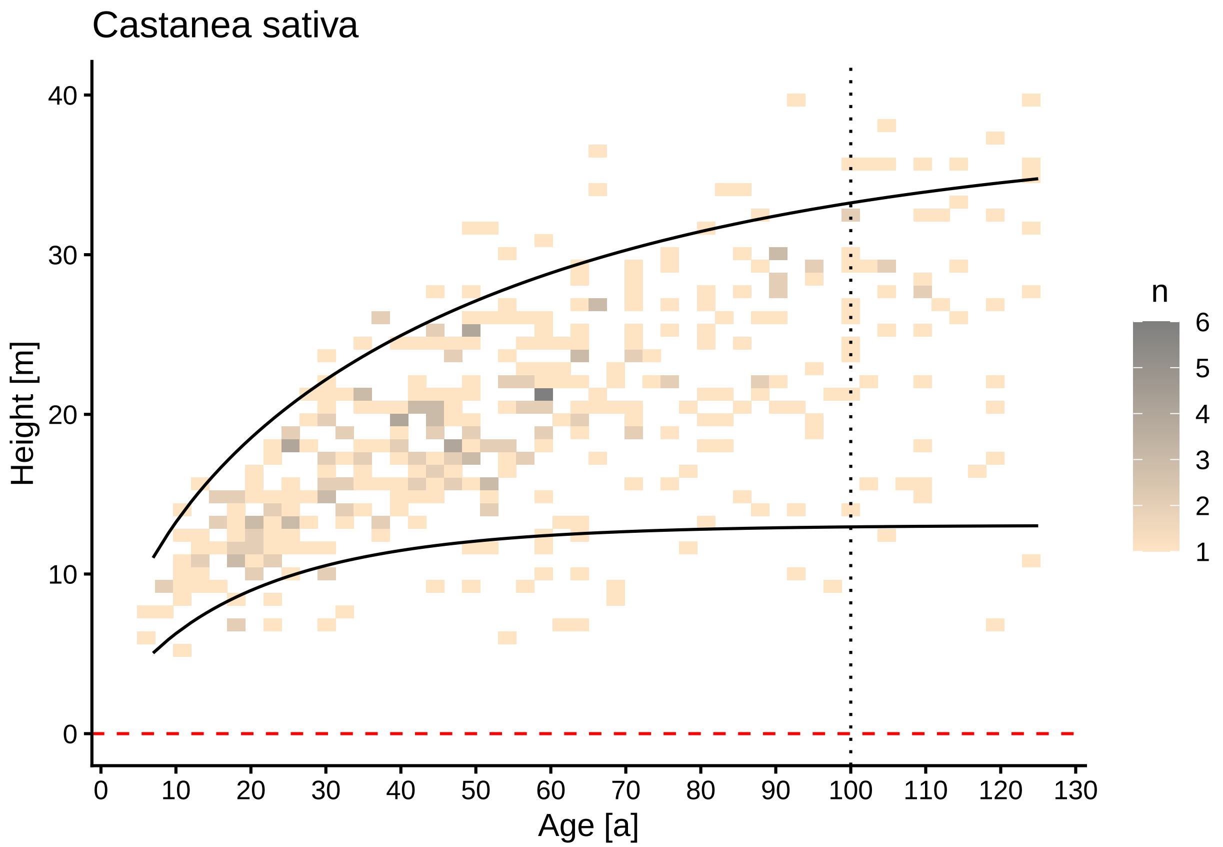

Model statistics and evaluation

Summary

Predictor acronyms: Bio.1 = Mean annual temperature [°C], Bio.12 = Annual precipitation sum [mm/m2], sp_p = Sum of precipitation [mm/m2] within months 3 to 5, su_p = Sum of precipitation [mm/m2] within months 6 to 8, wi_p = Sum of precipitation [mm/m2] within months 12,1,2, sp_t = Mean temperature [°C] within months 3 to 5, su_t = Mean temperature [°C] within months 6 to 8, wi_t = Mean temperature [°C] within months 12,1,2.

```
##
## Family: gaussian
## Link function: identity
##
## Formula:
## H03 ~ s(reference_19812010_su_t, k = 3) + s(reference_19812010_wi_t,
##     k = 3) + s(reference_19812010_sp_p, k = 3) + s(reference_19812010_su_p,
##     k = 3)
##
## Parametric coefficients:
##             Estimate Std. Error t value Pr(>|t|)
## (Intercept)   0.3333     0.0183   18.21   <2e-16 ***
## ---
## Signif. codes:  0 '***' 0.001 '**' 0.01 '*' 0.05 '.' 0.1 ' ' 1
##
## Approximate significance of smooth terms:
##                              edf Ref.df     F  p-value
## s(reference_19812010_su_t) 1.917  1.992 19.07  < 2e-16 ***
## s(reference_19812010_wi_t) 1.957  1.997 36.42  < 2e-16 ***
## s(reference_19812010_sp_p) 1.970  1.999 19.22  < 2e-16 ***
## s(reference_19812010_su_p) 1.000  1.000 23.15 2.93e-06 ***
## ---
## Signif. codes:  0 '***' 0.001 '**' 0.01 '*' 0.05 '.' 0.1 ' ' 1
##
## R-sq.(adj) =  0.581   Deviance explained =   59%
## -REML = 108.84  Scale est. = 0.10515   n = 314
```

Variance inflation factor (VIF)

Predictor acronyms: Bio.1 = Mean annual temperature [°C], Bio.12 = Annual precipitation sum [mm/m2], sp_p = Sum of precipitation [mm/m2] within months 3 to 5, su_p = Sum of precipitation [mm/m2] within months 6 to 8, wi_p = Sum of precipitation [mm/m2] within months 12,1,2, sp_t = Mean temperature [°C] within months 3 to 5, su_t = Mean temperature [°C] within months 6 to 8, wi_t = Mean temperature [°C] within months 12,1,2.

```
##                  Variables      VIF
## 1 reference_19812010_su_t 1.651358
## 2 reference_19812010_wi_t 2.740455
## 3 reference_19812010_sp_p 2.502621
## 4 reference_19812010_su_p 2.757532
```

Correlation matrix

Correlation matrix between the predictor variables and the target variable in the model. Correlation coefficient according to PEARSON. Predictor acronyms: Bio.1 = Mean annual temperature [°C], Bio.12 = Annual precipitation sum [mm/m2], sp__p = Sum of precipitation [mm/m2] within months 3 to 5, su__p = Sum of precipitation [mm/m2] within months 6 to 8, wi__p = Sum of precipitation [mm/m2] within months 12,1,2, sp__t = Mean temperature [°C] within months 3 to 5, su__t = Mean temperature [°C] within months 6 to 8, wi__t = Mean temperature [°C] within months 12,1,2.

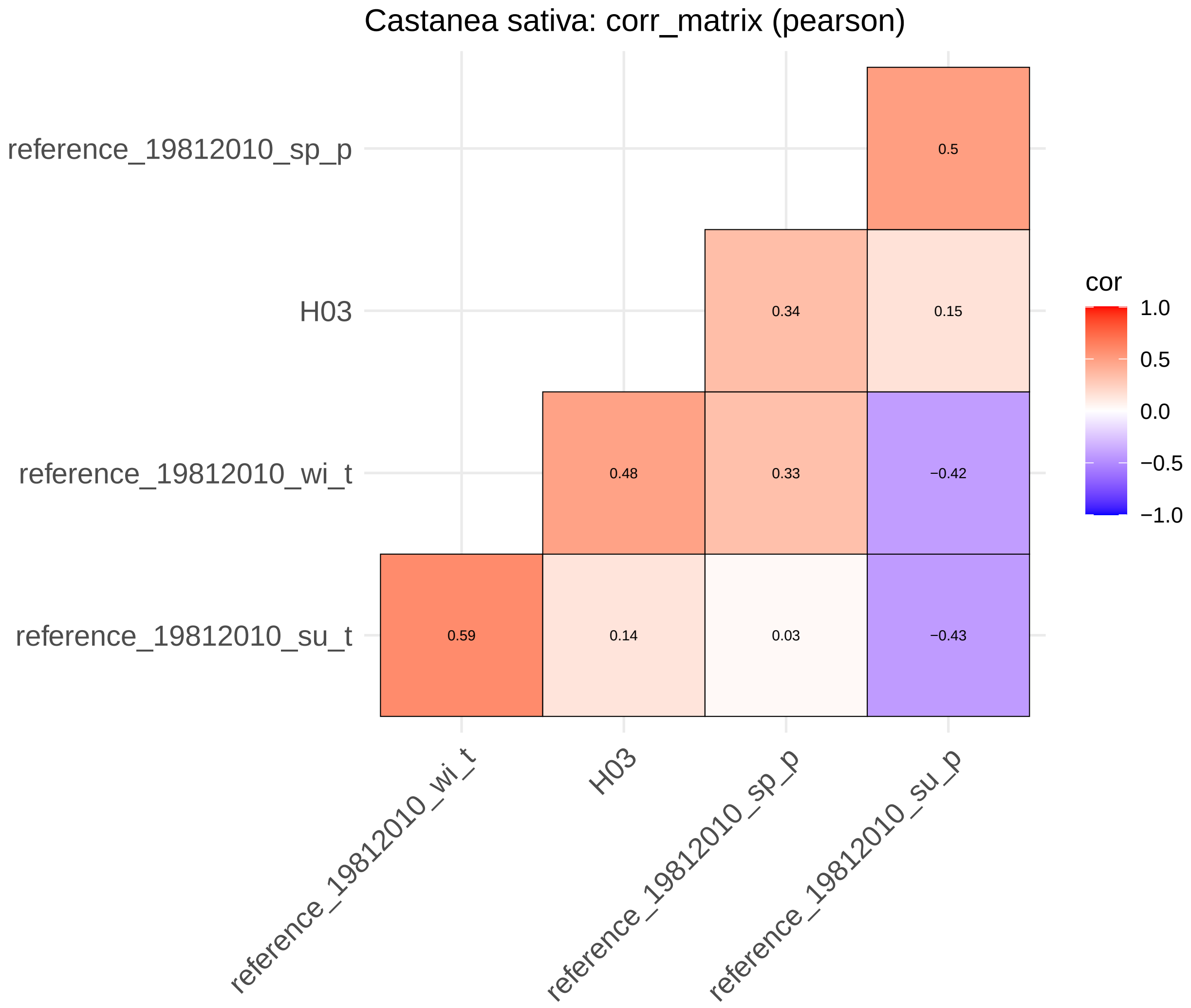

Response curves

Response curves (also known as effect curves) show how each predictor variable affects the target variable (H03 = european Site index, SIrel). H03 values below zero represent 'Growth absences'. Predictor acronyms: Bio.1 = Mean annual temperature [°C], Bio.12 = Annual precipitation sum [mm/m2], sp_p = Sum of precipitation [mm/m2] within months 3 to 5, su_p = Sum of precipitation [mm/m2] within months 6 to 8, wi_p = Sum of precipitation [mm/m2] within months 12,1,2, sp_t = Mean temperature [°C] within months 3 to 5, su_t = Mean temperature [°C] within months 6 to 8, wi_t = Mean temperature [°C] within months 12,1,2.

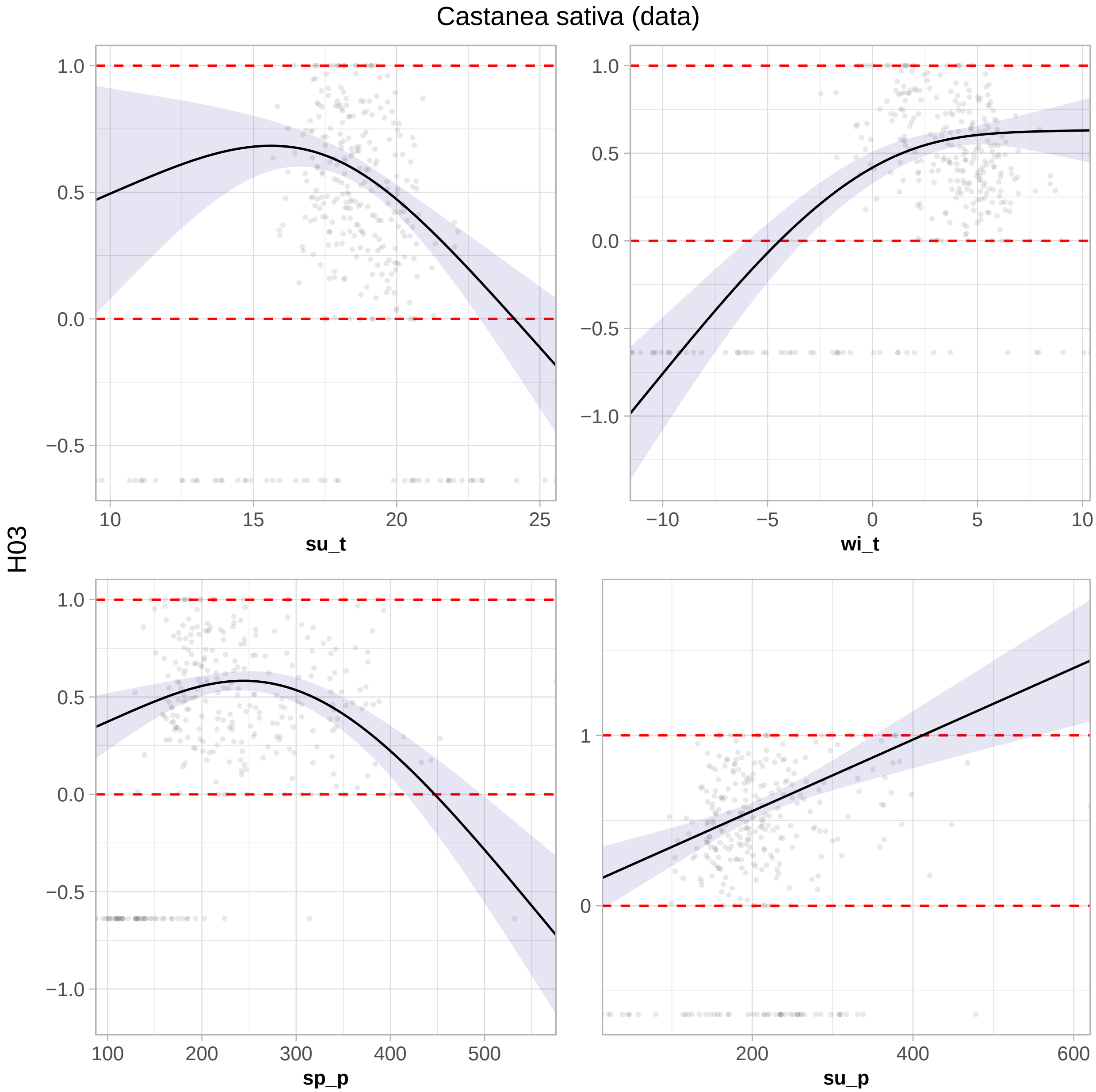

Response maps

Response maps (also referred as partial effect maps). Each map visualizes how a predictor affect the target variable (top height [m] at Age 100). Technically their work like response curves in a geographical area, that is setting all predictor variables except the one shown in the figure on their mean, and mapping the prediction. Predictor acronyms: Bio.1 = Mean annual temperature [°C], Bio.12 = Annual precipitation sum [mm/m2], sp_p = Sum of precipitation [mm/m2] within months 3 to 5, su_p = Sum of precipitation [mm/m2] within months 6 to 8, wi_p = Sum of precipitation [mm/m2] within months 12,1,2, sp_t = Mean temperature [°C] within months 3 to 5, su_t = Mean temperature [°C] within months 6 to 8, wi_t = Mean temperature [°C] within months 12,1,2.

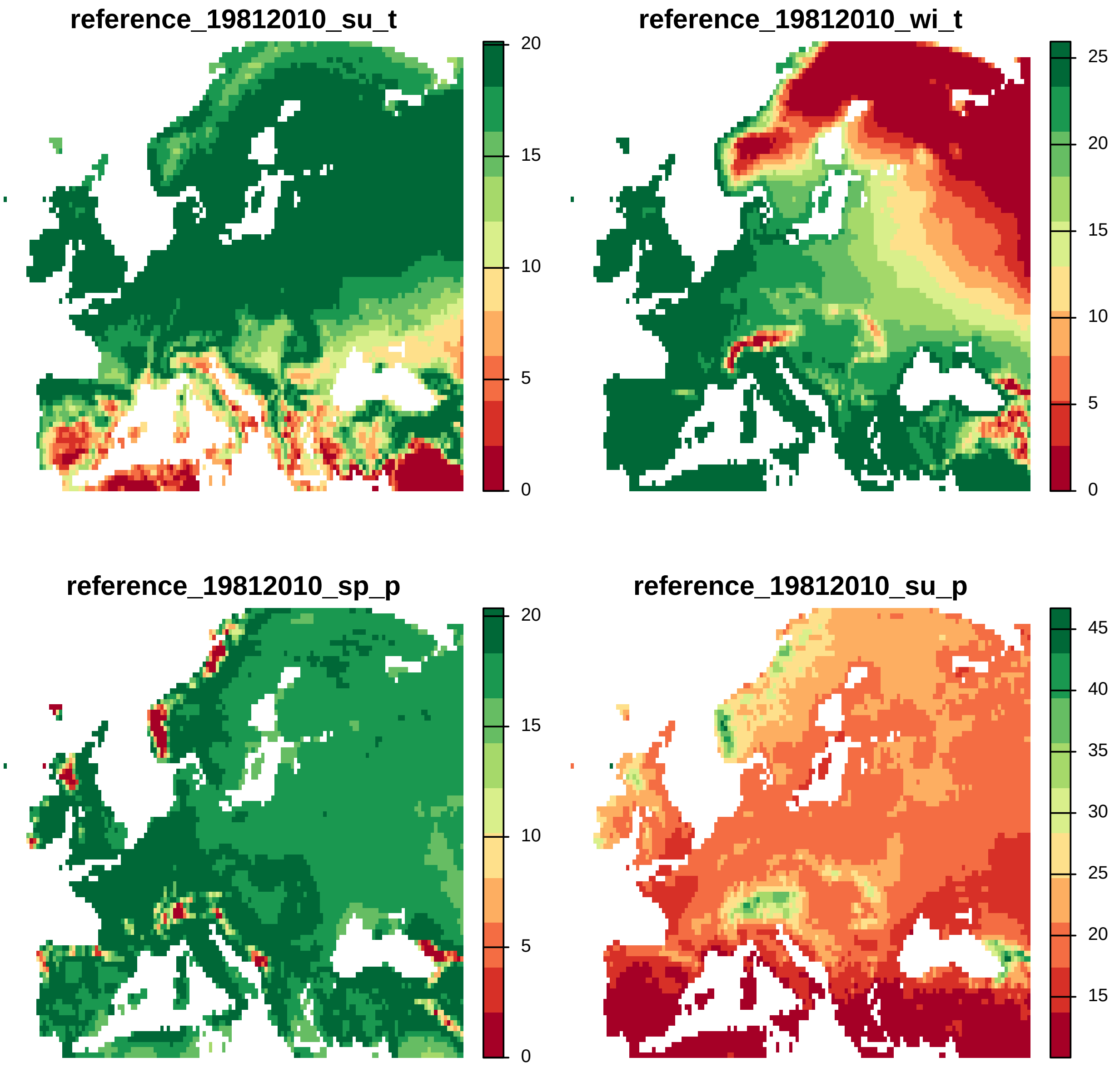

Residual distribution

The multi-panel plot includes a histogram of the residuals (top left), residuals over fitted values (top right), a histogram of observed and predicted values (bottom left) and boxplot diagram of observed and predicted values (bottom right). Observed values are shown in light green, while predicted ones are depicted in light red.

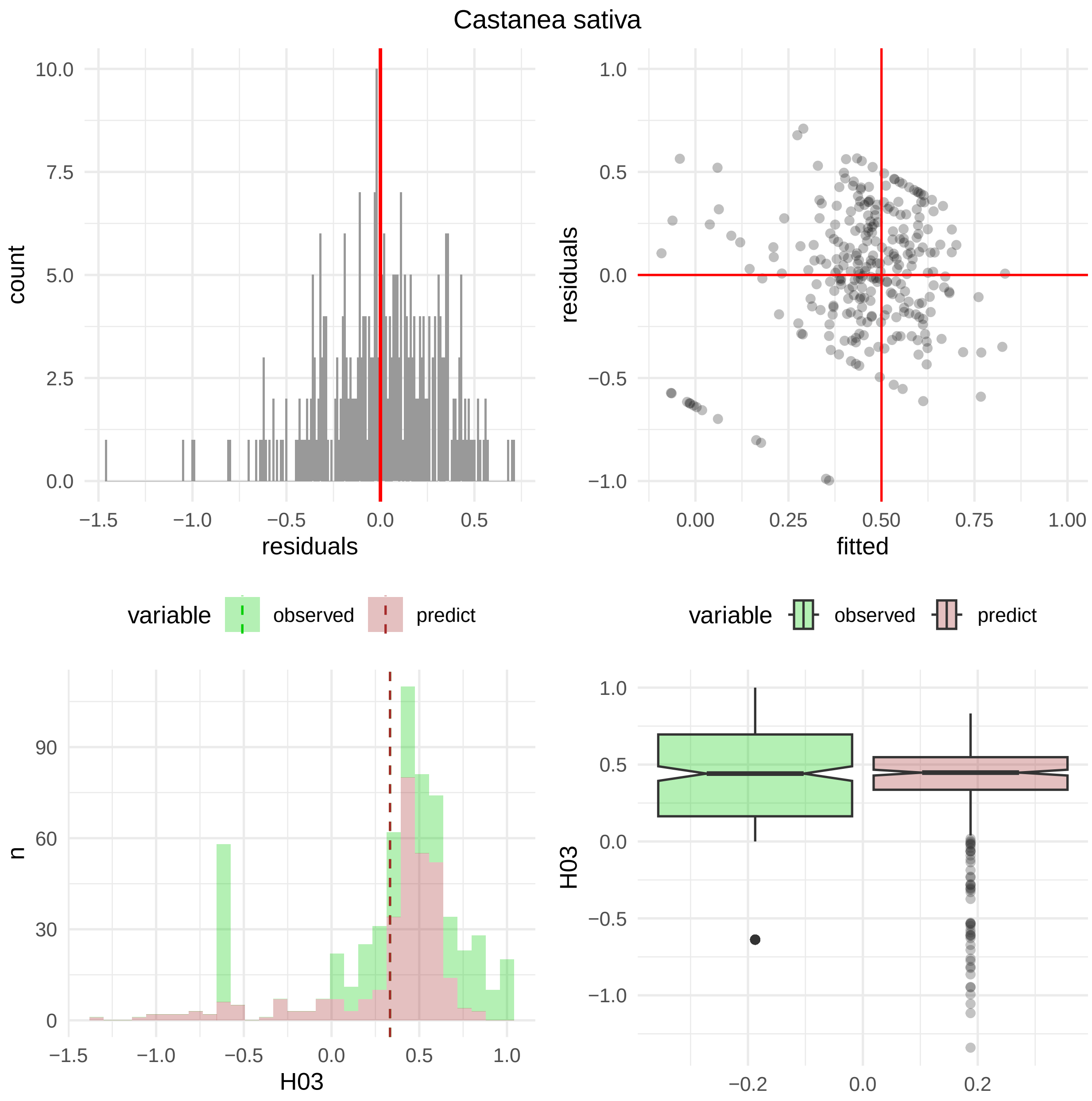

Correlation between predict and observed site index

Relationship between predicted and observed site index (density cloud), as well as linear regressions of presences and absences (= 'growth absences') (red line) and presences only (magenta line). The formulas, significance, R2 and number of observations are displayed below for both regressions. Ideally, both the point cloud and the regression lines lie close to the dashed line. For presences only we additionally calculated the correlation coefficient according to PEARSON (cor.pre) in the bottom right corner.

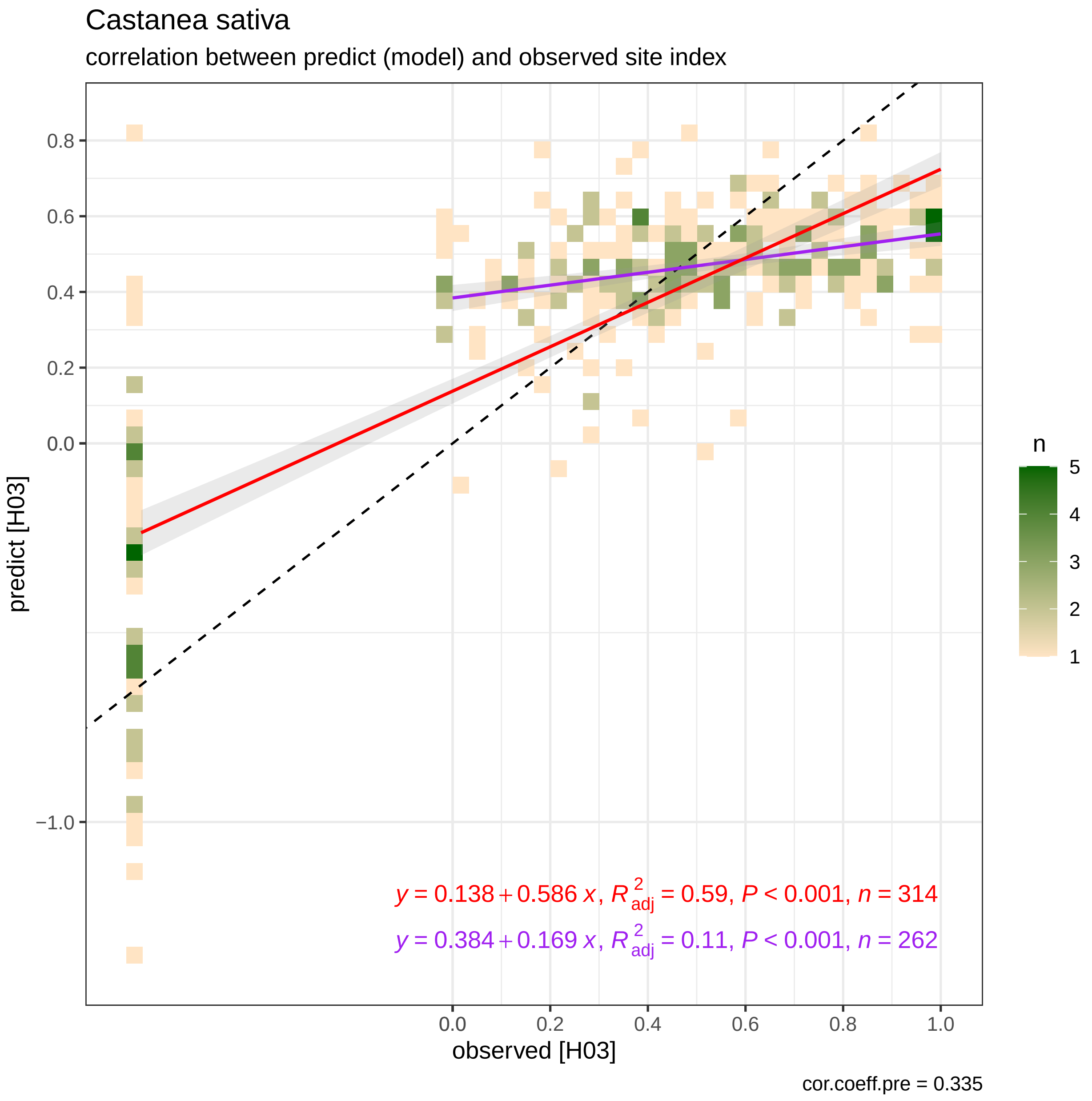

Predictions and forecasts

Predict

European predict for the reference period (1981 to 2010). Dark green symbolizes a high site index (tree height in meters at age 100), orange a lower site index and red no growth. Magenta-coloured dots represent inventory points with growth information, light blue dots are absences (= 'growth absences'). Results were aggregated on 25 km x 25 km scale.

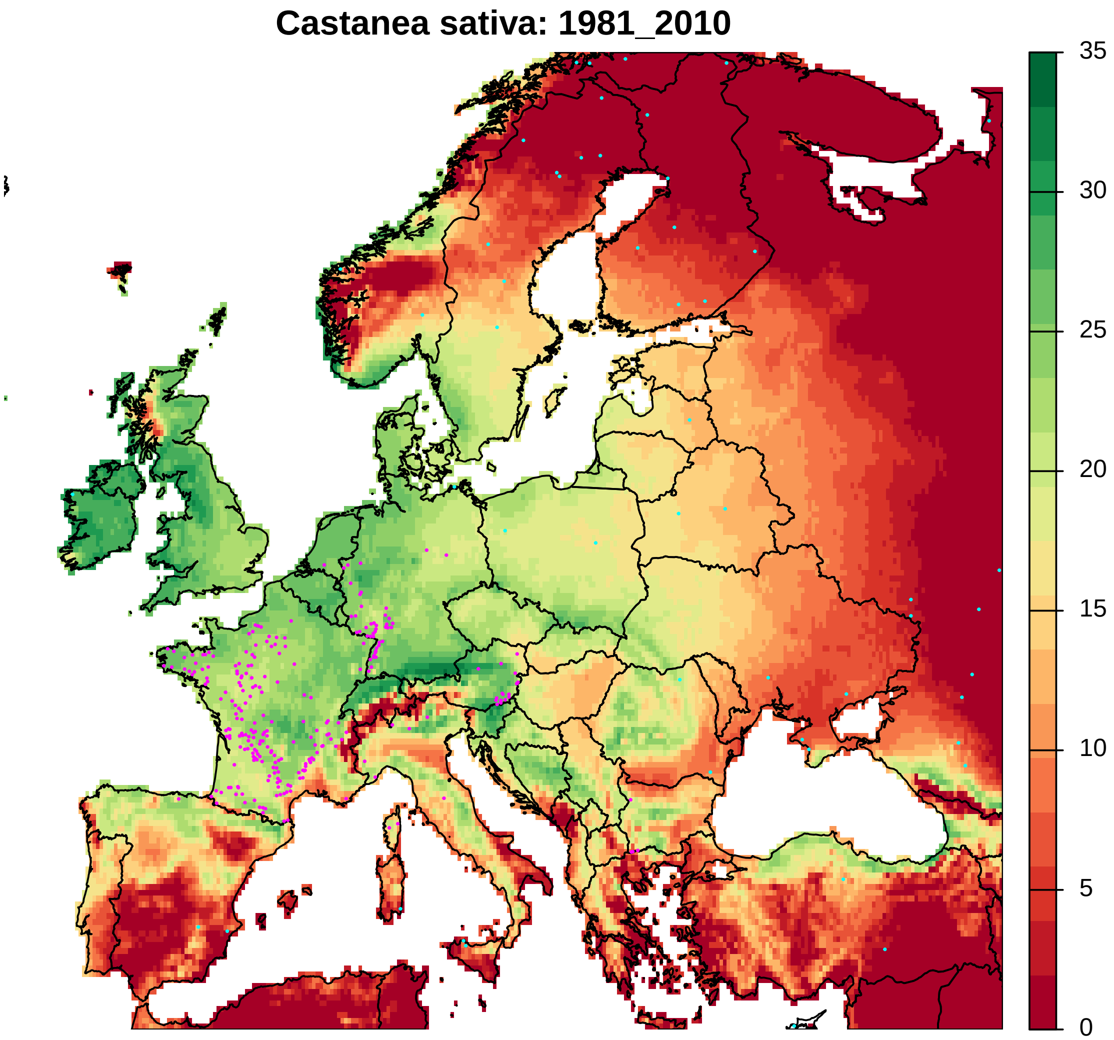

Forecast

Prediction for the reference period (1981 to 2010), as well as forecasts to 2071 to 2100 under szenario RCP4.5 and RCP8.5. Dark green symbolizes a high site index (tree height in m at age 100), orange a lower site index and red no growth. Results were aggregated on 25 km x 25 km scale.

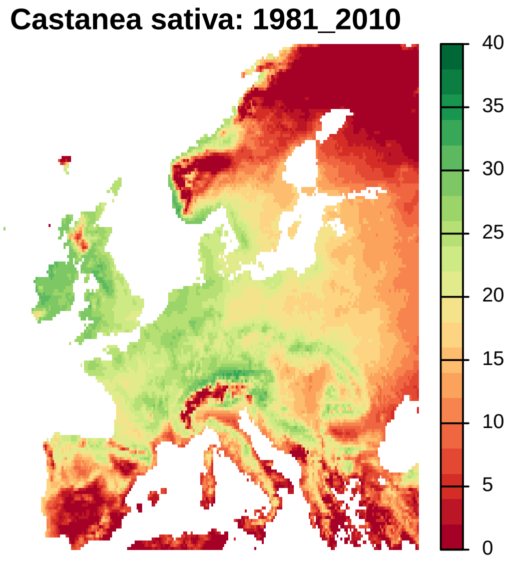

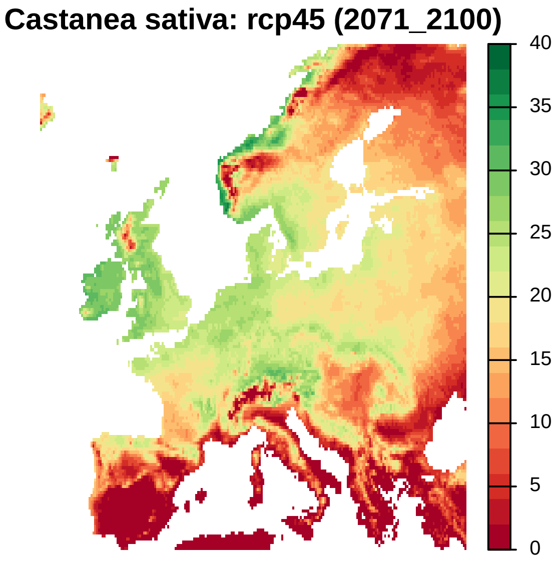

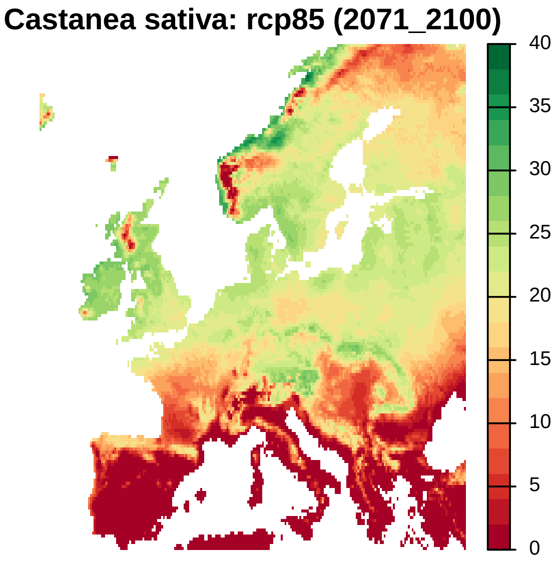

Fagus sylvatica

Site index curves

Site index curves of Fagus sylvatica created with non-linear quantile regressions based on the algorithm of Koenker and Park (1992). The site index (SI) was created by setting all points on the 95 percent quantile (upper line) and above to one (SI = 1) and all on the 5 percent quantile (lower line) and below to zero (SI = 0). The points between the quantile boundaries were assigned a site index between zero and one according to the ratio of their position between the quantile boundaries. We set selected absences (see chapter 2.1.3) on Height = 0 m (at age 100), which means, depending on the site index curves, for each tree species a SI near -1 (red line).

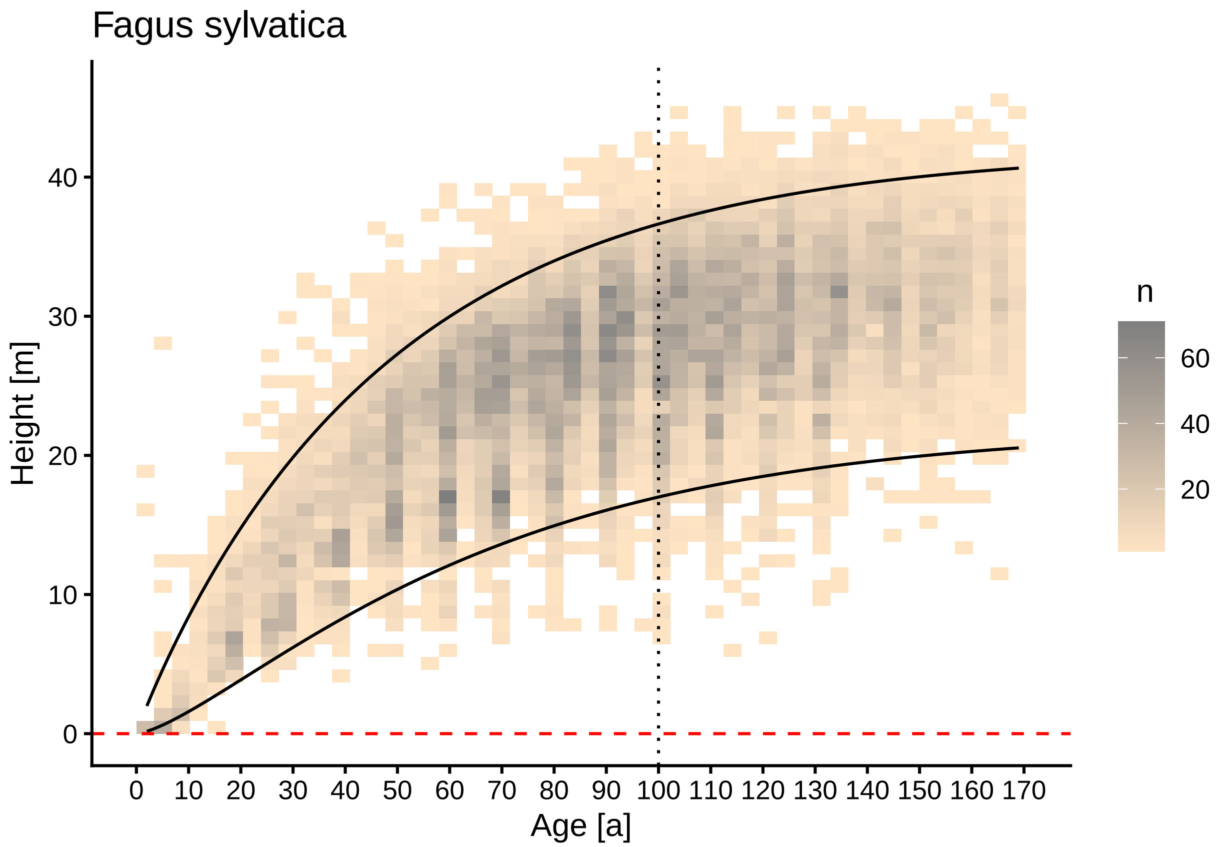

Model statistics and evaluation

Summary

Predictor acronyms: Bio.1 = Mean annual temperature [°C], Bio.12 = Annual precipitation sum [mm/m2], sp_p = Sum of precipitation [mm/m2] within months 3 to 5, su_p = Sum of precipitation [mm/m2] within months 6 to 8, wi_p = Sum of precipitation [mm/m2] within months 12,1,2, sp_t = Mean temperature [°C] within months 3 to 5, su_t = Mean temperature [°C] within months 6 to 8, wi_t = Mean temperature [°C] within months 12,1,2.

```
##
## Family: gaussian
## Link function: identity
##
## Formula:
## H03 ~ s(reference_19812010_su_t, k = 3) + s(reference_19812010_wi_t,
##     k = 3) + s(reference_19812010_sp_p, k = 3)
##
## Parametric coefficients:
##             Estimate Std. Error t value Pr(>|t|)
## (Intercept) 0.440664   0.008177   53.89   <2e-16 ***
## ---
## Signif. codes:  0 '***' 0.001 '**' 0.01 '*' 0.05 '.' 0.1 ' ' 1
##
## Approximate significance of smooth terms:
##                              edf Ref.df      F p-value
## s(reference_19812010_su_t) 1.997      2 388.96  <2e-16 ***
## s(reference_19812010_wi_t) 1.996      2 342.88  <2e-16 ***
## s(reference_19812010_sp_p) 1.992      2  68.72  <2e-16 ***
## ---
## Signif. codes:  0 '***' 0.001 '**' 0.01 '*' 0.05 '.' 0.1 ' ' 1
##
## R-sq.(adj) =  0.564   Deviance explained = 56.5%
## -REML = 1497.3  Scale est. = 0.17773   n = 2658
```

Variance inflation factor (VIF)

Predictor acronyms: Bio.1 = Mean annual temperature [°C], Bio.12 = Annual precipitation sum [mm/m2], sp_p = Sum of precipitation [mm/m2] within months 3 to 5, su_p = Sum of precipitation [mm/m2] within months 6 to 8, wi_p = Sum of precipitation [mm/m2] within months 12,1,2, sp_t = Mean temperature [°C] within months 3 to 5, su_t = Mean temperature [°C] within months 6 to 8, wi_t = Mean temperature [°C] within months 12,1,2.

```
##                   Variables      VIF
## 1 reference_19812010_su_t 1.597571
## 2 reference_19812010_wi_t 1.481182
## 3 reference_19812010_sp_p 1.191400
```

Correlation matrix

Correlation matrix between the predictor variables and the target variable in the model. Correlation coefficient according to PEARSON. Predictor acronyms: Bio.1 = Mean annual temperature [°C], Bio.12 = Annual precipitation sum [mm/m2], sp_p = Sum of precipitation [mm/m2] within months 3 to 5, su_p = Sum of precipitation [mm/m2] within months 6 to 8, wi_p = Sum of precipitation [mm/m2] within months 12,1,2, sp_t = Mean temperature [°C] within months 3 to 5, su_t = Mean temperature [°C] within months 6 to 8, wi_t = Mean temperature [°C] within months 12,1,2.

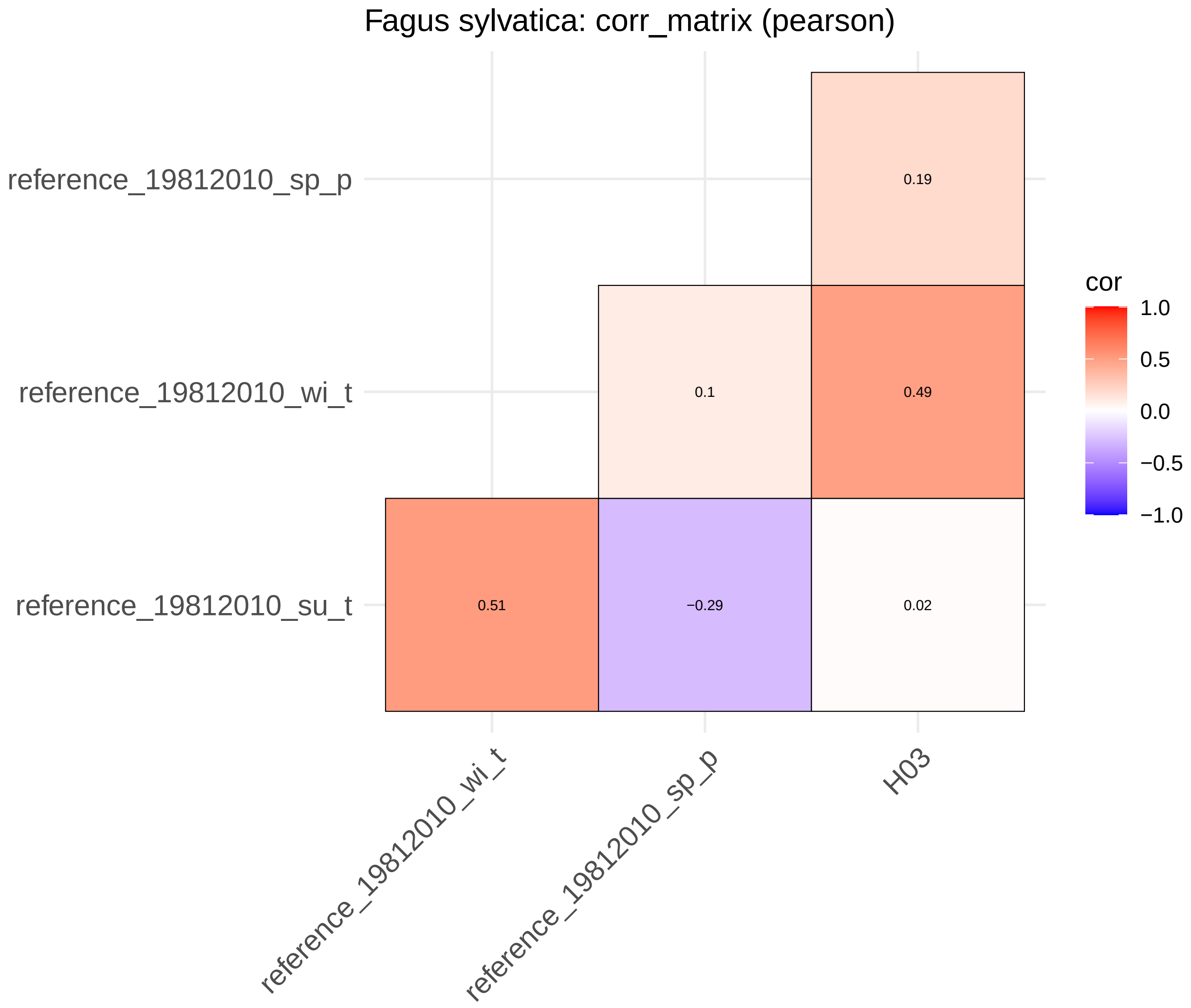

Response curves

Response curves (also known as effect curves) show how each predictor variable affects the target variable (H03 = european Site index, SIrel). H03 values below zero represent 'Growth absences'. Predictor acronyms: Bio.1 = Mean annual temperature [°C], Bio.12 = Annual precipitation sum [mm/m2], sp__p = Sum of precipitation [mm/m2] within months 3 to 5, su__p = Sum of precipitation [mm/m2] within months 6 to 8, wi__p = Sum of precipitation [mm/m2] within months 12,1,2, sp__t = Mean temperature [°C] within months 3 to 5, su__t = Mean temperature [°C] within months 6 to 8, wi__t = Mean temperature [°C] within months 12,1,2.

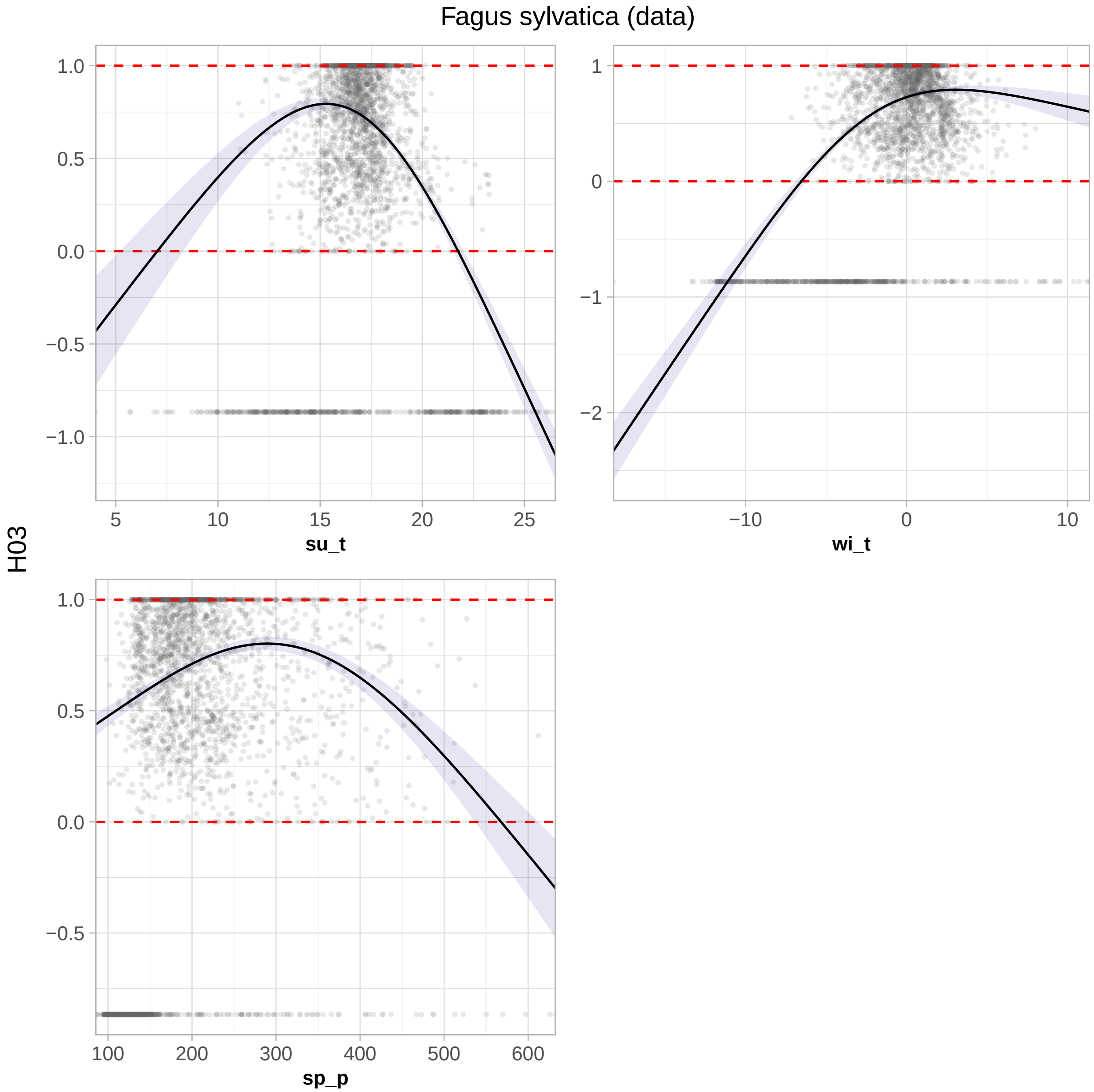

Response maps

Response maps (also referred as partial effect maps). Each map visualizes how a predictor affect the target variable (top height [m] at Age 100). Technically their work like response curves in a geographical area, that is setting all predictor variables except the one shown in the figure on their mean, and mapping the prediction. Predictor acronyms: Bio.1 = Mean annual temperature [°C], Bio.12 = Annual precipitation sum [mm/m2], sp_p = Sum of precipitation [mm/m2] within months 3 to 5, su_p = Sum of precipitation [mm/m2] within months 6 to 8, wi_p = Sum of precipitation [mm/m2] within months 12,1,2, sp_t = Mean temperature [°C] within months 3 to 5, su_t = Mean temperature [°C] within months 6 to 8, wi_t = Mean temperature [°C] within months 12,1,2.

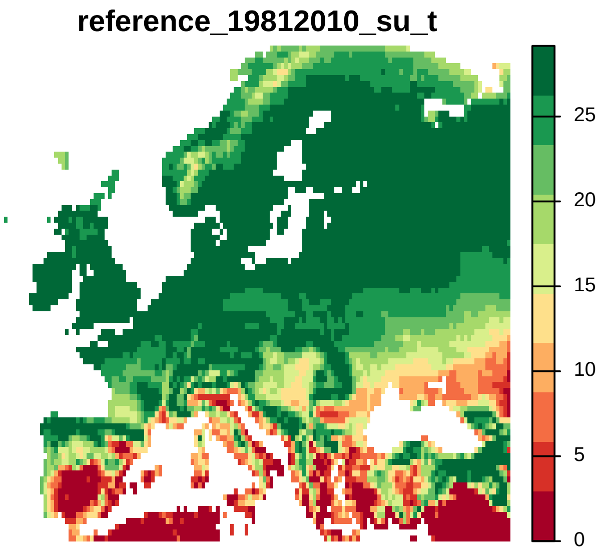

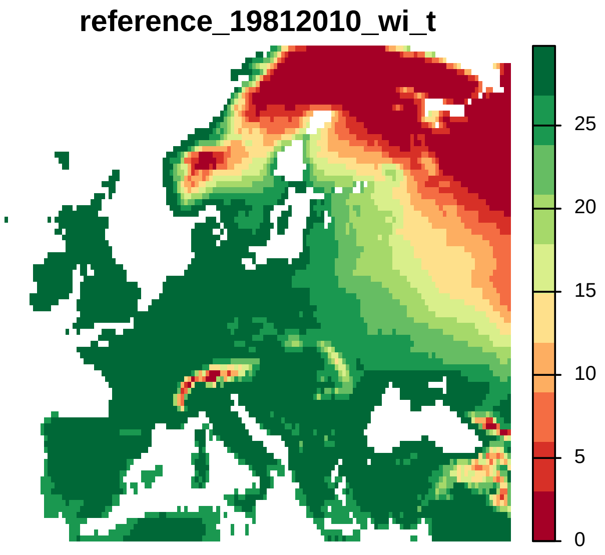

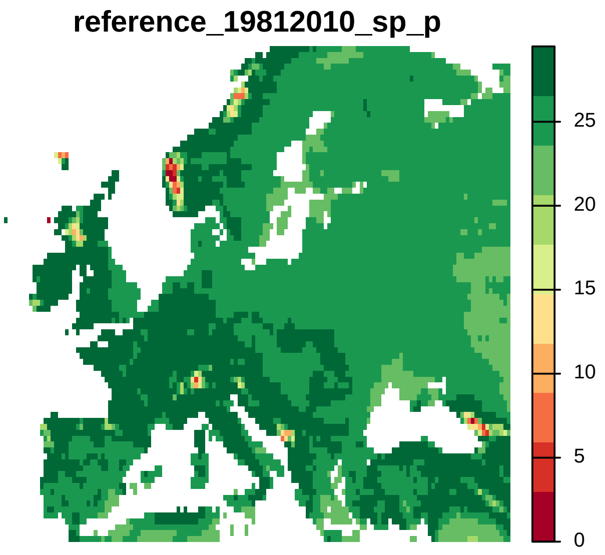

Residual distribution

The multi-panel plot includes a histogram of the residuals (top left), residuals over fitted values (top right), a histogram of observed and predicted values (bottom left) and boxplot diagram of observed and predicted values (bottom right). Observed values are shown in light green, while predicted ones are depicted in light red.

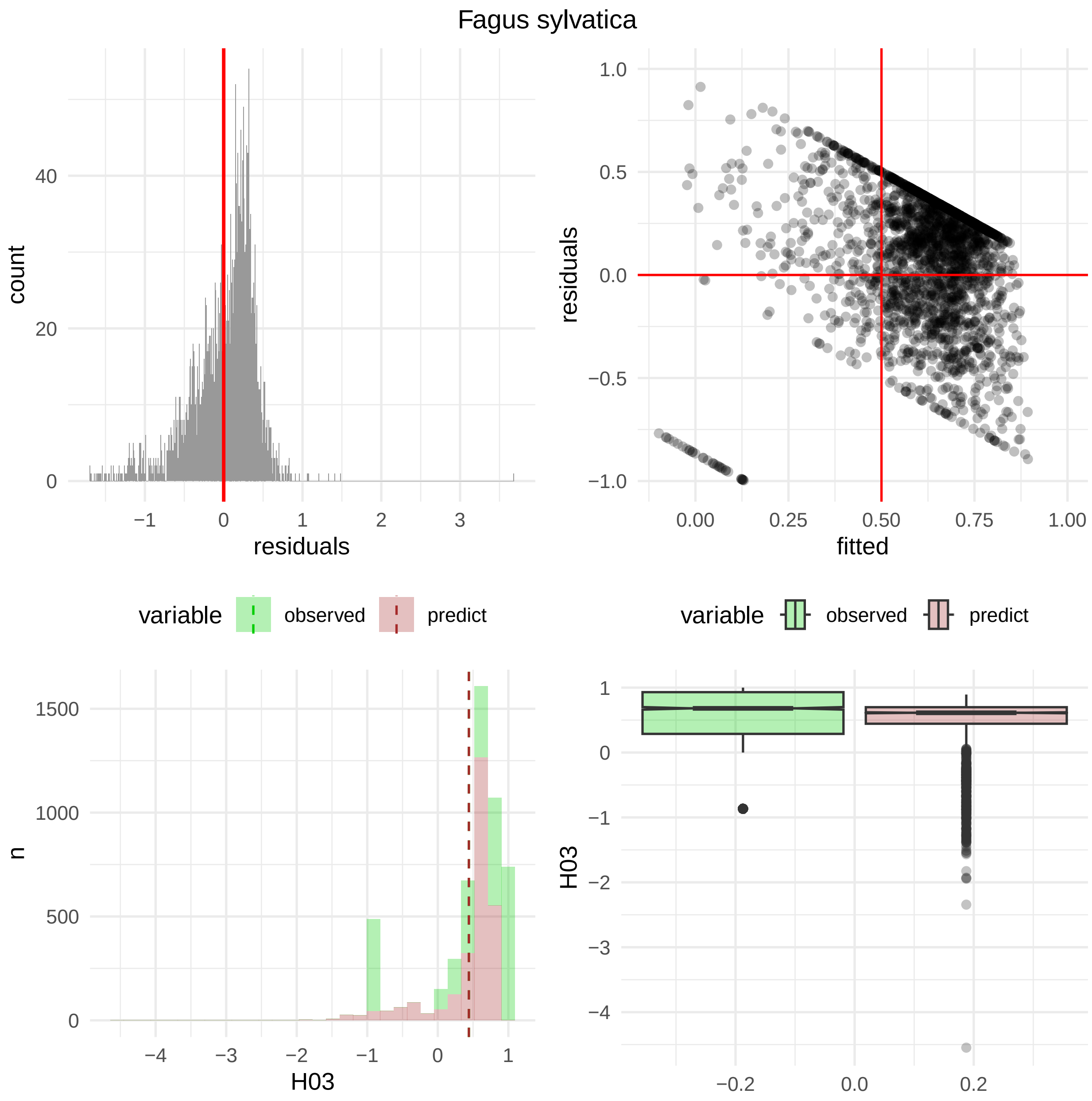

Correlation between predict and observed site index

Relationship between predicted and observed site index (density cloud), as well as linear regressions of presences and absences (= 'growth absences') (red line) and presences only (magenta line). The formulas, significance, R2 and number of observations are displayed below for both regressions. Ideally, both the point cloud and the regression lines lie close to the dashed line. For presences only we additionally calculated the correlation coefficient according to PEARSON (cor.pre) in the bottom right corner.

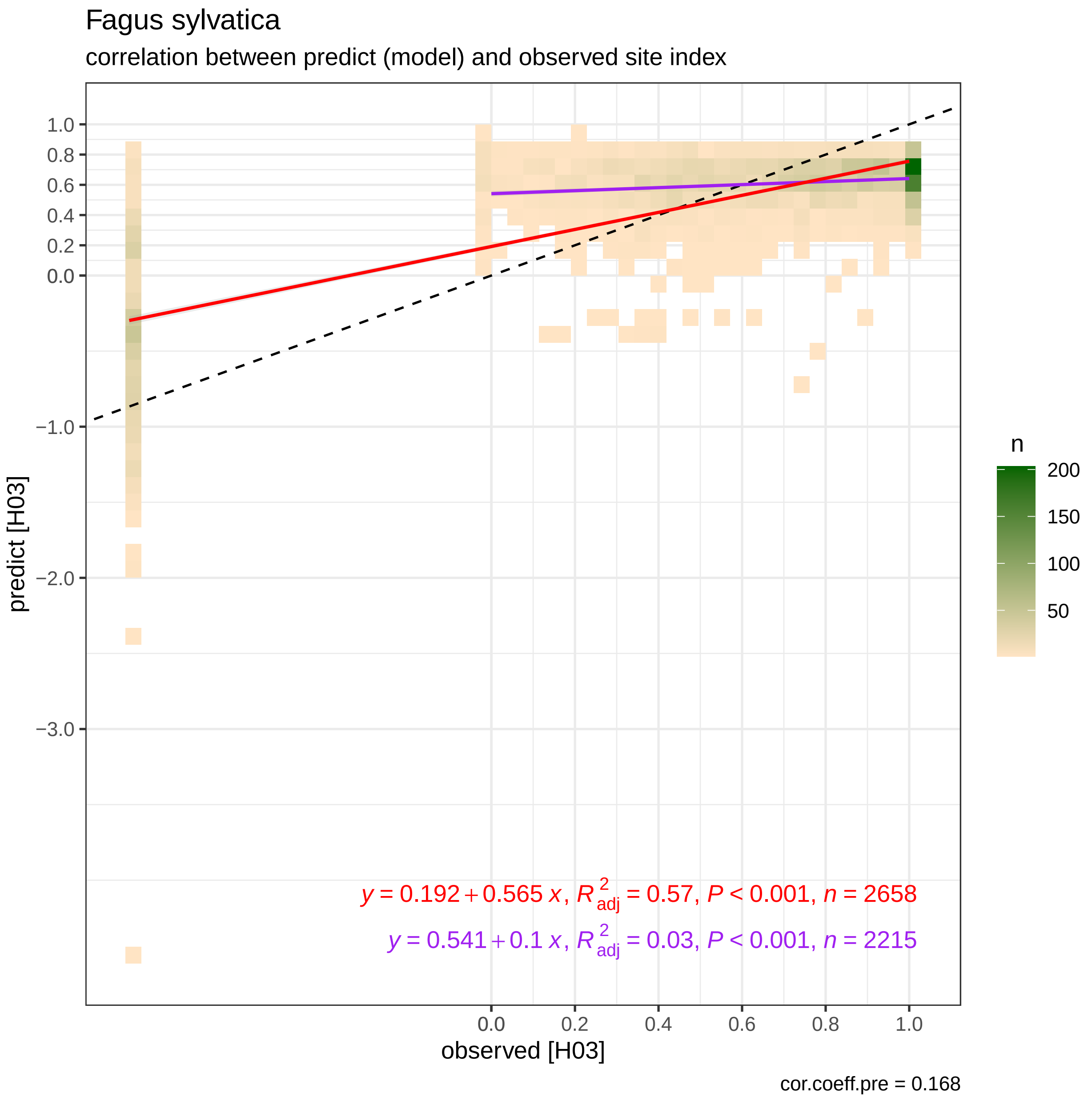

Predictions and forecasts

Predict

European predict for the reference period (1981 to 2010). Dark green symbolizes a high site index (tree height in meters at age 100), orange a lower site index and red no growth. Magenta-coloured dots represent inventory points with growth information, light blue dots are absences (= 'growth absences'). Results were aggregated on 25 km x 25 km scale.

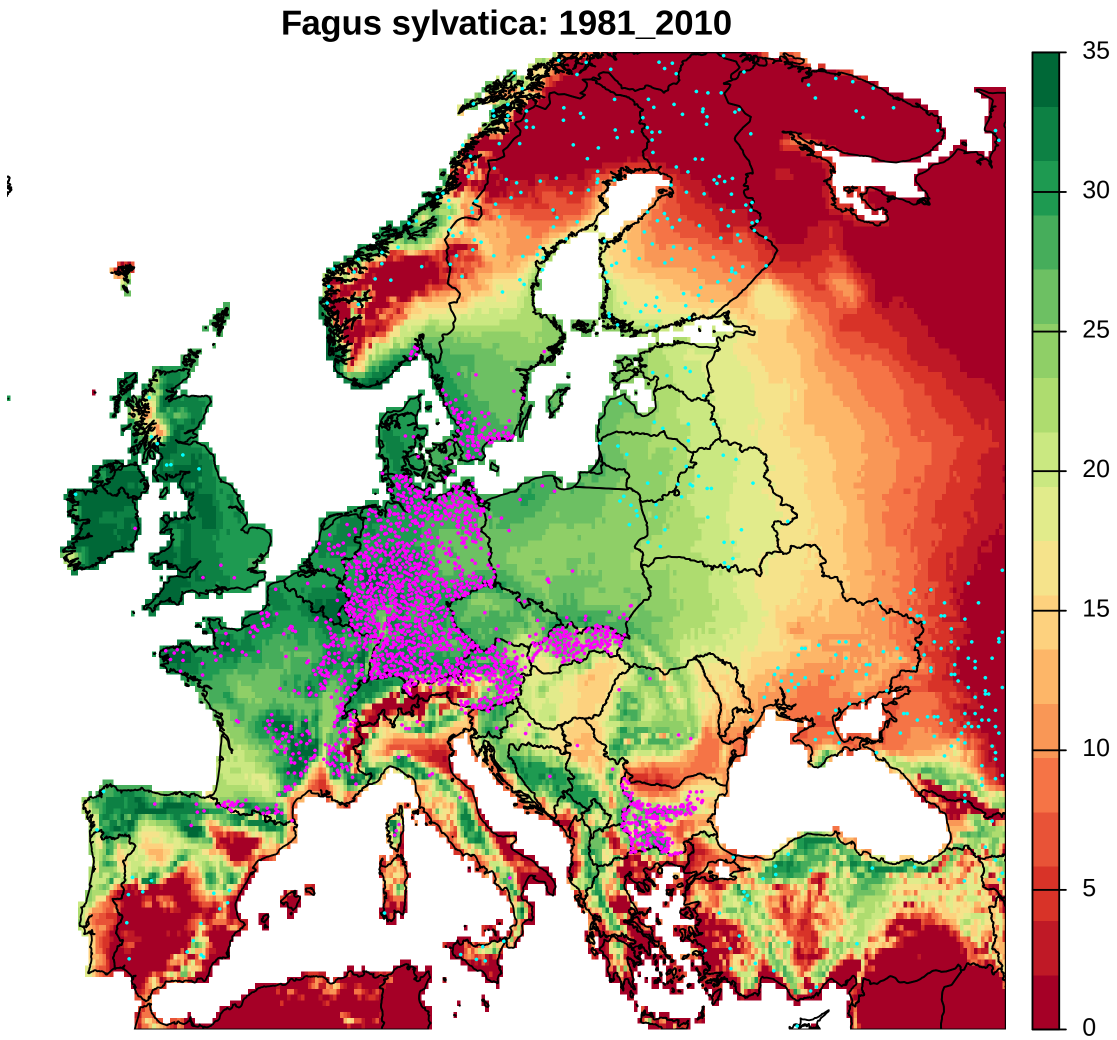

Forecast

Prediction for the reference period (1981 to 2010), as well as forecasts to 2071 to 2100 under szenario RCP4.5 and RCP8.5. Dark green symbolizes a high site index (tree height in m at age 100), orange a lower site index and red no growth. Results were aggregated on 25 km x 25 km scale.

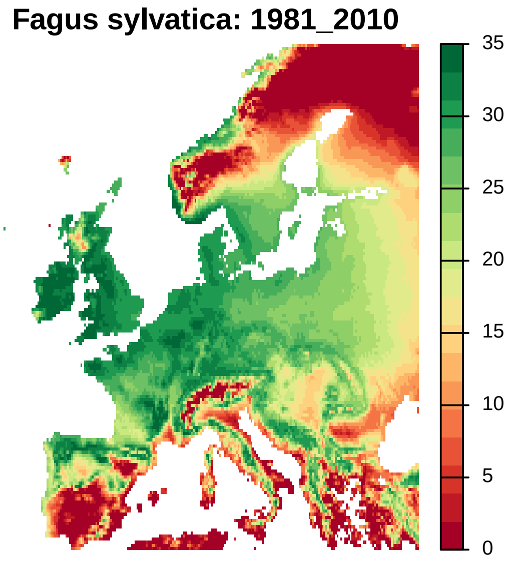

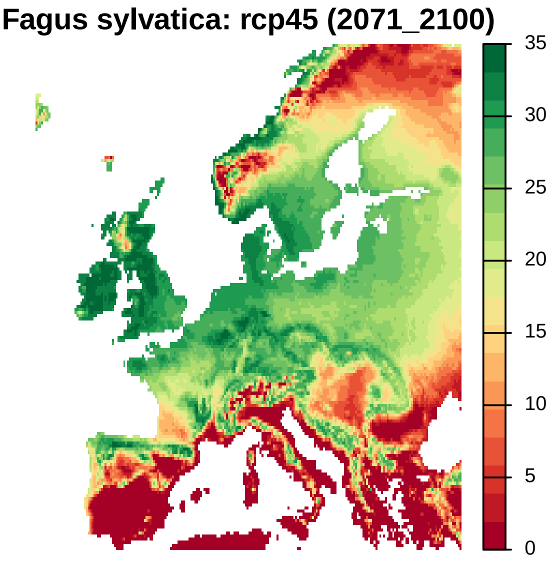

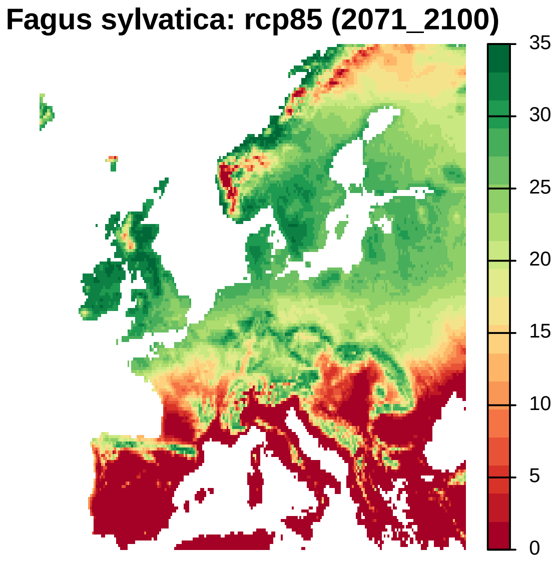

Fraxinus excelsior

Site index curves

Site index curves of Fraxinus excelsior created with non-linear quantile regressions based on the algorithm of Koenker and Park (1992). The site index (SI) was created by setting all points on the 95 percent quantile (upper line) and above to one (SI = 1) and all on the 5 percent quantile (lower line) and below to zero (SI = 0). The points between the quantile boundaries were assigned a site index between zero and one according to the ratio of their position between the quantile boundaries. We set selected absences (see chapter 2.1.3) on Height = 0 m (at age 100), which means, depending on the site index curves, for each tree species a SI near -1 (red line).

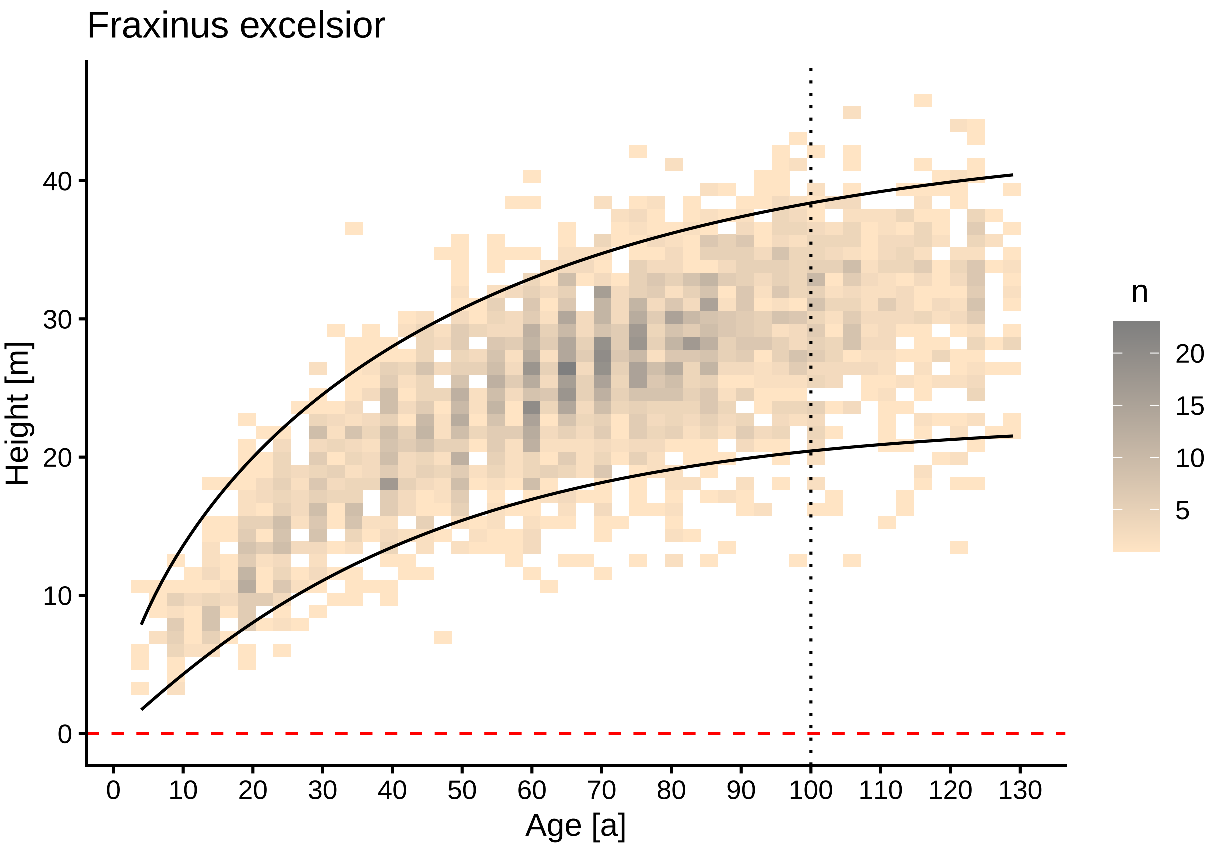

Model statistics and evaluation

Summary

Predictor acronyms: Bio.1 = Mean annual temperature [°C], Bio.12 = Annual precipitation sum [mm/m2], sp_p = Sum of precipitation [mm/m2] within months 3 to 5, su_p = Sum of precipitation [mm/m2] within months 6 to 8, wi_p = Sum of precipitation [mm/m2] within months 12,1,2, sp_t = Mean temperature [°C] within months 3 to 5, su_t = Mean temperature [°C] within months 6 to 8, wi_t = Mean temperature [°C] within months 12,1,2.

```
##
## Family: gaussian
## Link function: identity
##
## Formula:
## H03 ~ s(reference_19812010_wi_t, k = 3) + s(reference_19812010_su_t,
##     k = 3) + s(reference_19812010_sp_p, k = 3)
##
## Parametric coefficients:
##             Estimate Std. Error t value Pr(>|t|)
## (Intercept)  0.29219    0.01023   28.56   <2e-16 ***
## ---
## Signif. codes:  0 '***' 0.001 '**' 0.01 '*' 0.05 '.' 0.1 ' ' 1
##
## Approximate significance of smooth terms:
##                              edf Ref.df      F p-value
## s(reference_19812010_wi_t) 1.997      2 293.55  <2e-16 ***
## s(reference_19812010_su_t) 1.995      2 248.38  <2e-16 ***
## s(reference_19812010_sp_p) 1.985      2  34.68  <2e-16 ***
## ---
## Signif. codes:  0 '***' 0.001 '**' 0.01 '*' 0.05 '.' 0.1 ' ' 1
##
## R-sq.(adj) =  0.689   Deviance explained =   69%
## -REML = 685.73  Scale est. = 0.14908   n = 1424
```

Variance inflation factor (VIF)

Predictor acronyms: Bio.1 = Mean annual temperature [°C], Bio.12 = Annual precipitation sum [mm/m2], sp_p = Sum of precipitation [mm/m2] within months 3 to 5, su_p = Sum of precipitation [mm/m2] within months 6 to 8, wi_p = Sum of precipitation [mm/m2] within months 12,1,2, sp_t = Mean temperature [°C] within months 3 to 5, su_t = Mean temperature [°C] within months 6 to 8, wi_t = Mean temperature [°C] within months 12,1,2.

```
##                   Variables      VIF
## 1 reference_19812010_wi_t 1.909419
## 2 reference_19812010_su_t 1.894527
## 3 reference_19812010_sp_p 1.133014
```

Correlation matrix

Correlation matrix between the predictor variables and the target variable in the model. Correlation coefficient according to PEARSON. Predictor acronyms: Bio.1 = Mean annual temperature [°C], Bio.12 = Annual precipitation sum [mm/m2], sp_p = Sum of precipitation [mm/m2] within months 3 to 5, su_p = Sum of precipitation [mm/m2] within months 6 to 8, wi_p = Sum of precipitation [mm/m2] within months 12,1,2, sp_t = Mean temperature [°C] within months 3 to 5, su_t = Mean temperature [°C] within months 6 to 8, wi_t = Mean temperature [°C] within months 12,1,2.

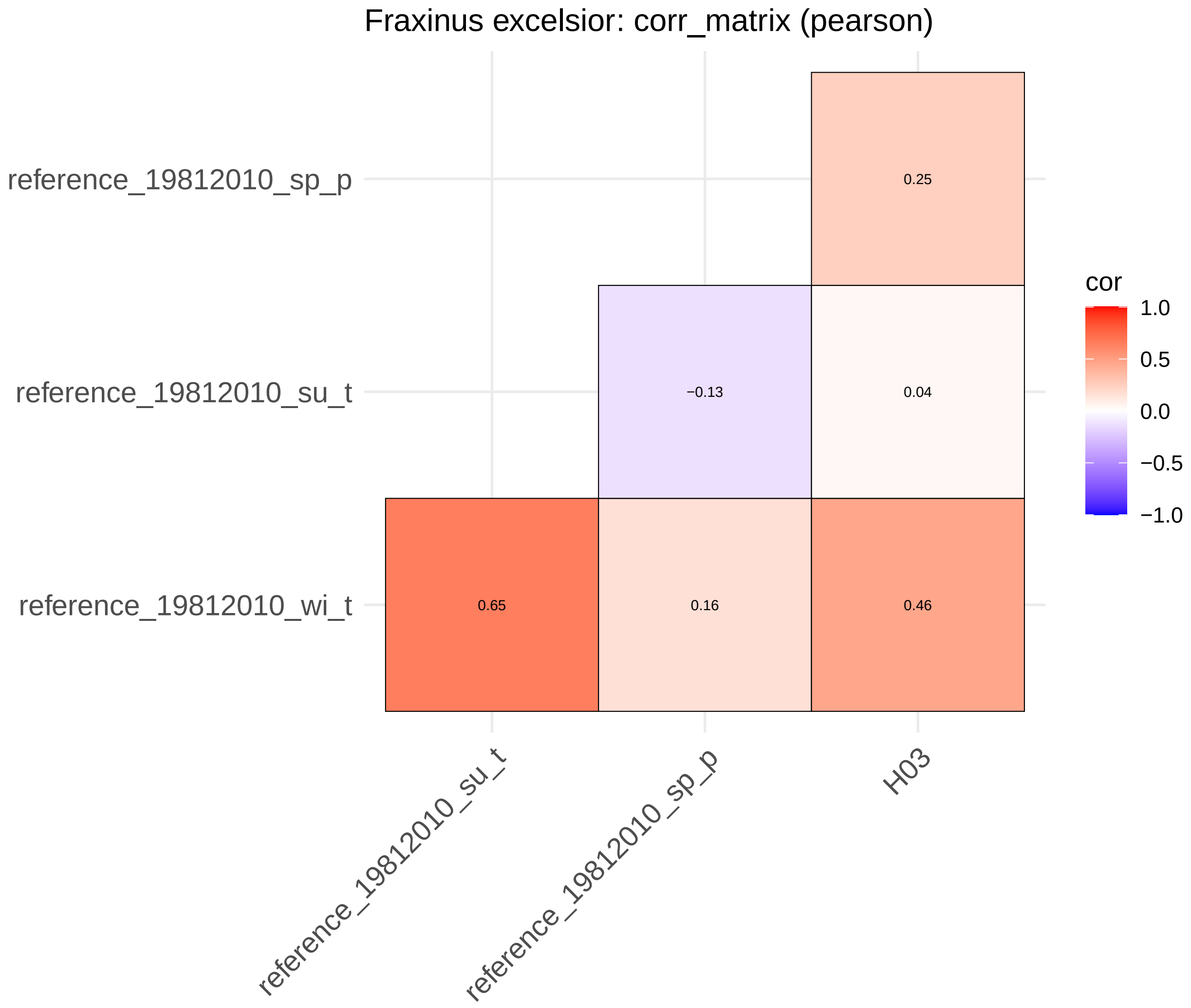

Response curves

Response curves (also known as effect curves) show how each predictor variable affects the target variable (H03 = european Site index, SIrel). H03 values below zero represent 'Growth absences'. Predictor acronyms: Bio.1 = Mean annual temperature [°C], Bio.12 = Annual precipitation sum [mm/m2], sp_p = Sum of precipitation [mm/m2] within months 3 to 5, su_p = Sum of precipitation [mm/m2] within months 6 to 8, wi_p = Sum of precipitation [mm/m2] within months 12,1,2, sp_t = Mean temperature [°C] within months 3 to 5, su_t = Mean temperature [°C] within months 6 to 8, wi_t = Mean temperature [°C] within months 12,1,2.

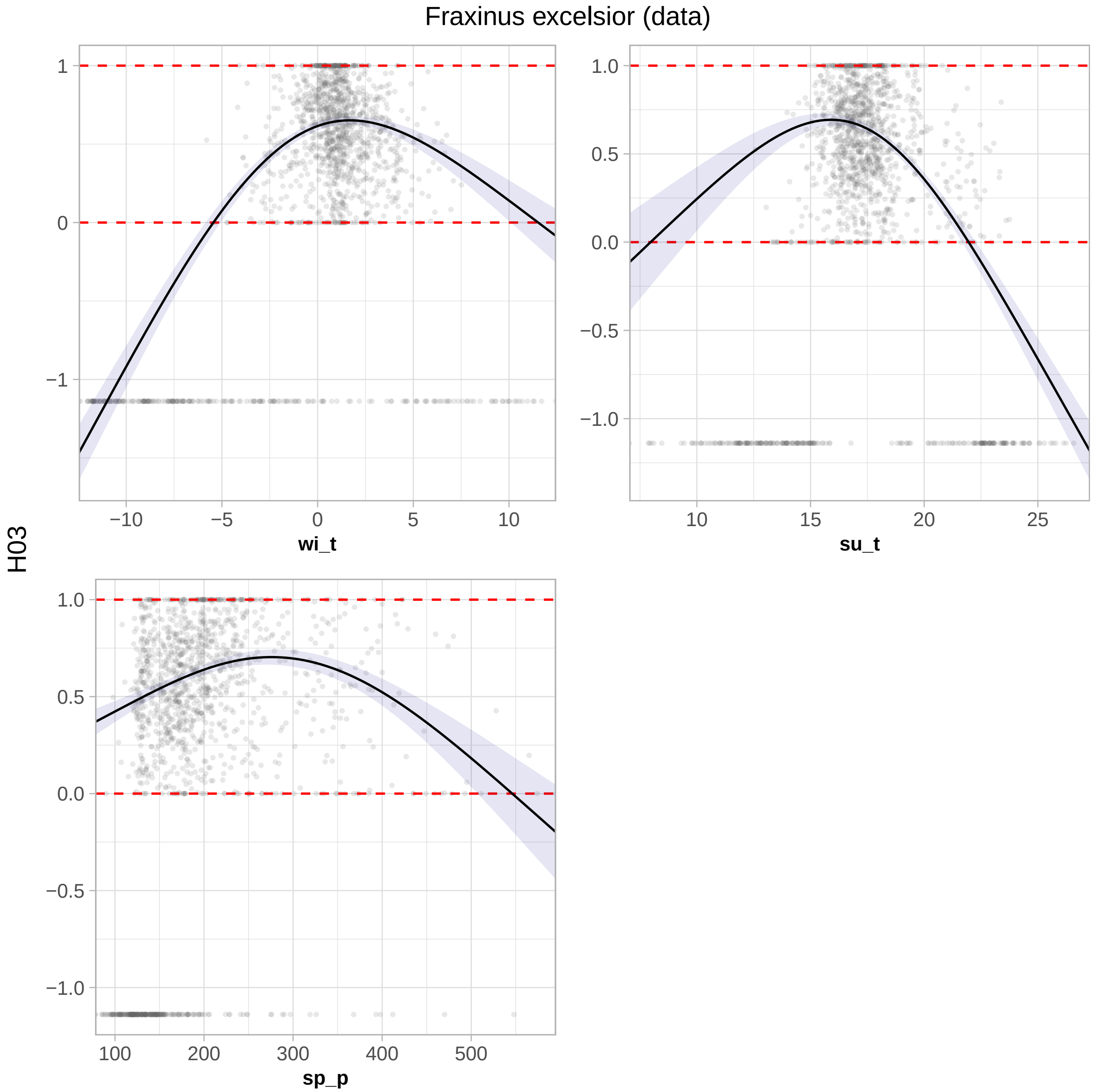

Response maps

Response maps (also referred as partial effect maps). Each map visualizes how a predictor affect the target variable (top height [m] at Age 100). Technically their work like response curves in a geographical area, that is setting all predictor variables except the one shown in the figure on their mean, and mapping the prediction. Predictor acronyms: Bio.1 = Mean annual temperature [°C], Bio.12 = Annual precipitation sum [mm/m2], sp_p = Sum of precipitation [mm/m2] within months 3 to 5, su_p = Sum of precipitation [mm/m2] within months 6 to 8, wi_p = Sum of precipitation [mm/m2] within months 12,1,2, sp_t = Mean temperature [°C] within months 3 to 5, su_t = Mean temperature [°C] within months 6 to 8, wi_t = Mean temperature [°C] within months 12,1,2.

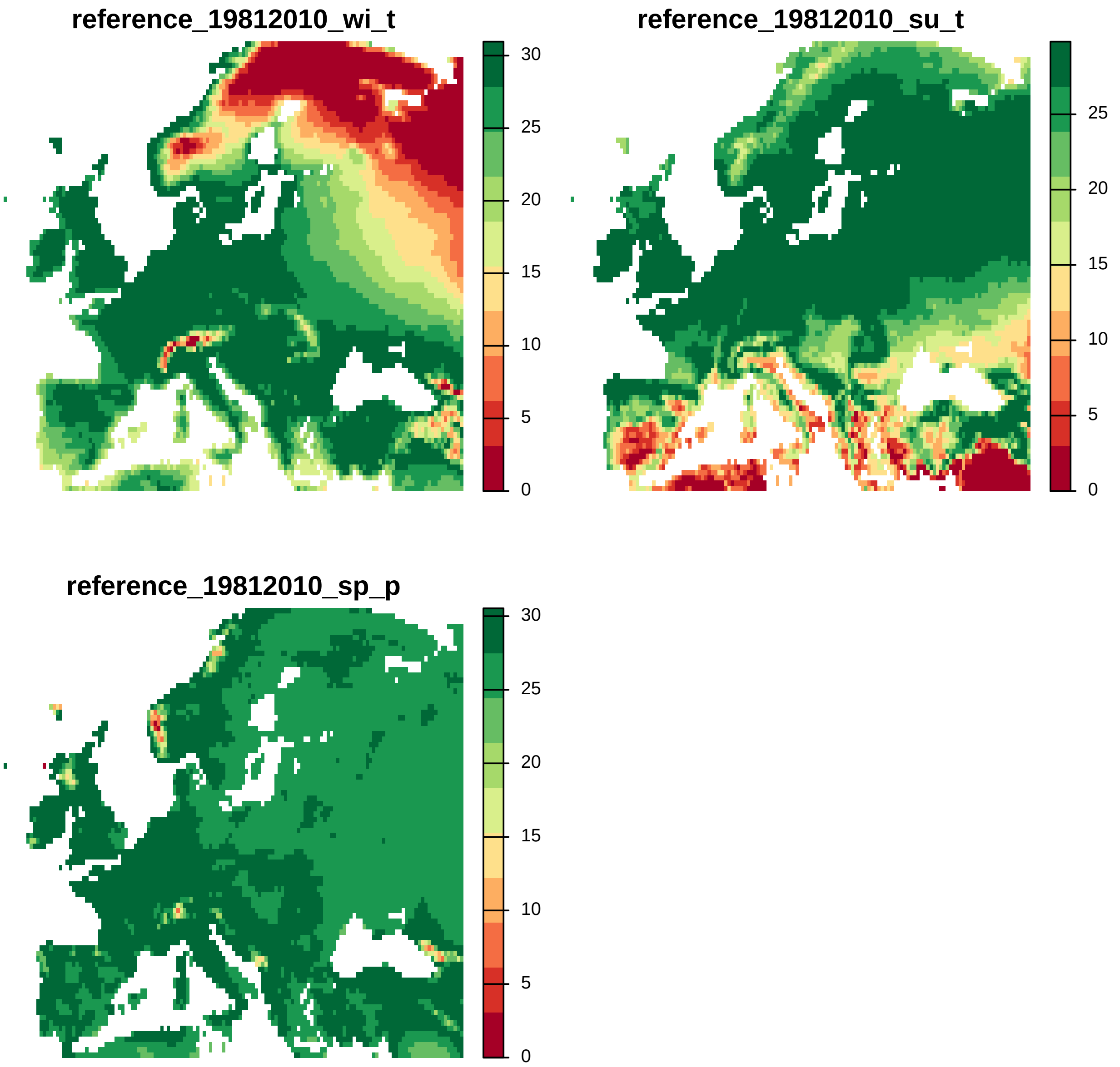

Residual distribution

The multi-panel plot includes a histogram of the residuals (top left), residuals over fitted values (top right), a histogram of observed and predicted values (bottom left) and boxplot diagram of observed and predicted values (bottom right). Observed values are shown in light green, while predicted ones are depicted in light red.

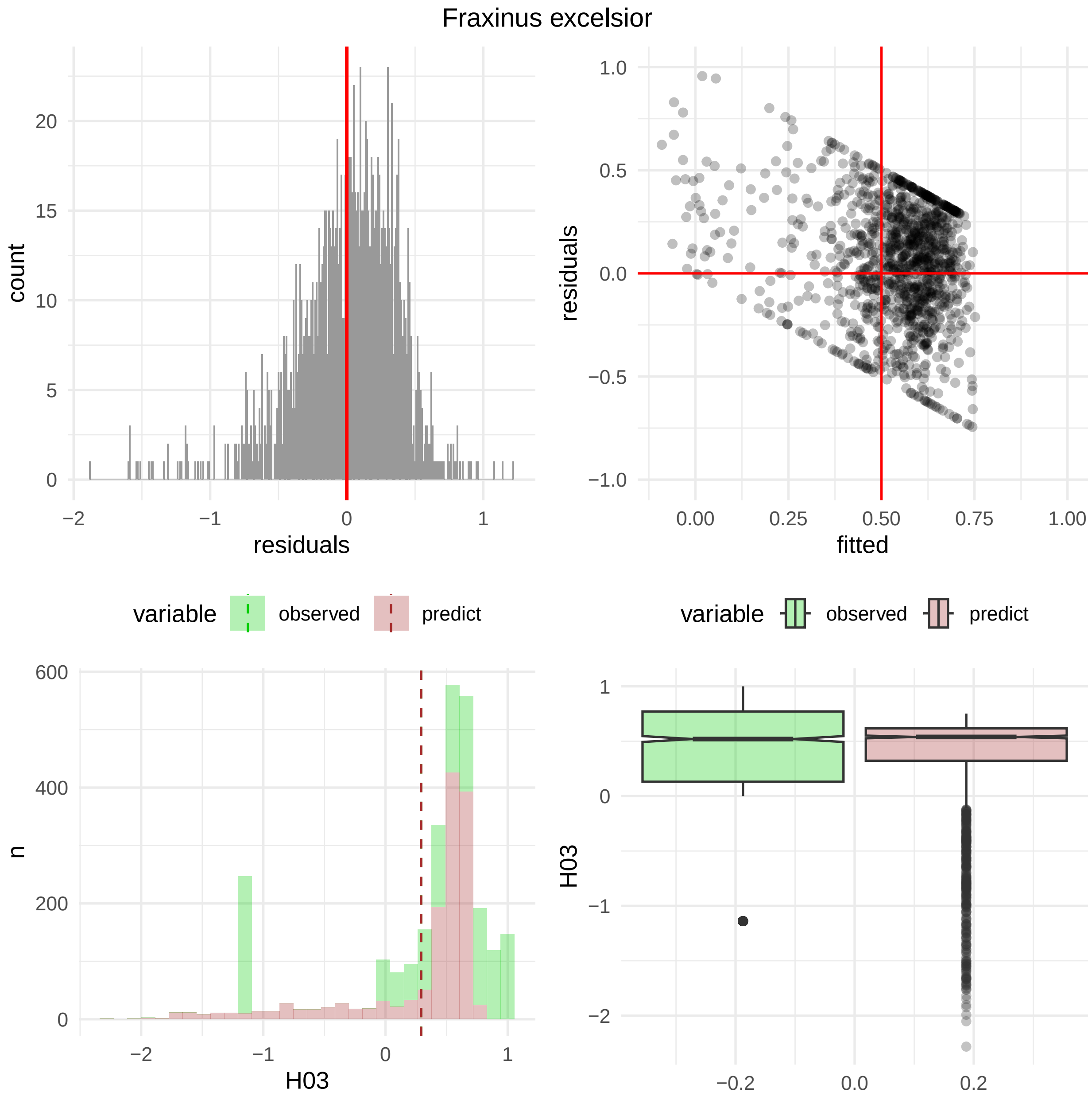

Correlation between predict and observed site index

Relationship between predicted and observed site index (density cloud), as well as linear regressions of presences and absences (= 'growth absences') (red line) and presences only (magenta line). The formulas, significance, R2 and number of observations are displayed below for both regressions. Ideally, both the point cloud and the regression lines lie close to the dashed line. For presences only we additionally calculated the correlation coefficient according to PEARSON (cor.pre) in the bottom right corner.

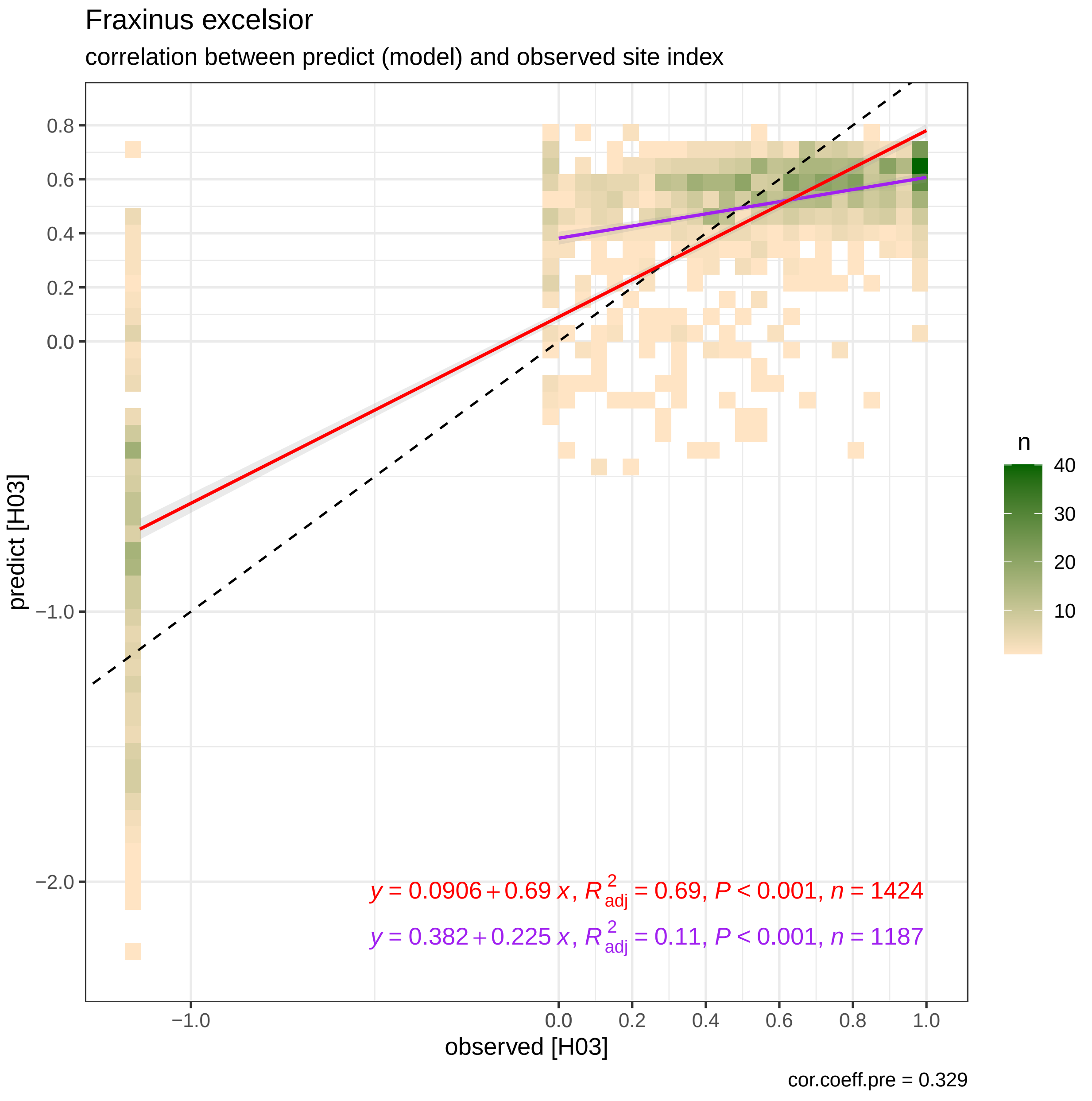

Predictions and forecasts

Predict

European predict for the reference period (1981 to 2010). Dark green symbolizes a high site index (tree height in meters at age 100), orange a lower site index and red no growth. Magenta-coloured dots represent inventory points with growth information, light blue dots are absences (= 'growth absences'). Results were aggregated on 25 km x 25 km scale.

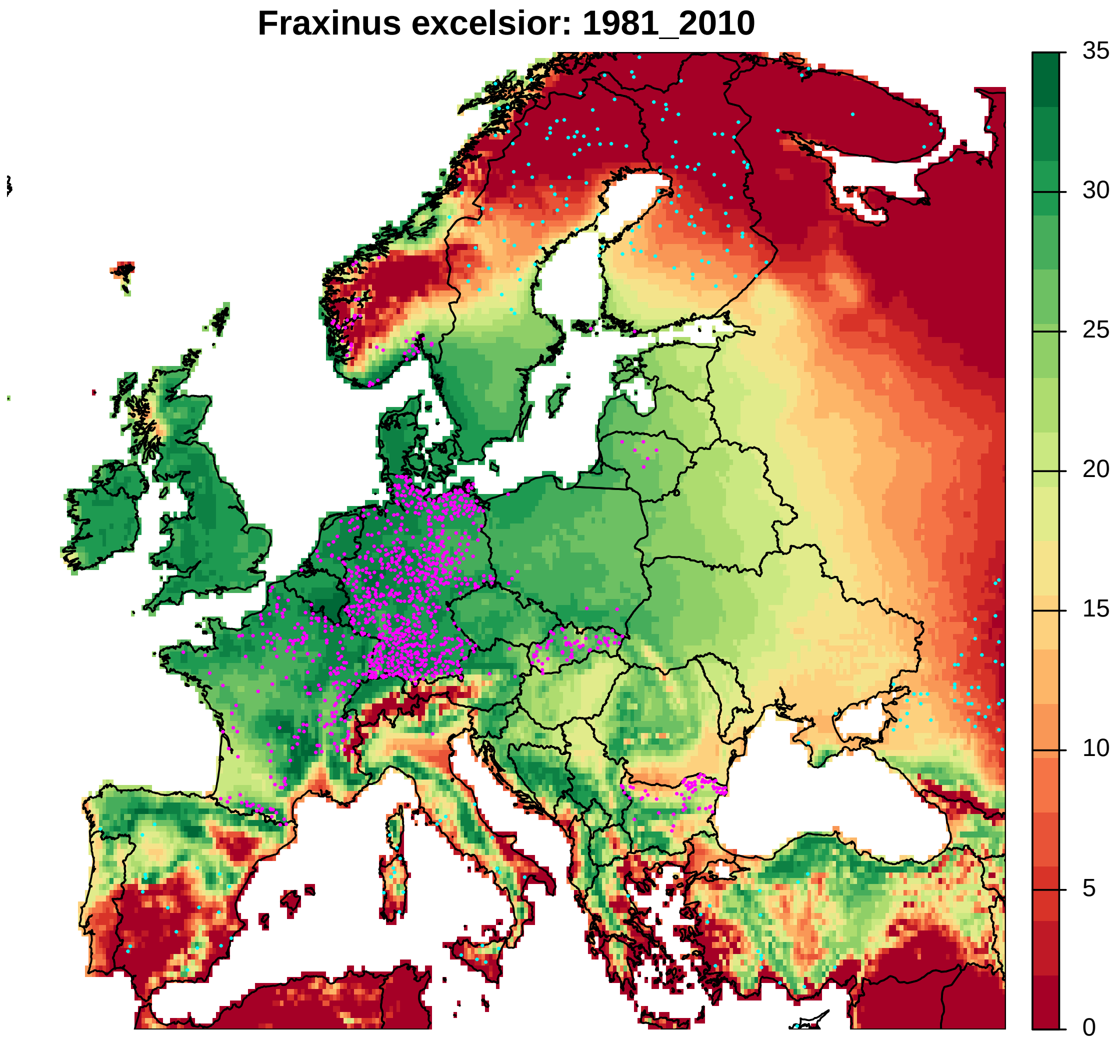

Forecast

Prediction for the reference period (1981 to 2010), as well as forecasts to 2071 to 2100 under szenario RCP4.5 and RCP8.5. Dark green symbolizes a high site index (tree height in m at age 100), orange a lower site index and red no growth. Results were aggregated on 25 km x 25 km scale.

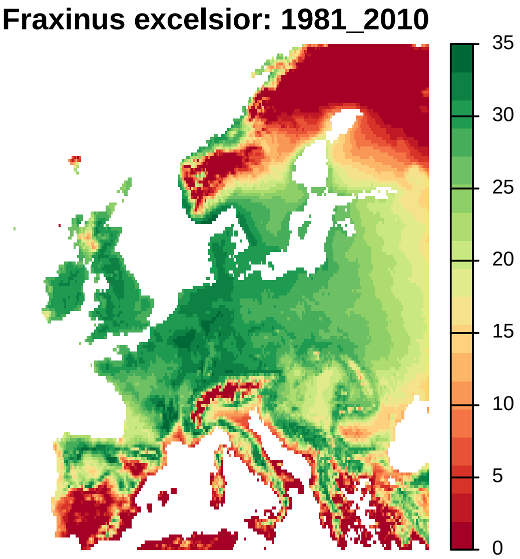

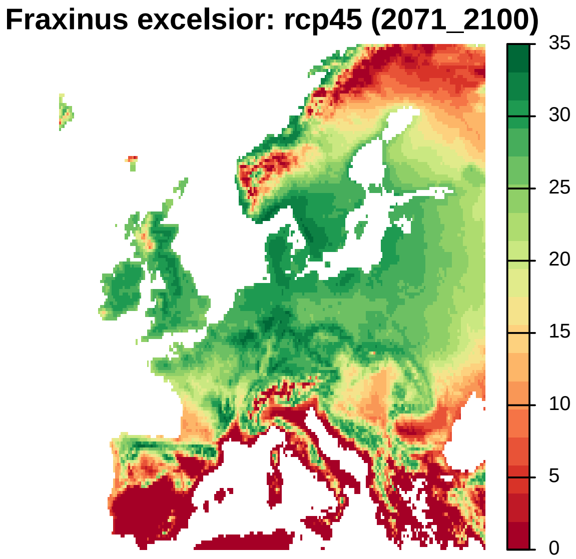

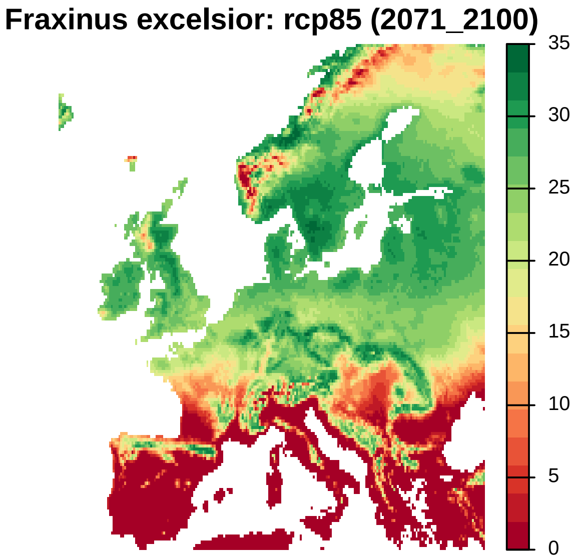

Larix decidua

Site index curves

Site index curves of Larix decidua created with non-linear quantile regressions based on the algorithm of Koenker and Park (1992). The site index (SI) was created by setting all points on the 95 percent quantile (upper line) and above to one (SI = 1) and all on the 5 percent quantile (lower line) and below to zero (SI = 0). The points between the quantile boundaries were assigned a site index between zero and one according to the ratio of their position between the quantile boundaries. We set selected absences (see chapter 2.1.3) on Height = 0 m (at age 100), which means, depending on the site index curves, for each tree species a SI near -1 (red line).

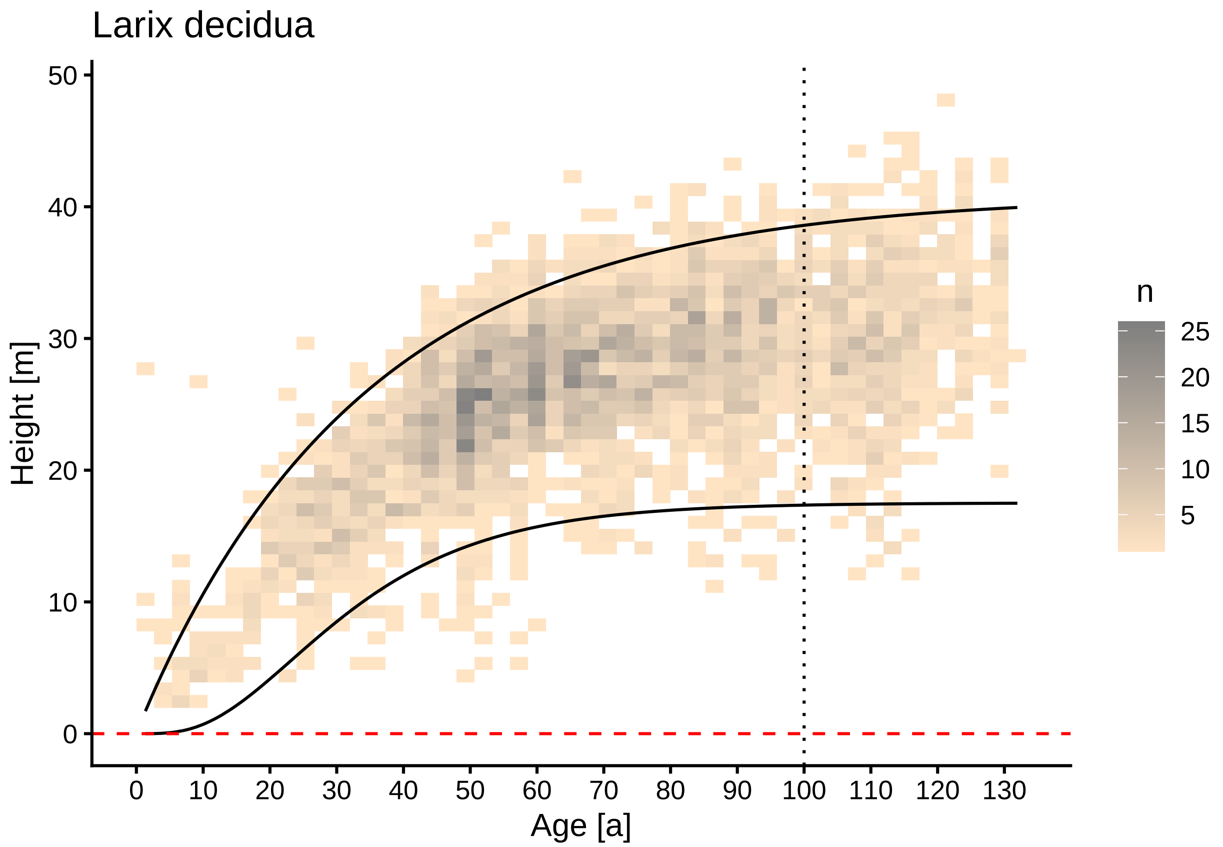

Model statistics and evaluation

Summary

Predictor acronyms: Bio.1 = Mean annual temperature [°C], Bio.12 = Annual precipitation sum [mm/m2], sp_p = Sum of precipitation [mm/m2] within months 3 to 5, su_p = Sum of precipitation [mm/m2] within months 6 to 8, wi_p = Sum of precipitation [mm/m2] within months 12,1,2, sp_t = Mean temperature [°C] within months 3 to 5, su_t = Mean temperature [°C] within months 6 to 8, wi_t = Mean temperature [°C] within months 12,1,2.

```
##
## Family: gaussian
## Link function: identity
##
## Formula:
## H03 ~ s(reference_19812010_Bio.1, k = 3) + s(reference_19812010_Bio.12,
##     k = 3)
##
## Parametric coefficients:
##             Estimate Std. Error t value Pr(>|t|)
## (Intercept)  0.45784    0.01288   35.55   <2e-16 ***
## ---
## Signif. codes:  0 '***' 0.001 '**' 0.01 '*' 0.05 '.' 0.1 ' ' 1
##
## Approximate significance of smooth terms:
##                               edf Ref.df      F p-value
## s(reference_19812010_Bio.1)  1.998      2 335.18  <2e-16 ***
## s(reference_19812010_Bio.12) 1.992      2  77.64  <2e-16 ***
## ---
## Signif. codes:  0 '***' 0.001 '**' 0.01 '*' 0.05 '.' 0.1 ' ' 1
##
## R-sq.(adj) =  0.365   Deviance explained = 36.6%
## -REML = 988.34  Scale est. = 0.23357   n = 1408
```

Variance inflation factor (VIF)

Predictor acronyms: Bio.1 = Mean annual temperature [°C], Bio.12 = Annual precipitation sum [mm/m2], sp_p = Sum of precipitation [mm/m2] within months 3 to 5, su_p = Sum of precipitation [mm/m2] within months 6 to 8, wi_p = Sum of precipitation [mm/m2] within months 12,1,2, sp_t = Mean temperature [°C] within months 3 to 5, su_t = Mean temperature [°C] within months 6 to 8, wi_t = Mean temperature [°C] within months 12,1,2.

```
##                    Variables      VIF
## 1  reference_19812010_Bio.1 1.166267
## 2 reference_19812010_Bio.12 1.166267
```

Correlation matrix

Correlation matrix between the predictor variables and the target variable in the model. Correlation coefficient according to PEARSON. Predictor acronyms: Bio.1 = Mean annual temperature [°C], Bio.12 = Annual precipitation sum [mm/m2], sp_p = Sum of precipitation [mm/m2] within months 3 to 5, su_p = Sum of precipitation [mm/m2] within months 6 to 8, wi_p = Sum of precipitation [mm/m2] within months 12,1,2, sp_t = Mean temperature [°C] within months 3 to 5, su_t = Mean temperature [°C] within months 6 to 8, wi_t = Mean temperature [°C] within months 12,1,2.

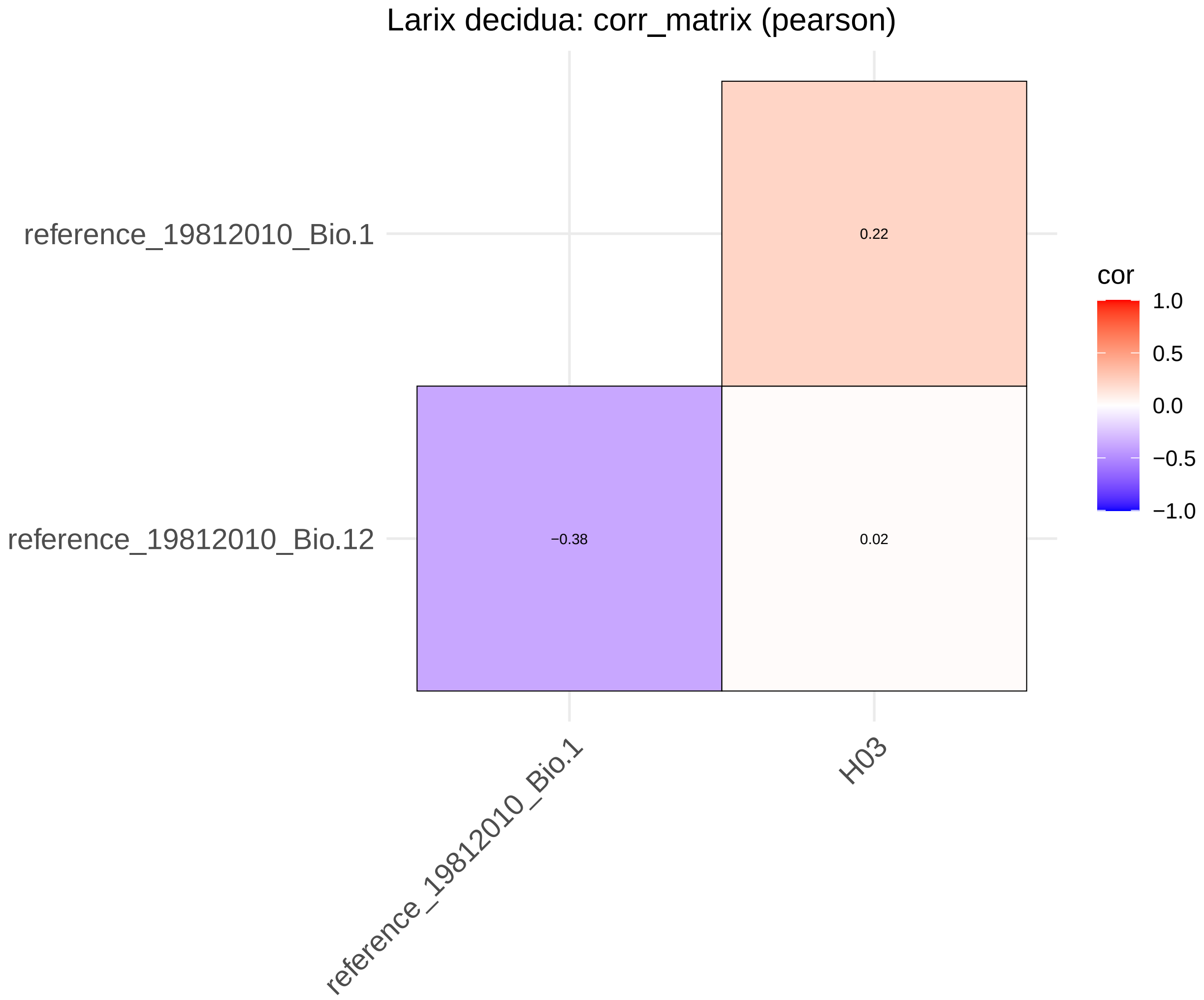

Response curves

Response curves (also known as effect curves) show how each predictor variable affects the target variable (H03 = european Site index, SIrel). H03 values below zero represent 'Growth absences'. Predictor acronyms: Bio.1 = Mean annual temperature [°C], Bio.12 = Annual precipitation sum [mm/m2], sp_p = Sum of precipitation [mm/m2] within months 3 to 5, su_p = Sum of precipitation [mm/m2] within months 6 to 8, wi_p = Sum of precipitation [mm/m2] within months 12,1,2, sp_t = Mean temperature [°C] within months 3 to 5, su_t = Mean temperature [°C] within months 6 to 8, wi_t = Mean temperature [°C] within months 12,1,2.

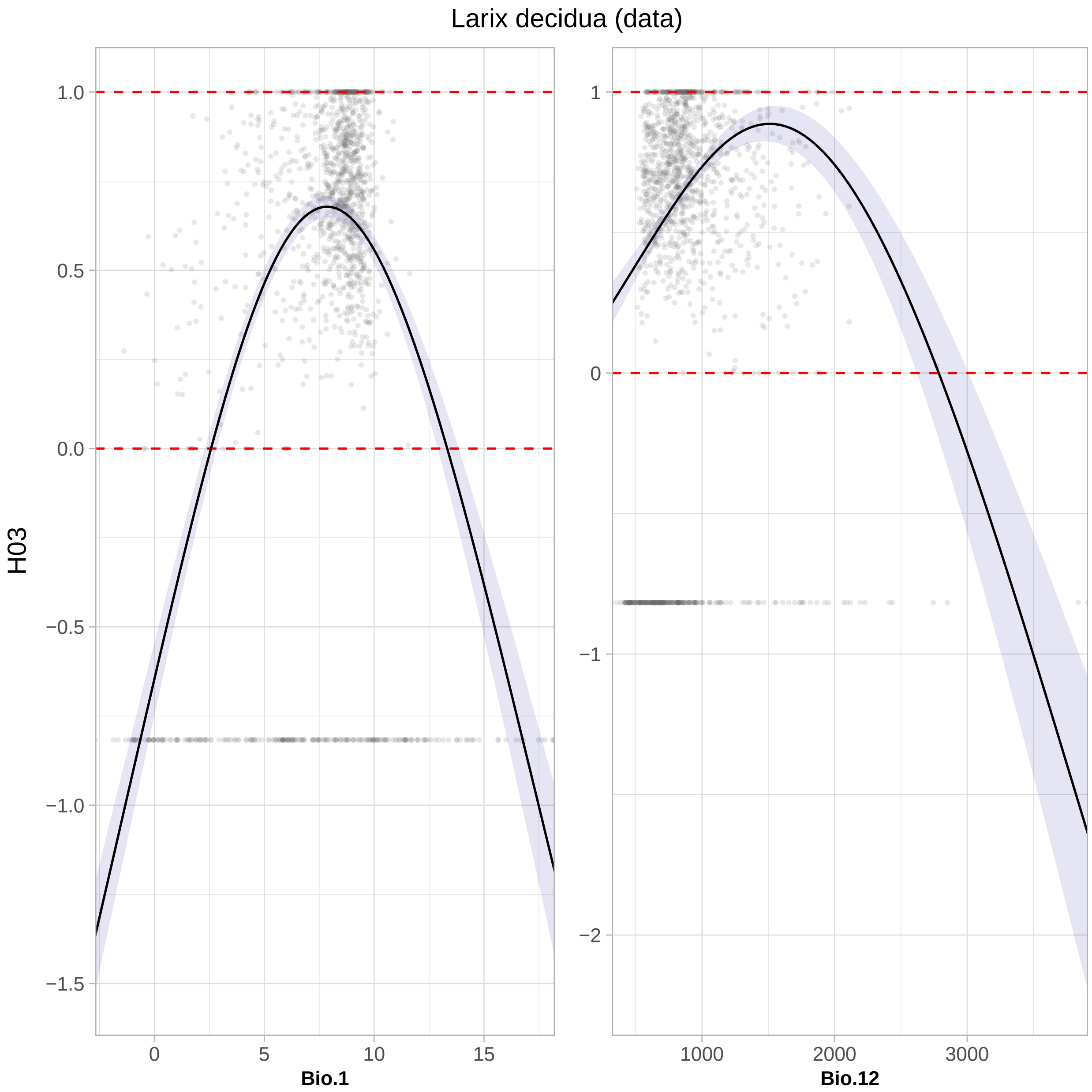

Response maps

Response maps (also referred as partial effect maps). Each map visualizes how a predictor affect the target variable (top height [m] at Age 100). Technically their work like response curves in a geographical area, that is setting all predictor variables except the one shown in the figure on their mean, and mapping the prediction. Predictor acronyms: Bio.1 = Mean annual temperature [°C], Bio.12 = Annual precipitation sum [mm/m2], sp_p = Sum of precipitation [mm/m2] within months 3 to 5, su_p = Sum of precipitation [mm/m2] within months 6 to 8, wi_p = Sum of precipitation [mm/m2] within months 12,1,2, sp_t = Mean temperature [°C] within months 3 to 5, su_t = Mean temperature [°C] within months 6 to 8, wi_t = Mean temperature [°C] within months 12,1,2.

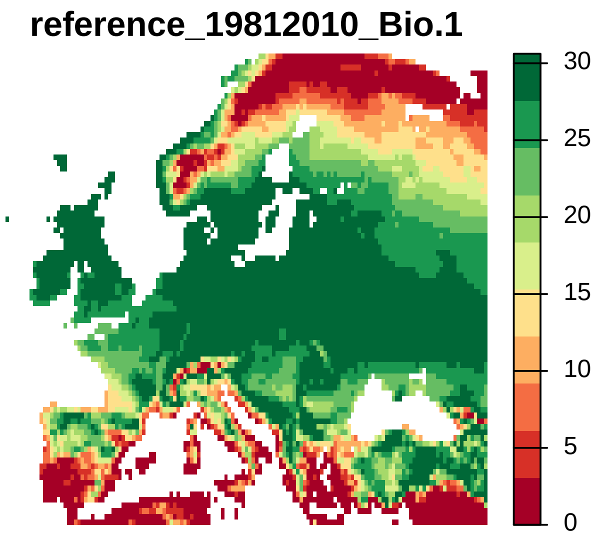

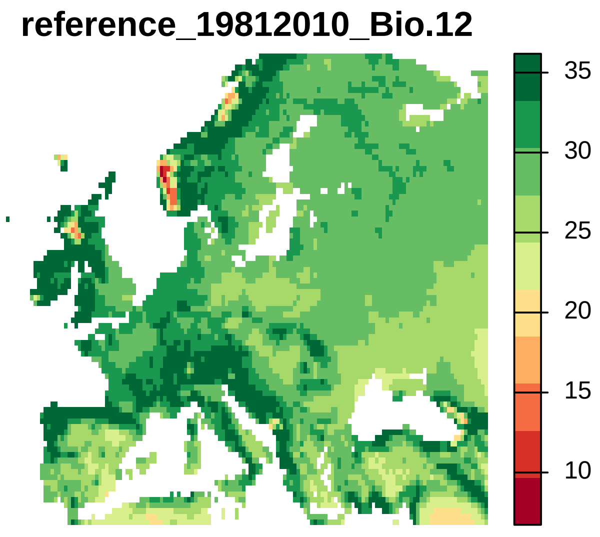

Residual distribution

The multi-panel plot includes a histogram of the residuals (top left), residuals over fitted values (top right), a histogram of observed and predicted values (bottom left) and boxplot diagram of observed and predicted values (bottom right). Observed values are shown in light green, while predicted ones are depicted in light red.

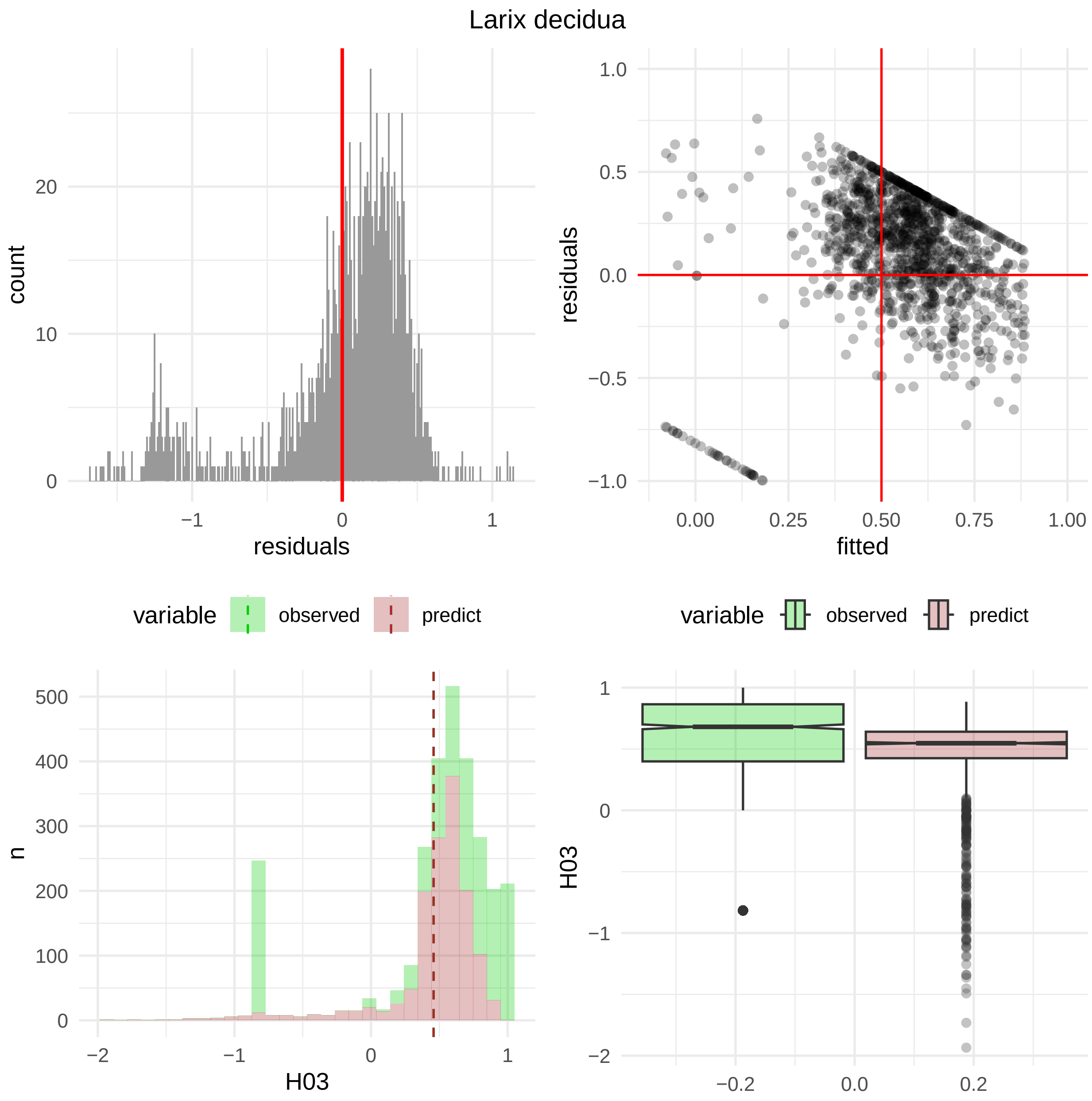

Correlation between predict and observed site index

Relationship between predicted and observed site index (density cloud), as well as linear regressions of presences and absences (= 'growth absences') (red line) and presences only (magenta line). The formulas, significance, R2 and number of observations are displayed below for both regressions. Ideally, both the point cloud and the regression lines lie close to the dashed line. For presences only we additionally calculated the correlation coefficient according to PEARSON (cor.pre) in the bottom right corner.

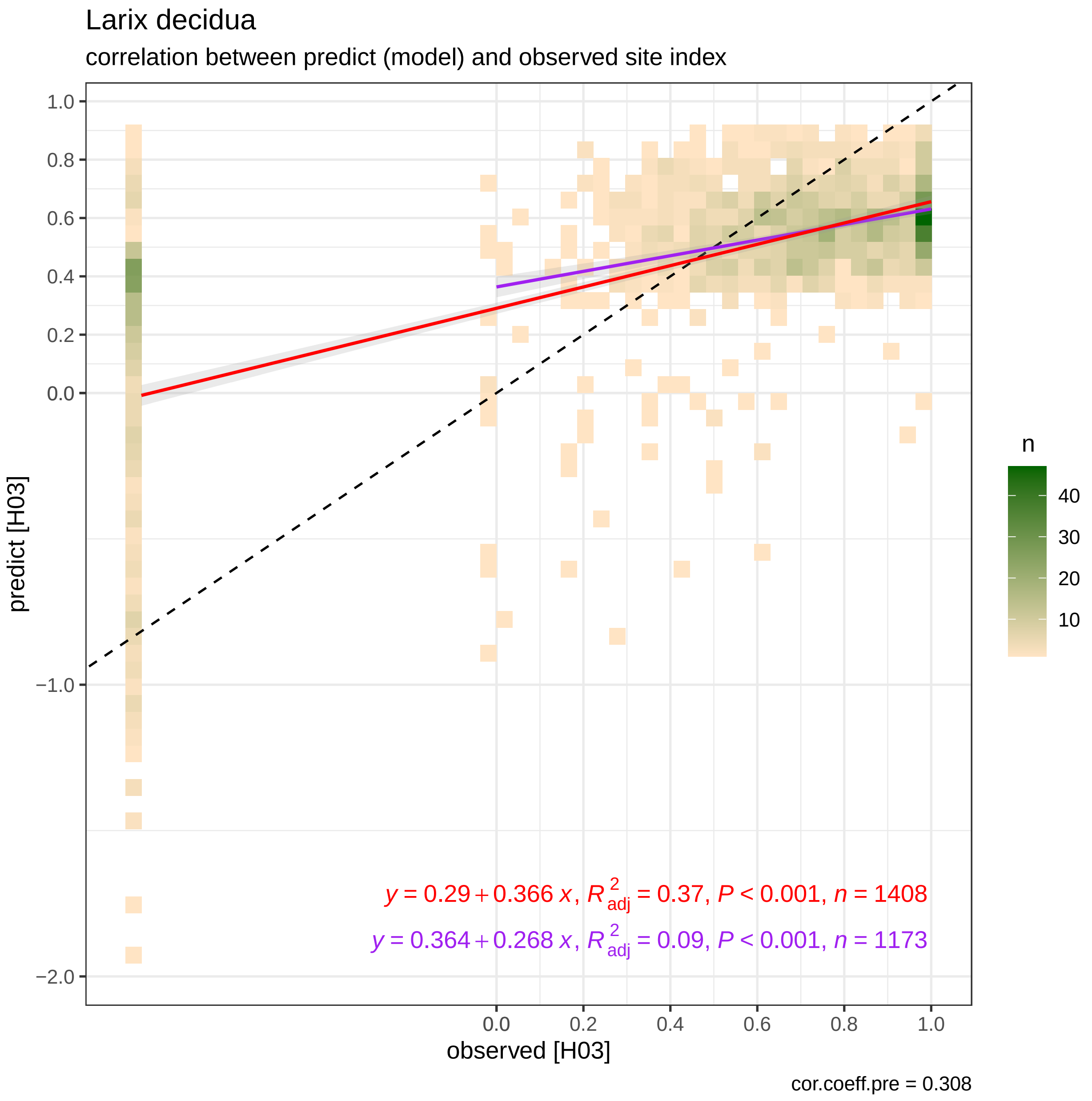

Predictions and forecasts

Predict

European predict for the reference period (1981 to 2010). Dark green symbolizes a high site index (tree height in meters at age 100), orange a lower site index and red no growth. Magenta-coloured dots represent inventory points with growth information, light blue dots are absences (= 'growth absences'). Results were aggregated on 25 km x 25 km scale.

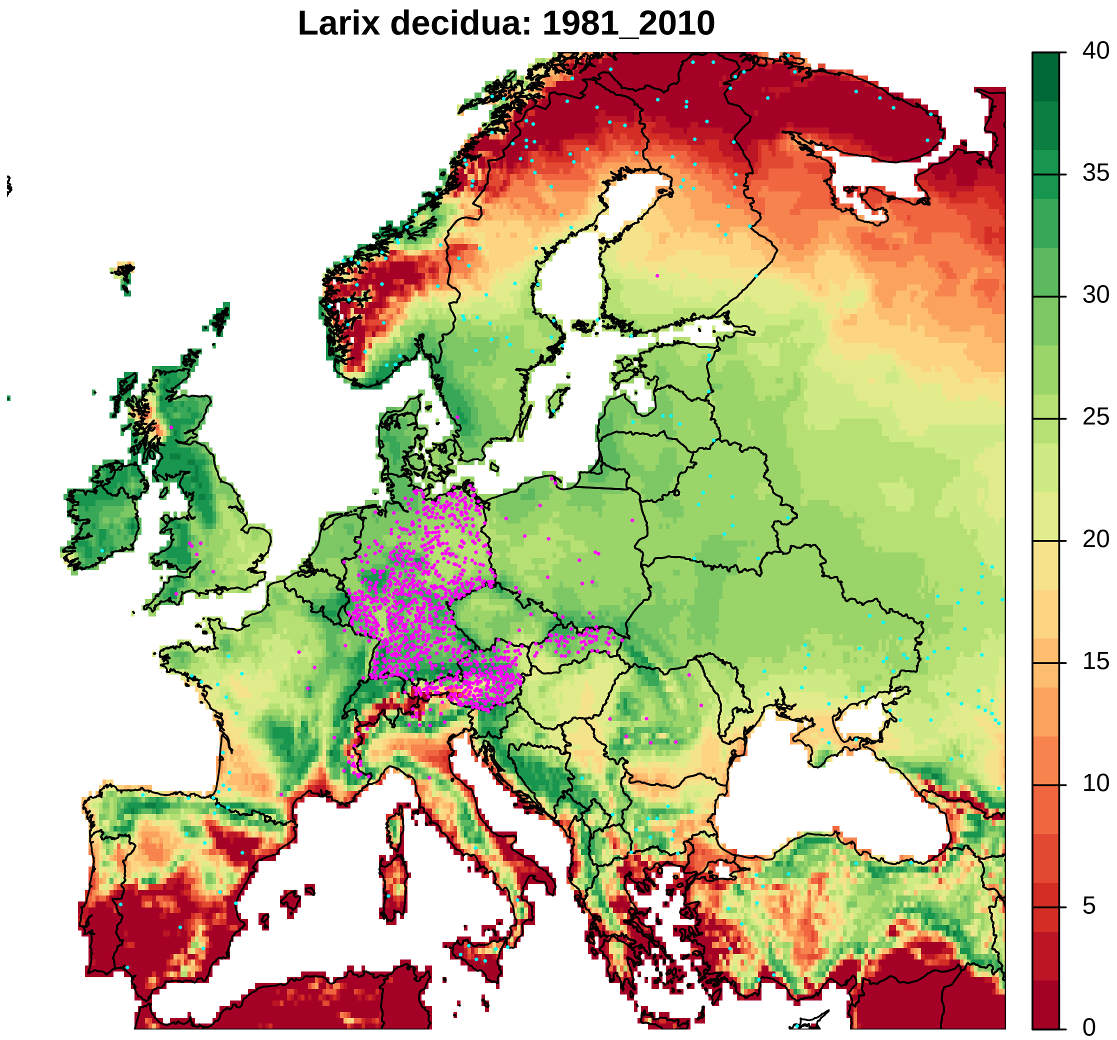

Forecast

Prediction for the reference period (1981 to 2010), as well as forecasts to 2071 to 2100 under szenario RCP4.5 and RCP8.5. Dark green symbolizes a high site index (tree height in m at age 100), orange a lower site index and red no growth. Results were aggregated on 25 km x 25 km scale.

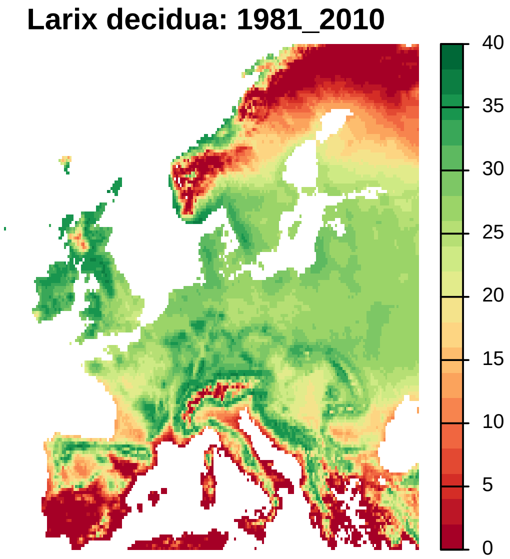

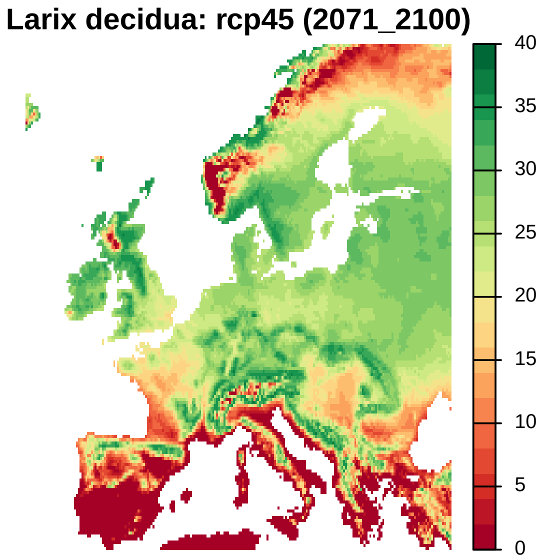

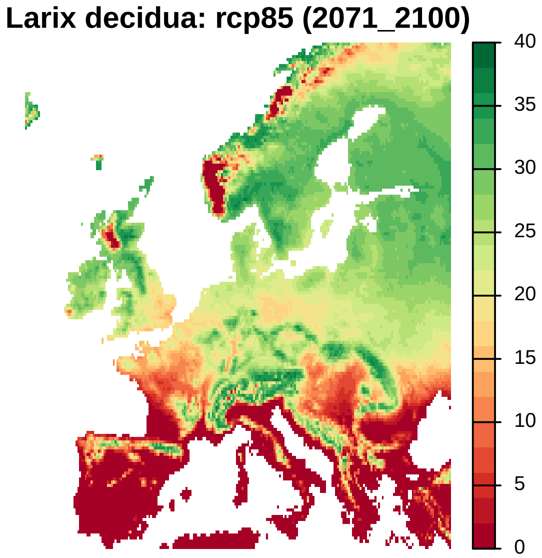

Picea abies

Site index curves

Site index curves of Picea abies created with non-linear quantile regressions based on the algorithm of Koenker and Park (1992). The site index (SI) was created by setting all points on the 95 percent quantile (upper line) and above to one (SI = 1) and all on the 5 percent quantile (lower line) and below to zero (SI = 0). The points between the quantile boundaries were assigned a site index between zero and one according to the ratio of their position between the quantile boundaries. We set selected absences (see chapter 2.1.3) on Height = 0 m (at age 100), which means, depending on the site index curves, for each tree species a SI near -1 (red line).

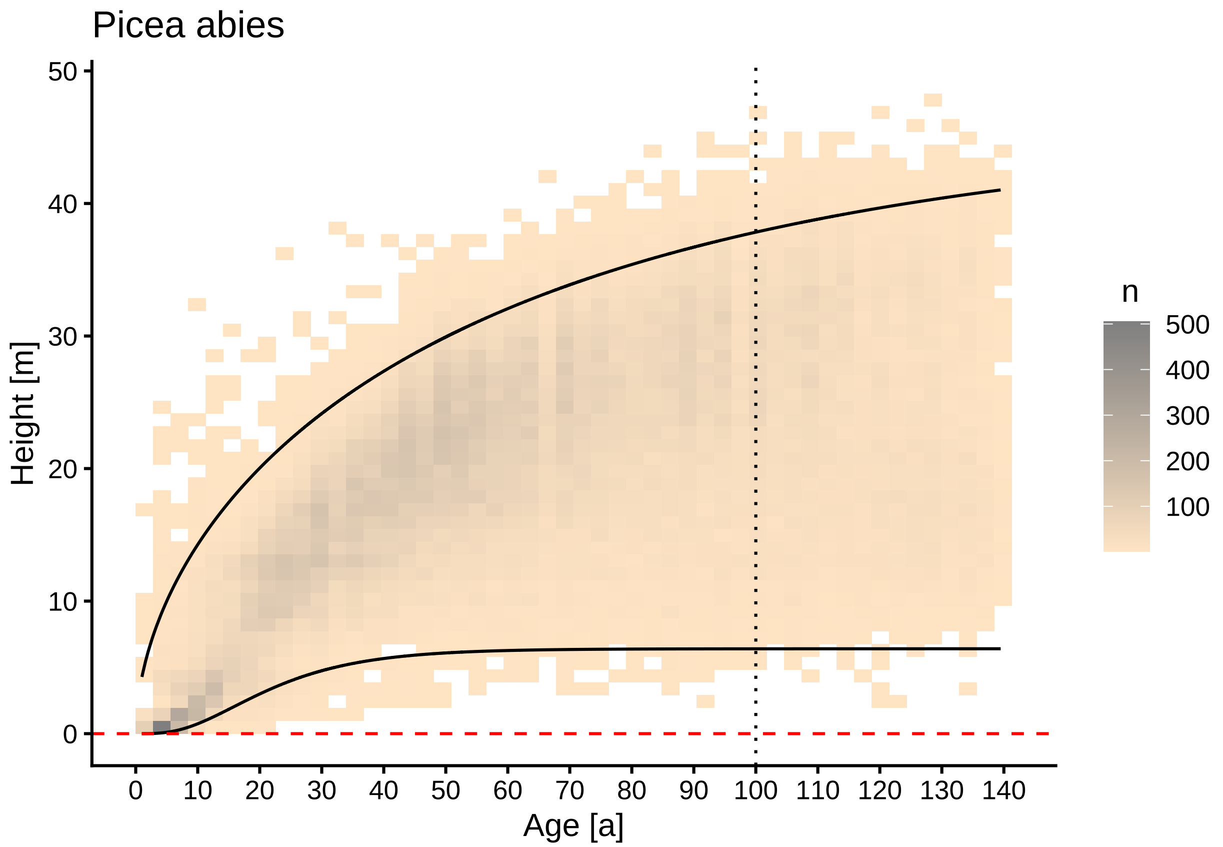

Model statistics and evaluation

Summary

Predictor acronyms: Bio.1 = Mean annual temperature [°C], Bio.12 = Annual precipitation sum [mm/m2], sp_p = Sum of precipitation [mm/m2] within months 3 to 5, su_p = Sum of precipitation [mm/m2] within months 6 to 8, wi_p = Sum of precipitation [mm/m2] within months 12,1,2, sp_t = Mean temperature [°C] within months 3 to 5, su_t = Mean temperature [°C] within months 6 to 8, wi_t = Mean temperature [°C] within months 12,1,2.

```
##
## Family: gaussian
## Link function: identity
##
## Formula:
## H03 ~ s(reference_19812010_su_t, k = 3) + s(reference_19812010_su_p,
##     k = 3) + s(reference_19812010_wi_t, k = 3)
##
## Parametric coefficients:
##             Estimate Std. Error t value Pr(>|t|)
## (Intercept) 0.533488   0.003029   176.1   <2e-16 ***
## ---
## Signif. codes:  0 '***' 0.001 '**' 0.01 '*' 0.05 '.' 0.1 ' ' 1
##
## Approximate significance of smooth terms:
##                              edf Ref.df      F p-value
## s(reference_19812010_su_t) 2.000      2 1450.5  <2e-16 ***
## s(reference_19812010_su_p) 1.996      2  356.2  <2e-16 ***
## s(reference_19812010_wi_t) 1.994      2  429.6  <2e-16 ***
## ---
## Signif. codes:  0 '***' 0.001 '**' 0.01 '*' 0.05 '.' 0.1 ' ' 1
##
## R-sq.(adj) =  0.631   Deviance explained = 63.1%
## -REML = 47.938  Scale est. = 0.058945  n = 6426
```

Variance inflation factor (VIF)

Predictor acronyms: Bio.1 = Mean annual temperature [°C], Bio.12 = Annual precipitation sum [mm/m2], sp_p = Sum of precipitation [mm/m2] within months 3 to 5, su_p = Sum of precipitation [mm/m2] within months 6 to 8, wi_p = Sum of precipitation [mm/m2] within months 12,1,2, sp_t = Mean temperature [°C] within months 3 to 5, su_t = Mean temperature [°C] within months 6 to 8, wi_t = Mean temperature [°C] within months 12,1,2.

```
##                  Variables      VIF
## 1 reference_19812010_su_t 2.608766
## 2 reference_19812010_su_p 1.714842
## 3 reference_19812010_wi_t 1.746163
```

Correlation matrix

Correlation matrix between the predictor variables and the target variable in the model. Correlation coefficient according to PEARSON. Predictor acronyms: Bio.1 = Mean annual temperature [°C], Bio.12 = Annual precipitation sum [mm/m2], sp_p = Sum of precipitation [mm/m2] within months 3 to 5, su_p = Sum of precipitation [mm/m2] within months 6 to 8, wi_p = Sum of precipitation [mm/m2] within months 12,1,2, sp_t = Mean temperature [°C] within months 3 to 5, su_t = Mean temperature [°C] within months 6 to 8, wi_t = Mean temperature [°C] within months 12,1,2.

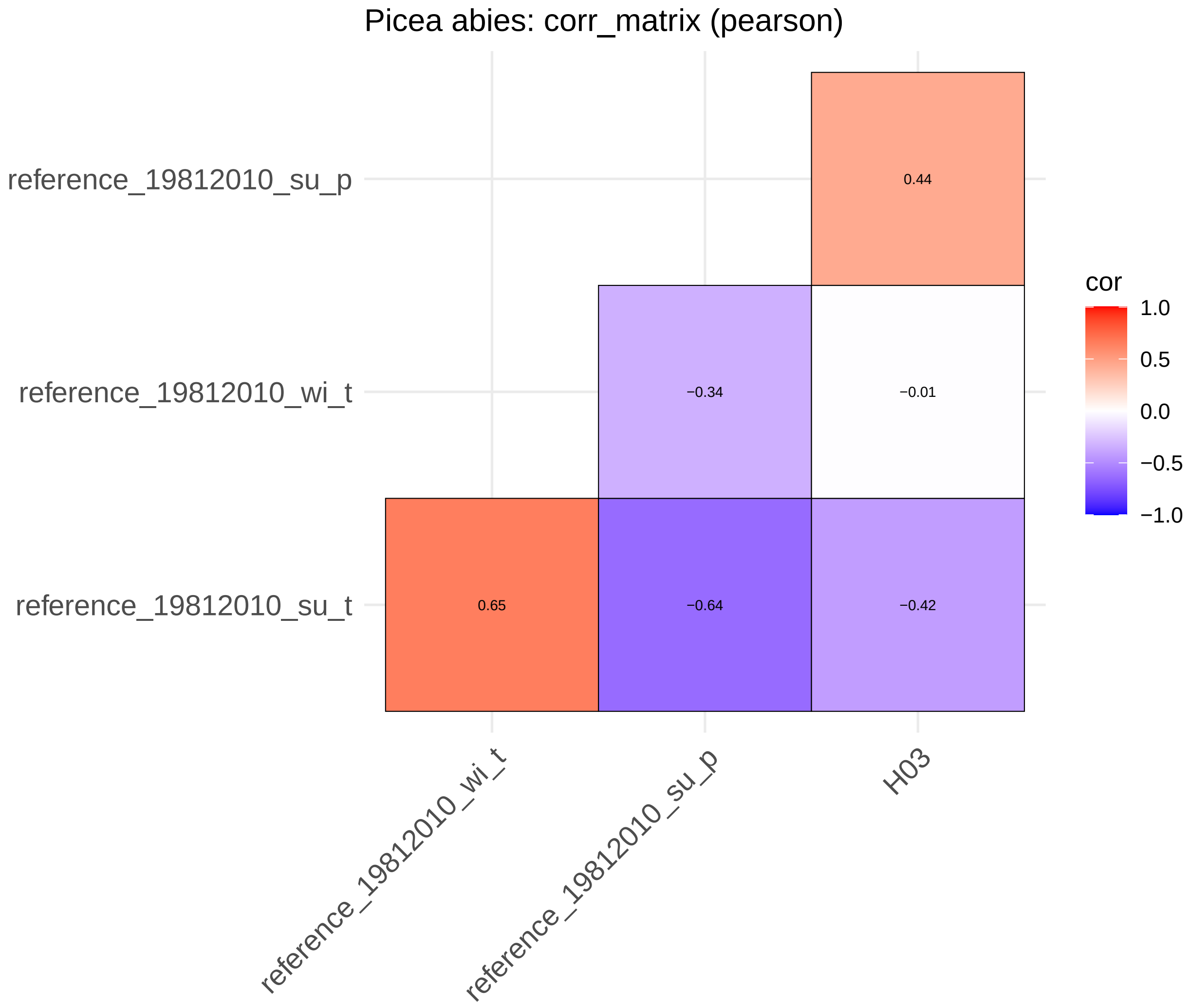

Response curves

Response curves (also known as effect curves) show how each predictor variable affects the target variable (H03 = european Site index, SIrel). H03 values below zero represent ‘Growth absences’. Predictor acronyms: Bio.1 = Mean annual temperature [°C], Bio.12 = Annual precipitation sum [mm/m2], sp_p = Sum of precipitation [mm/m2] within months 3 to 5, su_p = Sum of precipitation [mm/m2] within months 6 to 8, wi_p = Sum of precipitation [mm/m2] within months 12,1,2, sp_t = Mean temperature [°C] within months 3 to 5, su_t = Mean temperature [°C] within months 6 to 8, wi_t = Mean temperature [°C] within months 12,1,2.

Picea abies (data)

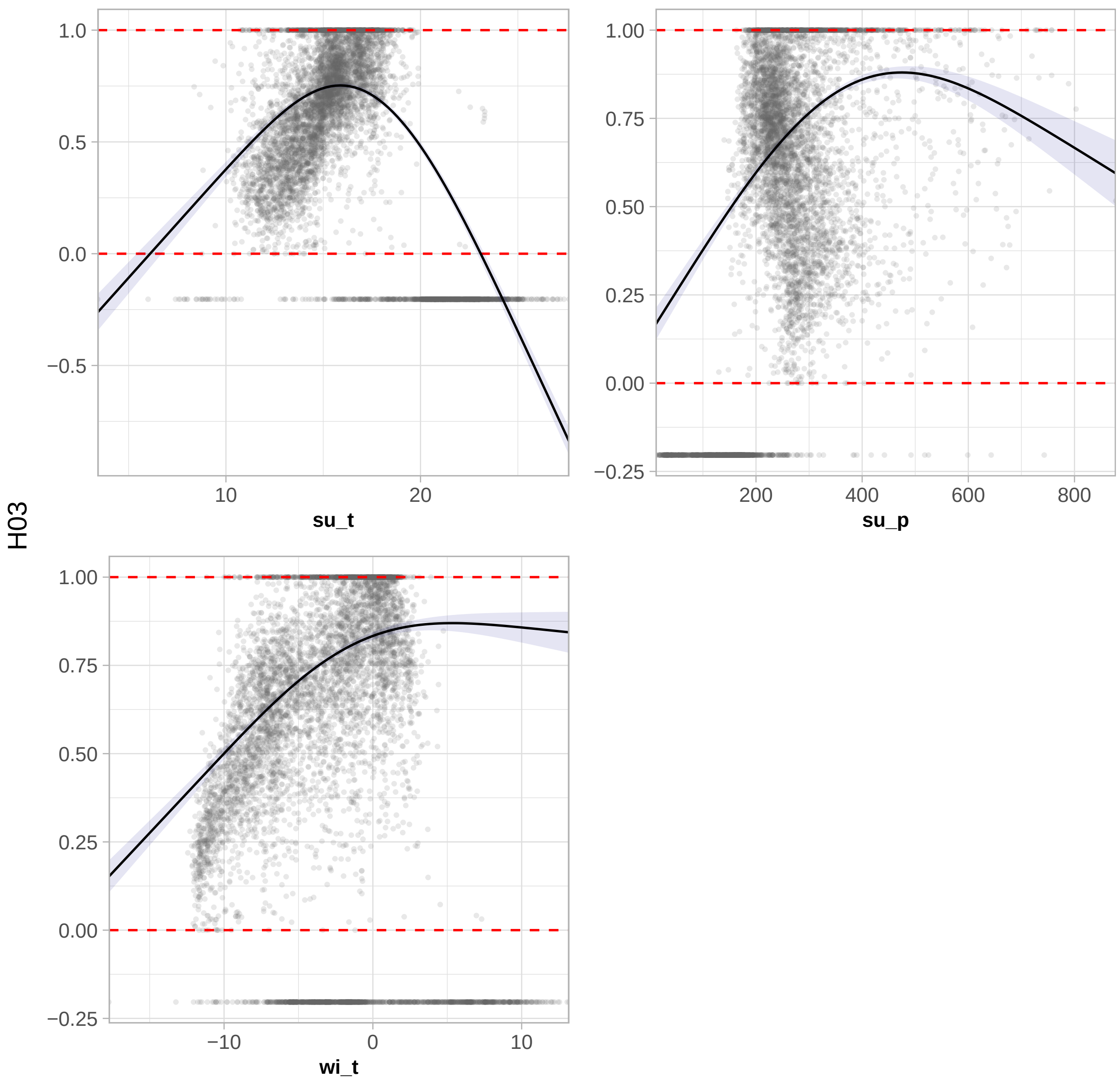

Response maps

Response maps (also referred as partial effect maps). Each map visualizes how a predictor affect the target variable (top height [m] at Age 100). Technically their work like response curves in a geographical area, that is setting all predictor variables except the one shown in the figure on their mean, and mapping the prediction. Predictor acronyms: Bio.1 = Mean annual temperature [°C], Bio.12 = Annual precipitation sum [mm/m2], sp_p = Sum of precipitation [mm/m2] within months 3 to 5, su_p = Sum of precipitation [mm/m2] within months 6 to 8, wi_p = Sum of precipitation [mm/m2] within months 12,1,2, sp_t = Mean temperature [°C] within months 3 to 5, su_t = Mean temperature [°C] within months 6 to 8, wi_t = Mean temperature [°C] within months 12,1,2.

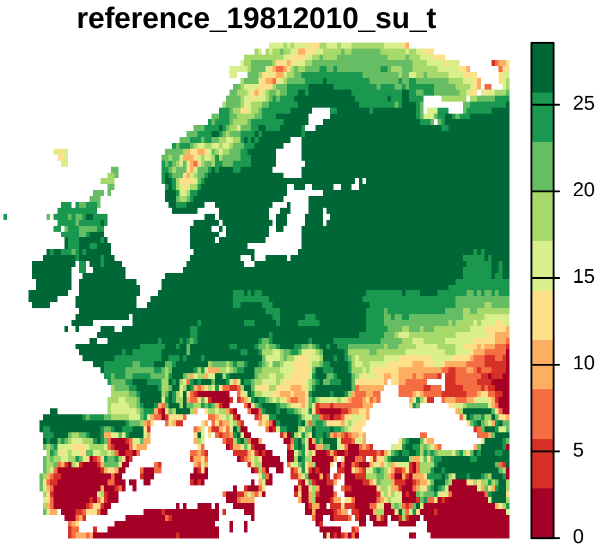

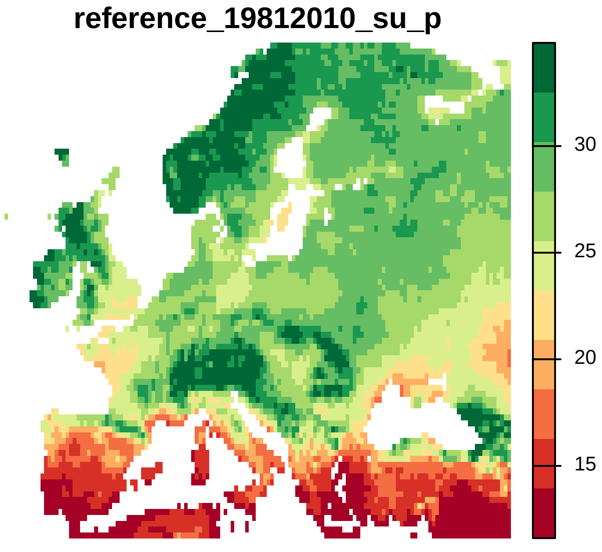

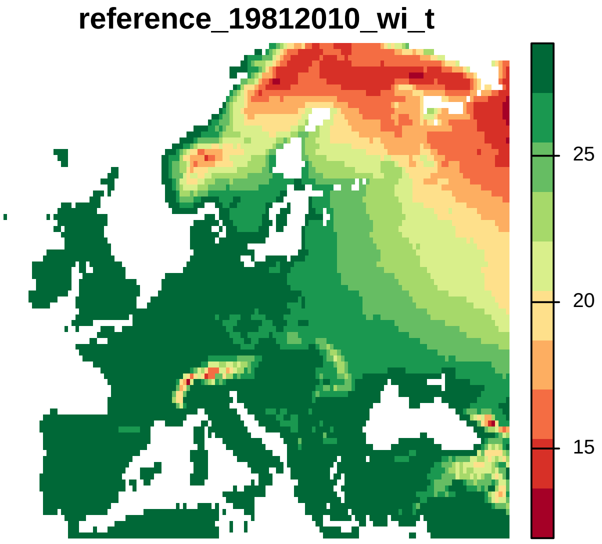

Residual distribution

The multi-panel plot includes a histogram of the residuals (top left), residuals over fitted values (top right), a histogram of observed and predicted values (bottom left) and boxplot diagram of observed and predicted values (bottom right). Observed values are shown in light green, while predicted ones are depicted in light red.

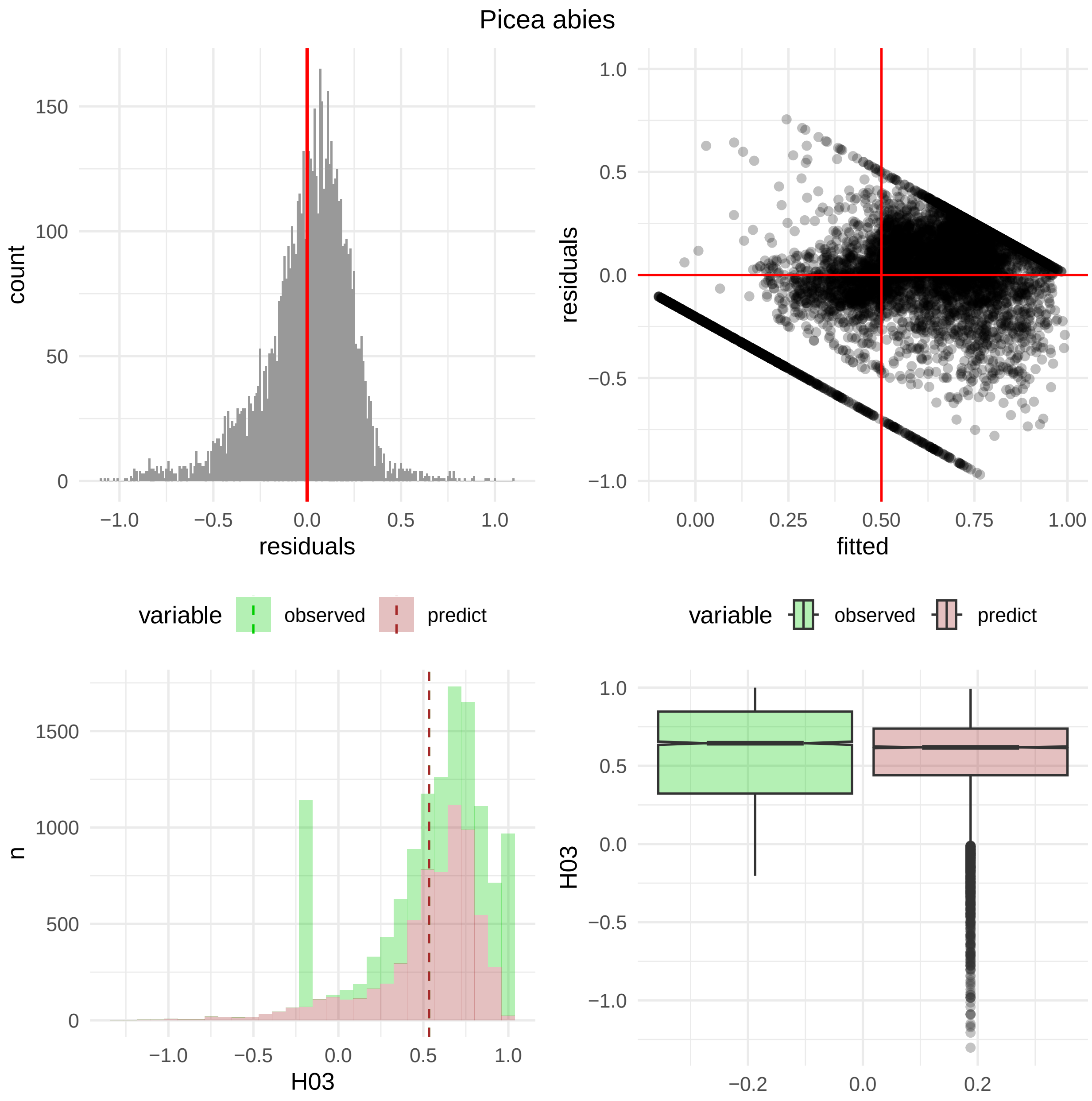

Correlation between predict and observed site index

Relationship between predicted and observed site index (density cloud), as well as linear regressions of presences and absences (= 'growth absences') (red line) and presences only (magenta line). The formulas, significance, R2 and number of observations are displayed below for both regressions. Ideally, both the point cloud and the regression lines lie close to the dashed line. For presences only we additionally calculated the correlation coefficient according to PEARSON (cor.pre) in the bottom right corner.

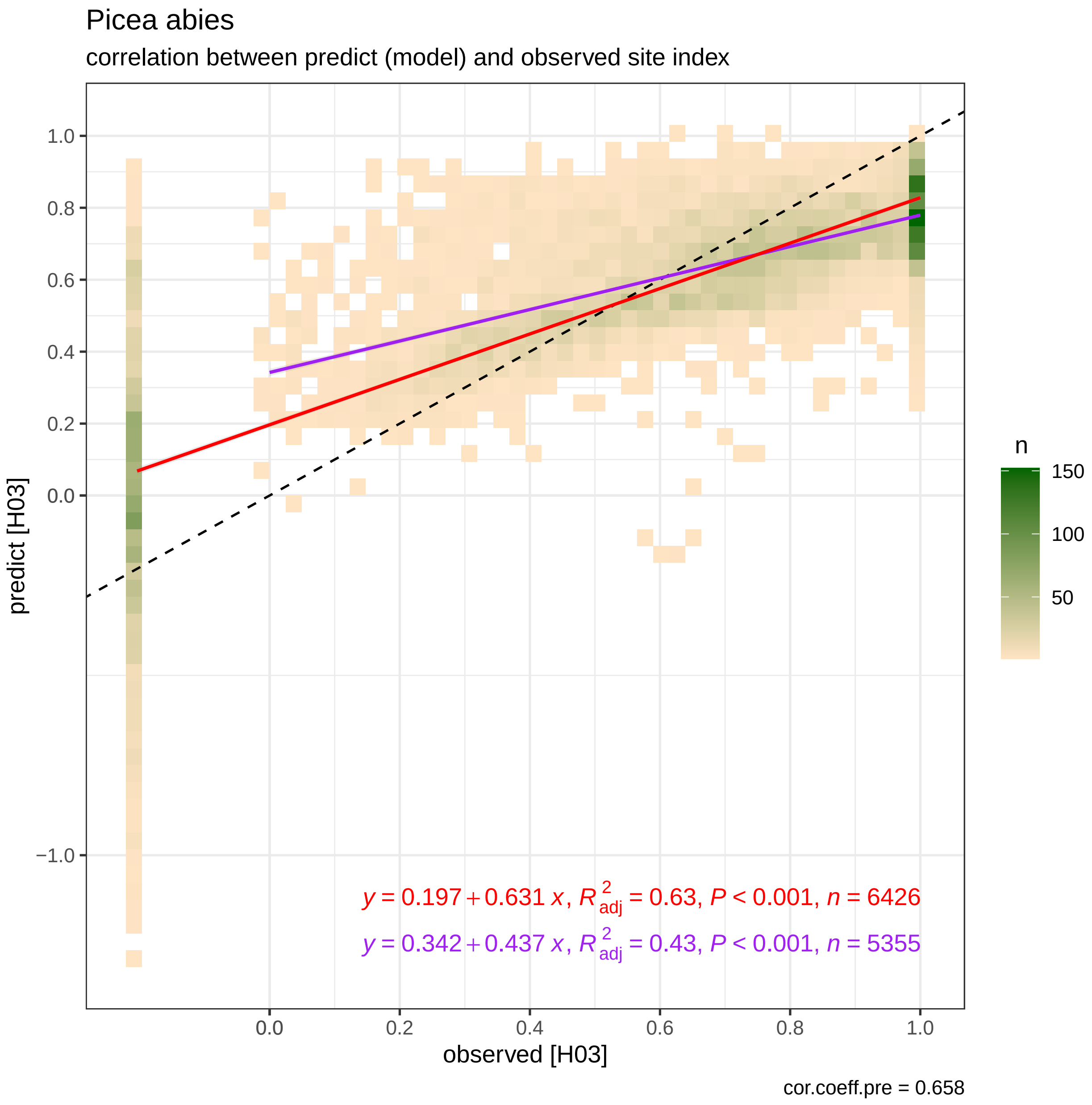

Predictions and forecasts

Predict

European predict for the reference period (1981 to 2010). Dark green symbolizes a high site index (tree height in meters at age 100), orange a lower site index and red no growth. Magenta-coloured dots represent inventory points with growth information, light blue dots are absences (= 'growth absences'). Results were aggregated on 25 km x 25 km scale.

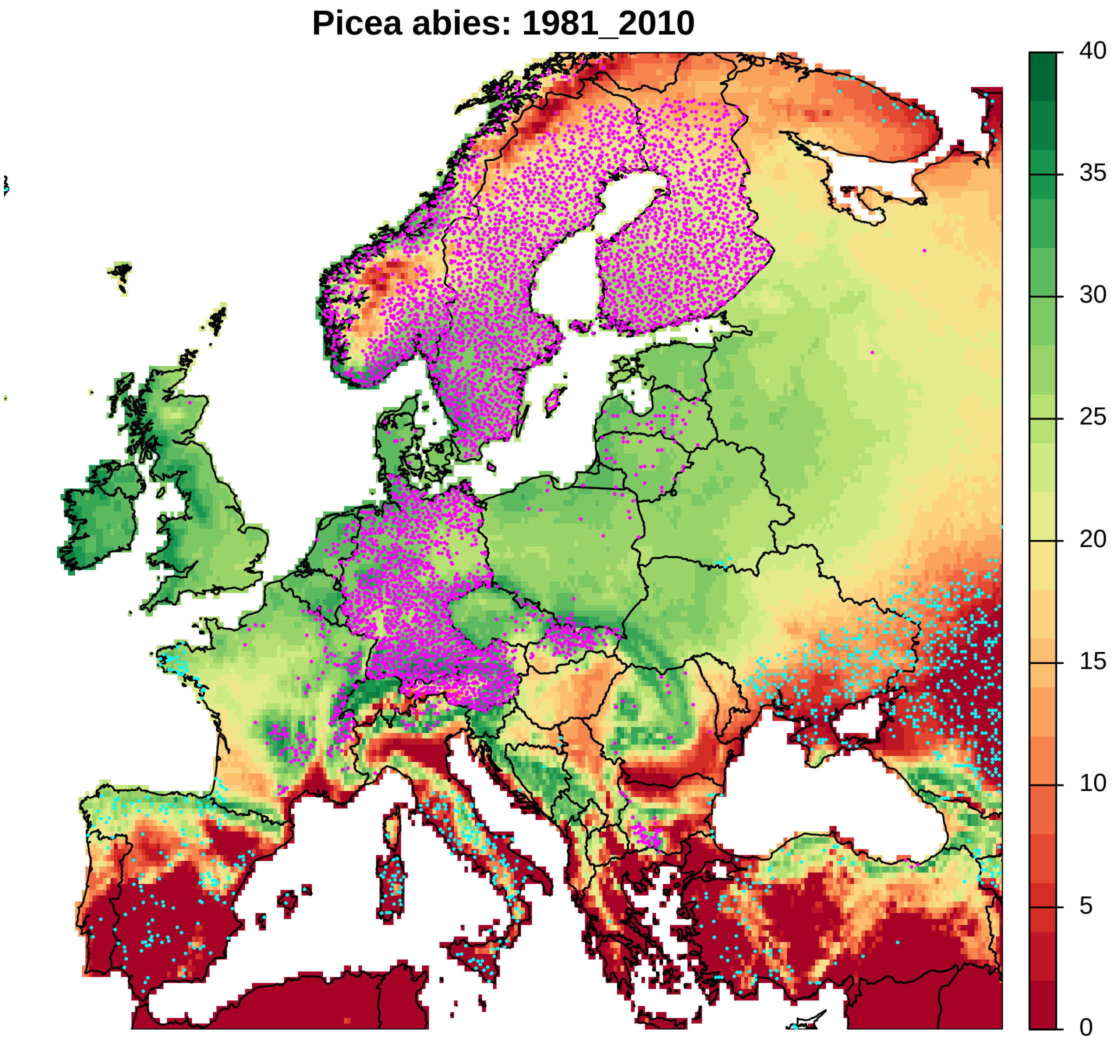

Forecast

Prediction for the reference period (1981 to 2010), as well as forecasts to 2071 to 2100 under szenario RCP4.5 and RCP8.5. Dark green symbolizes a high site index (tree height in m at age 100), orange a lower site index and red no growth. Results were aggregated on 25 km x 25 km scale.

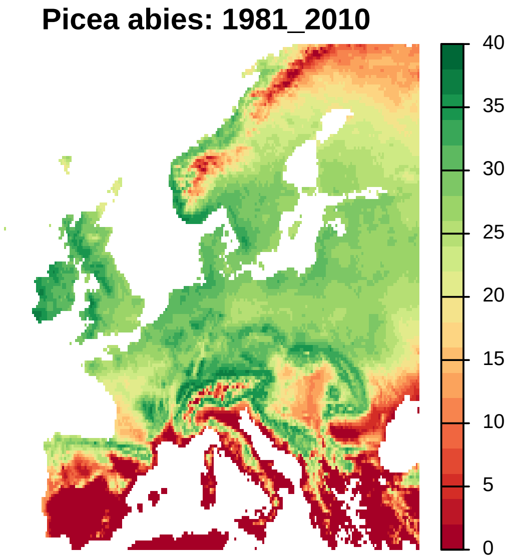

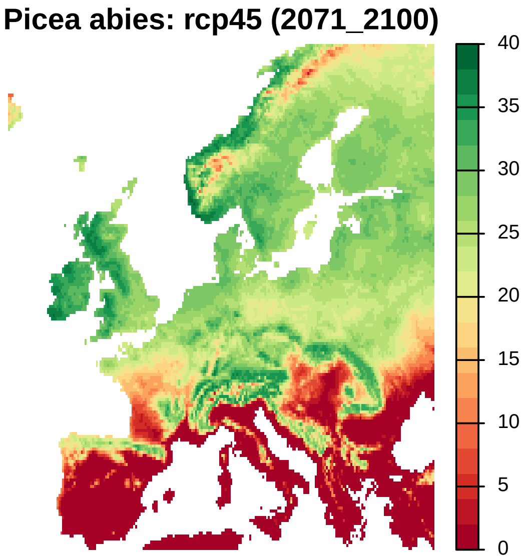

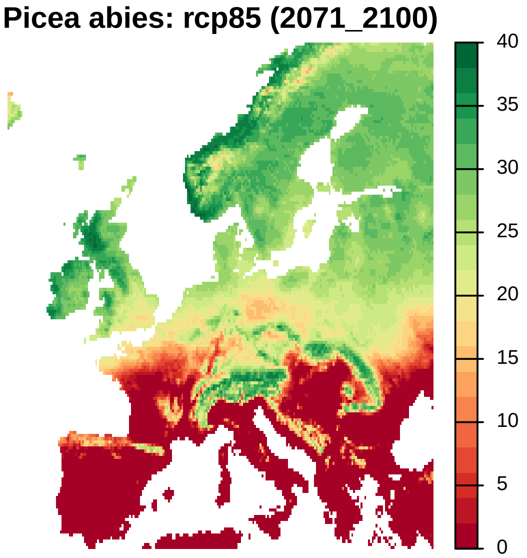

Pinus nigra

Site index curves

Site index curves of Pinus nigra created with non-linear quantile regressions based on the algorithm of Koenker and Park (1992). The site index (SI) was created by setting all points on the 95 percent quantile (upper line) and above to one (SI = 1) and all on the 5 percent quantile (lower line) and below to zero (SI = 0). The points between the quantile boundaries were assigned a site index between zero and one according to the ratio of their position between the quantile boundaries. We set selected absences (see chapter 2.1.3) on Height = 0 m (at age 100), which means, depending on the site index curves, for each tree species a SI near -1 (red line).

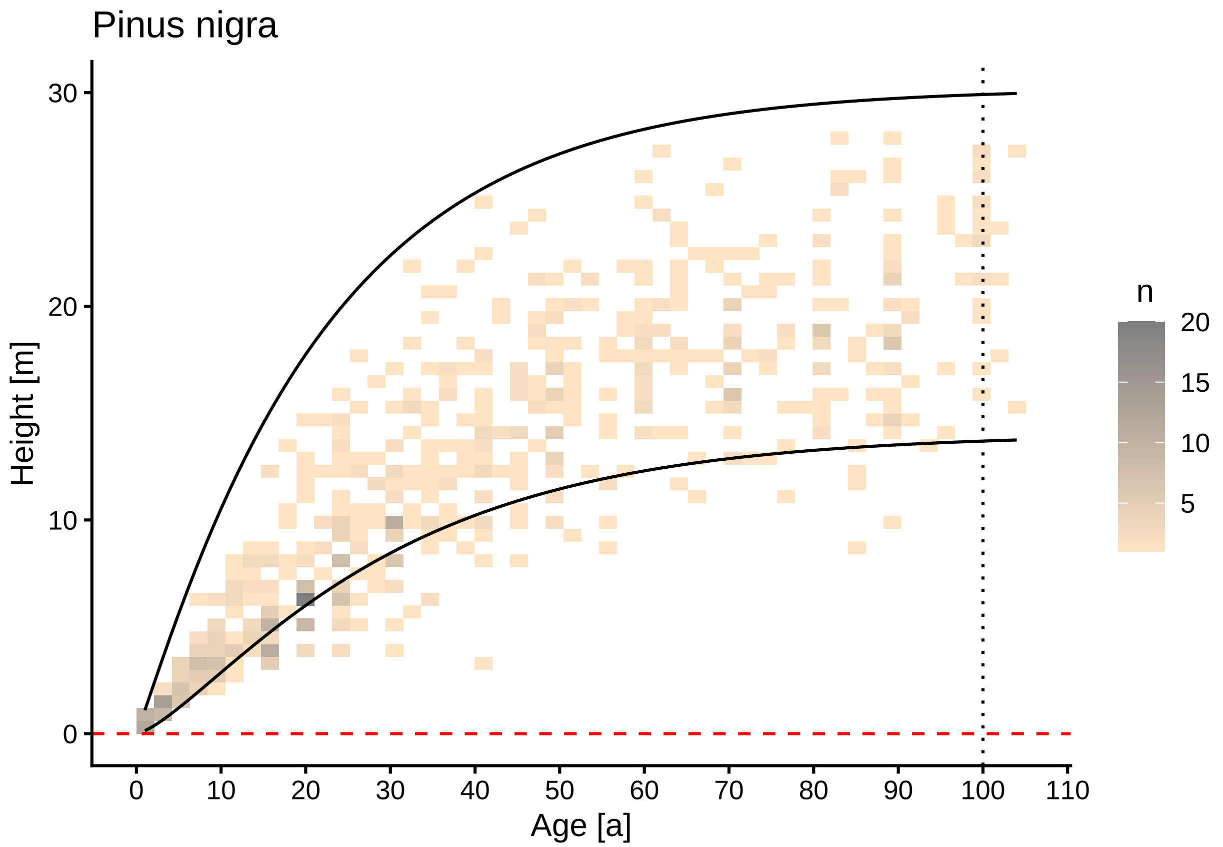

Model statistics and evaluation

Summary

Predictor acronyms: Bio.1 = Mean annual temperature [°C], Bio.12 = Annual precipitation sum [mm/m2], sp_p = Sum of precipitation [mm/m2] within months 3 to 5, su_p = Sum of precipitation [mm/m2] within months 6 to 8, wi_p = Sum of precipitation [mm/m2] within months 12,1,2, sp_t = Mean temperature [°C] within months 3 to 5, su_t = Mean temperature [°C] within months 6 to 8, wi_t = Mean temperature [°C] within months 12,1,2.

```
##
## Family: gaussian
## Link function: identity
##
## Formula:
## H03 ~ s(reference_19812010_wi_t, k = 3) + s(reference_19812010_sp_p,
##     k = 3) + s(reference_19812010_su_p, k = 3)
##
## Parametric coefficients:
##             Estimate Std. Error t value Pr(>|t|)
## (Intercept)  0.14807    0.01699   8.714   <2e-16 ***
## ---
## Signif. codes:  0 '***' 0.001 '**' 0.01 '*' 0.05 '.' 0.1 ' ' 1
##
## Approximate significance of smooth terms:
##                              edf Ref.df      F  p-value
## s(reference_19812010_wi_t) 1.966  1.999 115.19  < 2e-16 ***
## s(reference_19812010_sp_p) 1.949  1.997  12.48 7.81e-06 ***
## s(reference_19812010_su_p) 1.953  1.997  27.46  < 2e-16 ***
## ---
## Signif. codes:  0 '***' 0.001 '**' 0.01 '*' 0.05 '.' 0.1 ' ' 1
##
## R-sq.(adj) =  0.542   Deviance explained = 54.9%
## -REML = 145.95  Scale est. = 0.11377   n = 394
```

Variance inflation factor (VIF)

Predictor acronyms: Bio.1 = Mean annual temperature [°C], Bio.12 = Annual precipitation sum [mm/m2], sp_p = Sum of precipitation [mm/m2] within months 3 to 5, su_p = Sum of precipitation [mm/m2] within months 6 to 8, wi_p = Sum of precipitation [mm/m2] within months 12,1,2, sp_t = Mean temperature [°C] within months 3 to 5, su_t = Mean temperature [°C] within months 6 to 8, wi_t = Mean temperature [°C] within months 12,1,2.

```
##                  Variables      VIF
## 1 reference_19812010_wi_t 1.729142
## 2 reference_19812010_sp_p 1.466856
## 3 reference_19812010_su_p 1.967429
```

Correlation matrix

Correlation matrix between the predictor variables and the target variable in the model. Correlation coefficient according to PEARSON. Predictor acronyms: Bio.1 = Mean annual temperature [°C], Bio.12 = Annual precipitation sum [mm/m2], sp_p = Sum of precipitation [mm/m2] within months 3 to 5, su_p = Sum of precipitation [mm/m2] within months 6 to 8, wi_p = Sum of precipitation [mm/m2] within months 12,1,2, sp_t = Mean temperature [°C] within months 3 to 5, su_t = Mean temperature [°C] within months 6 to 8, wi_t = Mean temperature [°C] within months 12,1,2.

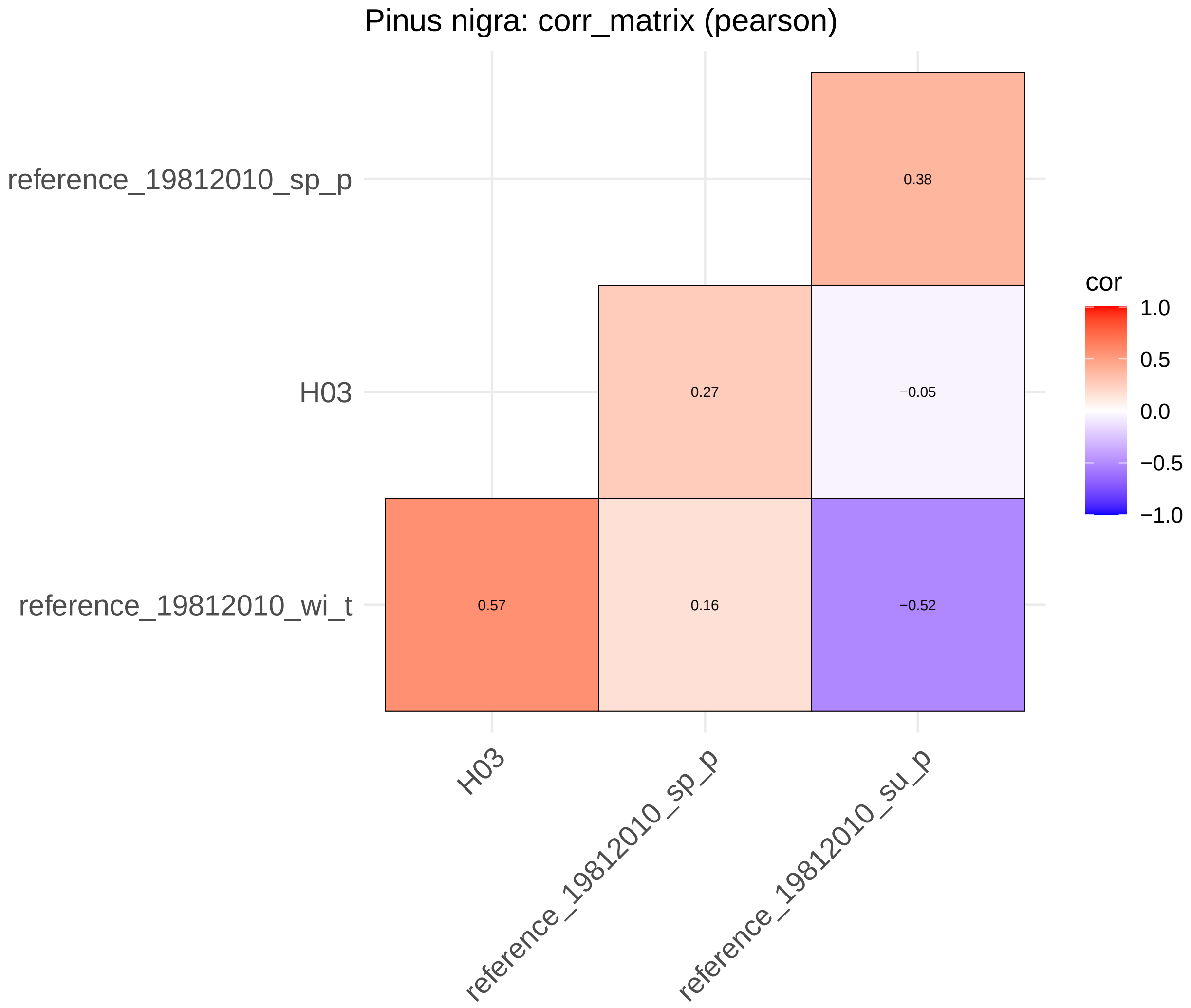

Response curves

Response curves (also known as effect curves) show how each predictor variable affects the target variable (H03 = european Site index, SIrel). H03 values below zero represent 'Growth absences'. Predictor acronyms: Bio.1 = Mean annual temperature [°C], Bio.12 = Annual precipitation sum [mm/m2], sp_p = Sum of precipitation [mm/m2] within months 3 to 5, su_p = Sum of precipitation [mm/m2] within months 6 to 8, wi_p = Sum of precipitation [mm/m2] within months 12,1,2, sp_t = Mean temperature [°C] within months 3 to 5, su_t = Mean temperature [°C] within months 6 to 8, wi_t = Mean temperature [°C] within months 12,1,2.

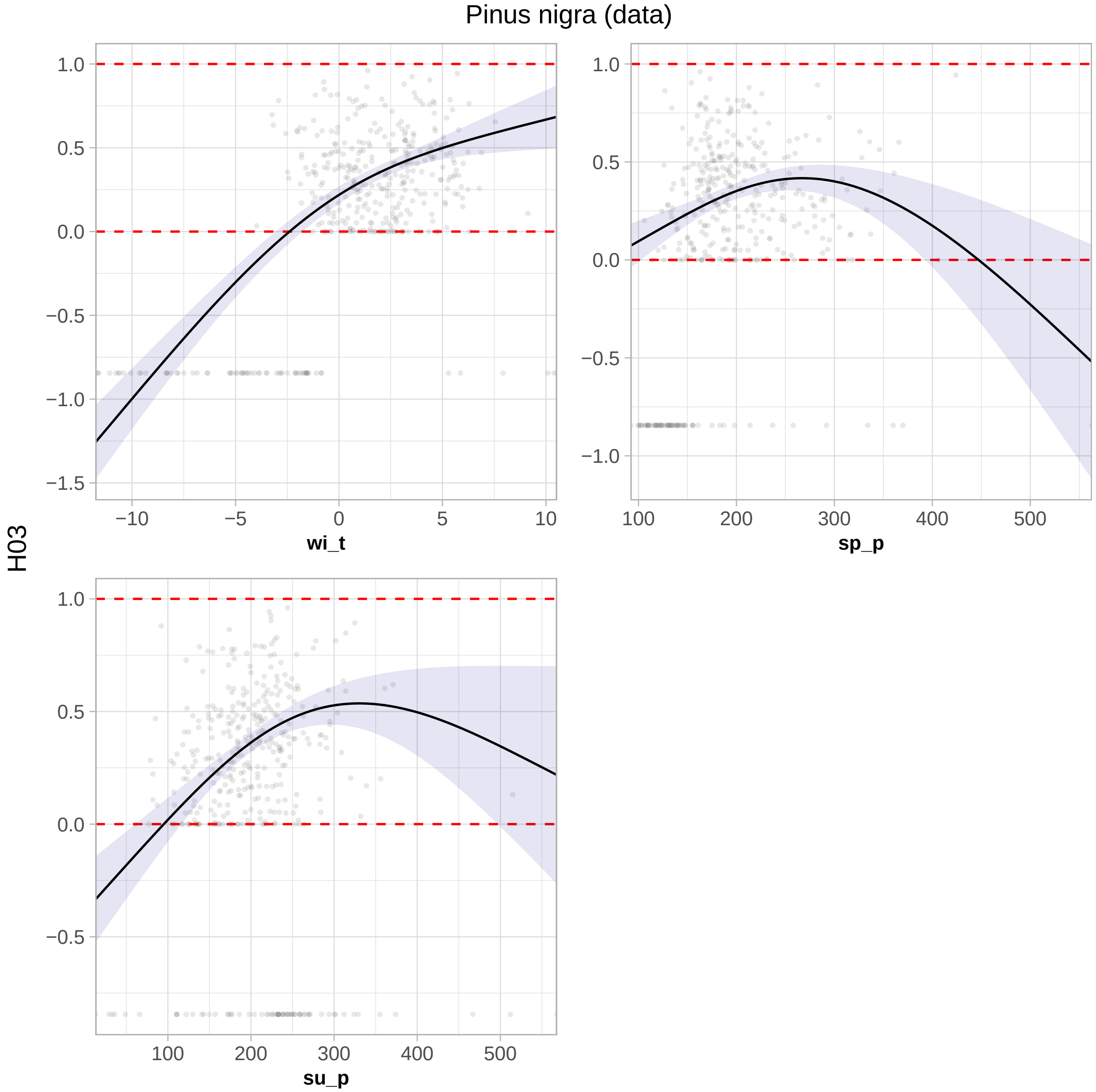

Response maps

Response maps (also referred as partial effect maps). Each map visualizes how a predictor affect the target variable (top height [m] at Age 100). Technically their work like response curves in a geographical area, that is setting all predictor variables except the one shown in the figure on their mean, and mapping the prediction. Predictor acronyms: Bio.1 = Mean annual temperature [°C], Bio.12 = Annual precipitation sum [mm/m2], sp_p = Sum of precipitation [mm/m2] within months 3 to 5, su_p = Sum of precipitation [mm/m2] within months 6 to 8, wi_p = Sum of precipitation [mm/m2] within months 12,1,2, sp_t = Mean temperature [°C] within months 3 to 5, su_t = Mean temperature [°C] within months 6 to 8, wi_t = Mean temperature [°C] within months 12,1,2.

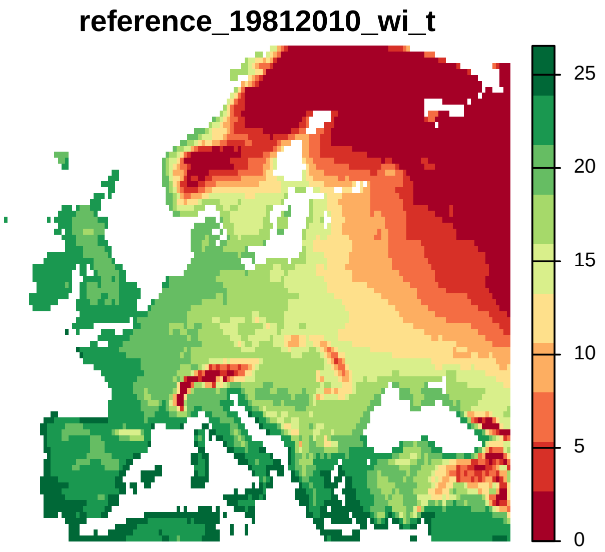

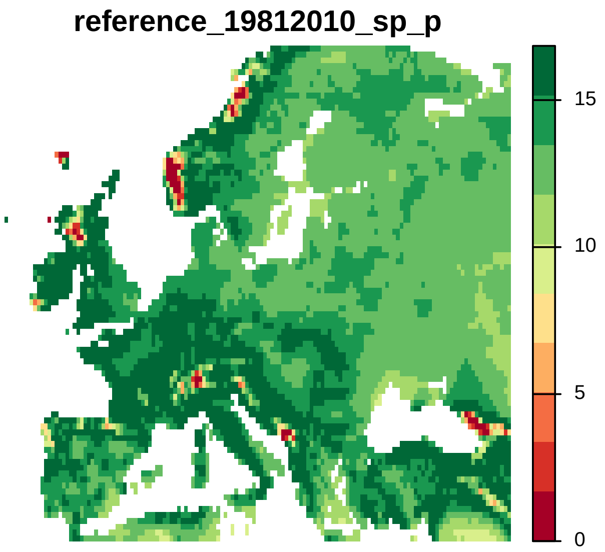

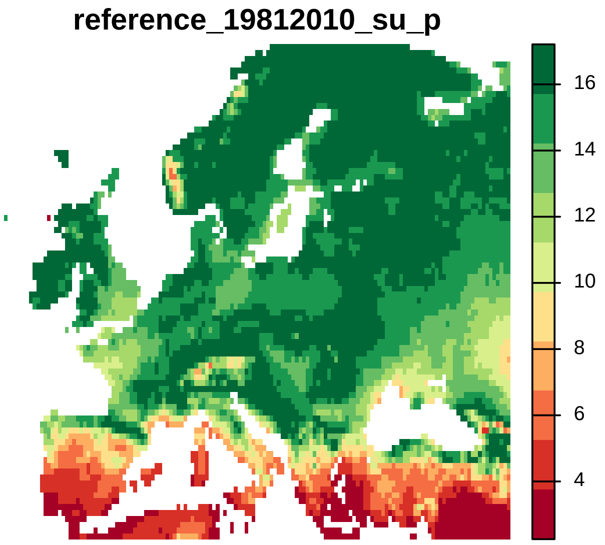

Residual distribution

The multi-panel plot includes a histogram of the residuals (top left), residuals over fitted values (top right), a histogram of observed and predicted values (bottom left) and boxplot diagram of observed and predicted values (bottom right). Observed values are shown in light green, while predicted ones are depicted in light red.

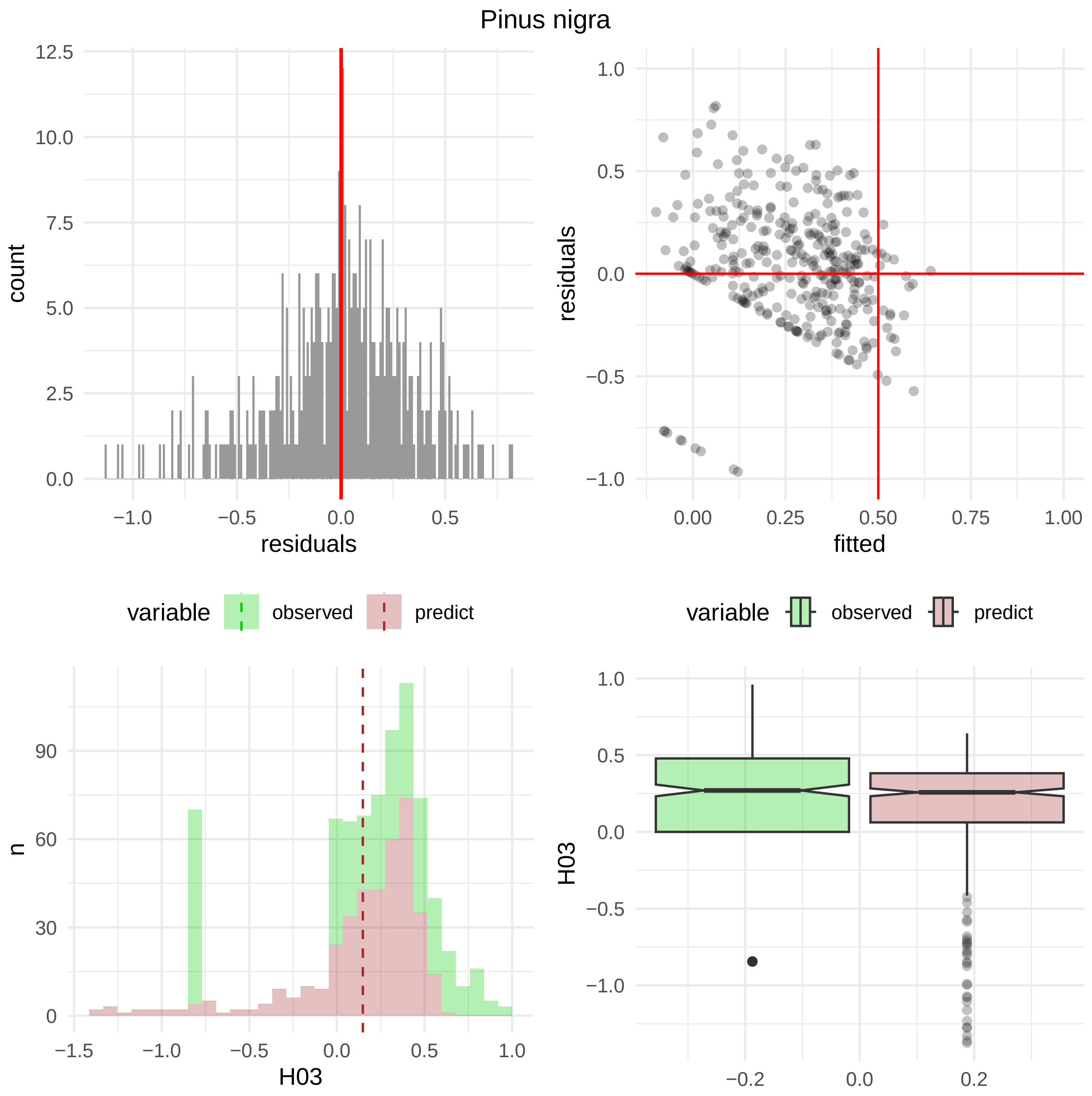

Correlation between predict and observed site index

Relationship between predicted and observed site index (density cloud), as well as linear regressions of presences and absences (= 'growth absences') (red line) and presences only (magenta line). The formulas, significance, R2 and number of observations are displayed below for both regressions. Ideally, both the point cloud and the regression lines lie close to the dashed line. For presences only we additionally calculated the correlation coefficient according to PEARSON (cor.pre) in the bottom right corner.

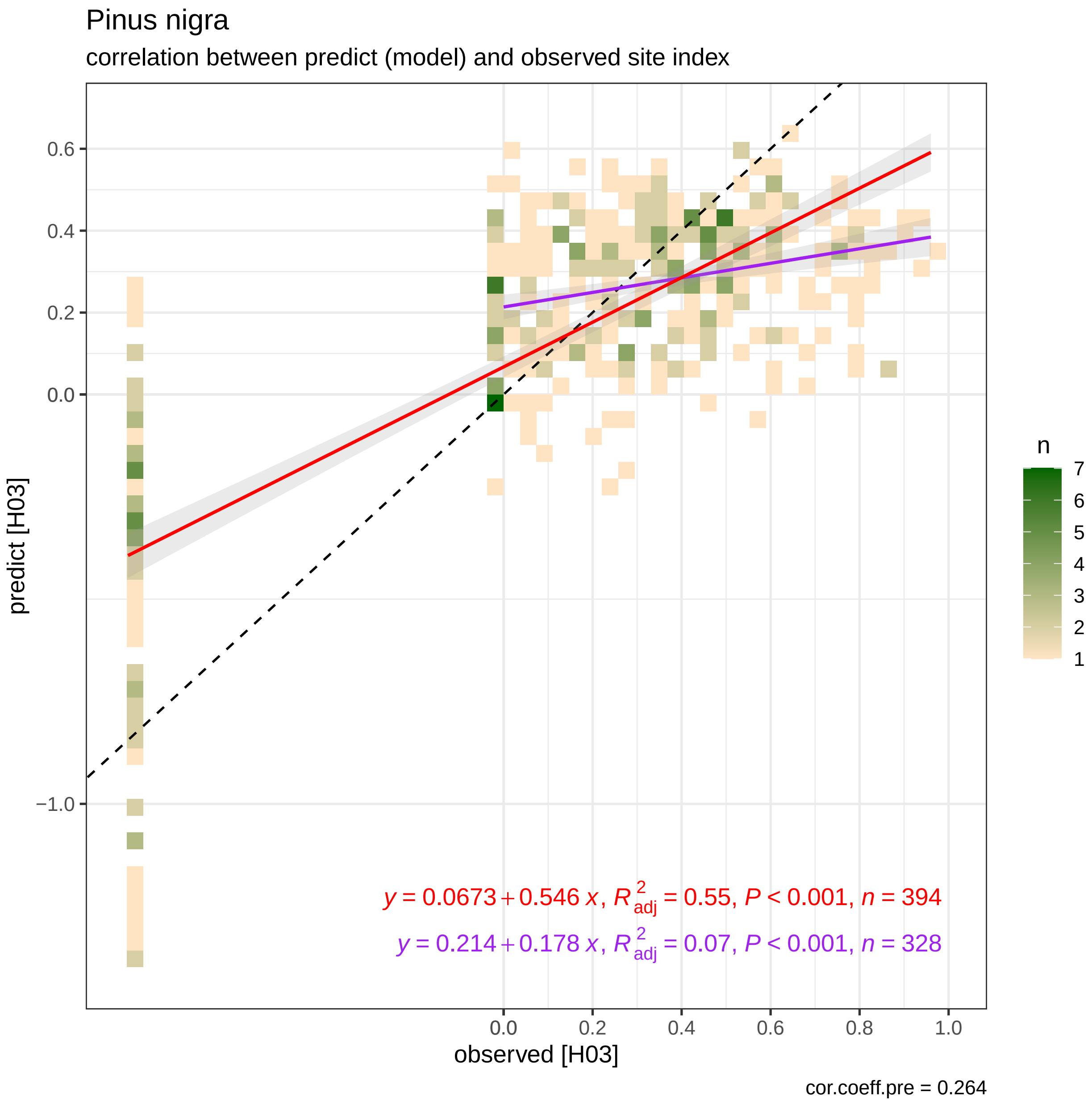

Predictions and forecasts

Predict

European predict for the reference period (1981 to 2010). Dark green symbolizes a high site index (tree height in meters at age 100), orange a lower site index and red no growth. Magenta-coloured dots represent inventory points with growth information, light blue dots are absences (= 'growth absences'). Results were aggregated on 25 km x 25 km scale.

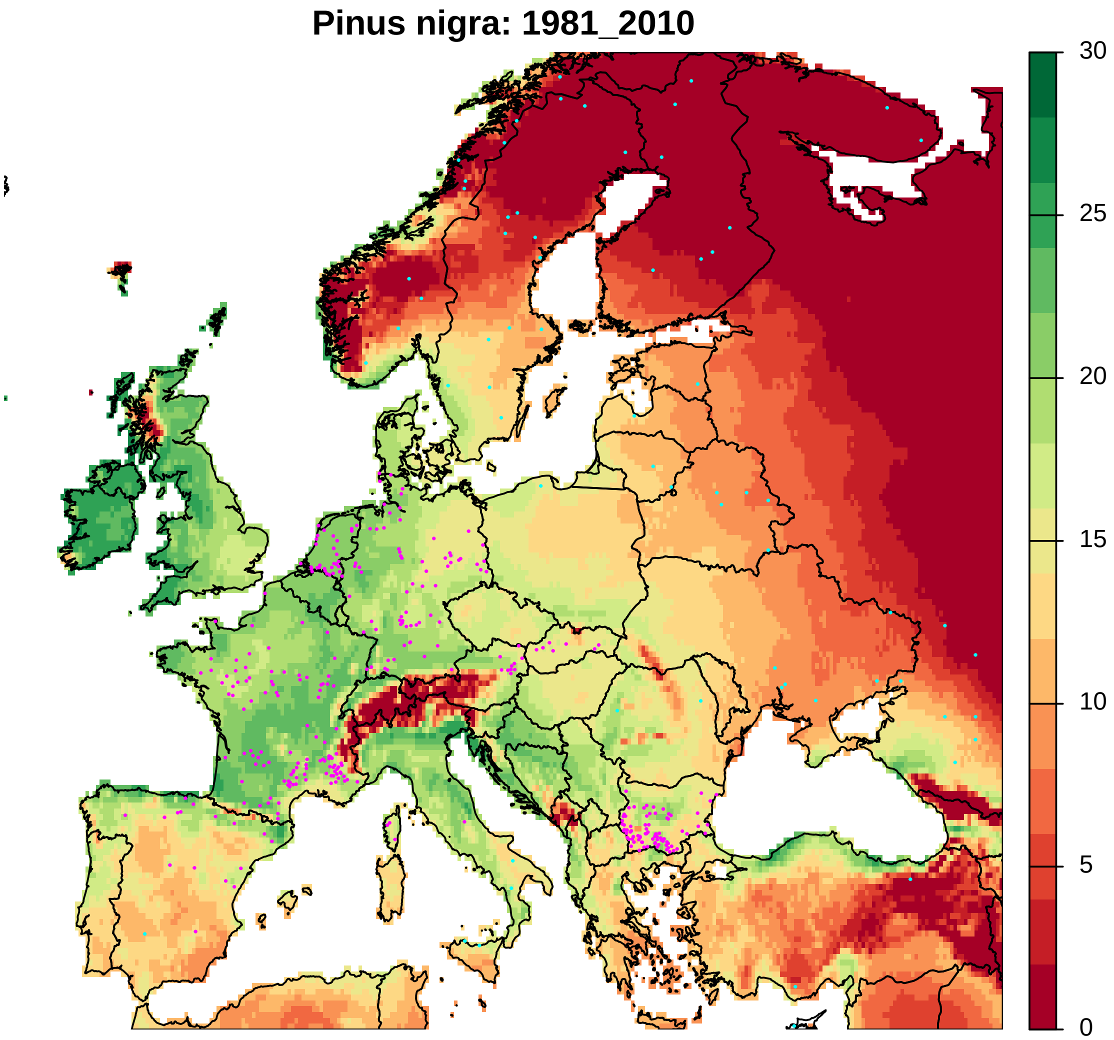

Forecast

Prediction for the reference period (1981 to 2010), as well as forecasts to 2071 to 2100 under szenario RCP4.5 and RCP8.5. Dark green symbolizes a high site index (tree height in m at age 100), orange a lower site index and red no growth. Results were aggregated on 25 km x 25 km scale.

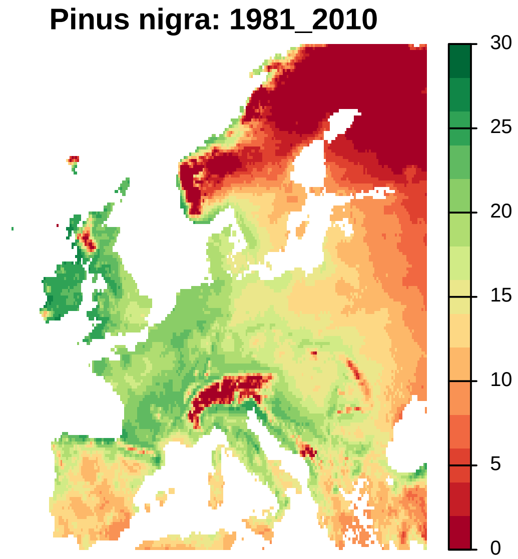

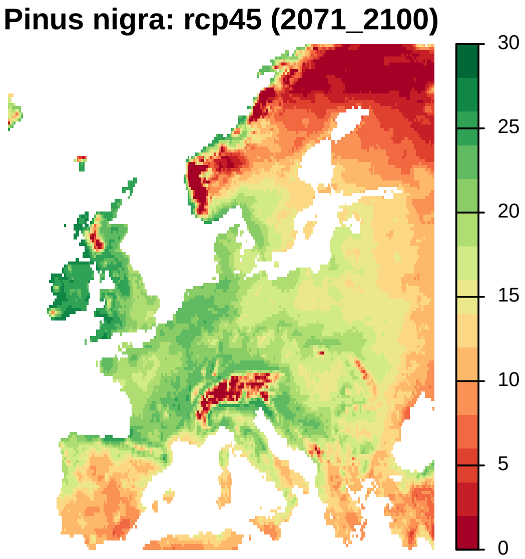

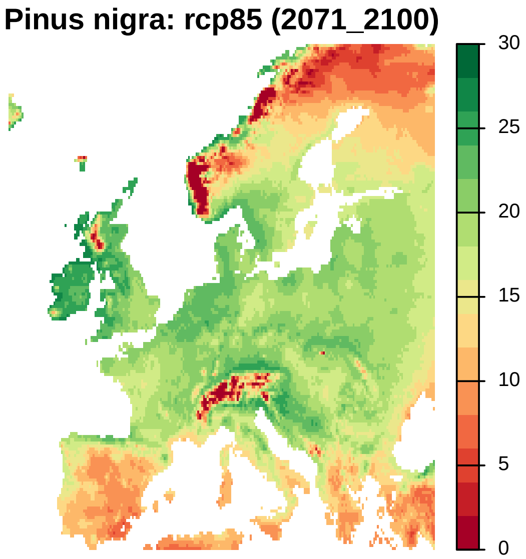

Pinus sylvestris

Site index curves

Site index curves of Pinus sylvestris created with non-linear quantile regressions based on the algorithm of Koenker and Park (1992). The site index (SI) was created by setting all points on the 95 percent quantile (upper line) and above to one (SI = 1) and all on the 5 percent quantile (lower line) and below to zero (SI = 0). The points between the quantile boundaries were assigned a site index between zero and one according to the ratio of their position between the quantile boundaries. We set selected absences (see chapter 2.1.3) on Height = 0 m (at age 100), which means, depending on the site index curves, for each tree species a SI near -1 (red line).

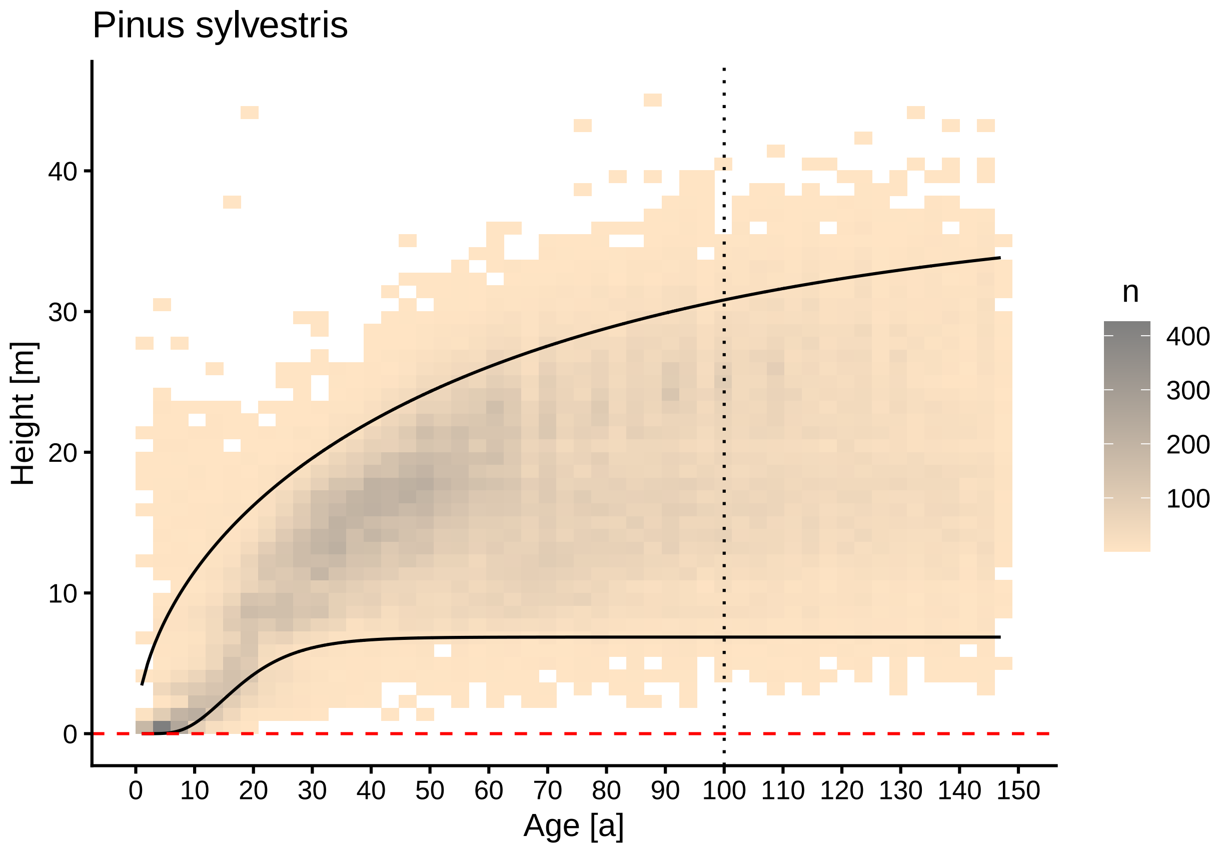

Model statistics and evaluation

Summary

Predictor acronyms: Bio.1 = Mean annual temperature [°C], Bio.12 = Annual precipitation sum [mm/m2], sp_p = Sum of precipitation [mm/m2] within months 3 to 5, su_p = Sum of precipitation [mm/m2] within months 6 to 8, wi_p = Sum of precipitation [mm/m2] within months 12,1,2, sp_t = Mean temperature [°C] within months 3 to 5, su_t = Mean temperature [°C] within months 6 to 8, wi_t = Mean temperature [°C] within months 12,1,2.

```
##
## Family: gaussian
## Link function: identity
##
## Formula:
## H03 ~ s(reference_19812010_su_t, k = 3) + s(reference_19812010_wi_p,
##     k = 3) + s(reference_19812010_su_p, k = 3)
##
## Parametric coefficients:
##             Estimate Std. Error t value Pr(>|t|)
## (Intercept) 0.553034   0.003249   170.2   <2e-16 ***
## ---
## Signif. codes:  0 '***' 0.001 '**' 0.01 '*' 0.05 '.' 0.1 ' ' 1
##
## Approximate significance of smooth terms:
##                              edf Ref.df      F p-value
## s(reference_19812010_su_t) 2.000  2.000 2771.5  <2e-16 ***
## s(reference_19812010_wi_p) 1.945  1.997  159.2  <2e-16 ***
## s(reference_19812010_su_p) 1.958  1.998  154.0  <2e-16 ***
## ---
## Signif. codes:  0 '***' 0.001 '**' 0.01 '*' 0.05 '.' 0.1 ' ' 1
##
## R-sq.(adj) =  0.624   Deviance explained = 62.4%
## -REML = 865.07  Scale est. = 0.074336  n = 7044
```

Variance inflation factor (VIF)

Predictor acronyms: Bio.1 = Mean annual temperature [°C], Bio.12 = Annual precipitation sum [mm/m2], sp_p = Sum of precipitation [mm/m2] within months 3 to 5, su_p = Sum of precipitation [mm/m2] within months 6 to 8, wi_p = Sum of precipitation [mm/m2] within months 12,1,2, sp_t = Mean temperature [°C] within months 3 to 5, su_t = Mean temperature [°C] within months 6 to 8, wi_t = Mean temperature [°C] within months 12,1,2.

```
##                  Variables      VIF
## 1 reference_19812010_su_t 2.009341
## 2 reference_19812010_wi_p 1.274309
## 3 reference_19812010_su_p 2.376633
```

Correlation matrix

Correlation matrix between the predictor variables and the target variable in the model. Correlation coefficient according to PEARSON. Predictor acronyms: Bio.1 = Mean annual temperature [°C], Bio.12 = Annual precipitation sum [mm/m2], sp_p = Sum of precipitation [mm/m2] within months 3 to 5, su_p = Sum of precipitation [mm/m2] within months 6 to 8, wi_p = Sum of precipitation [mm/m2] within months 12,1,2, sp_t = Mean temperature [°C] within months 3 to 5, su_t = Mean temperature [°C] within months 6 to 8, wi_t = Mean temperature [°C] within months 12,1,2.

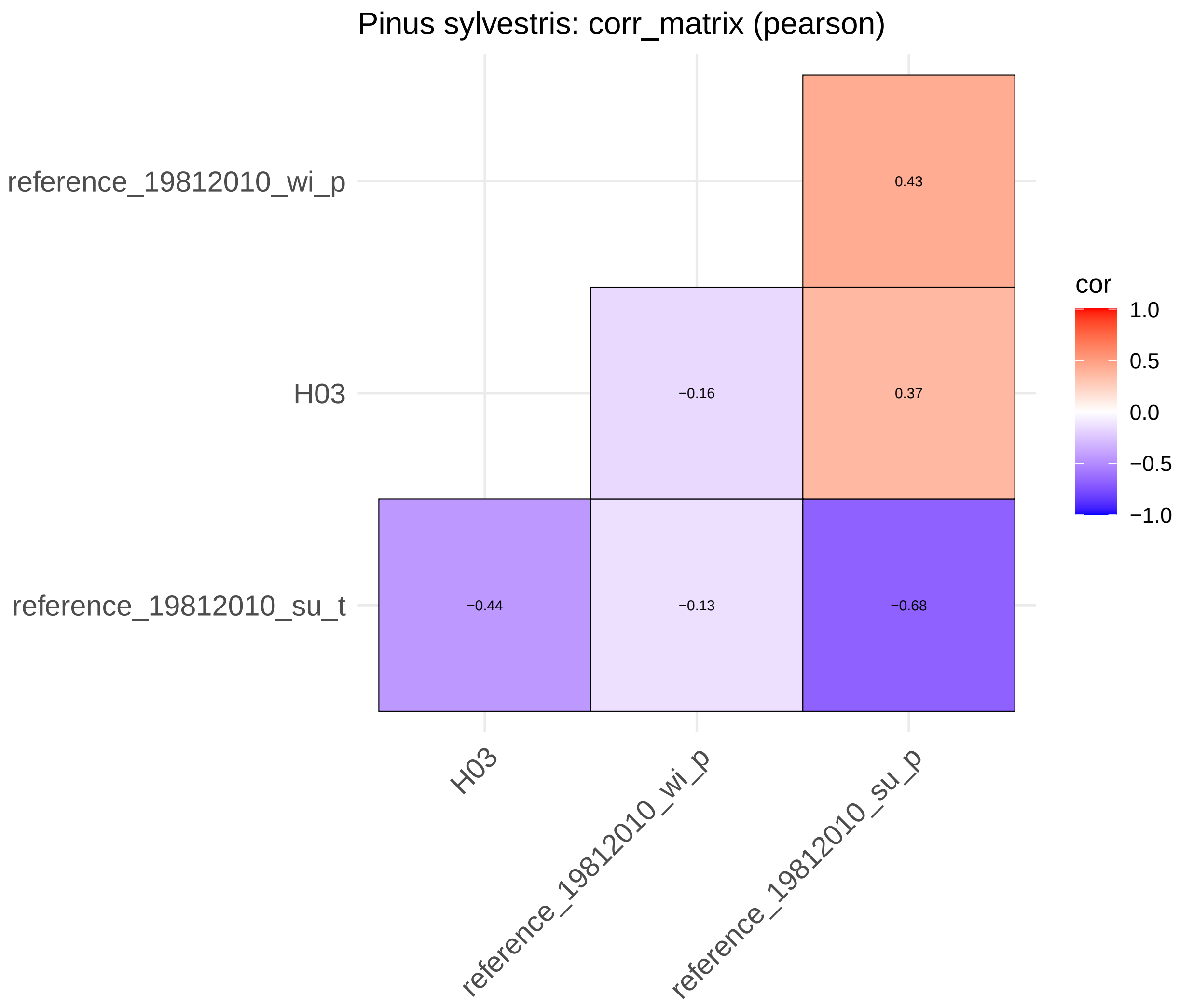

Response curves

Response curves (also known as effect curves) show how each predictor variable affects the target variable (H03 = european Site index, SIrel). H03 values below zero represent 'Growth absences'. Predictor acronyms: Bio.1 = Mean annual temperature [°C], Bio.12 = Annual precipitation sum [mm/m2], sp_p = Sum of precipitation [mm/m2] within months 3 to 5, su_p = Sum of precipitation [mm/m2] within months 6 to 8, wi_p = Sum of precipitation [mm/m2] within months 12,1,2, sp_t = Mean temperature [°C] within months 3 to 5, su_t = Mean temperature [°C] within months 6 to 8, wi_t = Mean temperature [°C] within months 12,1,2.

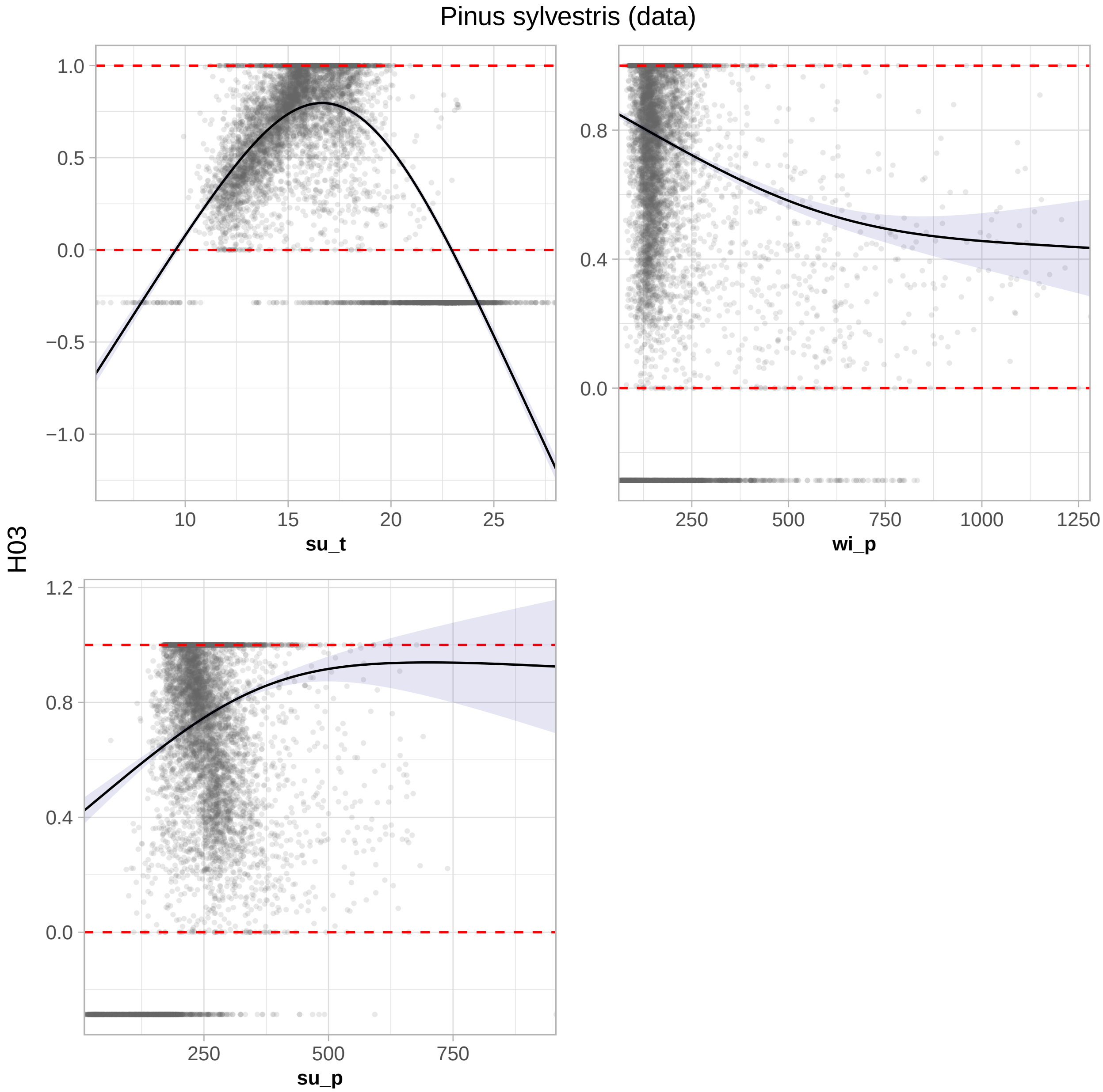

Response maps

Response maps (also referred as partial effect maps). Each map visualizes how a predictor affect the target variable (top height [m] at Age 100). Technically their work like response curves in a geographical area, that is setting all predictor variables except the one shown in the figure on their mean, and mapping the prediction. Predictor acronyms: Bio.1 = Mean annual temperature [°C], Bio.12 = Annual precipitation sum [mm/m2], sp_p = Sum of precipitation [mm/m2] within months 3 to 5, su_p = Sum of precipitation [mm/m2] within months 6 to 8, wi_p = Sum of precipitation [mm/m2] within months 12,1,2, sp_t = Mean temperature [°C] within months 3 to 5, su_t = Mean temperature [°C] within months 6 to 8, wi_t = Mean temperature [°C] within months 12,1,2.

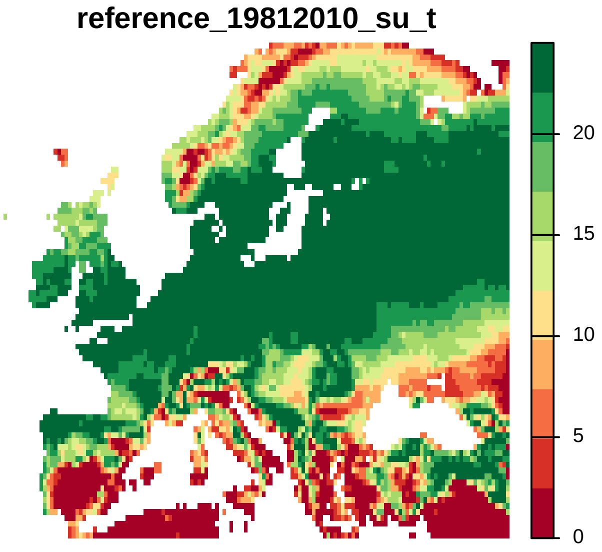

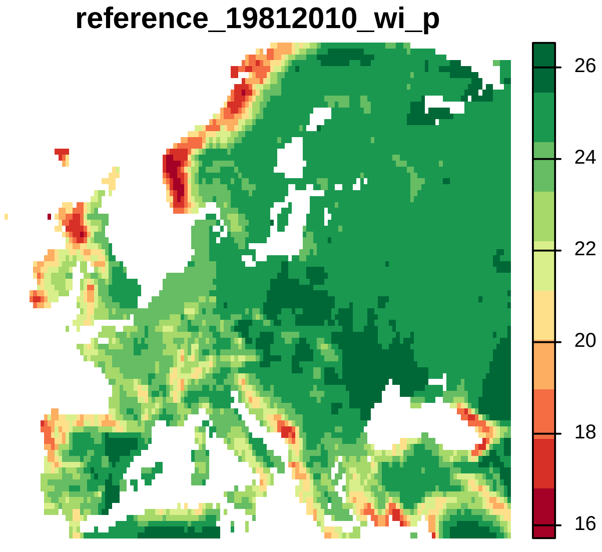

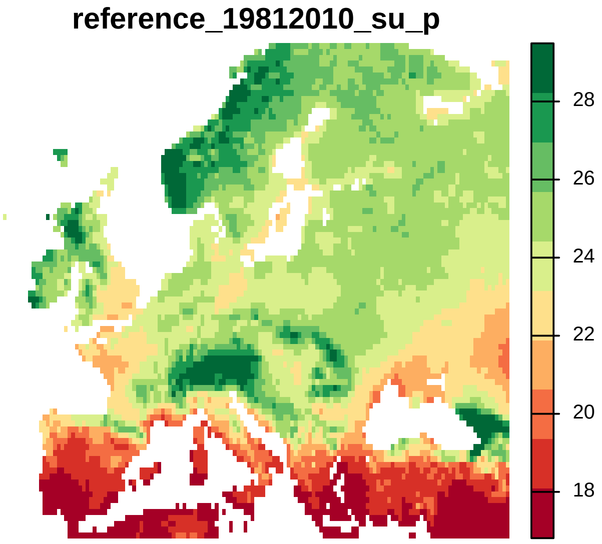

Residual distribution

The multi-panel plot includes a histogram of the residuals (top left), residuals over fitted values (top right), a histogram of observed and predicted values (bottom left) and boxplot diagram of observed and predicted values (bottom right). Observed values are shown in light green, while predicted ones are depicted in light red.

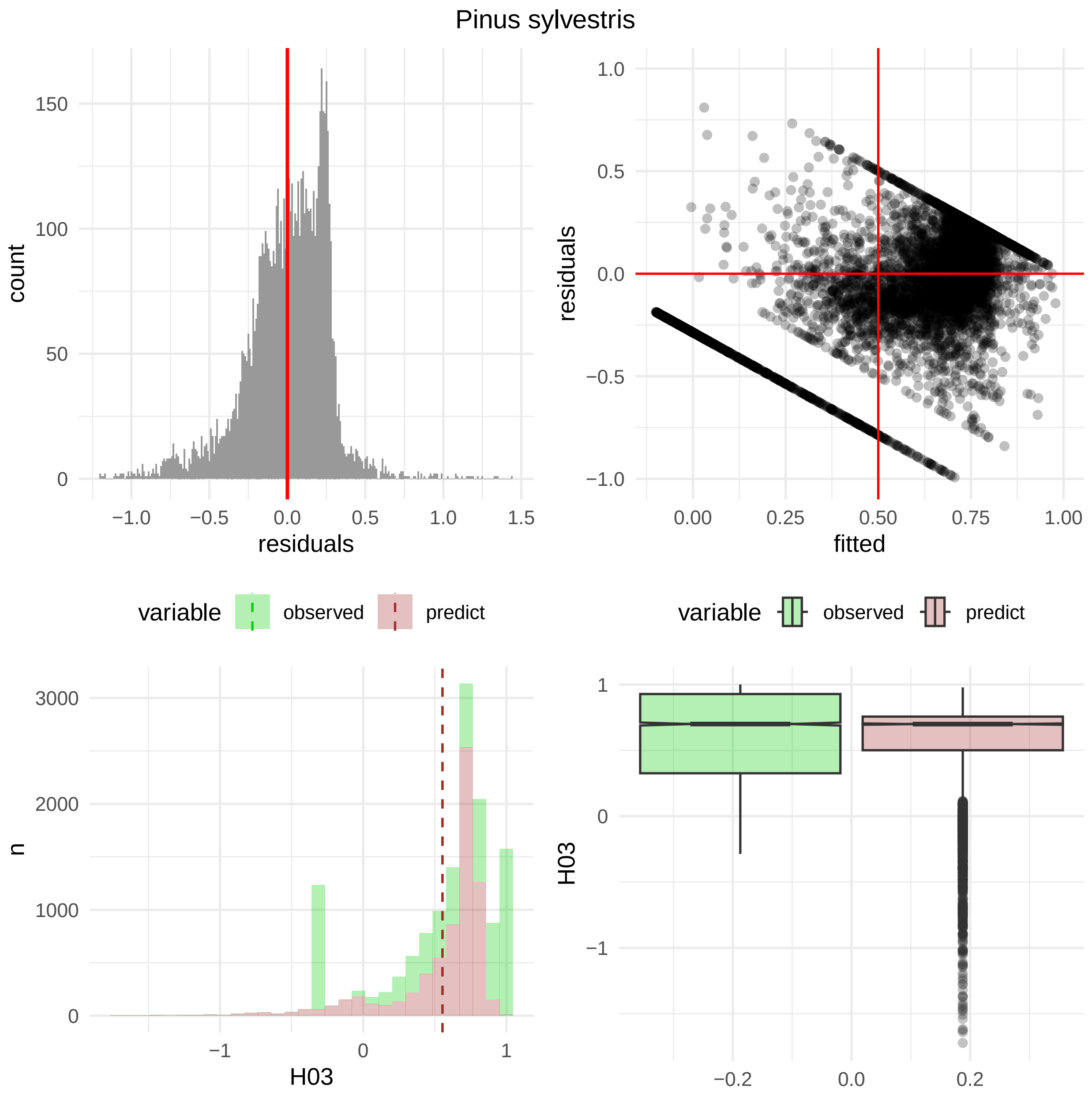

Correlation between predict and observed site index

Relationship between predicted and observed site index (density cloud), as well as linear regressions of presences and absences (= 'growth absences') (red line) and presences only (magenta line). The formulas, significance, R2 and number of observations are displayed below for both regressions. Ideally, both the point cloud and the regression lines lie close to the dashed line. For presences only we additionally calculated the correlation coefficient according to PEARSON (cor.pre) in the bottom right corner.

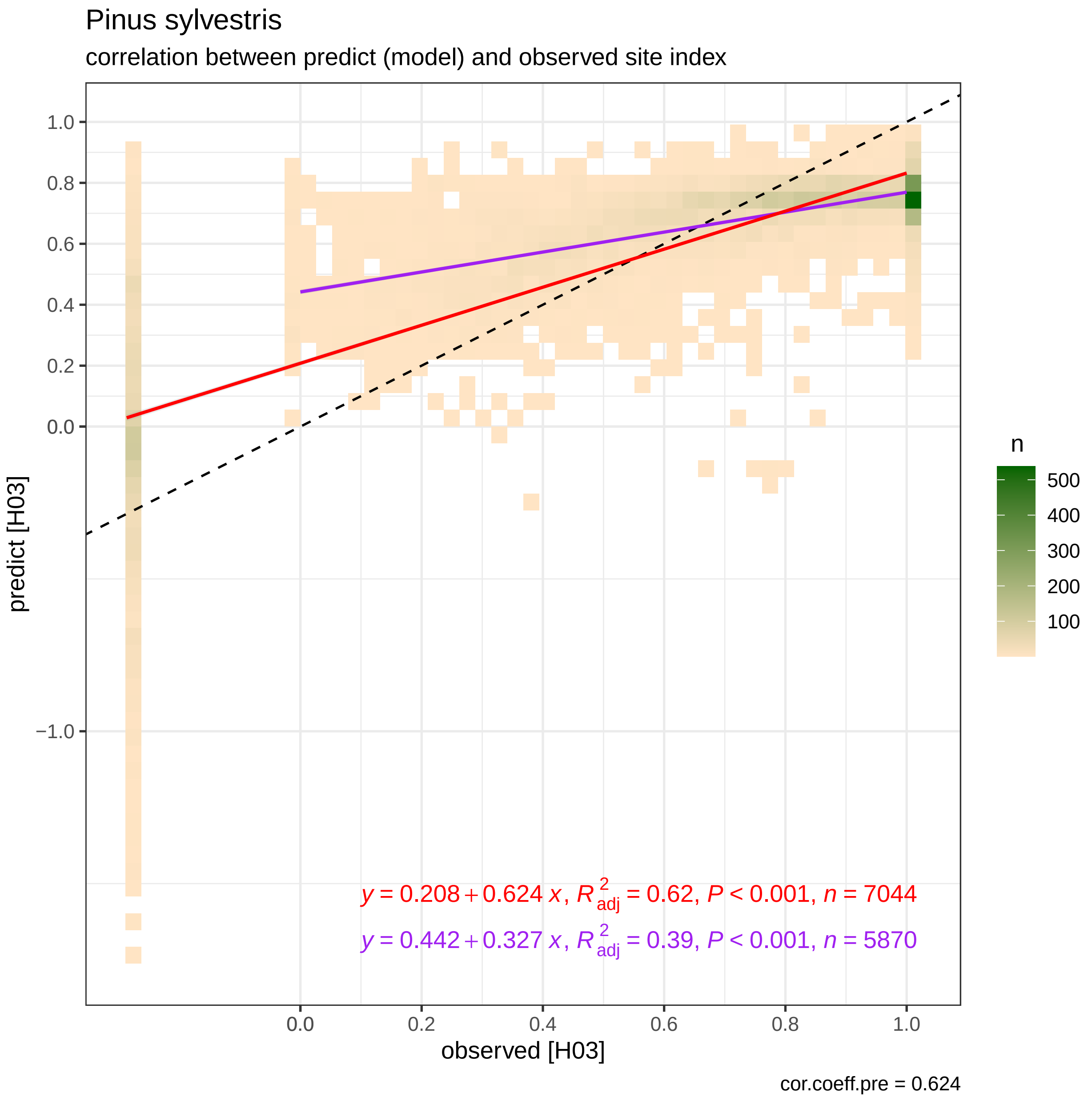

Predictions and forecasts

Predict

European predict for the reference period (1981 to 2010). Dark green symbolizes a high site index (tree height in meters at age 100), orange a lower site index and red no growth. Magenta-coloured dots represent inventory points with growth information, light blue dots are absences (= ‘growth absences’). Results were aggregated on 25 km x 25 km scale.

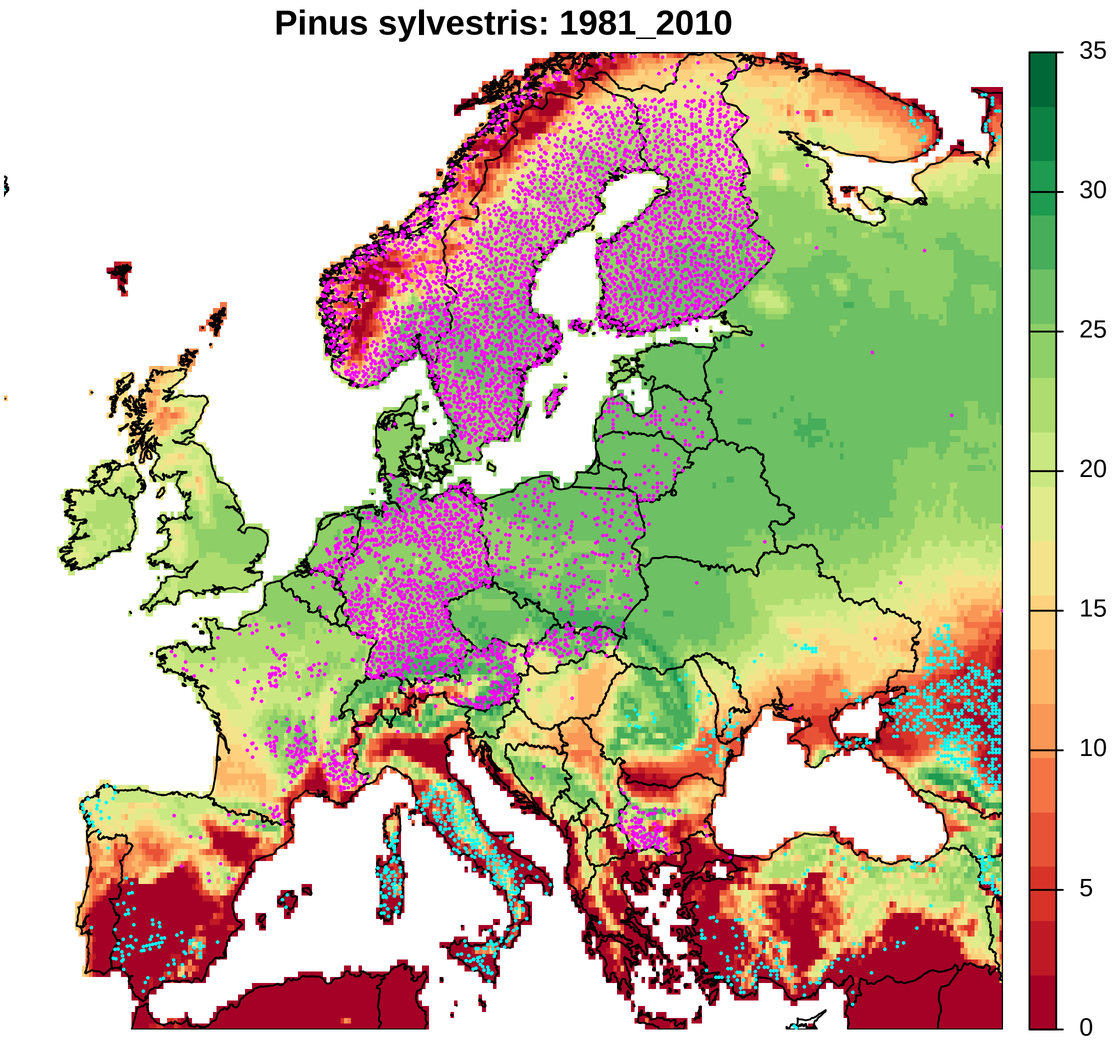

Forecast

Prediction for the reference period (1981 to 2010), as well as forecasts to 2071 to 2100 under szenario RCP4.5 and RCP8.5. Dark green symbolizes a high site index (tree height in m at age 100), orange a lower site index and red no growth. Results were aggregated on 25 km x 25 km scale.

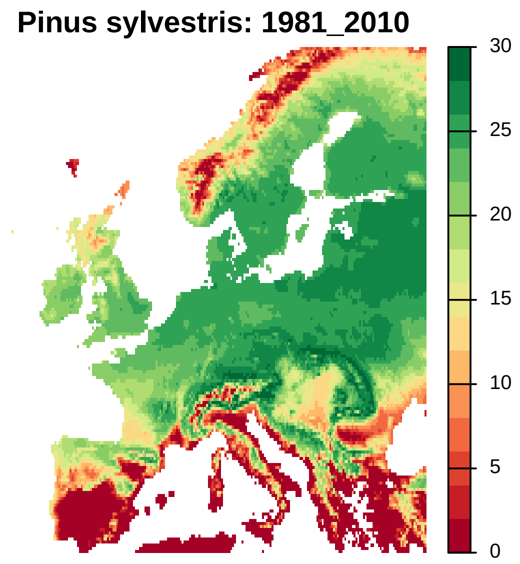

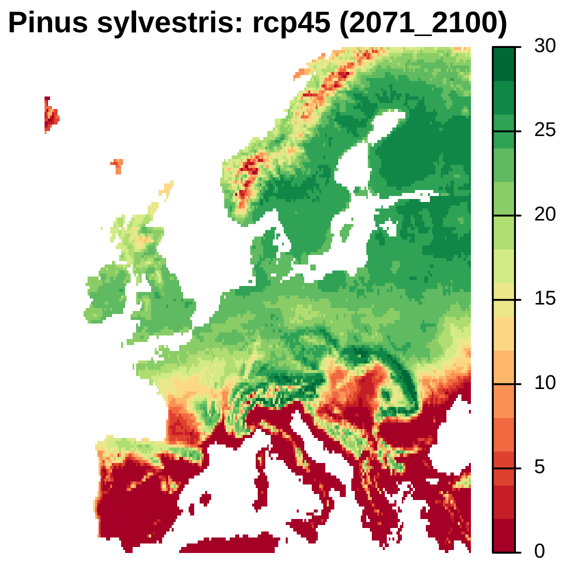

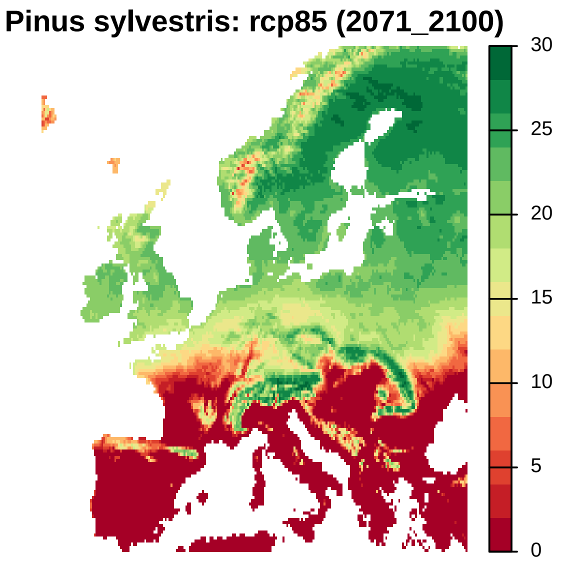

Prunus avium

Site index curves

Site index curves of Prunus avium created with non-linear quantile regressions based on the algorithm of Koenker and Park (1992). The site index (SI) was created by setting all points on the 95 percent quantile (upper line) and above to one (SI = 1) and all on the 5 percent quantile (lower line) and below to zero (SI = 0). The points between the quantile boundaries were assigned a site index between zero and one according to the ratio of their position between the quantile boundaries. We set selected absences (see chapter 2.1.3) on Height = 0 m (at age 100), which means, depending on the site index curves, for each tree species a SI near -1 (red line).

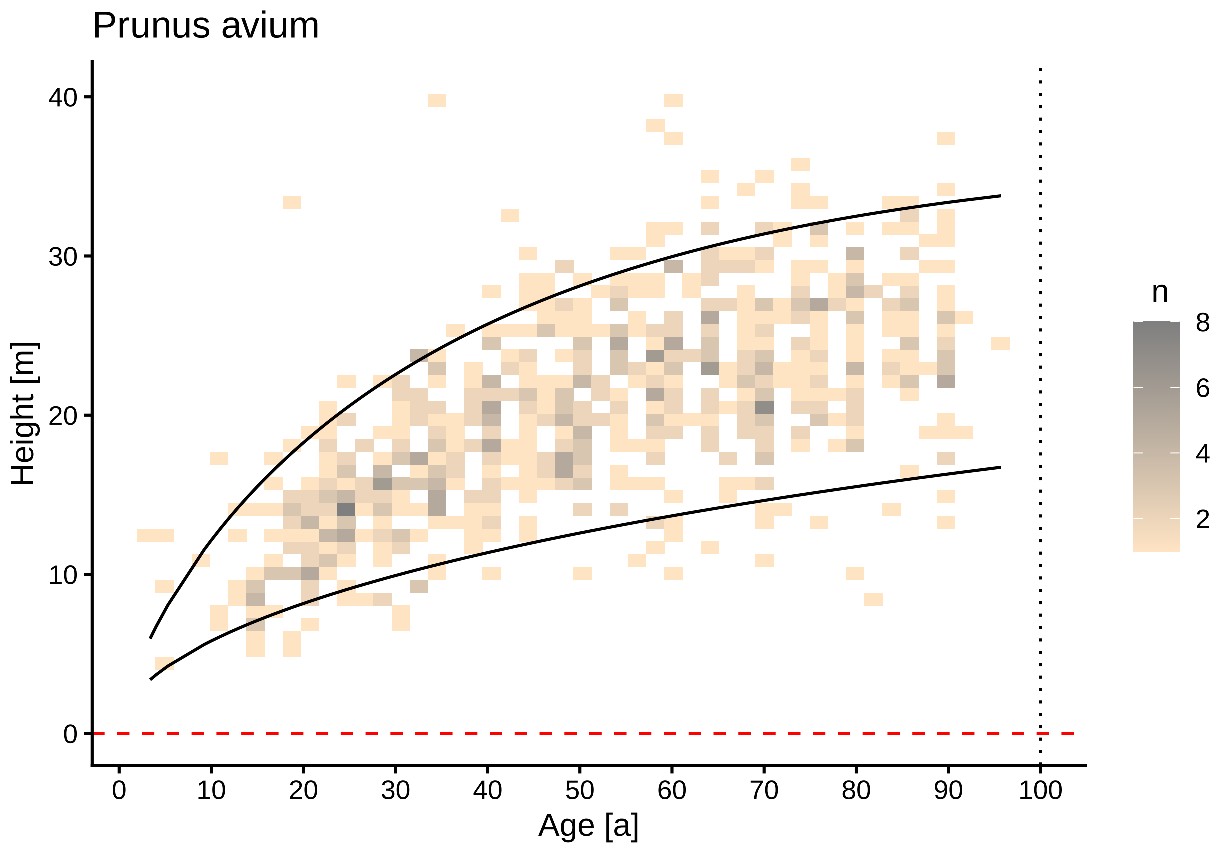

Model statistics and evaluation

Summary

Predictor acronyms: Bio.1 = Mean annual temperature [°C], Bio.12 = Annual precipitation sum [mm/m2], sp_p = Sum of precipitation [mm/m2] within months 3 to 5, su_p = Sum of precipitation [mm/m2] within months 6 to 8, wi_p = Sum of precipitation [mm/m2] within months 12,1,2, sp_t = Mean temperature [°C] within months 3 to 5, su_t = Mean temperature [°C] within months 6 to 8, wi_t = Mean temperature [°C] within months 12,1,2.

```
##
## Family: gaussian
## Link function: identity
##
## Formula:
## H03 ~ s(reference_19812010_su_t, k = 3) + s(reference_19812010_wi_t,
##     k = 3) + s(reference_19812010_sp_p, k = 3)
##
## Parametric coefficients:
##             Estimate Std. Error t value Pr(>|t|)
## (Intercept)  0.31814    0.01535   20.73   <2e-16 ***
## ---
## Signif. codes:  0 '***' 0.001 '**' 0.01 '*' 0.05 '.' 0.1 ' ' 1
##
## Approximate significance of smooth terms:
##                              edf Ref.df      F p-value
## s(reference_19812010_su_t) 1.988  2.000 126.31  <2e-16 ***
## s(reference_19812010_wi_t) 1.982  2.000 100.51  <2e-16 ***
## s(reference_19812010_sp_p) 1.977  1.999  27.12  <2e-16 ***
## ---
## Signif. codes:  0 '***' 0.001 '**' 0.01 '*' 0.05 '.' 0.1 ' ' 1
##
## R-sq.(adj) =  0.661   Deviance explained = 66.5%
## -REML = 275.05  Scale est. = 0.1397    n = 593
```

Variance inflation factor (VIF)

Predictor acronyms: Bio.1 = Mean annual temperature [°C], Bio.12 = Annual precipitation sum [mm/m2], sp_p = Sum of precipitation [mm/m2] within months 3 to 5, su_p = Sum of precipitation [mm/m2] within months 6 to 8, wi_p = Sum of precipitation [mm/m2] within months 12,1,2, sp_t = Mean temperature [°C] within months 3 to 5, su_t = Mean temperature [°C] within months 6 to 8, wi_t = Mean temperature [°C] within months 12,1,2.

```
##                  Variables      VIF
## 1 reference_19812010_su_t 1.521451
## 2 reference_19812010_wi_t 1.514800
## 3 reference_19812010_sp_p 1.140168
```

Correlation matrix

Correlation matrix between the predictor variables and the target variable in the model. Correlation coefficient according to PEARSON. Predictor acronyms: Bio.1 = Mean annual temperature [°C], Bio.12 = Annual precipitation sum [mm/m2], sp_p = Sum of precipitation [mm/m2] within months 3 to 5, su_p = Sum of precipitation [mm/m2] within months 6 to 8, wi_p = Sum of precipitation [mm/m2] within months 12,1,2, sp_t = Mean temperature [°C] within months 3 to 5, su_t = Mean temperature [°C] within months 6 to 8, wi_t = Mean temperature [°C] within months 12,1,2.

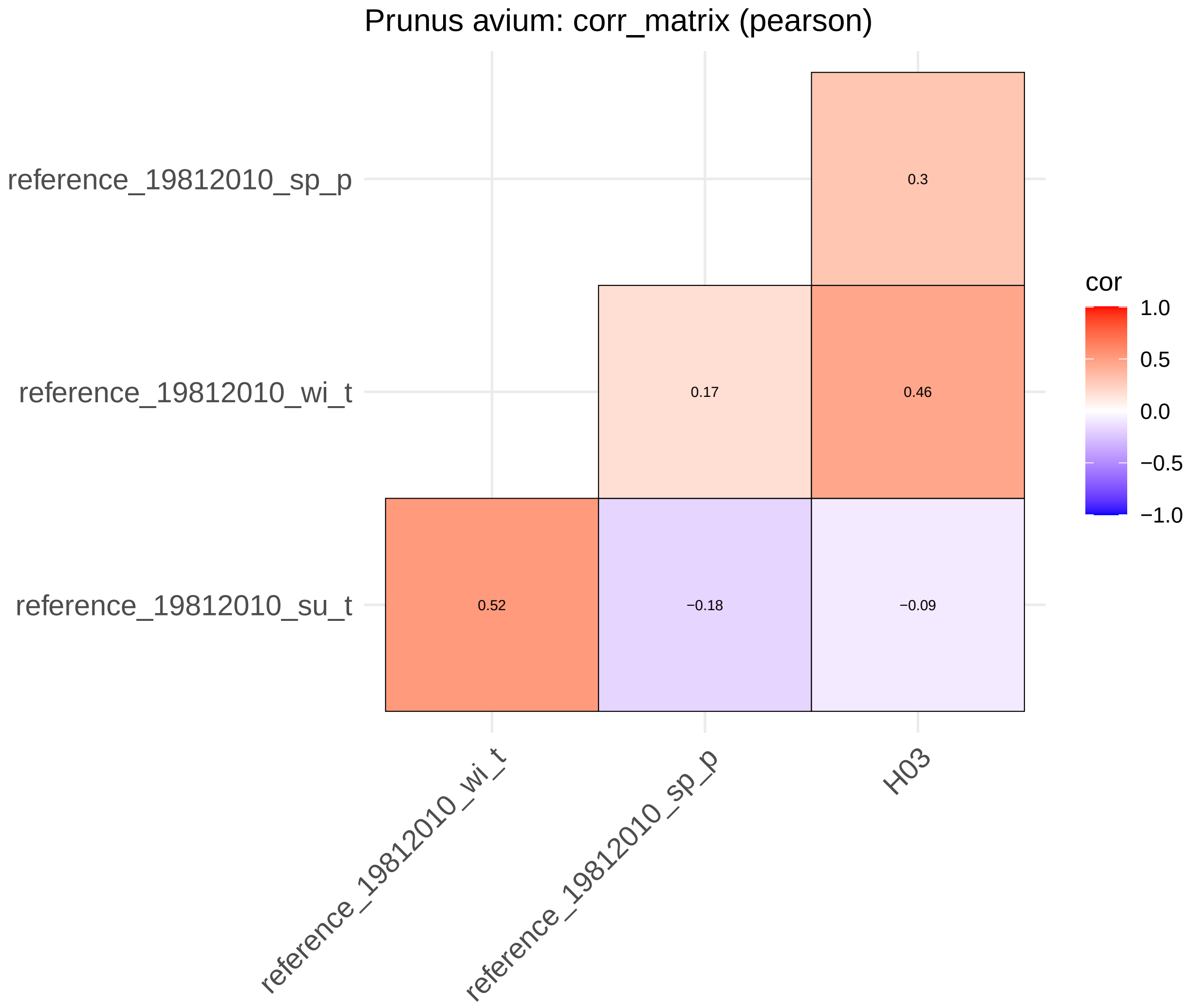

Response curves

Response curves (also known as effect curves) show how each predictor variable affects the target variable (H03 = european Site index, SIrel). H03 values below zero represent 'Growth absences'. Predictor acronyms: Bio.1 = Mean annual temperature [°C], Bio.12 = Annual precipitation sum [mm/m2], sp_p = Sum of precipitation [mm/m2] within months 3 to 5, su_p = Sum of precipitation [mm/m2] within months 6 to 8, wi_p = Sum of precipitation [mm/m2] within months 12,1,2, sp_t = Mean temperature [°C] within months 3 to 5, su_t = Mean temperature [°C] within months 6 to 8, wi_t = Mean temperature [°C] within months 12,1,2.

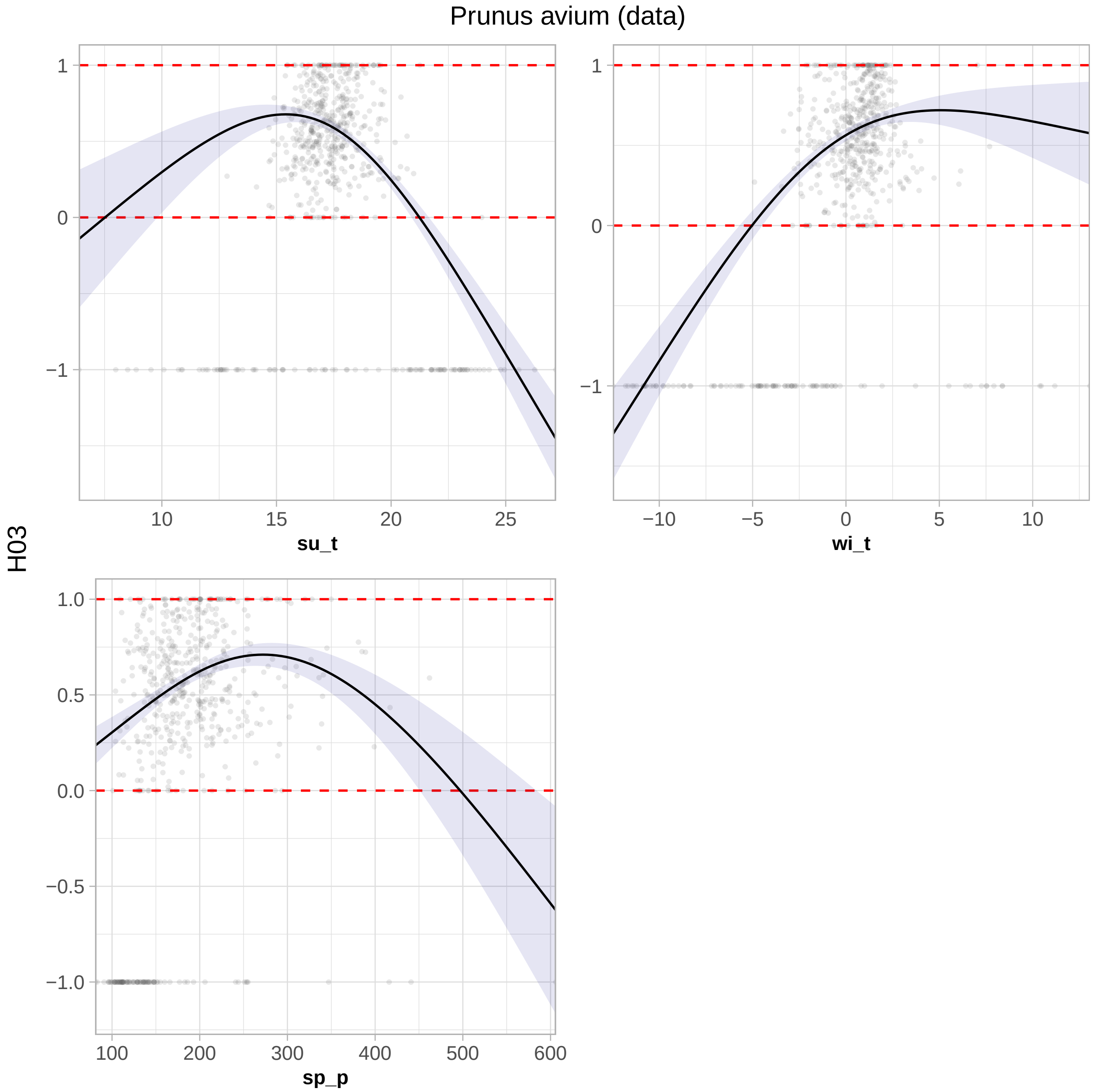

Response maps

Response maps (also referred as partial effect maps). Each map visualizes how a predictor affect the target variable (top height [m] at Age 100). Technically their work like response curves in a geographical area, that is setting all predictor variables except the one shown in the figure on their mean, and mapping the prediction. Predictor acronyms: Bio.1 = Mean annual temperature [°C], Bio.12 = Annual precipitation sum [mm/m2], sp_p = Sum of precipitation [mm/m2] within months 3 to 5, su_p = Sum of precipitation [mm/m2] within months 6 to 8, wi_p = Sum of precipitation [mm/m2] within months 12,1,2, sp_t = Mean temperature [°C] within months 3 to 5, su_t = Mean temperature [°C] within months 6 to 8, wi_t = Mean temperature [°C] within months 12,1,2.

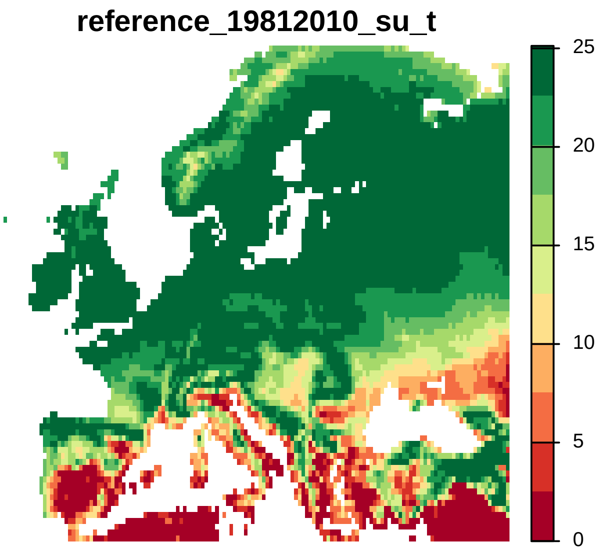

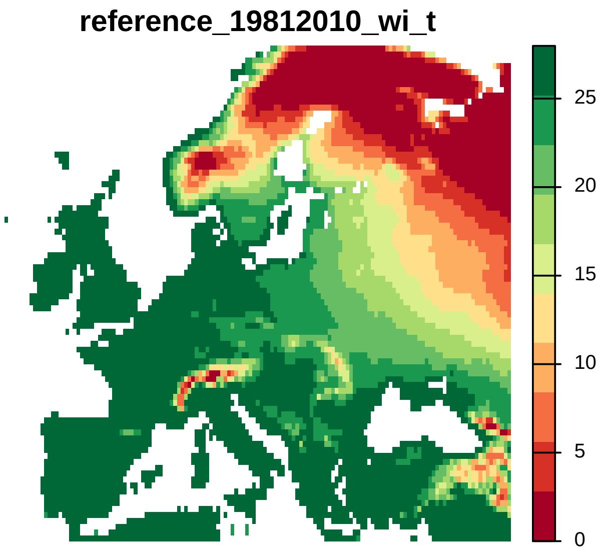

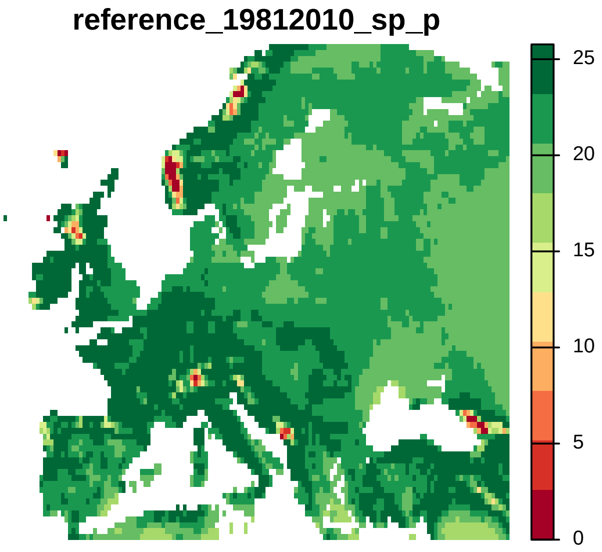

Residual distribution

The multi-panel plot includes a histogram of the residuals (top left), residuals over fitted values (top right), a histogram of observed and predicted values (bottom left) and boxplot diagram of observed and predicted values (bottom right). Observed values are shown in light green, while predicted ones are depicted in light red.

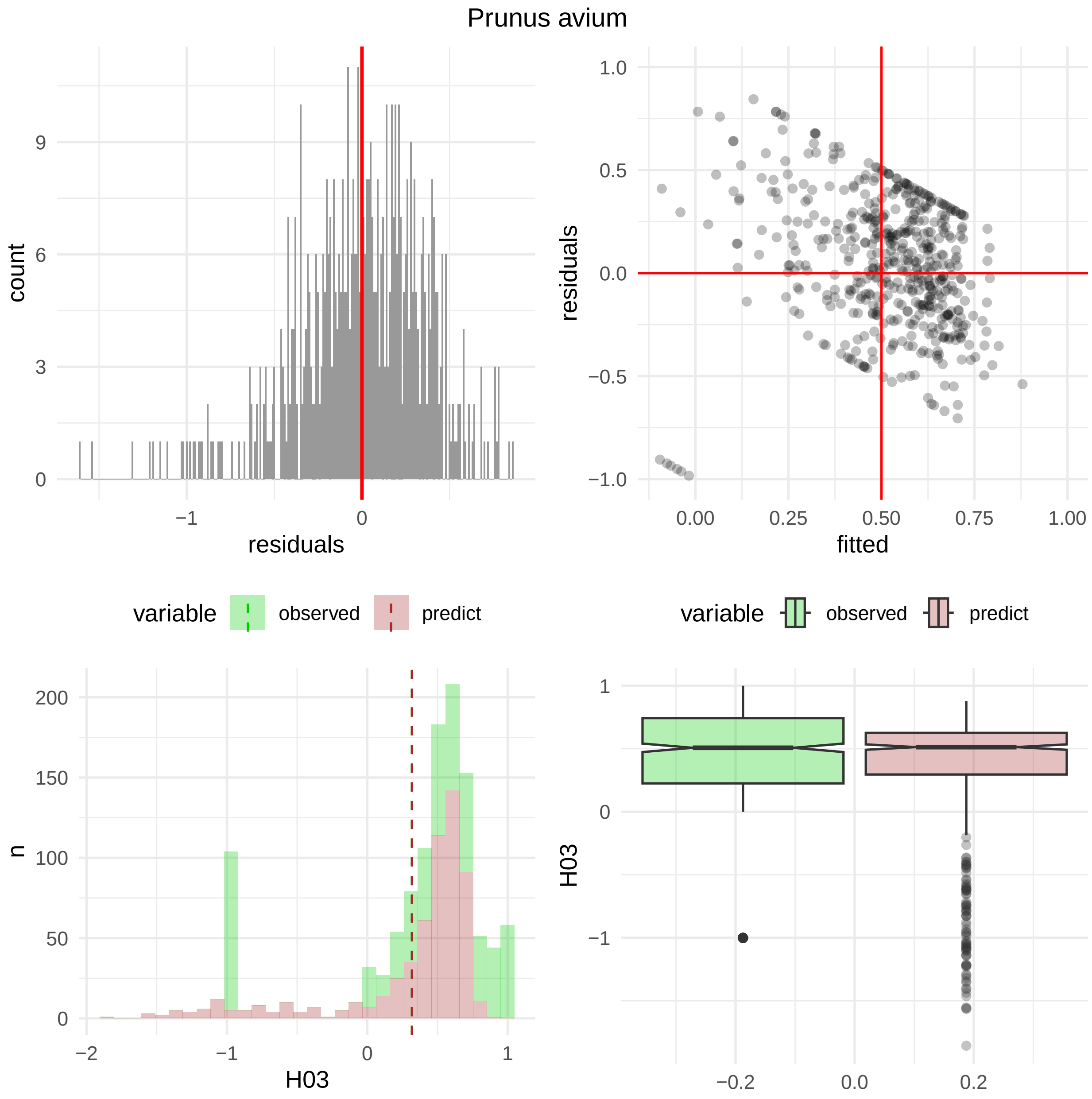

Correlation between predict and observed site index

Relationship between predicted and observed site index (density cloud), as well as linear regressions of presences and absences (= ‘growth absences’) (red line) and presences only (magenta line). The formulas, significance, R2 and number of observations are displayed below for both regressions. Ideally, both the point cloud and the regression lines lie close to the dashed line. For presences only we additionally calculated the correlation coefficient according to PEARSON (cor.pre) in the bottom right corner.

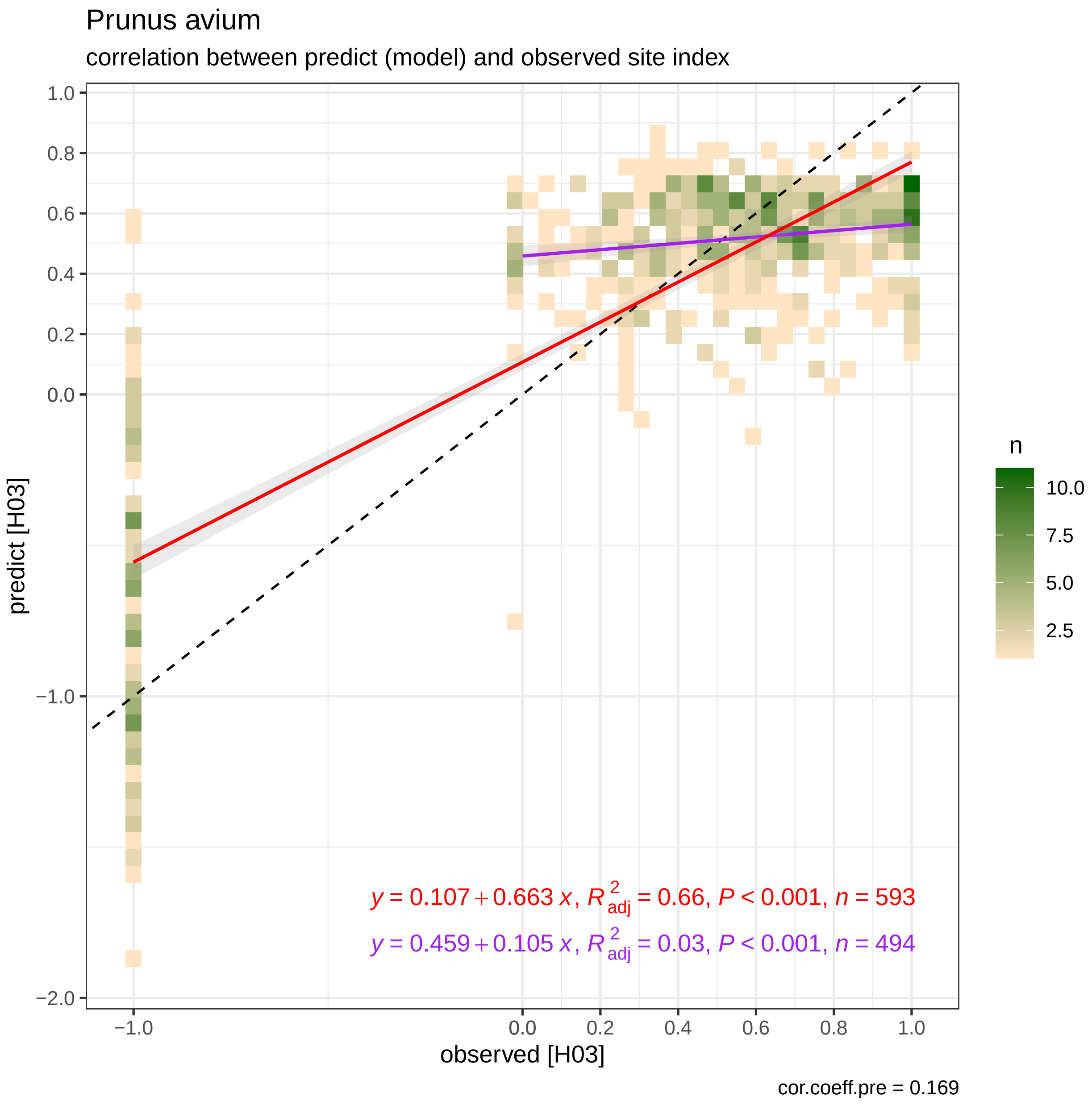

Predictions and forecasts

Predict

European predict for the reference period (1981 to 2010). Dark green symbolizes a high site index (tree height in meters at age 100), orange a lower site index and red no growth. Magenta-coloured dots represent inventory points with growth information, light blue dots are absences (= 'growth absences'). Results were aggregated on 25 km x 25 km scale.

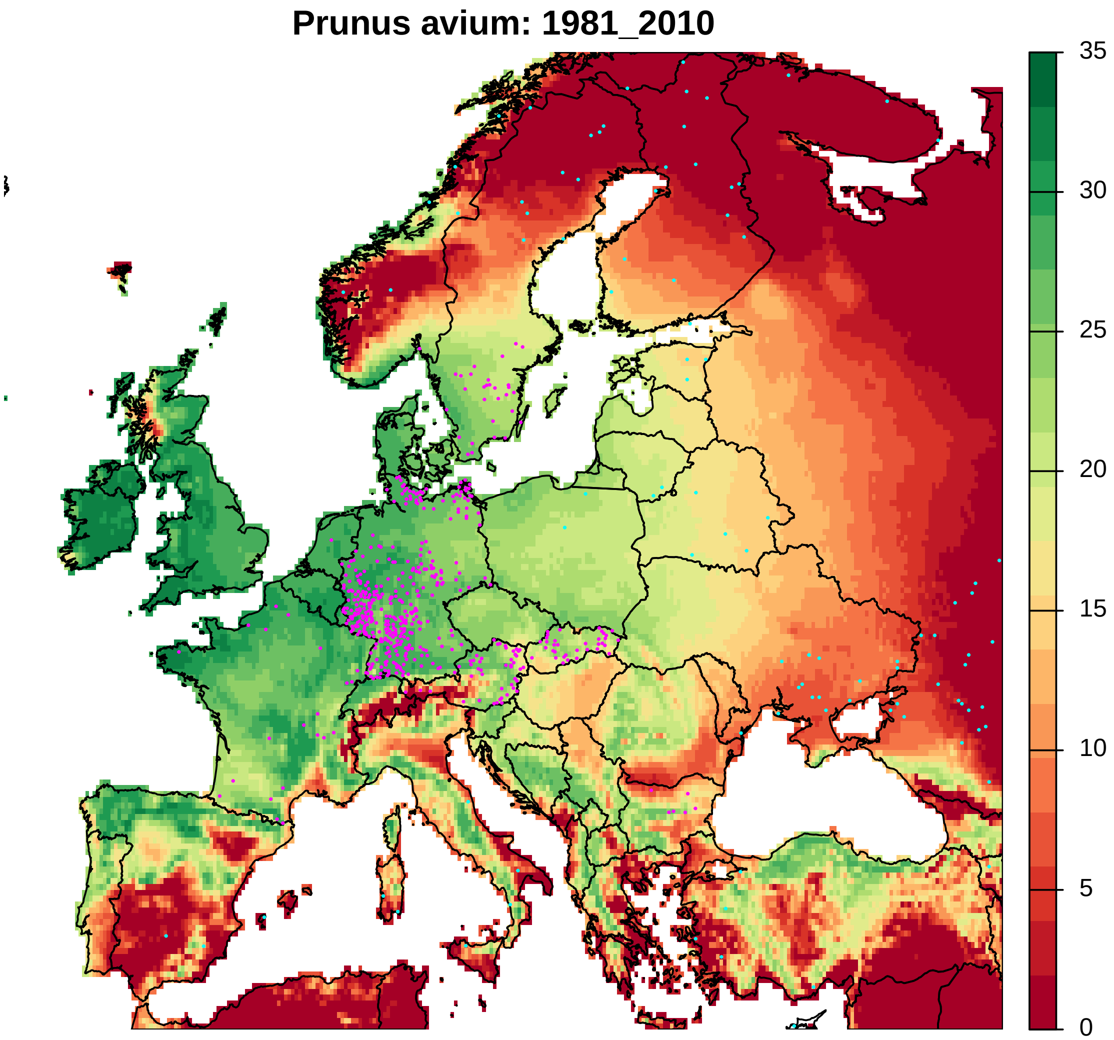

Forecast

Prediction for the reference period (1981 to 2010), as well as forecasts to 2071 to 2100 under szenario RCP4.5 and RCP8.5. Dark green symbolizes a high site index (tree height in m at age 100), orange a lower site index and red no growth. Results were aggregated on 25 km x 25 km scale.

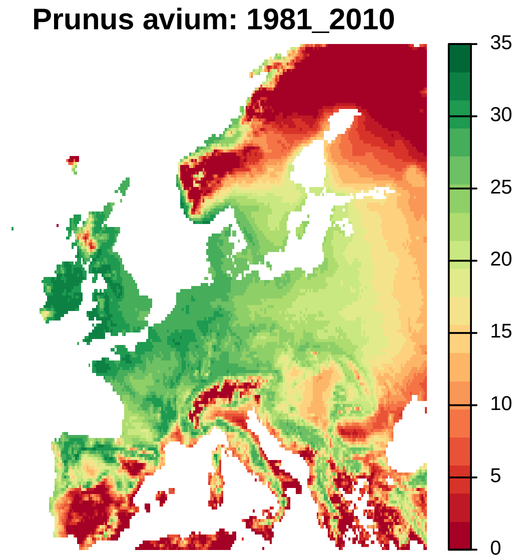

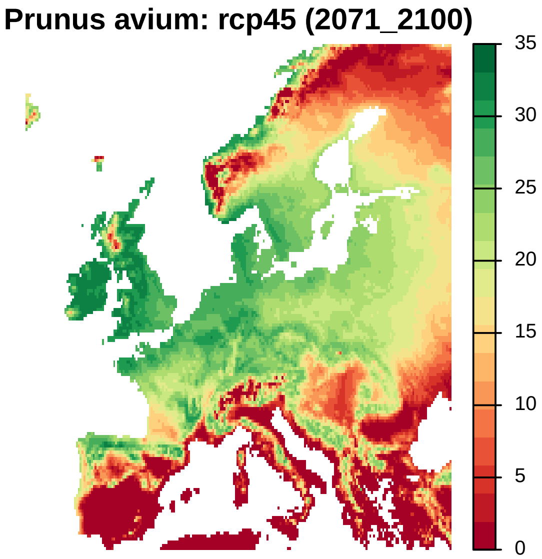

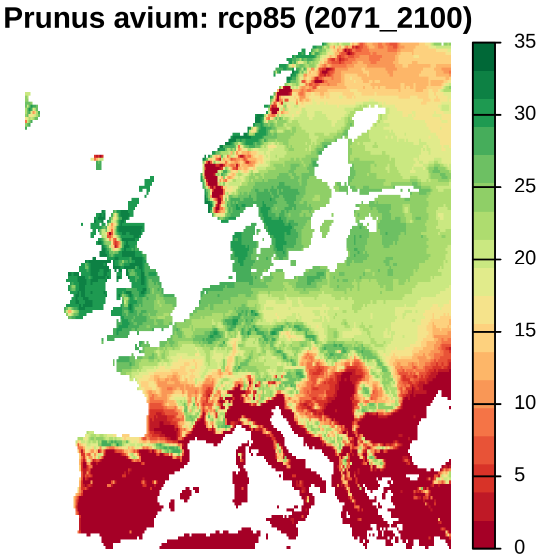

Pseudotsuga menziesii

Site index curves

Site index curves of Pseudotsuga menziesii created with non-linear quantile regressions based on the algorithm of Koenker and Park (1992). The site index (SI) was created by setting all points on the 95 percent quantile (upper line) and above to one (SI = 1) and all on the 5 percent quantile (lower line) and below to zero (SI = 0). The points between the quantile boundaries were assigned a site index between zero and one according to the ratio of their position between the quantile boundaries. We set selected absences (see chapter 2.1.3) on Height = 0 m (at age 100), which means, depending on the site index curves, for each tree species a SI near -1 (red line).

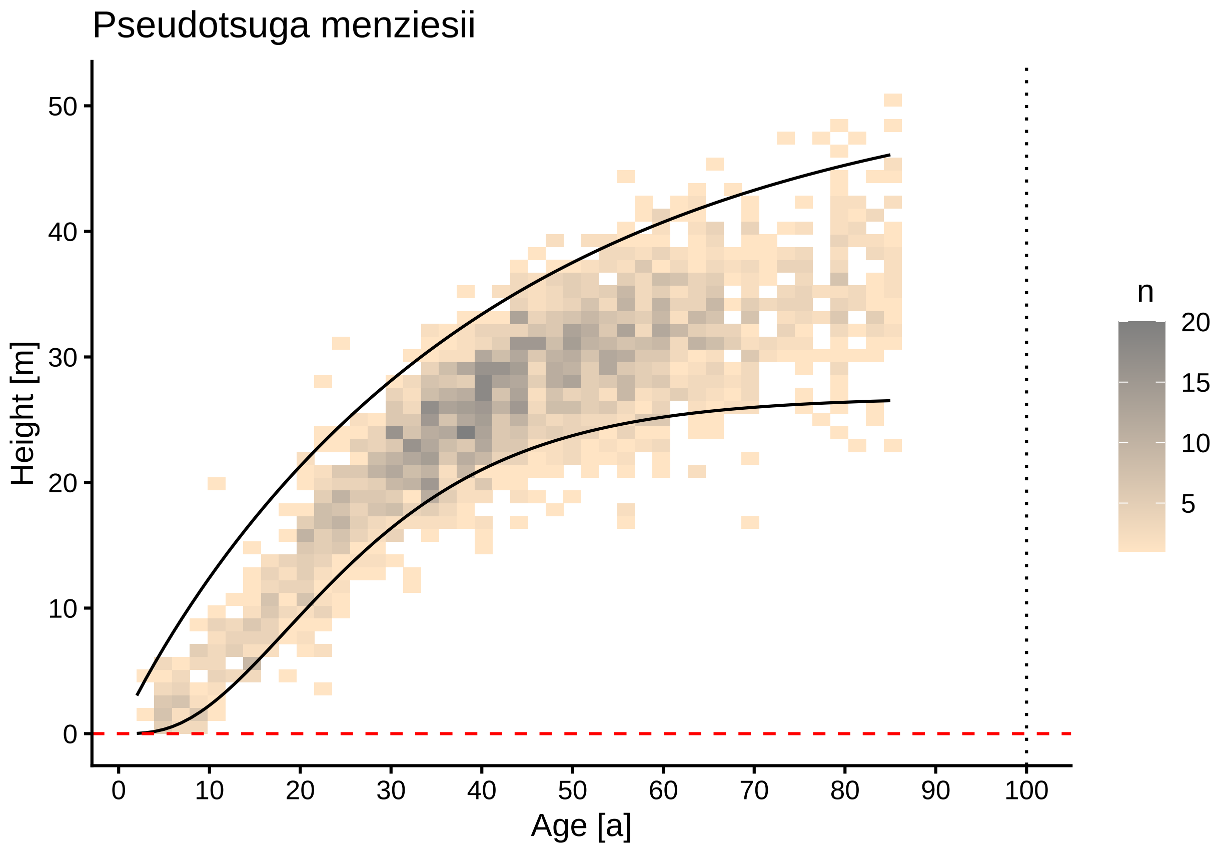

Model statistics and evaluation

Summary

Predictor acronyms: Bio.1 = Mean annual temperature [°C], Bio.12 = Annual precipitation sum [mm/m2], sp_p = Sum of precipitation [mm/m2] within months 3 to 5, su_p = Sum of precipitation [mm/m2] within months 6 to 8, wi_p = Sum of precipitation [mm/m2] within months 12,1,2, sp_t = Mean temperature [°C] within months 3 to 5, su_t = Mean temperature [°C] within months 6 to 8, wi_t = Mean temperature [°C] within months 12,1,2.

```
##
## Family: gaussian
## Link function: identity
##
## Formula:
## H03 ~ s(reference_19812010_su_t, k = 3) + s(reference_19812010_wi_t,
##     k = 3) + s(reference_19812010_sp_p, k = 3) + s(reference_19812010_su_p,
##     k = 3)
##
## Parametric coefficients:
##             Estimate Std. Error t value Pr(>|t|)
## (Intercept)  0.19309    0.01203   16.05   <2e-16 ***
## ---
## Signif. codes:  0 '***' 0.001 '**' 0.01 '*' 0.05 '.' 0.1 ' ' 1
##
## Approximate significance of smooth terms:
##                              edf Ref.df      F p-value
## s(reference_19812010_su_t) 1.993  2.000 126.13  <2e-16 ***
## s(reference_19812010_wi_t) 1.974  1.999 130.49  <2e-16 ***
## s(reference_19812010_sp_p) 1.984  2.000  33.06  <2e-16 ***
## s(reference_19812010_su_p) 1.845  1.976  21.84  <2e-16 ***
## ---
## Signif. codes:  0 '***' 0.001 '**' 0.01 '*' 0.05 '.' 0.1 ' ' 1
##
## R-sq.(adj) =  0.689   Deviance explained = 69.1%
## -REML = 507.28  Scale est. = 0.1497    n = 1034
```

Variance inflation factor (VIF)

Predictor acronyms: Bio.1 = Mean annual temperature [°C], Bio.12 = Annual precipitation sum [mm/m2], sp_p = Sum of precipitation [mm/m2] within months 3 to 5, su_p = Sum of precipitation [mm/m2] within months 6 to 8, wi_p = Sum of precipitation [mm/m2] within months 12,1,2, sp_t = Mean temperature [°C] within months 3 to 5, su_t = Mean temperature [°C] within months 6 to 8, wi_t = Mean temperature [°C] within months 12,1,2.

```
##                  Variables      VIF
## 1 reference_19812010_su_t 1.701305
## 2 reference_19812010_wi_t 2.063252
## 3 reference_19812010_sp_p 2.262898
## 4 reference_19812010_su_p 2.676020
```

Correlation matrix

Correlation matrix between the predictor variables and the target variable in the model. Correlation coefficient according to PEARSON. Predictor acronyms: Bio.1 = Mean annual temperature [°C], Bio.12 = Annual precipitation sum [mm/m2], sp_p = Sum of precipitation [mm/m2] within months 3 to 5, su_p = Sum of precipitation [mm/m2] within months 6 to 8, wi_p = Sum of precipitation [mm/m2] within months 12,1,2, sp_t = Mean temperature [°C] within months 3 to 5, su_t = Mean temperature [°C] within months 6 to 8, wi_t = Mean temperature [°C] within months 12,1,2.

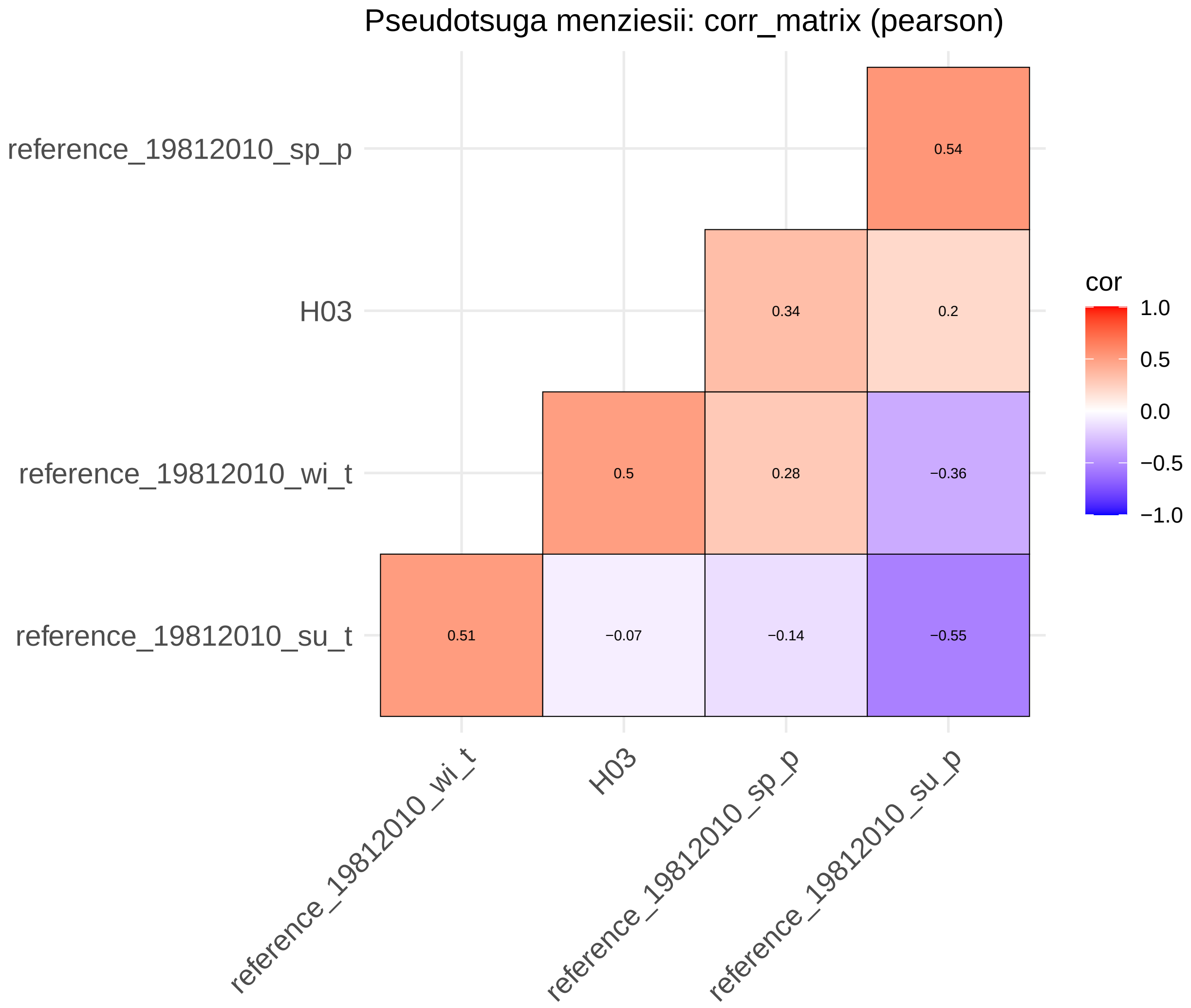

Response curves

Response curves (also known as effect curves) show how each predictor variable affects the target variable (H03 = european Site index, SIrel). H03 values below zero represent 'Growth absences'. Predictor acronyms: Bio.1 = Mean annual temperature [°C], Bio.12 = Annual precipitation sum [mm/m2], sp_p = Sum of precipitation [mm/m2] within months 3 to 5, su_p = Sum of precipitation [mm/m2] within months 6 to 8, wi_p = Sum of precipitation [mm/m2] within months 12,1,2, sp_t = Mean temperature [°C] within months 3 to 5, su_t = Mean temperature [°C] within months 6 to 8, wi_t = Mean temperature [°C] within months 12,1,2.

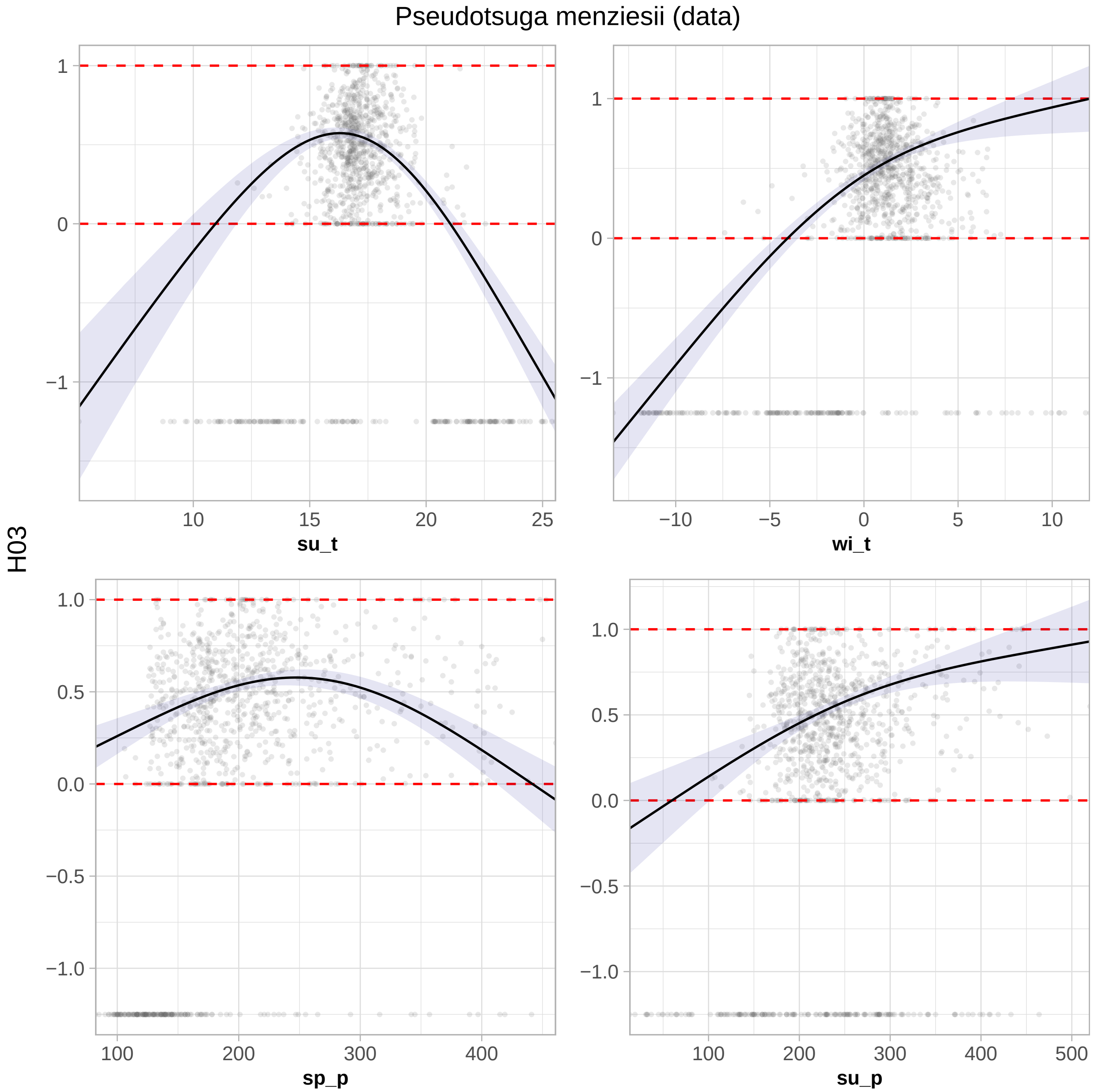

Response maps

Response maps (also referred as partial effect maps). Each map visualizes how a predictor affect the target variable (top height [m] at Age 100). Technically their work like response curves in a geographical area, that is setting all predictor variables except the one shown in the figure on their mean, and mapping the prediction. Predictor acronyms: Bio.1 = Mean annual temperature [°C], Bio.12 = Annual precipitation sum [mm/m2], sp_p = Sum of precipitation [mm/m2] within months 3 to 5, su_p = Sum of precipitation [mm/m2] within months 6 to 8, wi_p = Sum of precipitation [mm/m2] within months 12,1,2, sp_t = Mean temperature [°C] within months 3 to 5, su_t = Mean temperature [°C] within months 6 to 8, wi_t = Mean temperature [°C] within months 12,1,2.

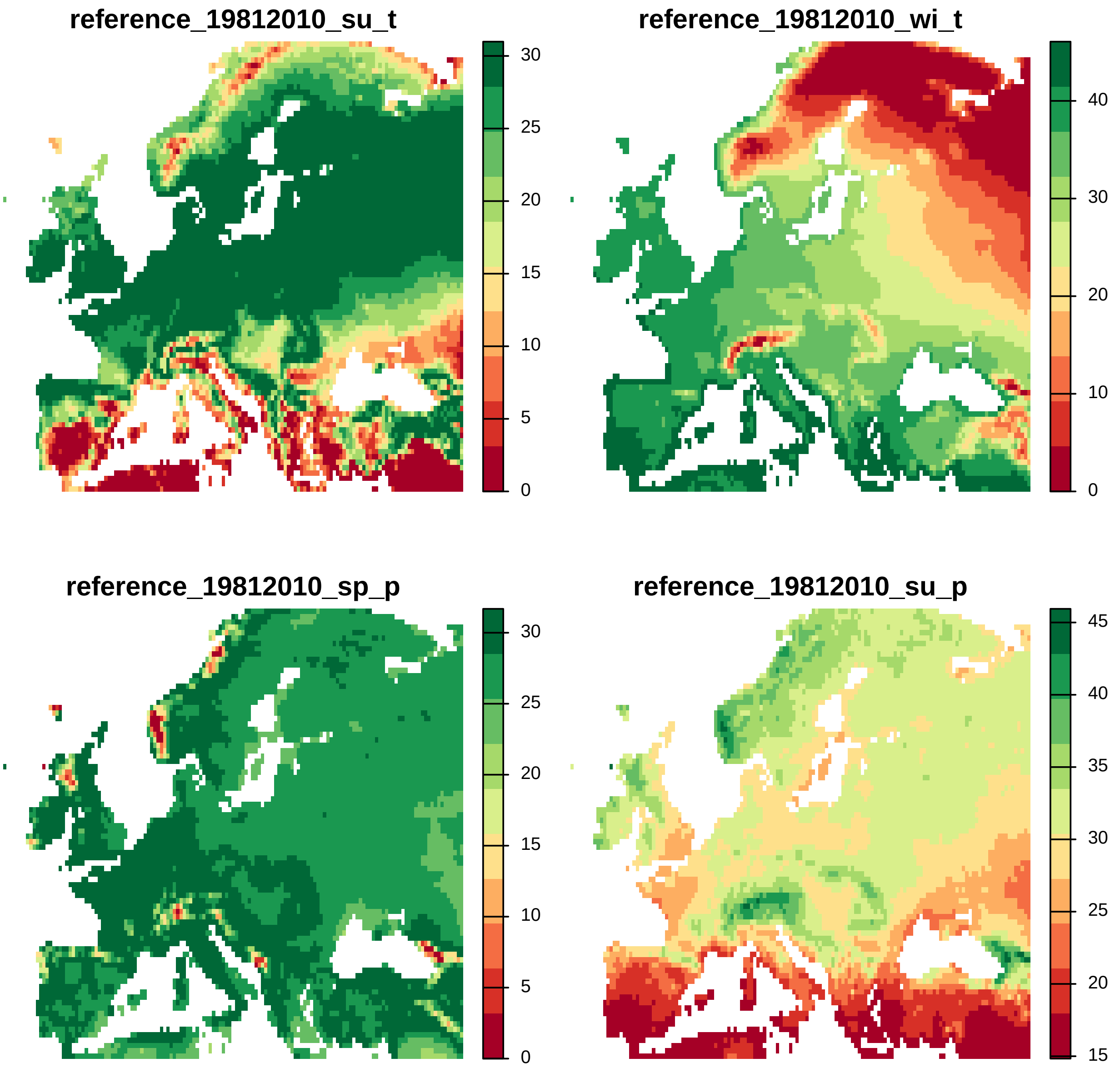

Residual distribution

The multi-panel plot includes a histogram of the residuals (top left), residuals over fitted values (top right), a histogram of observed and predicted values (bottom left) and boxplot diagram of observed and predicted values (bottom right). Observed values are shown in light green, while predicted ones are depicted in light red.

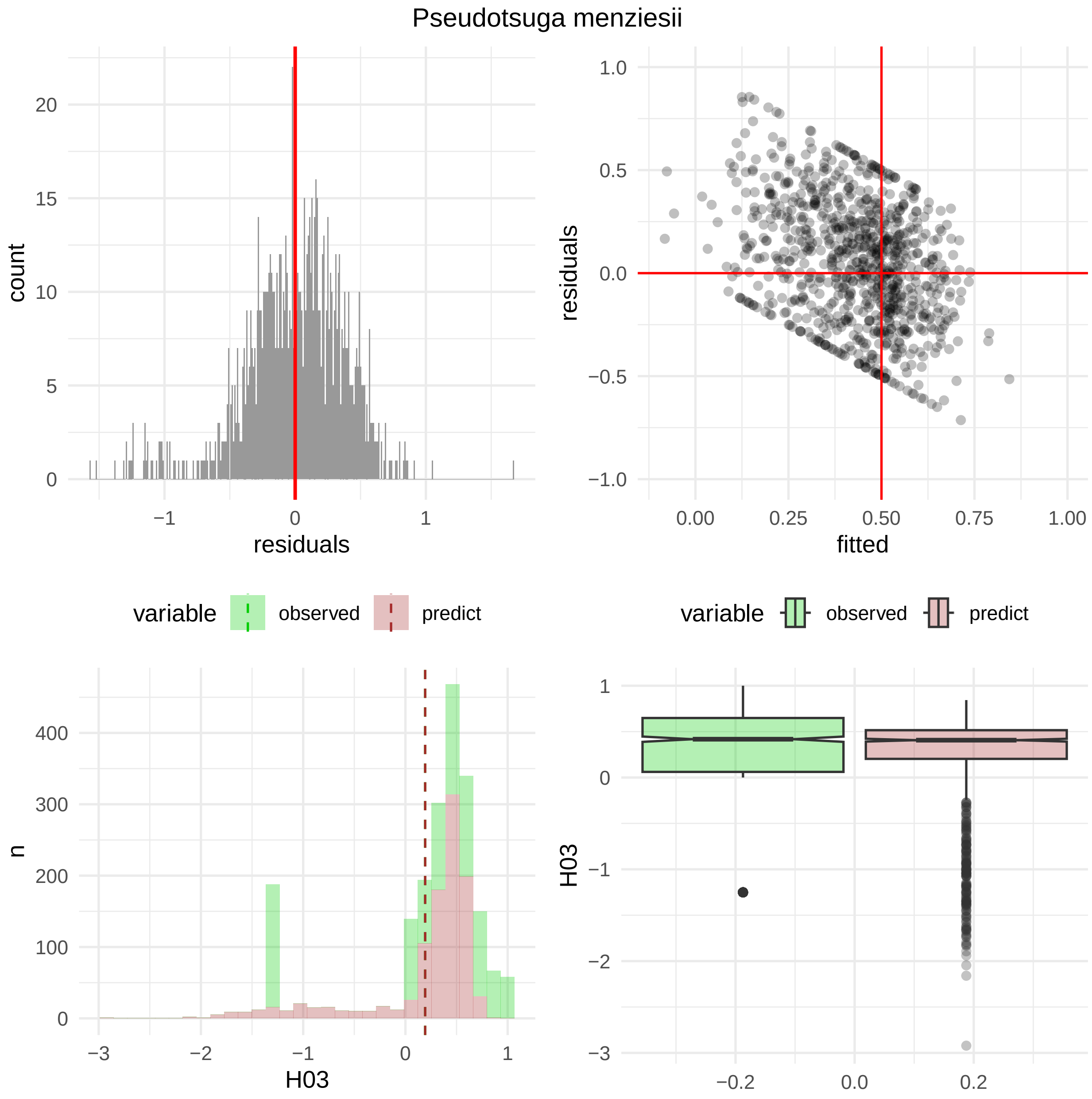

Correlation between predict and observed site index

Relationship between predicted and observed site index (density cloud), as well as linear regressions of presences and absences (= 'growth absences') (red line) and presences only (magenta line). The formulas, significance, R2 and number of observations are displayed below for both regressions. Ideally, both the point cloud and the regression lines lie close to the dashed line. For presences only we additionally calculated the correlation coefficient according to PEARSON (cor.pre) in the bottom right corner.

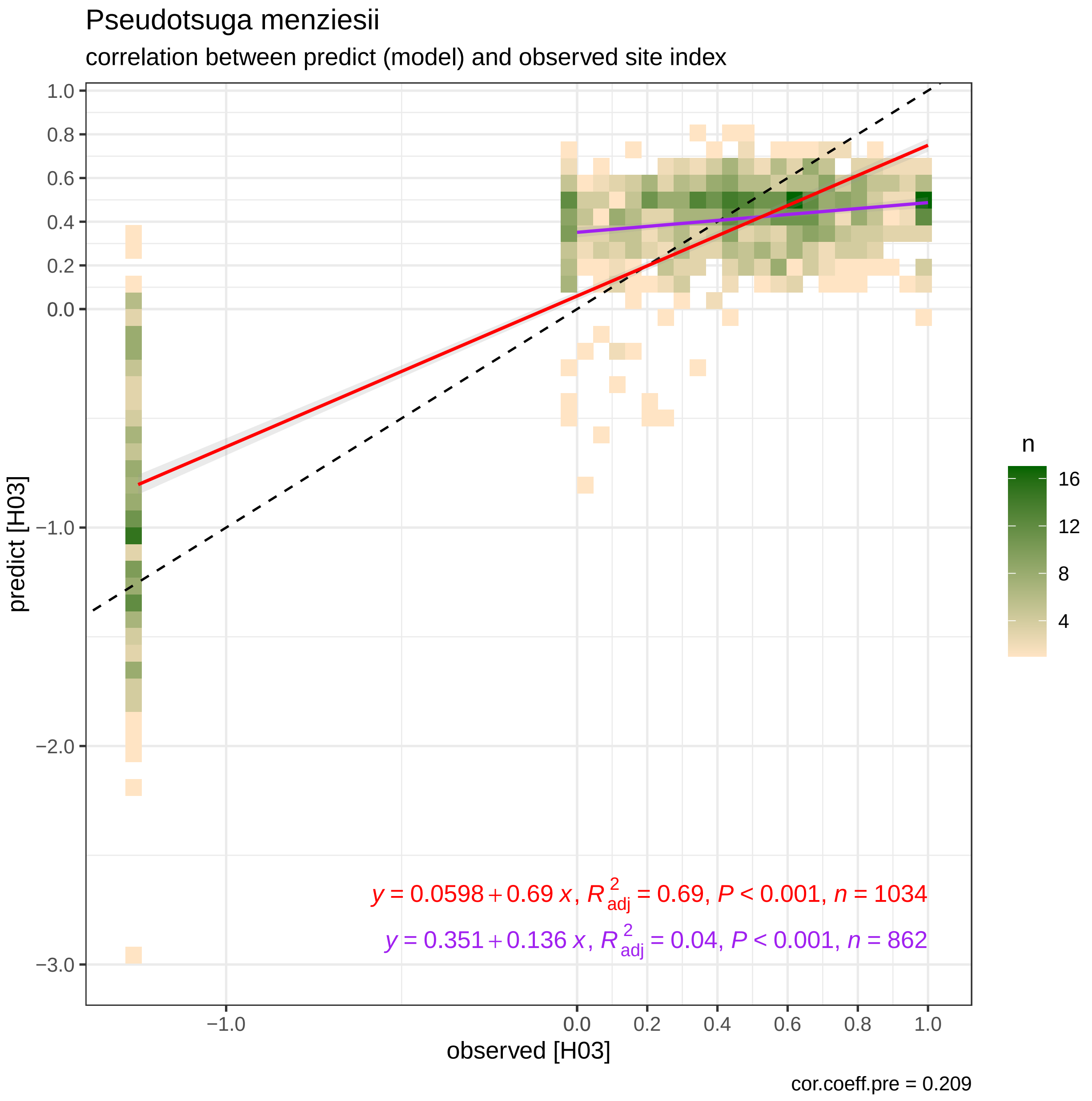

Predictions and forecasts

Predict

European predict for the reference period (1981 to 2010). Dark green symbolizes a high site index (tree height in meters at age 100), orange a lower site index and red no growth. Magenta-coloured dots represent inventory points with growth information, light blue dots are absences (= 'growth absences'). Results were aggregated on 25 km x 25 km scale.

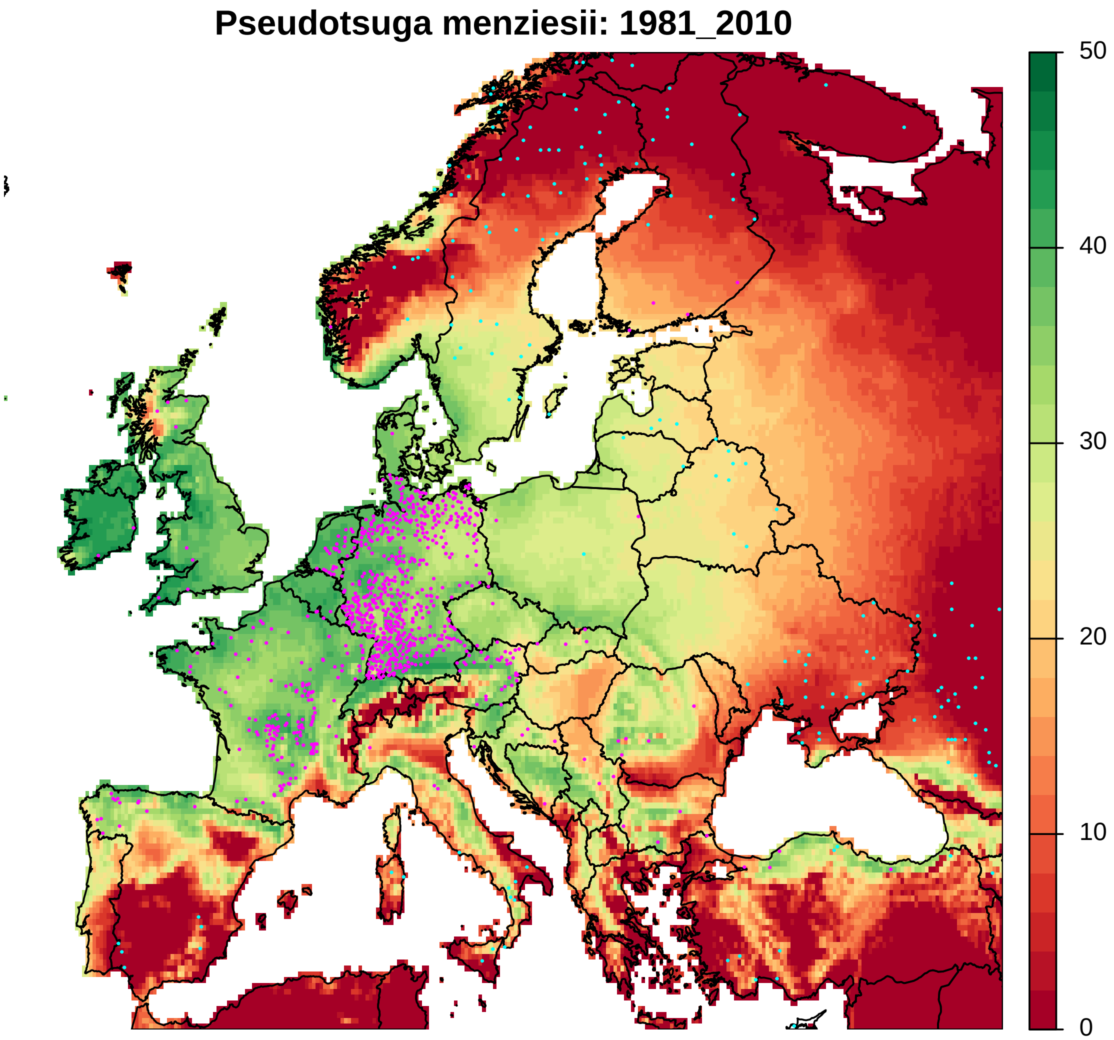

Forecast

Prediction for the reference period (1981 to 2010), as well as forecasts to 2071 to 2100 under szenario RCP4.5 and RCP8.5. Dark green symbolizes a high site index (tree height in m at age 100), orange a lower site index and red no growth. Results were aggregated on 25 km x 25 km scale.

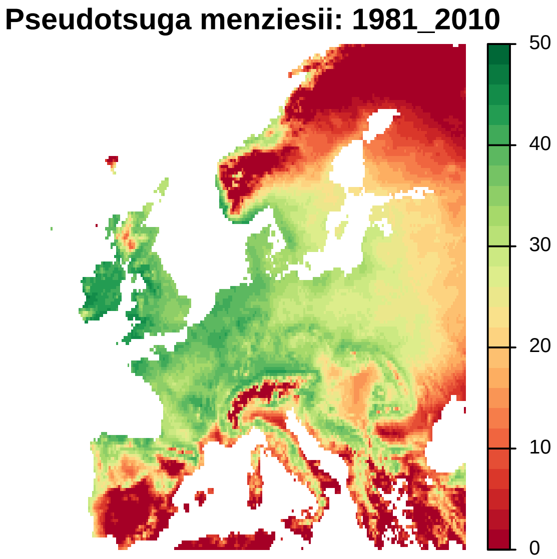

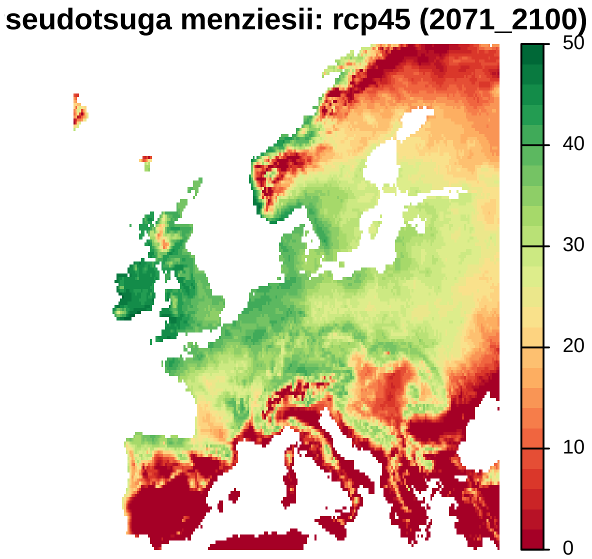

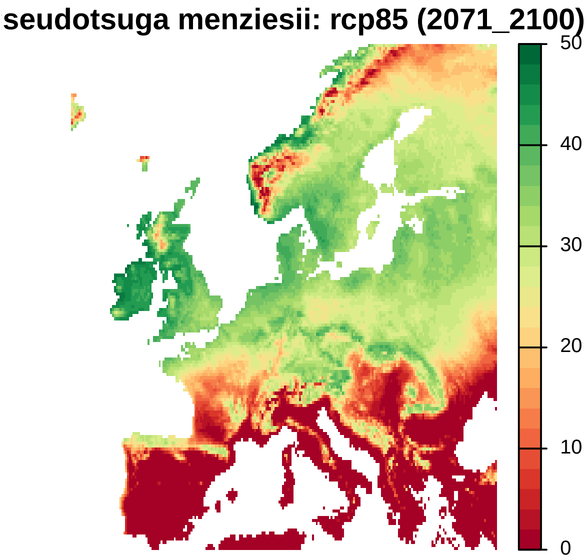

Quercus cerris

Site index curves

Site index curves of Quercus cerris created with non-linear quantile regressions based on the algorithm of Koenker and Park (1992). The site index (SI) was created by setting all points on the 95 percent quantile (upper line) and above to one (SI = 1) and all on the 5 percent quantile (lower line) and below to zero (SI = 0). The points between the quantile boundaries were assigned a site index between zero and one according to the ratio of their position between the quantile boundaries. We set selected absences (see chapter 2.1.3) on Height = 0 m (at age 100), which means, depending on the site index curves, for each tree species a SI near -1 (red line).

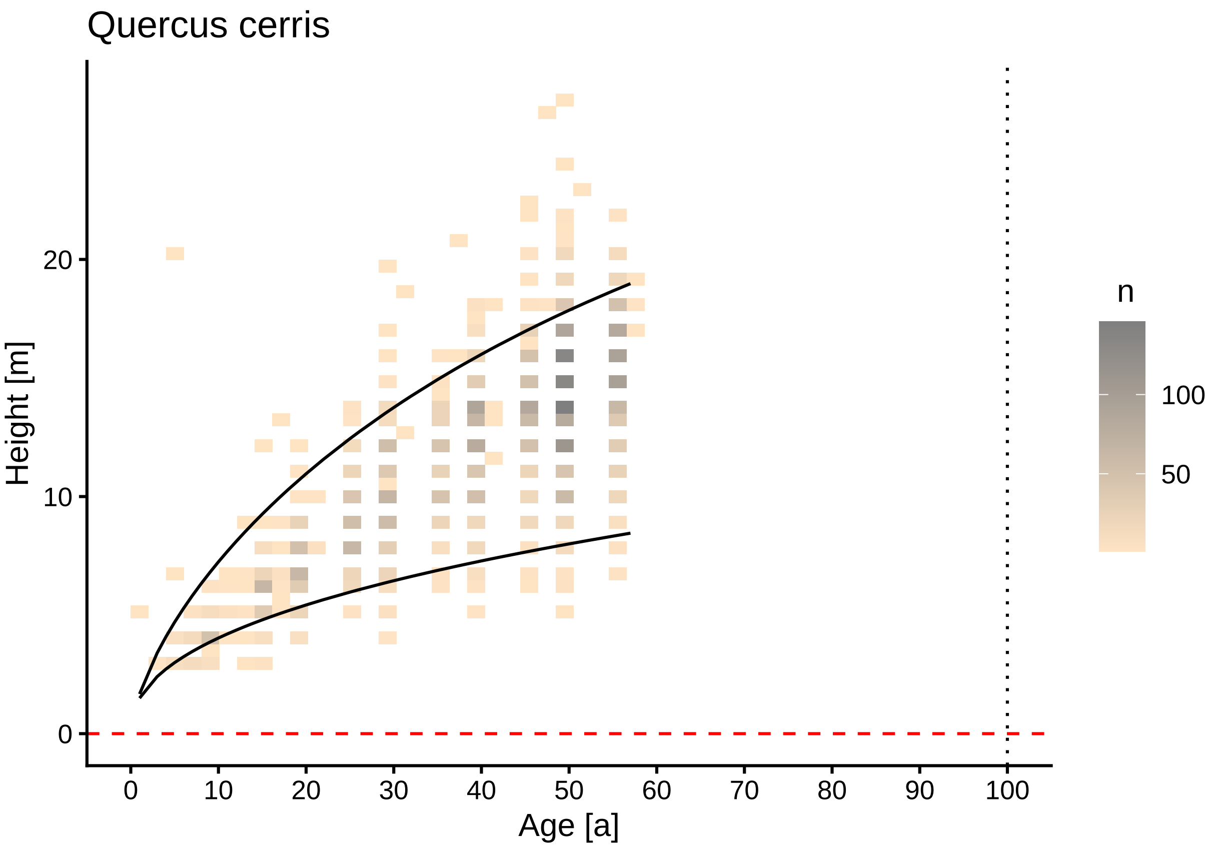

Model statistics and evaluation

Summary

Predictor acronyms: Bio.1 = Mean annual temperature [°C], Bio.12 = Annual precipitation sum [mm/m2], sp_p = Sum of precipitation [mm/m2] within months 3 to 5, su_p = Sum of precipitation [mm/m2] within months 6 to 8, wi_p = Sum of precipitation [mm/m2] within months 12,1,2, sp_t = Mean temperature [°C] within months 3 to 5, su_t = Mean temperature [°C] within months 6 to 8, wi_t = Mean temperature [°C] within months 12,1,2.

```
##
## Family: gaussian
## Link function: identity
##
## Formula:
## H03 ~ s(reference_19812010_su_t, k = 3) + s(reference_19812010_wi_p,
##     k = 3) + s(reference_19812010_wi_t, k = 3)
##
## Parametric coefficients:
##             Estimate Std. Error t value Pr(>|t|)
## (Intercept)  0.53593    0.02046    26.2   <2e-16 ***
## ---
## Signif. codes:  0 '***' 0.001 '**' 0.01 '*' 0.05 '.' 0.1 ' ' 1
##
## Approximate significance of smooth terms:
##                              edf Ref.df     F  p-value
## s(reference_19812010_su_t) 1.000  1.000 15.50 9.76e-05 ***
## s(reference_19812010_wi_p) 1.891  1.988 12.66 6.37e-06 ***
## s(reference_19812010_wi_t) 1.991  2.000 59.61  < 2e-16 ***
## ---
## Signif. codes:  0 '***' 0.001 '**' 0.01 '*' 0.05 '.' 0.1 ' ' 1
##
## R-sq.(adj) =  0.585   Deviance explained =   59%
## -REML = 225.83  Scale est. = 0.16865   n = 403
```

Variance inflation factor (VIF)

Predictor acronyms: Bio.1 = Mean annual temperature [°C], Bio.12 = Annual precipitation sum [mm/m2], sp_p = Sum of precipitation [mm/m2] within months 3 to 5, su_p = Sum of precipitation [mm/m2] within months 6 to 8, wi_p = Sum of precipitation [mm/m2] within months 12,1,2, sp_t = Mean temperature [°C] within months 3 to 5, su_t = Mean temperature [°C] within months 6 to 8, wi_t = Mean temperature [°C] within months 12,1,2.

```
##                  Variables      VIF
## 1 reference_19812010_su_t 4.071660
## 2 reference_19812010_wi_p 1.781762
## 3 reference_19812010_wi_t 3.317299
```

Correlation matrix

Correlation matrix between the predictor variables and the target variable in the model. Correlation coefficient according to PEARSON. Predictor acronyms: Bio.1 = Mean annual temperature [°C], Bio.12 = Annual precipitation sum [mm/m2], sp_p = Sum of precipitation [mm/m2] within months 3 to 5, su_p = Sum of precipitation [mm/m2] within months 6 to 8, wi_p = Sum of precipitation [mm/m2] within months 12,1,2, sp_t = Mean temperature [°C] within months 3 to 5, su_t = Mean temperature [°C] within months 6 to 8, wi_t = Mean temperature [°C] within months 12,1,2.

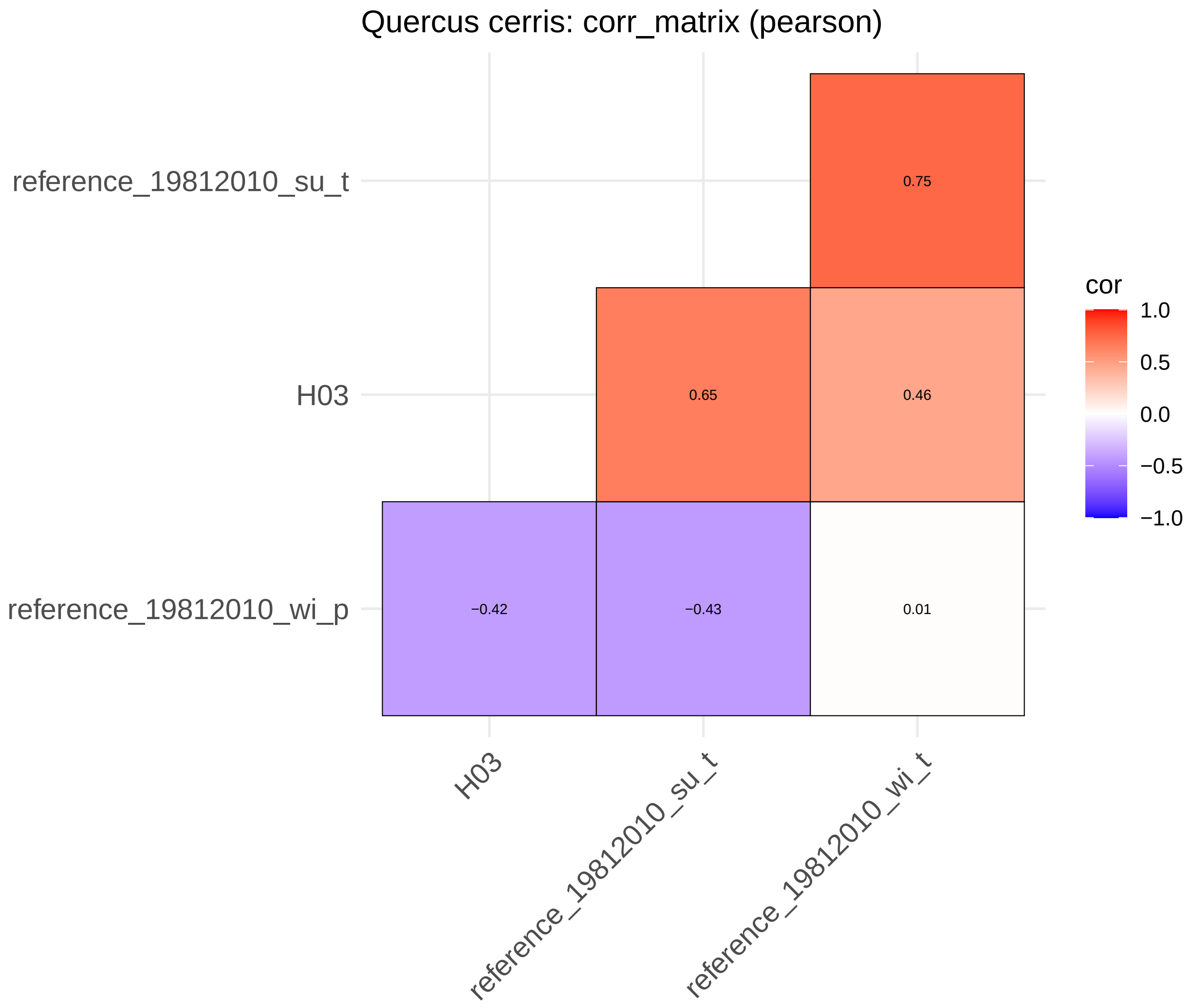

Response curves

Response curves (also known as effect curves) show how each predictor variable affects the target variable (H03 = european Site index, SIrel). H03 values below zero represent 'Growth absences'. Predictor acronyms: Bio.1 = Mean annual temperature [°C], Bio.12 = Annual precipitation sum [mm/m2], sp__p = Sum of precipitation [mm/m2] within months 3 to 5, su__p = Sum of precipitation [mm/m2] within months 6 to 8, wi__p = Sum of precipitation [mm/m2] within months 12,1,2, sp__t = Mean temperature [°C] within months 3 to 5, su__t = Mean temperature [°C] within months 6 to 8, wi__t = Mean temperature [°C] within months 12,1,2.

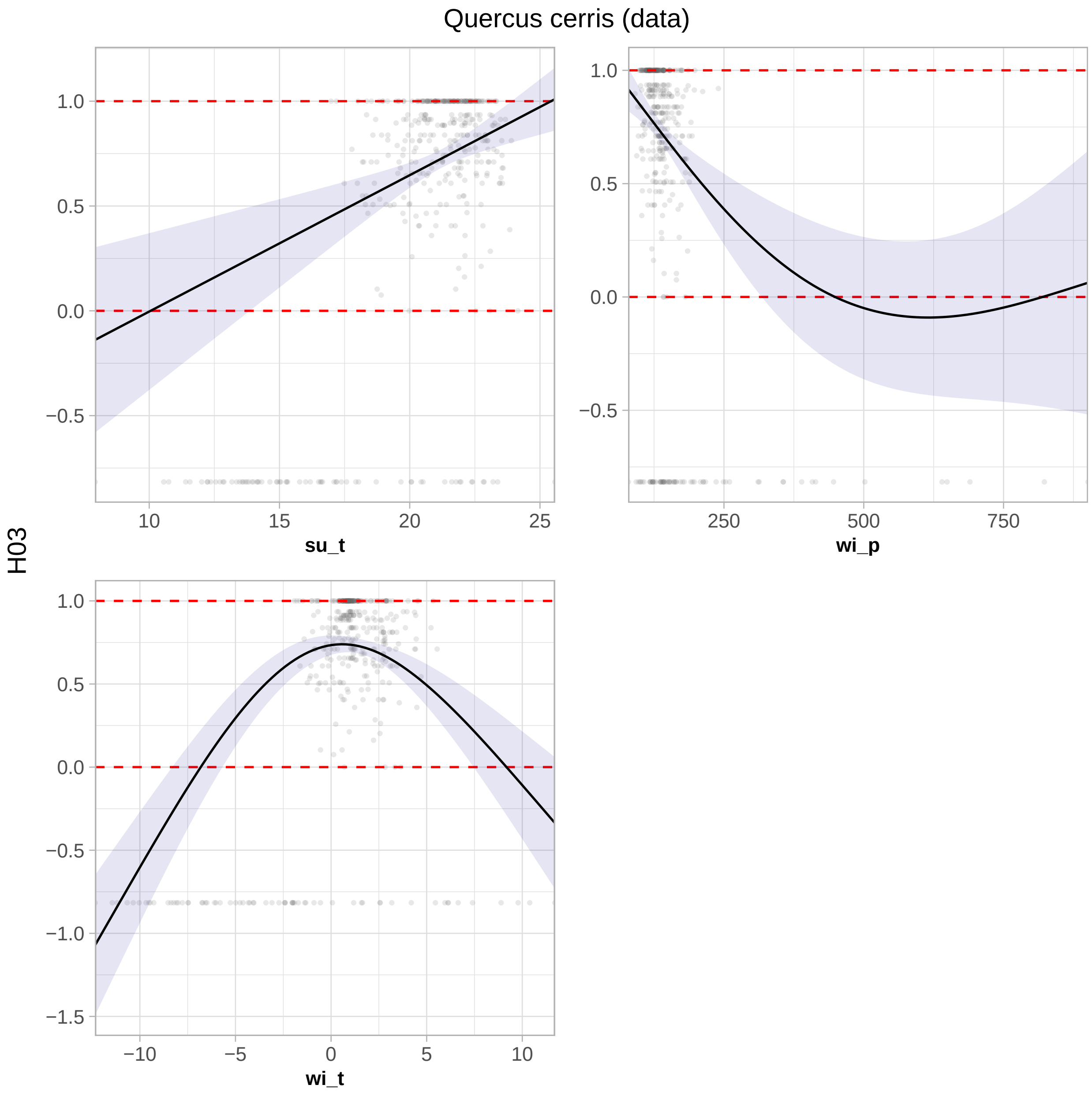

Response maps

Response maps (also referred as partial effect maps). Each map visualizes how a predictor affect the target variable (top height [m] at Age 100). Technically their work like response curves in a geographical area, that is setting all predictor variables except the one shown in the figure on their mean, and mapping the prediction. Predictor acronyms: Bio.1 = Mean annual temperature [°C], Bio.12 = Annual precipitation sum [mm/m2], sp_p = Sum of precipitation [mm/m2] within months 3 to 5, su_p = Sum of precipitation [mm/m2] within months 6 to 8, wi_p = Sum of precipitation [mm/m2] within months 12,1,2, sp_t = Mean temperature [°C] within months 3 to 5, su_t = Mean temperature [°C] within months 6 to 8, wi_t = Mean temperature [°C] within months 12,1,2.

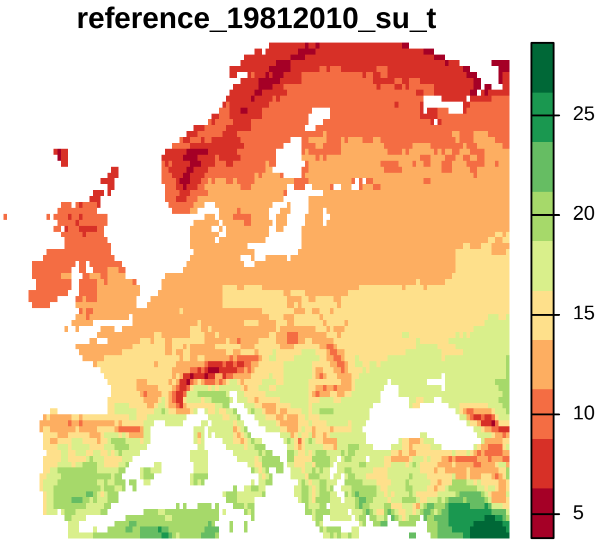

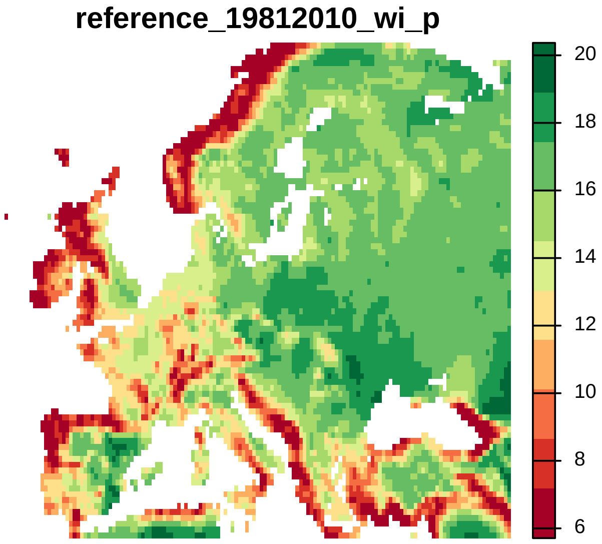

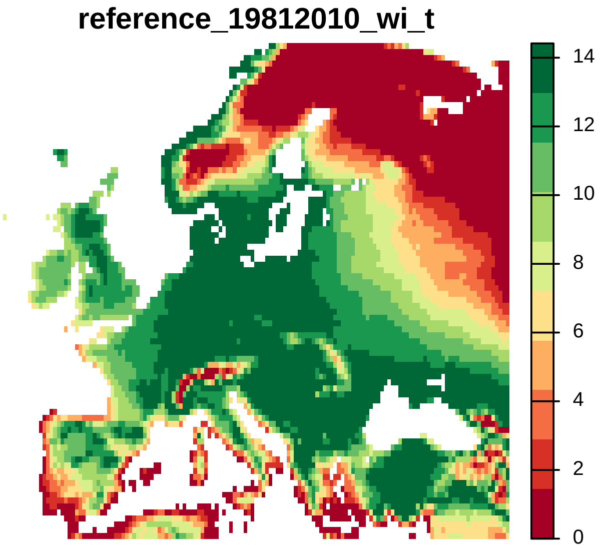

Residual distribution

The multi-panel plot includes a histogram of the residuals (top left), residuals over fitted values (top right), a histogram of observed and predicted values (bottom left) and boxplot diagram of observed and predicted values (bottom right). Observed values are shown in light green, while predicted ones are depicted in light red.

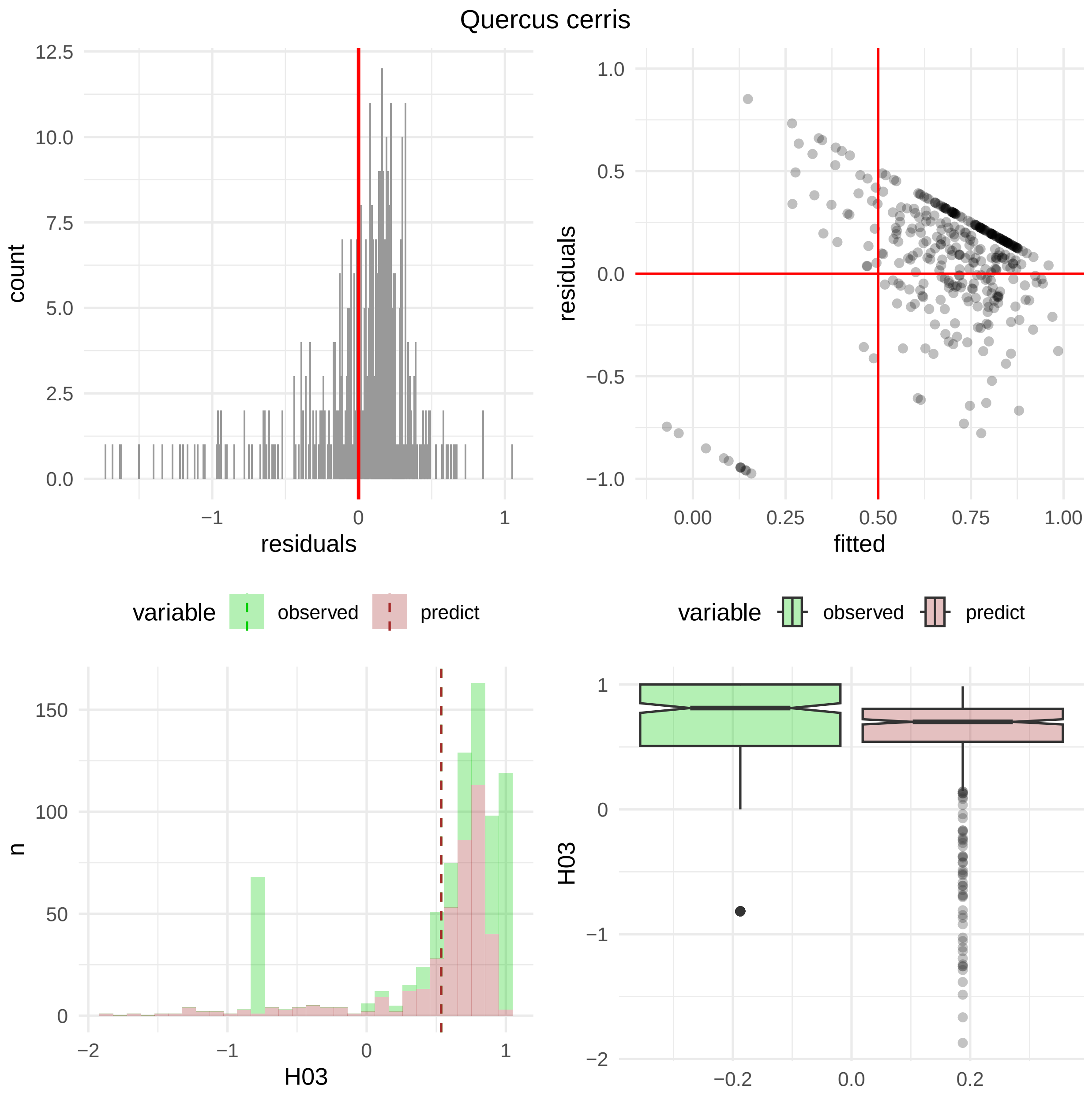

Correlation between predict and observed site index

Relationship between predicted and observed site index (density cloud), as well as linear regressions of presences and absences (= ‘growth absences’) (red line) and presences only (magenta line). The formulas, significance, R2 and number of observations are displayed below for both regressions. Ideally, both the point cloud and the regression lines lie close to the dashed line. For presences only we additionally calculated the correlation coefficient according to PEARSON (cor.pre) in the bottom right corner.

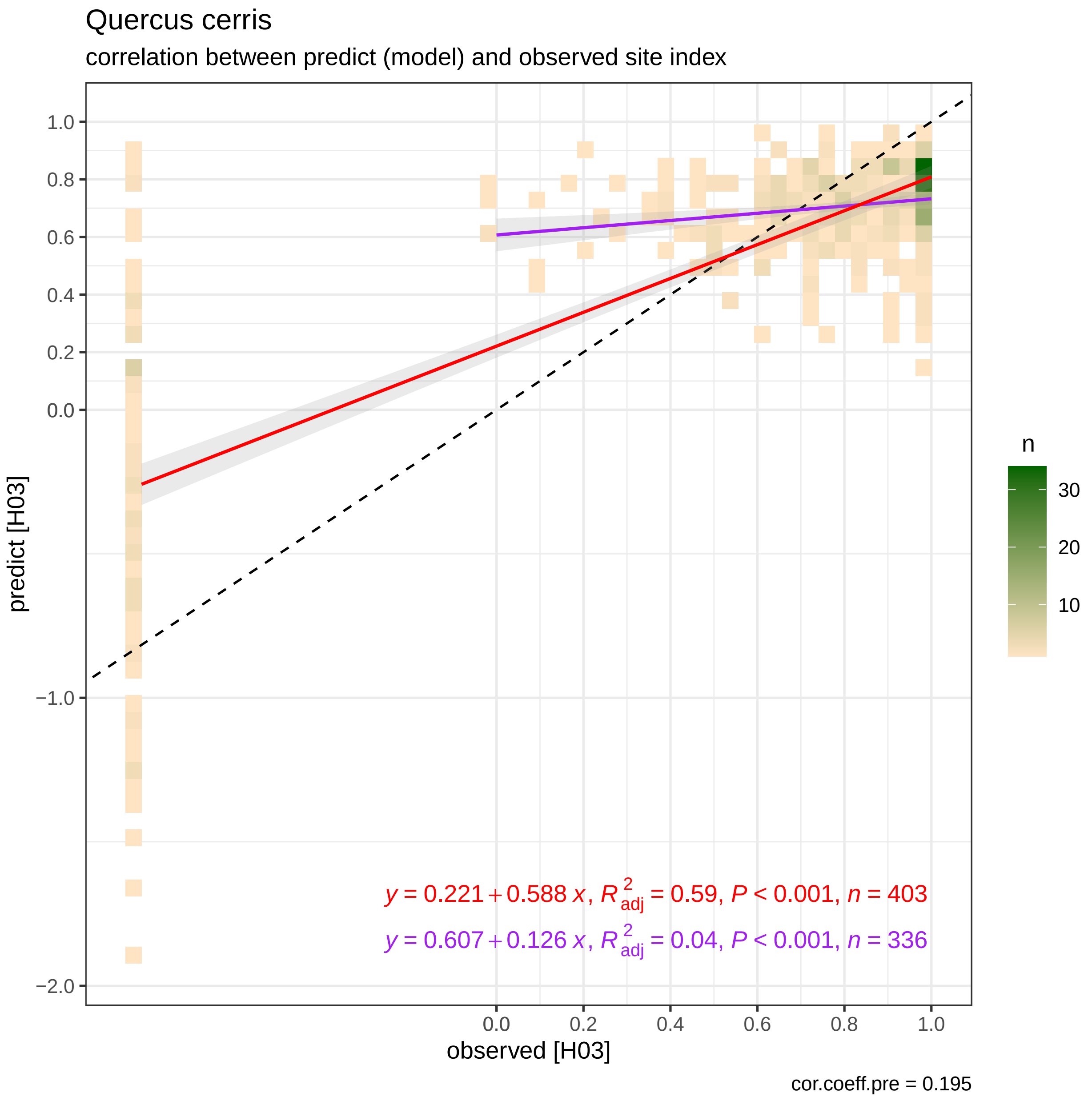

Predictions and forecasts

Predict

European predict for the reference period (1981 to 2010). Dark green symbolizes a high site index (tree height in meters at age 100), orange a lower site index and red no growth. Magenta-coloured dots represent inventory points with growth information, light blue dots are absences (= ‘growth absences’). Results were aggregated on 25 km x 25 km scale.

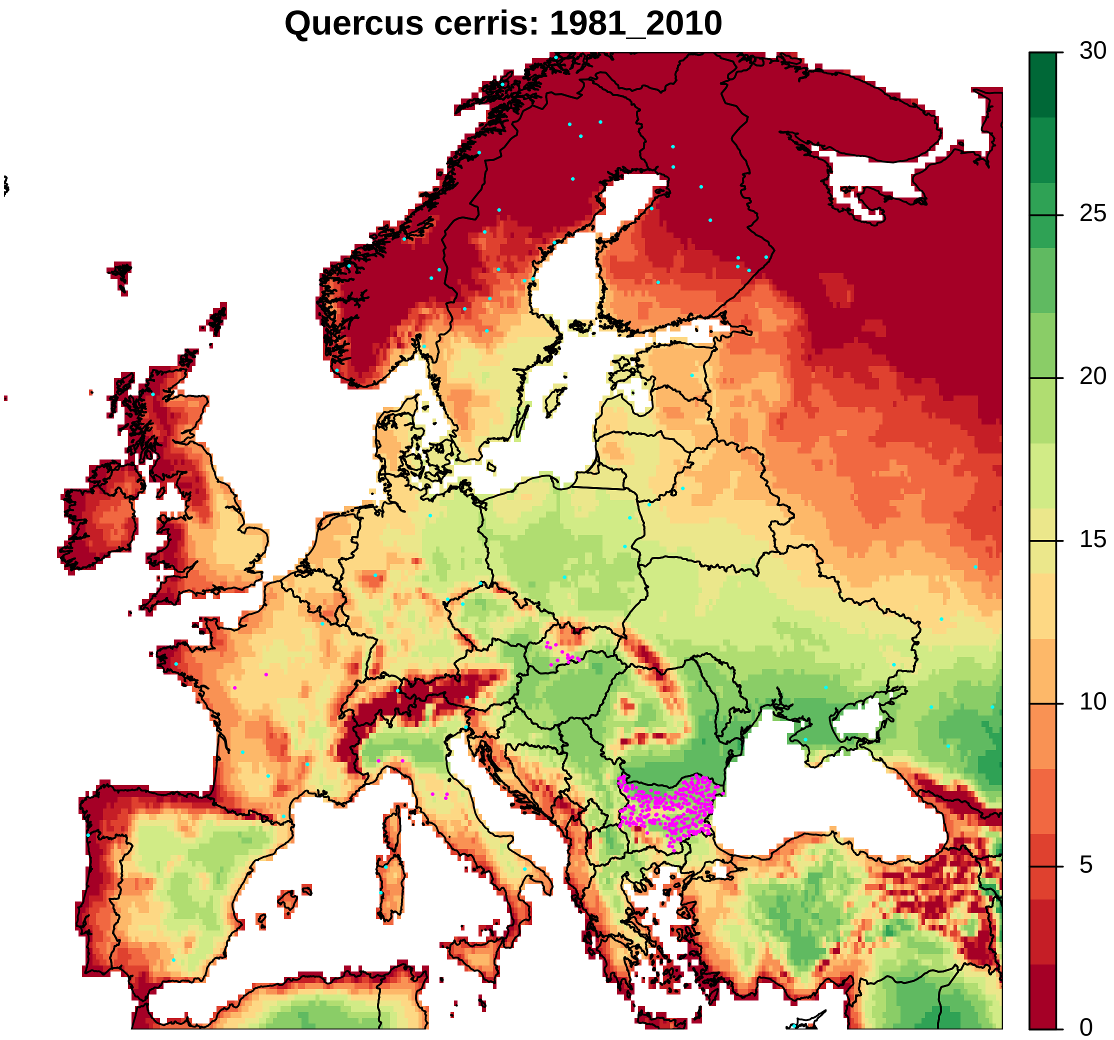

Forecast

Prediction for the reference period (1981 to 2010), as well as forecasts to 2071 to 2100 under szenario RCP4.5 and RCP8.5. Dark green symbolizes a high site index (tree height in m at age 100), orange a lower site index and red no growth. Results were aggregated on 25 km x 25 km scale.

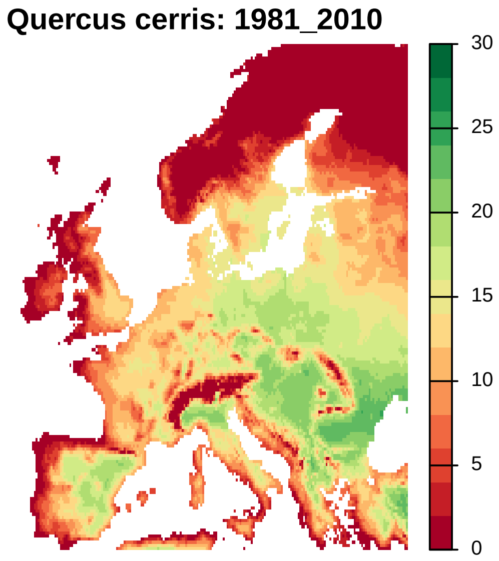

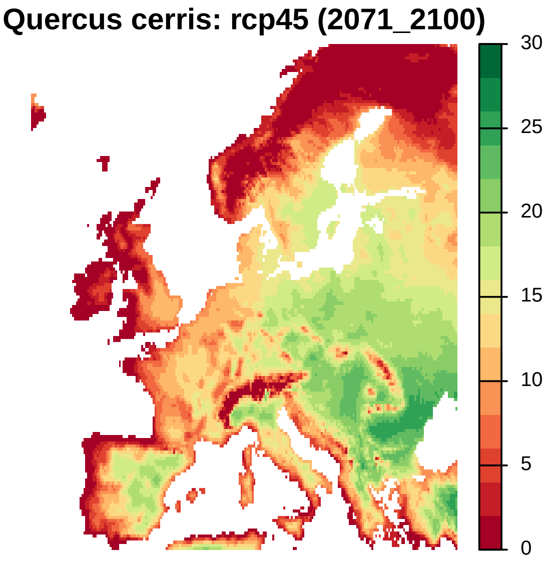

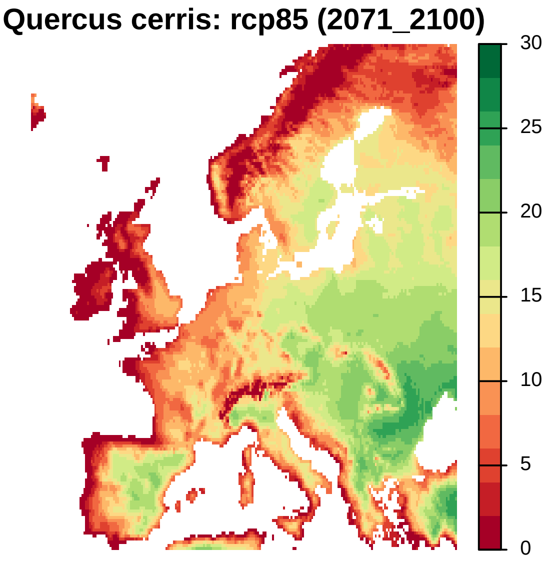

Quercus petraea

Site index curves

Site index curves of Quercus petraea created with non-linear quantile regressions based on the algorithm of Koenker and Park (1992). The site index (SI) was created by setting all points on the 95 percent quantile (upper line) and above to one (SI = 1) and all on the 5 percent quantile (lower line) and below to zero (SI = 0). The points between the quantile boundaries were assigned a site index between zero and one according to the ratio of their position between the quantile boundaries. We set selected absences (see chapter 2.1.3) on Height = 0 m (at age 100), which means, depending on the site index curves, for each tree species a SI near -1 (red line).

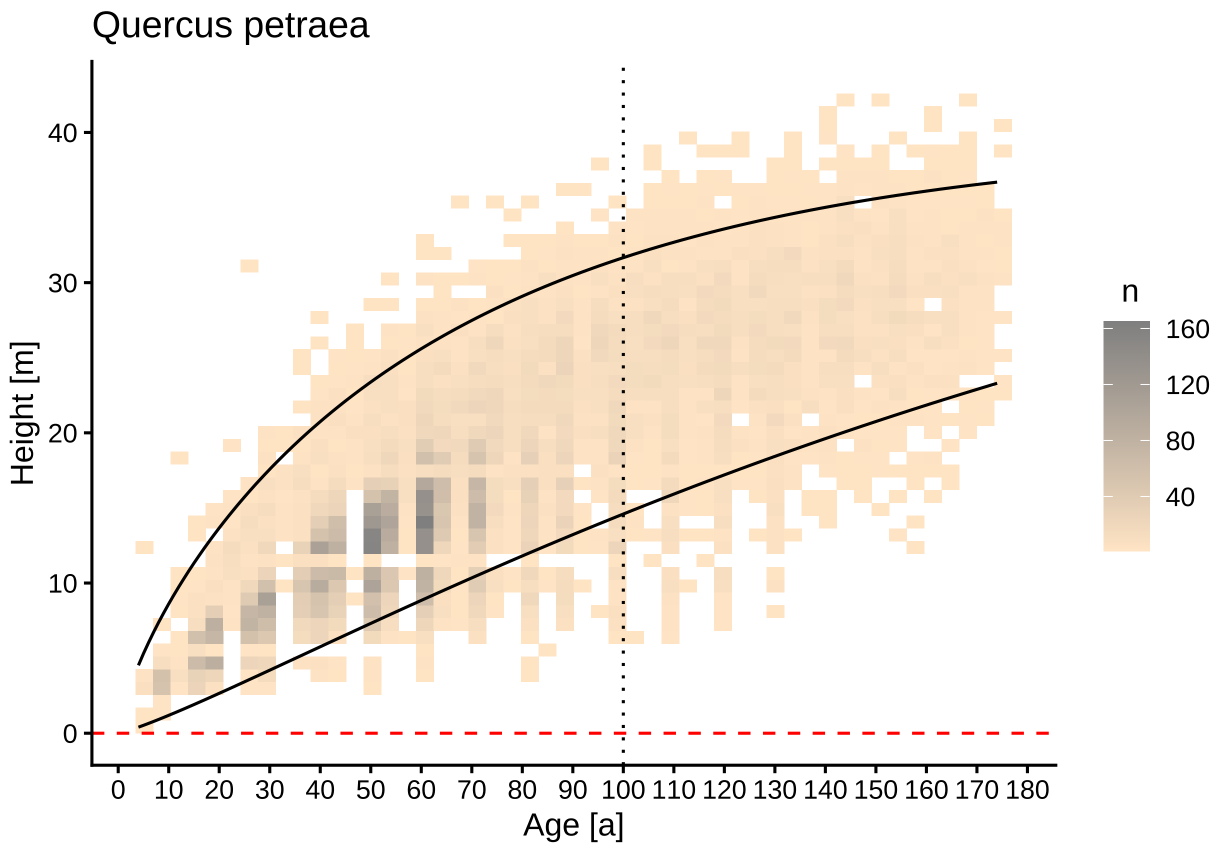

Model statistics and evaluation

Summary

Predictor acronyms: Bio.1 = Mean annual temperature [°C], Bio.12 = Annual precipitation sum [mm/m2], sp_p = Sum of precipitation [mm/m2] within months 3 to 5, su_p = Sum of precipitation [mm/m2] within months 6 to 8, wi_p = Sum of precipitation [mm/m2] within months 12,1,2, sp_t = Mean temperature [°C] within months 3 to 5, su_t = Mean temperature [°C] within months 6 to 8, wi_t = Mean temperature [°C] within months 12,1,2.

```
##
## Family: gaussian
## Link function: identity
##
## Formula:
## H03 ~ s(reference_19812010_wi_t, k = 3) + s(reference_19812010_su_t,
##     k = 3) + s(reference_19812010_su_p, k = 3)
##
## Parametric coefficients:
##             Estimate Std. Error t value Pr(>|t|)
## (Intercept) 0.402111   0.008636   46.56   <2e-16 ***
## ---
## Signif. codes:  0 '***' 0.001 '**' 0.01 '*' 0.05 '.' 0.1 ' ' 1
##
## Approximate significance of smooth terms:
##                              edf Ref.df      F p-value
## s(reference_19812010_wi_t) 1.996      2 386.09  <2e-16 ***
## s(reference_19812010_su_t) 1.987      2  67.94  <2e-16 ***
## s(reference_19812010_su_p) 1.994      2  91.00  <2e-16 ***
## ---
## Signif. codes:  0 '***' 0.001 '**' 0.01 '*' 0.05 '.' 0.1 ' ' 1
##
## R-sq.(adj) =    0.57   Deviance explained = 57.2%
## -REML = 1096.8  Scale est. = 0.15984   n = 2143
```

Variance inflation factor (VIF)

Predictor acronyms: Bio.1 = Mean annual temperature [°C], Bio.12 = Annual precipitation sum [mm/m2], sp_p = Sum of precipitation [mm/m2] within months 3 to 5, su_p = Sum of precipitation [mm/m2] within months 6 to 8, wi_p = Sum of precipitation [mm/m2] within months 12,1,2, sp_t = Mean temperature [°C] within months 3 to 5, su_t = Mean temperature [°C] within months 6 to 8, wi_t = Mean temperature [°C] within months 12,1,2.

```
##                   Variables      VIF
## 1 reference_19812010_wi_t 1.551785
## 2 reference_19812010_su_t 2.086973
## 3 reference_19812010_su_p 1.751859
```

Correlation matrix

Correlation matrix between the predictor variables and the target variable in the model. Correlation coefficient according to PEARSON. Predictor acronyms: Bio.1 = Mean annual temperature [°C], Bio.12 = Annual precipitation sum [mm/m2], sp_p = Sum of precipitation [mm/m2] within months 3 to 5, su_p = Sum of precipitation [mm/m2] within months 6 to 8, wi_p = Sum of precipitation [mm/m2] within months 12,1,2, sp_t = Mean temperature [°C] within months 3 to 5, su_t = Mean temperature [°C] within months 6 to 8, wi_t = Mean temperature [°C] within months 12,1,2.

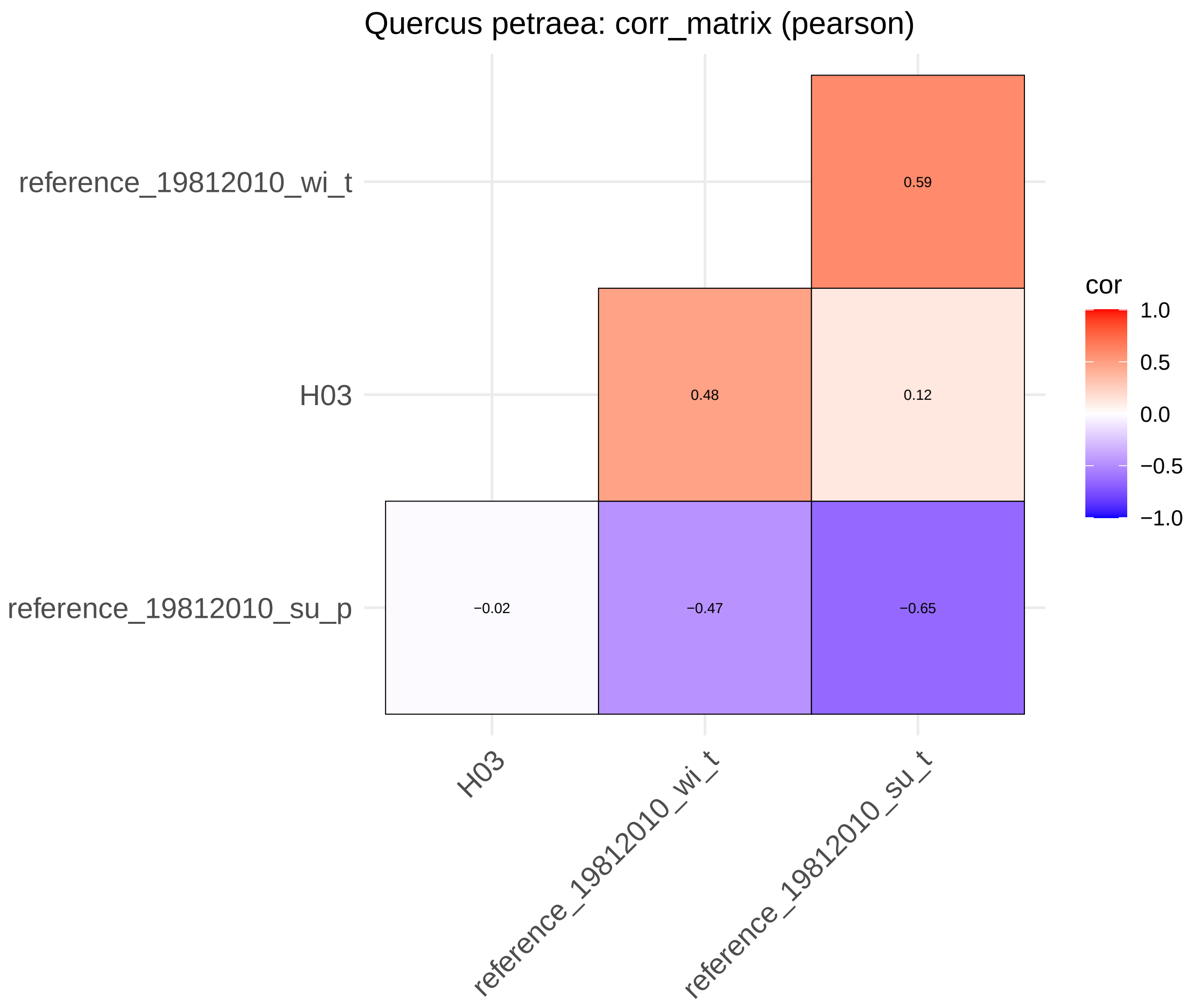

Response curves

Response curves (also known as effect curves) show how each predictor variable affects the target variable (H03 = european Site index, SIrel). H03 values below zero represent 'Growth absences'. Predictor acronyms: Bio.1 = Mean annual temperature [°C], Bio.12 = Annual precipitation sum [mm/m2], sp_p = Sum of precipitation [mm/m2] within months 3 to 5, su_p = Sum of precipitation [mm/m2] within months 6 to 8, wi_p = Sum of precipitation [mm/m2] within months 12,1,2, sp_t = Mean temperature [°C] within months 3 to 5, su_t = Mean temperature [°C] within months 6 to 8, wi_t = Mean temperature [°C] within months 12,1,2.

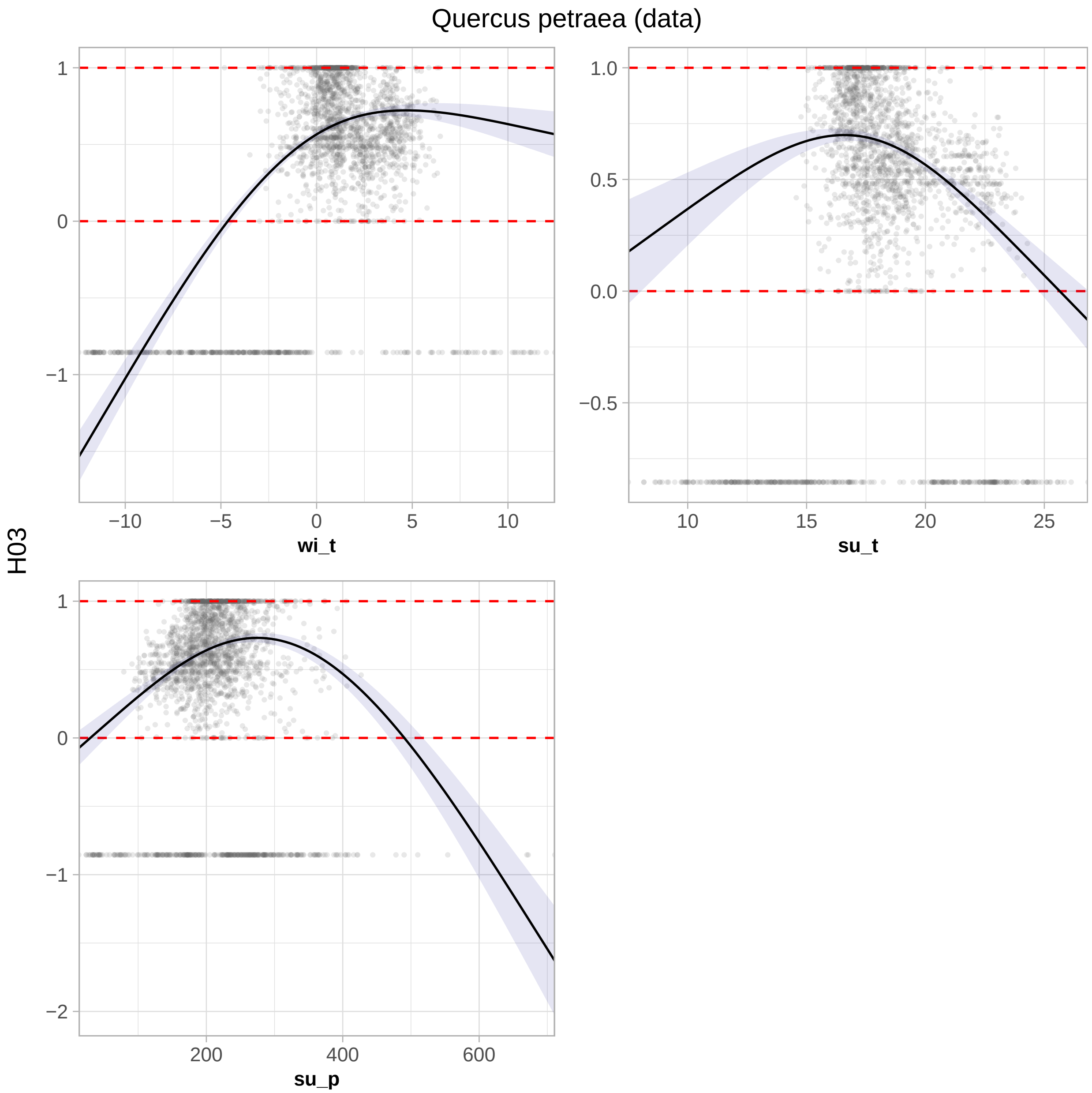

Response maps

Response maps (also referred as partial effect maps). Each map visualizes how a predictor affect the target variable (top height [m] at Age 100). Technically their work like response curves in a geographical area, that is setting all predictor variables except the one shown in the figure on their mean, and mapping the prediction. Predictor acronyms: Bio.1 = Mean annual temperature [°C], Bio.12 = Annual precipitation sum [mm/m2], sp_p = Sum of precipitation [mm/m2] within months 3 to 5, su_p = Sum of precipitation [mm/m2] within months 6 to 8, wi_p = Sum of precipitation [mm/m2] within months 12,1,2, sp_t = Mean temperature [°C] within months 3 to 5, su_t = Mean temperature [°C] within months 6 to 8, wi_t = Mean temperature [°C] within months 12,1,2.

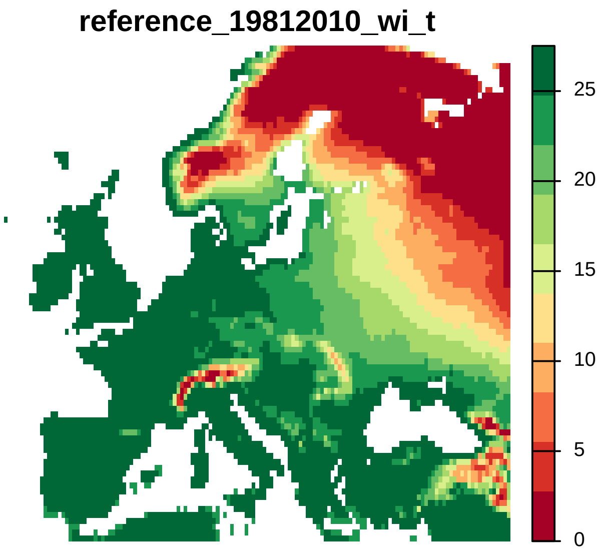

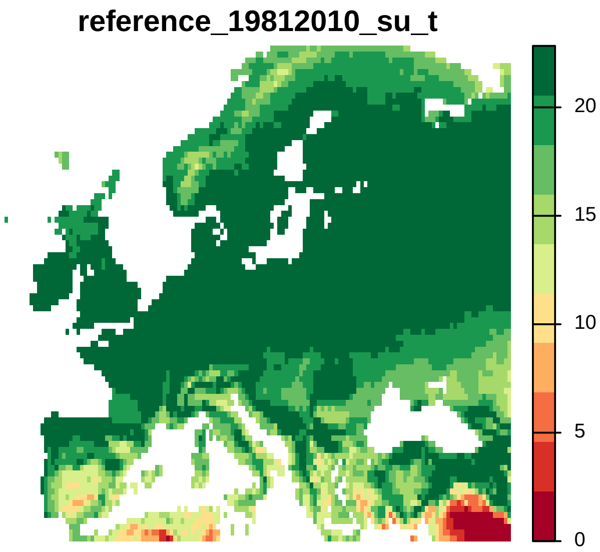

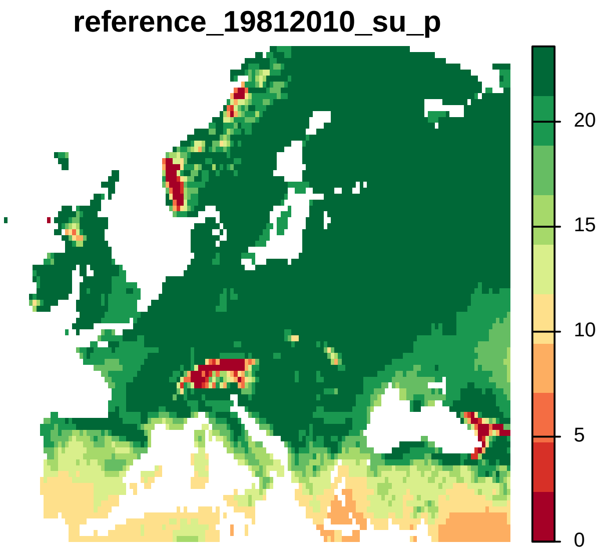

Residual distribution

The multi-panel plot includes a histogram of the residuals (top left), residuals over fitted values (top right), a histogram of observed and predicted values (bottom left) and boxplot diagram of observed and predicted values (bottom right). Observed values are shown in light green, while predicted ones are depicted in light red.

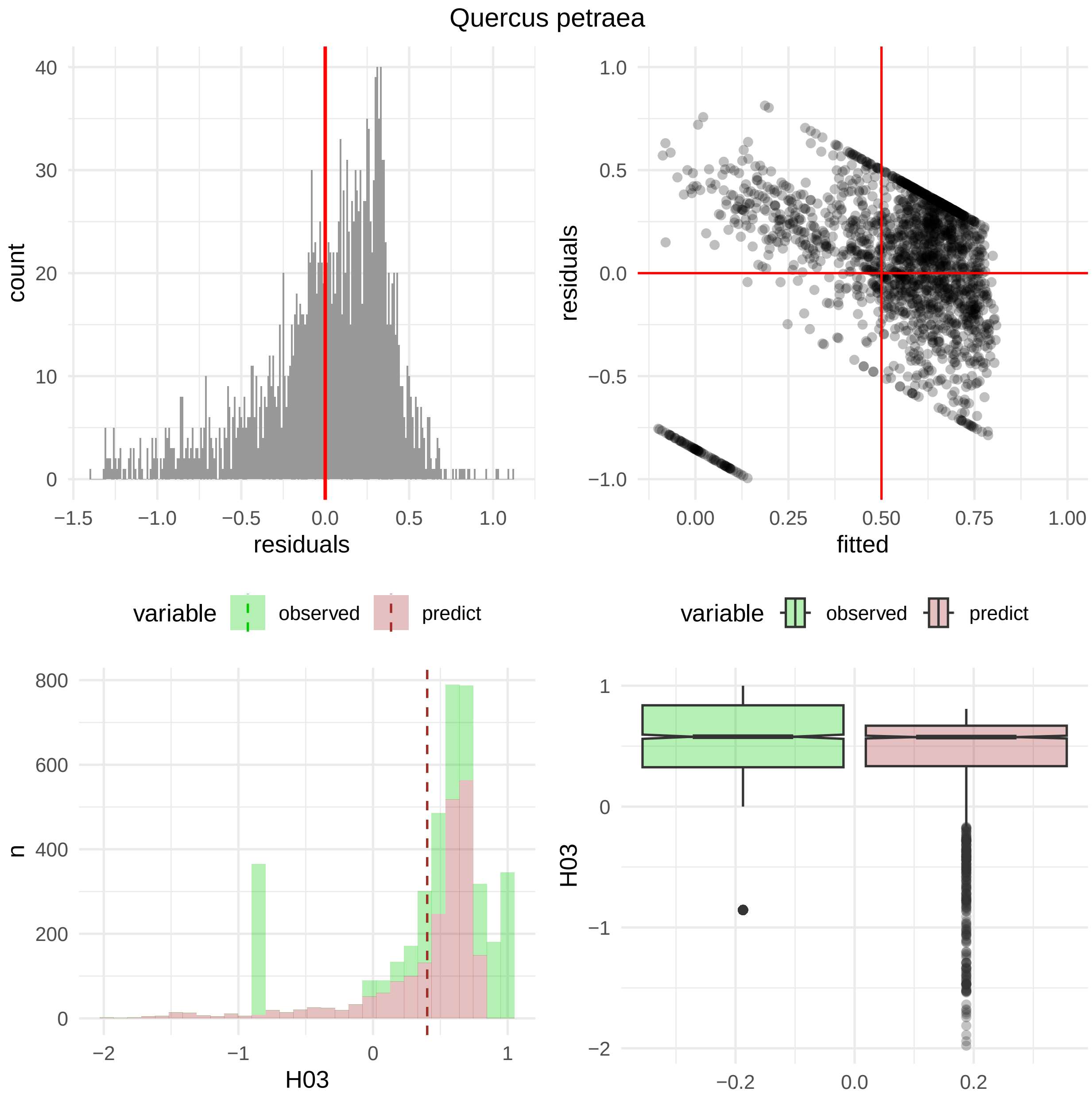

Correlation between predict and observed site index

Relationship between predicted and observed site index (density cloud), as well as linear regressions of presences and absences (= 'growth absences') (red line) and presences only (magenta line). The formulas, significance, R2 and number of observations are displayed below for both regressions. Ideally, both the point cloud and the regression lines lie close to the dashed line. For presences only we additionally calculated the correlation coefficient according to PEARSON (cor.pre) in the bottom right corner.

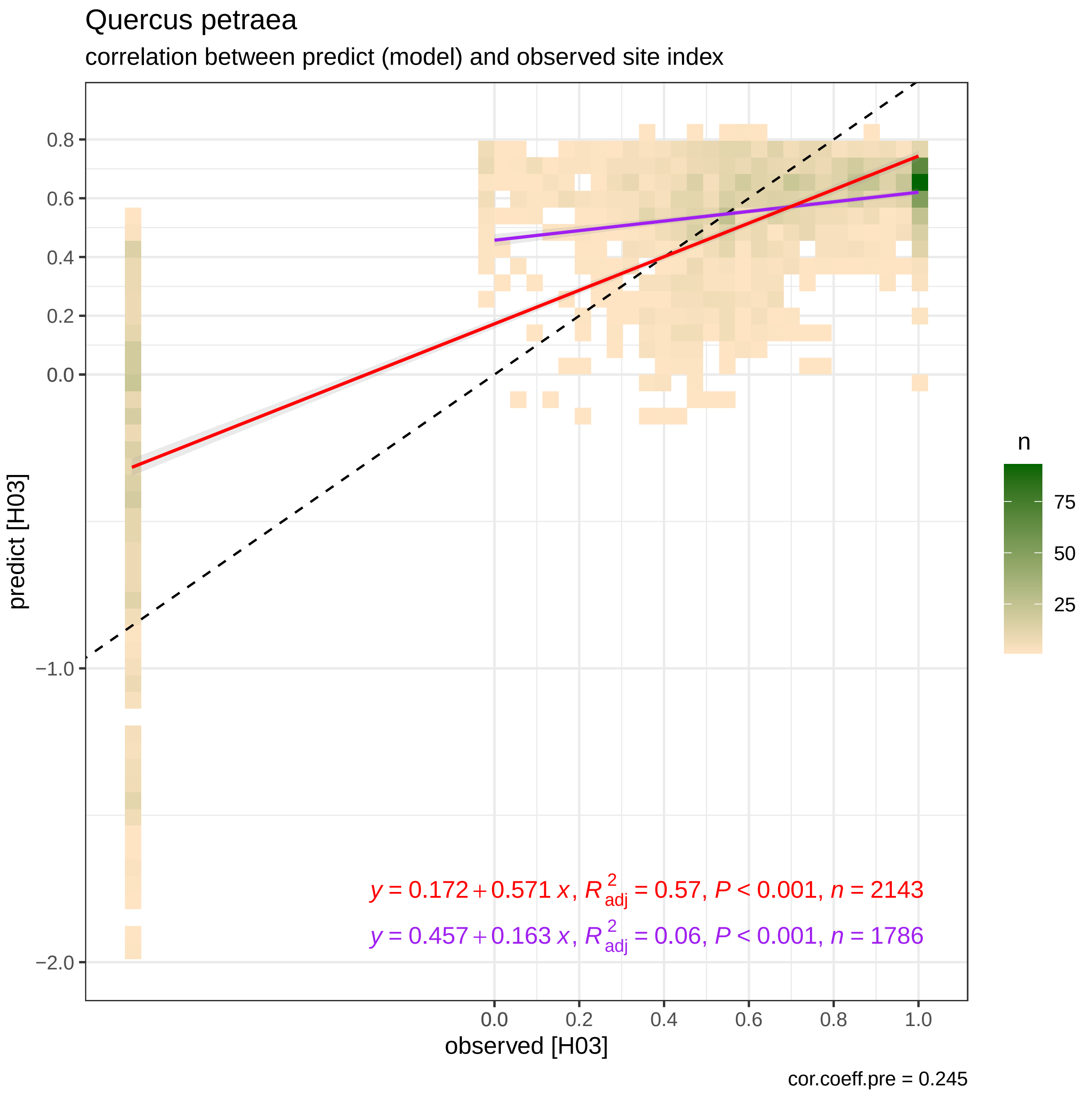

Predictions and forecasts

Predict

European predict for the reference period (1981 to 2010). Dark green symbolizes a high site index (tree height in meters at age 100), orange a lower site index and red no growth. Magenta-coloured dots represent inventory points with growth information, light blue dots are absences (= ‘growth absences’). Results were aggregated on 25 km x 25 km scale.

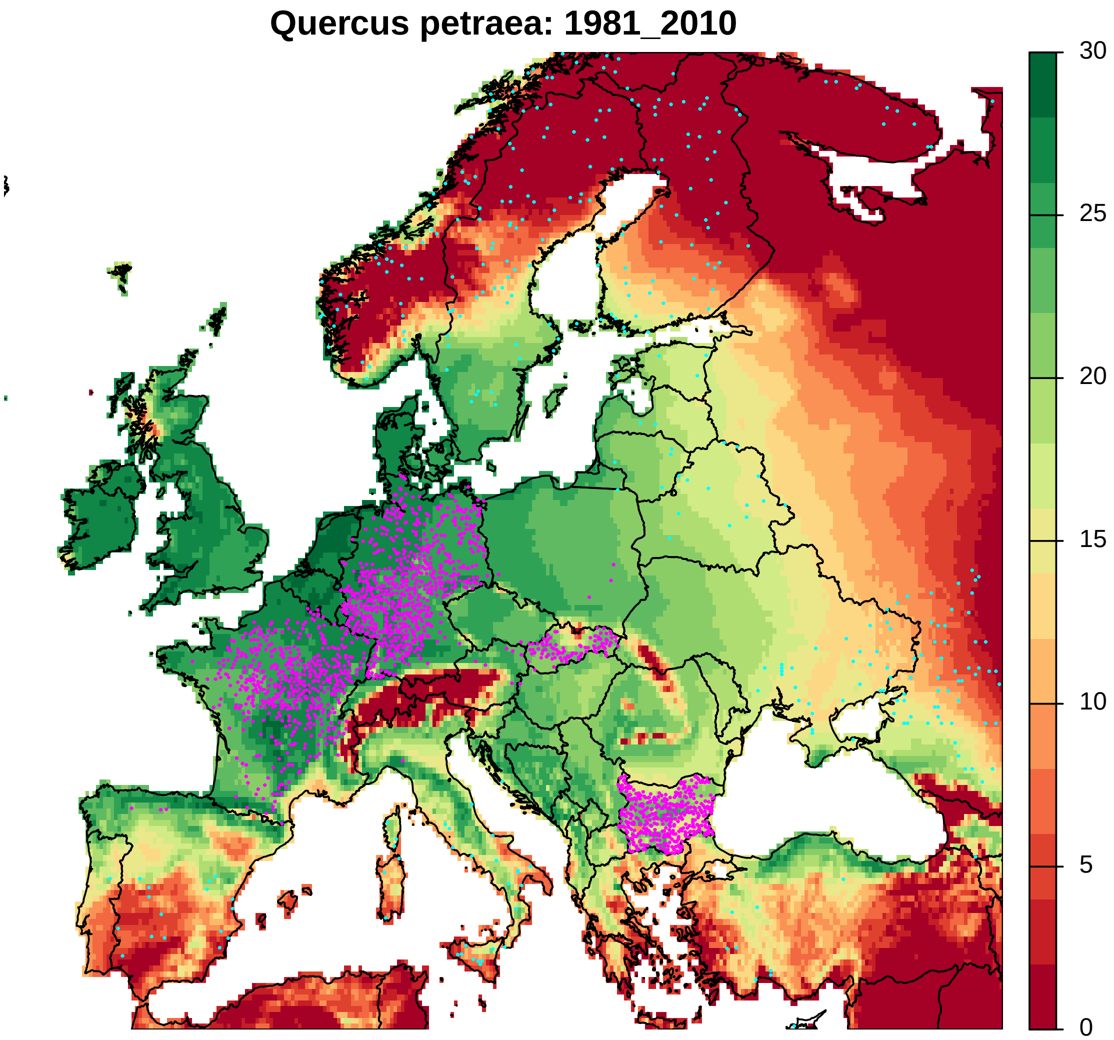

Forecast

Prediction for the reference period (1981 to 2010), as well as forecasts to 2071 to 2100 under szenario RCP4.5 and RCP8.5. Dark green symbolizes a high site index (tree height in m at age 100), orange a lower site index and red no growth. Results were aggregated on 25 km x 25 km scale.

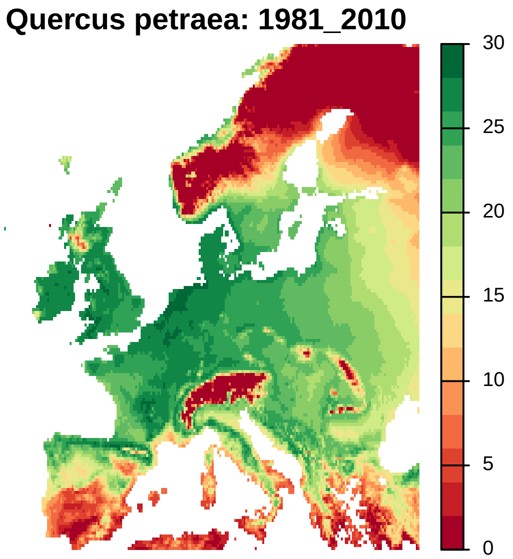

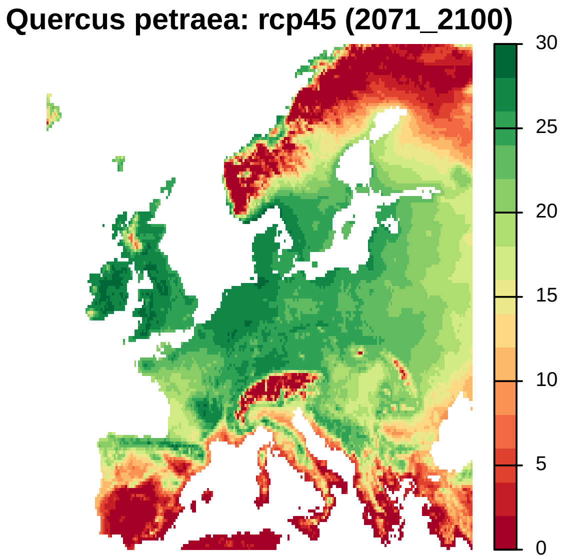

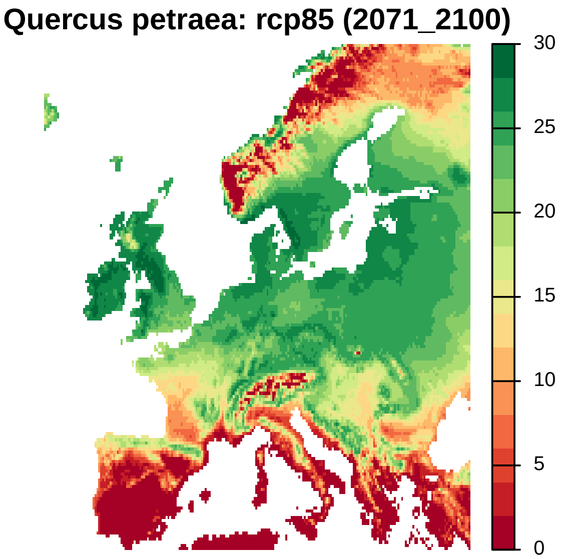

Quercus pubescens

Site index curves

Site index curves of Quercus pubescens created with non-linear quantile regressions based on the algorithm of Koenker and Park (1992). The site index (SI) was created by setting all points on the 95 percent quantile (upper line) and above to one (SI = 1) and all on the 5 percent quantile (lower line) and below to zero (SI = 0). The points between the quantile boundaries were assigned a site index between zero and one according to the ratio of their position between the quantile boundaries. We set selected absences (see chapter 2.1.3) on Height = 0 m (at age 100), which means, depending on the site index curves, for each tree species a SI near -1 (red line).

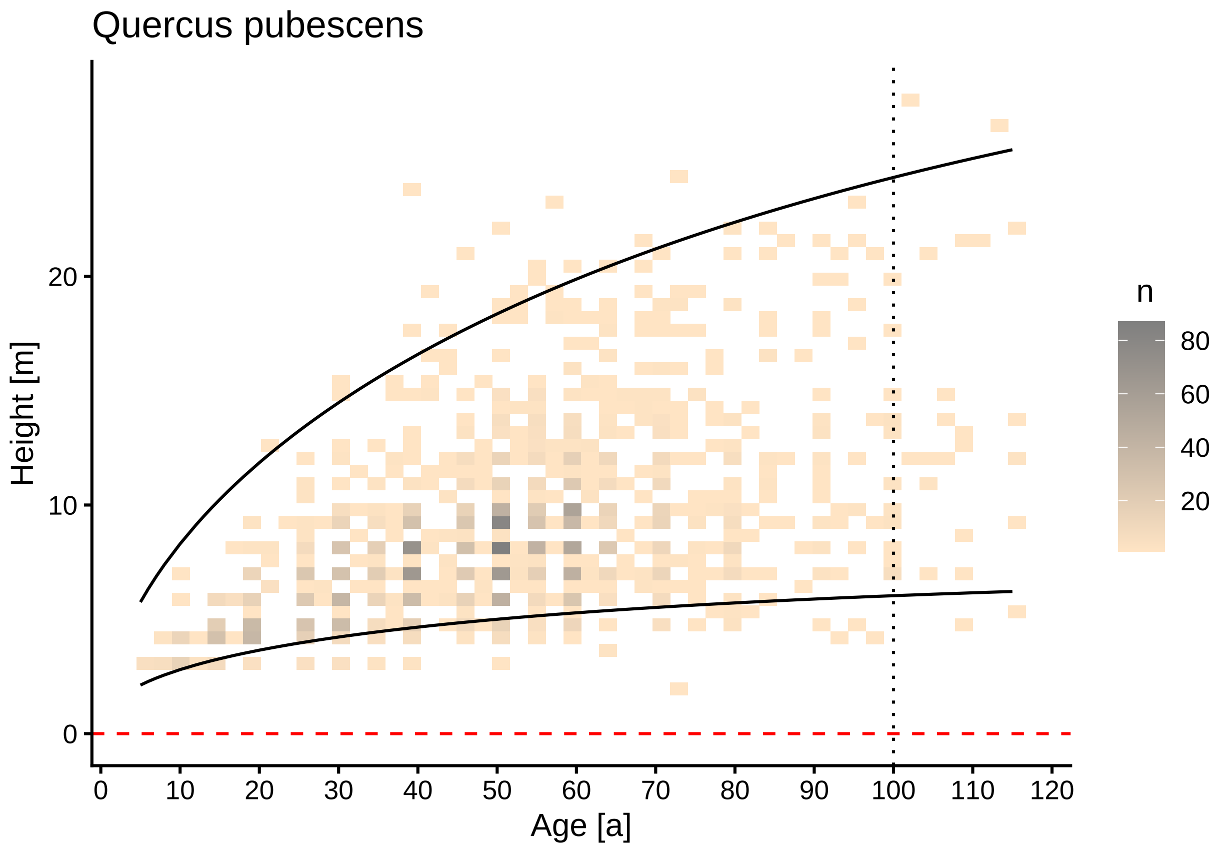

Model statistics and evaluation

Summary

Predictor acronyms: Bio.1 = Mean annual temperature [°C], Bio.12 = Annual precipitation sum [mm/m2], sp_p = Sum of precipitation [mm/m2] within months 3 to 5, su_p = Sum of precipitation [mm/m2] within months 6 to 8, wi_p = Sum of precipitation [mm/m2] within months 12,1,2, sp_t = Mean temperature [°C] within months 3 to 5, su_t = Mean temperature [°C] within months 6 to 8, wi_t = Mean temperature [°C] within months 12,1,2.

```
##
## Family: gaussian
## Link function: identity
##
## Formula:
## H03 ~ s(reference_19812010_wi_t, k = 3) + s(reference_19812010_su_p,
##     k = 3) + s(reference_19812010_sp_p, k = 3)
##
## Parametric coefficients:
##             Estimate Std. Error t value Pr(>|t|)
## (Intercept)  0.33056    0.01092   30.28   <2e-16 ***
## ---
## Signif. codes:  0 '***' 0.001 '**' 0.01 '*' 0.05 '.' 0.1 ' ' 1
##
## Approximate significance of smooth terms:
##                              edf Ref.df      F p-value
## s(reference_19812010_wi_t) 1.000  1.000 99.296  <2e-16 ***
## s(reference_19812010_su_p) 1.986  2.000 35.518  <2e-16 ***
## s(reference_19812010_sp_p) 1.634  1.866  3.585    0.06 .
## ---
## Signif. codes:  0 '***' 0.001 '**' 0.01 '*' 0.05 '.' 0.1 ' ' 1
##
## R-sq.(adj) =  0.448   Deviance explained = 45.2%
## -REML = 93.966  Scale est. = 0.07532   n = 632
```

Variance inflation factor (VIF)

Predictor acronyms: Bio.1 = Mean annual temperature [°C], Bio.12 = Annual precipitation sum [mm/m2], sp_p = Sum of precipitation [mm/m2] within months 3 to 5, su_p = Sum of precipitation [mm/m2] within months 6 to 8, wi_p = Sum of precipitation [mm/m2] within months 12,1,2, sp_t = Mean temperature [°C] within months 3 to 5, su_t = Mean temperature [°C] within months 6 to 8, wi_t = Mean temperature [°C] within months 12,1,2.

```
##                  Variables      VIF
## 1 reference_19812010_wi_t 3.327613
## 2 reference_19812010_su_p 3.407311
## 3 reference_19812010_sp_p 2.653727
```

Correlation matrix

Correlation matrix between the predictor variables and the target variable in the model. Correlation coefficient according to PEARSON. Predictor acronyms: Bio.1 = Mean annual temperature [°C], Bio.12 = Annual precipitation sum [mm/m2], sp_p = Sum of precipitation [mm/m2] within months 3 to 5, su_p = Sum of precipitation [mm/m2] within months 6 to 8, wi_p = Sum of precipitation [mm/m2] within months 12,1,2, sp_t = Mean temperature [°C] within months 3 to 5, su_t = Mean temperature [°C] within months 6 to 8, wi_t = Mean temperature [°C] within months 12,1,2.

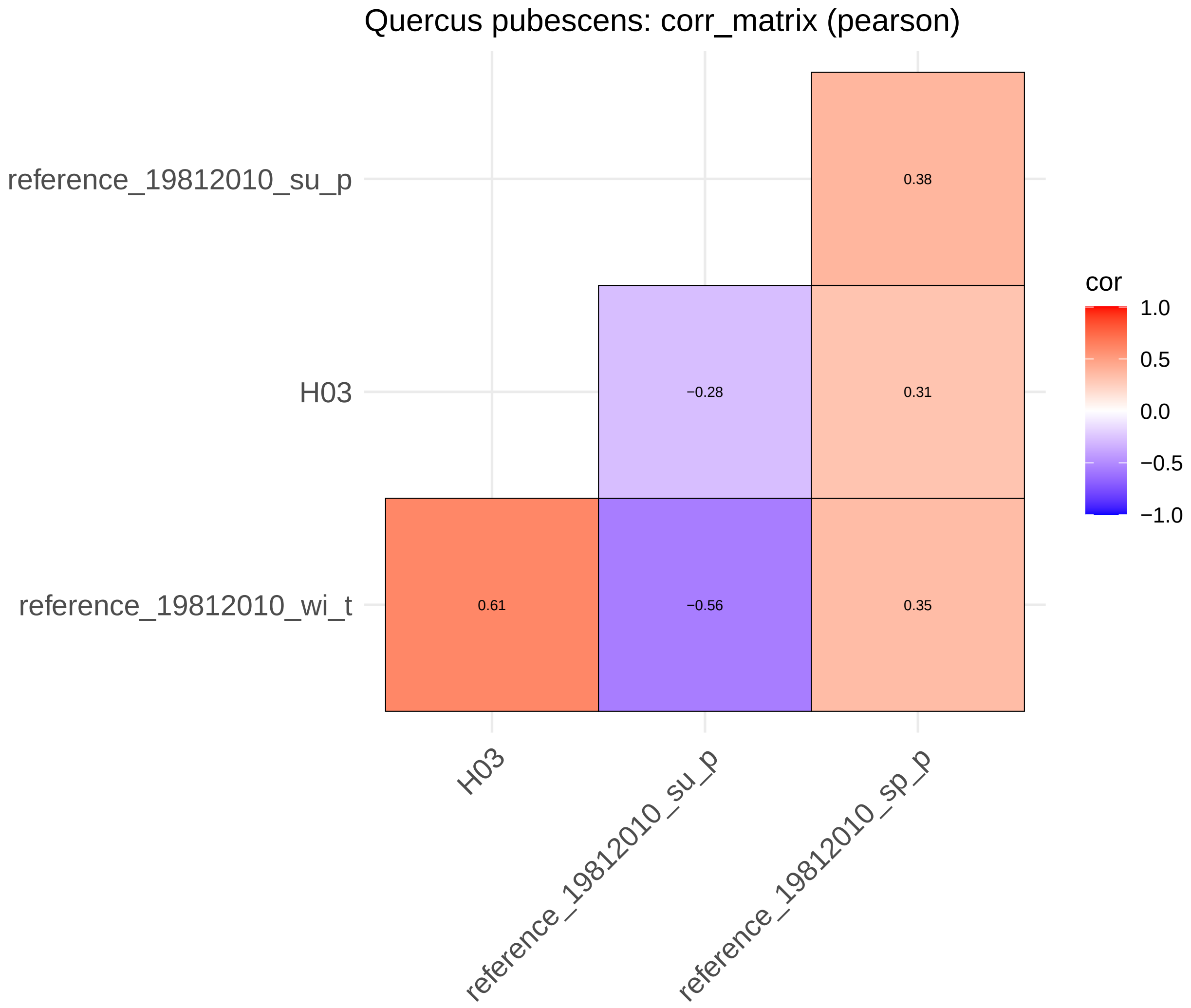

Response curves

Response curves (also known as effect curves) show how each predictor variable affects the target variable (H03 = european Site index, SIrel). H03 values below zero represent 'Growth absences'. Predictor acronyms: Bio.1 = Mean annual temperature [°C], Bio.12 = Annual precipitation sum [mm/m2], sp_p = Sum of precipitation [mm/m2] within months 3 to 5, su_p = Sum of precipitation [mm/m2] within months 6 to 8, wi_p = Sum of precipitation [mm/m2] within months 12,1,2, sp_t = Mean temperature [°C] within months 3 to 5, su_t = Mean temperature [°C] within months 6 to 8, wi_t = Mean temperature [°C] within months 12,1,2.

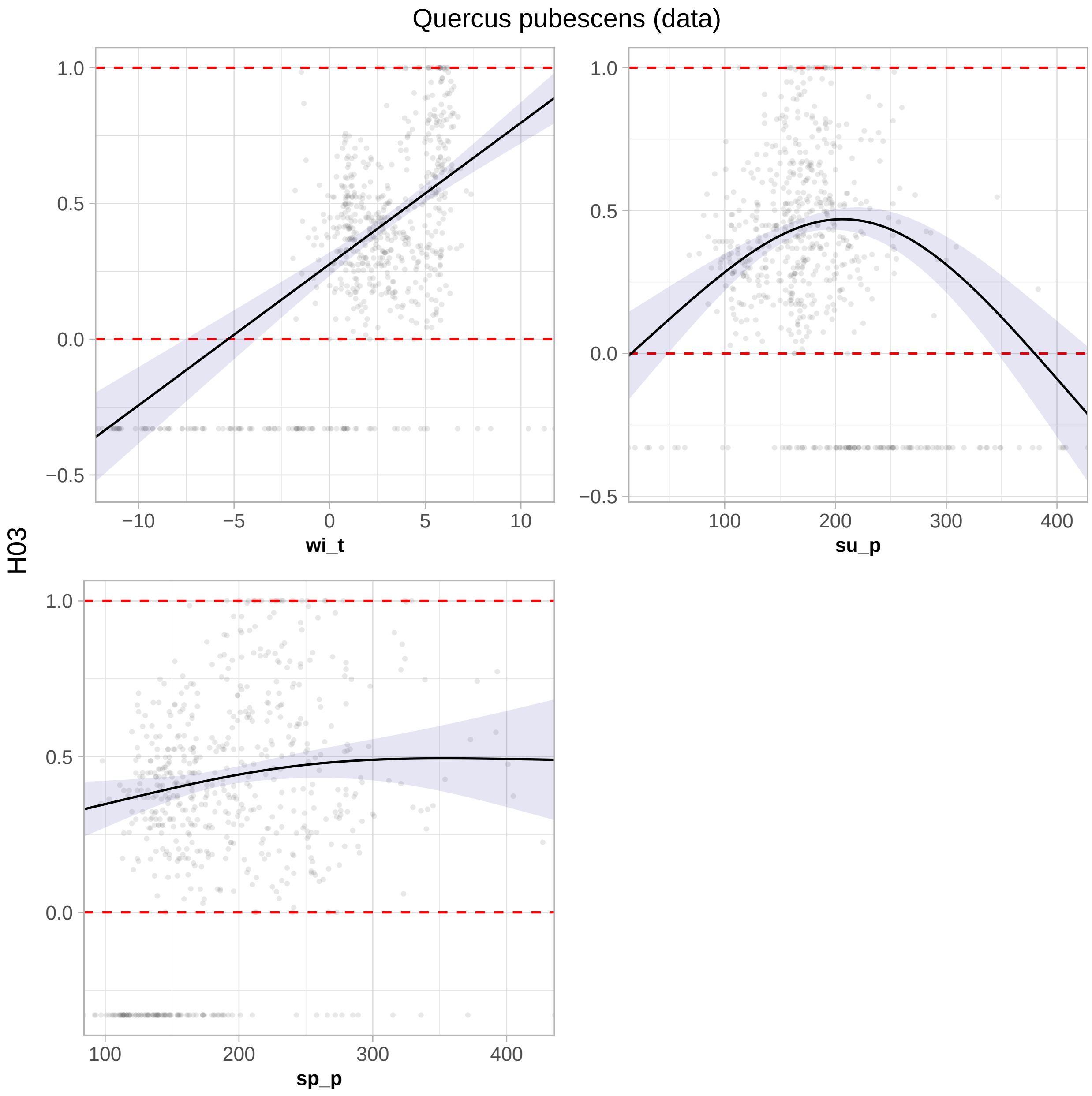

Response maps

Response maps (also referred as partial effect maps). Each map visualizes how a predictor affect the target variable (top height [m] at Age 100). Technically their work like response curves in a geographical area, that is setting all predictor variables except the one shown in the figure on their mean, and mapping the prediction. Predictor acronyms: Bio.1 = Mean annual temperature [°C], Bio.12 = Annual precipitation sum [mm/m2], sp_p = Sum of precipitation [mm/m2] within months 3 to 5, su_p = Sum of precipitation [mm/m2] within months 6 to 8, wi_p = Sum of precipitation [mm/m2] within months 12,1,2, sp_t = Mean temperature [°C] within months 3 to 5, su_t = Mean temperature [°C] within months 6 to 8, wi_t = Mean temperature [°C] within months 12,1,2.

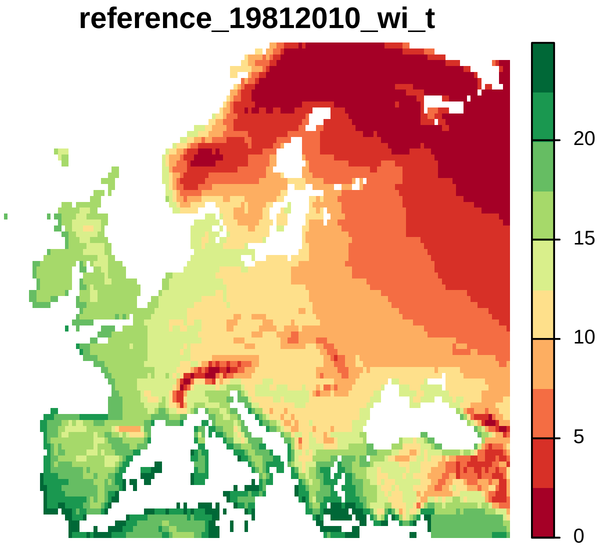

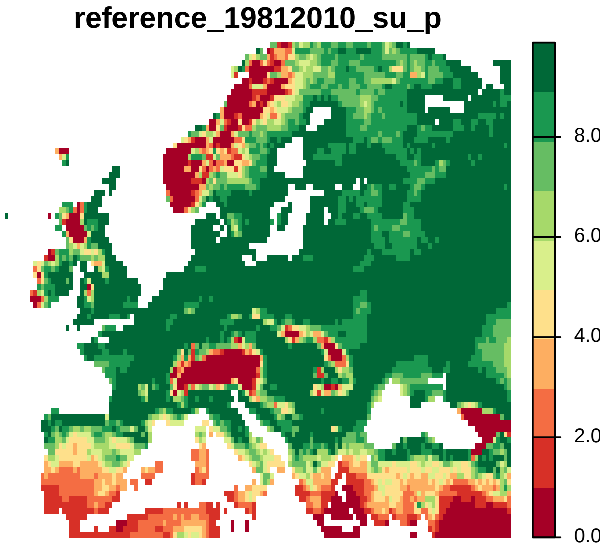

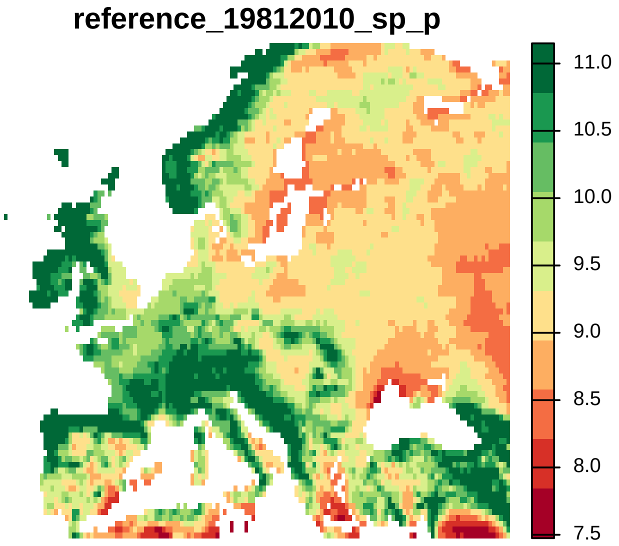

Residual distribution

The multi-panel plot includes a histogram of the residuals (top left), residuals over fitted values (top right), a histogram of observed and predicted values (bottom left) and boxplot diagram of observed and predicted values (bottom right). Observed values are shown in light green, while predicted ones are depicted in light red.

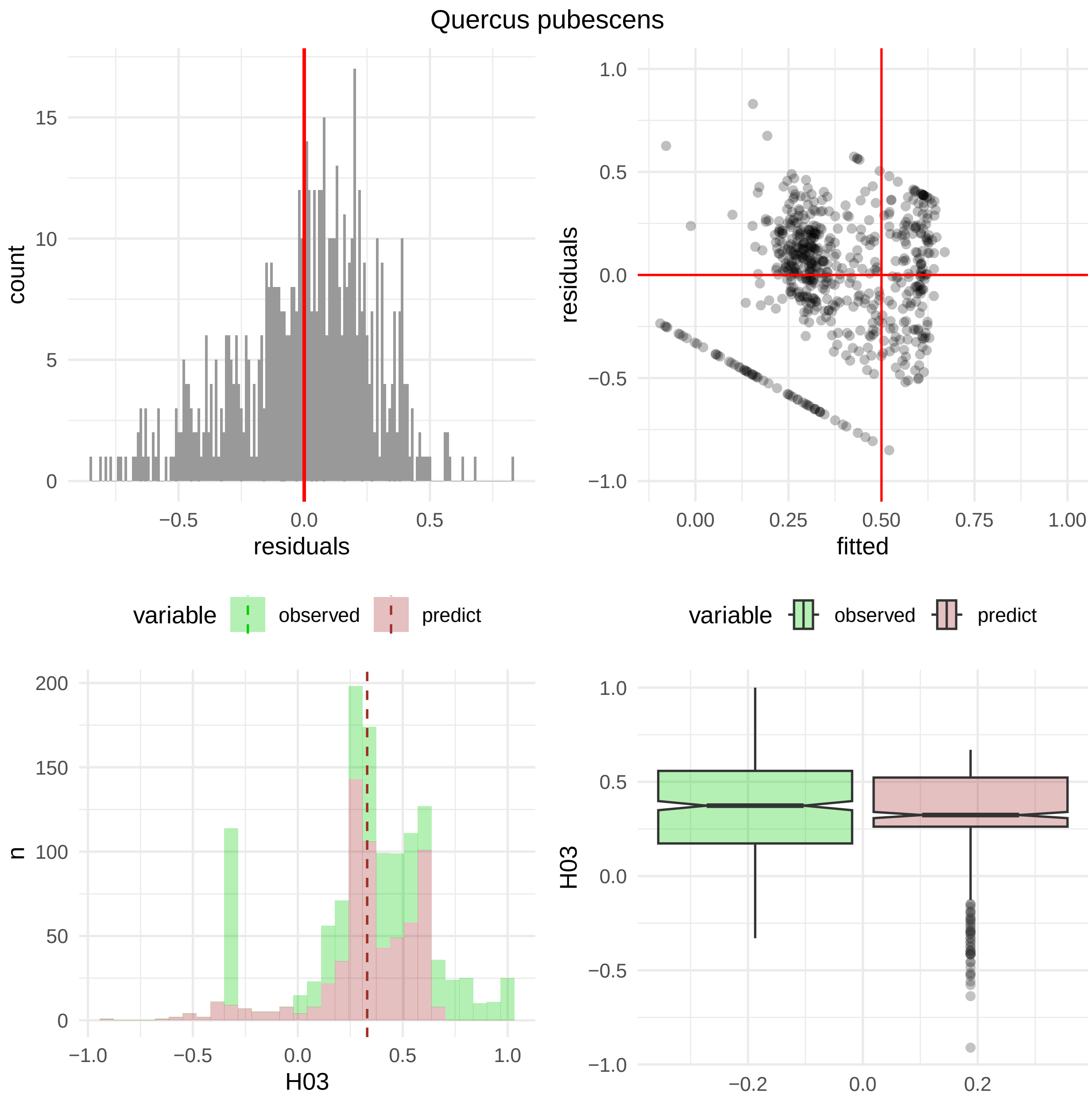

Correlation between predict and observed site index

Relationship between predicted and observed site index (density cloud), as well as linear regressions of presences and absences (= 'growth absences') (red line) and presences only (magenta line). The formulas, significance, R2 and number of observations are displayed below for both regressions. Ideally, both the point cloud and the regression lines lie close to the dashed line. For presences only we additionally calculated the correlation coefficient according to PEARSON (cor.pre) in the bottom right corner.

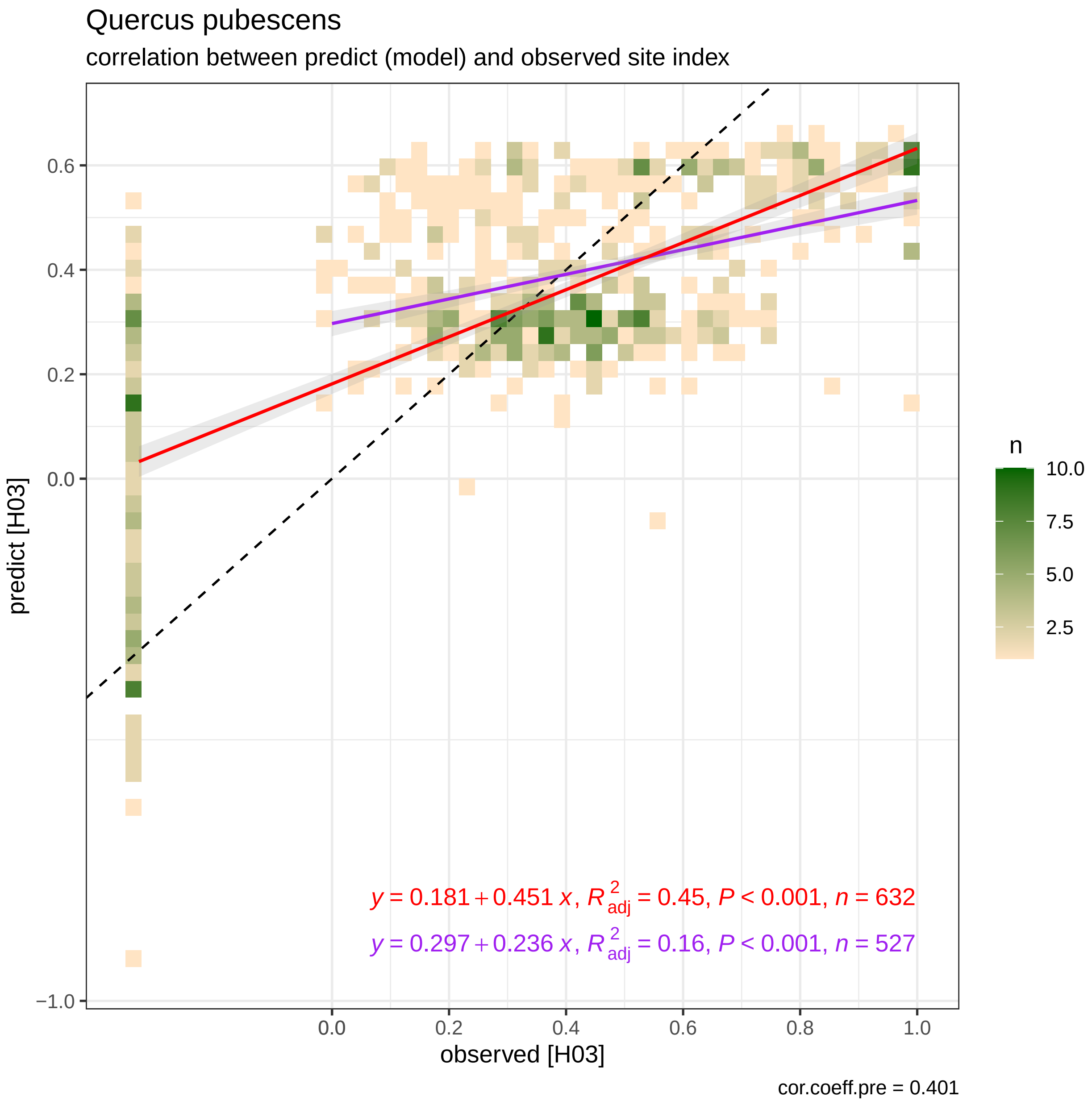

Predictions and forecasts

Predict

European predict for the reference period (1981 to 2010). Dark green symbolizes a high site index (tree height in meters at age 100), orange a lower site index and red no growth. Magenta-coloured dots represent inventory points with growth information, light blue dots are absences (= 'growth absences'). Results were aggregated on 25 km x 25 km scale.

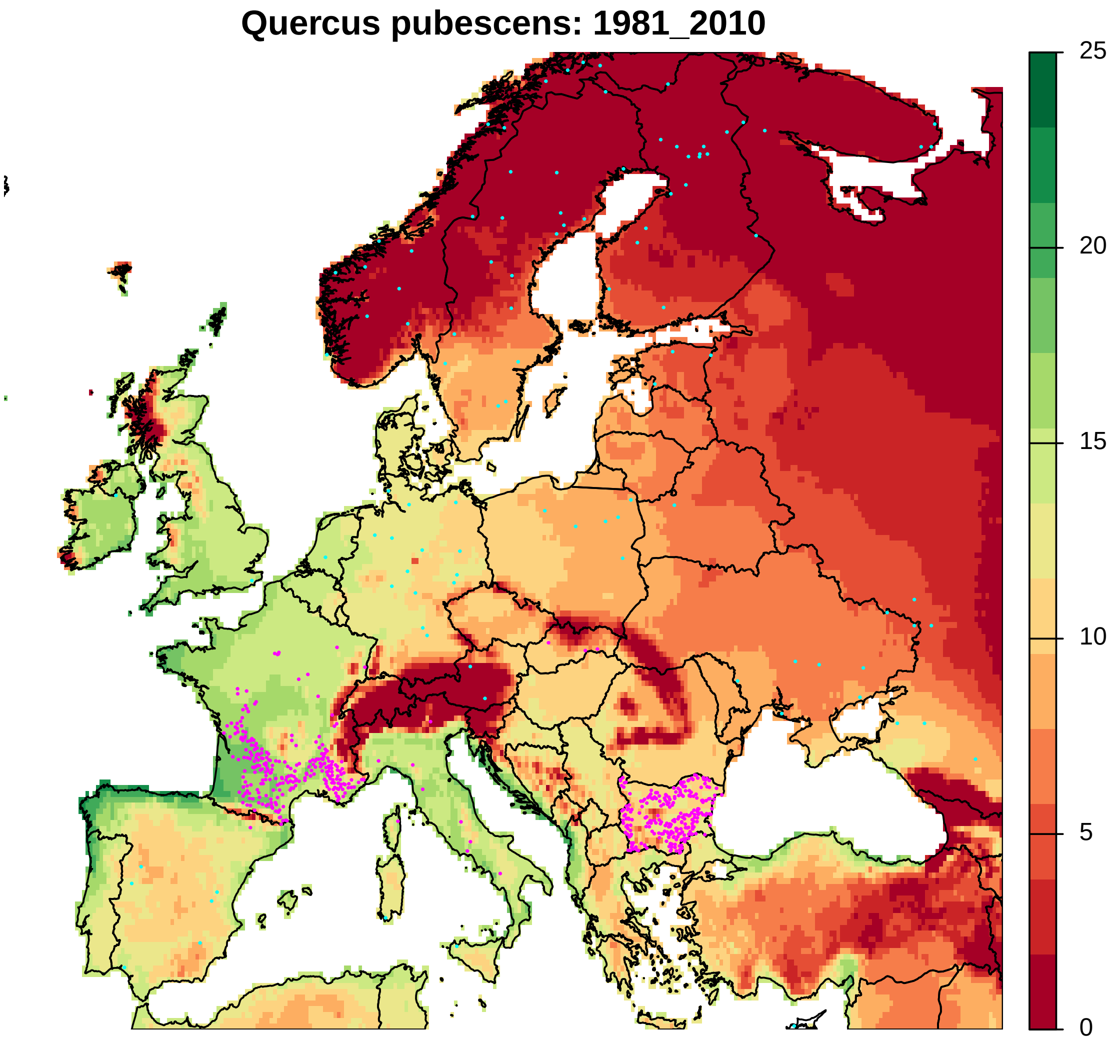

Forecast

Prediction for the reference period (1981 to 2010), as well as forecasts to 2071 to 2100 under szenario RCP4.5 and RCP8.5. Dark green symbolizes a high site index (tree height in m at age 100), orange a lower site index and red no growth. Results were aggregated on 25 km x 25 km scale.

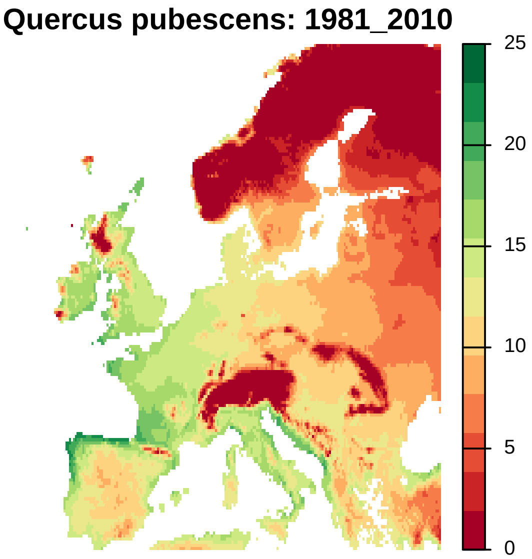

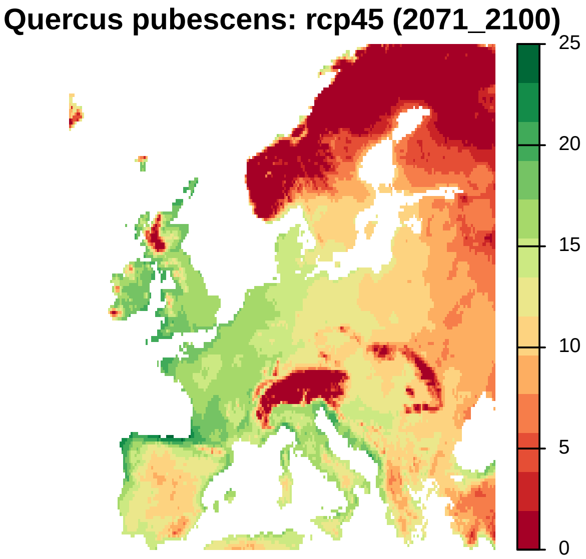

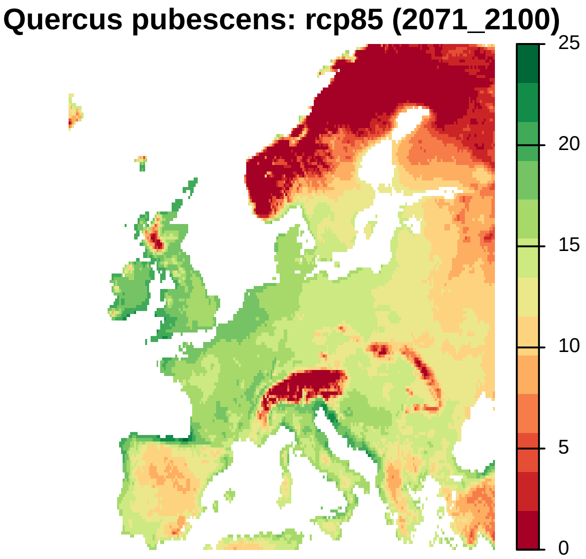

Quercus robur

Site index curves

Site index curves of Quercus robur created with non-linear quantile regressions based on the algorithm of Koenker and Park (1992). The site index (SI) was created by setting all points on the 95 percent quantile (upper line) and above to one (SI = 1) and all on the 5 percent quantile (lower line) and below to zero (SI = 0). The points between the quantile boundaries were assigned a site index between zero and one according to the ratio of their position between the quantile boundaries. We set selected absences (see chapter 2.1.3) on Height = 0 m (at age 100), which means, depending on the site index curves, for each tree species a SI near -1 (red line).

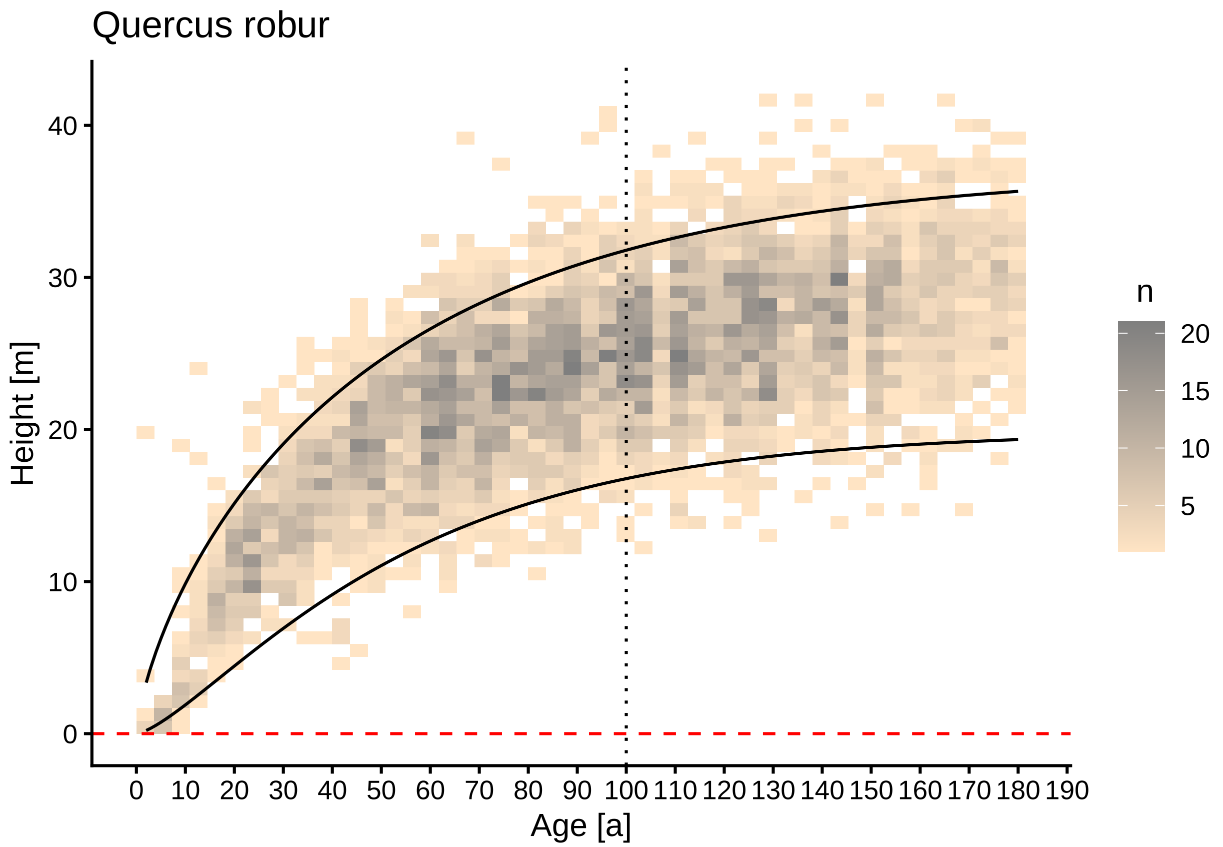

Model statistics and evaluation

Summary

Predictor acronyms: Bio.1 = Mean annual temperature [°C], Bio.12 = Annual precipitation sum [mm/m2], sp_p = Sum of precipitation [mm/m2] within months 3 to 5, su_p = Sum of precipitation [mm/m2] within months 6 to 8, wi_p = Sum of precipitation [mm/m2] within months 12,1,2, sp_t = Mean temperature [°C] within months 3 to 5, su_t = Mean temperature [°C] within months 6 to 8, wi_t = Mean temperature [°C] within months 12,1,2.

```
##
## Family: gaussian
## Link function: identity
##
## Formula:
## H03 ~ s(reference_19812010_su_t, k = 3) + s(reference_19812010_wi_t,
##     k = 3) + s(reference_19812010_su_p, k = 3)
##
## Parametric coefficients:
##             Estimate Std. Error t value Pr(>|t|)
## (Intercept) 0.358260   0.008691   41.22   <2e-16 ***
## ---
## Signif. codes:  0 '***' 0.001 '**' 0.01 '*' 0.05 '.' 0.1 ' ' 1
##
## Approximate significance of smooth terms:
##                              edf Ref.df     F p-value
## s(reference_19812010_su_t) 1.996      2 200.2  <2e-16 ***
## s(reference_19812010_wi_t) 1.996      2 311.2  <2e-16 ***
## s(reference_19812010_su_p) 1.993      2 105.7  <2e-16 ***
## ---
## Signif. codes:  0 '***' 0.001 '**' 0.01 '*' 0.05 '.' 0.1 ' ' 1
##
## R-sq.(adj) =  0.664   Deviance explained = 66.5%
## -REML = 1177.8  Scale est. = 0.16685   n = 2209
```

Variance inflation factor (VIF)

Predictor acronyms: Bio.1 = Mean annual temperature [°C], Bio.12 = Annual precipitation sum [mm/m2], sp_p = Sum of precipitation [mm/m2] within months 3 to 5, su_p = Sum of precipitation [mm/m2] within months 6 to 8, wi_p = Sum of precipitation [mm/m2] within months 12,1,2, sp_t = Mean temperature [°C] within months 3 to 5, su_t = Mean temperature [°C] within months 6 to 8, wi_t = Mean temperature [°C] within months 12,1,2.

```
##                   Variables      VIF
## 1 reference_19812010_su_t 2.281978
## 2 reference_19812010_wi_t 1.814770
## 3 reference_19812010_su_p 1.758395
```

Correlation matrix

Correlation matrix between the predictor variables and the target variable in the model. Correlation coefficient according to PEARSON. Predictor acronyms: Bio.1 = Mean annual temperature [°C], Bio.12 = Annual precipitation sum [mm/m2], sp_p = Sum of precipitation [mm/m2] within months 3 to 5, su_p = Sum of precipitation [mm/m2] within months 6 to 8, wi_p = Sum of precipitation [mm/m2] within months 12,1,2, sp_t = Mean temperature [°C] within months 3 to 5, su_t = Mean temperature [°C] within months 6 to 8, wi_t = Mean temperature [°C] within months 12,1,2.

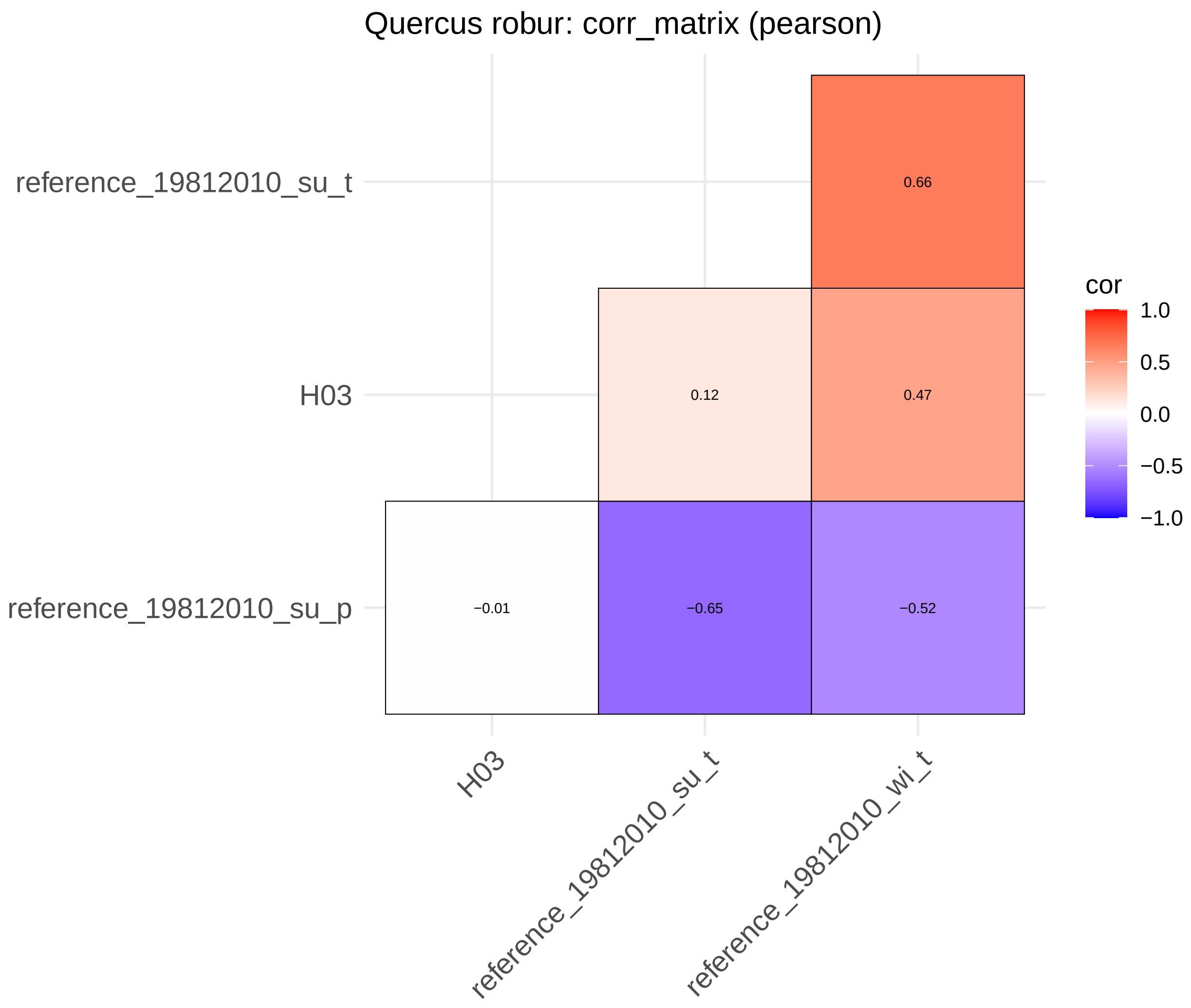

Response curves

Response curves (also known as effect curves) show how each predictor variable affects the target variable (H03 = european Site index, SIrel). H03 values below zero represent 'Growth absences'. Predictor acronyms: Bio.1 = Mean annual temperature [°C], Bio.12 = Annual precipitation sum [mm/m2], sp_p = Sum of precipitation [mm/m2] within months 3 to 5, su_p = Sum of precipitation [mm/m2] within months 6 to 8, wi_p = Sum of precipitation [mm/m2] within months 12,1,2, sp_t = Mean temperature [°C] within months 3 to 5, su_t = Mean temperature [°C] within months 6 to 8, wi_t = Mean temperature [°C] within months 12,1,2.

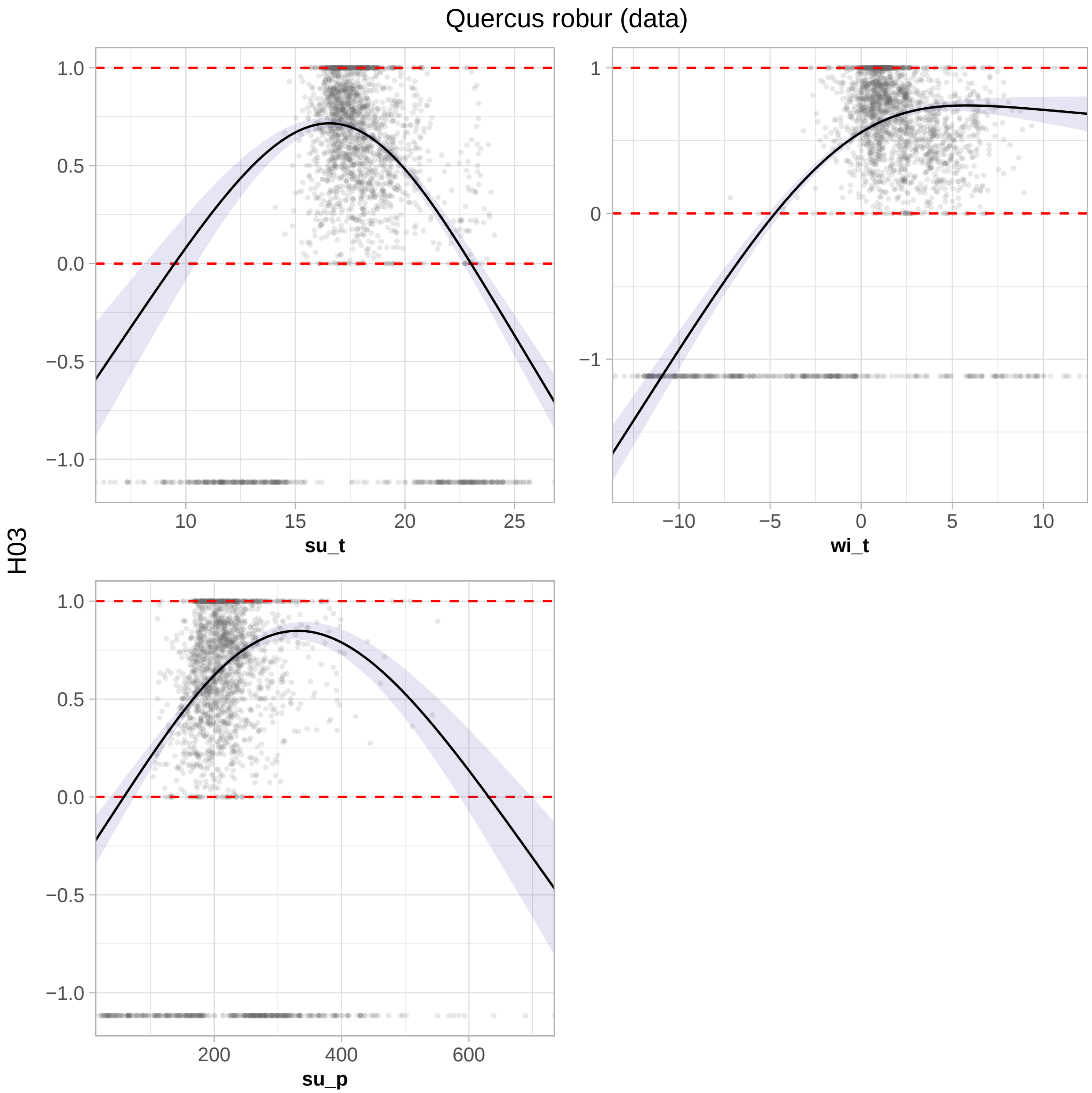

Response maps

Response maps (also referred as partial effect maps). Each map visualizes how a predictor affect the target variable (top height [m] at Age 100). Technically their work like response curves in a geographical area, that is setting all predictor variables except the one shown in the figure on their mean, and mapping the prediction. Predictor acronyms: Bio.1 = Mean annual temperature [°C], Bio.12 = Annual precipitation sum [mm/m2], sp_p = Sum of precipitation [mm/m2] within months 3 to 5, su_p = Sum of precipitation [mm/m2] within months 6 to 8, wi_p = Sum of precipitation [mm/m2] within months 12,1,2, sp_t = Mean temperature [°C] within months 3 to 5, su_t = Mean temperature [°C] within months 6 to 8, wi_t = Mean temperature [°C] within months 12,1,2.

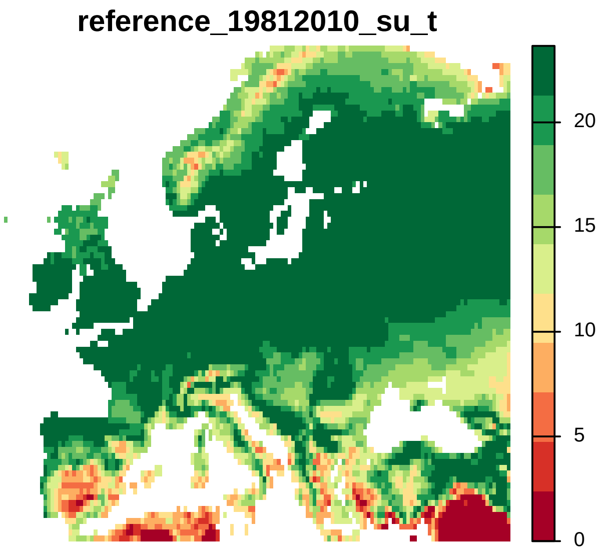

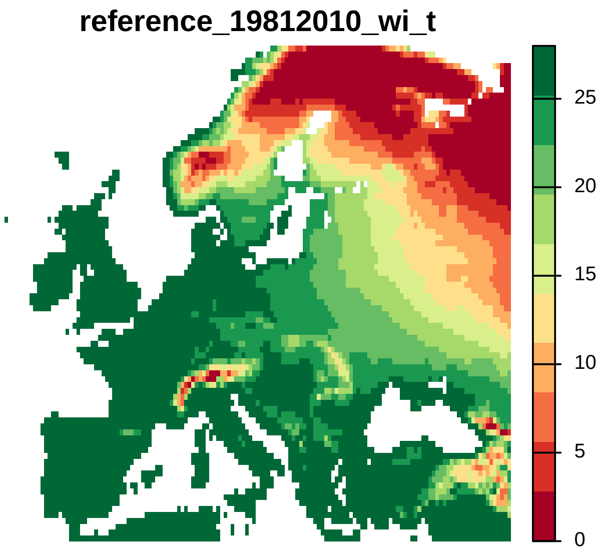

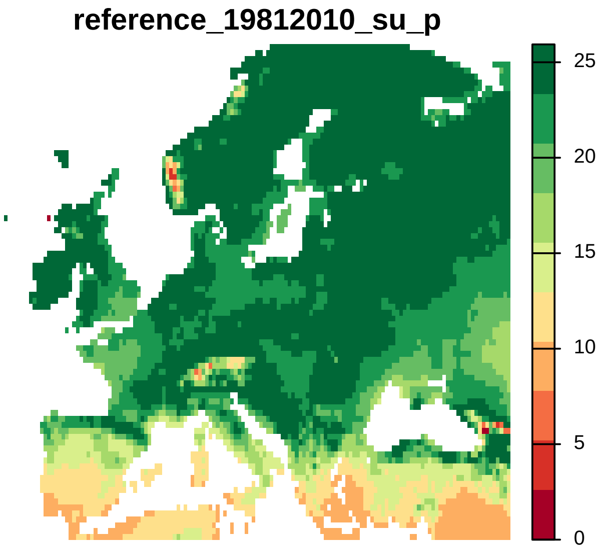

Residual distribution

The multi-panel plot includes a histogram of the residuals (top left), residuals over fitted values (top right), a histogram of observed and predicted values (bottom left) and boxplot diagram of observed and predicted values (bottom right). Observed values are shown in light green, while predicted ones are depicted in light red.

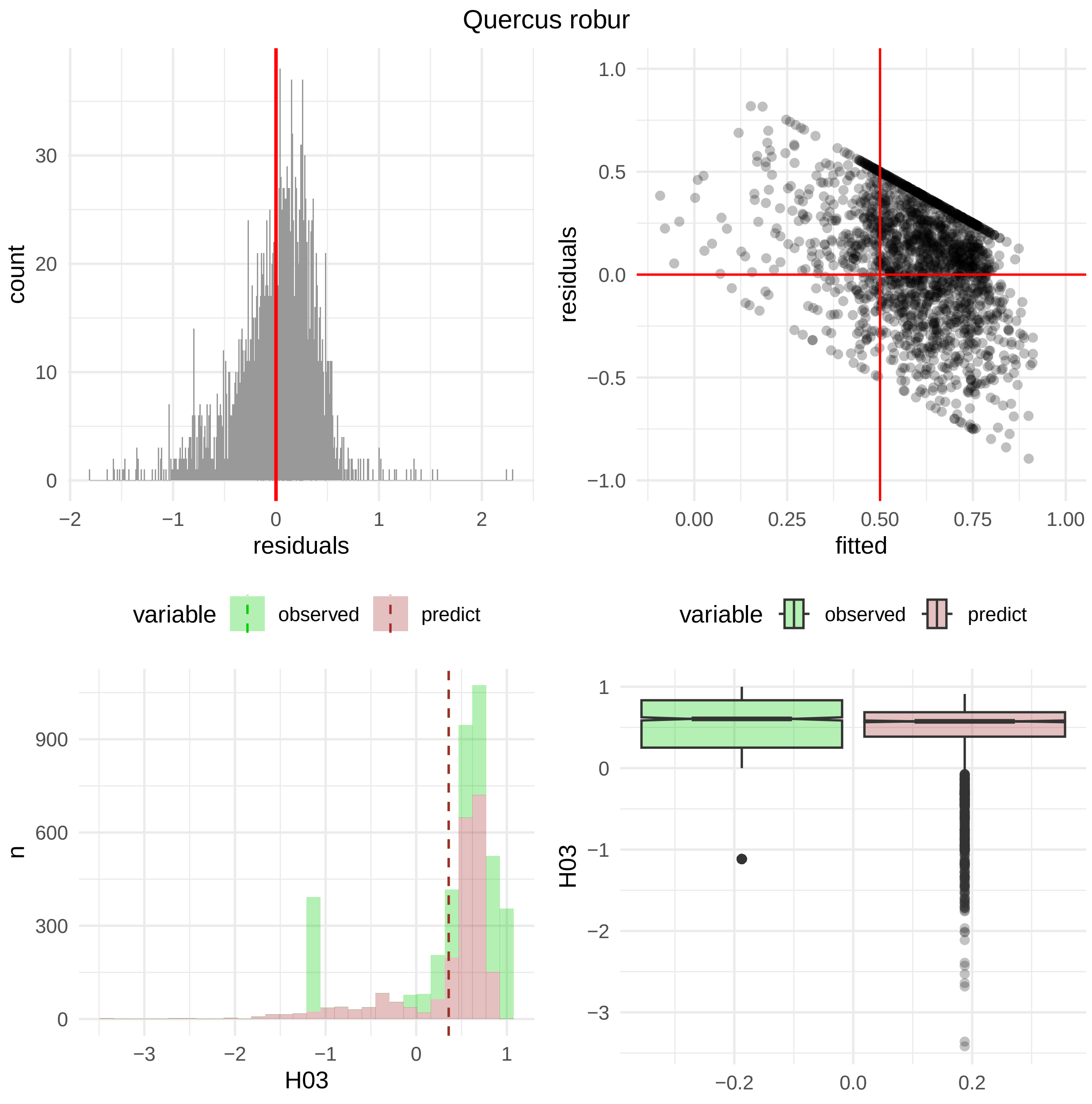

Correlation between predict and observed site index

Relationship between predicted and observed site index (density cloud), as well as linear regressions of presences and absences (= 'growth absences') (red line) and presences only (magenta line). The formulas, significance, R2 and number of observations are displayed below for both regressions. Ideally, both the point cloud and the regression lines lie close to the dashed line. For presences only we additionally calculated the correlation coefficient according to PEARSON (cor.pre) in the bottom right corner.

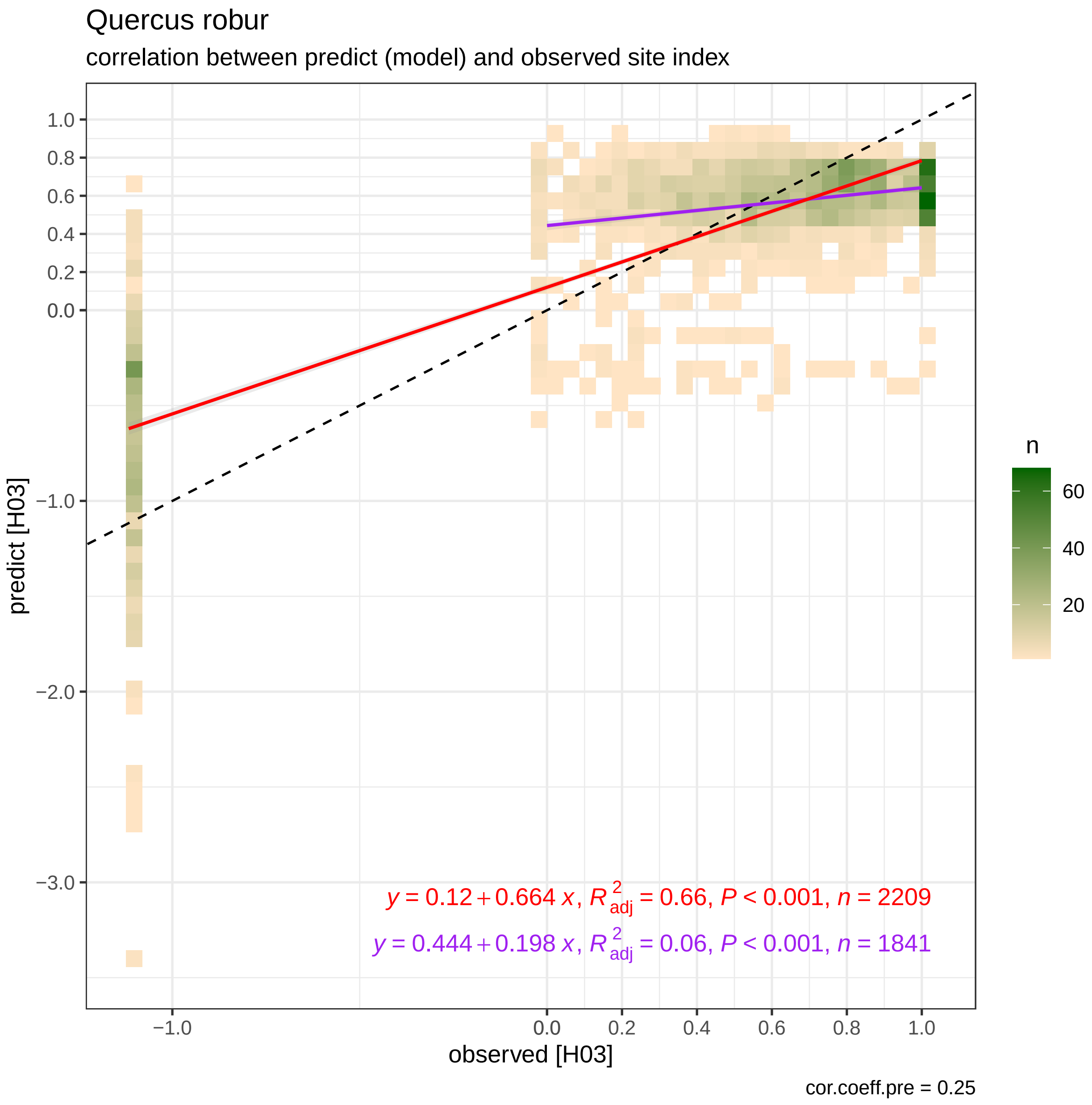

Predictions and forecasts

Predict

European predict for the reference period (1981 to 2010). Dark green symbolizes a high site index (tree height in meters at age 100), orange a lower site index and red no growth. Magenta-coloured dots represent inventory points with growth information, light blue dots are absences (= 'growth absences'). Results were aggregated on 25 km x 25 km scale.

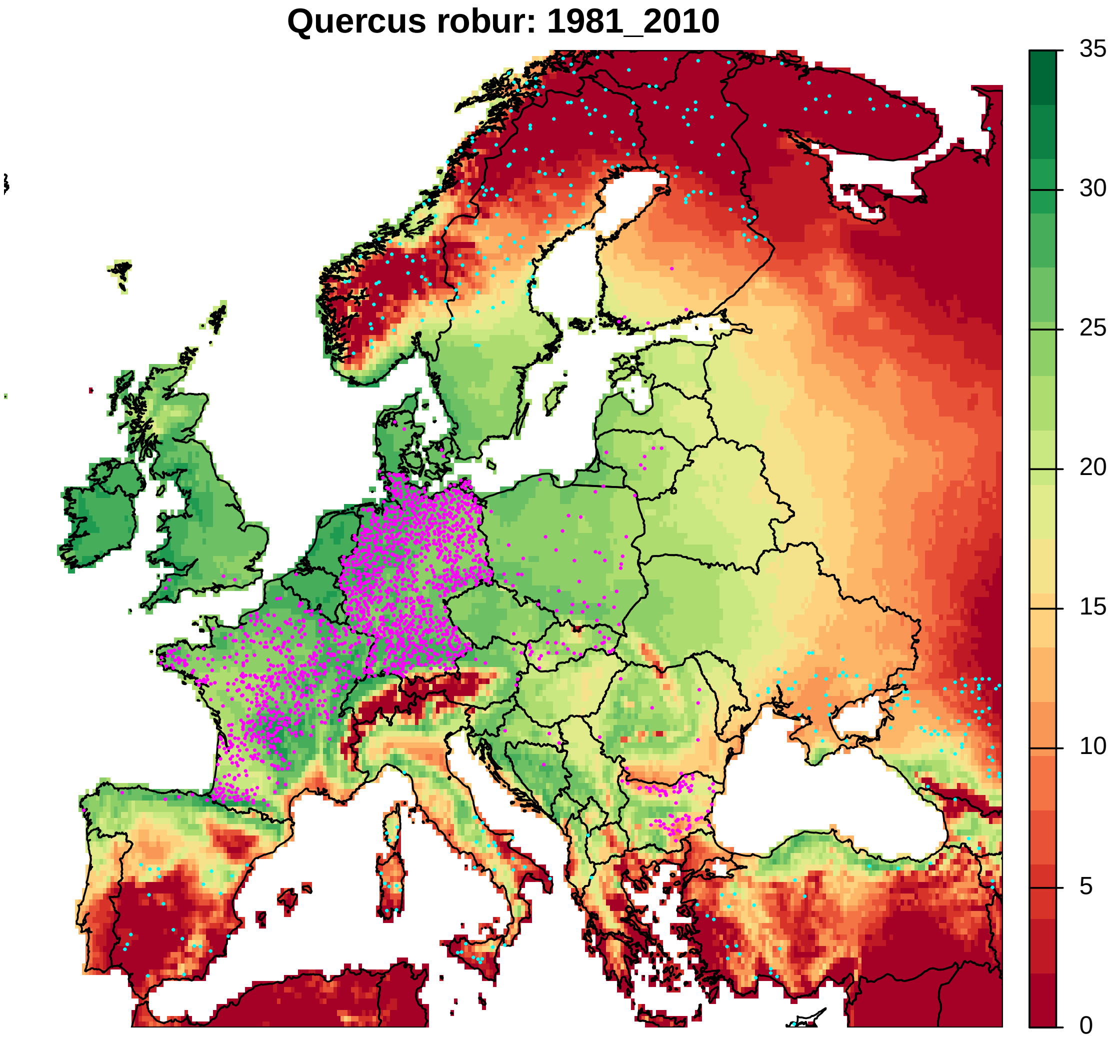

Forecast

Prediction for the reference period (1981 to 2010), as well as forecasts to 2071 to 2100 under szenario RCP4.5 and RCP8.5. Dark green symbolizes a high site index (tree height in m at age 100), orange a lower site index and red no growth. Results were aggregated on 25 km x 25 km scale.

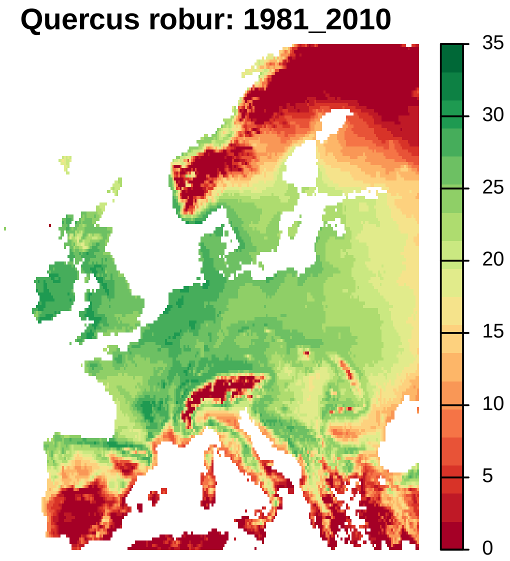

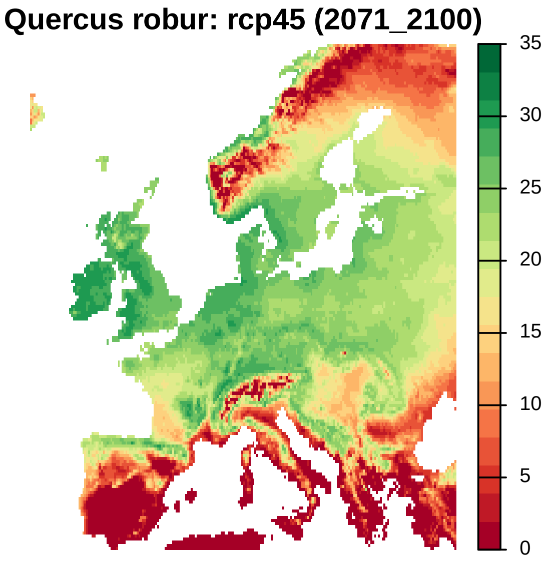

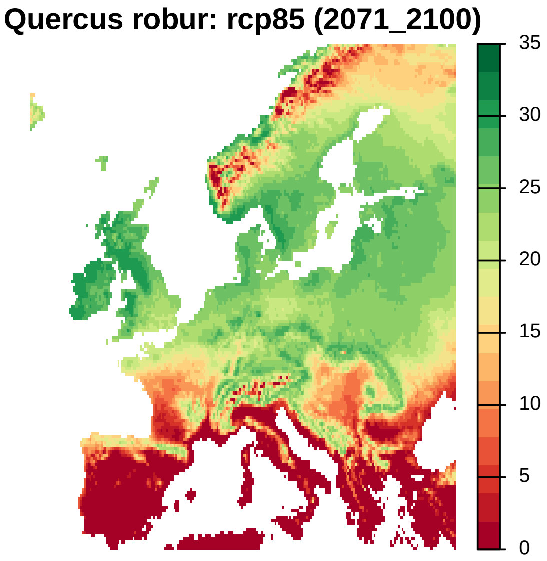

Quercus rubra

Site index curves

Site index curves of Quercus rubra created with non-linear quantile regressions based on the algorithm of Koenker and Park (1992). The site index (SI) was created by setting all points on the 95 percent quantile (upper line) and above to one (SI = 1) and all on the 5 percent quantile (lower line) and below to zero (SI = 0). The points between the quantile boundaries were assigned a site index between zero and one according to the ratio of their position between the quantile boundaries. We set selected absences (see chapter 2.1.3) on Height = 0 m (at age 100), which means, depending on the site index curves, for each tree species a SI near -1 (red line).

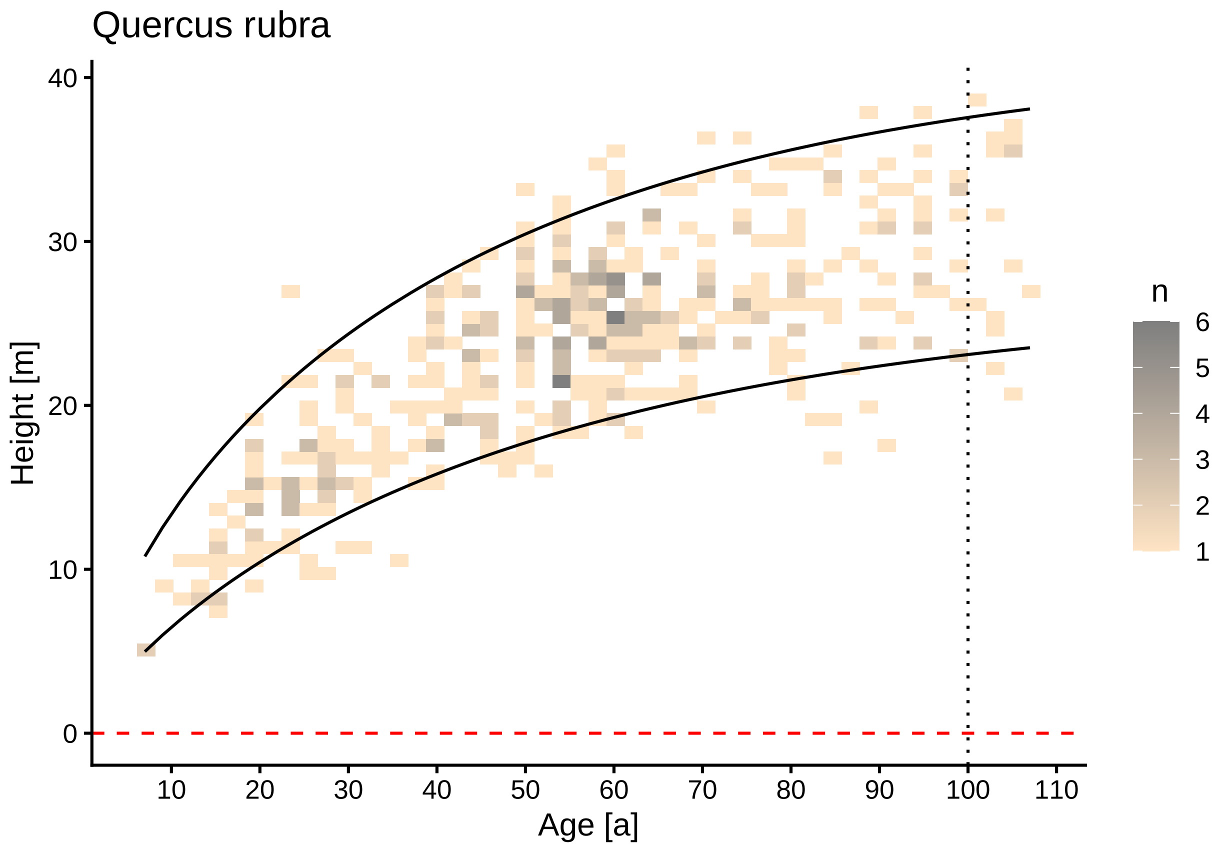

Model statistics and evaluation

Summary

Predictor acronyms: Bio.1 = Mean annual temperature [°C], Bio.12 = Annual precipitation sum [mm/m2], sp_p = Sum of precipitation [mm/m2] within months 3 to 5, su_p = Sum of precipitation [mm/m2] within months 6 to 8, wi_p = Sum of precipitation [mm/m2] within months 12,1,2, sp_t = Mean temperature [°C] within months 3 to 5, su_t = Mean temperature [°C] within months 6 to 8, wi_t = Mean temperature [°C] within months 12,1,2.

```
##
## Family: gaussian
## Link function: identity
##
## Formula:
## H03 ~ s(reference_19812010_su_t, k = 3) + s(reference_19812010_su_p,
##     k = 3) + s(reference_19812010_wi_t, k = 3)
##
## Parametric coefficients:
##             Estimate Std. Error t value Pr(>|t|)
## (Intercept)  0.12460    0.02509   4.966 1.02e-06 ***
## ---
## Signif. codes:  0 '***' 0.001 '**' 0.01 '*' 0.05 '.' 0.1 ' ' 1
##
## Approximate significance of smooth terms:
##                              edf Ref.df     F p-value
## s(reference_19812010_su_t) 1.969  1.998 22.57  <2e-16 ***
## s(reference_19812010_su_p) 1.964  1.998 21.94  <2e-16 ***
## s(reference_19812010_wi_t) 1.909  1.991 55.42  <2e-16 ***
## ---
## Signif. codes:  0 '***' 0.001 '**' 0.01 '*' 0.05 '.' 0.1 ' ' 1
##
## R-sq.(adj) =  0.619   Deviance explained = 62.5%
## -REML = 307.84  Scale est. = 0.25307   n = 402
```

Variance inflation factor (VIF)

Predictor acronyms: Bio.1 = Mean annual temperature [°C], Bio.12 = Annual precipitation sum [mm/m2], sp_p = Sum of precipitation [mm/m2] within months 3 to 5, su_p = Sum of precipitation [mm/m2] within months 6 to 8, wi_p = Sum of precipitation [mm/m2] within months 12,1,2, sp_t = Mean temperature [°C] within months 3 to 5, su_t = Mean temperature [°C] within months 6 to 8, wi_t = Mean temperature [°C] within months 12,1,2.

```
##                  Variables      VIF
## 1 reference_19812010_su_t 2.428293
## 2 reference_19812010_su_p 1.389882
## 3 reference_19812010_wi_t 1.974257
```

Correlation matrix

Correlation matrix between the predictor variables and the target variable in the model. Correlation coefficient according to PEARSON. Predictor acronyms: Bio.1 = Mean annual temperature [°C], Bio.12 = Annual precipitation sum [mm/m2], sp_p = Sum of precipitation [mm/m2] within months 3 to 5, su_p = Sum of precipitation [mm/m2] within months 6 to 8, wi_p = Sum of precipitation [mm/m2] within months 12,1,2, sp_t = Mean temperature [°C] within months 3 to 5, su_t = Mean temperature [°C] within months 6 to 8, wi_t = Mean temperature [°C] within months 12,1,2.

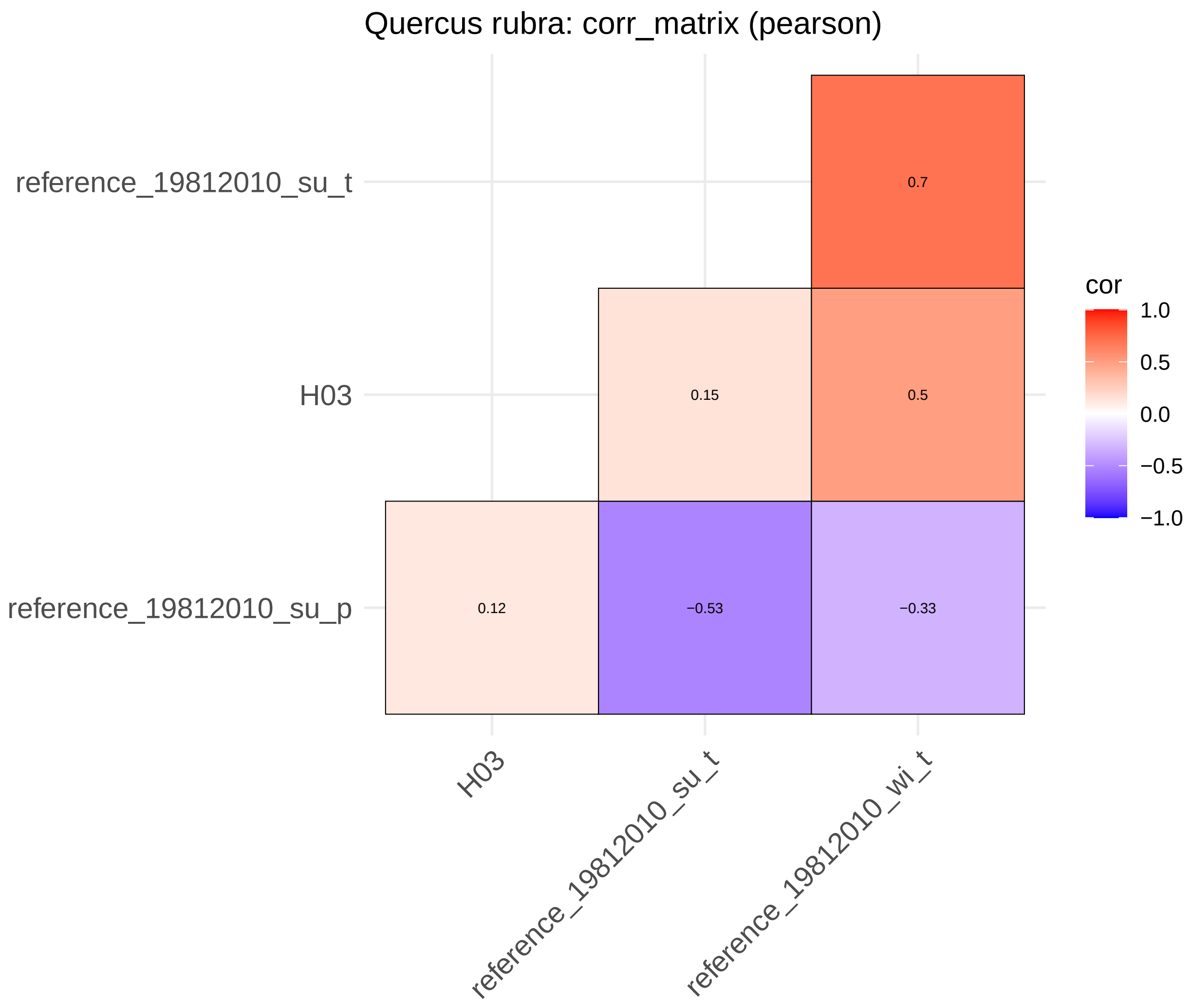

Response curves

Response curves (also known as effect curves) show how each predictor variable affects the target variable (H03 = european Site index, SIrel). H03 values below zero represent 'Growth absences'. Predictor acronyms: Bio.1 = Mean annual temperature [°C], Bio.12 = Annual precipitation sum [mm/m2], sp__p = Sum of precipitation [mm/m2] within months 3 to 5, su__p = Sum of precipitation [mm/m2] within months 6 to 8, wi__p = Sum of precipitation [mm/m2] within months 12,1,2, sp__t = Mean temperature [°C] within months 3 to 5, su__t = Mean temperature [°C] within months 6 to 8, wi__t = Mean temperature [°C] within months 12,1,2.

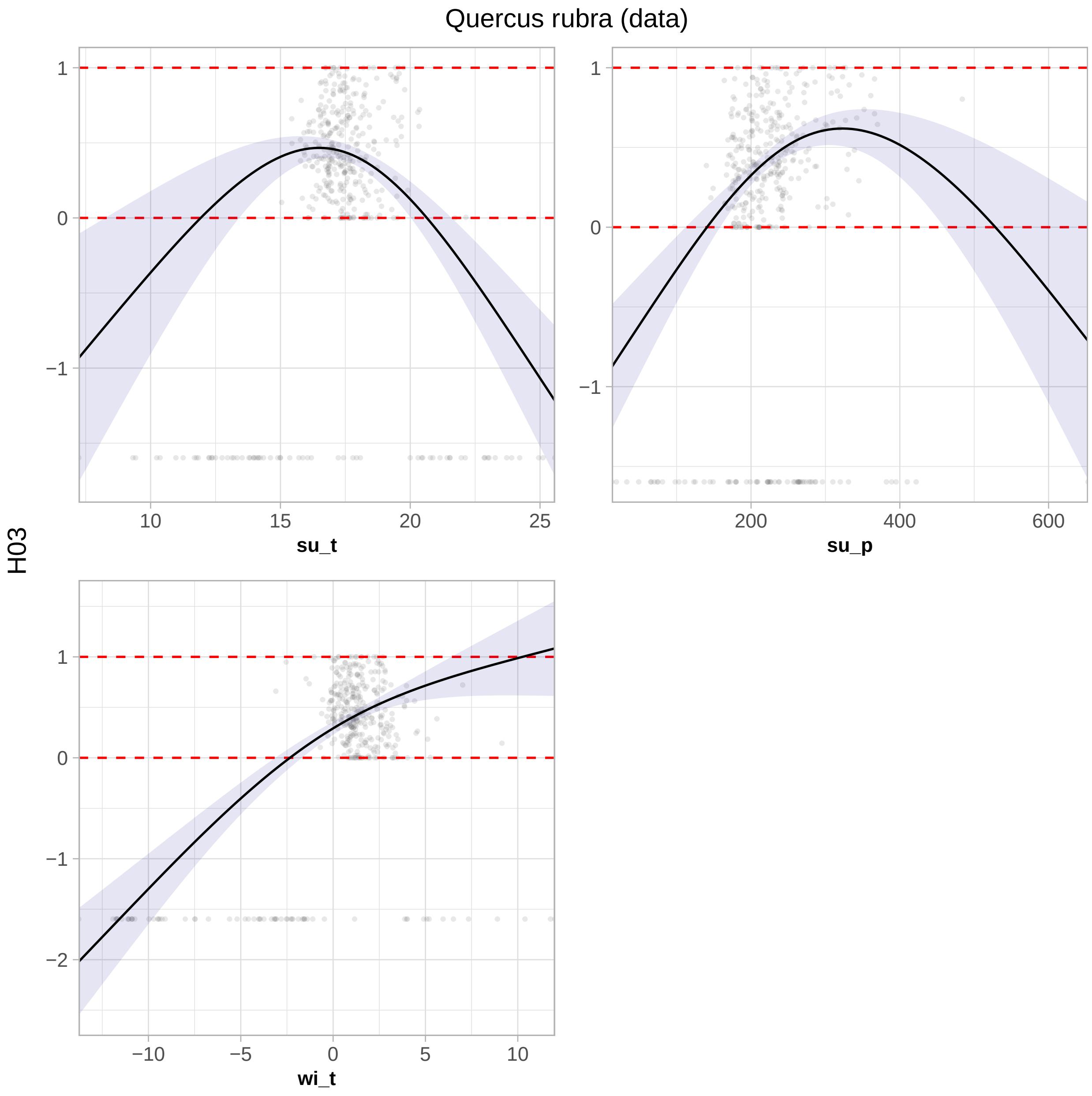

Response maps

Response maps (also referred as partial effect maps). Each map visualizes how a predictor affect the target variable (top height [m] at Age 100). Technically their work like response curves in a geographical area, that is setting all predictor variables except the one shown in the figure on their mean, and mapping the prediction. Predictor acronyms: Bio.1 = Mean annual temperature [°C], Bio.12 = Annual precipitation sum [mm/m2], sp_p = Sum of precipitation [mm/m2] within months 3 to 5, su_p = Sum of precipitation [mm/m2] within months 6 to 8, wi_p = Sum of precipitation [mm/m2] within months 12,1,2, sp_t = Mean temperature [°C] within months 3 to 5, su_t = Mean temperature [°C] within months 6 to 8, wi_t = Mean temperature [°C] within months 12,1,2.

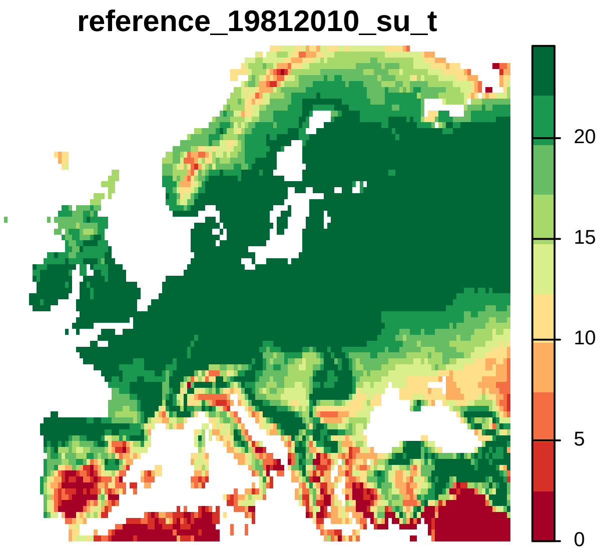

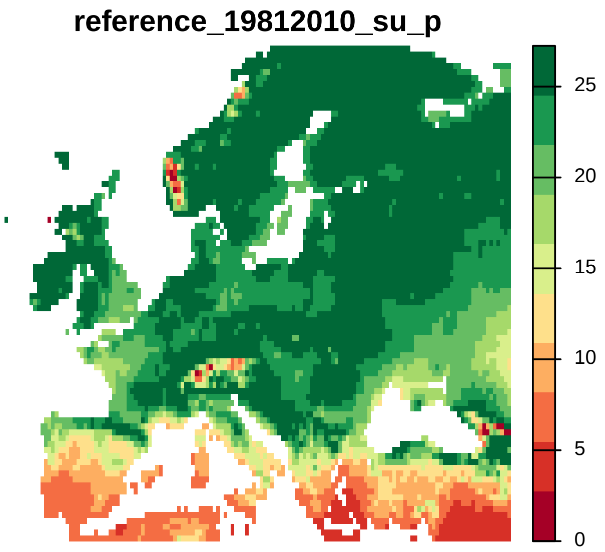

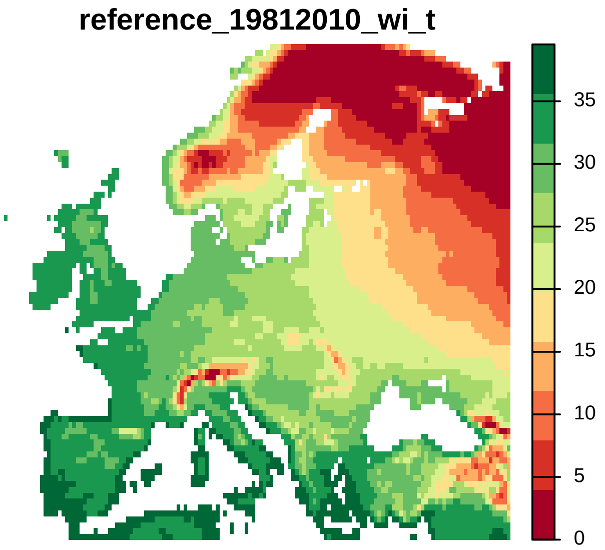

Residual distribution

The multi-panel plot includes a histogram of the residuals (top left), residuals over fitted values (top right), a histogram of observed and predicted values (bottom left) and boxplot diagram of observed and predicted values (bottom right). Observed values are shown in light green, while predicted ones are depicted in light red.

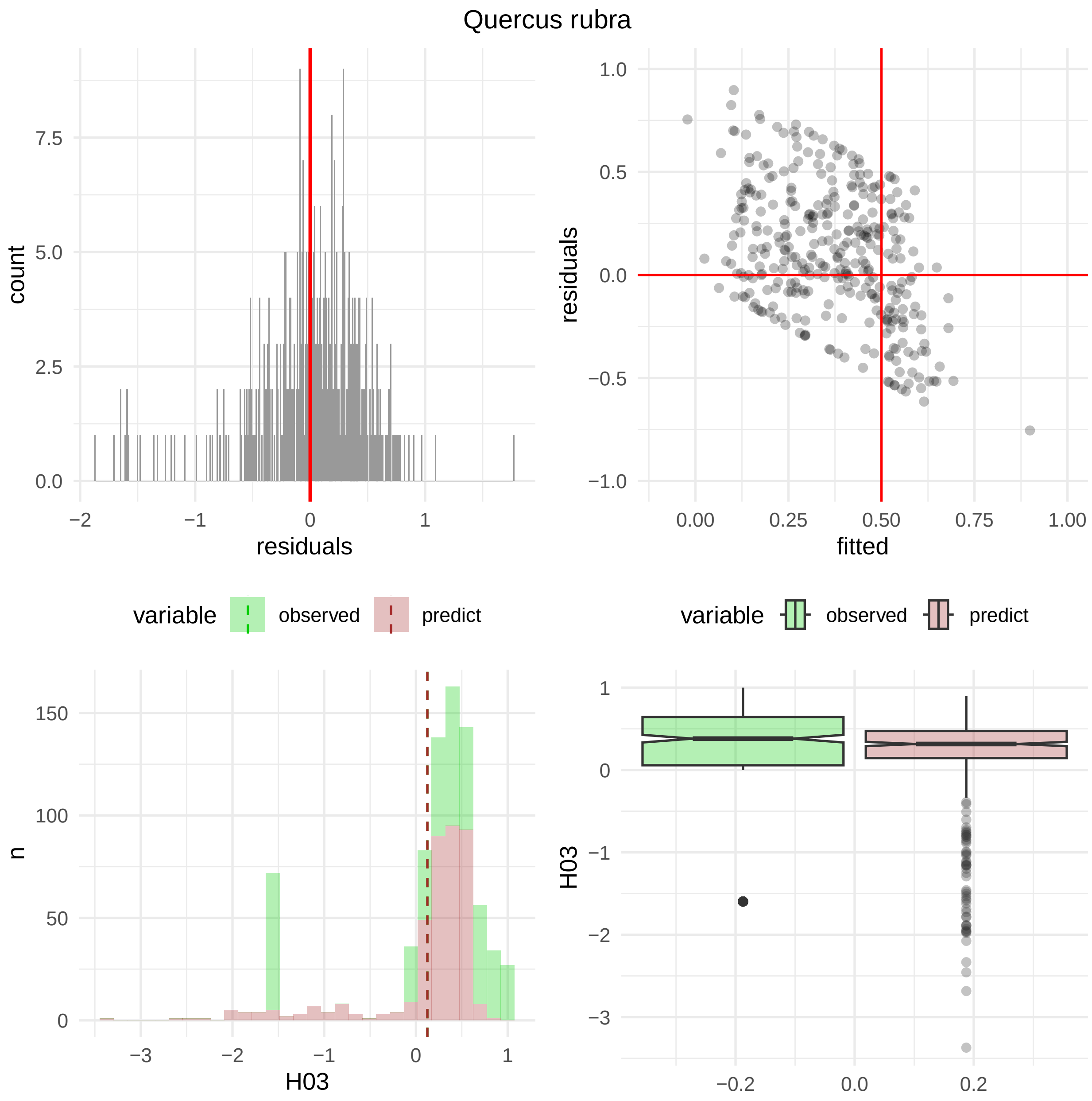

Correlation between predict and observed site index

Relationship between predicted and observed site index (density cloud), as well as linear regressions of presences and absences (= ‘growth absences’) (red line) and presences only (magenta line). The formulas, significance, R2 and number of observations are displayed below for both regressions. Ideally, both the point cloud and the regression lines lie close to the dashed line. For presences only we additionally calculated the correlation coefficient according to PEARSON (cor.pre) in the bottom right corner.

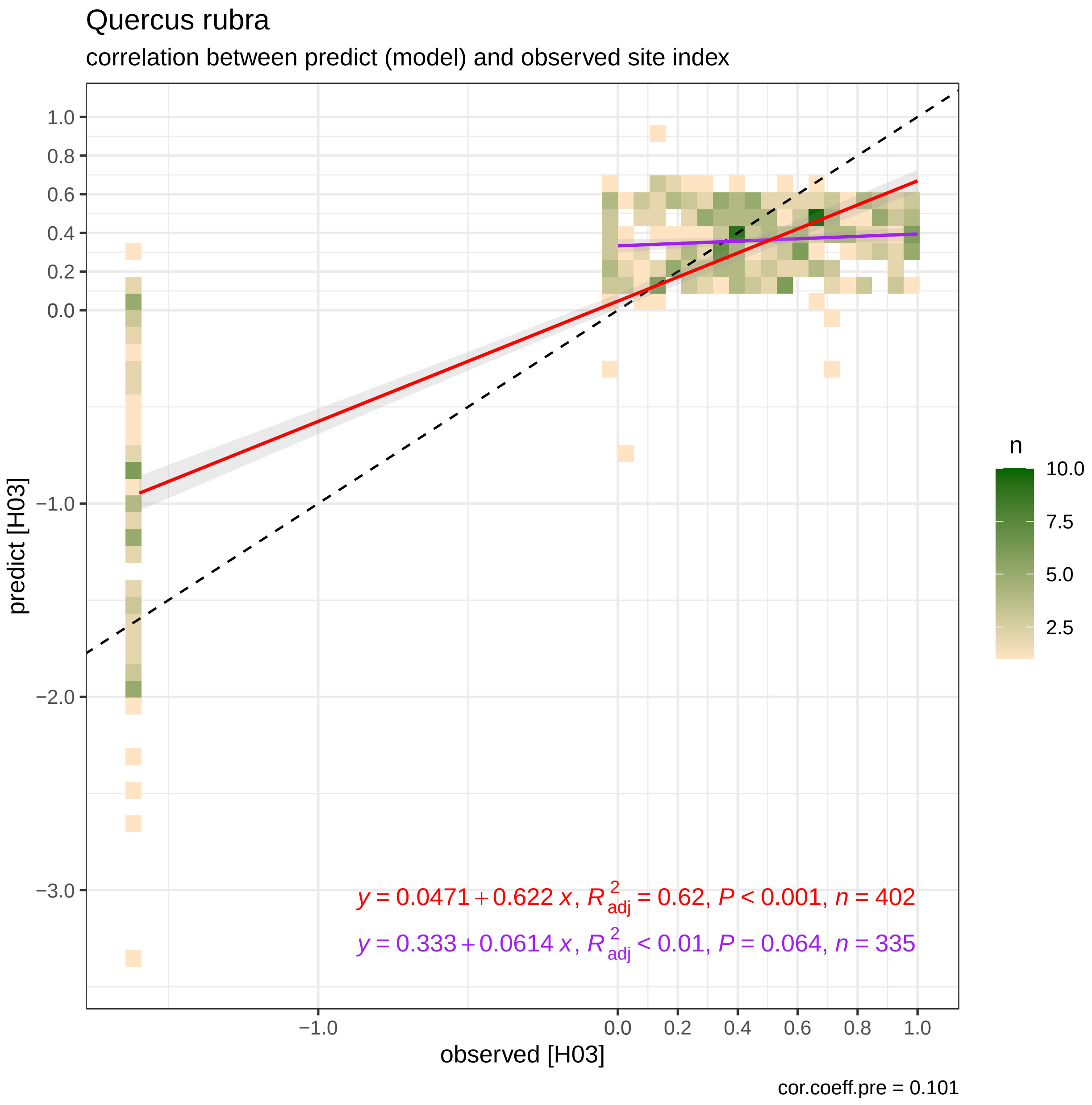

Predictions and forecasts

Predict

European predict for the reference period (1981 to 2010). Dark green symbolizes a high site index (tree height in meters at age 100), orange a lower site index and red no growth. Magenta-coloured dots represent inventory points with growth information, light blue dots are absences (= ‘growth absences’). Results were aggregated on 25 km x 25 km scale.

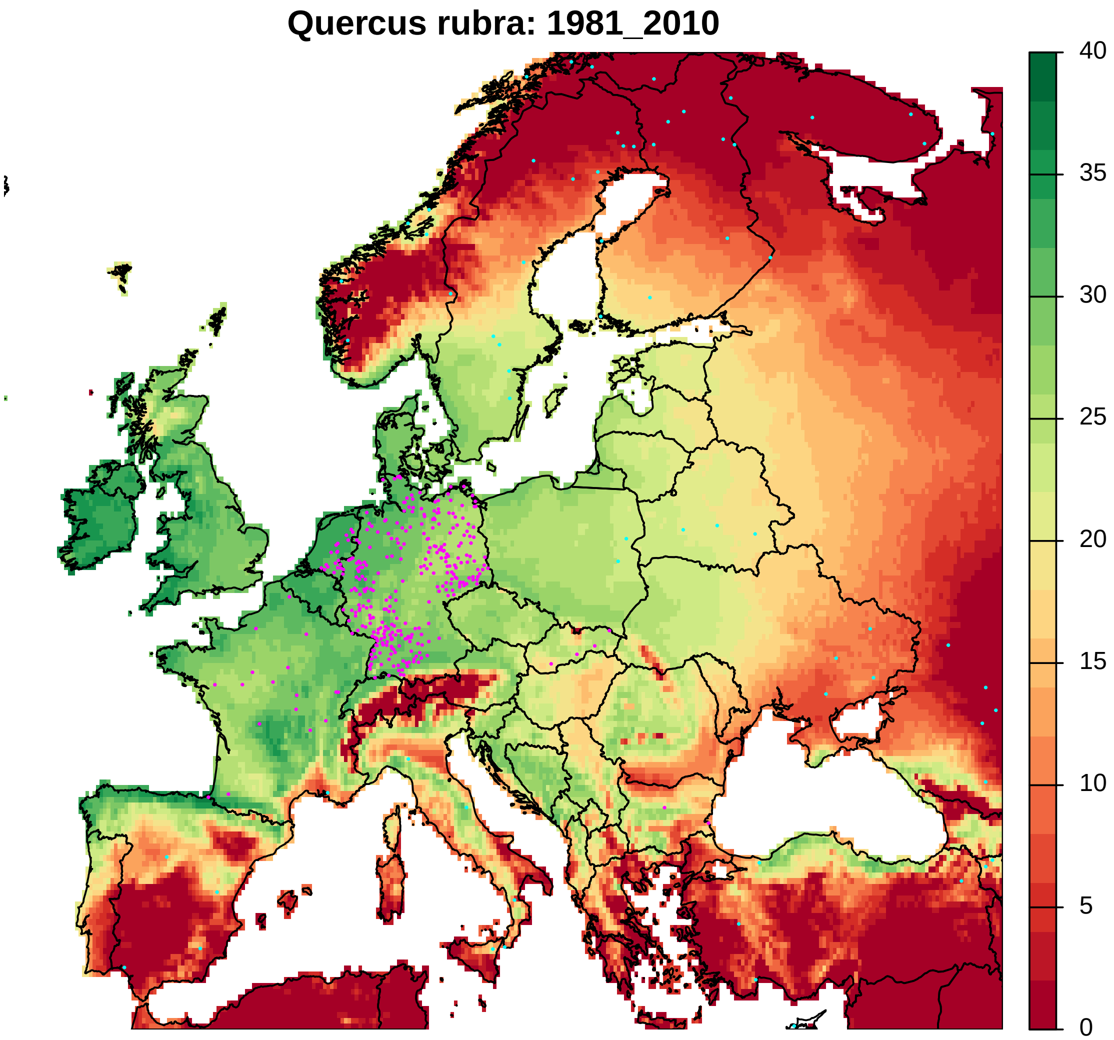

Forecast

Prediction for the reference period (1981 to 2010), as well as forecasts to 2071 to 2100 under szenario RCP4.5 and RCP8.5. Dark green symbolizes a high site index (tree height in m at age 100), orange a lower site index and red no growth. Results were aggregated on 25 km x 25 km scale.

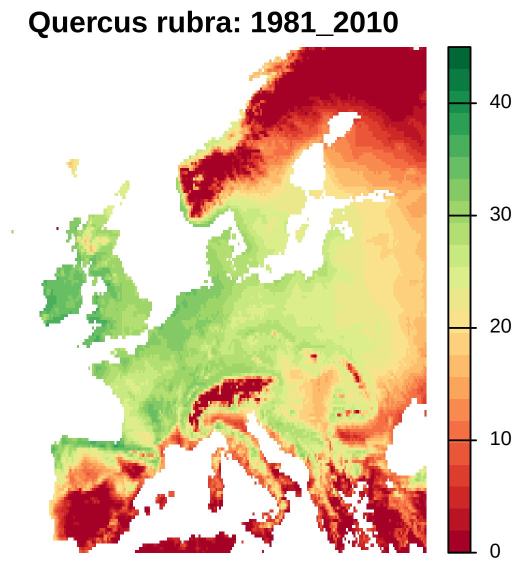

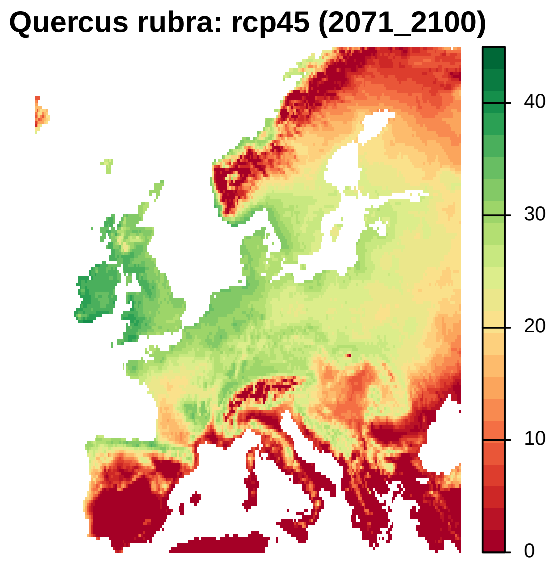

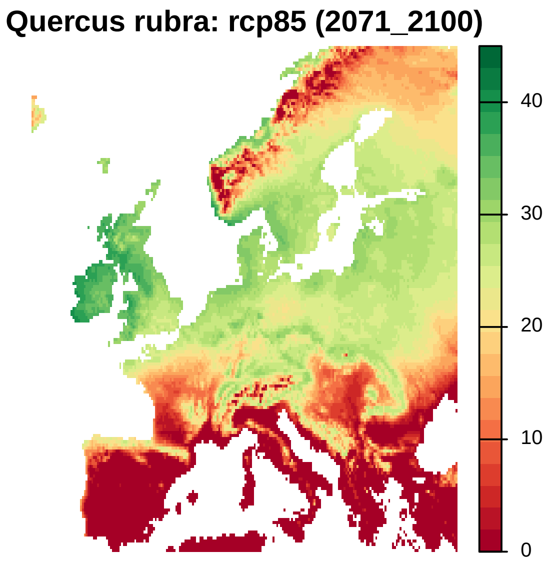

Robinia pseudoacacia

Site index curves

Site index curves of Robinia pseudoacacia created with non-linear quantile regressions based on the algorithm of Koenker and Park (1992). The site index (SI) was created by setting all points on the 95 percent quantile (upper line) and above to one (SI = 1) and all on the 5 percent quantile (lower line) and below to zero (SI = 0). The points between the quantile boundaries were assigned a site index between zero and one according to the ratio of their position between the quantile boundaries. We set selected absences (see chapter 2.1.3) on Height = 0 m (at age 100), which means, depending on the site index curves, for each tree species a SI near -1 (red line).

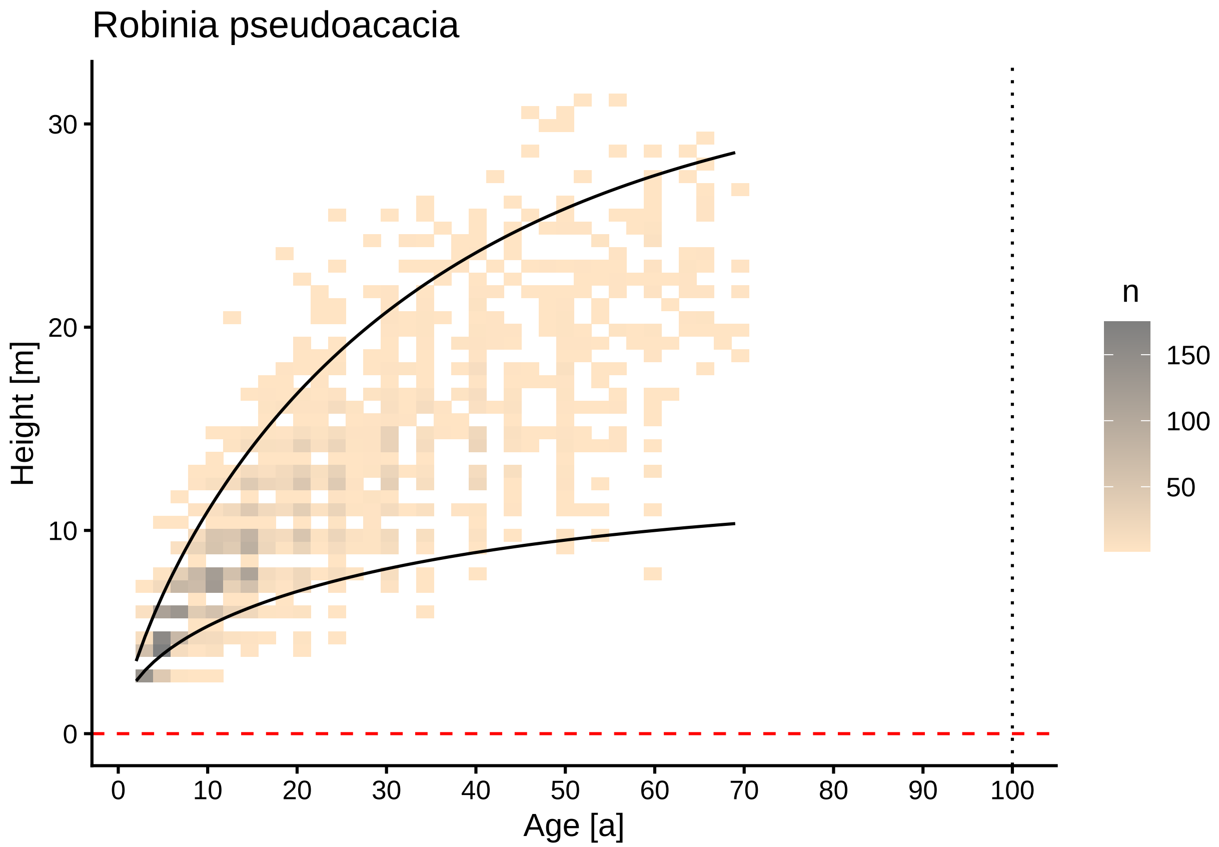

Model statistics and evaluation

Summary

Predictor acronyms: Bio.1 = Mean annual temperature [°C], Bio.12 = Annual precipitation sum [mm/m2], sp_p = Sum of precipitation [mm/m2] within months 3 to 5, su_p = Sum of precipitation [mm/m2] within months 6 to 8, wi_p = Sum of precipitation [mm/m2] within months 12,1,2, sp_t = Mean temperature [°C] within months 3 to 5, su_t = Mean temperature [°C] within months 6 to 8, wi_t = Mean temperature [°C] within months 12,1,2.

```
##
## Family: gaussian
## Link function: identity
##
## Formula:
## H03 ~ s(reference_19812010_wi_t, k = 3) + s(reference_19812010_su_p,
##     k = 3) + s(reference_19812010_wi_p, k = 3)
##
## Parametric coefficients:
##             Estimate Std. Error t value Pr(>|t|)
## (Intercept)   0.4744     0.0131    36.2   <2e-16 ***
## ---
## Signif. codes:  0 '***' 0.001 '**' 0.01 '*' 0.05 '.' 0.1 ' ' 1
##
## Approximate significance of smooth terms:
##                              edf Ref.df      F p-value
## s(reference_19812010_wi_t) 1.967  1.998 292.04  <2e-16 ***
## s(reference_19812010_su_p) 1.964  1.998  79.04  <2e-16 ***
## s(reference_19812010_wi_p) 1.945  1.996  50.25  <2e-16 ***
## ---
## Signif. codes:  0 '***' 0.001 '**' 0.01 '*' 0.05 '.' 0.1 ' ' 1
##
## R-sq.(adj) =  0.511   Deviance explained = 51.5%
## -REML = 342.74  Scale est. = 0.13462   n = 784
```

Variance inflation factor (VIF)

Predictor acronyms: Bio.1 = Mean annual temperature [°C], Bio.12 = Annual precipitation sum [mm/m2], sp_p = Sum of precipitation [mm/m2] within months 3 to 5, su_p = Sum of precipitation [mm/m2] within months 6 to 8, wi_p = Sum of precipitation [mm/m2] within months 12,1,2, sp_t = Mean temperature [°C] within months 3 to 5, su_t = Mean temperature [°C] within months 6 to 8, wi_t = Mean temperature [°C] within months 12,1,2.

```
##                   Variables      VIF
## 1 reference_19812010_wi_t 1.588890
## 2 reference_19812010_su_p 2.124361
## 3 reference_19812010_wi_p 1.570273
```

Correlation matrix

Correlation matrix between the predictor variables and the target variable in the model. Correlation coefficient according to PEARSON. Predictor acronyms: Bio.1 = Mean annual temperature [°C], Bio.12 = Annual precipitation sum [mm/m2], sp_p = Sum of precipitation [mm/m2] within months 3 to 5, su_p = Sum of precipitation [mm/m2] within months 6 to 8, wi_p = Sum of precipitation [mm/m2] within months 12,1,2, sp_t = Mean temperature [°C] within months 3 to 5, su_t = Mean temperature [°C] within months 6 to 8, wi_t = Mean temperature [°C] within months 12,1,2.

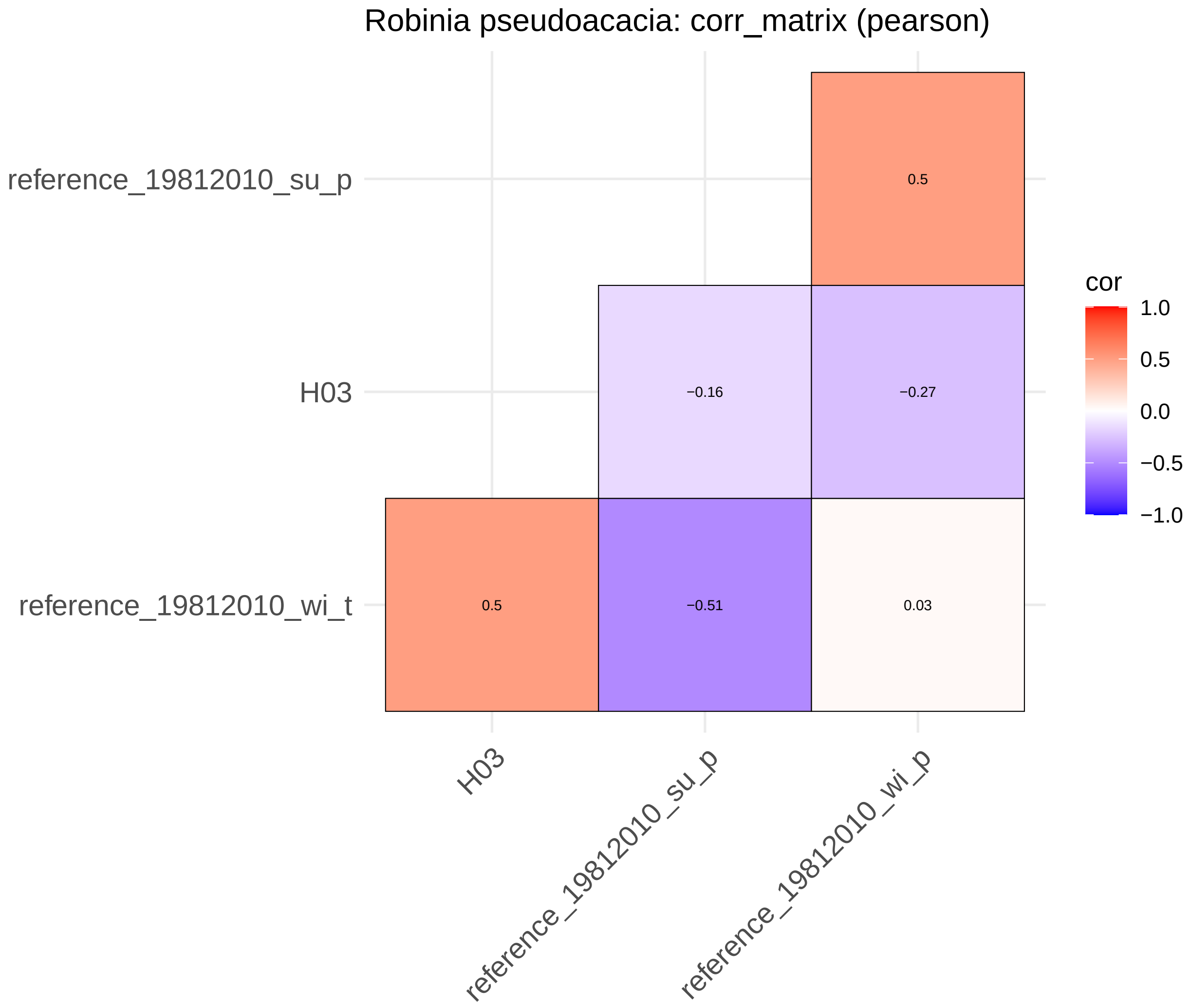

Response curves

Response curves (also known as effect curves) show how each predictor variable affects the target variable (H03 = european Site index, SIrel). H03 values below zero represent 'Growth absences'. Predictor acronyms: Bio.1 = Mean annual temperature [°C], Bio.12 = Annual precipitation sum [mm/m2], sp_p = Sum of precipitation [mm/m2] within months 3 to 5, su_p = Sum of precipitation [mm/m2] within months 6 to 8, wi_p = Sum of precipitation [mm/m2] within months 12,1,2, sp_t = Mean temperature [°C] within months 3 to 5, su_t = Mean temperature [°C] within months 6 to 8, wi_t = Mean temperature [°C] within months 12,1,2.

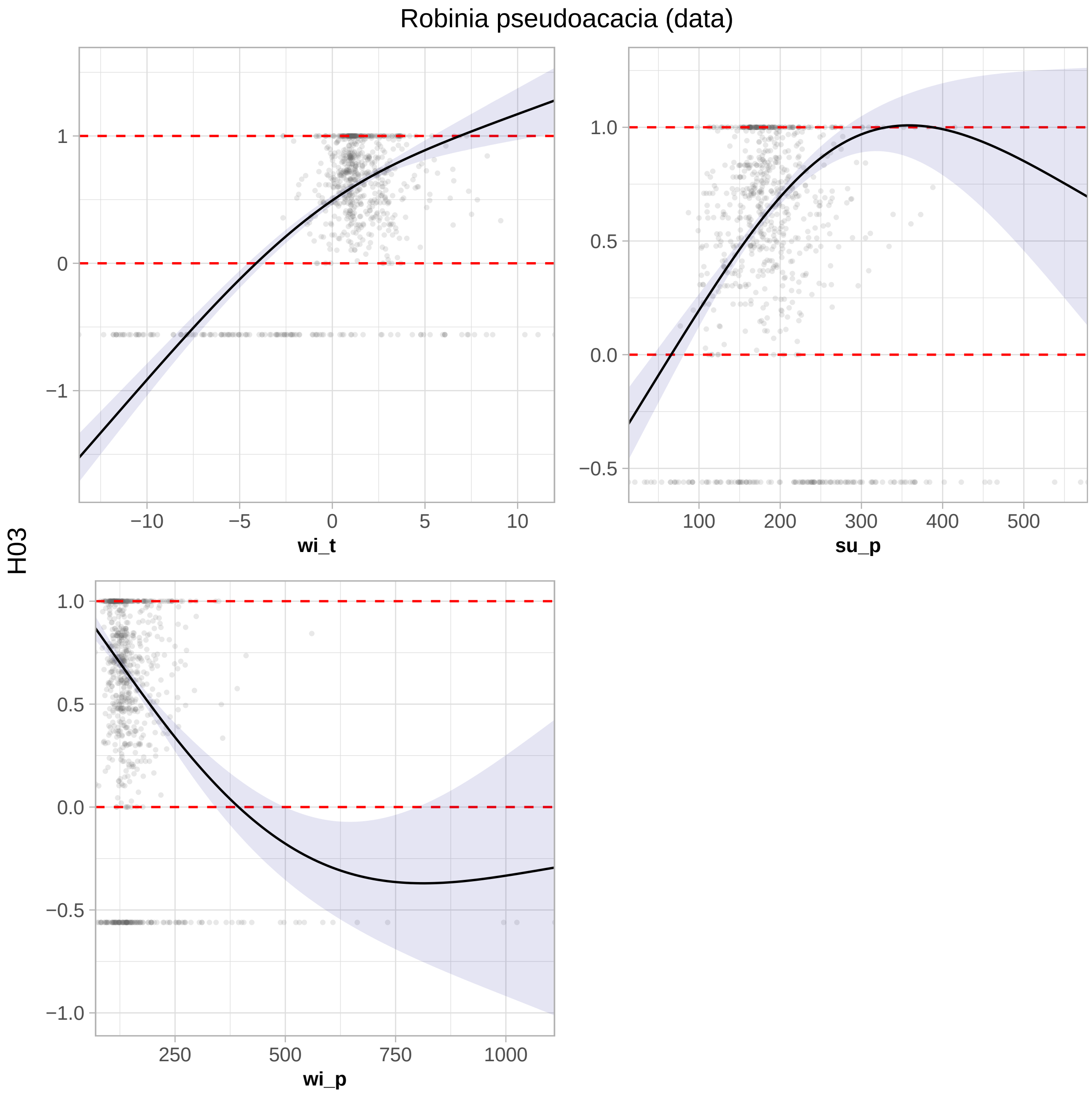

Response maps

Response maps (also referred as partial effect maps). Each map visualizes how a predictor affect the target variable (top height [m] at Age 100). Technically their work like response curves in a geographical area, that is setting all predictor variables except the one shown in the figure on their mean, and mapping the prediction. Predictor acronyms: Bio.1 = Mean annual temperature [°C], Bio.12 = Annual precipitation sum [mm/m2], sp_p = Sum of precipitation [mm/m2] within months 3 to 5, su_p = Sum of precipitation [mm/m2] within months 6 to 8, wi_p = Sum of precipitation [mm/m2] within months 12,1,2, sp_t = Mean temperature [°C] within months 3 to 5, su_t = Mean temperature [°C] within months 6 to 8, wi_t = Mean temperature [°C] within months 12,1,2.

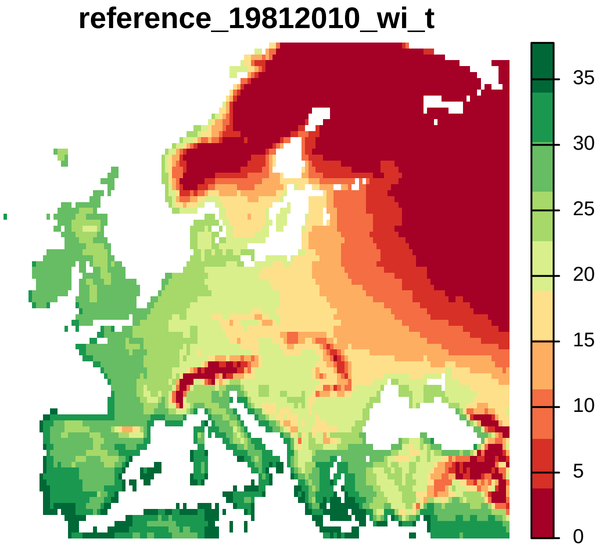

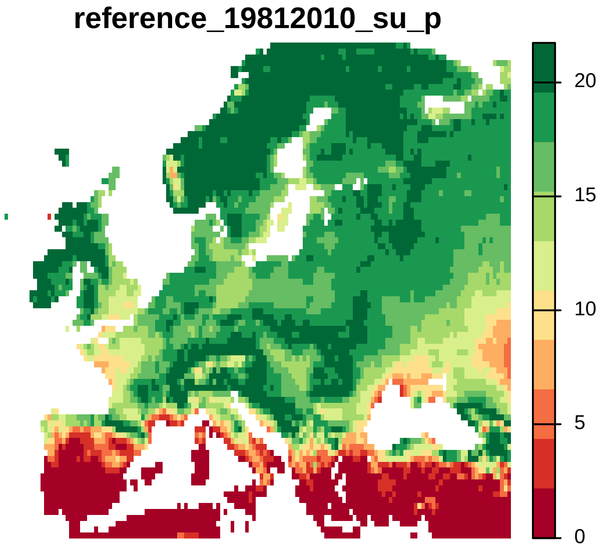

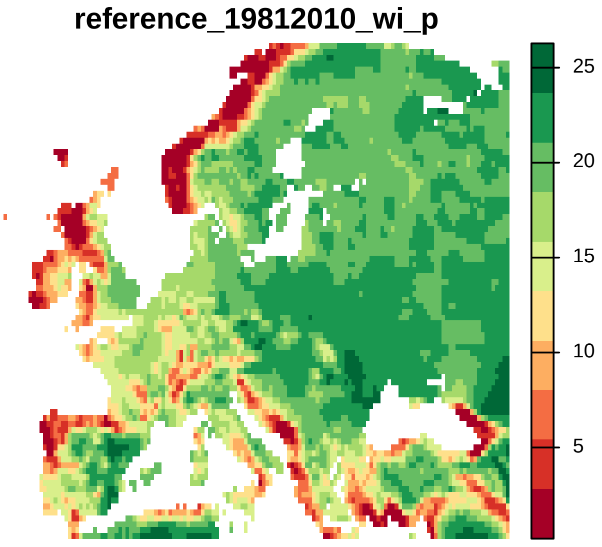

Residual distribution

The multi-panel plot includes a histogram of the residuals (top left), residuals over fitted values (top right), a histogram of observed and predicted values (bottom left) and boxplot diagram of observed and predicted values (bottom right). Observed values are shown in light green, while predicted ones are depicted in light red.

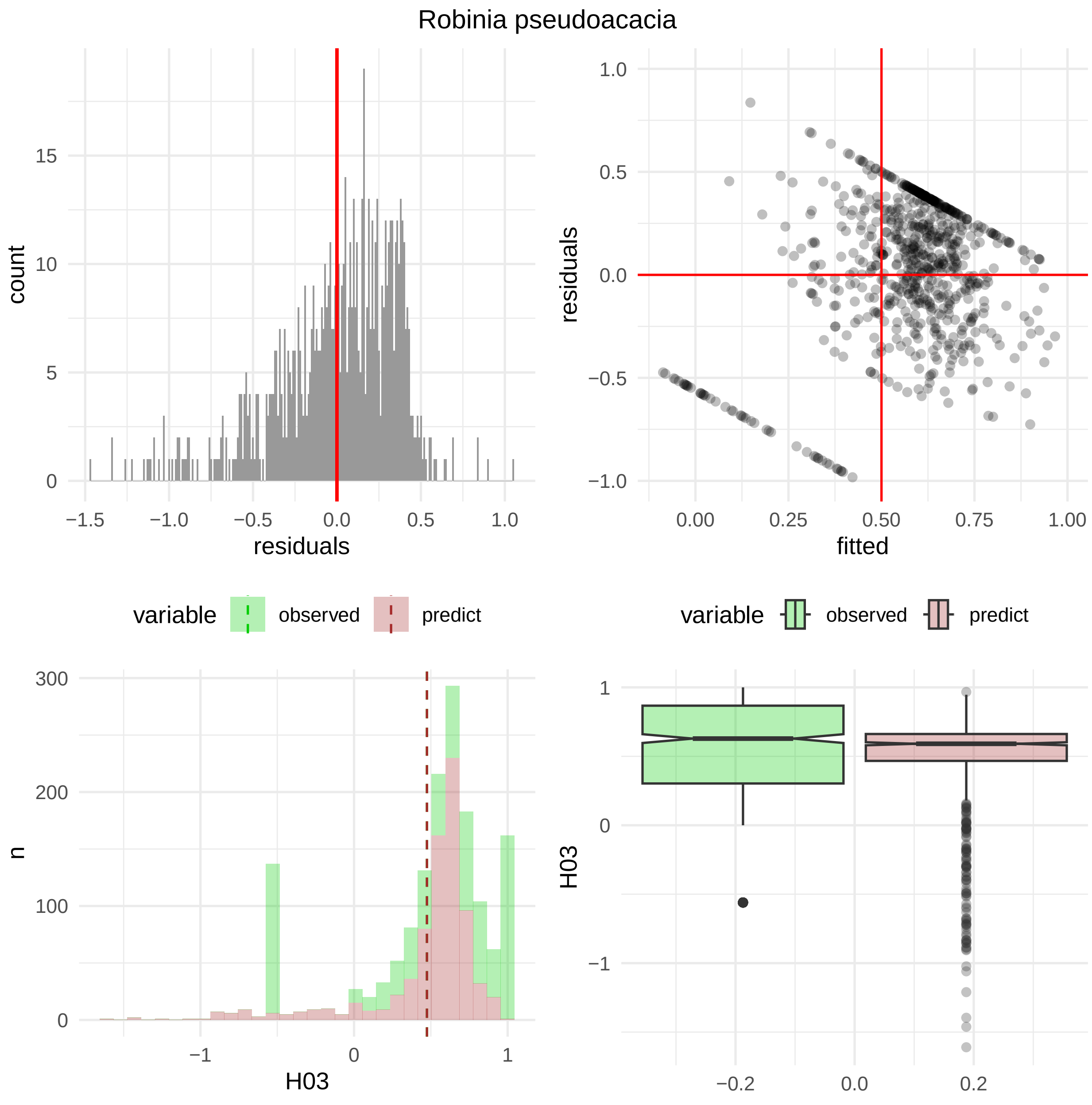

Correlation between predict and observed site index

Relationship between predicted and observed site index (density cloud), as well as linear regressions of presences and absences (= 'growth absences') (red line) and presences only (magenta line). The formulas, significance, R2 and number of observations are displayed below for both regressions. Ideally, both the point cloud and the regression lines lie close to the dashed line. For presences only we additionally calculated the correlation coefficient according to PEARSON (cor.pre) in the bottom right corner.

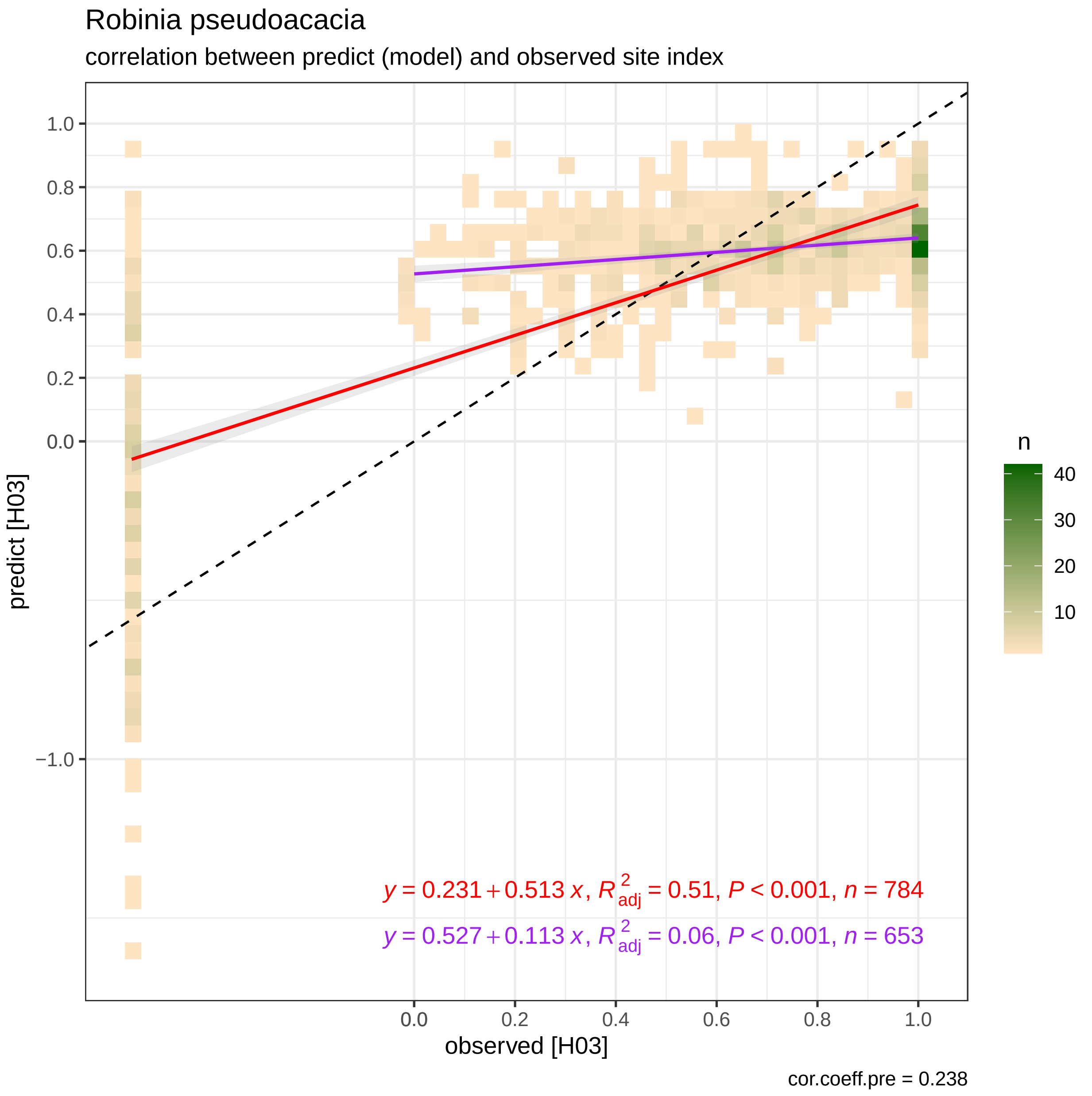

Predictions and forecasts

Predict

European predict for the reference period (1981 to 2010). Dark green symbolizes a high site index (tree height in meters at age 100), orange a lower site index and red no growth. Magenta-coloured dots represent inventory points with growth information, light blue dots are absences (= 'growth absences'). Results were aggregated on 25 km x 25 km scale.

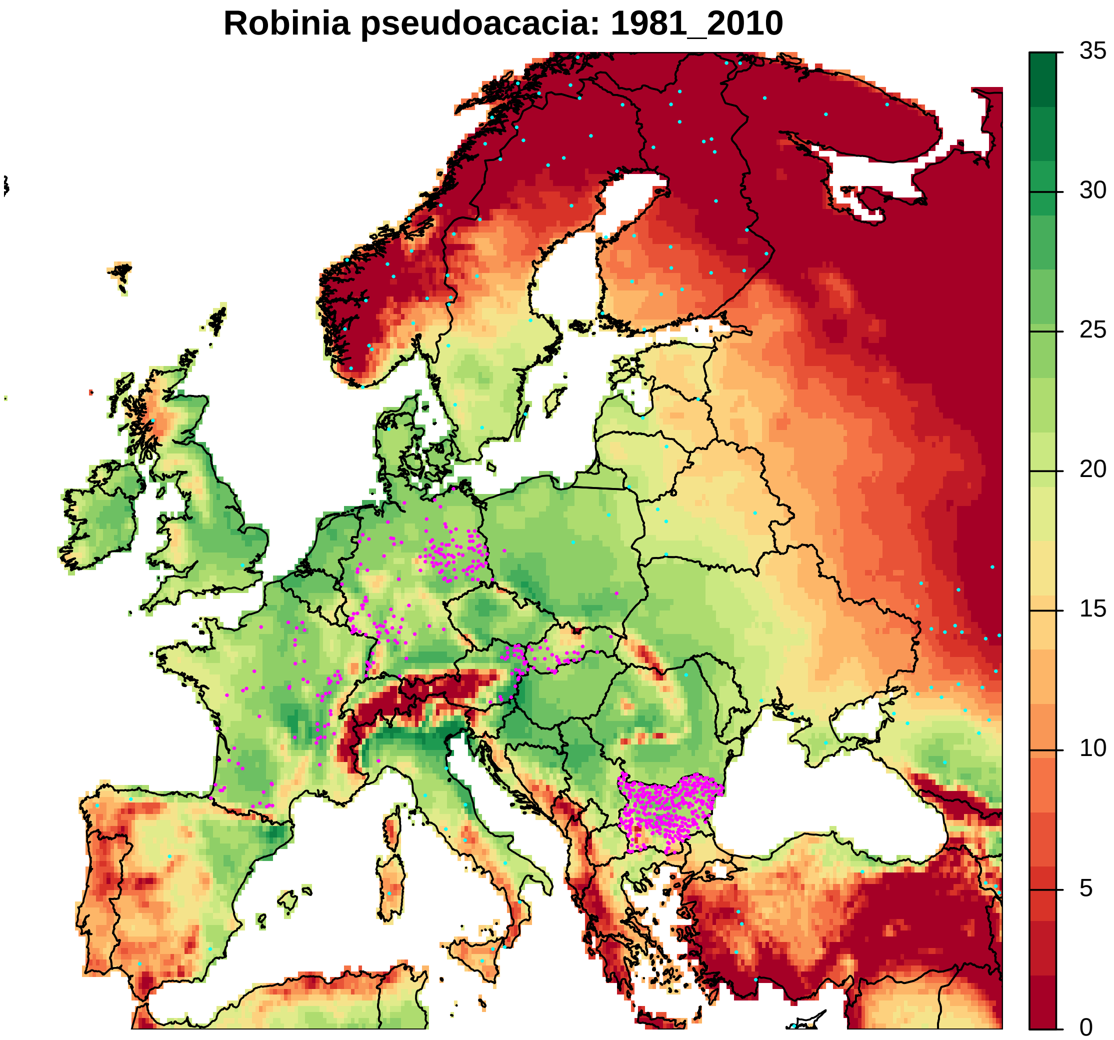

Forecast

Prediction for the reference period (1981 to 2010), as well as forecasts to 2071 to 2100 under szenario RCP4.5 and RCP8.5. Dark green symbolizes a high site index (tree height in m at age 100), orange a lower site index and red no growth. Results were aggregated on 25 km x 25 km scale.

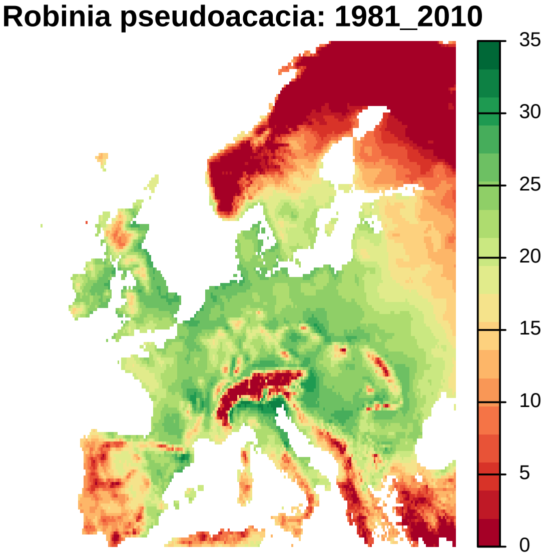

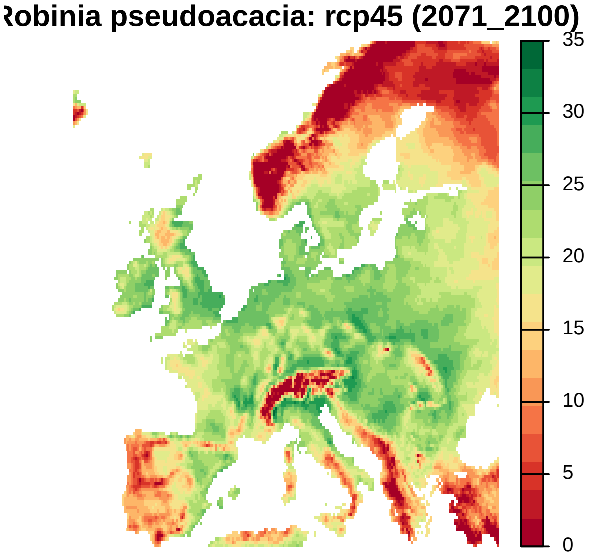

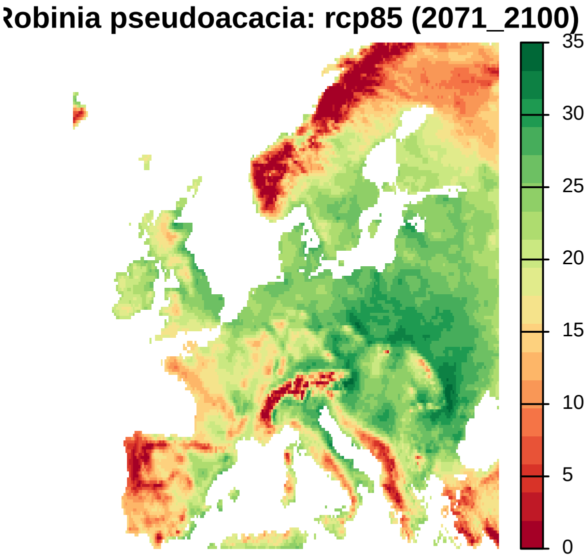

Sorbus aucuparia

Site index curves

Site index curves of Sorbus aucuparia created with non-linear quantile regressions based on the algorithm of Koenker and Park (1992). The site index (SI) was created by setting all points on the 95 percent quantile (upper line) and above to one (SI = 1) and all on the 5 percent quantile (lower line) and below to zero (SI = 0). The points between the quantile boundaries were assigned a site index between zero and one according to the ratio of their position between the quantile boundaries. We set selected absences (see chapter 2.1.3) on Height = 0 m (at age 100), which means, depending on the site index curves, for each tree species a SI near -1 (red line).

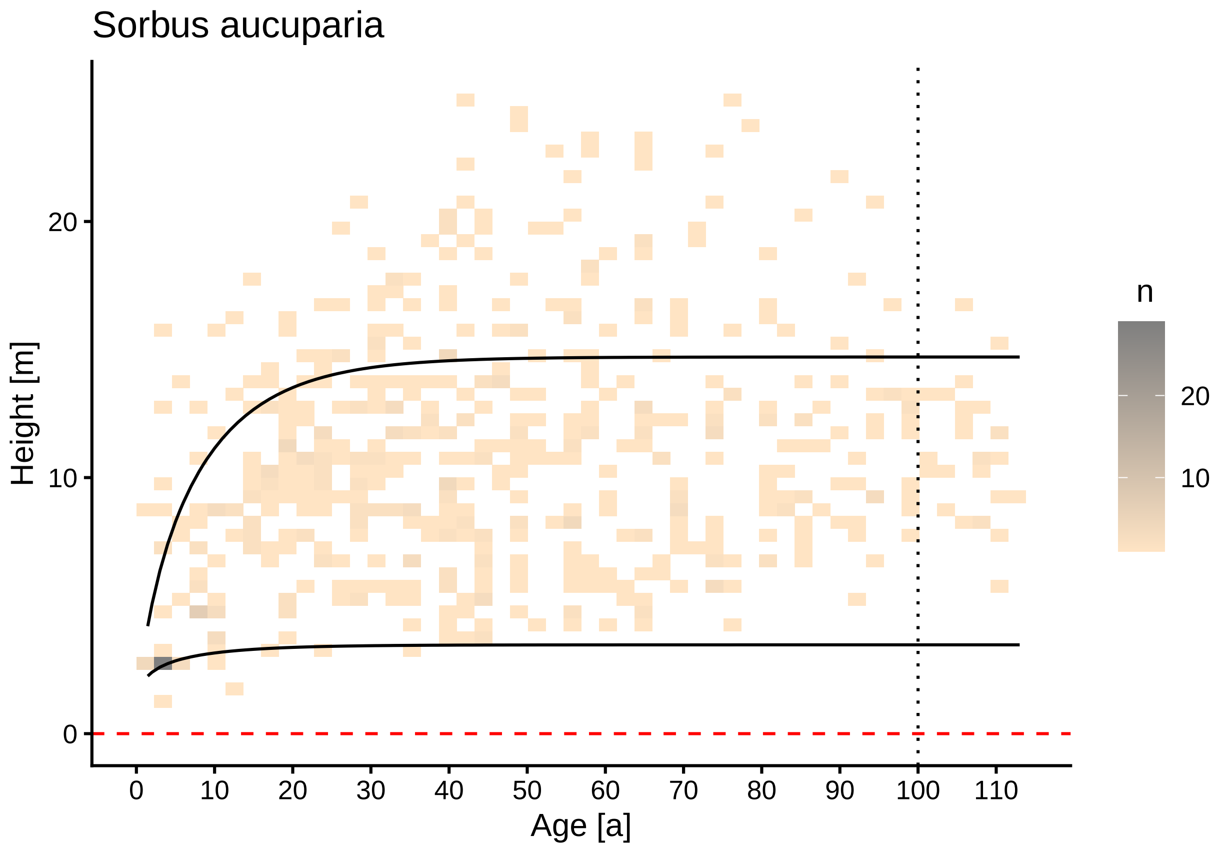

Model statistics and evaluation

Summary

Predictor acronyms: Bio.1 = Mean annual temperature [°C], Bio.12 = Annual precipitation sum [mm/m2], sp_p = Sum of precipitation [mm/m2] within months 3 to 5, su_p = Sum of precipitation [mm/m2] within months 6 to 8, wi_p = Sum of precipitation [mm/m2] within months 12,1,2, sp_t = Mean temperature [°C] within months 3 to 5, su_t = Mean temperature [°C] within months 6 to 8, wi_t = Mean temperature [°C] within months 12,1,2.

```
##
## Family: gaussian
## Link function: identity
##
## Formula:
## H03 ~ s(reference_19812010_su_t, k = 3) + s(reference_19812010_wi_t,
##     k = 3) + s(reference_19812010_su_p, k = 3)
##
## Parametric coefficients:
##             Estimate Std. Error t value Pr(>|t|)
## (Intercept)  0.47709    0.01269    37.6   <2e-16 ***
## ---
## Signif. codes:  0 '***' 0.001 '**' 0.01 '*' 0.05 '.' 0.1 ' ' 1
##
## Approximate significance of smooth terms:
##                              edf Ref.df      F  p-value
## s(reference_19812010_su_t) 1.995  2.000 124.39  < 2e-16 ***
## s(reference_19812010_wi_t) 1.000  1.000  20.18 8.68e-06 ***
## s(reference_19812010_su_p) 1.965  1.999  14.51 1.55e-06 ***
## ---
## Signif. codes:  0 '***' 0.001 '**' 0.01 '*' 0.05 '.' 0.1 ' ' 1
##
## R-sq.(adj) =  0.527   Deviance explained = 53.1%
## -REML = 171.09  Scale est. = 0.09772   n = 607
```

Variance inflation factor (VIF)

Predictor acronyms: Bio.1 = Mean annual temperature [°C], Bio.12 = Annual precipitation sum [mm/m2], sp_p = Sum of precipitation [mm/m2] within months 3 to 5, su_p = Sum of precipitation [mm/m2] within months 6 to 8, wi_p = Sum of precipitation [mm/m2] within months 12,1,2, sp_t = Mean temperature [°C] within months 3 to 5, su_t = Mean temperature [°C] within months 6 to 8, wi_t = Mean temperature [°C] within months 12,1,2.

```
##                  Variables      VIF
## 1 reference_19812010_su_t 2.721473
## 2 reference_19812010_wi_t 1.527563
## 3 reference_19812010_su_p 2.033671
```

Correlation matrix

Correlation matrix between the predictor variables and the target variable in the model. Correlation coefficient according to PEARSON. Predictor acronyms: Bio.1 = Mean annual temperature [°C], Bio.12 = Annual precipitation sum [mm/m2], sp_p = Sum of precipitation [mm/m2] within months 3 to 5, su_p = Sum of precipitation [mm/m2] within months 6 to 8, wi_p = Sum of precipitation [mm/m2] within months 12,1,2, sp_t = Mean temperature [°C] within months 3 to 5, su_t = Mean temperature [°C] within months 6 to 8, wi_t = Mean temperature [°C] within months 12,1,2.

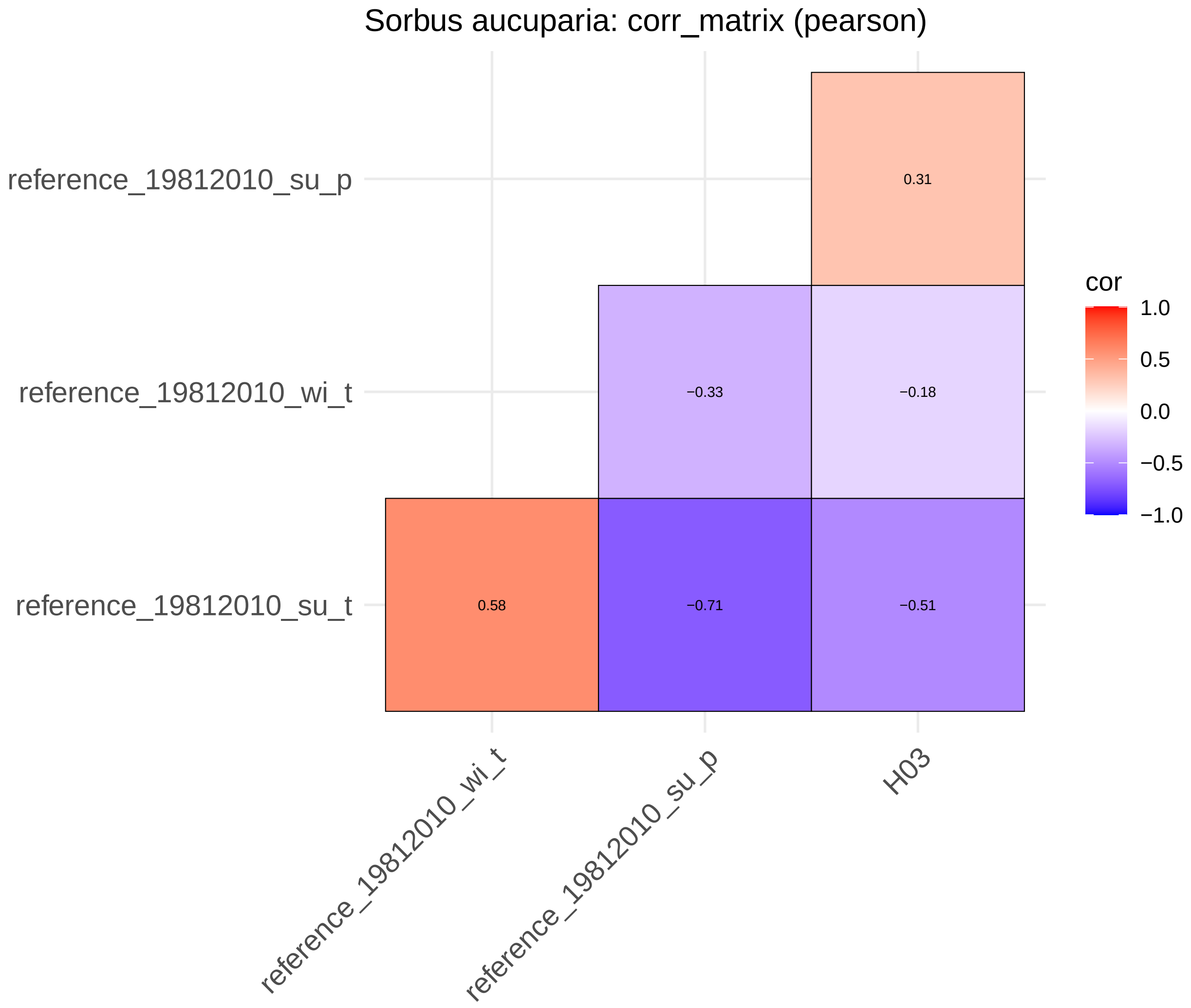

Response curves

Response curves (also known as effect curves) show how each predictor variable affects the target variable (H03 = european Site index, SIrel). H03 values below zero represent 'Growth absences'. Predictor acronyms: Bio.1 = Mean annual temperature [°C], Bio.12 = Annual precipitation sum [mm/m2], sp_p = Sum of precipitation [mm/m2] within months 3 to 5, su_p = Sum of precipitation [mm/m2] within months 6 to 8, wi_p = Sum of precipitation [mm/m2] within months 12,1,2, sp_t = Mean temperature [°C] within months 3 to 5, su_t = Mean temperature [°C] within months 6 to 8, wi_t = Mean temperature [°C] within months 12,1,2.

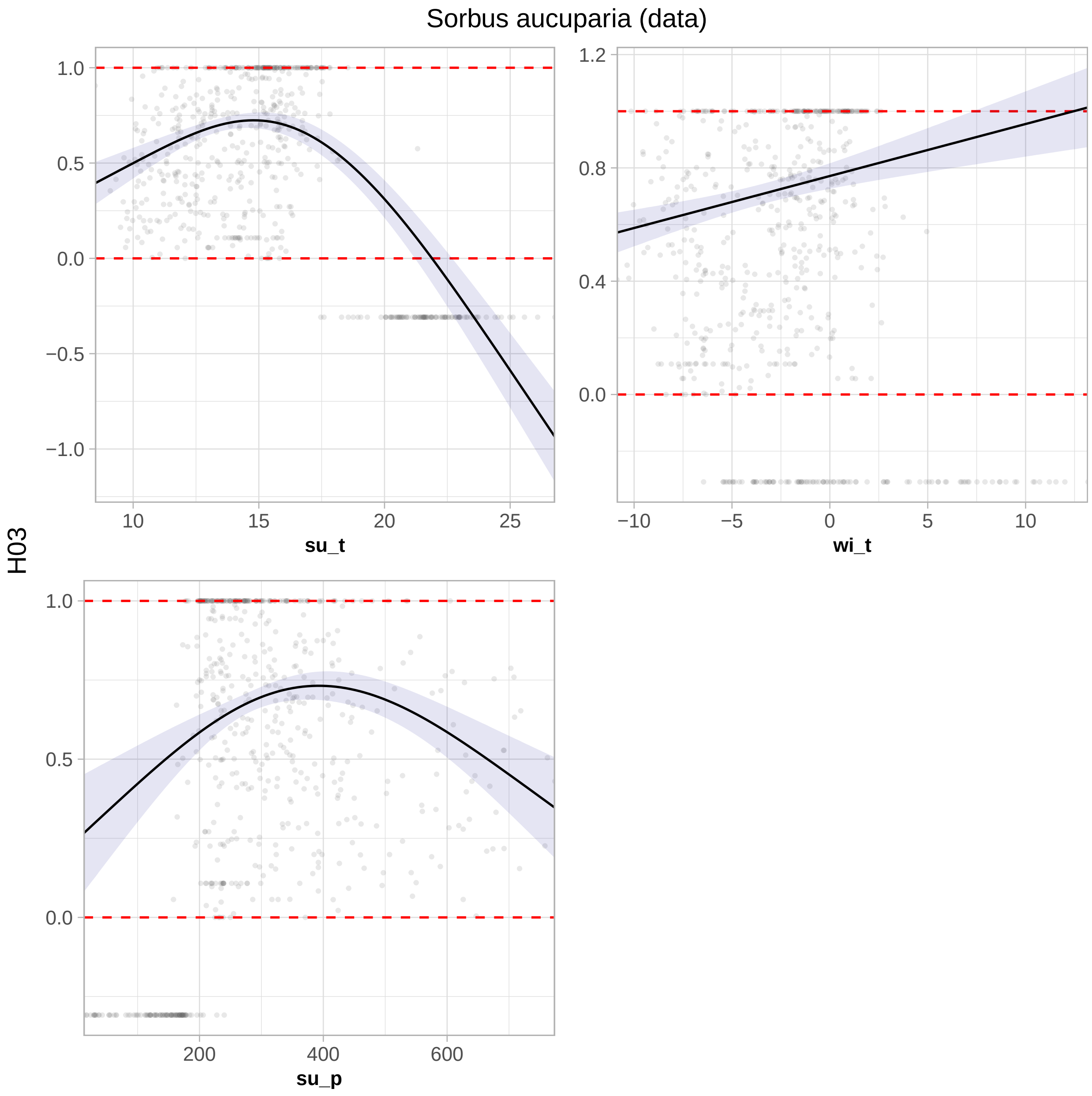

Response maps

Response maps (also referred as partial effect maps). Each map visualizes how a predictor affect the target variable (top height [m] at Age 100). Technically their work like response curves in a geographical area, that is setting all predictor variables except the one shown in the figure on their mean, and mapping the prediction. Predictor acronyms: Bio.1 = Mean annual temperature [°C], Bio.12 = Annual precipitation sum [mm/m2], sp_p = Sum of precipitation [mm/m2] within months 3 to 5, su_p = Sum of precipitation [mm/m2] within months 6 to 8, wi_p = Sum of precipitation [mm/m2] within months 12,1,2, sp_t = Mean temperature [°C] within months 3 to 5, su_t = Mean temperature [°C] within months 6 to 8, wi_t = Mean temperature [°C] within months 12,1,2.

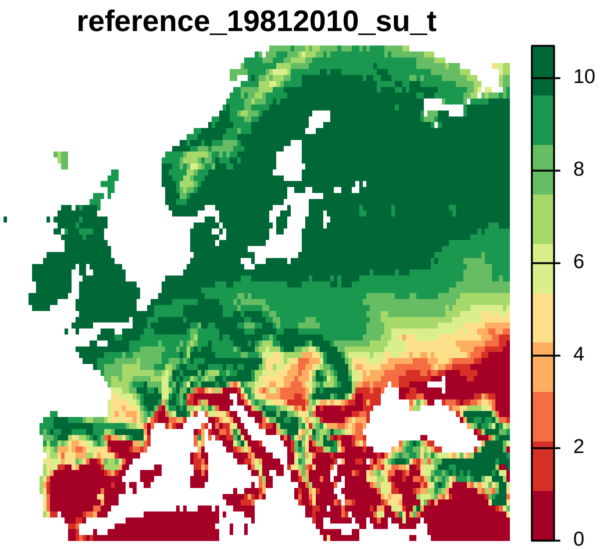

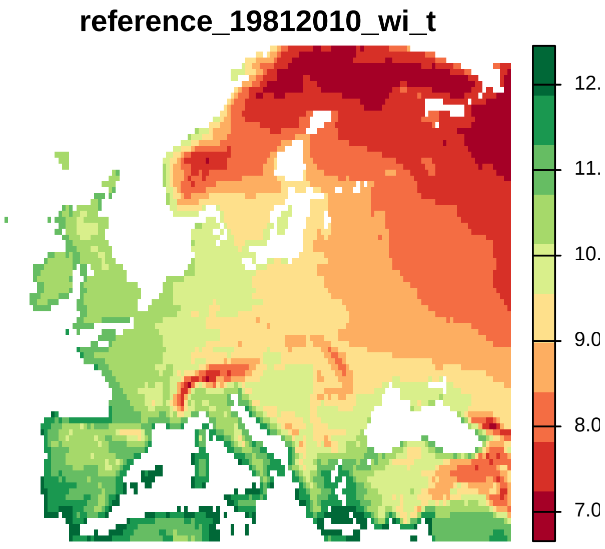

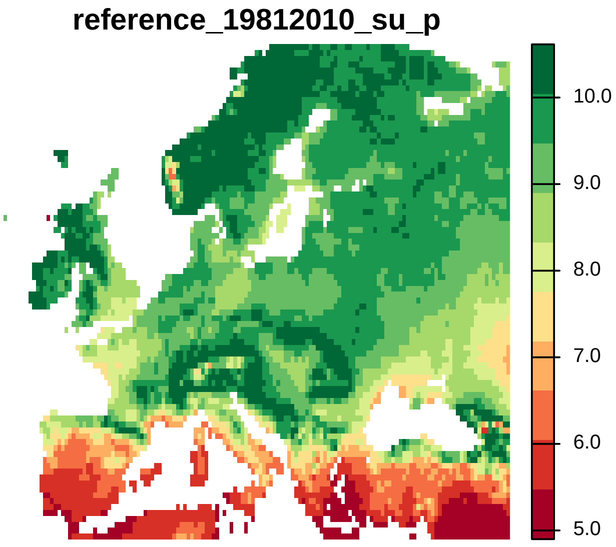

Residual distribution

The multi-panel plot includes a histogram of the residuals (top left), residuals over fitted values (top right), a histogram of observed and predicted values (bottom left) and boxplot diagram of observed and predicted values (bottom right). Observed values are shown in light green, while predicted ones are depicted in light red.

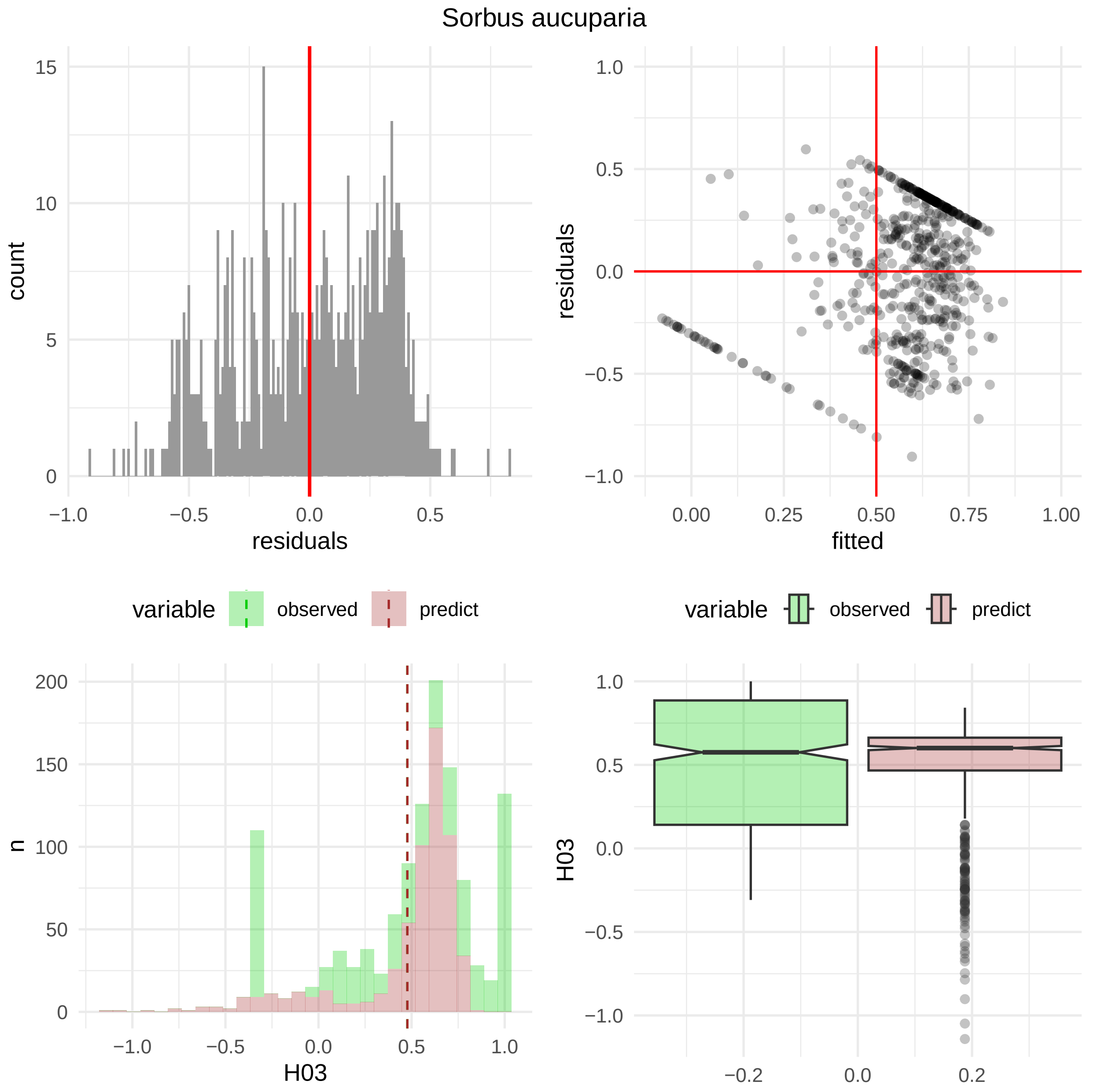

Correlation between predict and observed site index

Relationship between predicted and observed site index (density cloud), as well as linear regressions of presences and absences (= 'growth absences') (red line) and presences only (magenta line). The formulas, significance, R2 and number of observations are displayed below for both regressions. Ideally, both the point cloud and the regression lines lie close to the dashed line. For presences only we additionally calculated the correlation coefficient according to PEARSON (cor.pre) in the bottom right corner.

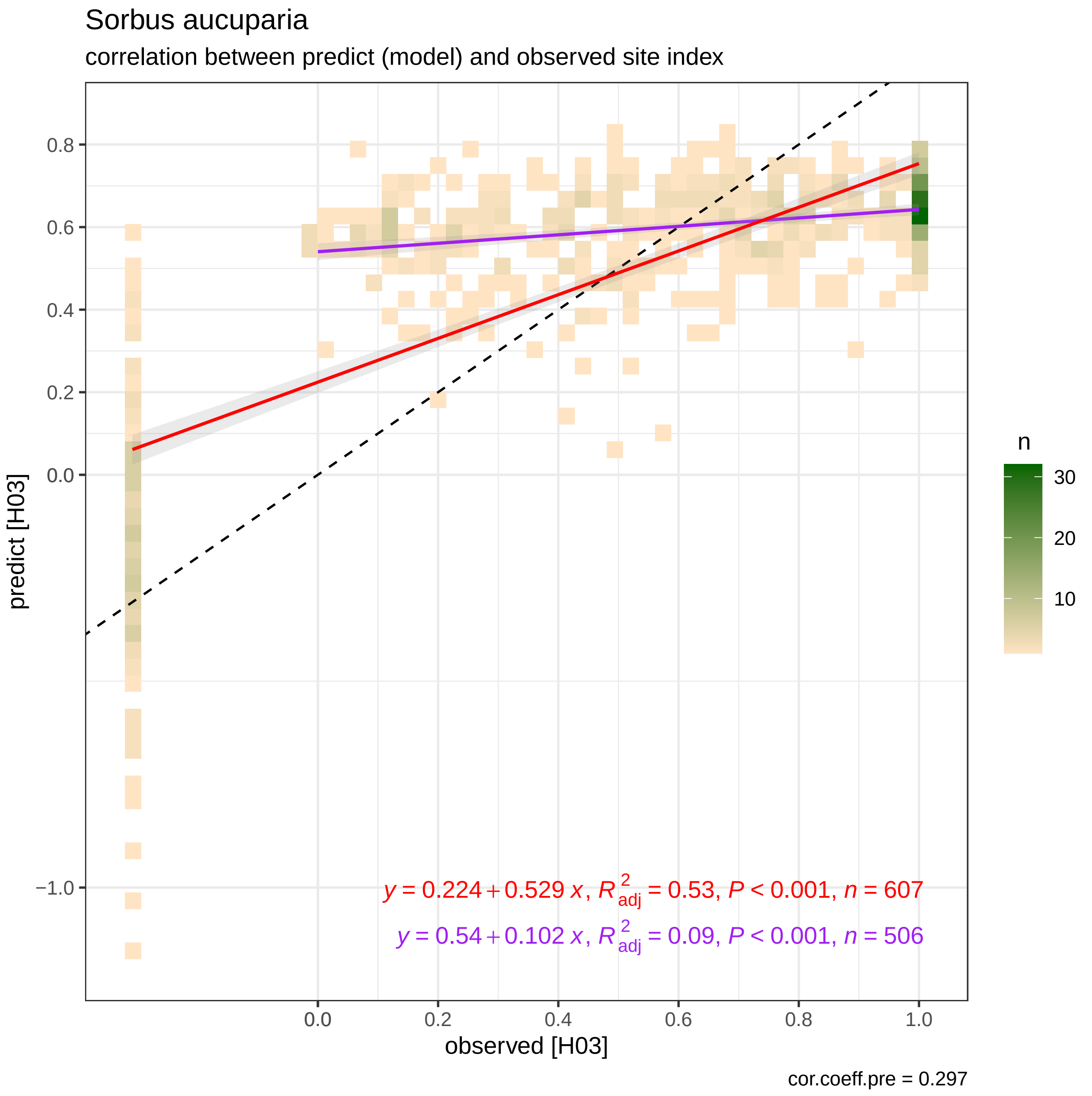

Predictions and forecasts

Predict

European predict for the reference period (1981 to 2010). Dark green symbolizes a high site index (tree height in meters at age 100), orange a lower site index and red no growth. Magenta-coloured dots represent inventory points with growth information, light blue dots are absences (= 'growth absences'). Results were aggregated on 25 km x 25 km scale.

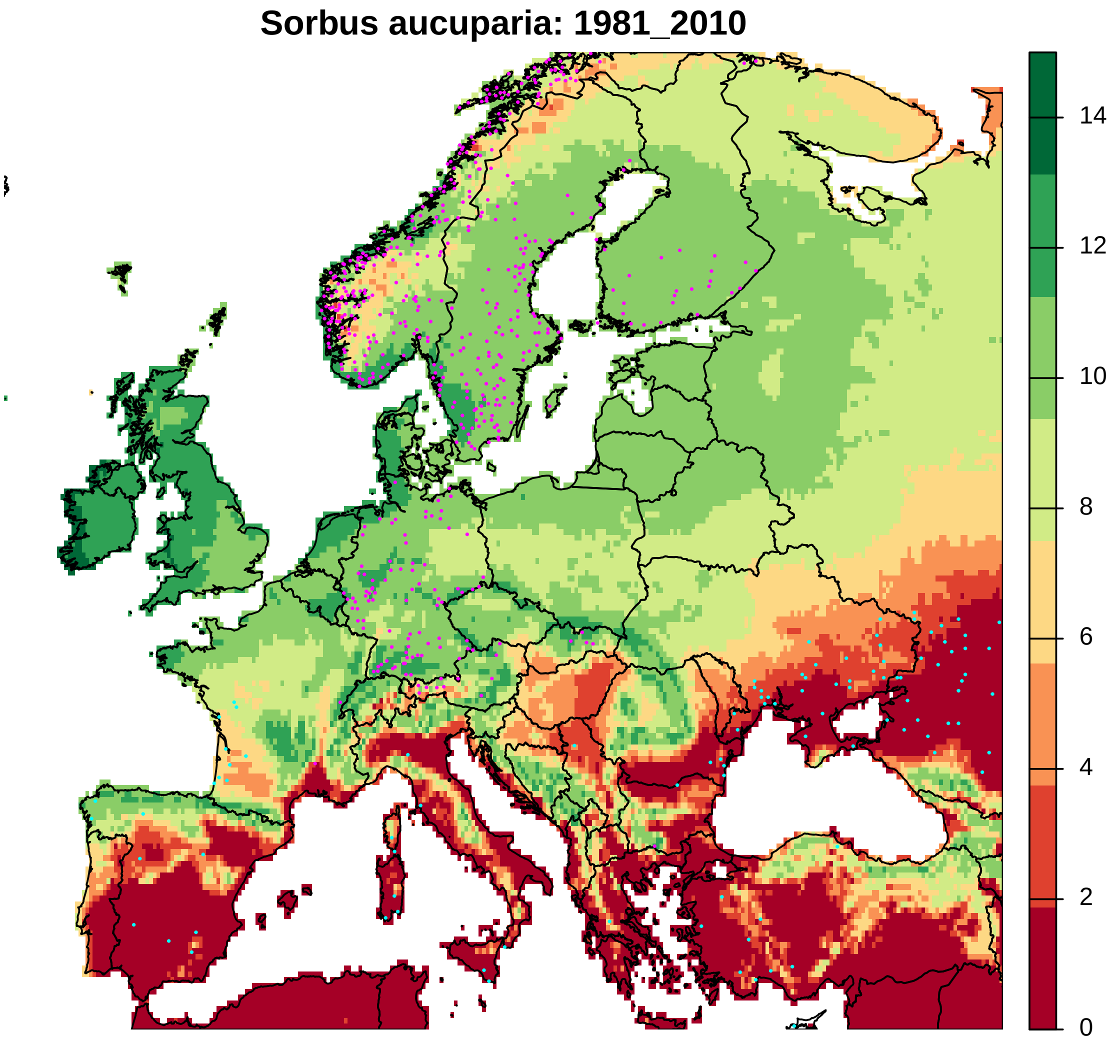

Forecast

Prediction for the reference period (1981 to 2010), as well as forecasts to 2071 to 2100 under szenario RCP4.5 and RCP8.5. Dark green symbolizes a high site index (tree height in m at age 100), orange a lower site index and red no growth. Results were aggregated on 25 km x 25 km scale.

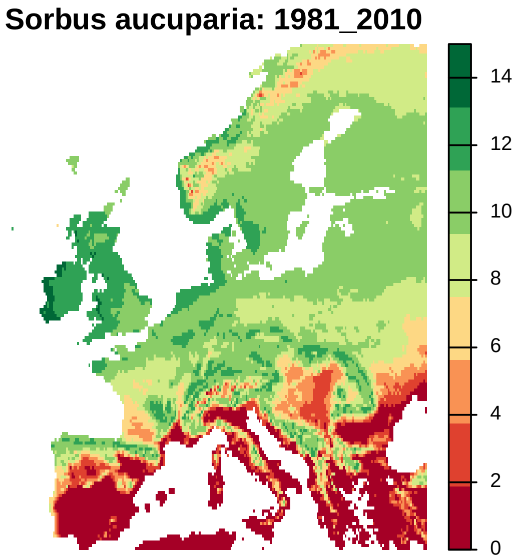

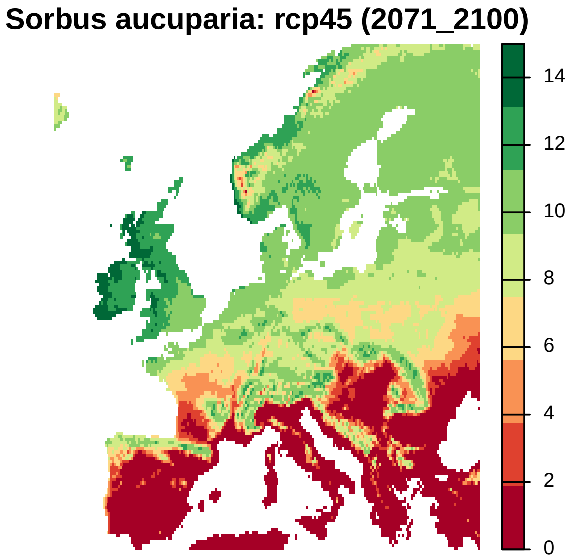

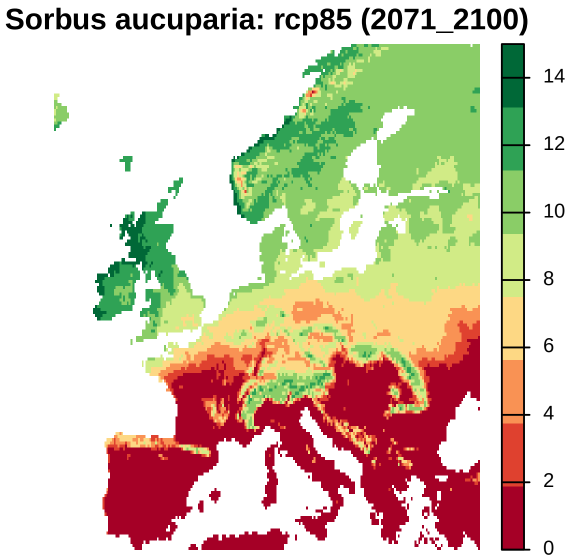

Tilia cordata

Site index curves

Site index curves of Tilia cordata created with non-linear quantile regressions based on the algorithm of Koenker and Park (1992). The site index (SI) was created by setting all points on the 95 percent quantile (upper line) and above to one (SI = 1) and all on the 5 percent quantile (lower line) and below to zero (SI = 0). The points between the quantile boundaries were assigned a site index between zero and one according to the ratio of their position between the quantile boundaries. We set selected absences (see chapter 2.1.3) on Height = 0 m (at age 100), which means, depending on the site index curves, for each tree species a SI near -1 (red line).

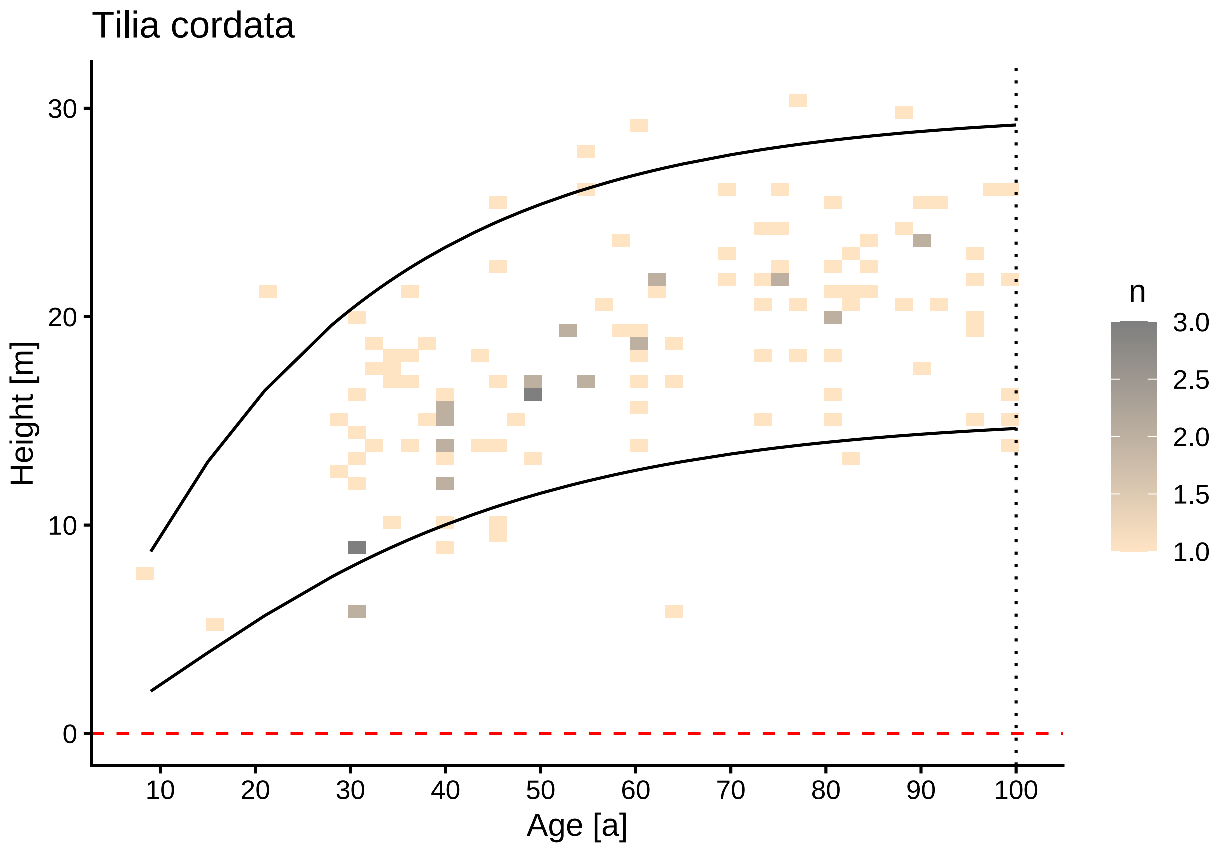

Model statistics and evaluation

Summary

Predictor acronyms: Bio.1 = Mean annual temperature [°C], Bio.12 = Annual precipitation sum [mm/m2], sp_p = Sum of precipitation [mm/m2] within months 3 to 5, su_p = Sum of precipitation [mm/m2] within months 6 to 8, wi_p = Sum of precipitation [mm/m2] within months 12,1,2, sp_t = Mean temperature [°C] within months 3 to 5, su_t = Mean temperature [°C] within months 6 to 8, wi_t = Mean temperature [°C] within months 12,1,2.

```
##
## Family: gaussian
## Link function: identity
##
## Formula:
## H03 ~ s(reference_19812010_Bio.1, k = 3) + s(reference_19812010_su_p,
##     k = 3)
##
## Parametric coefficients:
##             Estimate Std. Error t value Pr(>|t|)
## (Intercept)  0.25585    0.03969   6.447 3.37e-09 ***
## ---
## Signif. codes:  0 '***' 0.001 '**' 0.01 '*' 0.05 '.' 0.1 ' ' 1
##
## Approximate significance of smooth terms:
##                                edf Ref.df     F  p-value
## s(reference_19812010_Bio.1) 1.976  1.999 27.10  < 2e-16 ***
## s(reference_19812010_su_p)  1.916  1.992 17.21 1.22e-06 ***
## ---
## Signif. codes:  0 '***' 0.001 '**' 0.01 '*' 0.05 '.' 0.1 ' ' 1
##
## R-sq.(adj) =   0.55   Deviance explained = 56.6%
## -REML = 70.135  Scale est. = 0.17642   n = 112
```

Variance inflation factor (VIF)

Predictor acronyms: Bio.1 = Mean annual temperature [°C], Bio.12 = Annual precipitation sum [mm/m2], sp_p = Sum of precipitation [mm/m2] within months 3 to 5, su_p = Sum of precipitation [mm/m2] within months 6 to 8, wi_p = Sum of precipitation [mm/m2] within months 12,1,2, sp_t = Mean temperature [°C] within months 3 to 5, su_t = Mean temperature [°C] within months 6 to 8, wi_t = Mean temperature [°C] within months 12,1,2.

```
##                    Variables      VIF
## 1 reference_19812010_Bio.1 1.898257
## 2  reference_19812010_su_p 1.898257
```

Correlation matrix

Correlation matrix between the predictor variables and the target variable in the model. Correlation coefficient according to PEARSON. Predictor acronyms: Bio.1 = Mean annual temperature [°C], Bio.12 = Annual precipitation sum [mm/m2], sp_p = Sum of precipitation [mm/m2] within months 3 to 5, su_p = Sum of precipitation [mm/m2] within months 6 to 8, wi_p = Sum of precipitation [mm/m2] within months 12,1,2, sp_t = Mean temperature [°C] within months 3 to 5, su_t = Mean temperature [°C] within months 6 to 8, wi_t = Mean temperature [°C] within months 12,1,2.

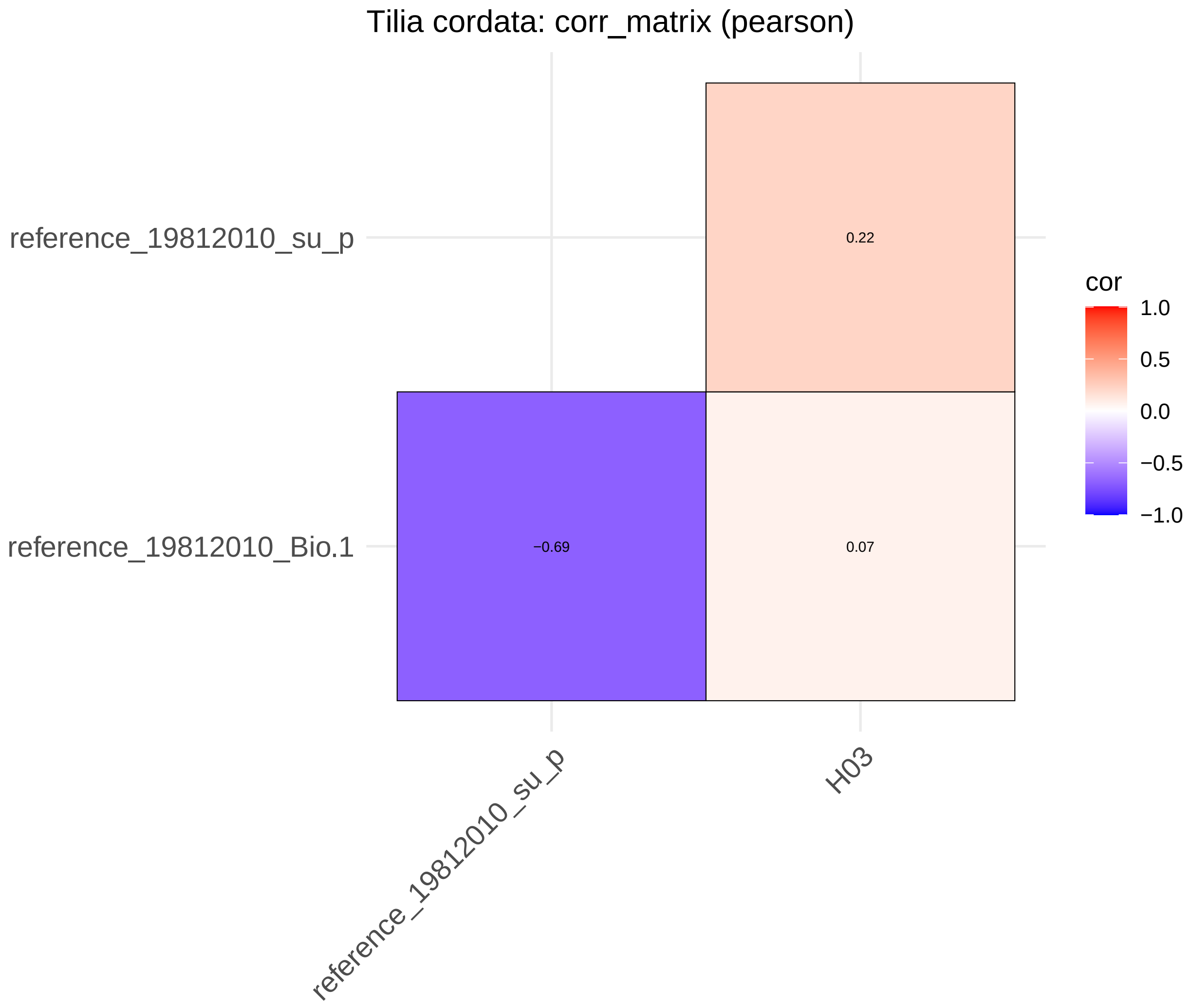

Response curves

Response curves (also known as effect curves) show how each predictor variable affects the target variable (H03 = european Site index, SIrel). H03 values below zero represent 'Growth absences'. Predictor acronyms: Bio.1 = Mean annual temperature [°C], Bio.12 = Annual precipitation sum [mm/m2], sp_p = Sum of precipitation [mm/m2] within months 3 to 5, su_p = Sum of precipitation [mm/m2] within months 6 to 8, wi_p = Sum of precipitation [mm/m2] within months 12,1,2, sp_t = Mean temperature [°C] within months 3 to 5, su_t = Mean temperature [°C] within months 6 to 8, wi_t = Mean temperature [°C] within months 12,1,2.

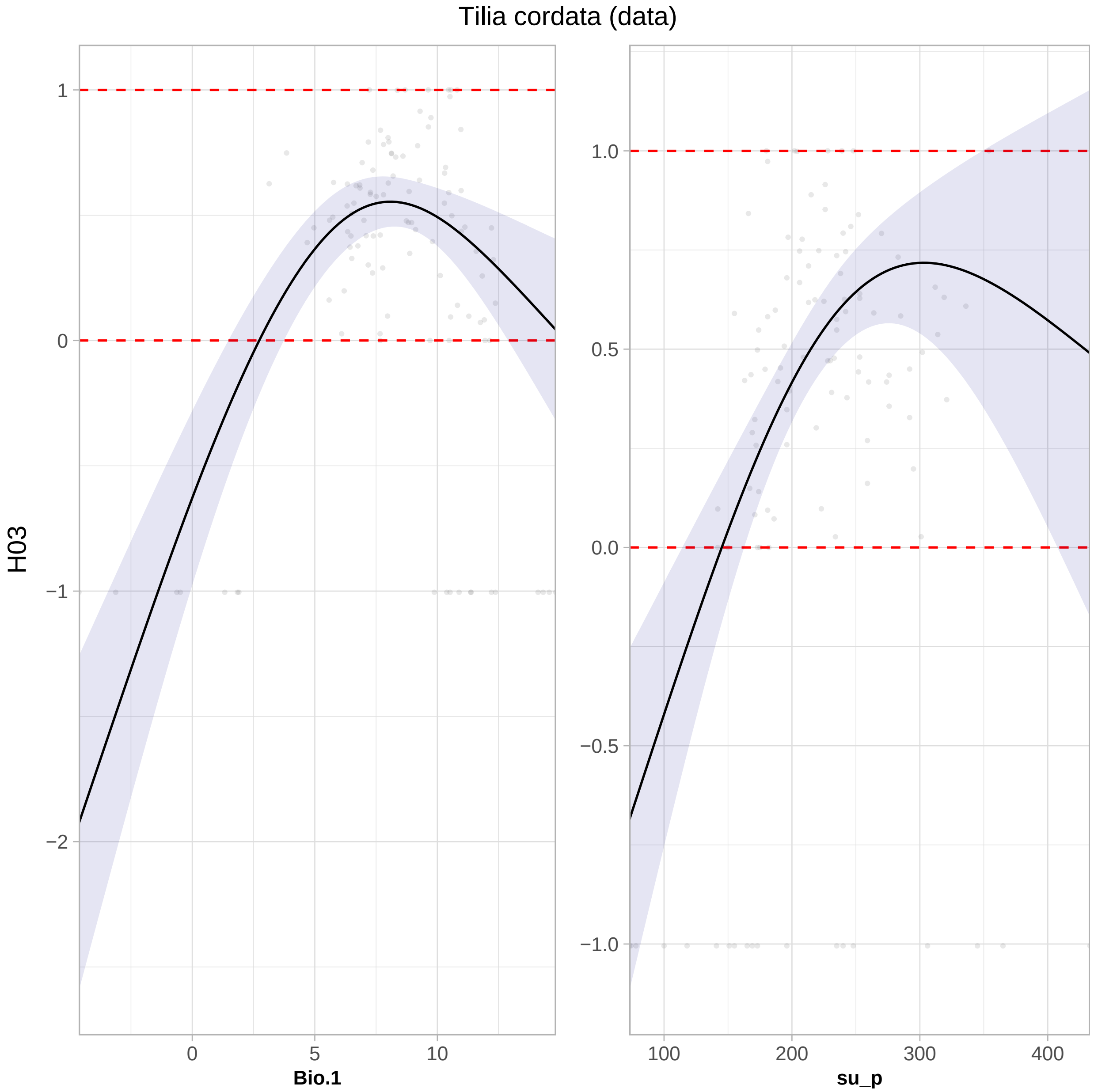

Response maps

Response maps (also referred as partial effect maps). Each map visualizes how a predictor affect the target variable (top height [m] at Age 100). Technically their work like response curves in a geographical area, that is setting all predictor variables except the one shown in the figure on their mean, and mapping the prediction. Predictor acronyms: Bio.1 = Mean annual temperature [°C], Bio.12 = Annual precipitation sum [mm/m2], sp_p = Sum of precipitation [mm/m2] within months 3 to 5, su_p = Sum of precipitation [mm/m2] within months 6 to 8, wi_p = Sum of precipitation [mm/m2] within months 12,1,2, sp_t = Mean temperature [°C] within months 3 to 5, su_t = Mean temperature [°C] within months 6 to 8, wi_t = Mean temperature [°C] within months 12,1,2.

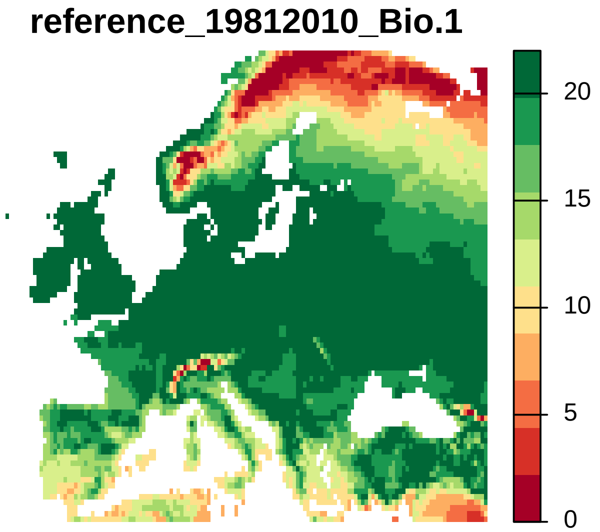

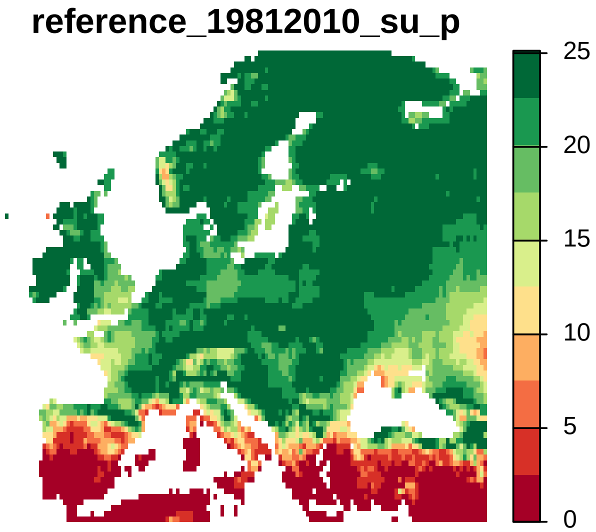

Residual distribution

The multi-panel plot includes a histogram of the residuals (top left), residuals over fitted values (top right), a histogram of observed and predicted values (bottom left) and boxplot diagram of observed and predicted values (bottom right). Observed values are shown in light green, while predicted ones are depicted in light red.

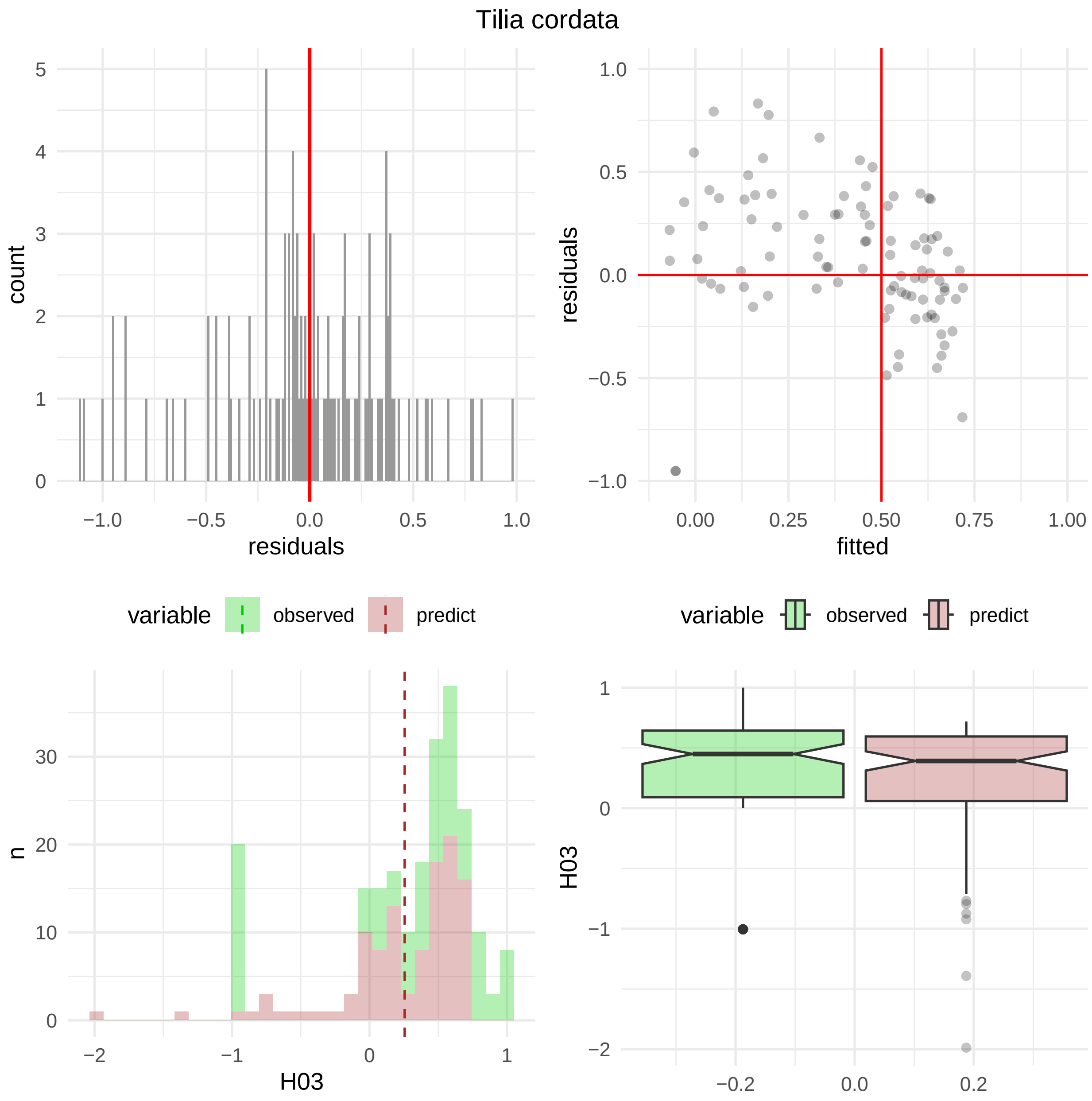

Correlation between predict and observed site index

Relationship between predicted and observed site index (density cloud), as well as linear regressions of presences and absences (= 'growth absences') (red line) and presences only (magenta line). The formulas, significance, R2 and number of observations are displayed below for both regressions. Ideally, both the point cloud and the regression lines lie close to the dashed line. For presences only we additionally calculated the correlation coefficient according to PEARSON (cor.pre) in the bottom right corner.

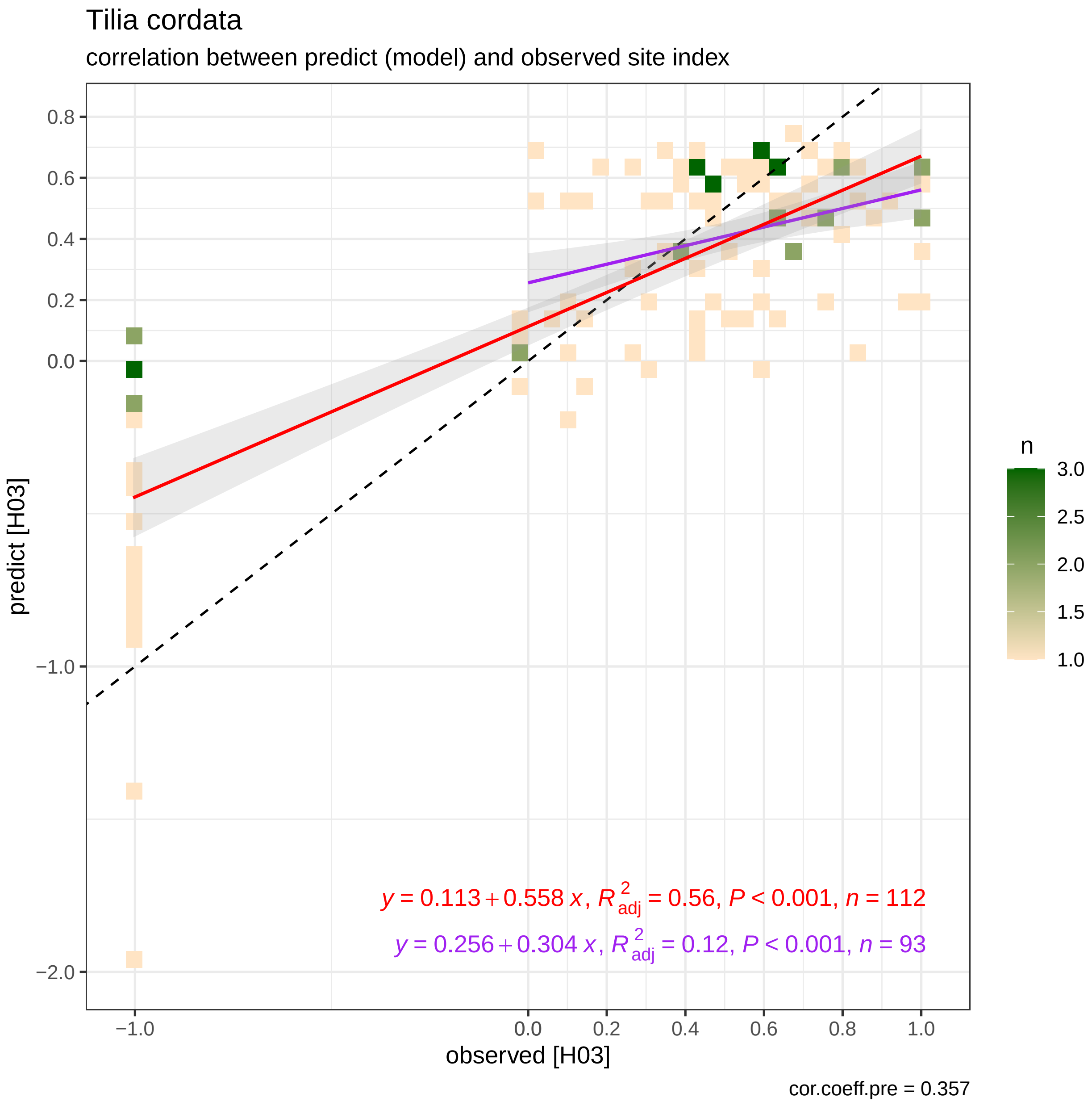

Predictions and forecasts

Predict

European predict for the reference period (1981 to 2010). Dark green symbolizes a high site index (tree height in meters at age 100), orange a lower site index and red no growth. Magenta-coloured dots represent inventory points with growth information, light blue dots are absences (= ‘growth absences’). Results were aggregated on 25 km x 25 km scale.

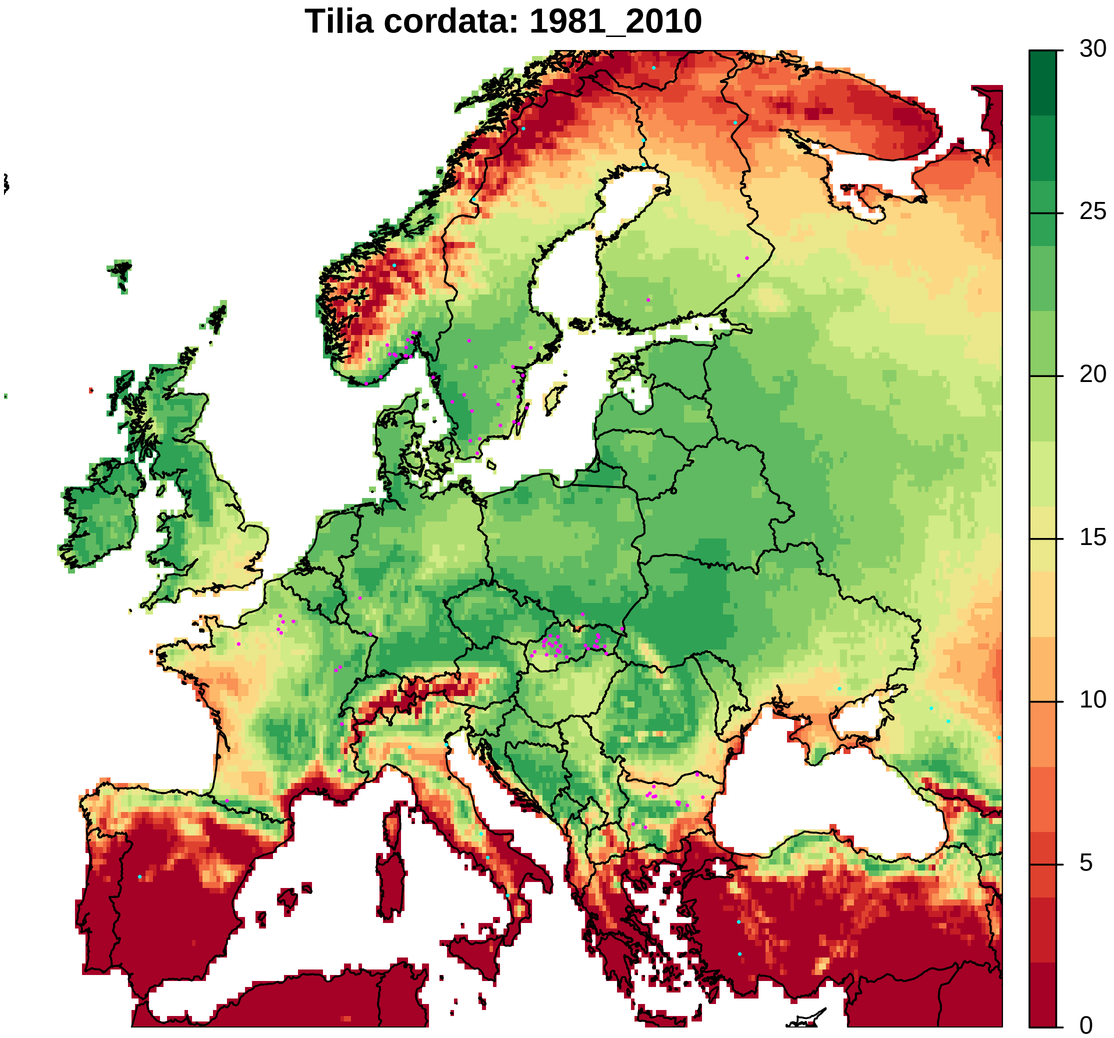

Forecast

Prediction for the reference period (1981 to 2010), as well as forecasts to 2071 to 2100 under szenario RCP4.5 and RCP8.5. Dark green symbolizes a high site index (tree height in m at age 100), orange a lower site index and red no growth. Results were aggregated on 25 km x 25 km scale.

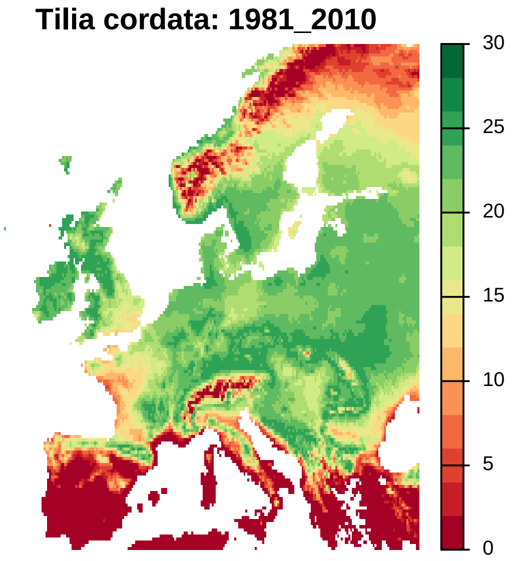

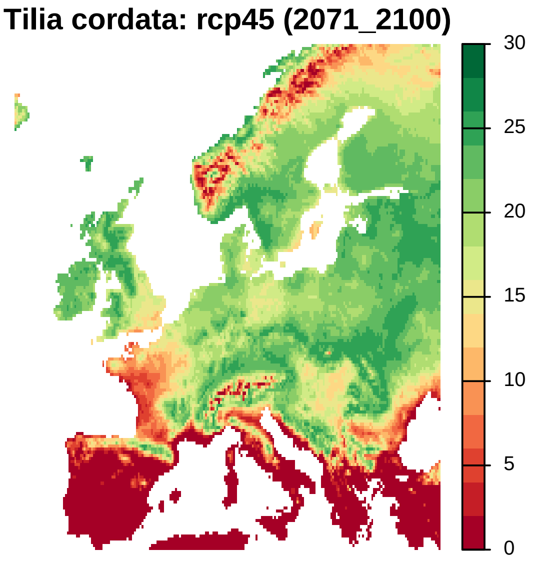

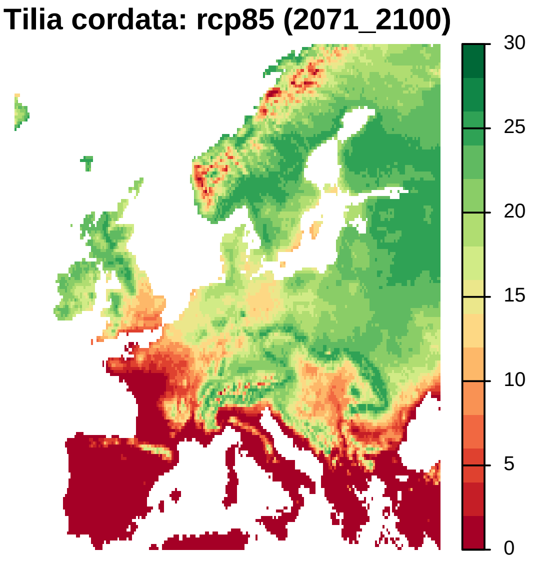

Supplement: Supplementary file 1 [file mmc1.pdf]
