# Supplementary material for: Pan-European maps and models of current and future tree species distributions and their growth potential
Source: Data Brief. 2026 Jun 27;67:113027. doi: 10.1016/j.dib.2026.113027 (PMC13355197; doi:10.1016/j.dib.2026.113027)

Supplementary material 2: Species distribution models

Wöhlbrandt et al. (2026)

Contents

SDM thresholds **12**

Abies alba **13**

Model statistics and evaluation . . . 13

Summary . . . 13

Evaluation parameter . . . 13

Response curves and response maps . . . 14

Response curves . . . 14

Response maps . . . 15

Model projections . . . 16

Projection with plotted input data . . . 16

Projections . . . 17

Climate risk classes . . . 18

Abies grandis **19**

Model statistics and evaluation . . . 19

Summary . . . 19

Evaluation parameter . . . 19

Response curves and response maps . . . 20

Response curves . . . 20

Response maps . . . 21

Model projections . . . 22

Projection with plotted input data . . . 22

Projections . . . 23

Climate risk classes . . . 24

Acer campestre **25**
Model statistics and evaluation 25
Summary 25
Evaluation parameter 25
Response curves and response maps 26
Response curves 26
Response maps 27
Model projections 28
Projection with plotted input data 28
Projections 29
Climate risk classes 30

Acer platanoides **31**
Model statistics and evaluation 31
Summary 31
Evaluation parameter 31
Response curves and response maps 32
Response curves 32
Response maps 33
Model projections 34
Projection with plotted input data 34
Projections 35
Climate risk classes 36

Acer pseudoplatanus **37**
Model statistics and evaluation 37
Summary 37
Evaluation parameter 37
Response curves and response maps 38
Response curves 38
Response maps 39
Model projections 40
Projection with plotted input data 40
Projections 41
Climate risk classes 42

Alnus glutinosa 43
Model statistics and evaluation 43
Summary 43
Evaluation parameter 43
Response curves and response maps 44
Response curves 44
Response maps 45
Model projections 46
Projection with plotted input data 46
Projections 47
Climate risk classes 48

Betula pendula 49
Model statistics and evaluation 49
Summary 49
Evaluation parameter 49
Response curves and response maps 50
Response curves 50
Response maps 51
Model projections 52
Projection with plotted input data 52
Projections 53
Climate risk classes 54

Carpinus betulus 55
Model statistics and evaluation 55
Summary 55
Evaluation parameter 55
Response curves and response maps 56
Response curves 56
Response maps 57
Model projections 58
Projection with plotted input data 58
Projections 59
Climate risk classes 60

Castanea sativa 61
Model statistics and evaluation . . . 61
Summary . . . 61
Evaluation parameter . . . 61
Response curves and response maps . . . 62
Response curves . . . 62
Response maps . . . 63
Model projections . . . 64
Projection with plotted input data . . . 64
Projections . . . 65
Climate risk classes . . . 66

Fagus sylvatica 67
Model statistics and evaluation . . . 67
Summary . . . 67
Evaluation parameter . . . 67
Response curves and response maps . . . 68
Response curves . . . 68
Response maps . . . 69
Model projections . . . 70
Projection with plotted input data . . . 70
Projections . . . 71
Climate risk classes . . . 72

Fraxinus excelsior 73
Model statistics and evaluation . . . 73
Summary . . . 73
Evaluation parameter . . . 73
Response curves and response maps . . . 74
Response curves . . . 74
Response maps . . . 75
Model projections . . . 76
Projection with plotted input data . . . 76
Projections . . . 77
Climate risk classes . . . 78

Juglans nigra **79**
Model statistics and evaluation 79
Summary 79
Evaluation parameter 79
Response curves and response maps 80
Response curves 80
Response maps 81
Model projections 82
Projection with plotted input data 82
Projections 83
Climate risk classes 84

Juglans regia **85**
Model statistics and evaluation 85
Summary 85
Evaluation parameter 85
Response curves and response maps 86
Response curves 86
Response maps 87
Model projections 88
Projection with plotted input data 88
Projections 89
Climate risk classes 90

Larix decidua **91**
Model statistics and evaluation 91
Summary 91
Evaluation parameter 91
Response curves and response maps 92
Response curves 92
Response maps 93
Model projections 94
Projection with plotted input data 94
Projections 95
Climate risk classes 96

Picea abies **97**
Model statistics and evaluation . . . 97
Summary . . . 97
Evaluation parameter . . . 97
Response curves and response maps . . . 98
Response curves . . . 98
Response maps . . . 99
Model projections . . . 100
Projection with plotted input data . . . 100
Projections . . . 101
Climate risk classes . . . 102

Pinus nigra **103**
Model statistics and evaluation . . . 103
Summary . . . 103
Evaluation parameter . . . 103
Response curves and response maps . . . 104
Response curves . . . 104
Response maps . . . 105
Model projections . . . 106
Projection with plotted input data . . . 106
Projections . . . 107
Climate risk classes . . . 108

Pinus sylvestris **109**
Model statistics and evaluation . . . 109
Summary . . . 109
Evaluation parameter . . . 109
Response curves and response maps . . . 110
Response curves . . . 110
Response maps . . . 111
Model projections . . . 112
Projection with plotted input data . . . 112
Projections . . . 113
Climate risk classes . . . 114

Prunus avium **115**
Model statistics and evaluation 115
Summary 115
Evaluation parameter 115
Response curves and response maps 116
Response curves 116
Response maps 117
Model projections 118
Projection with plotted input data 118
Projections 119
Climate risk classes 120

Pseudotsuga menziesii **121**
Model statistics and evaluation 121
Summary 121
Evaluation parameter 121
Response curves and response maps 122
Response curves 122
Response maps 123
Model projections 124
Projection with plotted input data 124
Projections 125
Climate risk classes 126

Pyrus pyraster **127**
Model statistics and evaluation 127
Summary 127
Evaluation parameter 127
Response curves and response maps 128
Response curves 128
Response maps 129
Model projections 130
Projection with plotted input data 130
Projections 131
Climate risk classes 132

Quercus cerris **133**
Model statistics and evaluation . . . 133
Summary . . . 133
Evaluation parameter . . . 133
Response curves and response maps . . . 134
Response curves . . . 134
Response maps . . . 135
Model projections . . . 136
Projection with plotted input data . . . 136
Projections . . . 137
Climate risk classes . . . 138

Quercus petraea **139**
Model statistics and evaluation . . . 139
Summary . . . 139
Evaluation parameter . . . 139
Response curves and response maps . . . 140
Response curves . . . 140
Response maps . . . 141
Model projections . . . 142
Projection with plotted input data . . . 142
Projections . . . 143
Climate risk classes . . . 144

Quercus pubescens **145**
Model statistics and evaluation . . . 145
Summary . . . 145
Evaluation parameter . . . 145
Response curves and response maps . . . 146
Response curves . . . 146
Response maps . . . 147
Model projections . . . 148
Projection with plotted input data . . . 148
Projections . . . 149
Climate risk classes . . . 150

Quercus robur **151**
Model statistics and evaluation . . . 151
Summary . . . 151
Evaluation parameter . . . 151
Response curves and response maps . . . 152
Response curves . . . 152
Response maps . . . 153
Model projections . . . 154
Projection with plotted input data . . . 154
Projections . . . 155
Climate risk classes . . . 156

Quercus rubra **157**
Model statistics and evaluation . . . 157
Summary . . . 157
Evaluation parameter . . . 157
Response curves and response maps . . . 158
Response curves . . . 158
Response maps . . . 159
Model projections . . . 160
Projection with plotted input data . . . 160
Projections . . . 161
Climate risk classes . . . 162

Robinia pseudoacacia **163**
Model statistics and evaluation . . . 163
Summary . . . 163
Evaluation parameter . . . 163
Response curves and response maps . . . 164
Response curves . . . 164
Response maps . . . 165
Model projections . . . 166
Projection with plotted input data . . . 166
Projections . . . 167
Climate risk classes . . . 168

Sorbus aucuparia **169**
Model statistics and evaluation 169
Summary 169
Evaluation parameter 169
Response curves and response maps 170
Response curves 170
Response maps 171
Model projections 172
Projection with plotted input data 172
Projections 173
Climate risk classes 174

Sorbus torminalis **175**
Model statistics and evaluation 175
Summary 175
Evaluation parameter 175
Response curves and response maps 176
Response curves 176
Response maps 177
Model projections 178
Projection with plotted input data 178
Projections 179
Climate risk classes 180

Tilia cordata **181**
Model statistics and evaluation 181
Summary 181
Evaluation parameter 181
Response curves and response maps 182
Response curves 182
Response maps 183
Model projections 184
Projection with plotted input data 184
Projections 185
Climate risk classes 186

Ulmus laevis **187**
Model statistics and evaluation . . . 187
Summary . . . 187
Evaluation parameter . . . 187
Response curves and response maps . . . 188
Response curves . . . 188
Response maps . . . 189
Model projections . . . 190
Projection with plotted input data . . . 190
Projections . . . 191
Climate risk classes . . . 192

SDM thresholds

Table of SDM thresholds. To estimate the distribution potential of each species as a mask for the SIMs, the continuous SDM outputs were categorized into three classes: low, medium and high climatic risk. For this, SDM predictions were created using only the presences and subsequently reclassified using the quantiles 0.05 and 0.3 of the occurrence probability (OP) for each species. See subsection "Climate risk classes" in each species section for detailed maps of the risk classes. Risk classes: High climatic risk (OP value <= SDM_threshold_Q.05), medium climatic risk (SDM_threshold_Q.05 < OP value <= SDM_threshold_Q.3) and low climatic risk (SDM_threshold_Q.3 < OP value <= 1).

species	SDM_threshold_Q0.05	SDM_threshold_Q.3
Abies alba	0.31	0.61
Abies grandis	0.47	0.80
Acer campestre	0.38	0.72
Acer platanoides	0.34	0.63
Acer pseudoplatanus	0.37	0.68
Alnus glutinosa	0.39	0.63
Betula pendula	0.34	0.67
Carpinus betulus	0.37	0.75
Castanea sativa	0.37	0.80
Fagus sylvatica	0.38	0.75
Fraxinus excelsior	0.30	0.70
Juglans nigra	0.35	0.72
Juglans regia	0.32	0.69
Larix decidua	0.39	0.61
Picea abies	0.38	0.73
Pinus nigra	0.37	0.69
Pinus sylvestris	0.28	0.66
Prunus avium	0.33	0.69
Pseudotsuga menziesii	0.44	0.77
Pyrus pyraster	0.37	0.70
Quercus cerris	0.35	0.77
Quercus petraea	0.37	0.73
Quercus pubescens	0.39	0.73
Quercus robur	0.31	0.74
Quercus rubra	0.43	0.73
Robinia pseudoacacia	0.41	0.80
Sorbus aucuparia	0.32	0.64
Sorbus torminalis	0.40	0.75
Tilia cordata	0.36	0.64
Ulmus laevis	0.22	0.67

Abies alba

Model statistics and evaluation

Summary

Predictor acronyms: Bio.10 = Mean temperature of warmest quarter [°C] within months 6 to 8, Bio.11 = Mean temperature of coldest quarter [°C] within months 12,1,2, Bio.12 = Annual precipitation sum [mm/m2], Bio.18 = Mean monthly precipitation amount of the warmest quarter [mm/m2] within months 6 to 8.

```
##
## Family: binomial
## Link function: logit
##
## Formula:
## ba.2 ~ s(Bio.10, k = 3) + s(Bio.11, k = 3) + s(Bio.18, k = 3)
##
## Parametric coefficients:
##             Estimate Std. Error z value Pr(>|z|)
## (Intercept)  -0.3029     0.0473  -6.404 1.51e-10 ***
## ---
## Signif. codes:  0 '***' 0.001 '**' 0.01 '*' 0.05 '.' 0.1 ' ' 1
##
## Approximate significance of smooth terms:
##             edf Ref.df Chi.sq p-value
## s(Bio.10) 1.996  2.000  221.2  <2e-16 ***
## s(Bio.11) 1.980  2.000  177.8  <2e-16 ***
## s(Bio.18) 1.929  1.995  455.9  <2e-16 ***
## ---
## Signif. codes:  0 '***' 0.001 '**' 0.01 '*' 0.05 '.' 0.1 ' ' 1
##
## R-sq.(adj) =   0.39   Deviance explained = 33.2%
## -REML = 2043.7  Scale est. = 1         n = 4380
```

Evaluation parameter

Model performance was assessed using four statistical parameters: the area under the receiver operating characteristic curve (AUC), the true skill statistic (TSS), sensitivity (probability of the model to correctly predict a true presence) and specificity (probability of the model to correctly predict a true absence).

```
##    Species_name  AUC       TSS sensitivity specificity
## tp   Abies alba 0.86 0.5447489   0.8310502   0.7136986
```

Response curves and response maps

Response curves

Response curves (also known as effect curves) give an overview of the climatic niche of a species by relating the occurrence probability to corresponding climatic values. Predictor acronyms: Bio.10 = Mean temperature of warmest quarter [°C] within months 6 to 8, Bio.11 = Mean temperature of coldest quarter [°C] within months 12,1,2, Bio.12 = Annual precipitation sum [mm/m2], Bio.18 = Mean monthly precipitation amount of the warmest quarter [mm/m2] within months 6 to 8. Lines on the x-axis mark the upper and lower limit of the used presences (red), the mean (bold black) and the median (bold blue).

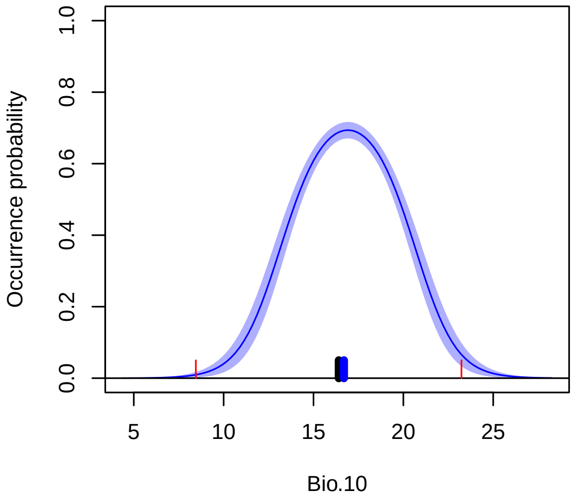

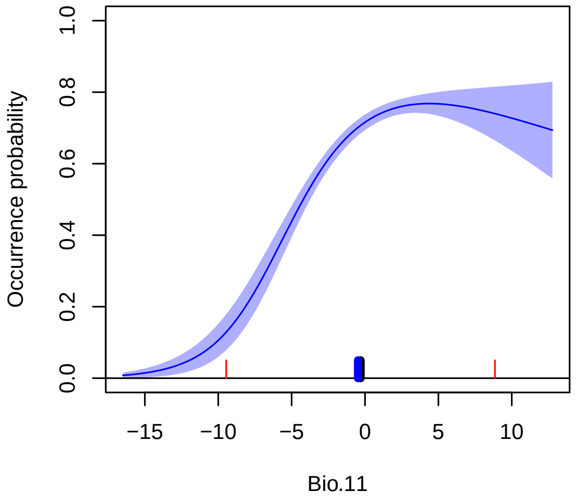

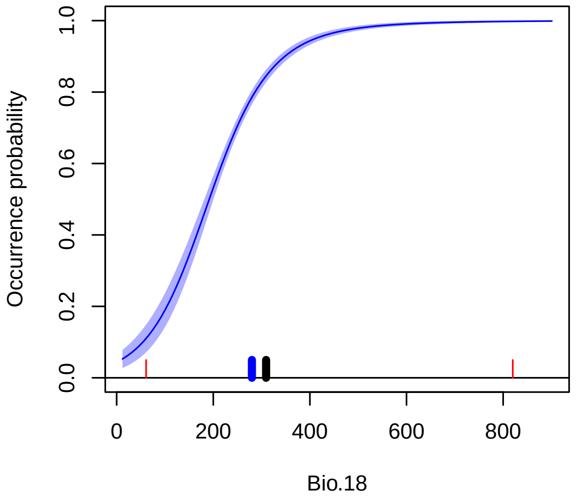

Response maps

Response maps (also referred to as partial effect maps). Each map represents how each predictor affects the occurrence probability. Predictor acronyms: Bio.10 = Mean temperature of warmest quarter [°C] within months 6 to 8, Bio.11 = Mean temperature of coldest quarter [°C] within months 12,1,2, Bio.12 = Annual precipitation sum [mm/m2], Bio.18 = Mean monthly precipitation amount of the warmest quarter [mm/m2] within months 6 to 8.

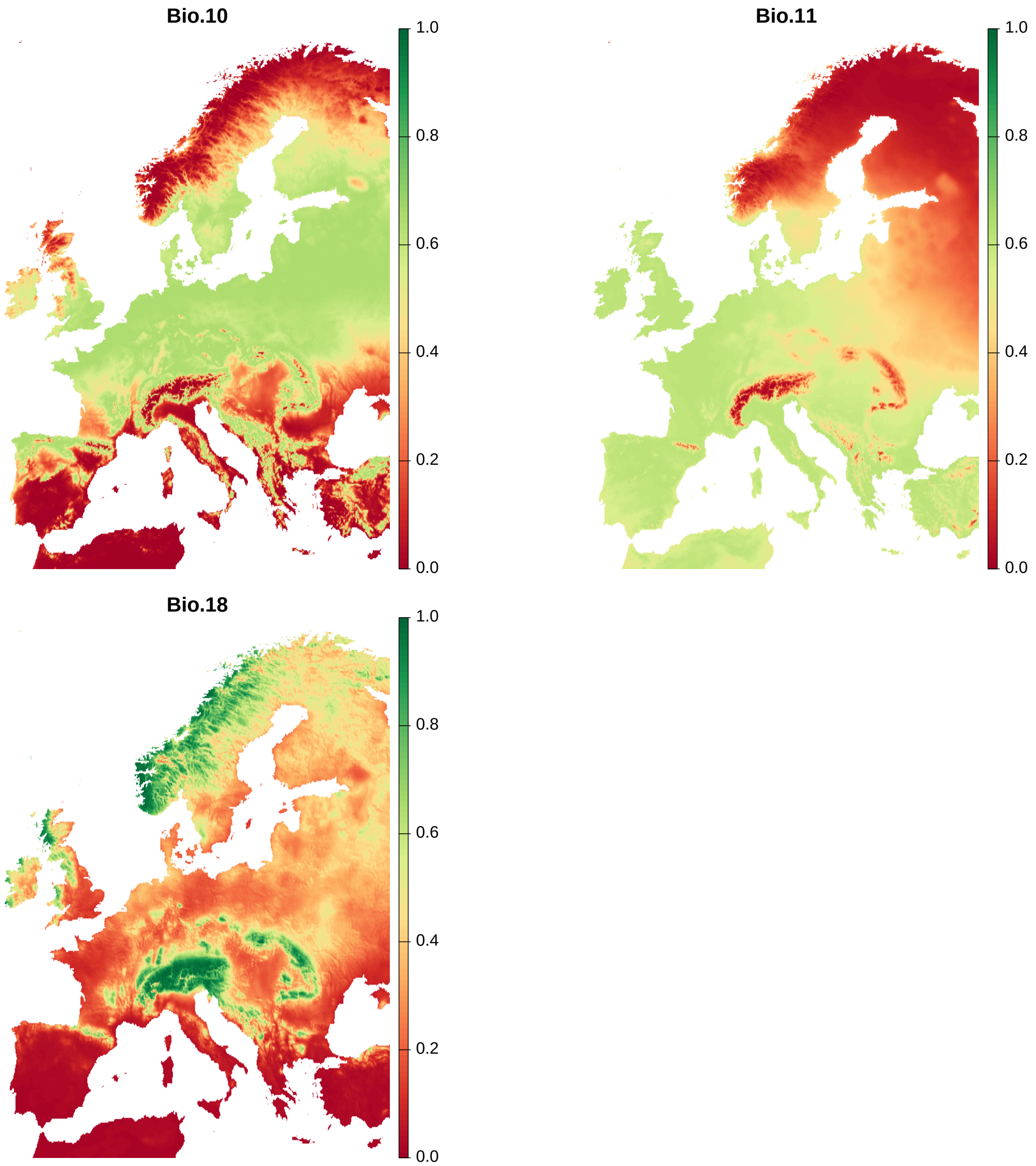

Model projections

Projection with plotted input data

Projection of species distribution model for reference period 1981-2010 over Europe. Occurrence probability ranges from 0 to 1 and is represented in dark red (low probability) to dark green (high probability). Input data used to calibrate the model is shown as presence points in magenta and absence points in black.

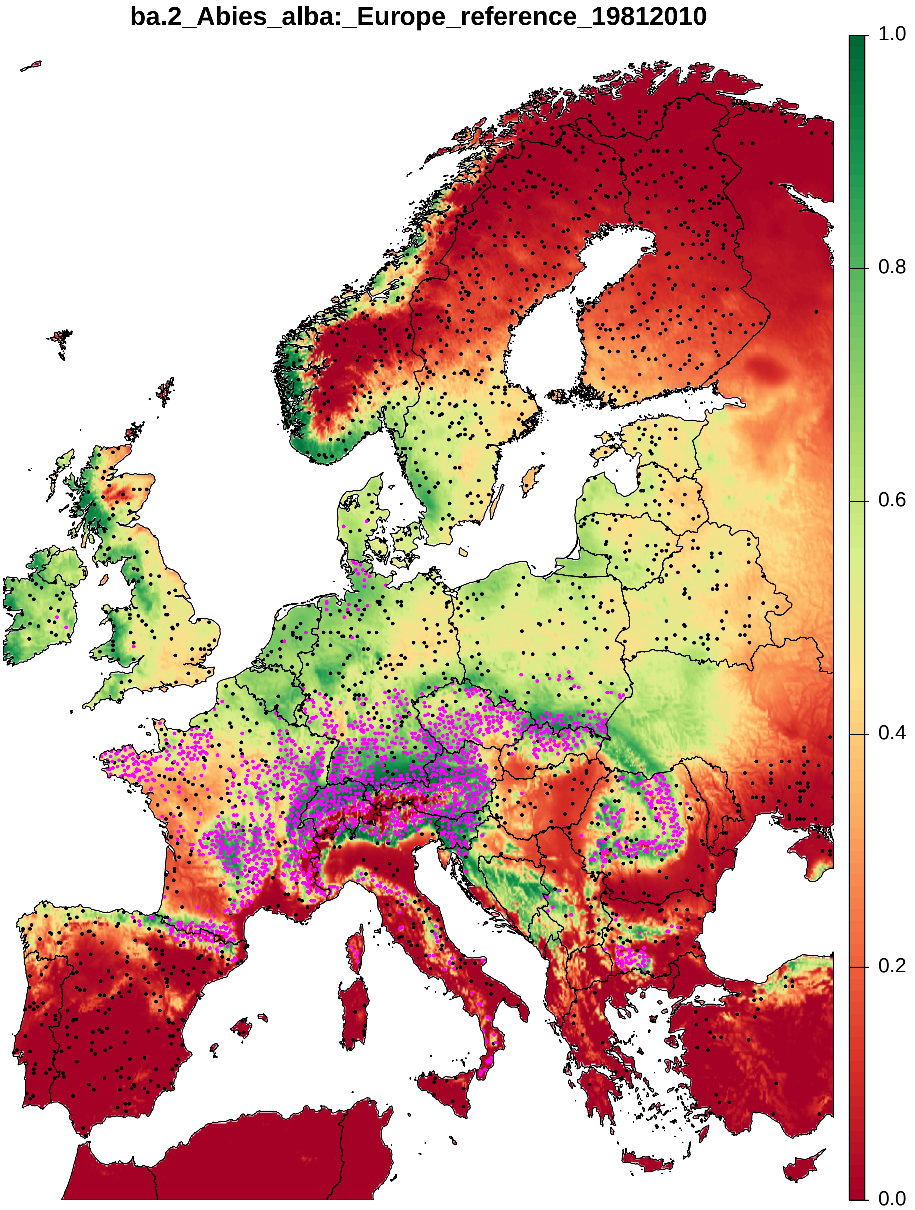

Projections

Projections of the species distribution models for reference period (1981-2010) and future scenarios RCP4.5 (2071-2100) and RCP8.5 (2071-2100) over Europe. Occurrence probabilities range from 0 to 1 and are represented from dark red (low probability) to dark green (high probability).

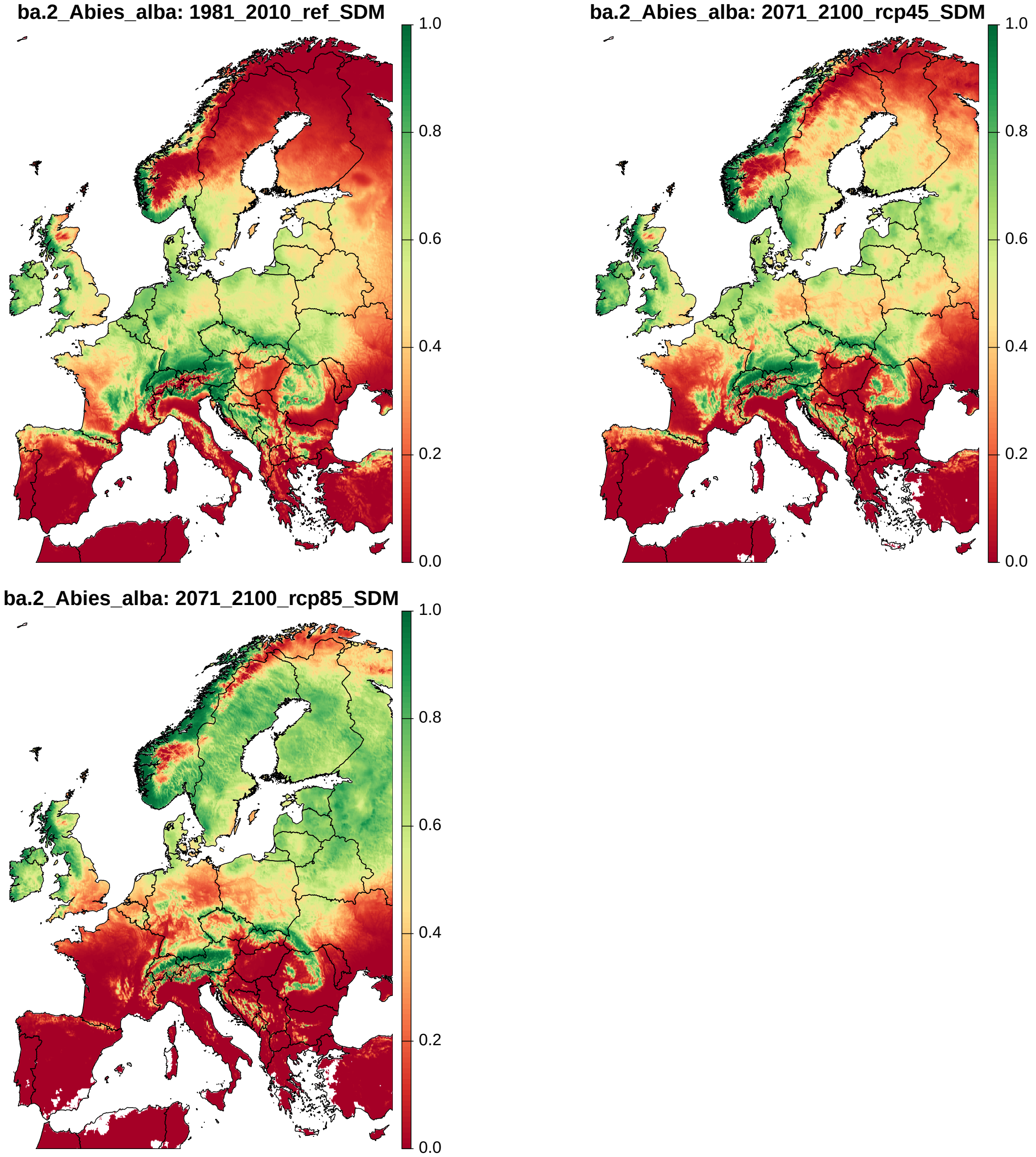

Climate risk classes

Maps of the climate risk classes. To estimate the distribution potential of each species as a mask for the SIMs, the continuous SDM outputs were categorized into three classes: low (yellow), medium (blue) and high climatic risk (red). The maps depict the risk classes in reference time (1981 to 2010), in climate scenario RCP4.5 (2071-2100) and RCP8.5 (2071-2100). To get an impression how well the thresholds fit to the data, presences (black) and absences (grey) were added on the reference map (top left). Refer to the legend and section “SDM thresholds” for the thresholds.

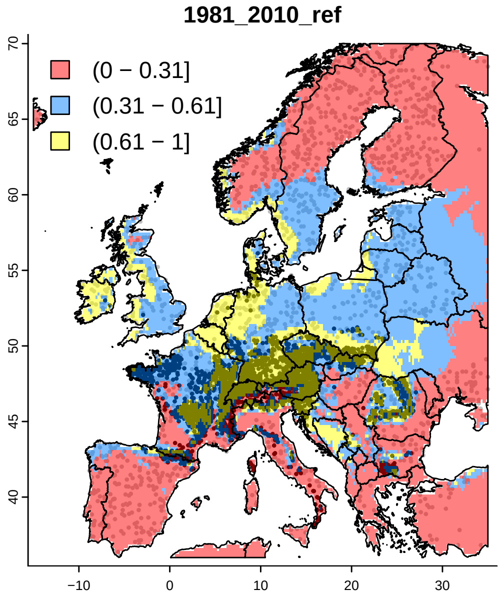

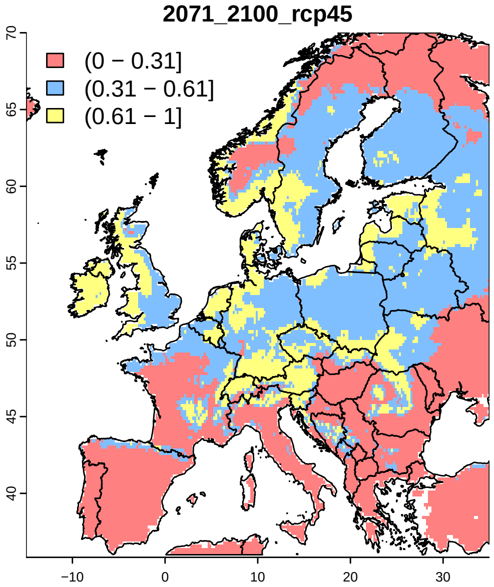

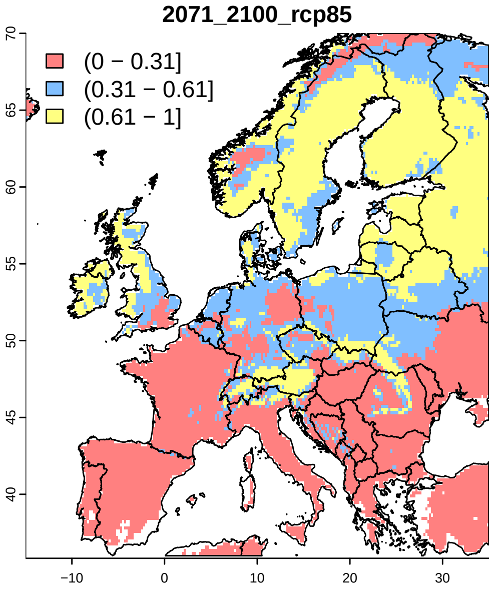

Abies grandis

Model statistics and evaluation

Summary

Predictor acronyms: Bio.10 = Mean temperature of warmest quarter [°C] within months 6 to 8, Bio.11 = Mean temperature of coldest quarter [°C] within months 12,1,2, Bio.12 = Annual precipitation sum [mm/m2], Bio.18 = Mean monthly precipitation amount of the warmest quarter [mm/m2] within months 6 to 8.

```
##
## Family: binomial
## Link function: logit
##
## Formula:
## ba.8 ~ s(Bio.10, k = 3) + s(Bio.11, k = 3) + s(Bio.18, k = 3)
##
## Parametric coefficients:
##             Estimate Std. Error z value Pr(>|z|)
## (Intercept)  -3.0995     0.3796  -8.164 3.23e-16 ***
## ---
## Signif. codes:  0 '***' 0.001 '**' 0.01 '*' 0.05 '.' 0.1 ' ' 1
##
## Approximate significance of smooth terms:
##             edf Ref.df Chi.sq  p-value
## s(Bio.10) 1.961  1.998  48.32  < 2e-16 ***
## s(Bio.11) 1.985  2.000 122.36  < 2e-16 ***
## s(Bio.18) 1.954  1.998  25.32 3.78e-06 ***
## ---
## Signif. codes:  0 '***' 0.001 '**' 0.01 '*' 0.05 '.' 0.1 ' ' 1
##
## R-sq.(adj) =   0.62   Deviance explained =   55%
## -REML = 320.63  Scale est. = 1         n = 994
```

Evaluation parameter

Model performance was assessed using four statistical parameters: the area under the receiver operating characteristic curve (AUC), the true skill statistic (TSS), sensitivity (probability of the model to correctly predict a true presence) and specificity (probability of the model to correctly predict a true absence).

```
##     Species_name  AUC       TSS sensitivity specificity
## tp Abies grandis 0.92 0.7424547   0.9416499   0.8008048
```

Response curves and response maps

Response curves

Response curves (also known as effect curves) give an overview of the climatic niche of a species by relating the occurrence probability to corresponding climatic values. Predictor acronyms: Bio.10 = Mean temperature of warmest quarter [°C] within months 6 to 8, Bio.11 = Mean temperature of coldest quarter [°C] within months 12,1,2, Bio.12 = Annual precipitation sum [mm/m2], Bio.18 = Mean monthly precipitation amount of the warmest quarter [mm/m2] within months 6 to 8. Lines on the x-axis mark the upper and lower limit of the used presences (red), the mean (bold black) and the median (bold blue).

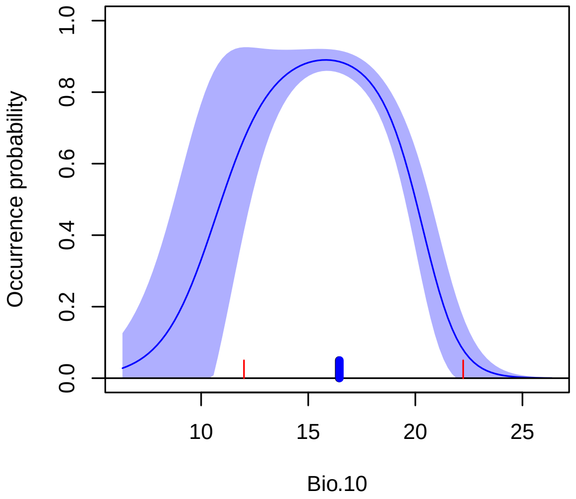

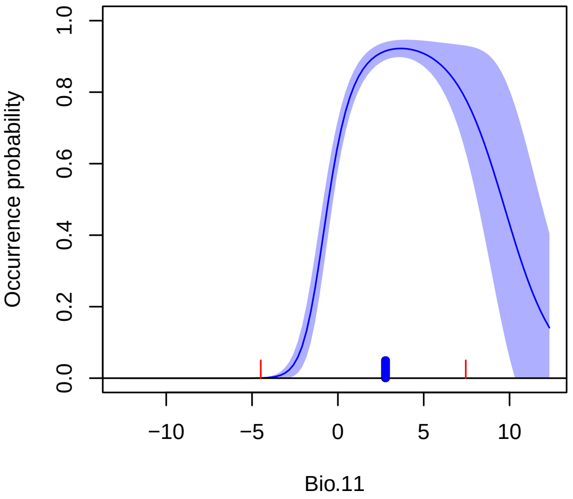

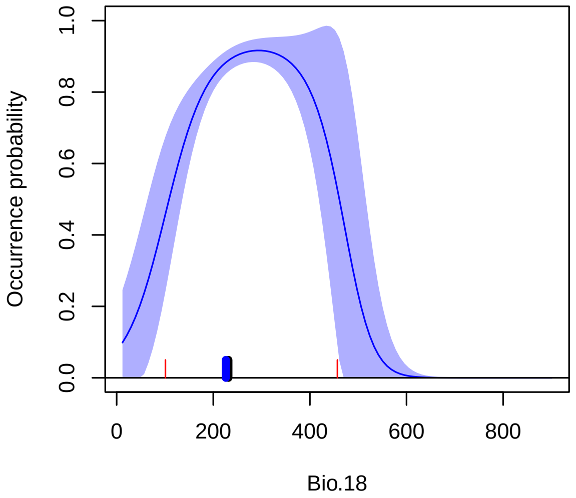

Response maps

Response maps (also referred to as partial effect maps). Each map represents how each predictor affects the occurrence probability. Predictor acronyms: Bio.10 = Mean temperature of warmest quarter [°C] within months 6 to 8, Bio.11 = Mean temperature of coldest quarter [°C] within months 12,1,2, Bio.12 = Annual precipitation sum [mm/m2], Bio.18 = Mean monthly precipitation amount of the warmest quarter [mm/m2] within months 6 to 8.

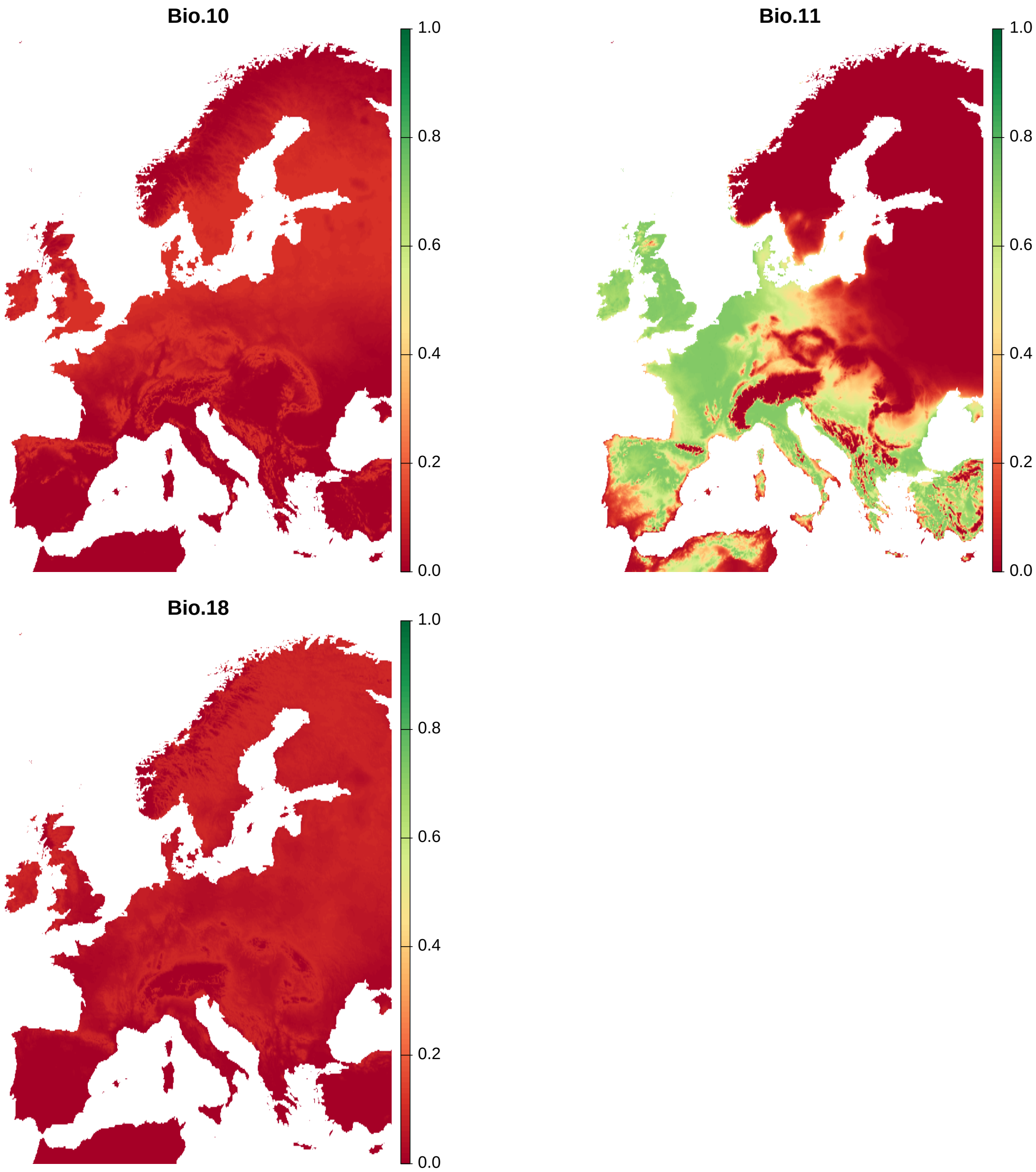

Model projections

Projection with plotted input data

Projection of species distribution model for reference period 1981-2010 over Europe. Occurrence probability ranges from 0 to 1 and is represented in dark red (low probability) to dark green (high probability). Input data used to calibrate the model is shown as presence points in magenta and absence points in black.

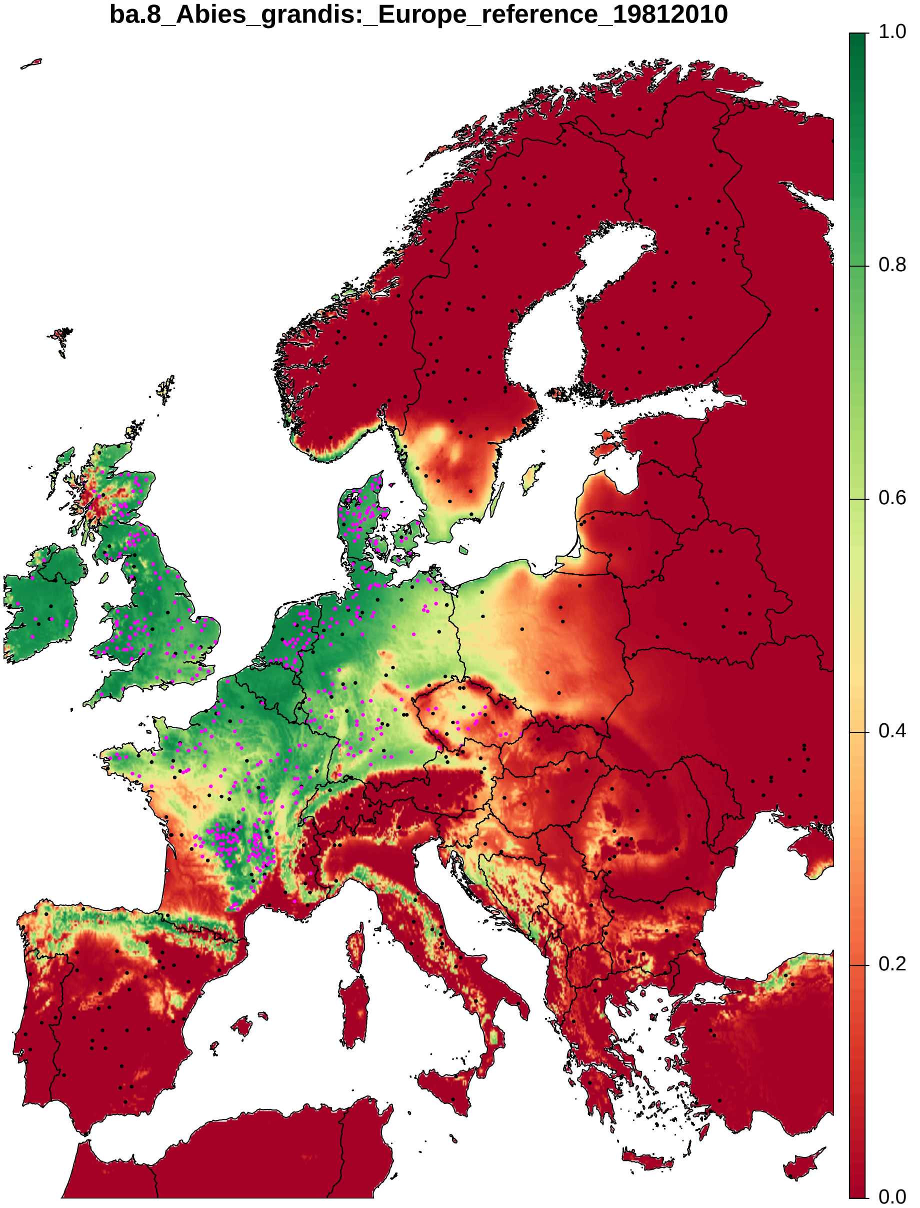

Projections

Projections of the species distribution models for reference period (1981-2010) and future scenarios RCP4.5 (2071-2100) and RCP8.5 (2071-2100) over Europe. Occurrence probabilities range from 0 to 1 and are represented from dark red (low probability) to dark green (high probability).

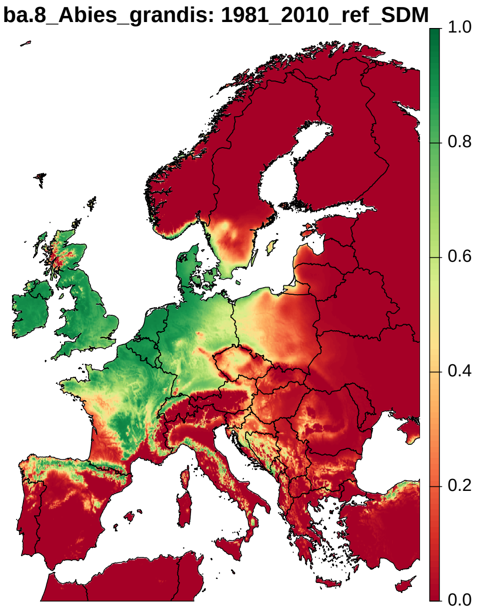

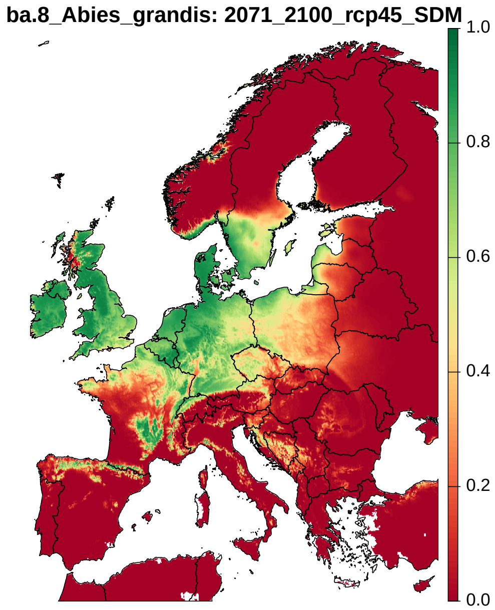

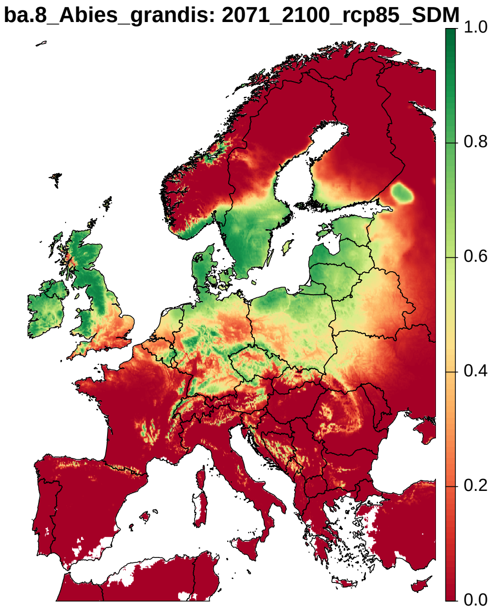

Climate risk classes

Maps of the climate risk classes. To estimate the distribution potential of each species as a mask for the SIMs, the continuous SDM outputs were categorized into three classes: low (yellow), medium (blue) and high climatic risk (red). The maps depict the risk classes in reference time (1981 to 2010), in climate scenario RCP4.5 (2071-2100) and RCP8.5 (2071-2100). To get an impression how well the thresholds fit to the data, presences (black) and absences (grey) were added on the reference map (top left). Refer to the legend and section “SDM thresholds” for the thresholds.

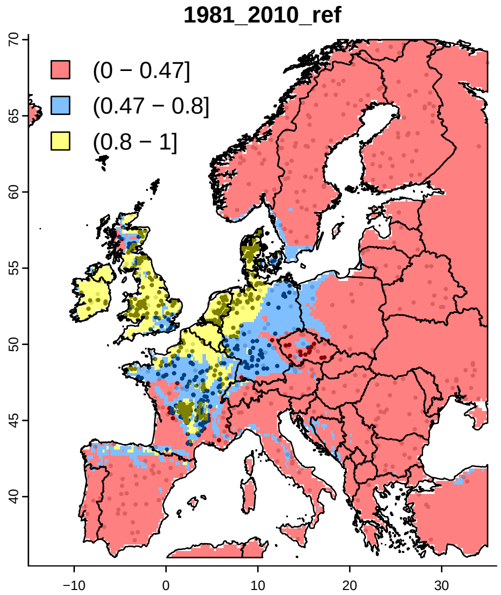

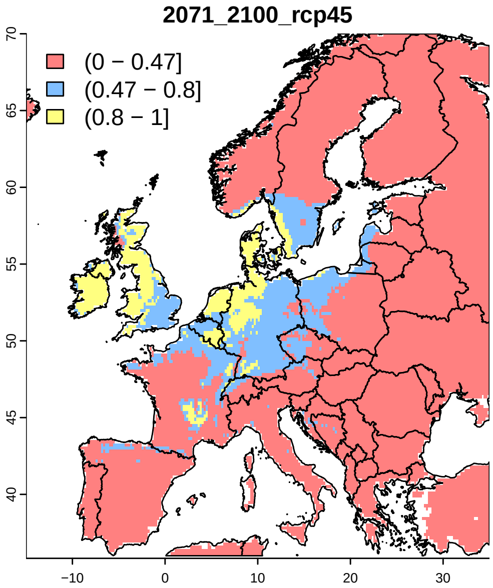

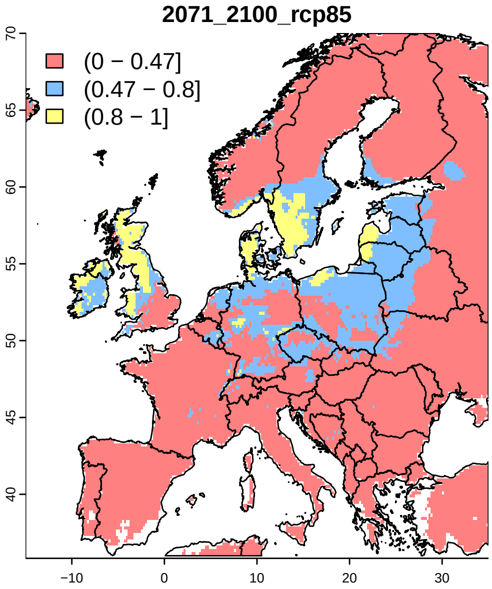

Acer campestre

Model statistics and evaluation

Summary

Predictor acronyms: Bio.10 = Mean temperature of warmest quarter [°C] within months 6 to 8, Bio.11 = Mean temperature of coldest quarter [°C] within months 12,1,2, Bio.12 = Annual precipitation sum [mm/m2], Bio.18 = Mean monthly precipitation amount of the warmest quarter [mm/m2] within months 6 to 8.

```
##
## Family: binomial
## Link function: logit
##
## Formula:
## ba.17 ~ s(Bio.10, k = 3) + s(Bio.11, k = 3) + s(Bio.18, k = 3)
##
## Parametric coefficients:
##             Estimate Std. Error z value Pr(>|z|)
## (Intercept) -2.10324    0.09569  -21.98   <2e-16 ***
## ---
## Signif. codes:  0 '***' 0.001 '**' 0.01 '*' 0.05 '.' 0.1 ' ' 1
##
## Approximate significance of smooth terms:
##             edf Ref.df Chi.sq p-value
## s(Bio.10) 1.998      2  603.0  <2e-16 ***
## s(Bio.11) 1.997      2  599.8  <2e-16 ***
## s(Bio.18) 1.989      2  263.4  <2e-16 ***
## ---
## Signif. codes:  0 '***' 0.001 '**' 0.01 '*' 0.05 '.' 0.1 ' ' 1
##
## R-sq.(adj) =  0.505   Deviance explained = 44.1%
## -REML = 2357.3  Scale est. = 1         n = 6040
```

Evaluation parameter

Model performance was assessed using four statistical parameters: the area under the receiver operating characteristic curve (AUC), the true skill statistic (TSS), sensitivity (probability of the model to correctly predict a true presence) and specificity (probability of the model to correctly predict a true absence).

```
##      Species_name  AUC      TSS sensitivity specificity
## tp Acer campestre 0.90 0.657947   0.8966887   0.7612583
```

Response curves and response maps

Response curves

Response curves (also known as effect curves) give an overview of the climatic niche of a species by relating the occurrence probability to corresponding climatic values. Predictor acronyms: Bio.10 = Mean temperature of warmest quarter [°C] within months 6 to 8, Bio.11 = Mean temperature of coldest quarter [°C] within months 12,1,2, Bio.12 = Annual precipitation sum [mm/m2], Bio.18 = Mean monthly precipitation amount of the warmest quarter [mm/m2] within months 6 to 8. Lines on the x-axis mark the upper and lower limit of the used presences (red), the mean (bold black) and the median (bold blue).

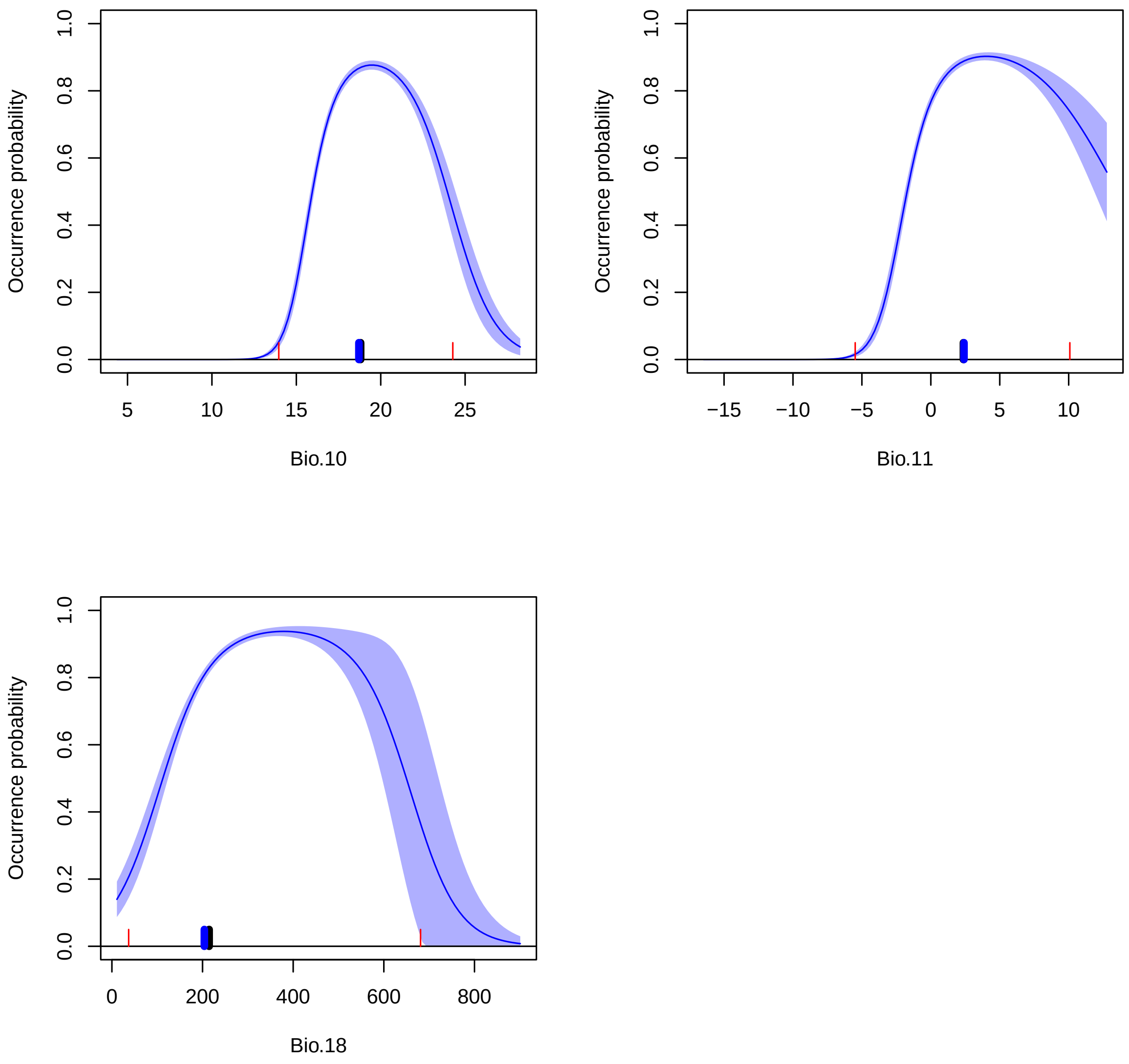

Response maps

Response maps (also referred to as partial effect maps). Each map represents how each predictor affects the occurrence probability. Predictor acronyms: Bio.10 = Mean temperature of warmest quarter [°C] within months 6 to 8, Bio.11 = Mean temperature of coldest quarter [°C] within months 12,1,2, Bio.12 = Annual precipitation sum [mm/m2], Bio.18 = Mean monthly precipitation amount of the warmest quarter [mm/m2] within months 6 to 8.

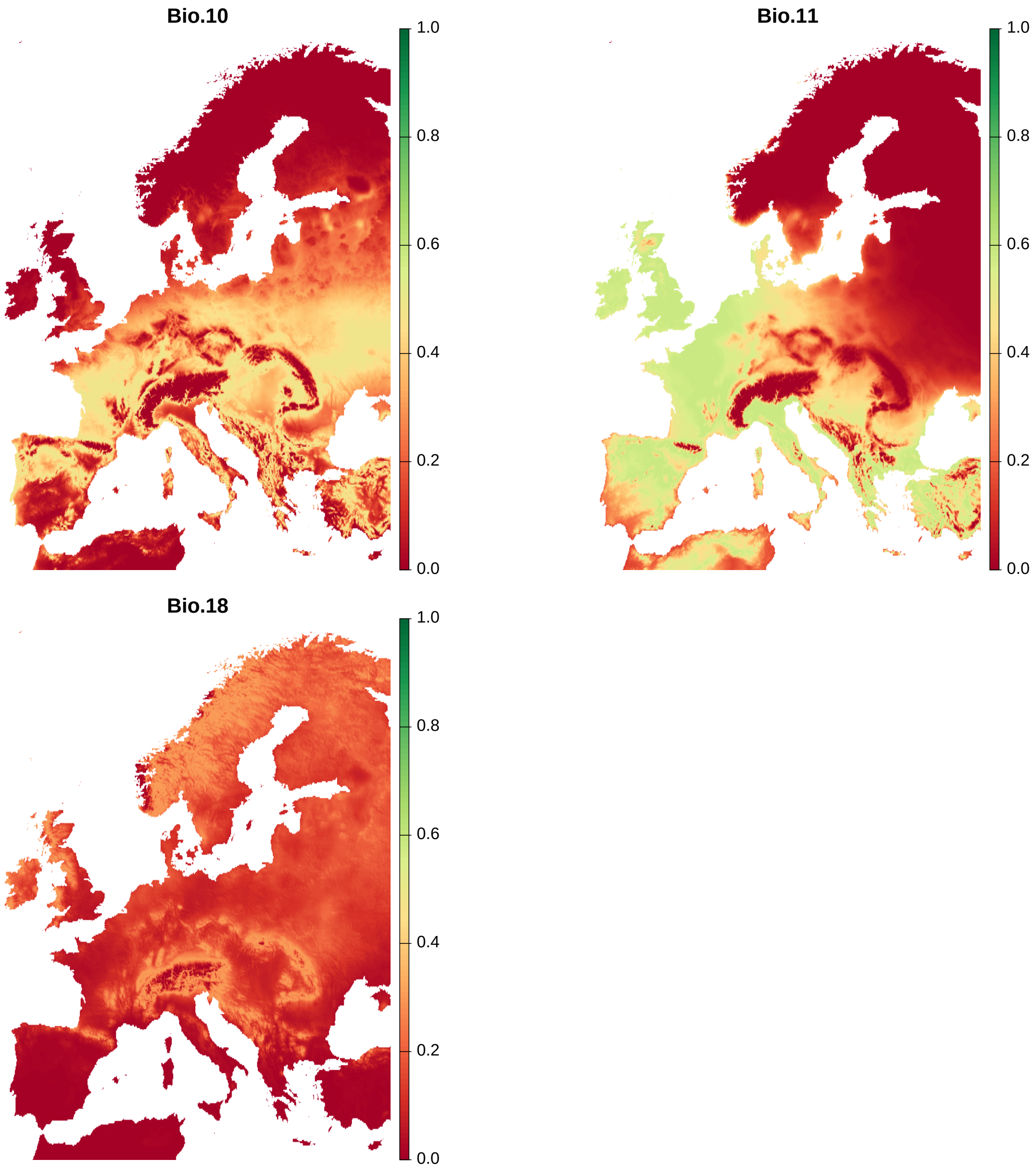

Model projections

Projection with plotted input data

Projection of species distribution model for reference period 1981-2010 over Europe. Occurrence probability ranges from 0 to 1 and is represented in dark red (low probability) to dark green (high probability). Input data used to calibrate the model is shown as presence points in magenta and absence points in black.

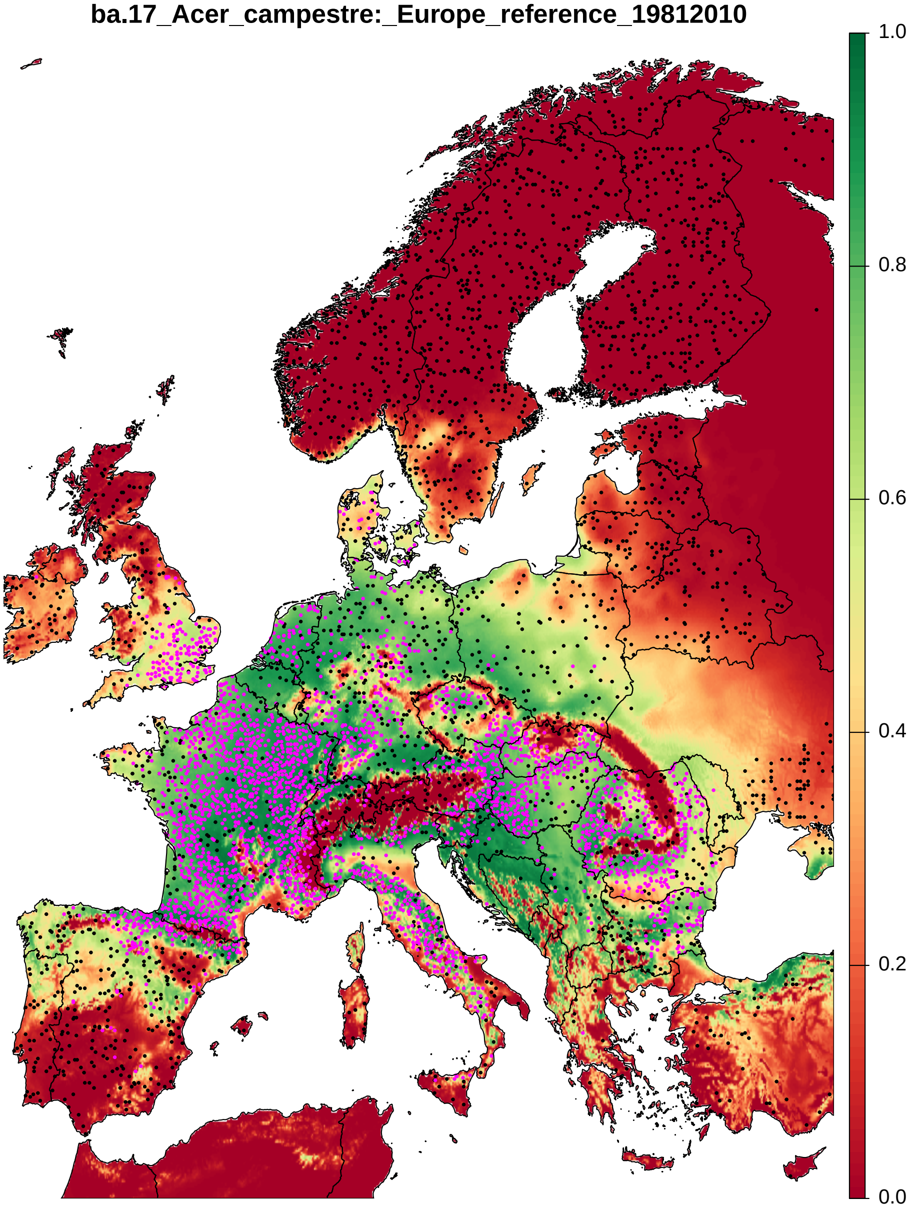

Projections

Projections of the species distribution models for reference period (1981-2010) and future scenarios RCP4.5 (2071-2100) and RCP8.5 (2071-2100) over Europe. Occurrence probabilities range from 0 to 1 and are represented from dark red (low probability) to dark green (high probability).

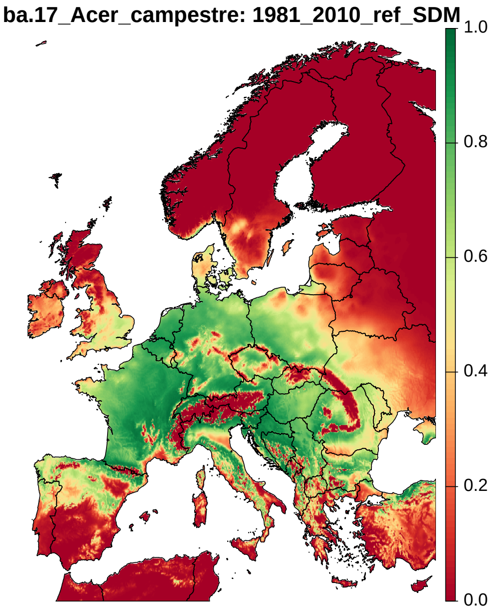

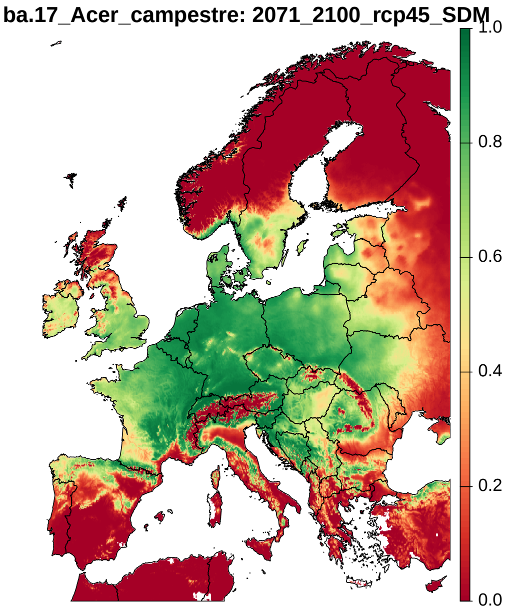

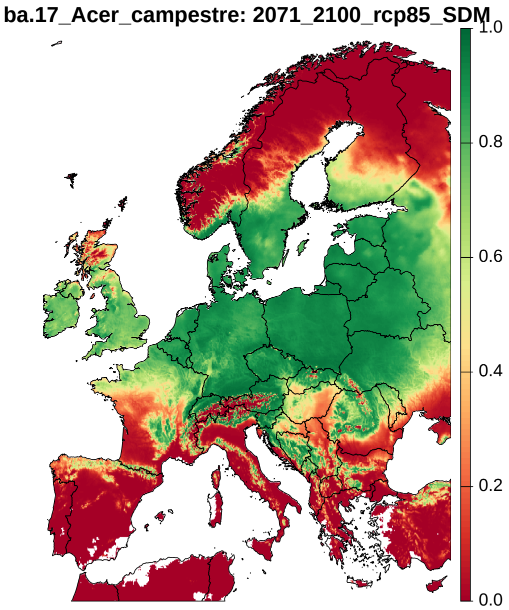

Climate risk classes

Maps of the climate risk classes. To estimate the distribution potential of each species as a mask for the SIMs, the continuous SDM outputs were categorized into three classes: low (yellow), medium (blue) and high climatic risk (red). The maps depict the risk classes in reference time (1981 to 2010), in climate scenario RCP4.5 (2071-2100) and RCP8.5 (2071-2100). To get an impression how well the thresholds fit to the data, presences (black) and absences (grey) were added on the reference map (top left). Refer to the legend and section "SDM thresholds" for the thresholds.

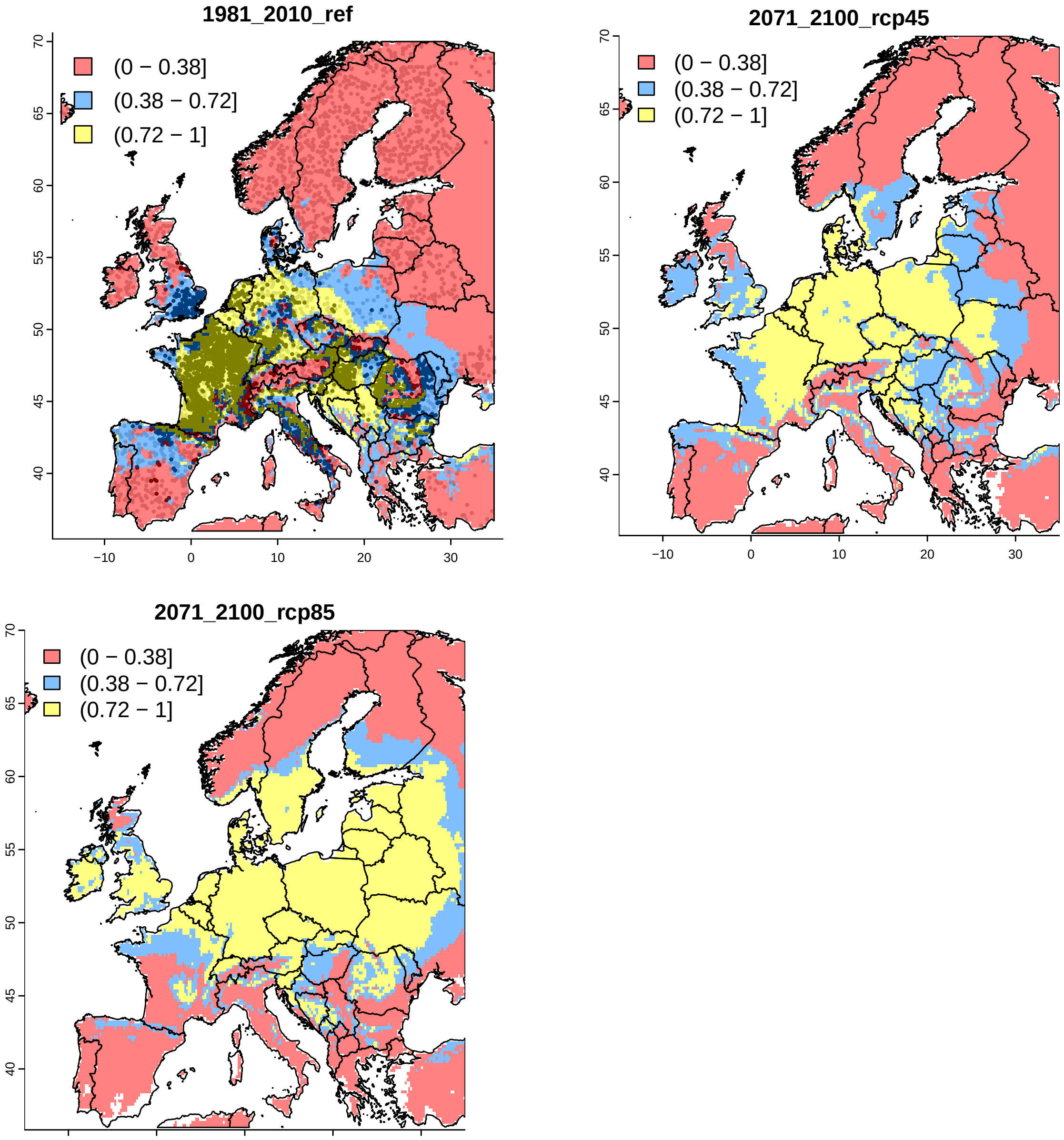

Acer platanoides

Model statistics and evaluation

Summary

Predictor acronyms: Bio.10 = Mean temperature of warmest quarter [°C] within months 6 to 8, Bio.11 = Mean temperature of coldest quarter [°C] within months 12,1,2, Bio.12 = Annual precipitation sum [mm/m2], Bio.18 = Mean monthly precipitation amount of the warmest quarter [mm/m2] within months 6 to 8.

```
##
## Family: binomial
## Link function: logit
##
## Formula:
## ba.16 ~ s(Bio.10, k = 3) + s(Bio.11, k = 3) + s(Bio.18, k = 3)
##
## Parametric coefficients:
##             Estimate Std. Error z value Pr(>|z|)
## (Intercept) -0.59682    0.05624  -10.61   <2e-16 ***
## ---
## Signif. codes:  0 '***' 0.001 '**' 0.01 '*' 0.05 '.' 0.1 ' ' 1
##
## Approximate significance of smooth terms:
##             edf Ref.df Chi.sq p-value
## s(Bio.10) 1.996  2.000  270.6  <2e-16 ***
## s(Bio.11) 1.994  2.000  170.8  <2e-16 ***
## s(Bio.18) 1.976  1.999  132.6  <2e-16 ***
## ---
## Signif. codes:  0 '***' 0.001 '**' 0.01 '*' 0.05 '.' 0.1 ' ' 1
##
## R-sq.(adj) =  0.363   Deviance explained = 31.1%
## -REML = 1863.8  Scale est. = 1         n = 3870
```

Evaluation parameter

Model performance was assessed using four statistical parameters: the area under the receiver operating characteristic curve (AUC), the true skill statistic (TSS), sensitivity (probability of the model to correctly predict a true presence) and specificity (probability of the model to correctly predict a true absence).

```
##        Species_name  AUC       TSS sensitivity specificity
## tp Acer platanoides 0.83 0.5674419   0.8904393   0.6770026
```

Response curves and response maps

Response curves

Response curves (also known as effect curves) give an overview of the climatic niche of a species by relating the occurrence probability to corresponding climatic values. Predictor acronyms: Bio.10 = Mean temperature of warmest quarter [°C] within months 6 to 8, Bio.11 = Mean temperature of coldest quarter [°C] within months 12,1,2, Bio.12 = Annual precipitation sum [mm/m2], Bio.18 = Mean monthly precipitation amount of the warmest quarter [mm/m2] within months 6 to 8. Lines on the x-axis mark the upper and lower limit of the used presences (red), the mean (bold black) and the median (bold blue).

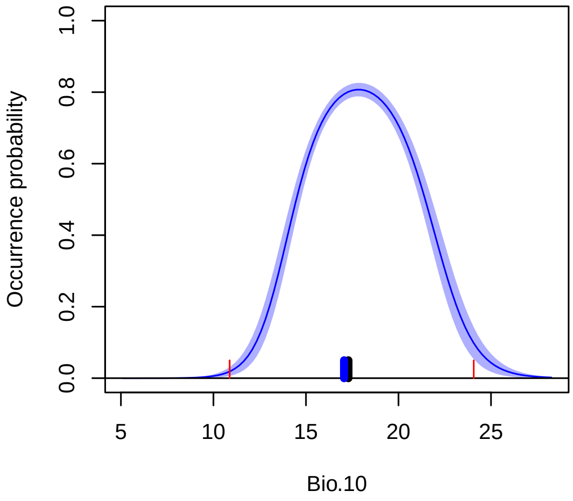

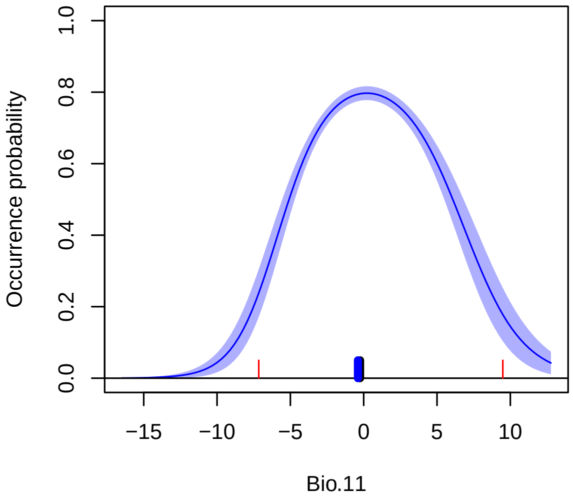

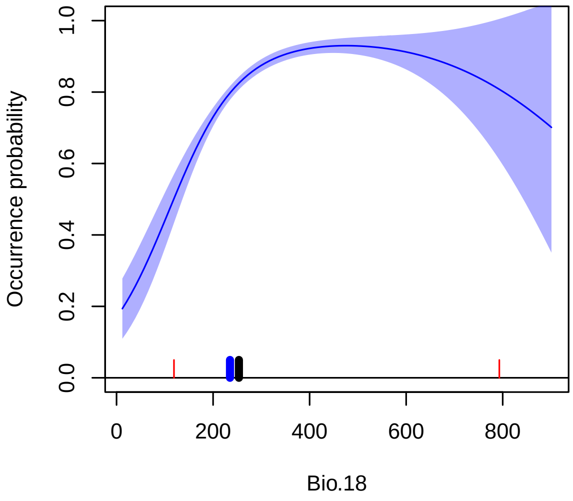

Response maps

Response maps (also referred to as partial effect maps). Each map represents how each predictor affects the occurrence probability. Predictor acronyms: Bio.10 = Mean temperature of warmest quarter [°C] within months 6 to 8, Bio.11 = Mean temperature of coldest quarter [°C] within months 12,1,2, Bio.12 = Annual precipitation sum [mm/m2], Bio.18 = Mean monthly precipitation amount of the warmest quarter [mm/m2] within months 6 to 8.

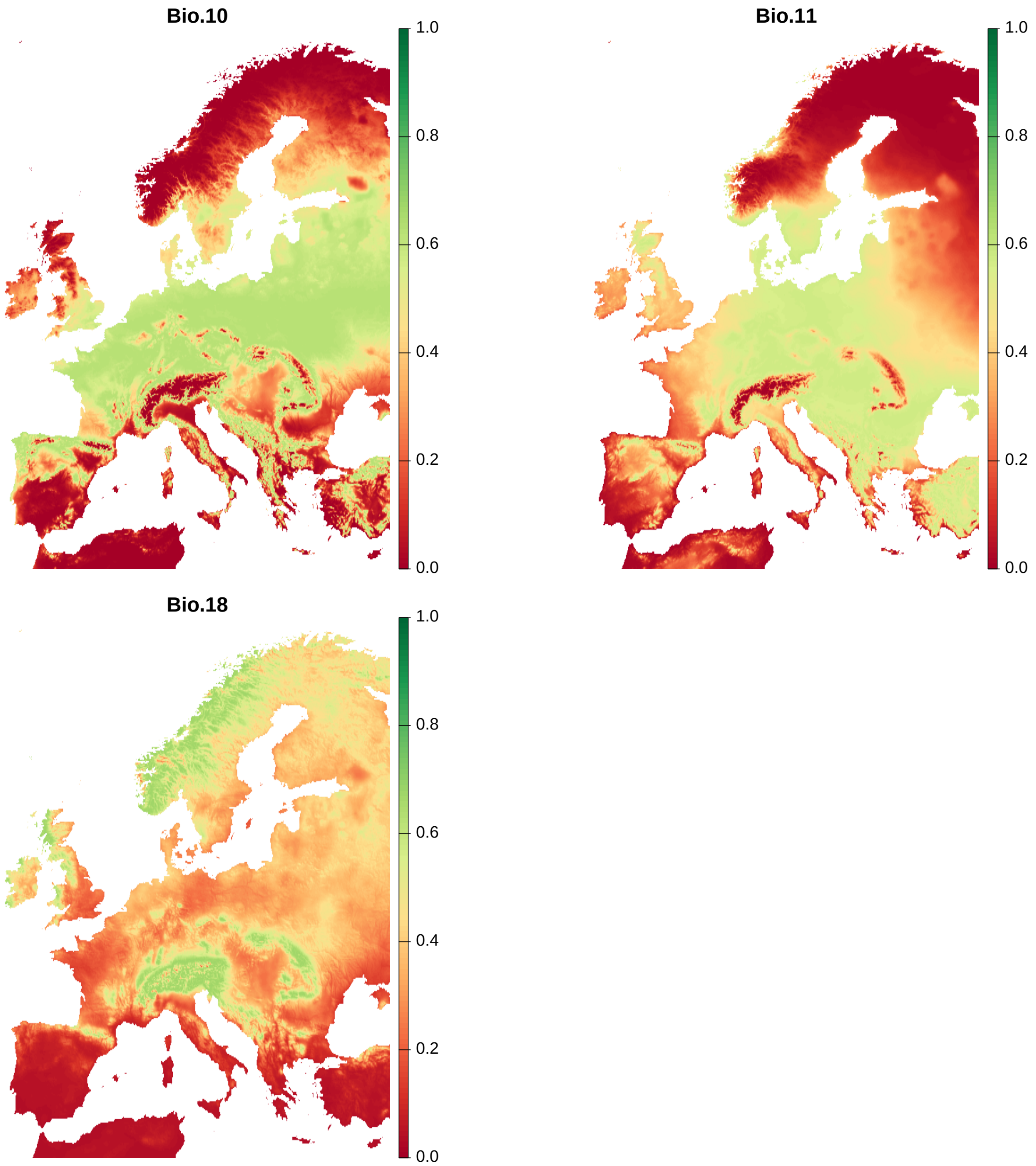

Model projections

Projection with plotted input data

Projection of species distribution model for reference period 1981-2010 over Europe. Occurrence probability ranges from 0 to 1 and is represented in dark red (low probability) to dark green (high probability). Input data used to calibrate the model is shown as presence points in magenta and absence points in black.

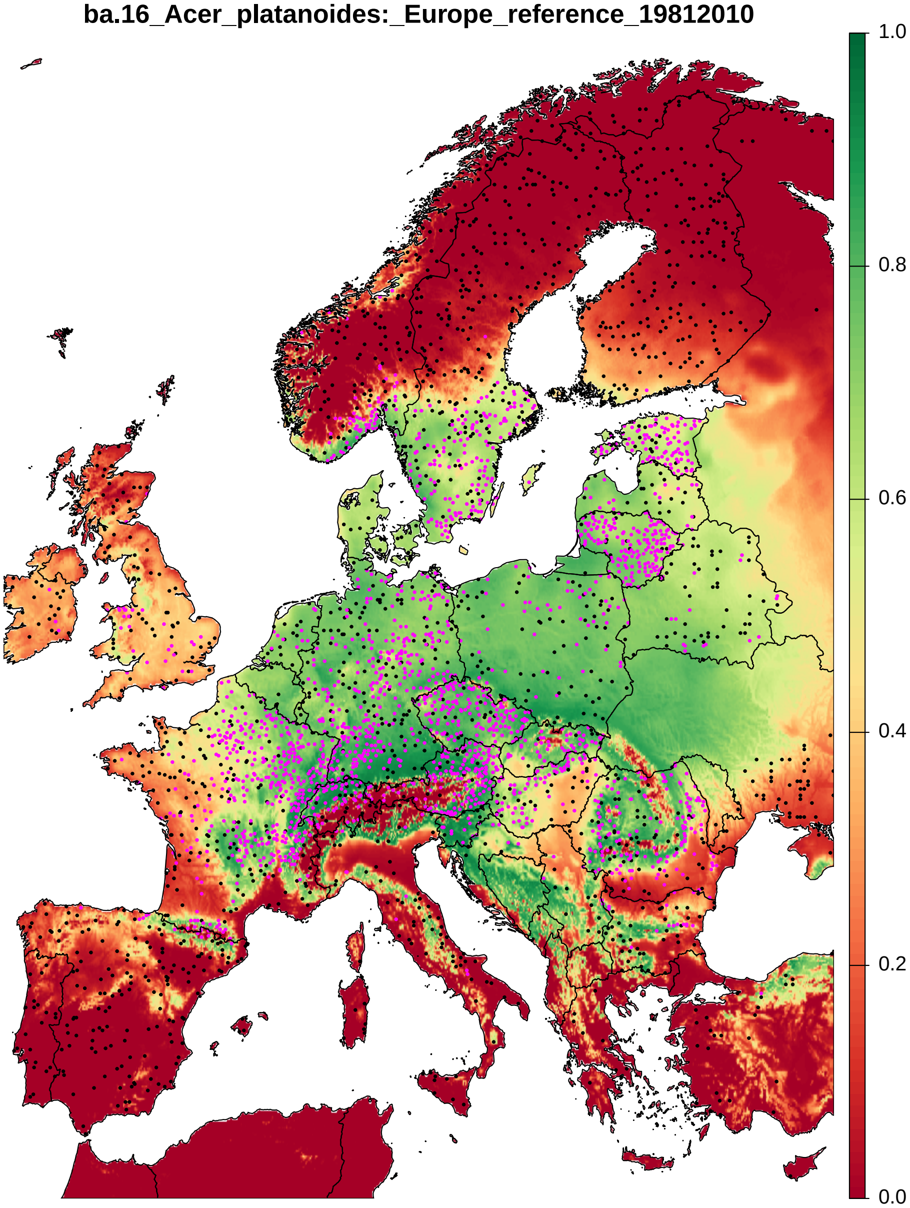

Projections

Projections of the species distribution models for reference period (1981-2010) and future scenarios RCP4.5 (2071-2100) and RCP8.5 (2071-2100) over Europe. Occurrence probabilities range from 0 to 1 and are represented from dark red (low probability) to dark green (high probability).

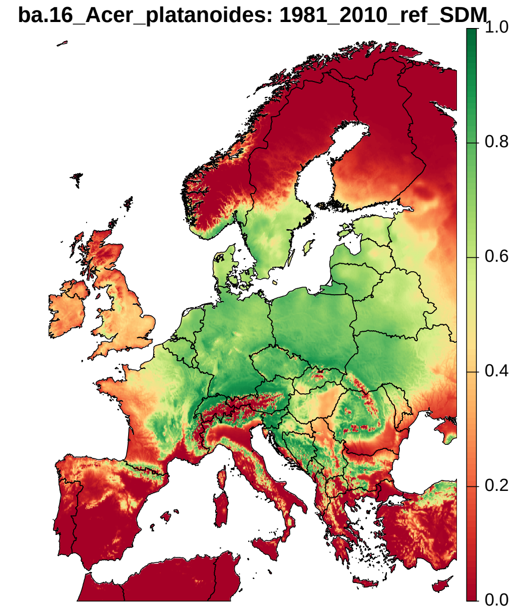

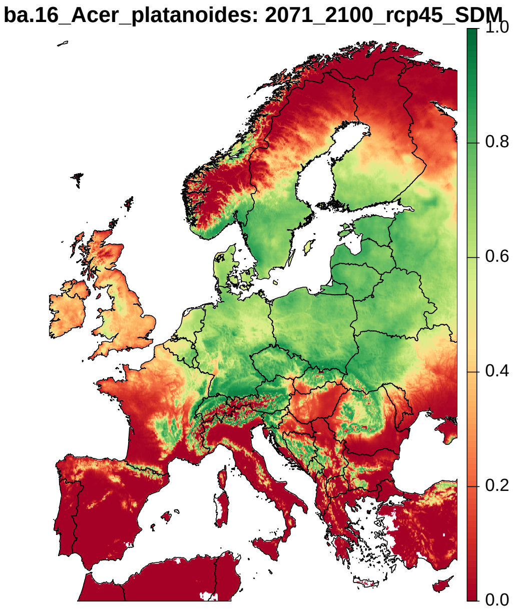

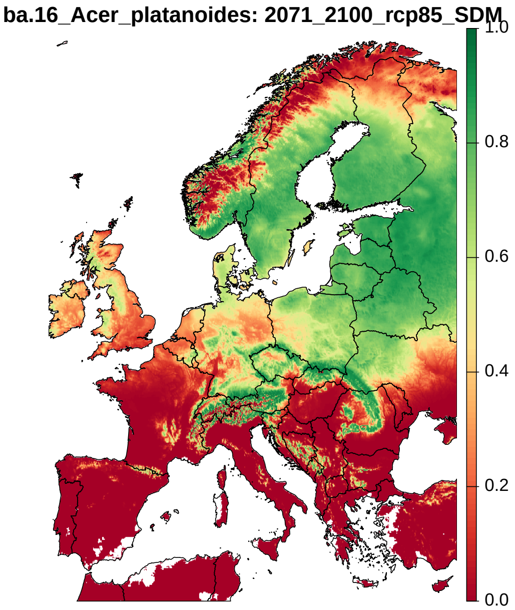

Climate risk classes

Maps of the climate risk classes. To estimate the distribution potential of each species as a mask for the SIMs, the continuous SDM outputs were categorized into three classes: low (yellow), medium (blue) and high climatic risk (red). The maps depict the risk classes in reference time (1981 to 2010), in climate scenario RCP4.5 (2071-2100) and RCP8.5 (2071-2100). To get an impression how well the thresholds fit to the data, presences (black) and absences (grey) were added on the reference map (top left). Refer to the legend and section "SDM thresholds" for the thresholds.

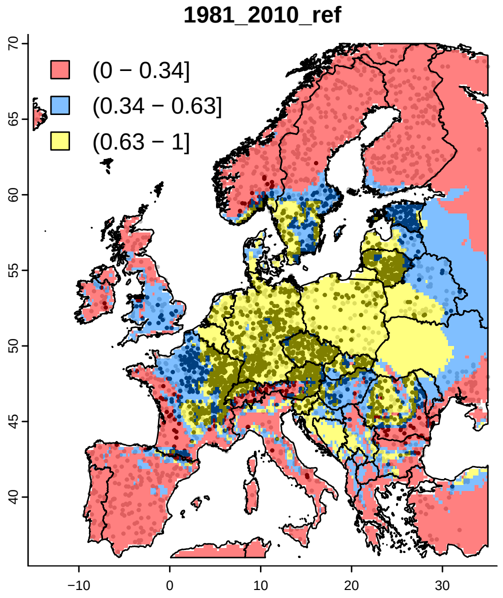

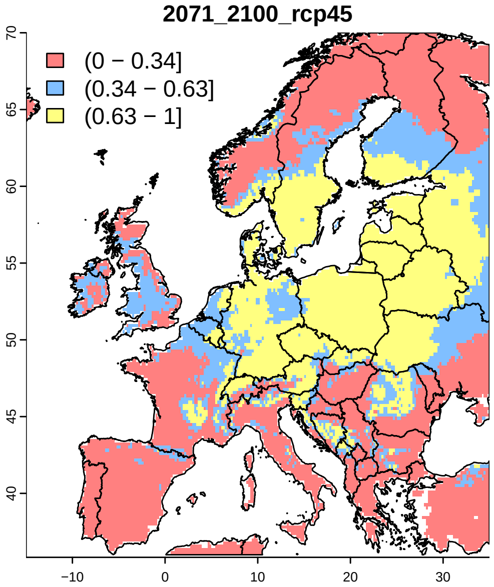

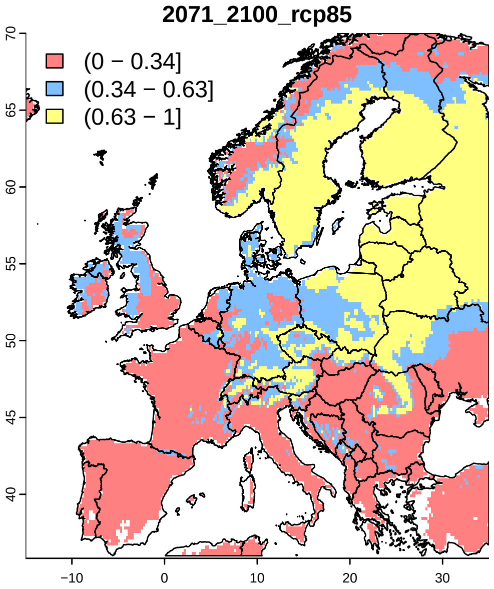

Acer pseudoplatanus

Model statistics and evaluation

Summary

Predictor acronyms: Bio.10 = Mean temperature of warmest quarter [°C] within months 6 to 8, Bio.11 = Mean temperature of coldest quarter [°C] within months 12,1,2, Bio.12 = Annual precipitation sum [mm/m2], Bio.18 = Mean monthly precipitation amount of the warmest quarter [mm/m2] within months 6 to 8.

```
##
## Family: binomial
## Link function: logit
##
## Formula:
## ba.15 ~ s(Bio.10, k = 3) + s(Bio.11, k = 3) + s(Bio.18, k = 3)
##
## Parametric coefficients:
##             Estimate Std. Error z value Pr(>|z|)
## (Intercept) -0.58392    0.04044  -14.44   <2e-16 ***
## ---
## Signif. codes:  0 '***' 0.001 '**' 0.01 '*' 0.05 '.' 0.1 ' ' 1
##
## Approximate significance of smooth terms:
##             edf Ref.df Chi.sq p-value
## s(Bio.10) 1.997  2.000  461.4  <2e-16 ***
## s(Bio.11) 1.996  2.000  957.2  <2e-16 ***
## s(Bio.18) 1.974  1.999  550.0  <2e-16 ***
## ---
## Signif. codes:  0 '***' 0.001 '**' 0.01 '*' 0.05 '.' 0.1 ' ' 1
##
## R-sq.(adj) =  0.446   Deviance explained =   37%
## -REML = 3839.7  Scale est. = 1         n = 8750
```

Evaluation parameter

Model performance was assessed using four statistical parameters: the area under the receiver operating characteristic curve (AUC), the true skill statistic (TSS), sensitivity (probability of the model to correctly predict a true presence) and specificity (probability of the model to correctly predict a true absence).

```
##           Species_name  AUC       TSS sensitivity specificity
## tp Acer pseudoplatanus 0.87 0.6377143   0.9069714   0.7307429
```

Response curves and response maps

Response curves

Response curves (also known as effect curves) give an overview of the climatic niche of a species by relating the occurrence probability to corresponding climatic values. Predictor acronyms: Bio.10 = Mean temperature of warmest quarter [°C] within months 6 to 8, Bio.11 = Mean temperature of coldest quarter [°C] within months 12,1,2, Bio.12 = Annual precipitation sum [mm/m2], Bio.18 = Mean monthly precipitation amount of the warmest quarter [mm/m2] within months 6 to 8. Lines on the x-axis mark the upper and lower limit of the used presences (red), the mean (bold black) and the median (bold blue).

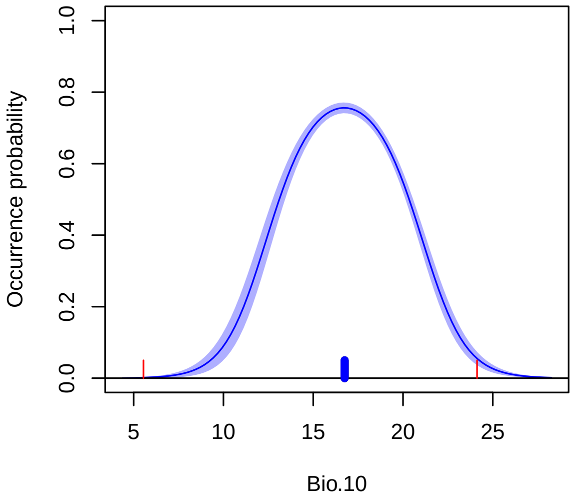

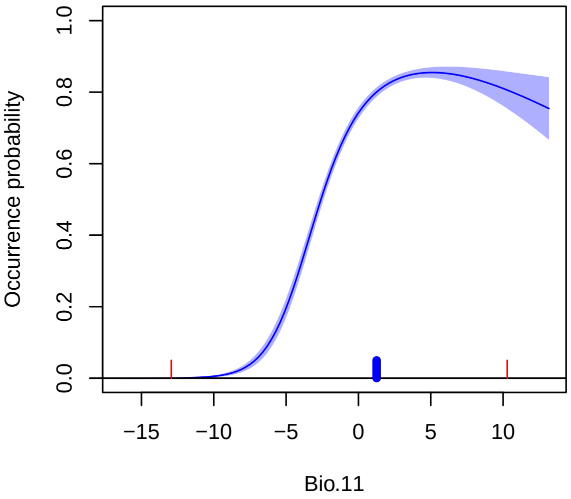

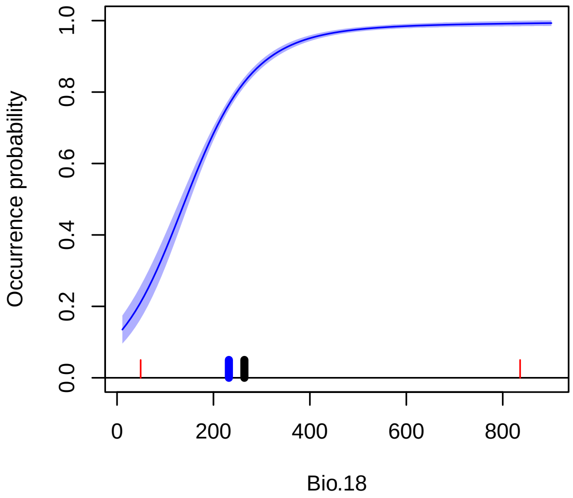

Response maps

Response maps (also referred to as partial effect maps). Each map represents how each predictor affects the occurrence probability. Predictor acronyms: Bio.10 = Mean temperature of warmest quarter [°C] within months 6 to 8, Bio.11 = Mean temperature of coldest quarter [°C] within months 12,1,2, Bio.12 = Annual precipitation sum [mm/m2], Bio.18 = Mean monthly precipitation amount of the warmest quarter [mm/m2] within months 6 to 8.

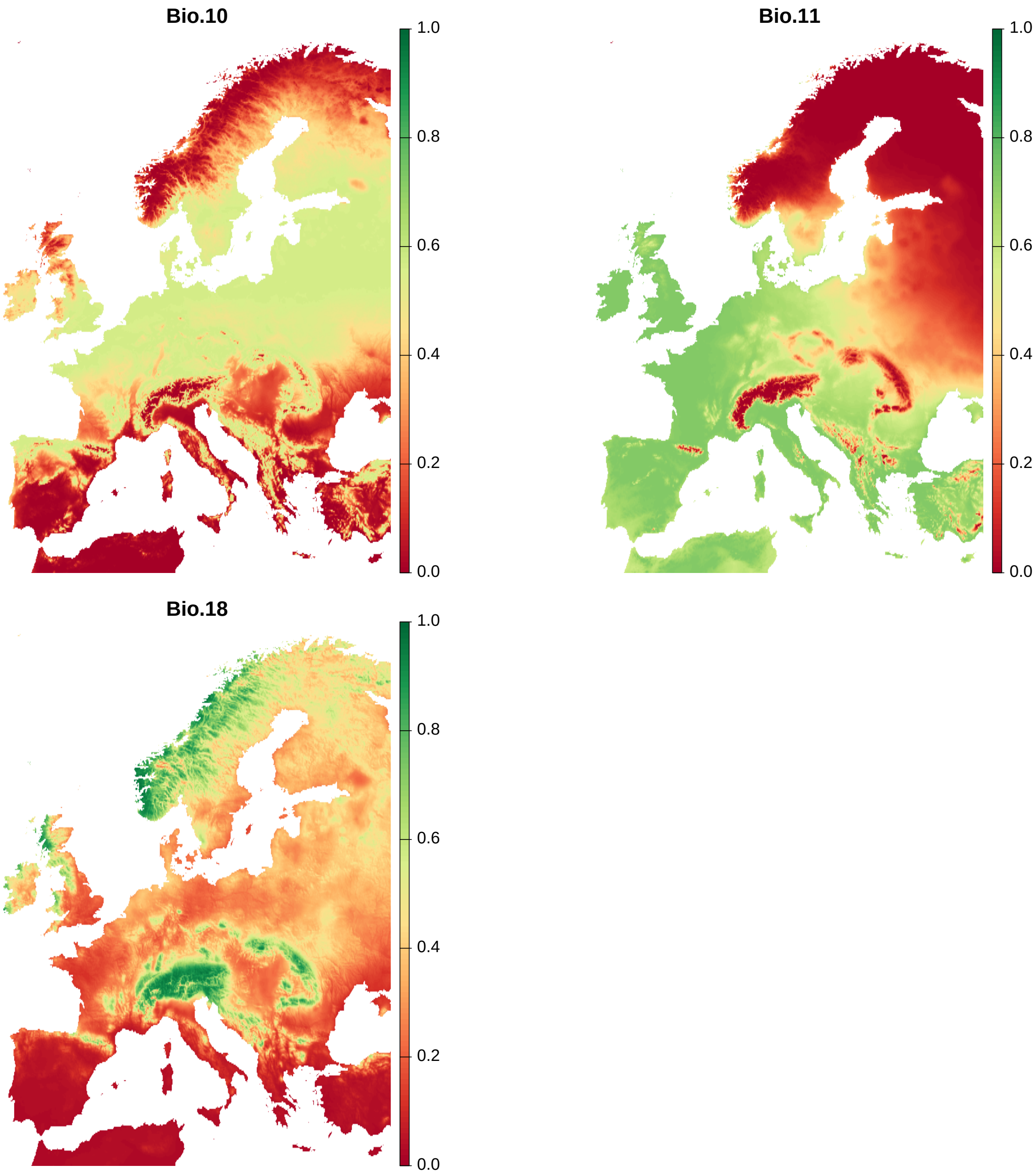

Model projections

Projection with plotted input data

Projection of species distribution model for reference period 1981-2010 over Europe. Occurrence probability ranges from 0 to 1 and is represented in dark red (low probability) to dark green (high probability). Input data used to calibrate the model is shown as presence points in magenta and absence points in black.

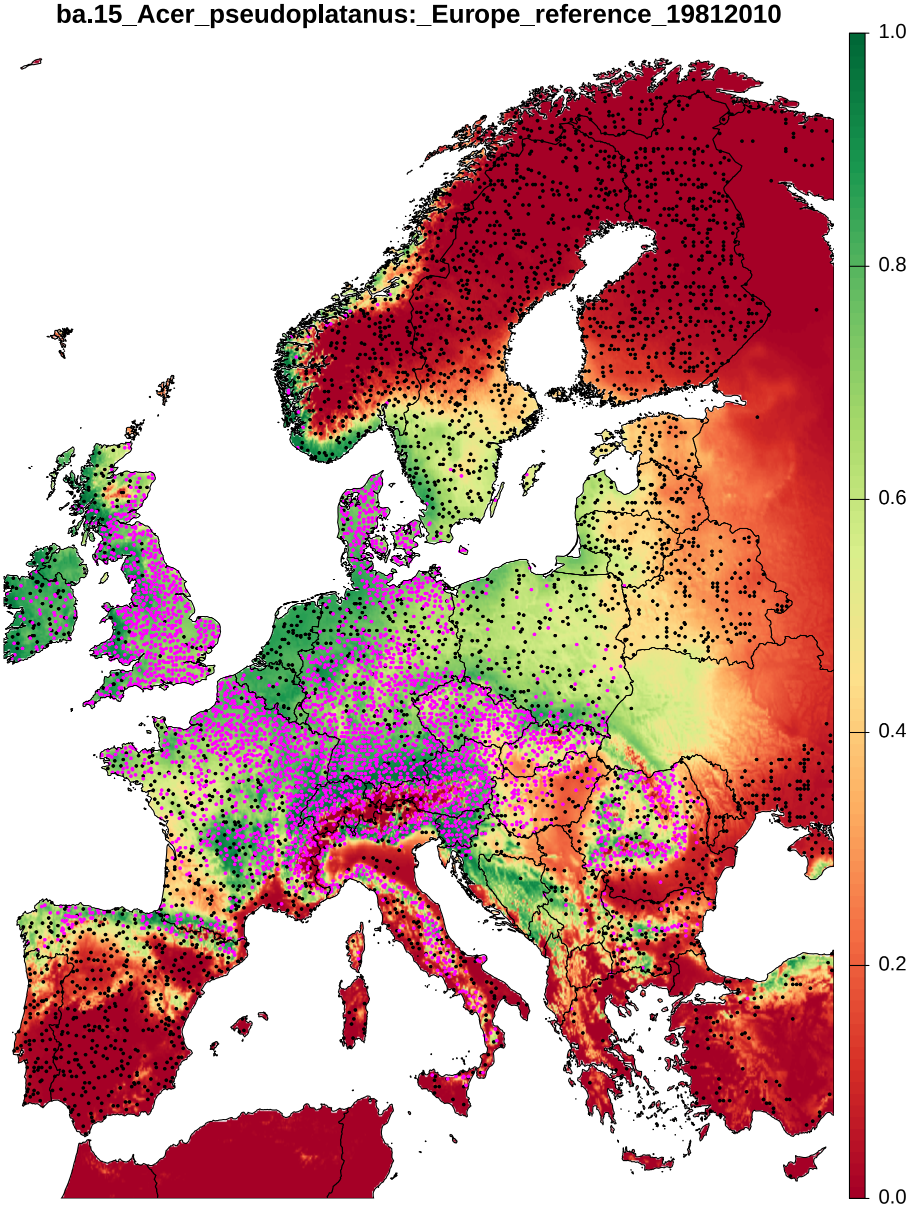

Projections

Projections of the species distribution models for reference period (1981-2010) and future scenarios RCP4.5 (2071-2100) and RCP8.5 (2071-2100) over Europe. Occurrence probabilities range from 0 to 1 and are represented from dark red (low probability) to dark green (high probability).

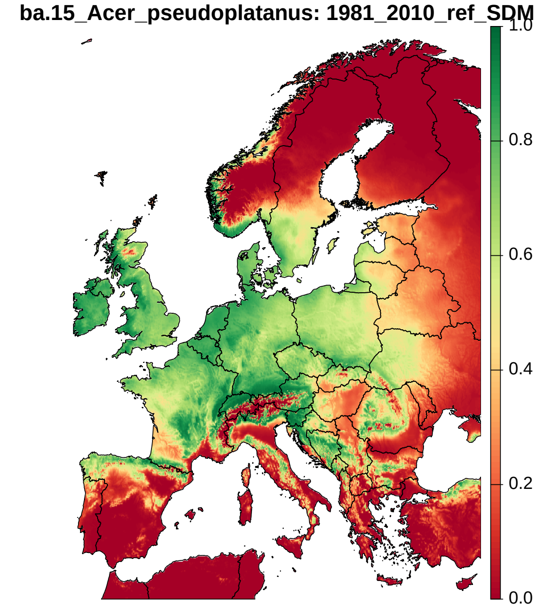

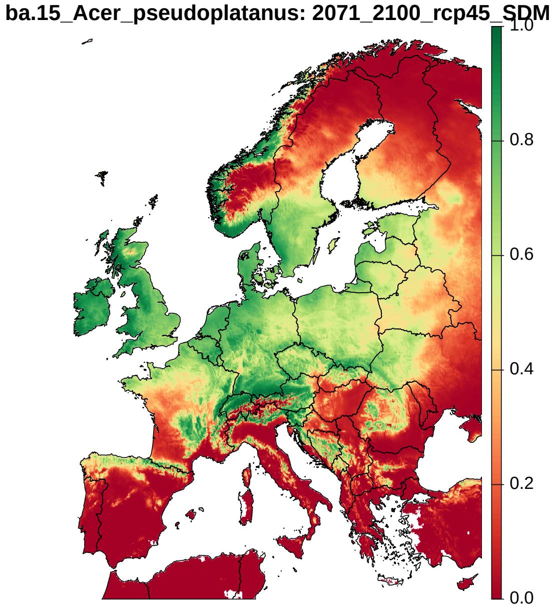

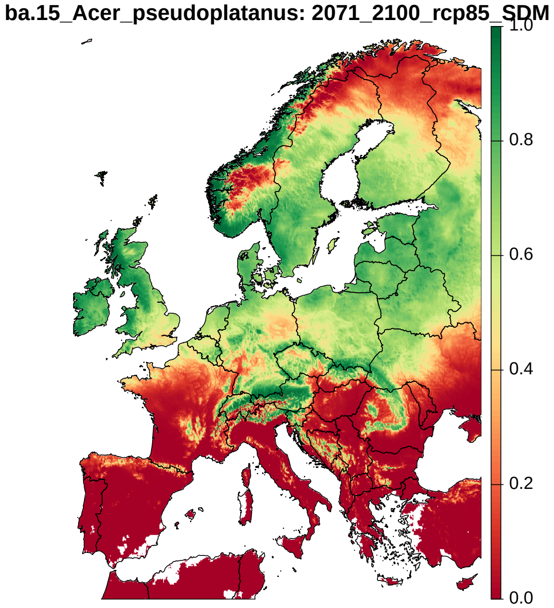

Climate risk classes

Maps of the climate risk classes. To estimate the distribution potential of each species as a mask for the SIMs, the continuous SDM outputs were categorized into three classes: low (yellow), medium (blue) and high climatic risk (red). The maps depict the risk classes in reference time (1981 to 2010), in climate scenario RCP4.5 (2071-2100) and RCP8.5 (2071-2100). To get an impression how well the thresholds fit to the data, presences (black) and absences (grey) were added on the reference map (top left). Refer to the legend and section “SDM thresholds” for the thresholds.

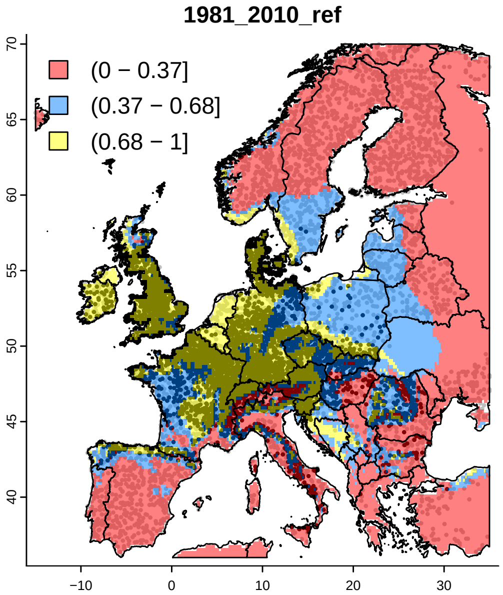

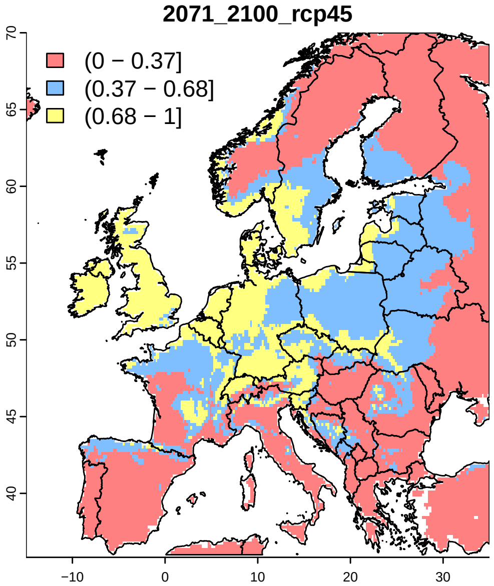

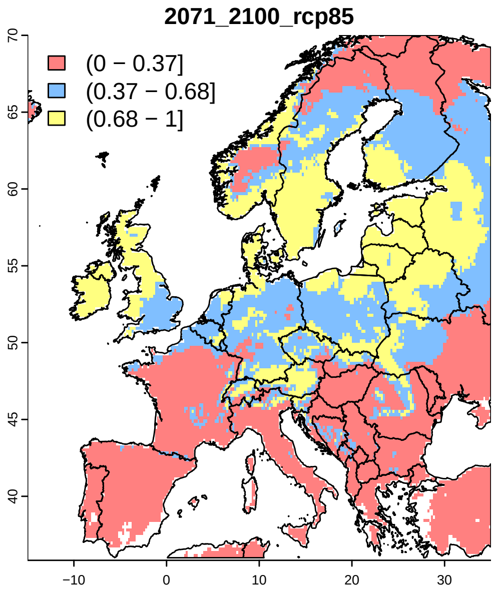

Alnus glutinosa

Model statistics and evaluation

Summary

Predictor acronyms: Bio.10 = Mean temperature of warmest quarter [°C] within months 6 to 8, Bio.11 = Mean temperature of coldest quarter [°C] within months 12,1,2, Bio.12 = Annual precipitation sum [mm/m2], Bio.18 = Mean monthly precipitation amount of the warmest quarter [mm/m2] within months 6 to 8.

```
##
## Family: binomial
## Link function: logit
##
## Formula:
## ba.24 ~ s(Bio.10, k = 3) + s(Bio.11, k = 3) + s(Bio.18, k = 3)
##
## Parametric coefficients:
##             Estimate Std. Error z value Pr(>|z|)
## (Intercept) -0.47552    0.03236  -14.69   <2e-16 ***
## ---
## Signif. codes:  0 '***' 0.001 '**' 0.01 '*' 0.05 '.' 0.1 ' ' 1
##
## Approximate significance of smooth terms:
##             edf Ref.df Chi.sq p-value
## s(Bio.10) 1.999      2  788.6  <2e-16 ***
## s(Bio.11) 1.995      2  931.3  <2e-16 ***
## s(Bio.18) 1.992      2  194.8  <2e-16 ***
## ---
## Signif. codes:  0 '***' 0.001 '**' 0.01 '*' 0.05 '.' 0.1 ' ' 1
##
## R-sq.(adj) =  0.372   Deviance explained =   31%
## -REML = 5107.2  Scale est. = 1         n = 10636
```

Evaluation parameter

Model performance was assessed using four statistical parameters: the area under the receiver operating characteristic curve (AUC), the true skill statistic (TSS), sensitivity (probability of the model to correctly predict a true presence) and specificity (probability of the model to correctly predict a true absence).

```
##       Species_name  AUC       TSS sensitivity specificity
## tp Alnus glutinosa 0.83 0.5584806   0.8772095   0.6812712
```

Response curves and response maps

Response curves

Response curves (also known as effect curves) give an overview of the climatic niche of a species by relating the occurrence probability to corresponding climatic values. Predictor acronyms: Bio.10 = Mean temperature of warmest quarter [°C] within months 6 to 8, Bio.11 = Mean temperature of coldest quarter [°C] within months 12,1,2, Bio.12 = Annual precipitation sum [mm/m2], Bio.18 = Mean monthly precipitation amount of the warmest quarter [mm/m2] within months 6 to 8. Lines on the x-axis mark the upper and lower limit of the used presences (red), the mean (bold black) and the median (bold blue).

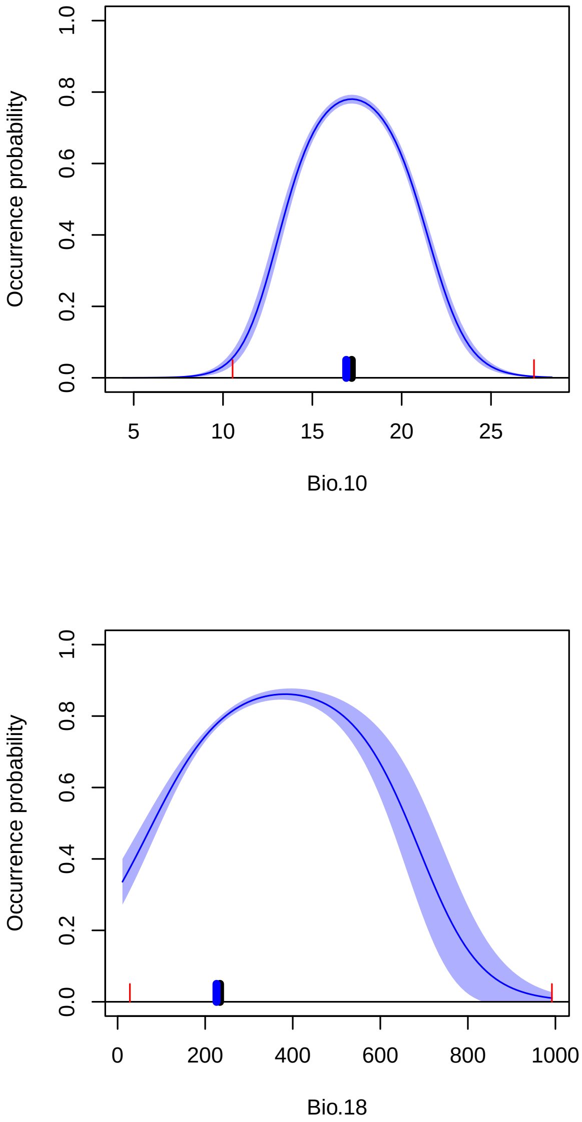

Response maps

Response maps (also referred to as partial effect maps). Each map represents how each predictor affects the occurrence probability. Predictor acronyms: Bio.10 = Mean temperature of warmest quarter [°C] within months 6 to 8, Bio.11 = Mean temperature of coldest quarter [°C] within months 12,1,2, Bio.12 = Annual precipitation sum [mm/m2], Bio.18 = Mean monthly precipitation amount of the warmest quarter [mm/m2] within months 6 to 8.

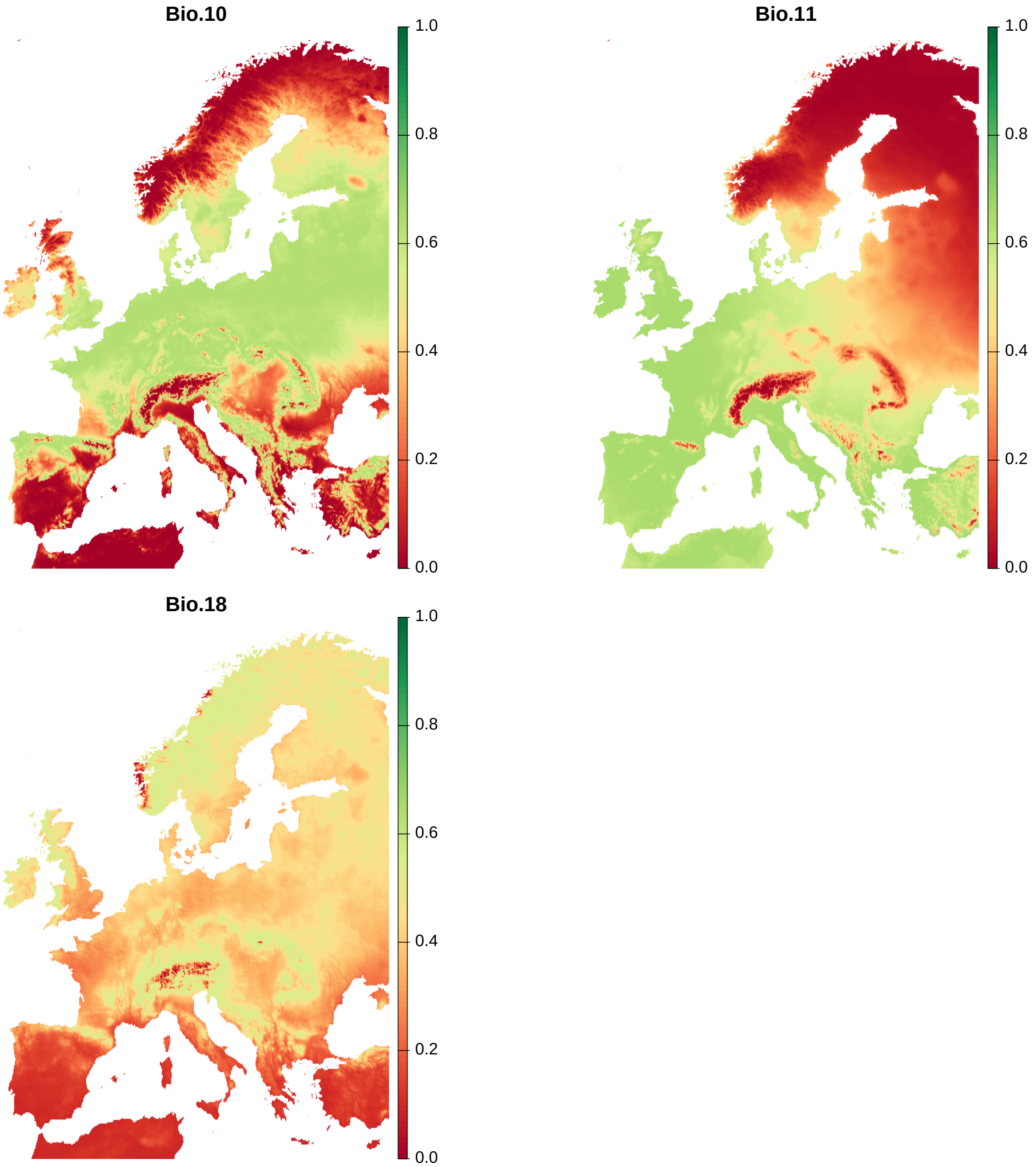

Model projections

Projection with plotted input data

Projection of species distribution model for reference period 1981-2010 over Europe. Occurrence probability ranges from 0 to 1 and is represented in dark red (low probability) to dark green (high probability). Input data used to calibrate the model is shown as presence points in magenta and absence points in black.

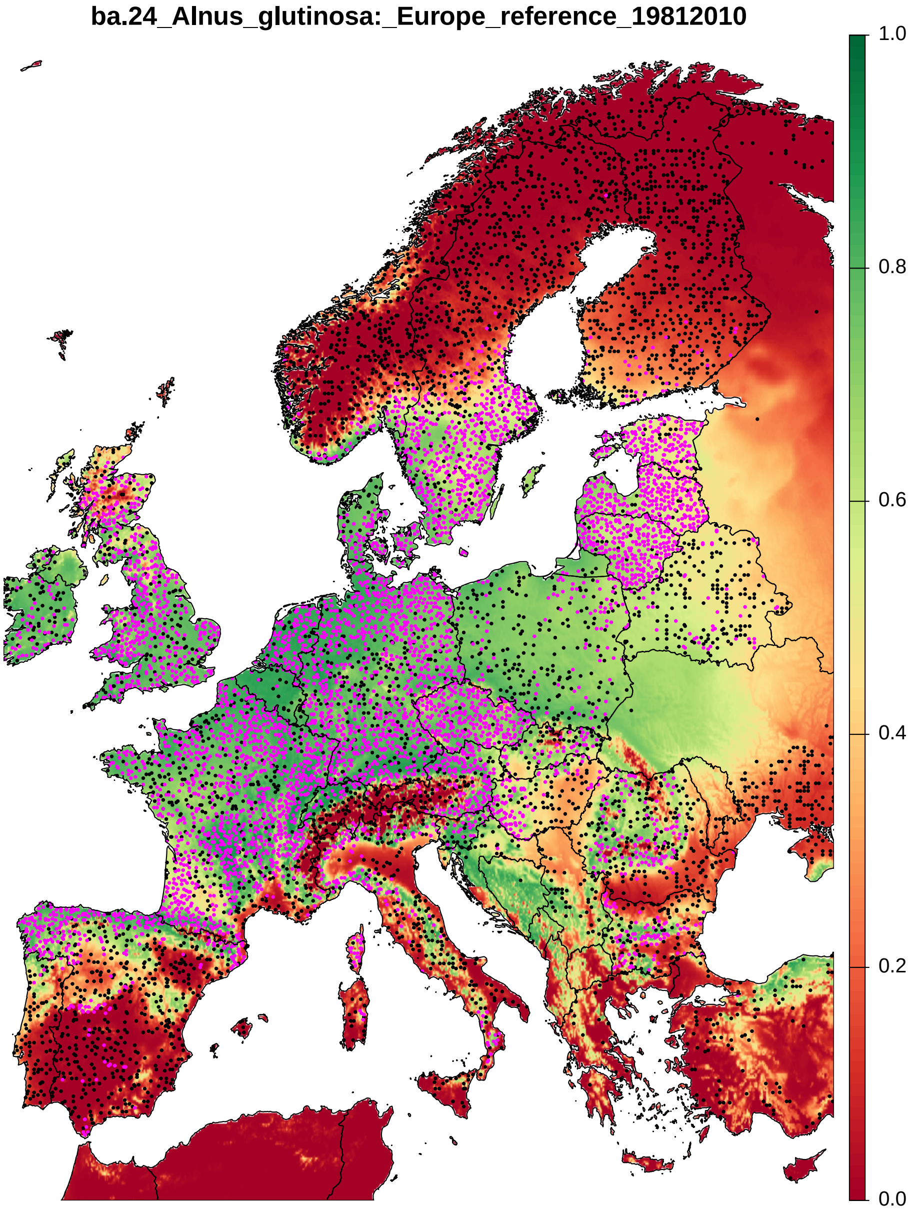

Projections

Projections of the species distribution models for reference period (1981-2010) and future scenarios RCP4.5 (2071-2100) and RCP8.5 (2071-2100) over Europe. Occurrence probabilities range from 0 to 1 and are represented from dark red (low probability) to dark green (high probability).

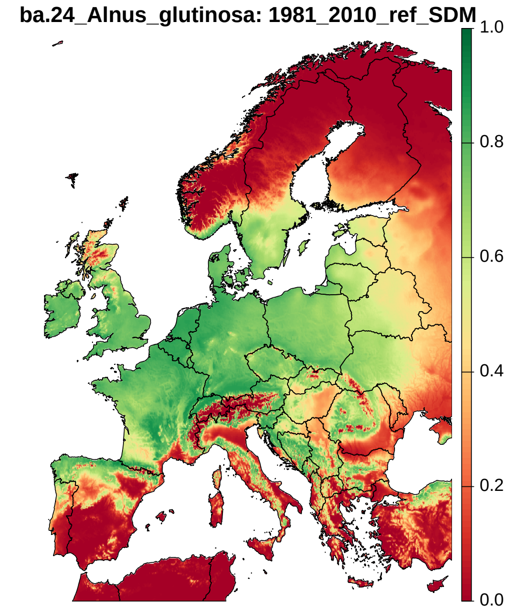

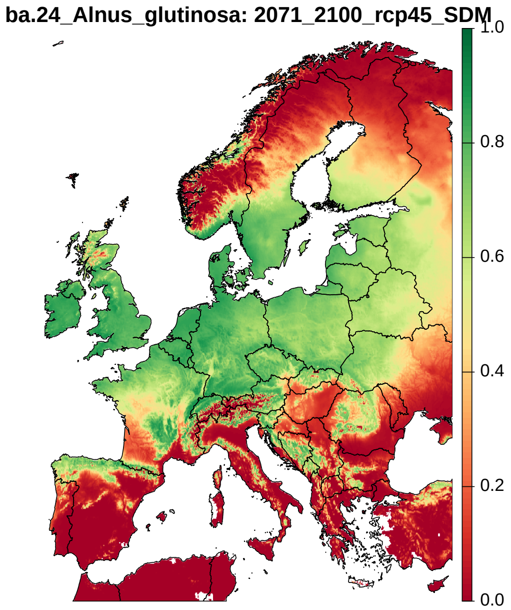

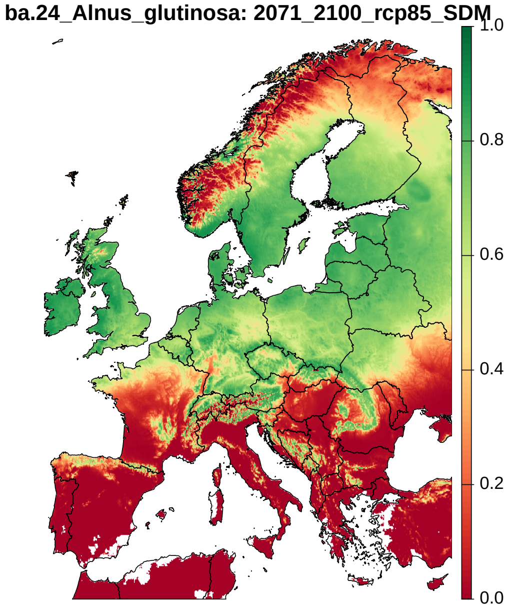

Climate risk classes

Maps of the climate risk classes. To estimate the distribution potential of each species as a mask for the SIMs, the continuous SDM outputs were categorized into three classes: low (yellow), medium (blue) and high climatic risk (red). The maps depict the risk classes in reference time (1981 to 2010), in climate scenario RCP4.5 (2071-2100) and RCP8.5 (2071-2100). To get an impression how well the thresholds fit to the data, presences (black) and absences (grey) were added on the reference map (top left). Refer to the legend and section "SDM thresholds" for the thresholds.

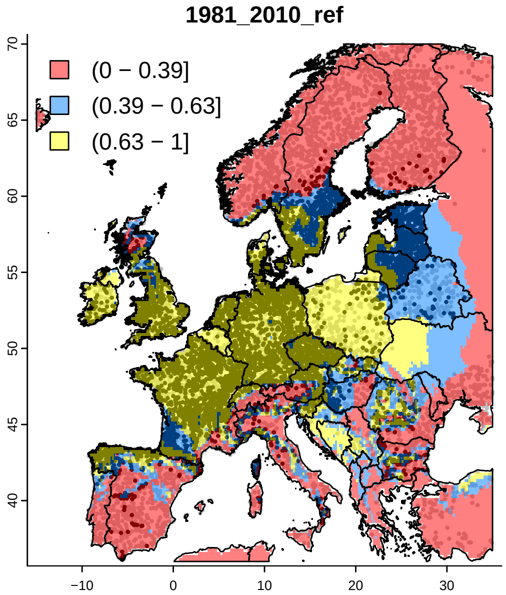

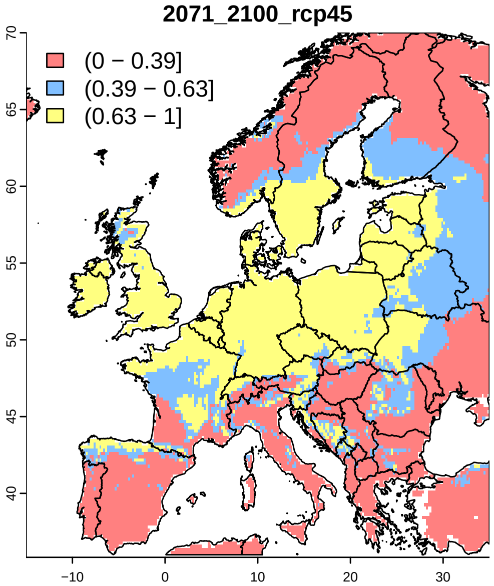

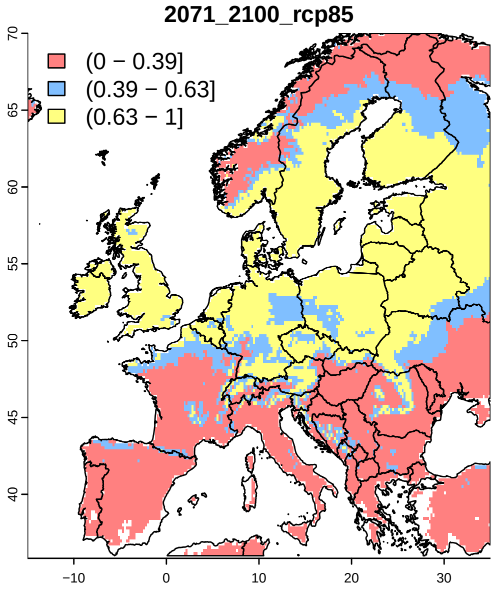

Betula pendula

Model statistics and evaluation

Summary

Predictor acronyms: Bio.10 = Mean temperature of warmest quarter [°C] within months 6 to 8, Bio.11 = Mean temperature of coldest quarter [°C] within months 12,1,2, Bio.12 = Annual precipitation sum [mm/m2], Bio.18 = Mean monthly precipitation amount of the warmest quarter [mm/m2] within months 6 to 8.

```
##
## Family: binomial
## Link function: logit
##
## Formula:
## ba.25 ~ s(Bio.10, k = 3) + s(Bio.11, k = 3) + s(Bio.18, k = 3)
##
## Parametric coefficients:
##             Estimate Std. Error z value Pr(>|z|)
## (Intercept) -0.61082    0.03054     -20   <2e-16 ***
## ---
## Signif. codes:  0 '***' 0.001 '**' 0.01 '*' 0.05 '.' 0.1 ' ' 1
##
## Approximate significance of smooth terms:
##             edf Ref.df  Chi.sq p-value
## s(Bio.10) 1.999  2.000 1762.88  <2e-16 ***
## s(Bio.11) 1.971  1.999   37.24  <2e-16 ***
## s(Bio.18) 1.981  2.000  211.37  <2e-16 ***
## ---
## Signif. codes:  0 '***' 0.001 '**' 0.01 '*' 0.05 '.' 0.1 ' ' 1
##
## R-sq.(adj) =  0.383   Deviance explained = 31.9%
## -REML = 6348.2  Scale est. = 1         n = 13400
```

Evaluation parameter

Model performance was assessed using four statistical parameters: the area under the receiver operating characteristic curve (AUC), the true skill statistic (TSS), sensitivity (probability of the model to correctly predict a true presence) and specificity (probability of the model to correctly predict a true absence).

```
##      Species_name  AUC       TSS sensitivity specificity
## tp Betula pendula 0.84 0.5637313   0.8849254    0.678806
```

Response curves and response maps

Response curves

Response curves (also known as effect curves) give an overview of the climatic niche of a species by relating the occurrence probability to corresponding climatic values. Predictor acronyms: Bio.10 = Mean temperature of warmest quarter [°C] within months 6 to 8, Bio.11 = Mean temperature of coldest quarter [°C] within months 12,1,2, Bio.12 = Annual precipitation sum [mm/m2], Bio.18 = Mean monthly precipitation amount of the warmest quarter [mm/m2] within months 6 to 8. Lines on the x-axis mark the upper and lower limit of the used presences (red), the mean (bold black) and the median (bold blue).

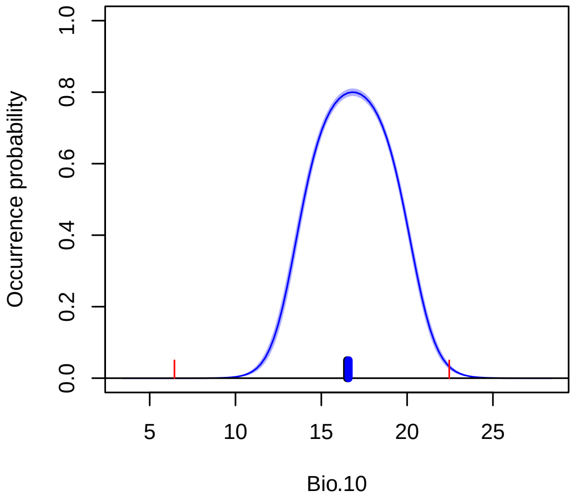

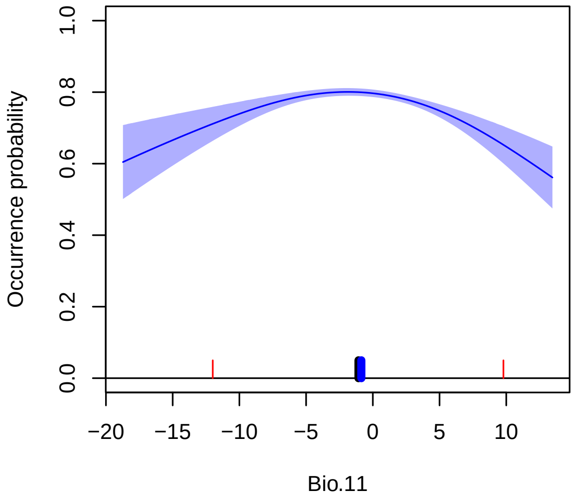

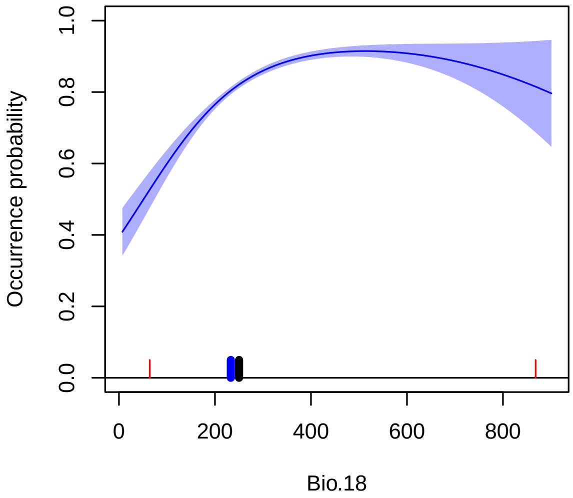

Response maps

Response maps (also referred to as partial effect maps). Each map represents how each predictor affects the occurrence probability. Predictor acronyms: Bio.10 = Mean temperature of warmest quarter [°C] within months 6 to 8, Bio.11 = Mean temperature of coldest quarter [°C] within months 12,1,2, Bio.12 = Annual precipitation sum [mm/m2], Bio.18 = Mean monthly precipitation amount of the warmest quarter [mm/m2] within months 6 to 8.

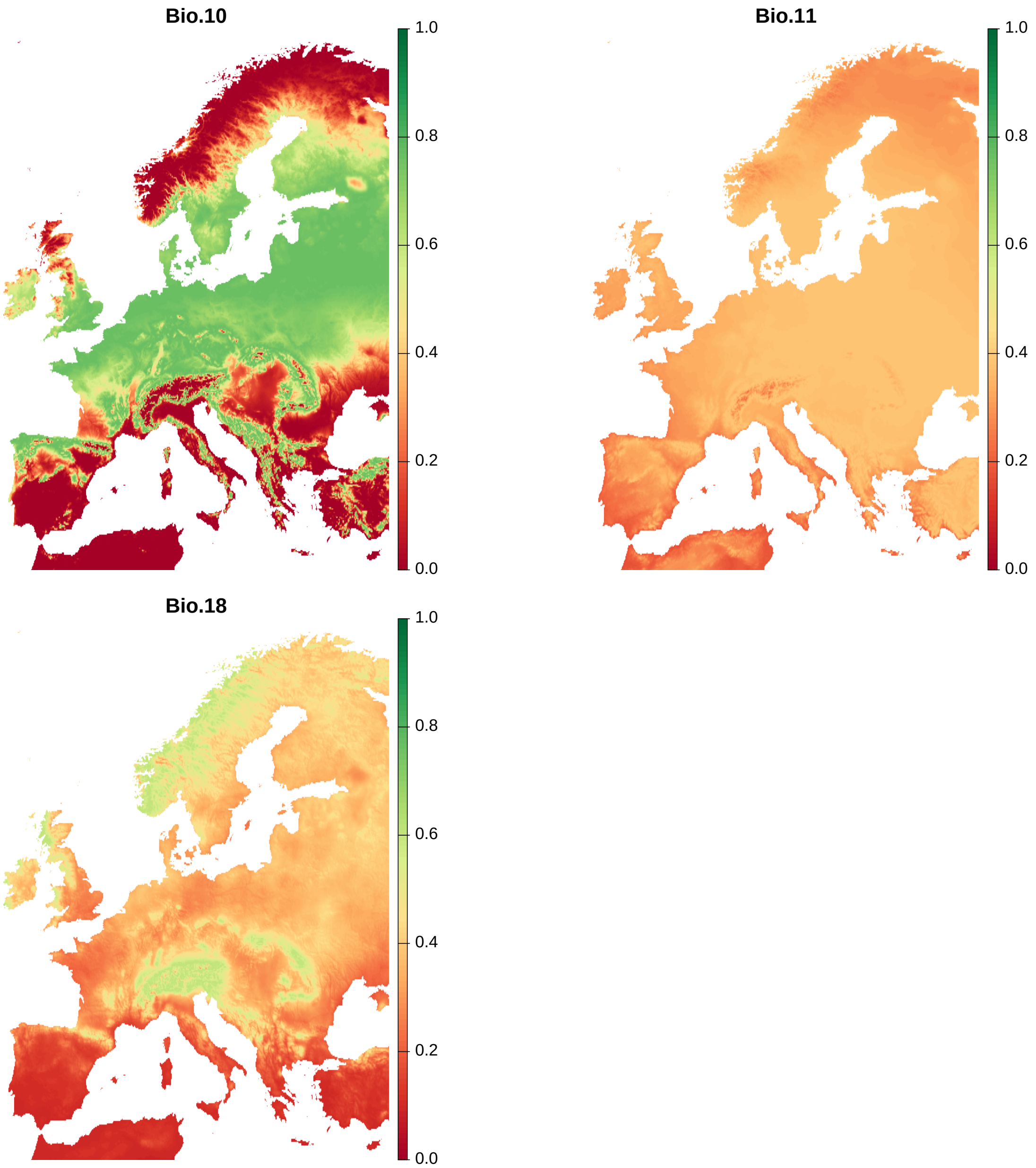

Model projections

Projection with plotted input data

Projection of species distribution model for reference period 1981-2010 over Europe. Occurrence probability ranges from 0 to 1 and is represented in dark red (low probability) to dark green (high probability). Input data used to calibrate the model is shown as presence points in magenta and absence points in black.

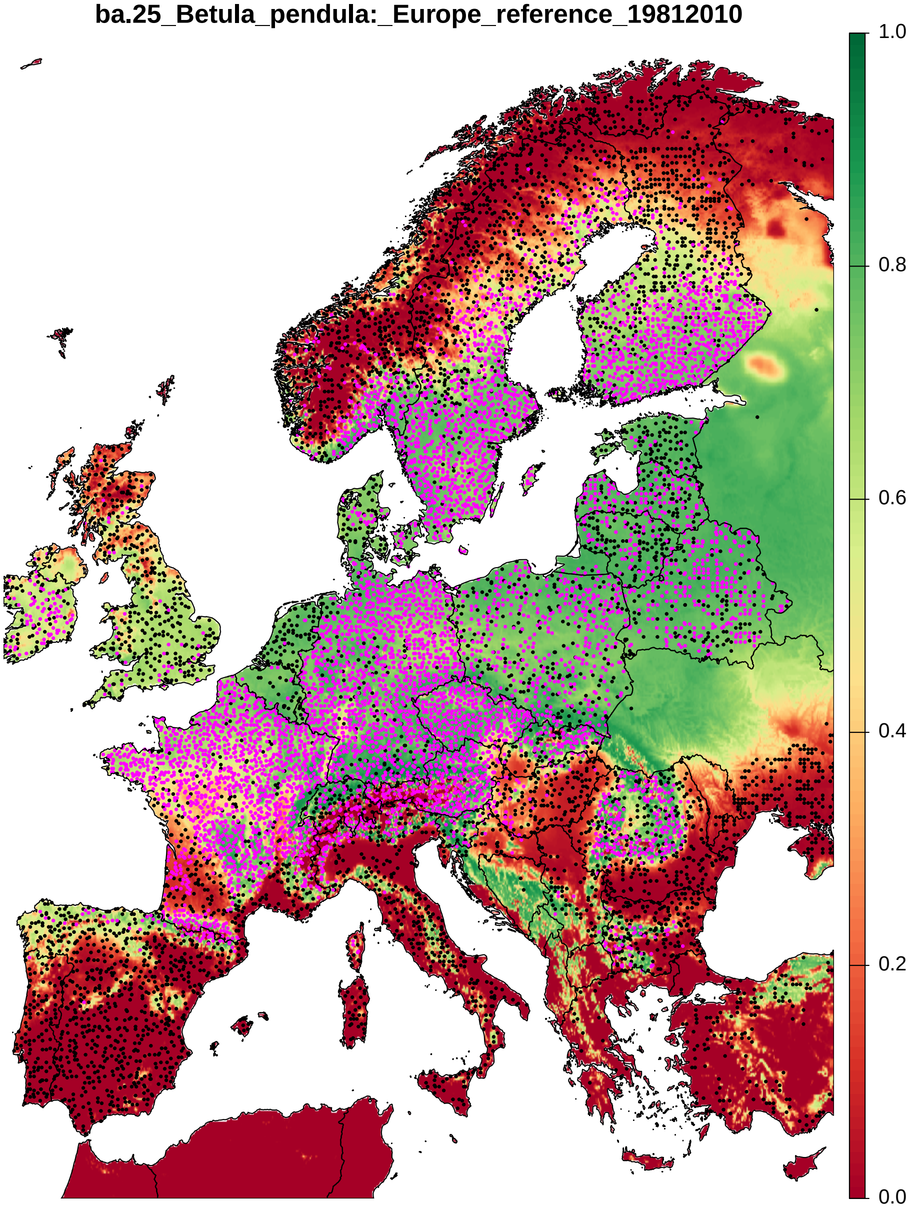

Projections

Projections of the species distribution models for reference period (1981-2010) and future scenarios RCP4.5 (2071-2100) and RCP8.5 (2071-2100) over Europe. Occurrence probabilities range from 0 to 1 and are represented from dark red (low probability) to dark green (high probability).

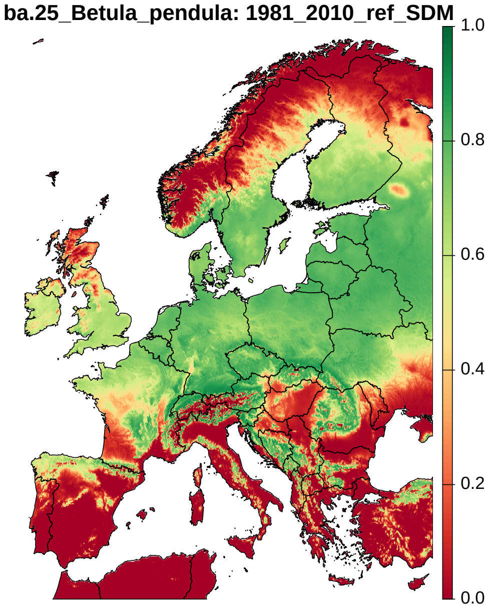

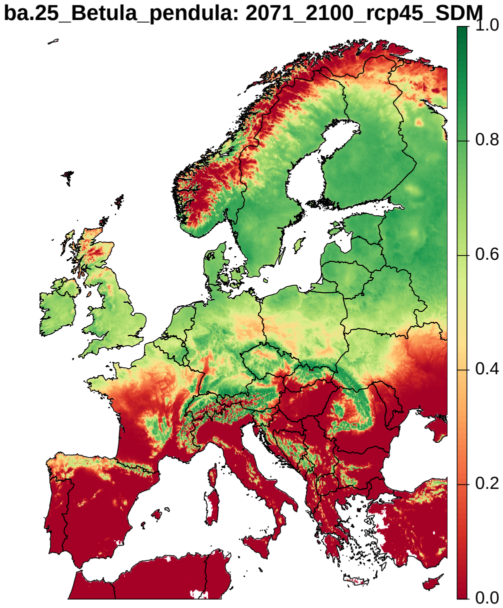

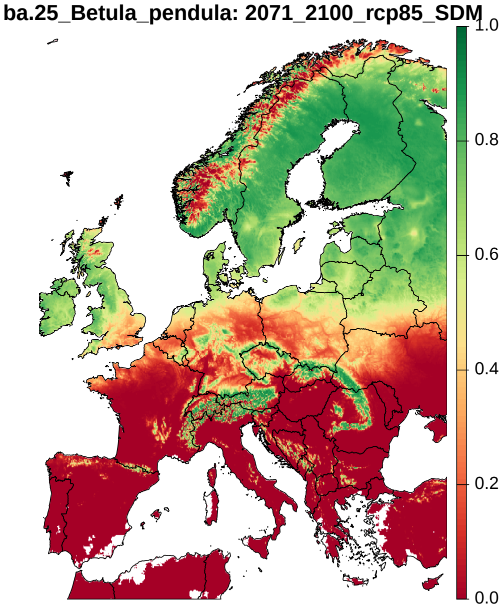

Climate risk classes

Maps of the climate risk classes. To estimate the distribution potential of each species as a mask for the SIMs, the continuous SDM outputs were categorized into three classes: low (yellow), medium (blue) and high climatic risk (red). The maps depict the risk classes in reference time (1981 to 2010), in climate scenario RCP4.5 (2071-2100) and RCP8.5 (2071-2100). To get an impression how well the thresholds fit to the data, presences (black) and absences (grey) were added on the reference map (top left). Refer to the legend and section "SDM thresholds" for the thresholds.

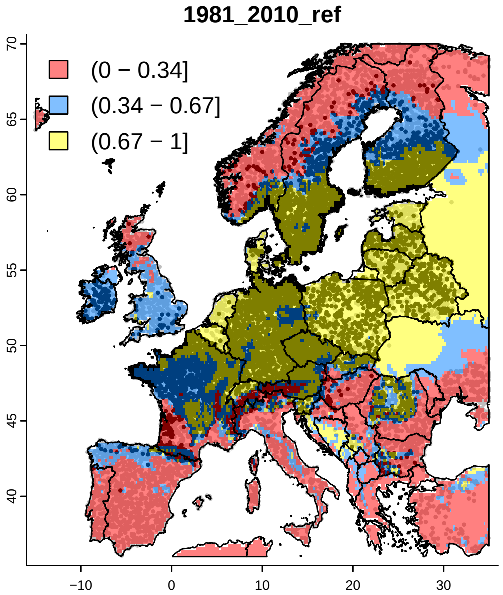

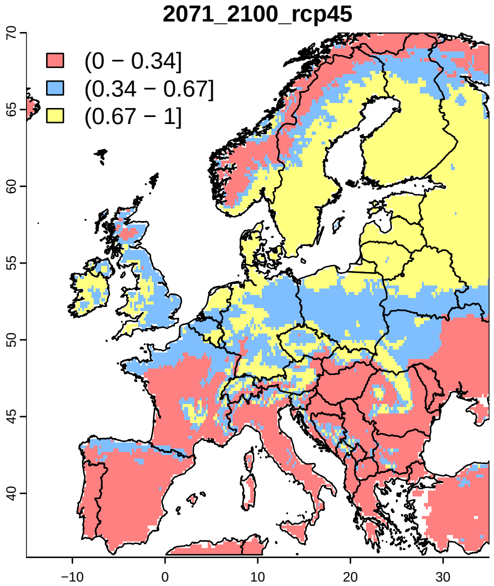

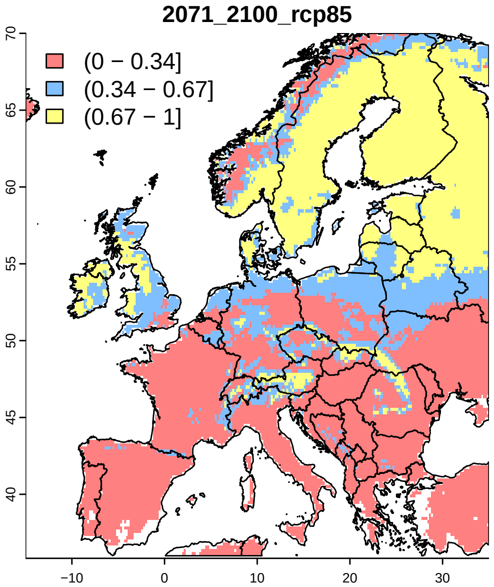

Carpinus betulus

Model statistics and evaluation

Summary

Predictor acronyms: Bio.10 = Mean temperature of warmest quarter [°C] within months 6 to 8, Bio.11 = Mean temperature of coldest quarter [°C] within months 12,1,2, Bio.12 = Annual precipitation sum [mm/m2], Bio.18 = Mean monthly precipitation amount of the warmest quarter [mm/m2] within months 6 to 8.

```
##
## Family: binomial
## Link function: logit
##
## Formula:
## ba.23 ~ s(Bio.10, k = 3) + s(Bio.11, k = 3) + s(Bio.18, k = 3)
##
## Parametric coefficients:
##             Estimate Std. Error z value Pr(>|z|)
## (Intercept) -2.14078    0.08176  -26.18   <2e-16 ***
## ---
## Signif. codes:  0 ’***’ 0.001 ’**’ 0.01 ’*’ 0.05 ’.’ 0.1 ’ ’ 1
##
## Approximate significance of smooth terms:
##             edf Ref.df Chi.sq p-value
## s(Bio.10) 1.999      2  853.9  <2e-16 ***
## s(Bio.11) 1.998      2  692.1  <2e-16 ***
## s(Bio.18) 1.993      2  484.6  <2e-16 ***
## ---
## Signif. codes:  0 ’***’ 0.001 ’**’ 0.01 ’*’ 0.05 ’.’ 0.1 ’ ’ 1
##
## R-sq.(adj) =  0.563   Deviance explained = 49.1%
## -REML = 2795.3  Scale est. = 1         n = 7866
```

Evaluation parameter

Model performance was assessed using four statistical parameters: the area under the receiver operating characteristic curve (AUC), the true skill statistic (TSS), sensitivity (probability of the model to correctly predict a true presence) and specificity (probability of the model to correctly predict a true absence).

```
##        Species_name  AUC       TSS sensitivity specificity
## tp Carpinus betulus 0.92 0.6981948   0.8934655   0.8047292
```

Response curves and response maps

Response curves

Response curves (also known as effect curves) give an overview of the climatic niche of a species by relating the occurrence probability to corresponding climatic values. Predictor acronyms: Bio.10 = Mean temperature of warmest quarter [°C] within months 6 to 8, Bio.11 = Mean temperature of coldest quarter [°C] within months 12,1,2, Bio.12 = Annual precipitation sum [mm/m2], Bio.18 = Mean monthly precipitation amount of the warmest quarter [mm/m2] within months 6 to 8. Lines on the x-axis mark the upper and lower limit of the used presences (red), the mean (bold black) and the median (bold blue).

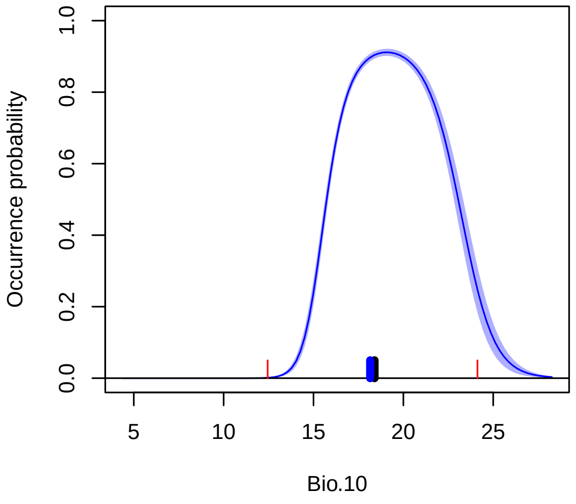

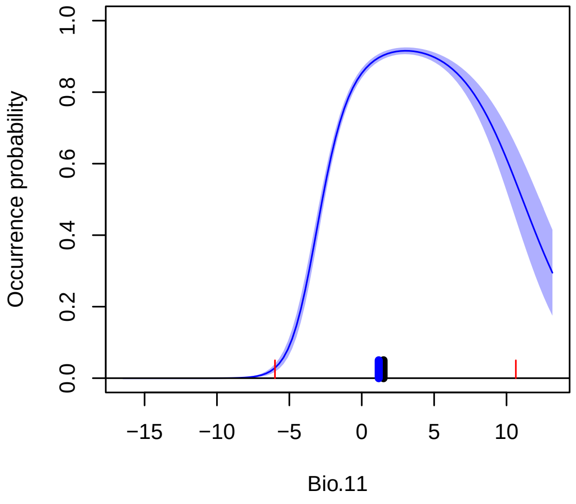

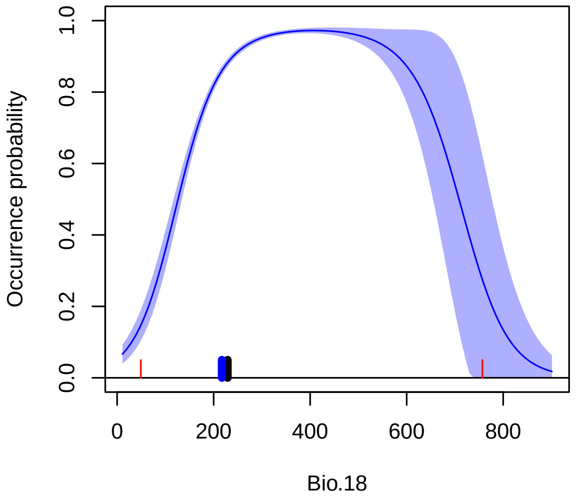

Response maps

Response maps (also referred to as partial effect maps). Each map represents how each predictor affects the occurrence probability. Predictor acronyms: Bio.10 = Mean temperature of warmest quarter [°C] within months 6 to 8, Bio.11 = Mean temperature of coldest quarter [°C] within months 12,1,2, Bio.12 = Annual precipitation sum [mm/m2], Bio.18 = Mean monthly precipitation amount of the warmest quarter [mm/m2] within months 6 to 8.

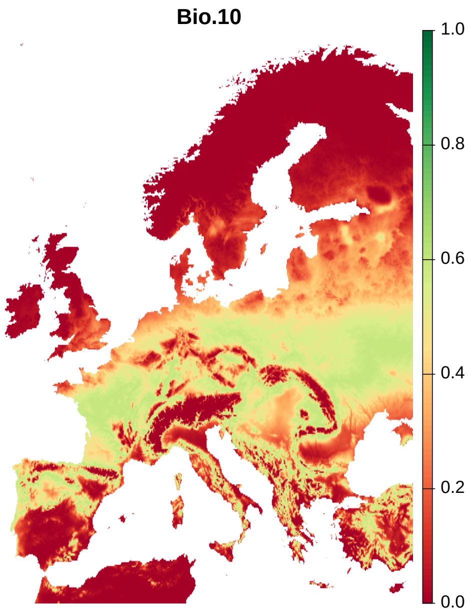

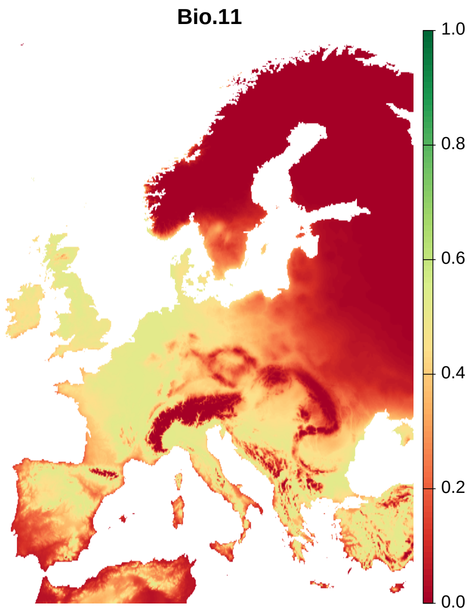

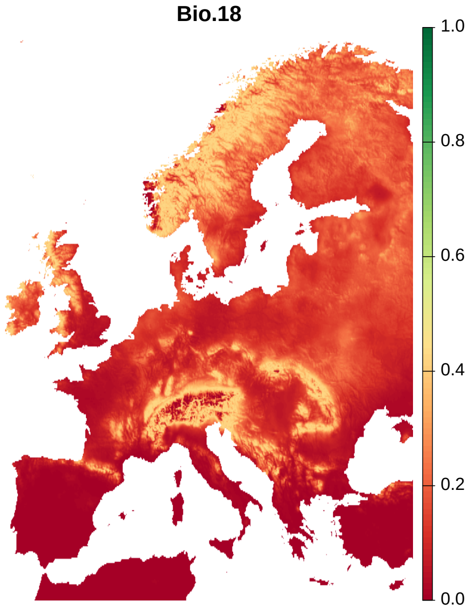

Model projections

Projection with plotted input data

Projection of species distribution model for reference period 1981-2010 over Europe. Occurrence probability ranges from 0 to 1 and is represented in dark red (low probability) to dark green (high probability). Input data used to calibrate the model is shown as presence points in magenta and absence points in black.

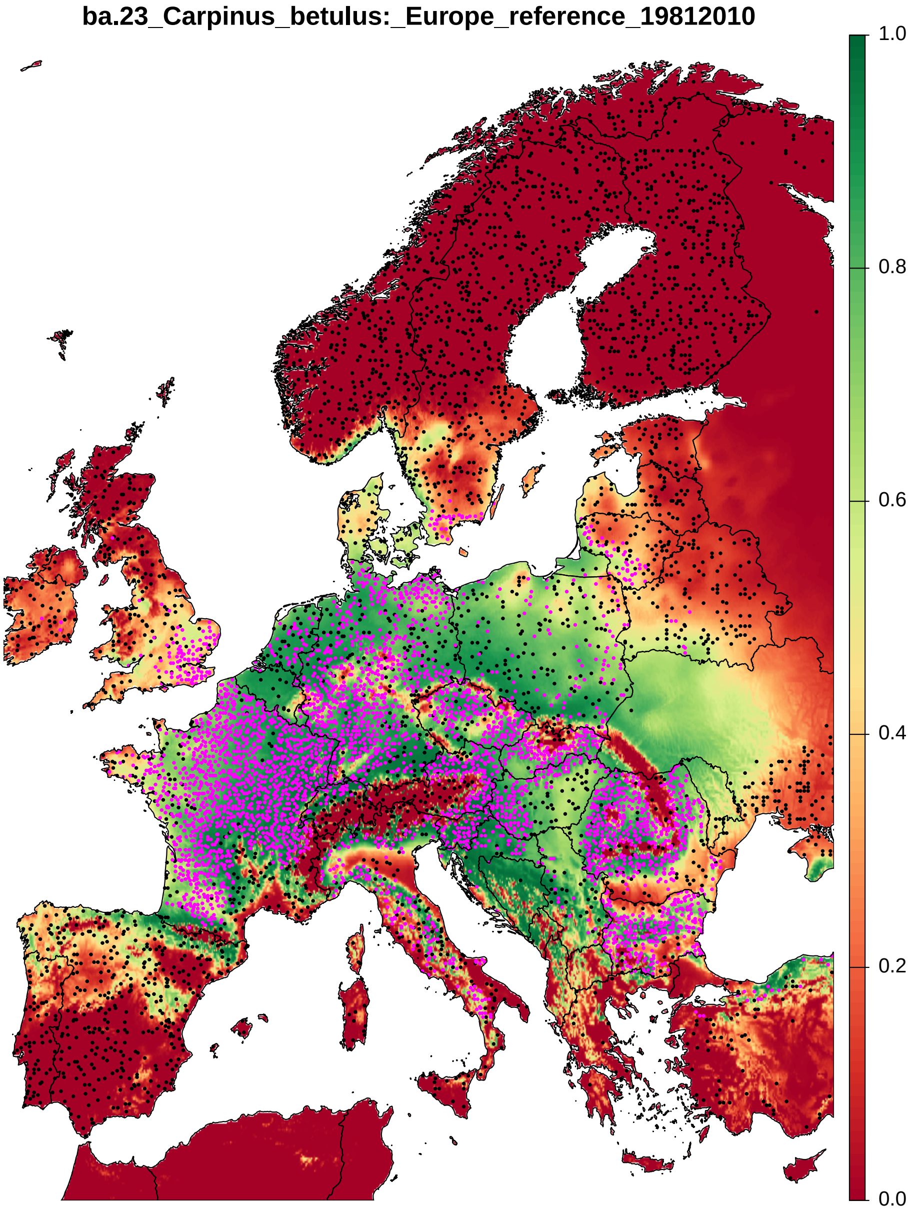

Projections

Projections of the species distribution models for reference period (1981-2010) and future scenarios RCP4.5 (2071-2100) and RCP8.5 (2071-2100) over Europe. Occurrence probabilities range from 0 to 1 and are represented from dark red (low probability) to dark green (high probability).

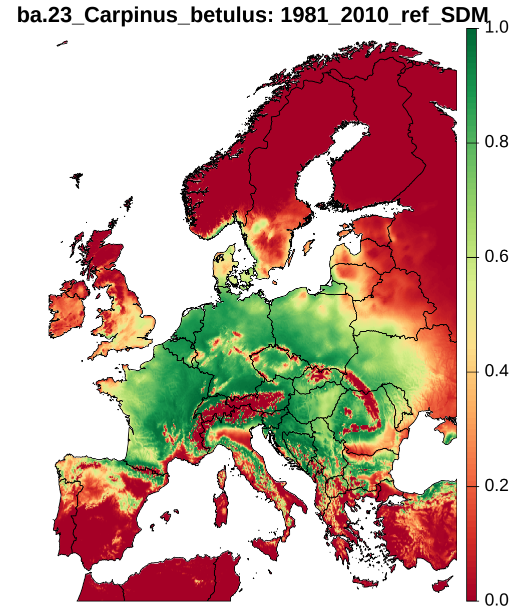

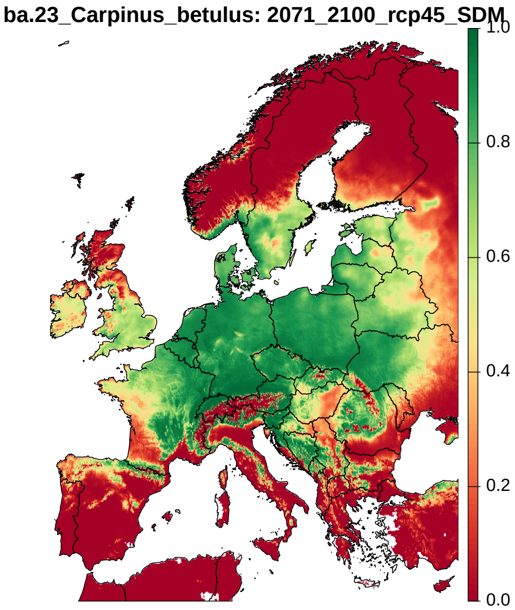

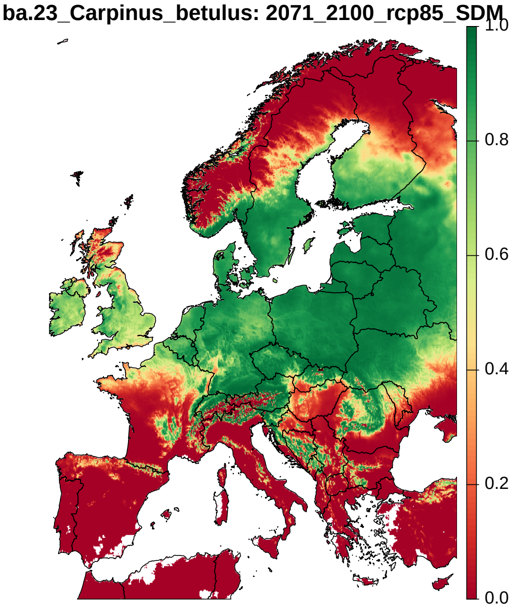

Climate risk classes

Maps of the climate risk classes. To estimate the distribution potential of each species as a mask for the SIMs, the continuous SDM outputs were categorized into three classes: low (yellow), medium (blue) and high climatic risk (red). The maps depict the risk classes in reference time (1981 to 2010), in climate scenario RCP4.5 (2071-2100) and RCP8.5 (2071-2100). To get an impression how well the thresholds fit to the data, presences (black) and absences (grey) were added on the reference map (top left). Refer to the legend and section “SDM thresholds” for the thresholds.

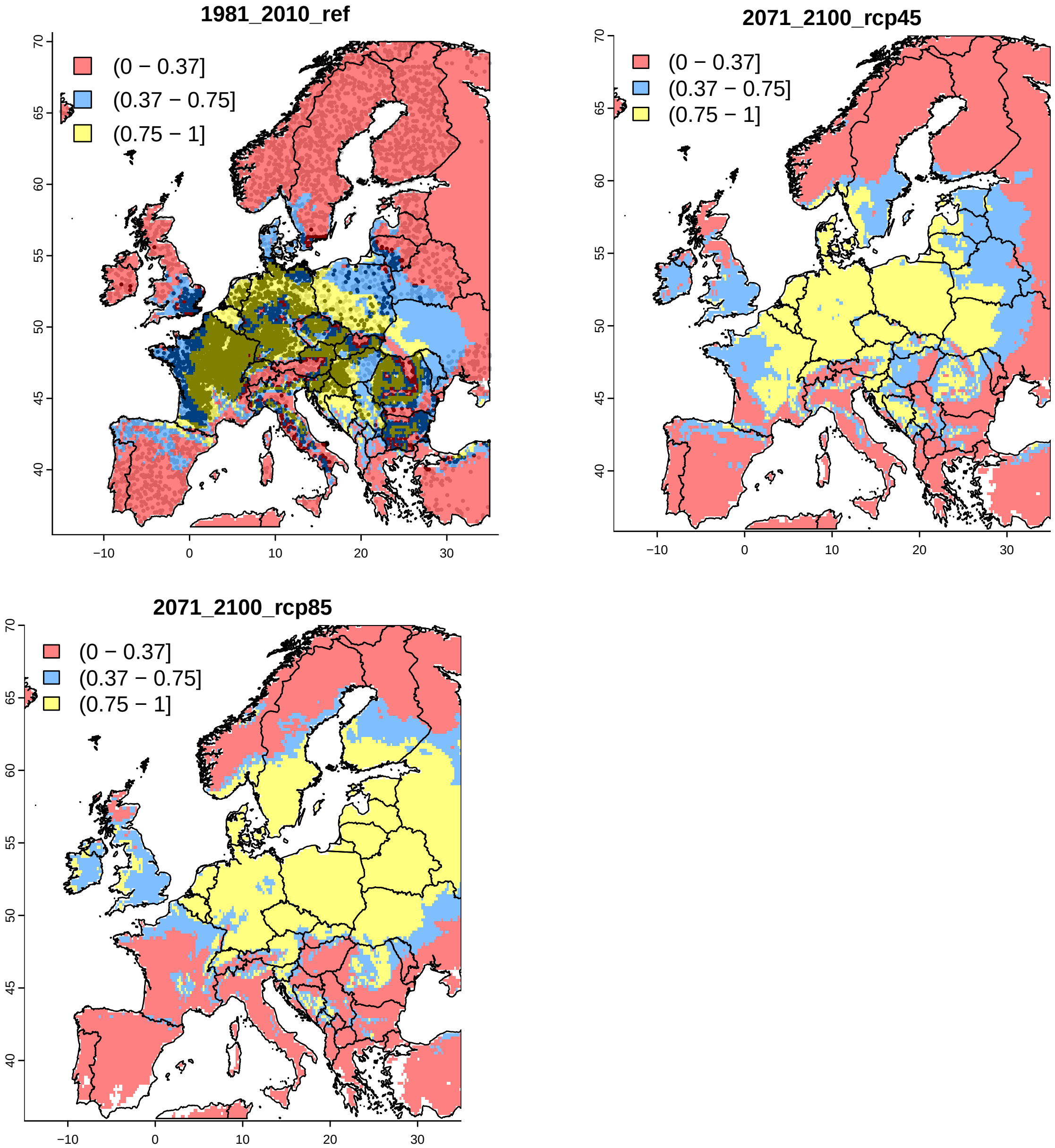

Castanea sativa

Model statistics and evaluation

Summary

Predictor acronyms: Bio.10 = Mean temperature of warmest quarter [°C] within months 6 to 8, Bio.11 = Mean temperature of coldest quarter [°C] within months 12,1,2, Bio.12 = Annual precipitation sum [mm/m2], Bio.18 = Mean monthly precipitation amount of the warmest quarter [mm/m2] within months 6 to 8.

```
##
## Family: binomial
## Link function: logit
##
## Formula:
## ba.31 ~ s(Bio.10, k = 3) + s(Bio.11, k = 3) + s(Bio.12, k = 3)
##
## Parametric coefficients:
##             Estimate Std. Error z value Pr(>|z|)
## (Intercept)  -1.6463     0.1217  -13.53   <2e-16 ***
## ---
## Signif. codes:  0 '***' 0.001 '**' 0.01 '*' 0.05 '.' 0.1 ' ' 1
##
## Approximate significance of smooth terms:
##             edf Ref.df Chi.sq p-value
## s(Bio.10) 1.998   2.00  482.1  <2e-16 ***
## s(Bio.11) 1.900   1.99  567.4  <2e-16 ***
## s(Bio.12) 1.989   2.00  376.5  <2e-16 ***
## ---
## Signif. codes:  0 '***' 0.001 '**' 0.01 '*' 0.05 '.' 0.1 ' ' 1
##
## R-sq.(adj) =  0.642   Deviance explained = 56.9%
## -REML = 1506.7  Scale est. = 1         n = 4990
```

Evaluation parameter

Model performance was assessed using four statistical parameters: the area under the receiver operating characteristic curve (AUC), the true skill statistic (TSS), sensitivity (probability of the model to correctly predict a true presence) and specificity (probability of the model to correctly predict a true absence).

```
##       Species_name  AUC      TSS sensitivity specificity
## tp Castanea sativa 0.94 0.757515    0.907014    0.850501
```

Response curves and response maps

Response curves

Response curves (also known as effect curves) give an overview of the climatic niche of a species by relating the occurrence probability to corresponding climatic values. Predictor acronyms: Bio.10 = Mean temperature of warmest quarter [°C] within months 6 to 8, Bio.11 = Mean temperature of coldest quarter [°C] within months 12,1,2, Bio.12 = Annual precipitation sum [mm/m2], Bio.18 = Mean monthly precipitation amount of the warmest quarter [mm/m2] within months 6 to 8. Lines on the x-axis mark the upper and lower limit of the used presences (red), the mean (bold black) and the median (bold blue).

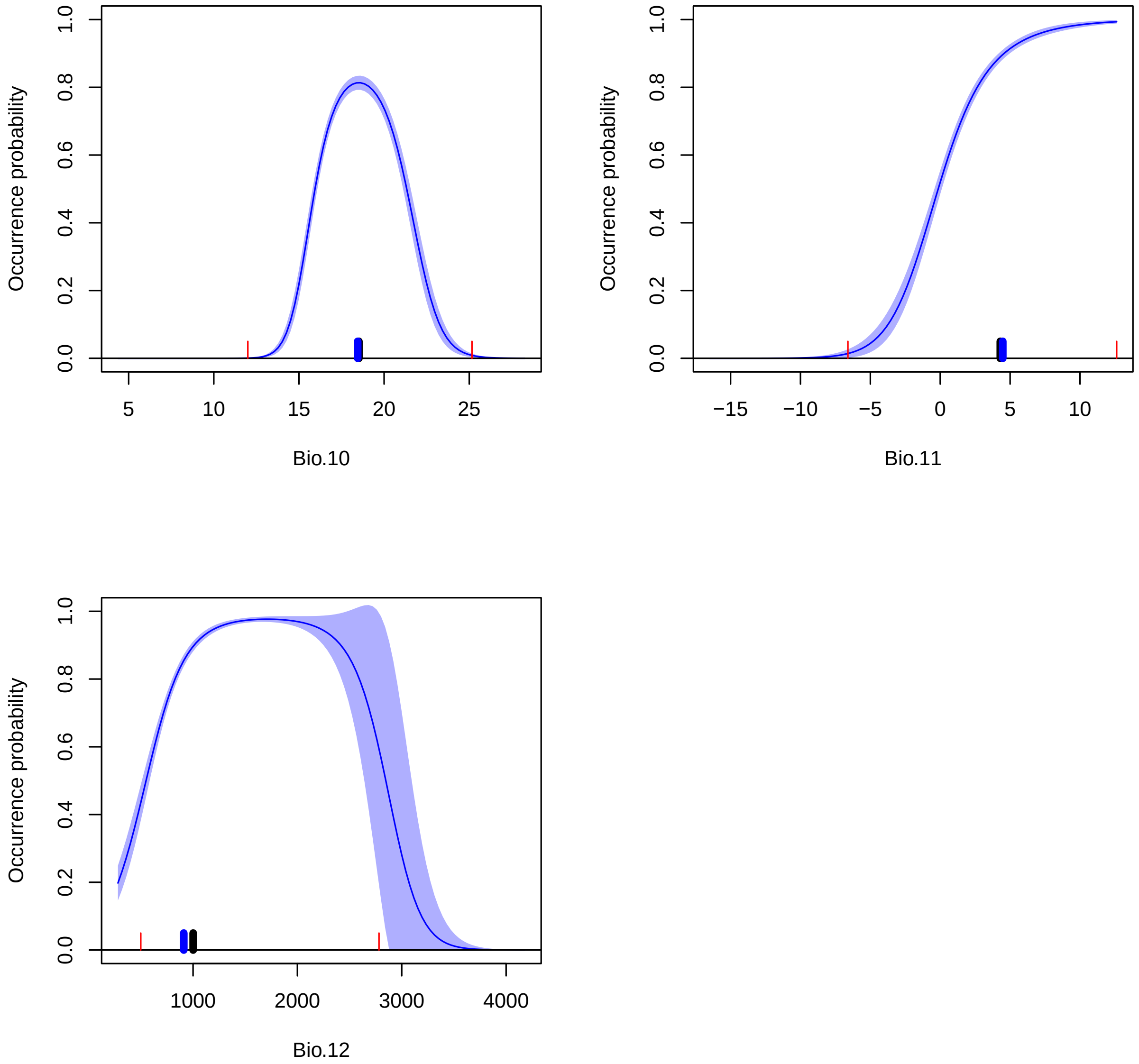

Response maps

Response maps (also referred to as partial effect maps). Each map represents how each predictor affects the occurrence probability. Predictor acronyms: Bio.10 = Mean temperature of warmest quarter [°C] within months 6 to 8, Bio.11 = Mean temperature of coldest quarter [°C] within months 12,1,2, Bio.12 = Annual precipitation sum [mm/m2], Bio.18 = Mean monthly precipitation amount of the warmest quarter [mm/m2] within months 6 to 8.

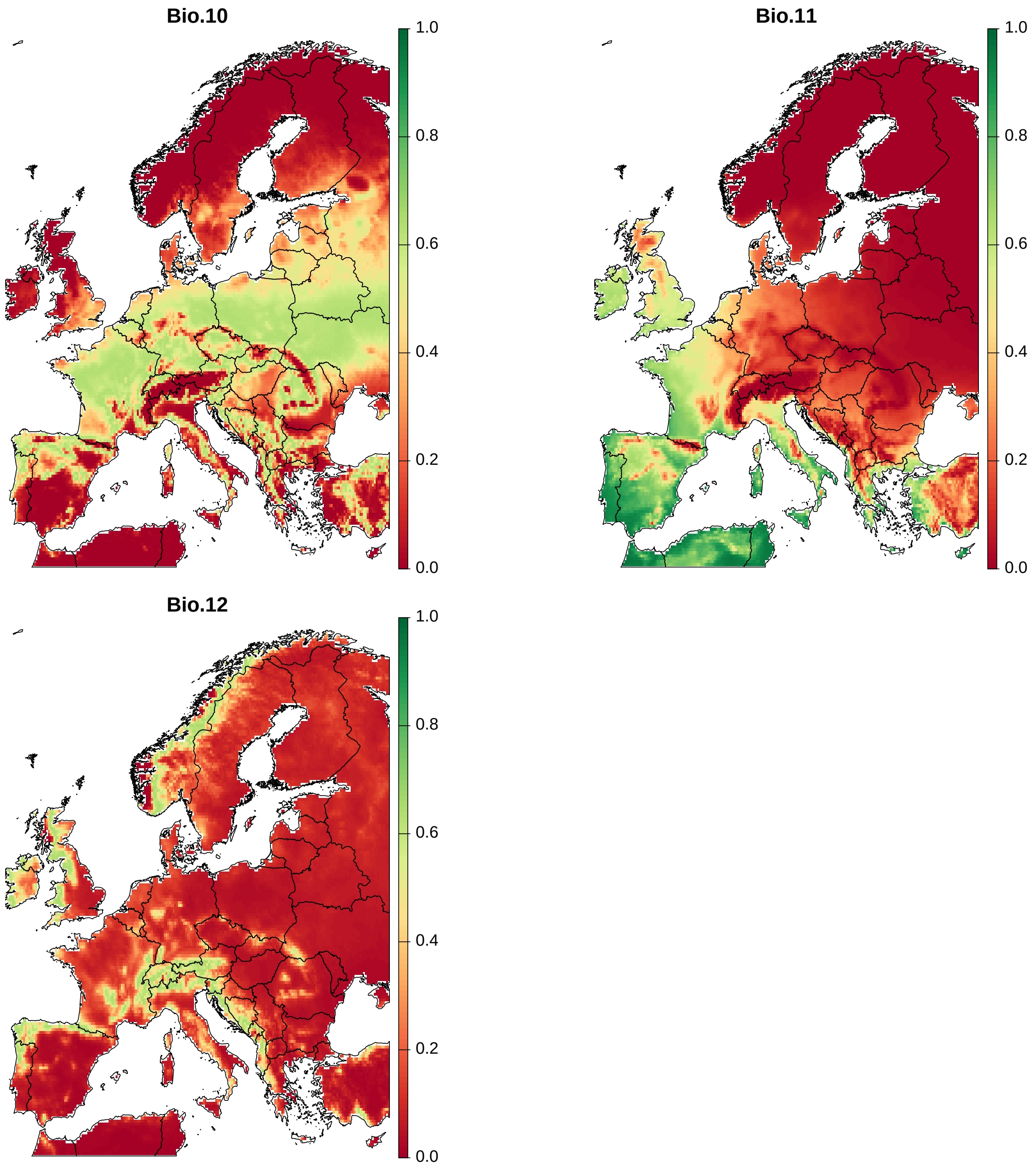

Model projections

Projection with plotted input data

Projection of species distribution model for reference period 1981-2010 over Europe. Occurrence probability ranges from 0 to 1 and is represented in dark red (low probability) to dark green (high probability). Input data used to calibrate the model is shown as presence points in magenta and absence points in black.

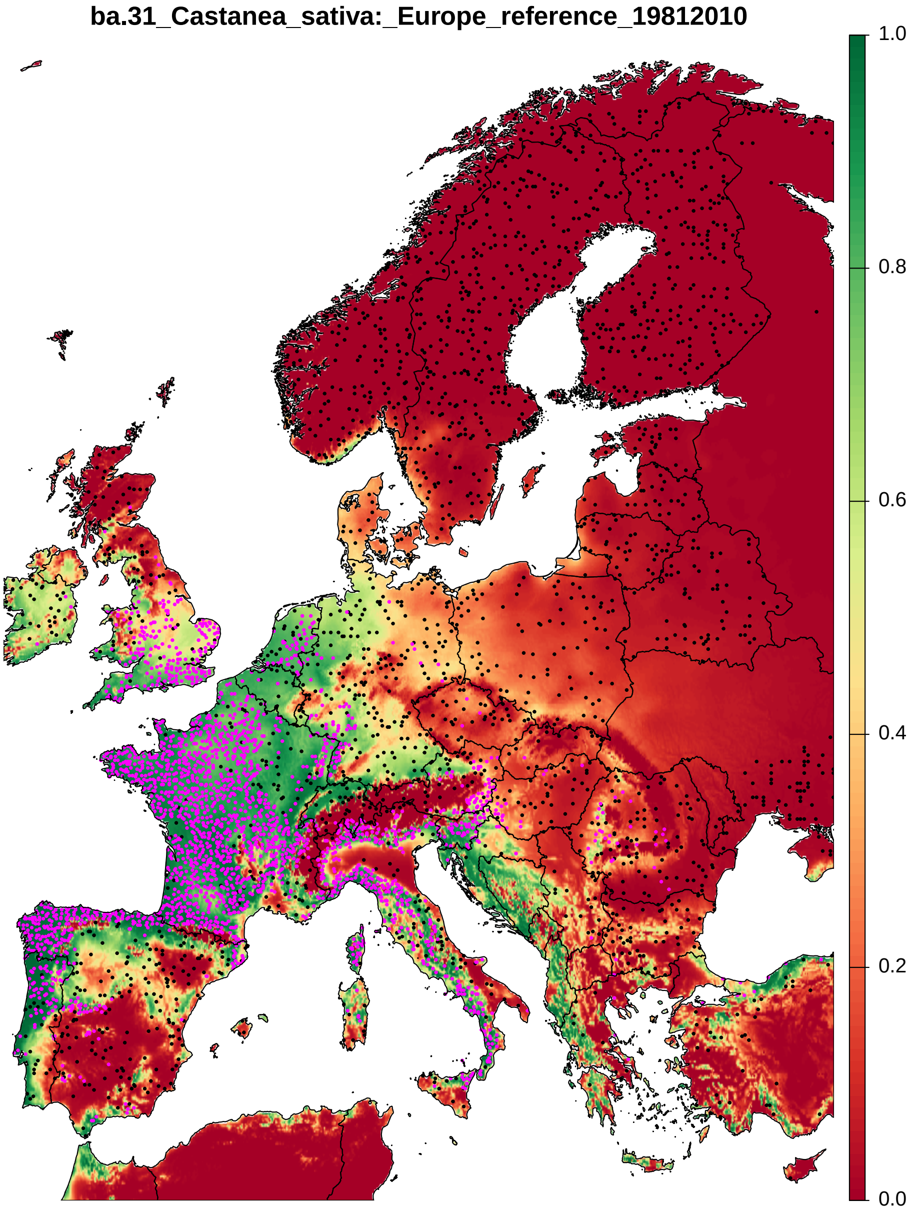

Projections

Projections of the species distribution models for reference period (1981-2010) and future scenarios RCP4.5 (2071-2100) and RCP8.5 (2071-2100) over Europe. Occurrence probabilities range from 0 to 1 and are represented from dark red (low probability) to dark green (high probability).

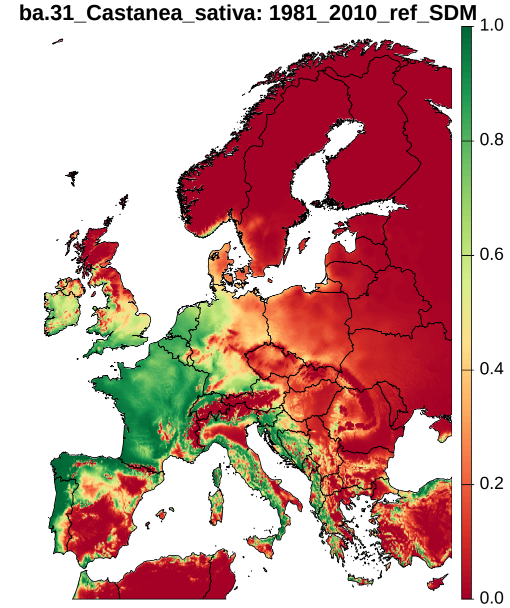

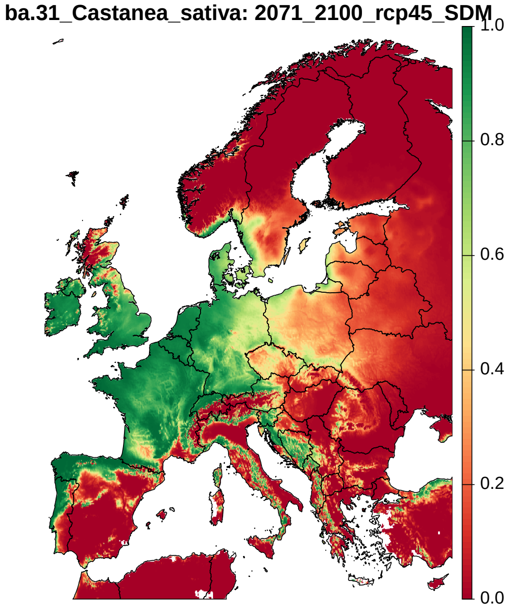

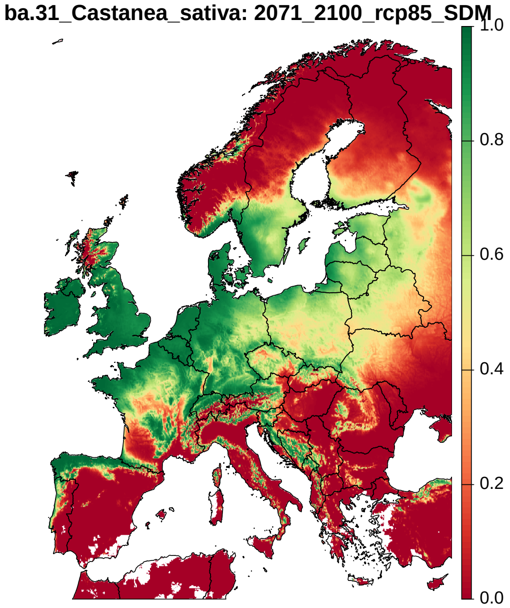

Climate risk classes

Maps of the climate risk classes. To estimate the distribution potential of each species as a mask for the SIMs, the continuous SDM outputs were categorized into three classes: low (yellow), medium (blue) and high climatic risk (red). The maps depict the risk classes in reference time (1981 to 2010), in climate scenario RCP4.5 (2071-2100) and RCP8.5 (2071-2100). To get an impression how well the thresholds fit to the data, presences (black) and absences (grey) were added on the reference map (top left). Refer to the legend and section "SDM thresholds" for the thresholds.

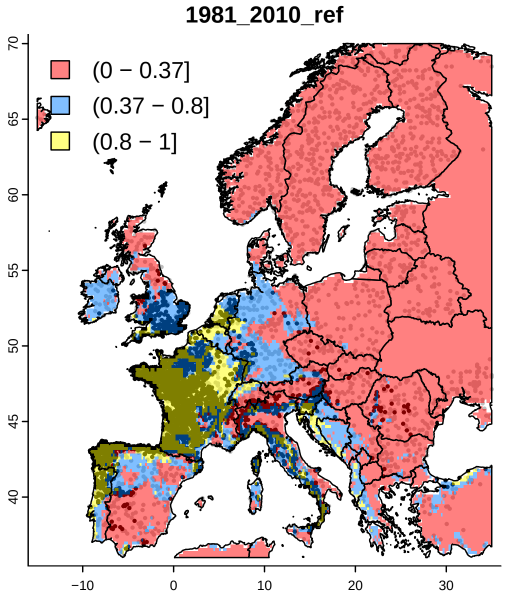

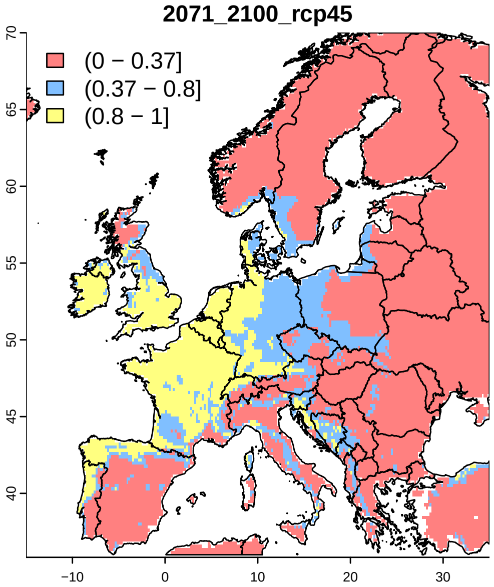

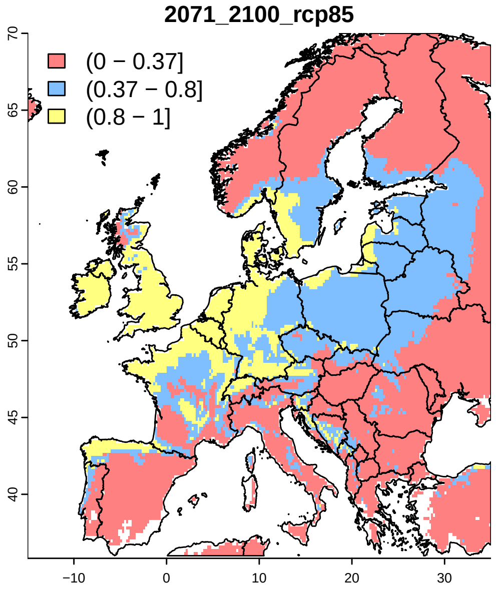

Fagus sylvatica

Model statistics and evaluation

Summary

Predictor acronyms: Bio.10 = Mean temperature of warmest quarter [°C] within months 6 to 8, Bio.11 = Mean temperature of coldest quarter [°C] within months 12,1,2, Bio.12 = Annual precipitation sum [mm/m2], Bio.18 = Mean monthly precipitation amount of the warmest quarter [mm/m2] within months 6 to 8.

```
##
## Family: binomial
## Link function: logit
##
## Formula:
## ba.9 ~ s(Bio.10, k = 3) + s(Bio.11, k = 3) + s(Bio.18, k = 3)
##
## Parametric coefficients:
##             Estimate Std. Error z value Pr(>|z|)
## (Intercept) -1.18469    0.04895   -24.2   <2e-16 ***
## ---
## Signif. codes:  0 '***' 0.001 '**' 0.01 '*' 0.05 '.' 0.1 ' ' 1
##
## Approximate significance of smooth terms:
##             edf Ref.df Chi.sq p-value
## s(Bio.10) 1.999      2  933.3  <2e-16 ***
## s(Bio.11) 1.998      2 1407.1  <2e-16 ***
## s(Bio.18) 1.993      2  741.6  <2e-16 ***
## ---
## Signif. codes:  0 '***' 0.001 '**' 0.01 '*' 0.05 '.' 0.1 ' ' 1
##
## R-sq.(adj) =  0.553   Deviance explained = 47.7%
## -REML =   4391  Scale est. = 1         n = 12060
```

Evaluation parameter

Model performance was assessed using four statistical parameters: the area under the receiver operating characteristic curve (AUC), the true skill statistic (TSS), sensitivity (probability of the model to correctly predict a true presence) and specificity (probability of the model to correctly predict a true absence).

```
##       Species_name  AUC       TSS sensitivity specificity
## tp Fagus sylvatica 0.91 0.7004975   0.9227197   0.7777778
```

Response curves and response maps

Response curves

Response curves (also known as effect curves) give an overview of the climatic niche of a species by relating the occurrence probability to corresponding climatic values. Predictor acronyms: Bio.10 = Mean temperature of warmest quarter [°C] within months 6 to 8, Bio.11 = Mean temperature of coldest quarter [°C] within months 12,1,2, Bio.12 = Annual precipitation sum [mm/m2], Bio.18 = Mean monthly precipitation amount of the warmest quarter [mm/m2] within months 6 to 8. Lines on the x-axis mark the upper and lower limit of the used presences (red), the mean (bold black) and the median (bold blue).

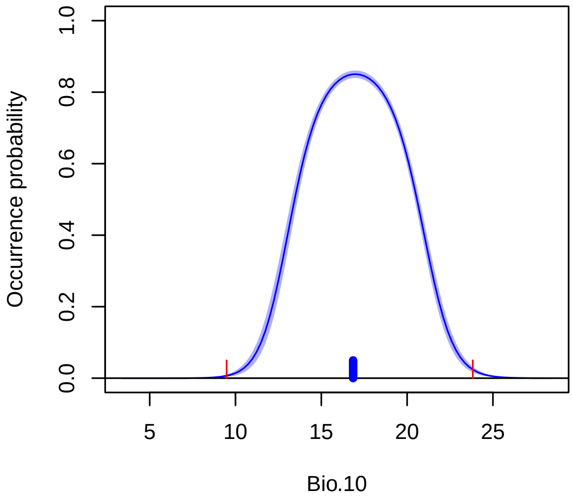

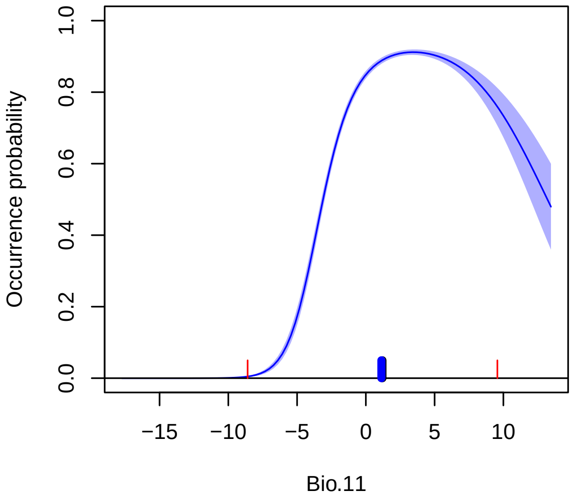

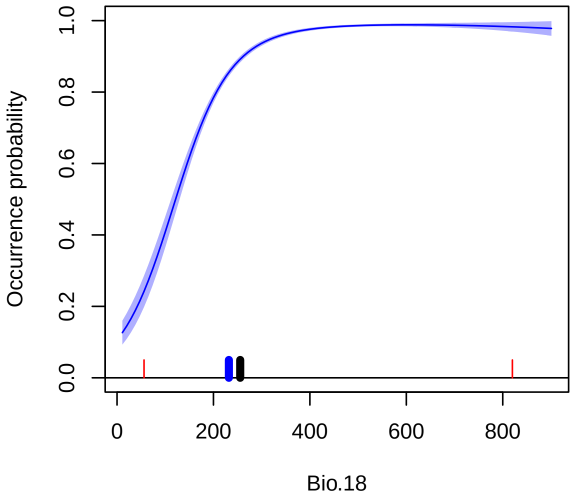

Response maps

Response maps (also referred to as partial effect maps). Each map represents how each predictor affects the occurrence probability. Predictor acronyms: Bio.10 = Mean temperature of warmest quarter [°C] within months 6 to 8, Bio.11 = Mean temperature of coldest quarter [°C] within months 12,1,2, Bio.12 = Annual precipitation sum [mm/m2], Bio.18 = Mean monthly precipitation amount of the warmest quarter [mm/m2] within months 6 to 8.

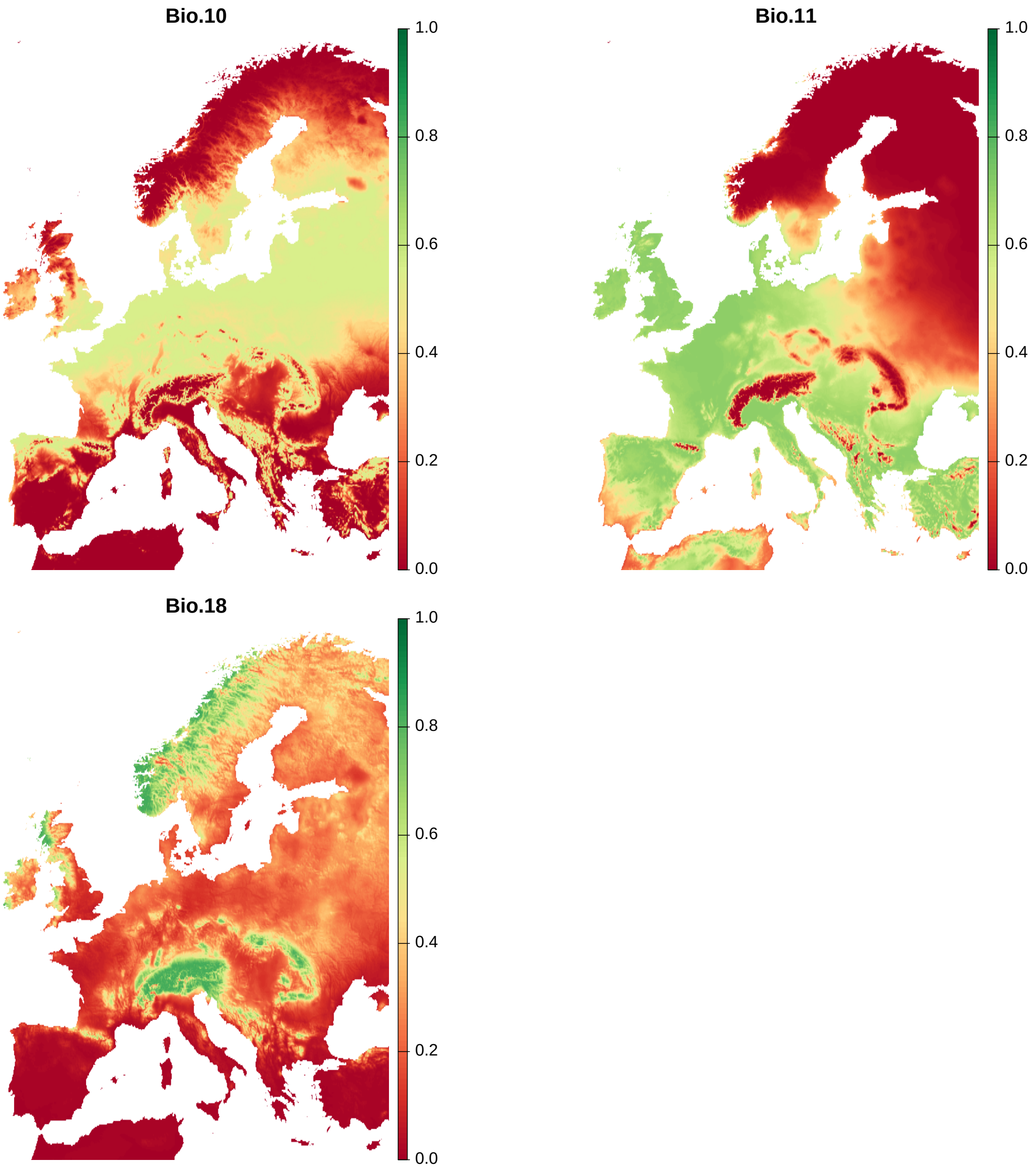

Model projections

Projection with plotted input data

Projection of species distribution model for reference period 1981-2010 over Europe. Occurrence probability ranges from 0 to 1 and is represented in dark red (low probability) to dark green (high probability). Input data used to calibrate the model is shown as presence points in magenta and absence points in black.

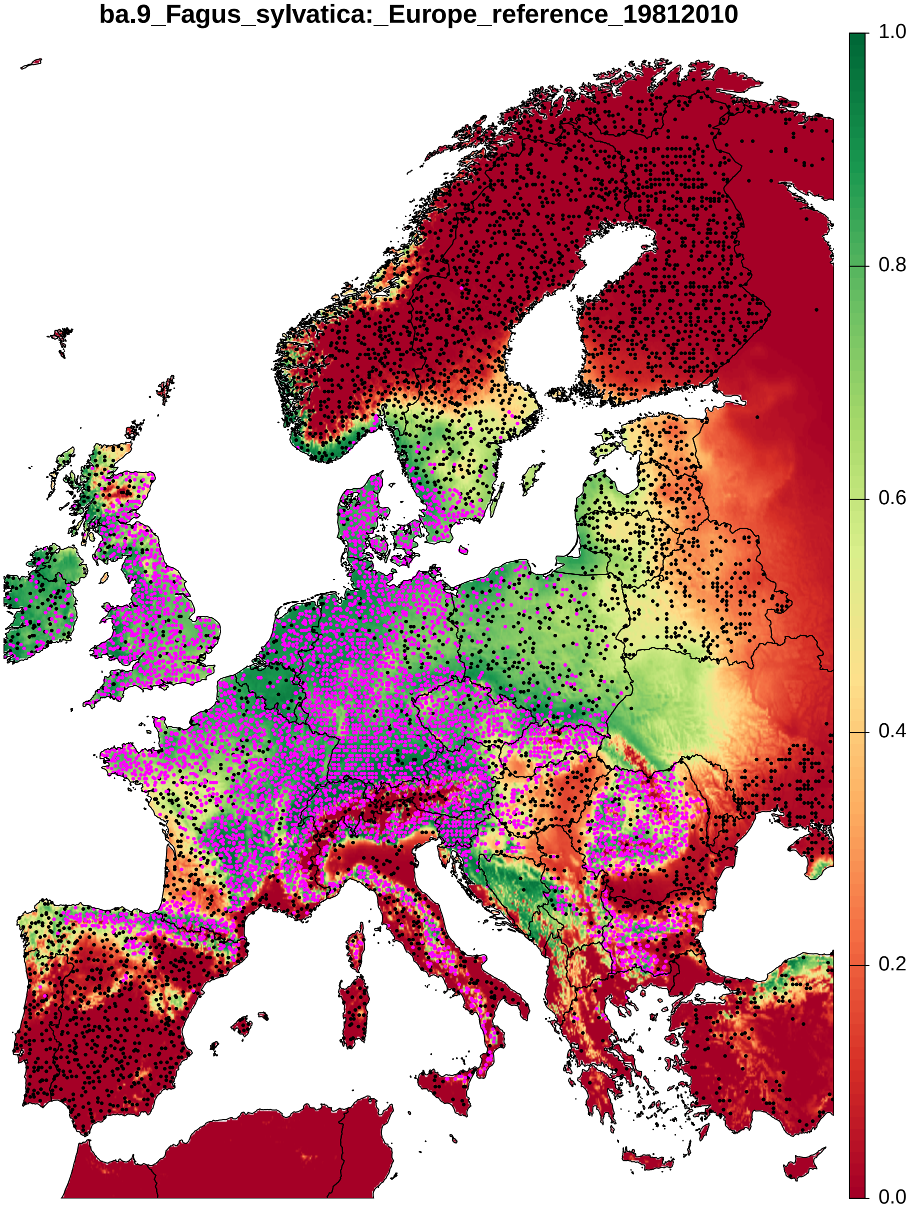

Projections

Projections of the species distribution models for reference period (1981-2010) and future scenarios RCP4.5 (2071-2100) and RCP8.5 (2071-2100) over Europe. Occurrence probabilities range from 0 to 1 and are represented from dark red (low probability) to dark green (high probability).

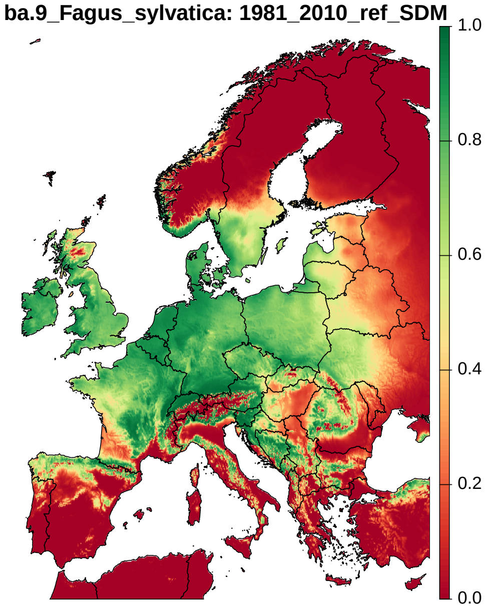

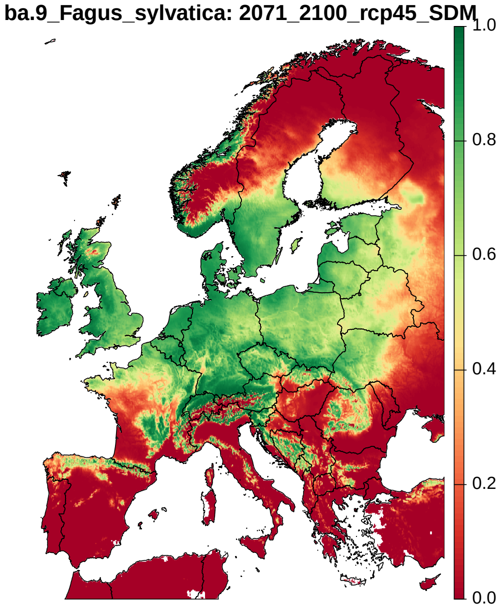

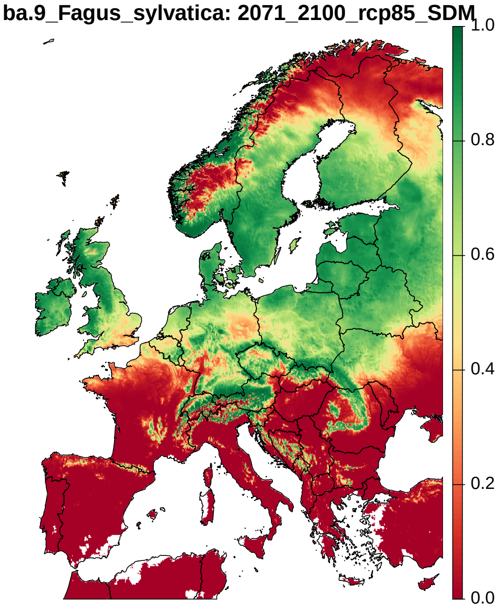

Climate risk classes

Maps of the climate risk classes. To estimate the distribution potential of each species as a mask for the SIMs, the continuous SDM outputs were categorized into three classes: low (yellow), medium (blue) and high climatic risk (red). The maps depict the risk classes in reference time (1981 to 2010), in climate scenario RCP4.5 (2071-2100) and RCP8.5 (2071-2100). To get an impression how well the thresholds fit to the data, presences (black) and absences (grey) were added on the reference map (top left). Refer to the legend and section "SDM thresholds" for the thresholds.

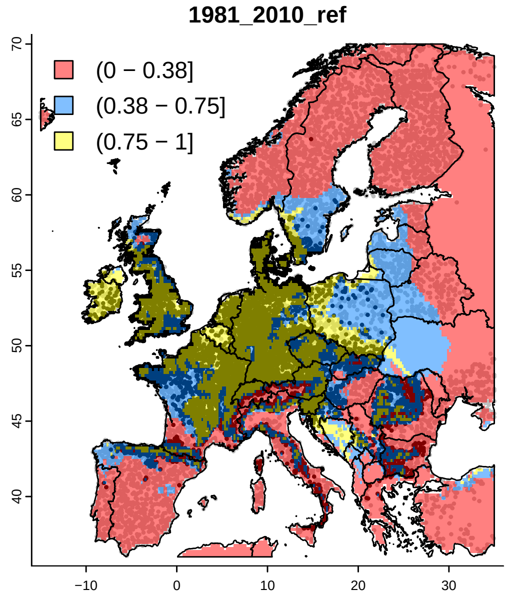

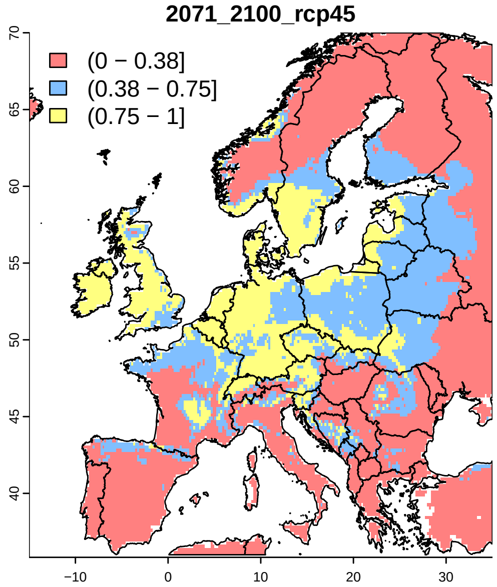

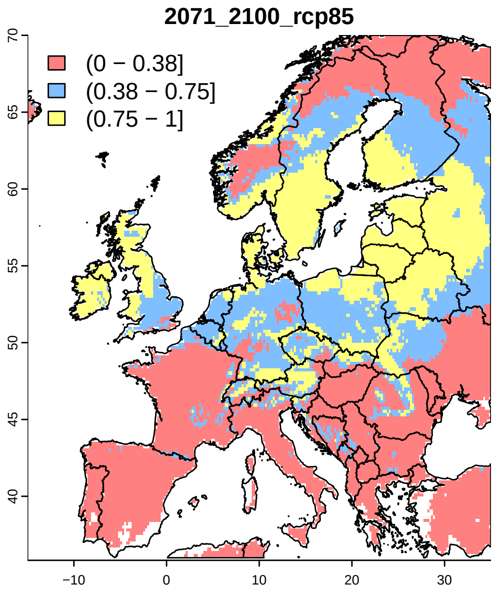

Fraxinus excelsior

Model statistics and evaluation

Summary

Predictor acronyms: Bio.10 = Mean temperature of warmest quarter [°C] within months 6 to 8, Bio.11 = Mean temperature of coldest quarter [°C] within months 12,1,2, Bio.12 = Annual precipitation sum [mm/m2], Bio.18 = Mean monthly precipitation amount of the warmest quarter [mm/m2] within months 6 to 8.

```
##
## Family: binomial
## Link function: logit
##
## Formula:
## ba.19 ~ s(Bio.10, k = 3) + s(Bio.11, k = 3) + s(Bio.18, k = 3)
##
## Parametric coefficients:
##             Estimate Std. Error z value Pr(>|z|)
## (Intercept) -0.72782    0.03933  -18.51   <2e-16 ***
## ---
## Signif. codes:  0 '***' 0.001 '**' 0.01 '*' 0.05 '.' 0.1 ' ' 1
##
## Approximate significance of smooth terms:
##             edf Ref.df Chi.sq p-value
## s(Bio.10) 1.999      2  756.0  <2e-16 ***
## s(Bio.11) 1.996      2 1476.9  <2e-16 ***
## s(Bio.18) 1.993      2  604.1  <2e-16 ***
## ---
## Signif. codes:  0 '***' 0.001 '**' 0.01 '*' 0.05 '.' 0.1 ' ' 1
##
## R-sq.(adj) =  0.448   Deviance explained = 38.8%
## -REML = 4907.2  Scale est. = 1         n = 11526
```

Evaluation parameter

Model performance was assessed using four statistical parameters: the area under the receiver operating characteristic curve (AUC), the true skill statistic (TSS), sensitivity (probability of the model to correctly predict a true presence) and specificity (probability of the model to correctly predict a true absence).

```
##          Species_name  AUC       TSS sensitivity specificity
## tp Fraxinus excelsior 0.88 0.5989936   0.8644803   0.7345133
```

Response curves and response maps

Response curves

Response curves (also known as effect curves) give an overview of the climatic niche of a species by relating the occurrence probability to corresponding climatic values. Predictor acronyms: Bio.10 = Mean temperature of warmest quarter [°C] within months 6 to 8, Bio.11 = Mean temperature of coldest quarter [°C] within months 12,1,2, Bio.12 = Annual precipitation sum [mm/m2], Bio.18 = Mean monthly precipitation amount of the warmest quarter [mm/m2] within months 6 to 8. Lines on the x-axis mark the upper and lower limit of the used presences (red), the mean (bold black) and the median (bold blue).

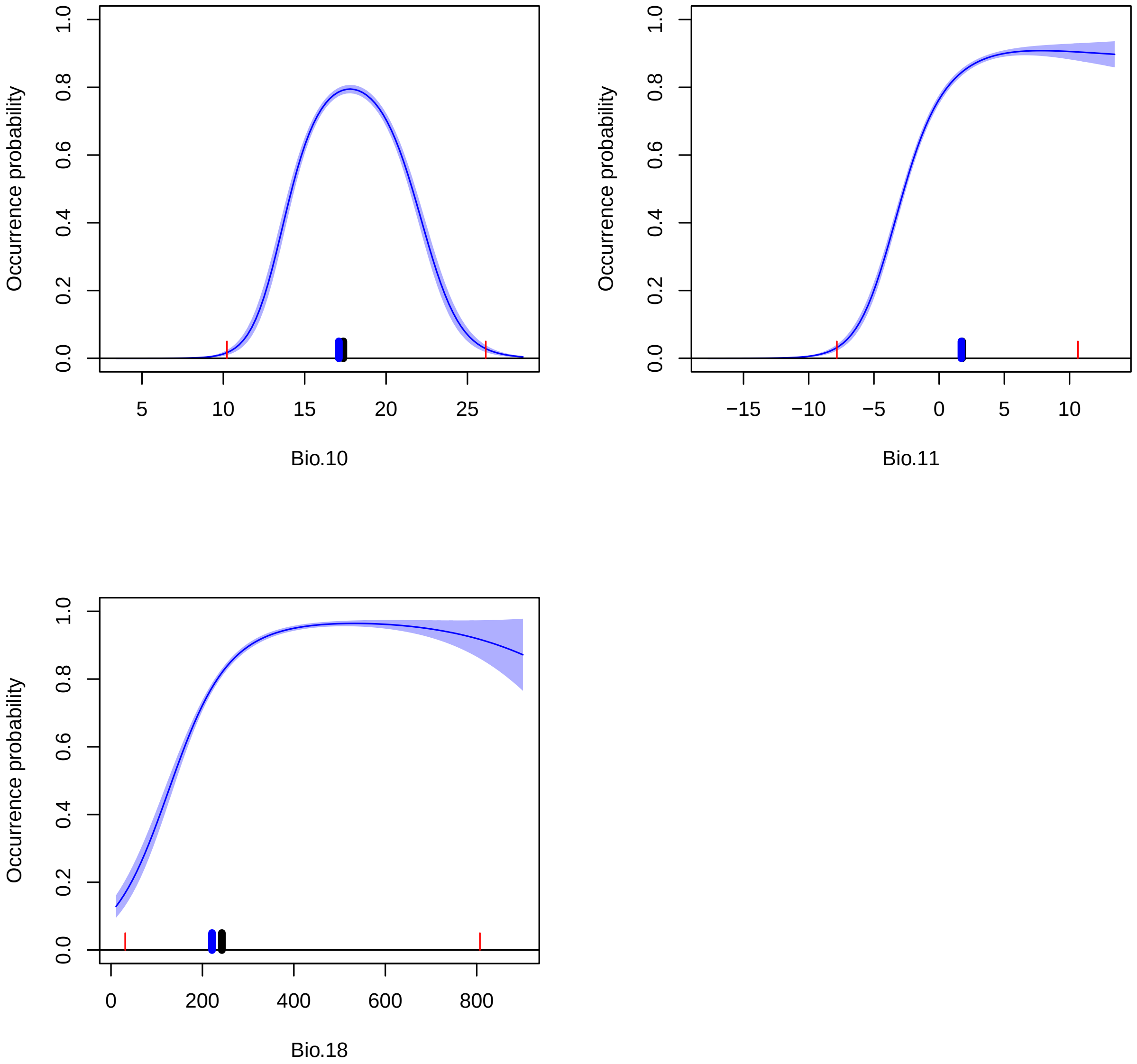

Response maps

Response maps (also referred to as partial effect maps). Each map represents how each predictor affects the occurrence probability. Predictor acronyms: Bio.10 = Mean temperature of warmest quarter [°C] within months 6 to 8, Bio.11 = Mean temperature of coldest quarter [°C] within months 12,1,2, Bio.12 = Annual precipitation sum [mm/m2], Bio.18 = Mean monthly precipitation amount of the warmest quarter [mm/m2] within months 6 to 8.

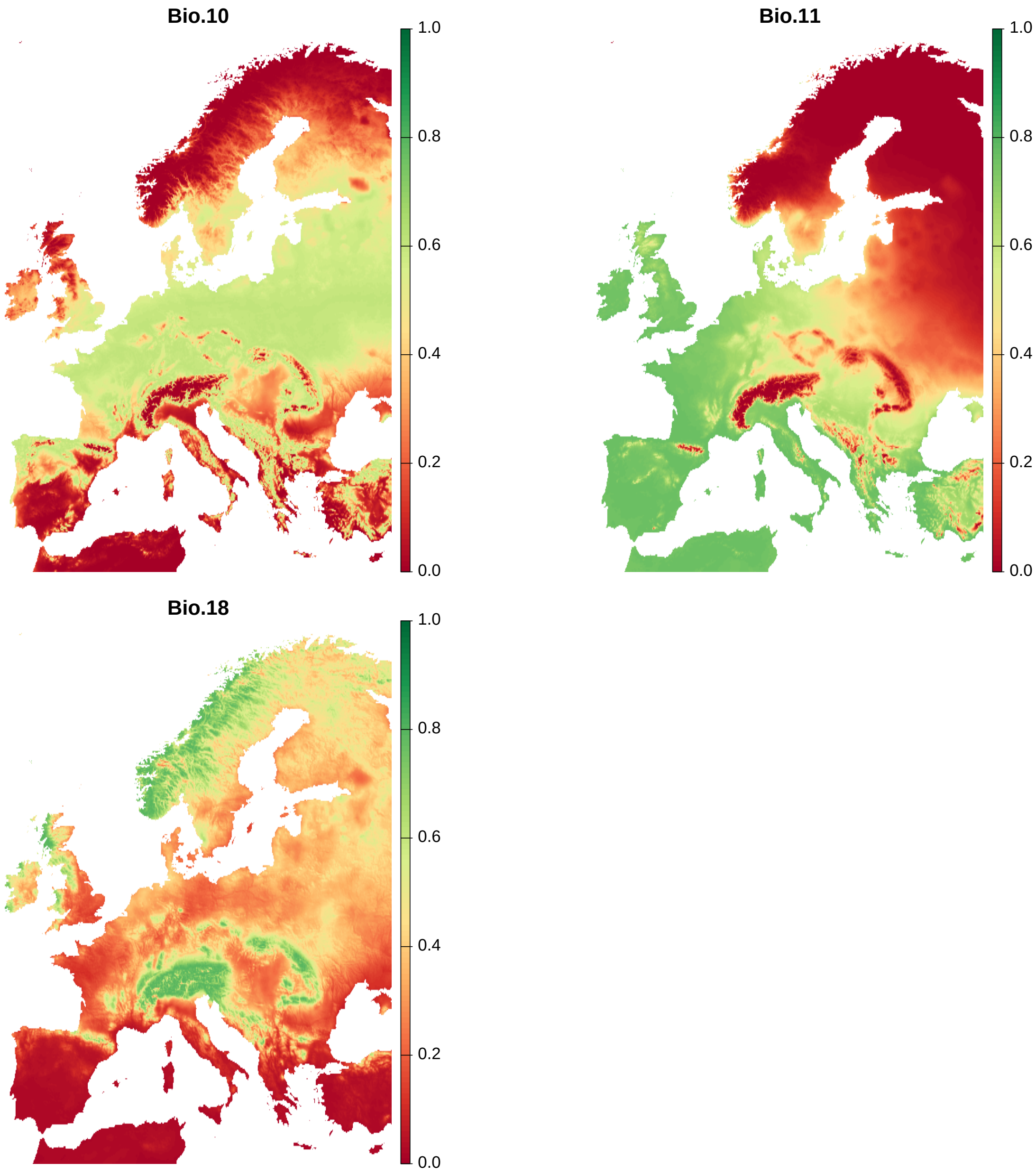

Model projections

Projection with plotted input data

Projection of species distribution model for reference period 1981-2010 over Europe. Occurrence probability ranges from 0 to 1 and is represented in dark red (low probability) to dark green (high probability). Input data used to calibrate the model is shown as presence points in magenta and absence points in black.

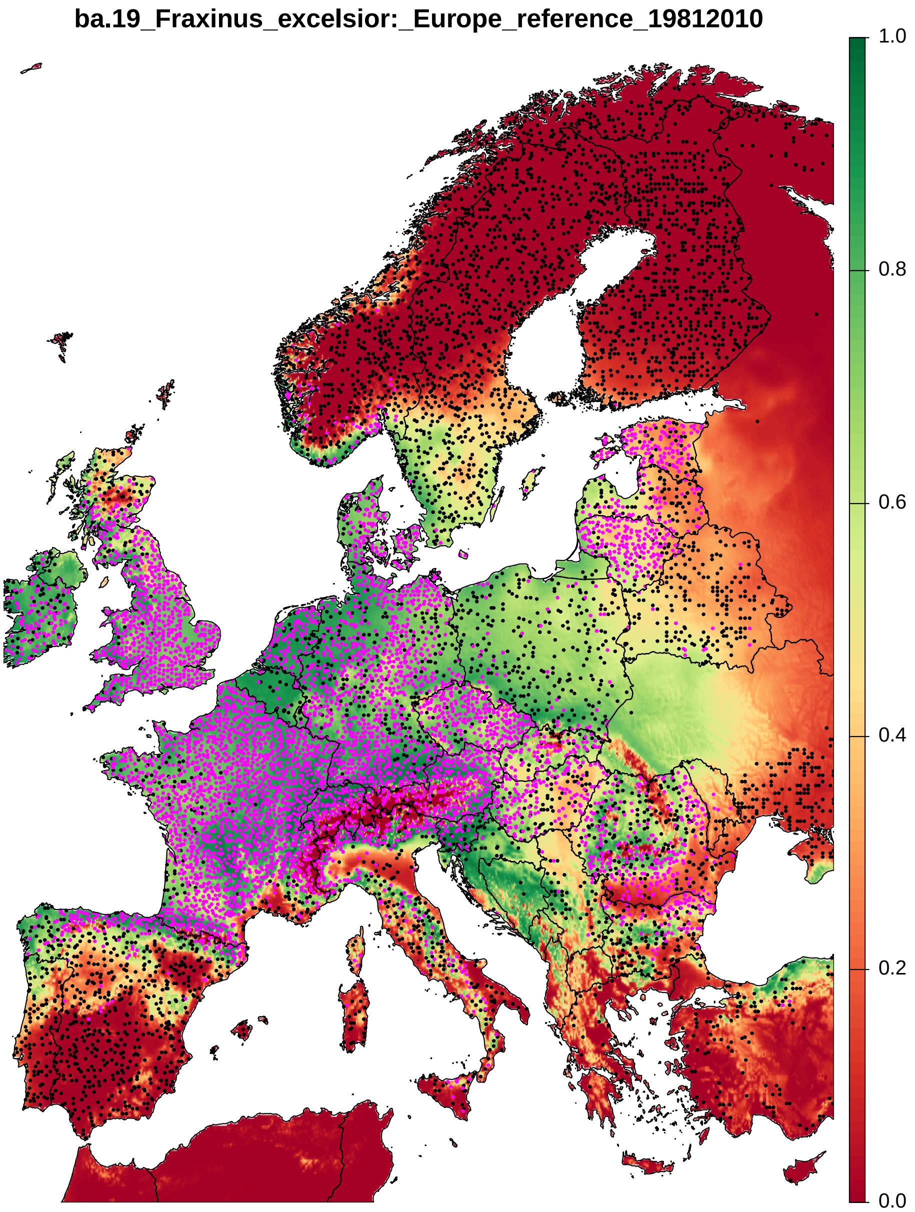

Projections

Projections of the species distribution models for reference period (1981-2010) and future scenarios RCP4.5 (2071-2100) and RCP8.5 (2071-2100) over Europe. Occurrence probabilities range from 0 to 1 and are represented from dark red (low probability) to dark green (high probability).

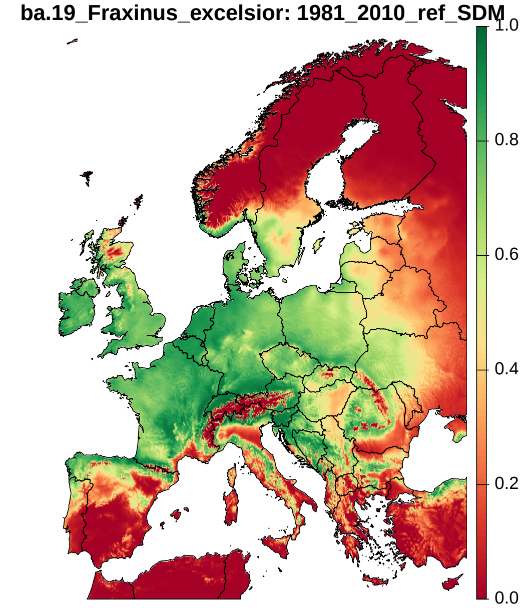

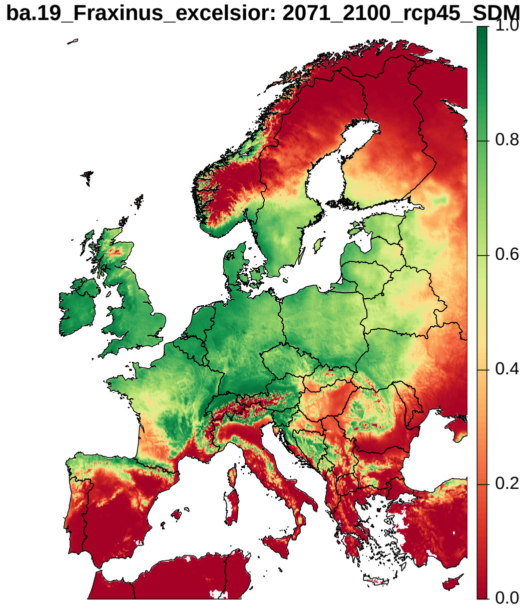

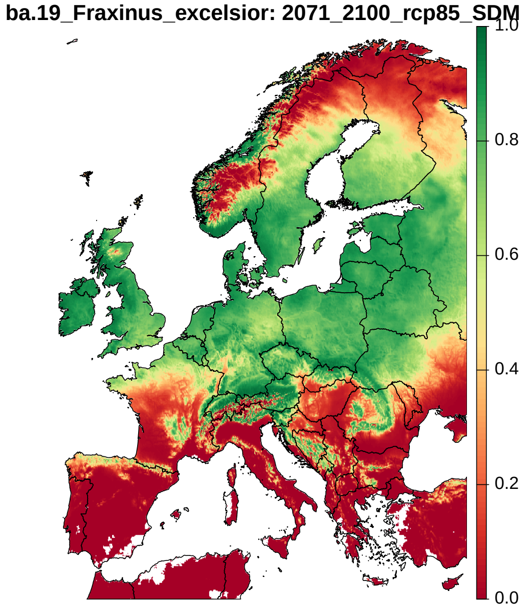

Climate risk classes

Maps of the climate risk classes. To estimate the distribution potential of each species as a mask for the SIMs, the continuous SDM outputs were categorized into three classes: low (yellow), medium (blue) and high climatic risk (red). The maps depict the risk classes in reference time (1981 to 2010), in climate scenario RCP4.5 (2071-2100) and RCP8.5 (2071-2100). To get an impression how well the thresholds fit to the data, presences (black) and absences (grey) were added on the reference map (top left). Refer to the legend and section "SDM thresholds" for the thresholds.

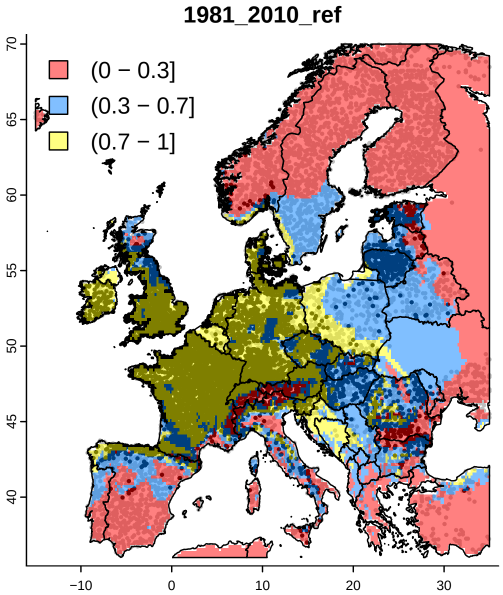

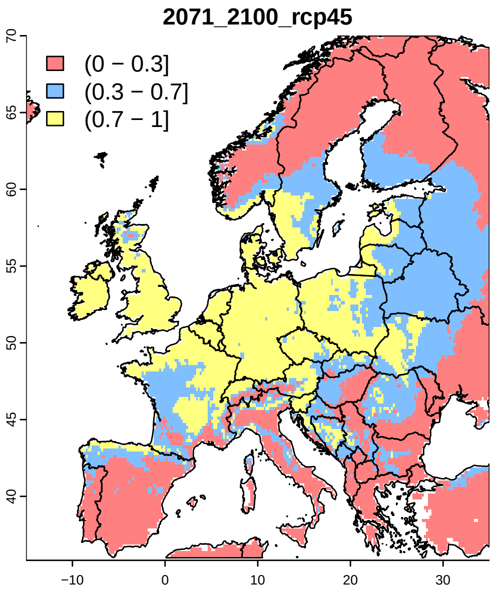

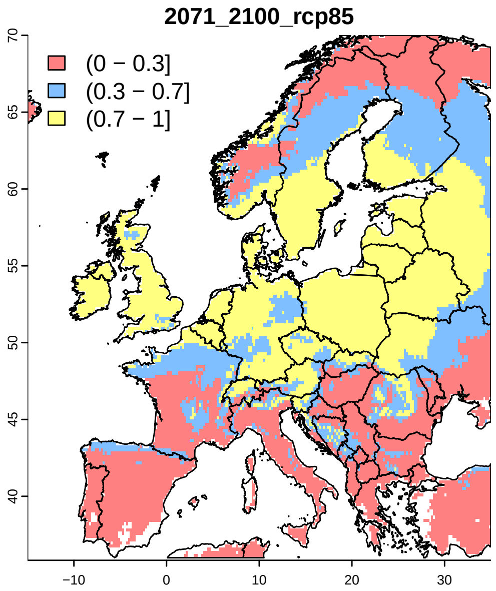

Juglans nigra

Model statistics and evaluation

Summary

Predictor acronyms: Bio.10 = Mean temperature of warmest quarter [°C] within months 6 to 8, Bio.11 = Mean temperature of coldest quarter [°C] within months 12,1,2, Bio.12 = Annual precipitation sum [mm/m2], Bio.18 = Mean monthly precipitation amount of the warmest quarter [mm/m2] within months 6 to 8.

```
##
## Family: binomial
## Link function: logit
##
## Formula:
## ba.86 ~ s(Bio.10, k = 3) + s(Bio.11, k = 3) + s(Bio.18, k = 3)
##
## Parametric coefficients:
##             Estimate Std. Error z value Pr(>|z|)
## (Intercept)  -2.0587     0.3043  -6.766 1.32e-11 ***
## ---
## Signif. codes:  0 '***' 0.001 '**' 0.01 '*' 0.05 '.' 0.1 ' ' 1
##
## Approximate significance of smooth terms:
##             edf Ref.df Chi.sq p-value
## s(Bio.10) 1.979  1.999  84.34  <2e-16 ***
## s(Bio.11) 1.944  1.997  33.94  <2e-16 ***
## s(Bio.18) 1.850  1.977  56.62  <2e-16 ***
## ---
## Signif. codes:  0 '***' 0.001 '**' 0.01 '*' 0.05 '.' 0.1 ' ' 1
##
## R-sq.(adj) =  0.538   Deviance explained = 47.6%
## -REML = 242.91  Scale est. = 1         n = 644
```

Evaluation parameter

Model performance was assessed using four statistical parameters: the area under the receiver operating characteristic curve (AUC), the true skill statistic (TSS), sensitivity (probability of the model to correctly predict a true presence) and specificity (probability of the model to correctly predict a true absence).

```
##     Species_name  AUC      TSS sensitivity specificity
## tp Juglans nigra 0.91 0.689441   0.8913043   0.7981366
```

Response curves and response maps

Response curves

Response curves (also known as effect curves) give an overview of the climatic niche of a species by relating the occurrence probability to corresponding climatic values. Predictor acronyms: Bio.10 = Mean temperature of warmest quarter [°C] within months 6 to 8, Bio.11 = Mean temperature of coldest quarter [°C] within months 12,1,2, Bio.12 = Annual precipitation sum [mm/m2], Bio.18 = Mean monthly precipitation amount of the warmest quarter [mm/m2] within months 6 to 8. Lines on the x-axis mark the upper and lower limit of the used presences (red), the mean (bold black) and the median (bold blue).

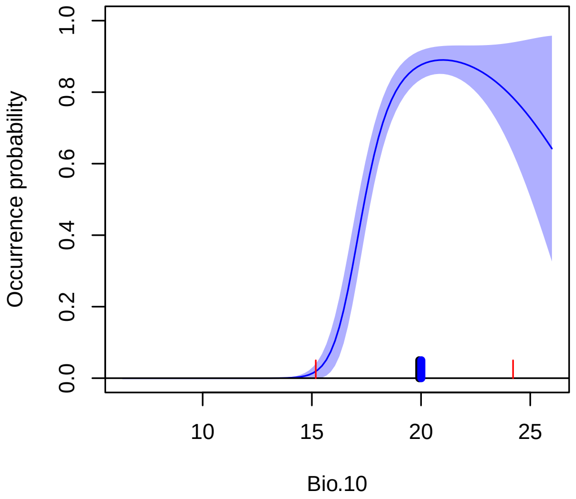

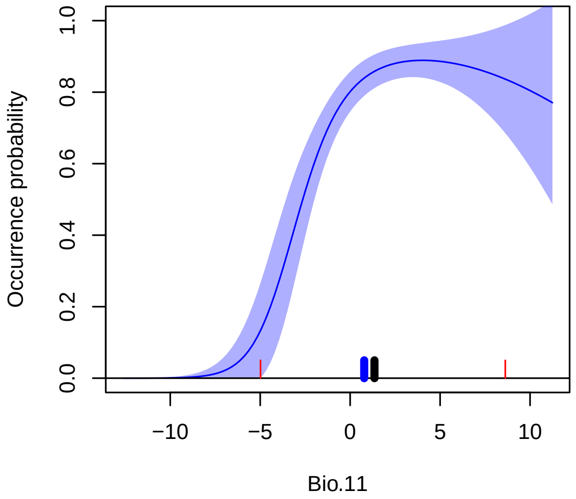

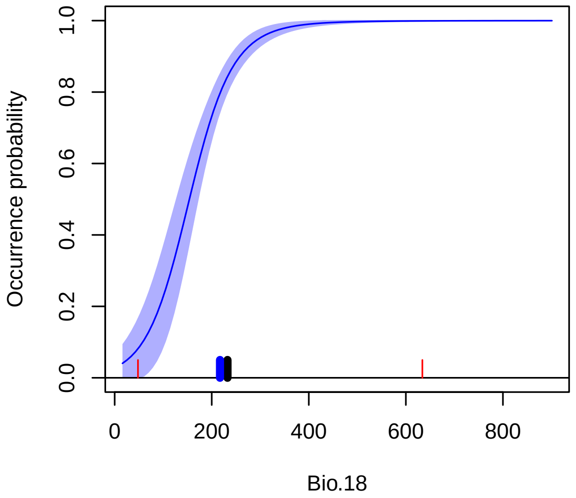

Response maps

Response maps (also referred to as partial effect maps). Each map represents how each predictor affects the occurrence probability. Predictor acronyms: Bio.10 = Mean temperature of warmest quarter [°C] within months 6 to 8, Bio.11 = Mean temperature of coldest quarter [°C] within months 12,1,2, Bio.12 = Annual precipitation sum [mm/m2], Bio.18 = Mean monthly precipitation amount of the warmest quarter [mm/m2] within months 6 to 8.

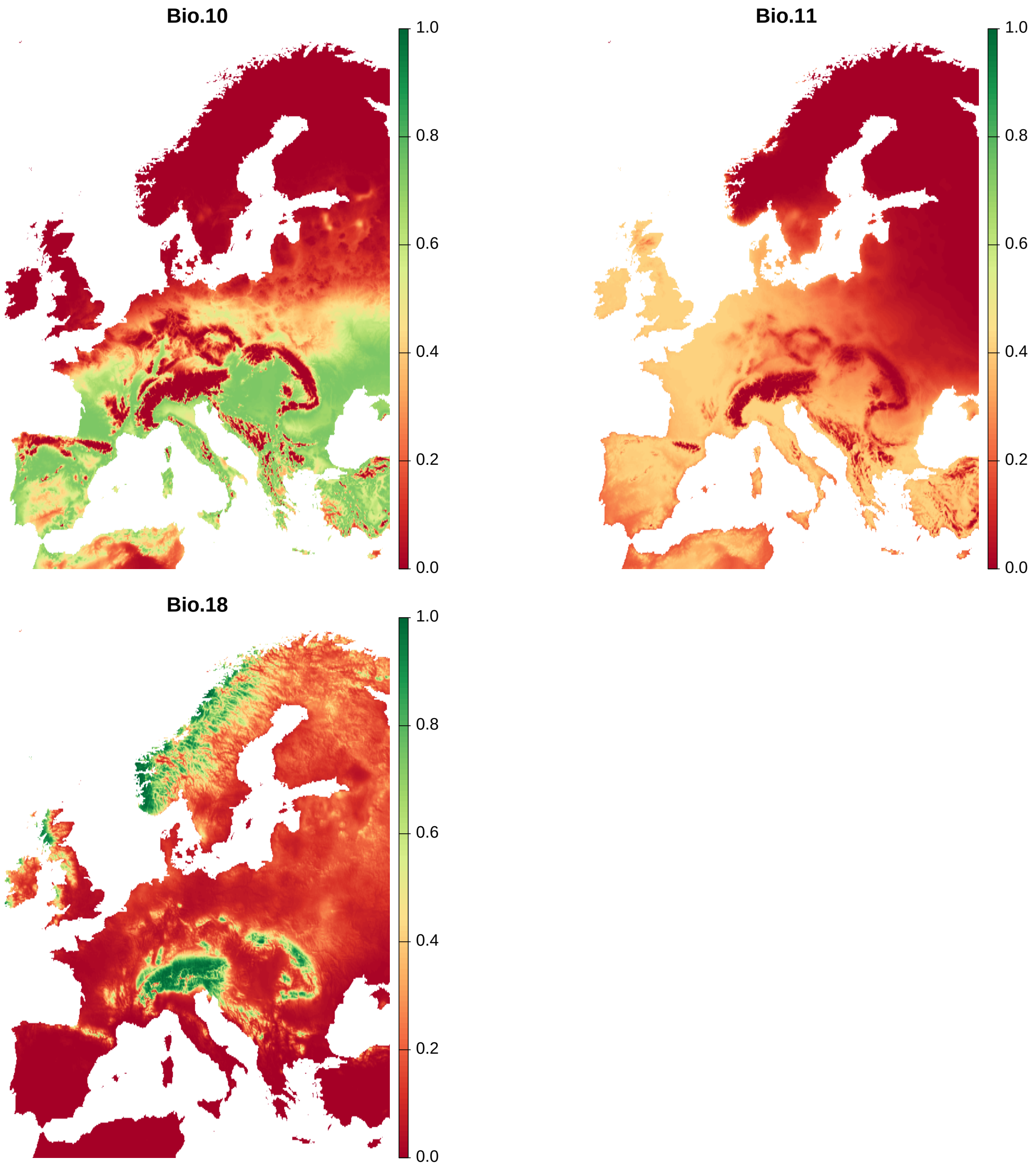

Model projections

Projection with plotted input data

Projection of species distribution model for reference period 1981-2010 over Europe. Occurrence probability ranges from 0 to 1 and is represented in dark red (low probability) to dark green (high probability). Input data used to calibrate the model is shown as presence points in magenta and absence points in black.

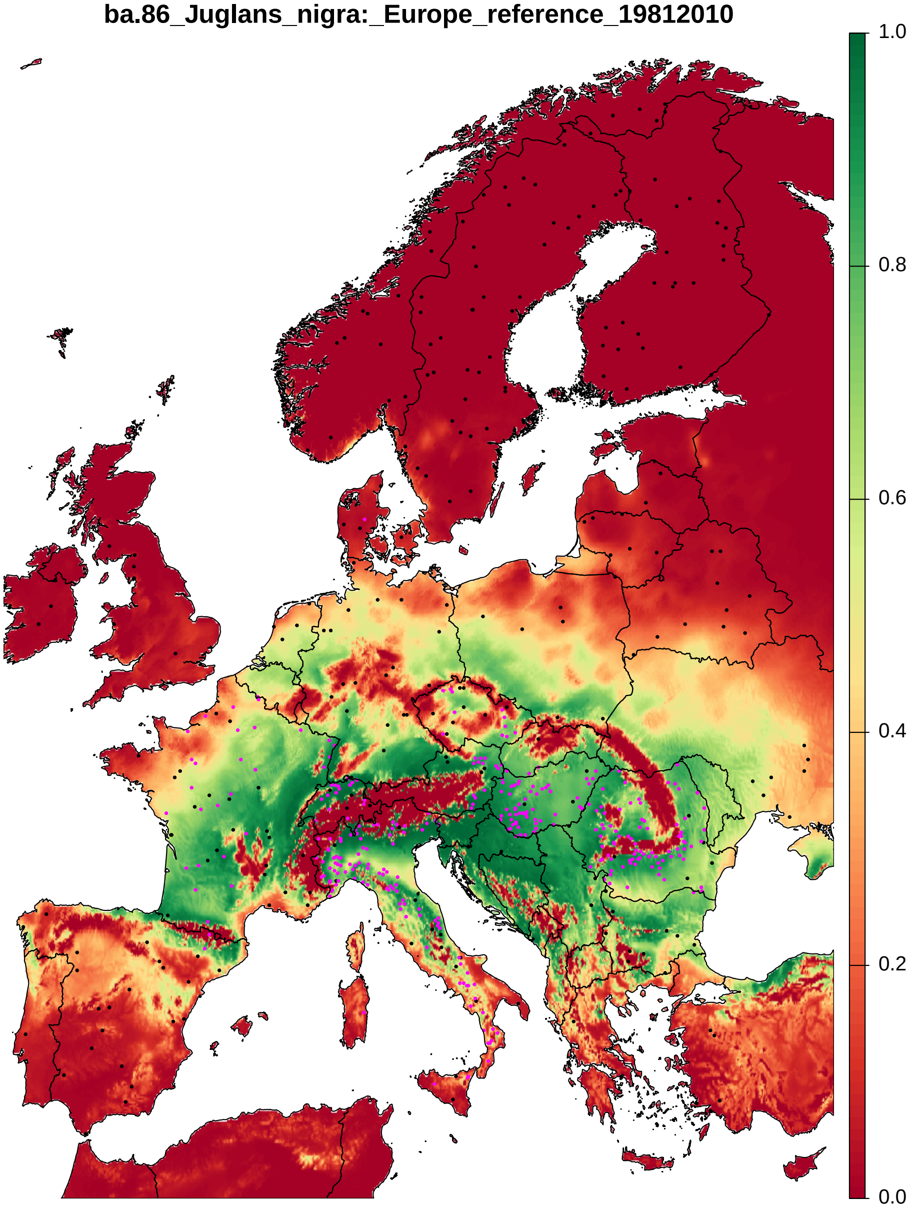

Projections

Projections of the species distribution models for reference period (1981-2010) and future scenarios RCP4.5 (2071-2100) and RCP8.5 (2071-2100) over Europe. Occurrence probabilities range from 0 to 1 and are represented from dark red (low probability) to dark green (high probability).

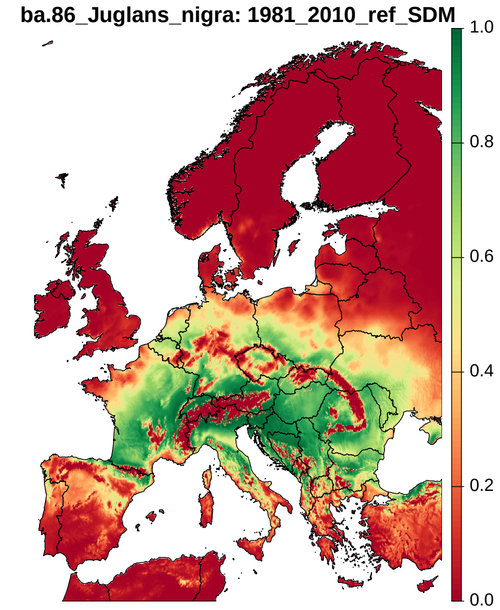

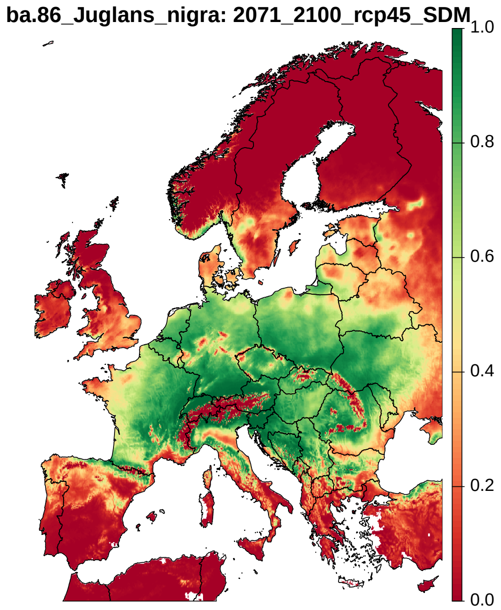

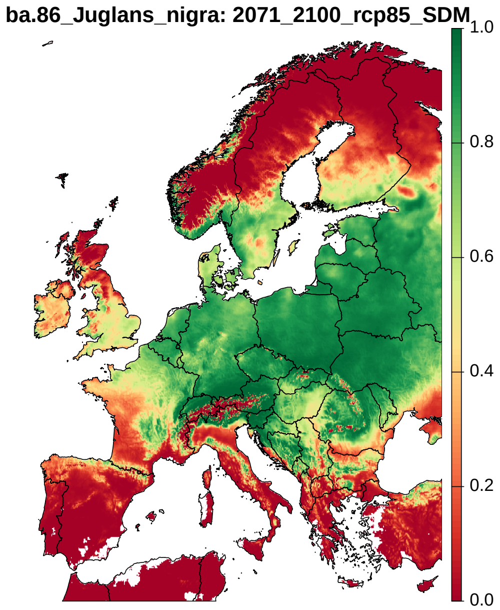

Climate risk classes

Maps of the climate risk classes. To estimate the distribution potential of each species as a mask for the SIMs, the continuous SDM outputs were categorized into three classes: low (yellow), medium (blue) and high climatic risk (red). The maps depict the risk classes in reference time (1981 to 2010), in climate scenario RCP4.5 (2071-2100) and RCP8.5 (2071-2100). To get an impression how well the thresholds fit to the data, presences (black) and absences (grey) were added on the reference map (top left). Refer to the legend and section “SDM thresholds” for the thresholds.

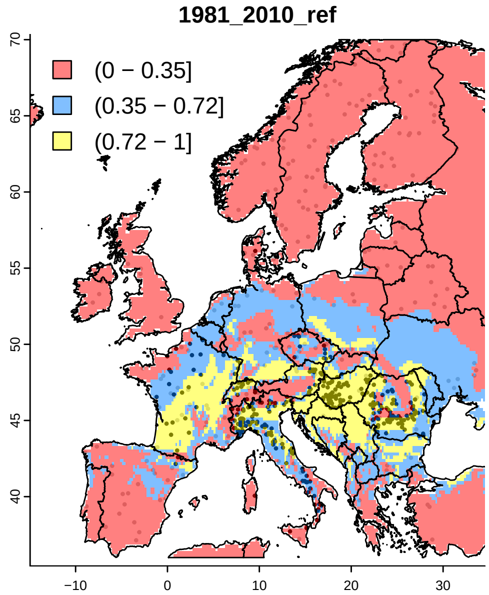

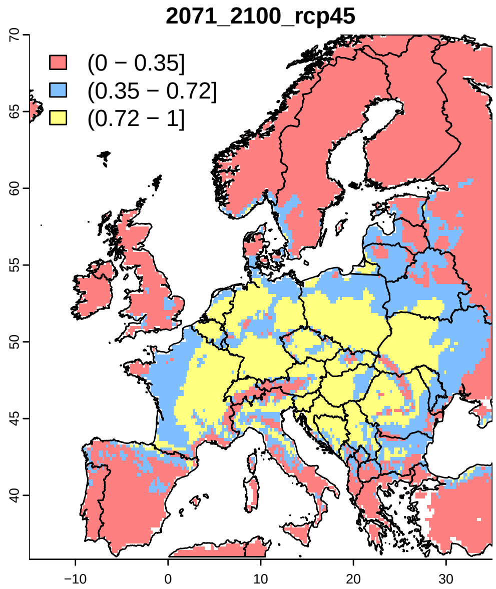

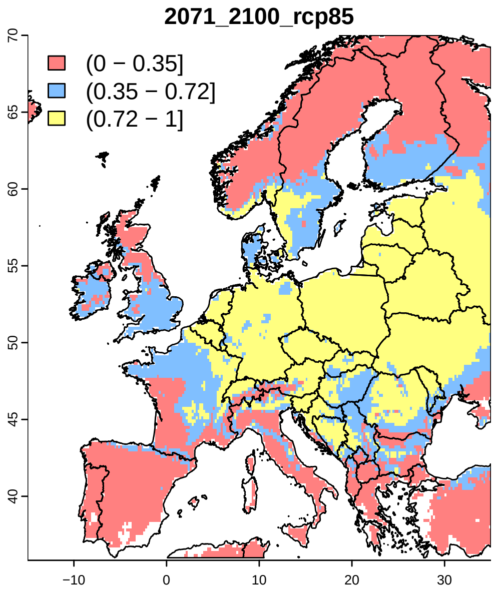

Juglans regia

Model statistics and evaluation

Summary

Predictor acronyms: Bio.10 = Mean temperature of warmest quarter [°C] within months 6 to 8, Bio.11 = Mean temperature of coldest quarter [°C] within months 12,1,2, Bio.12 = Annual precipitation sum [mm/m2], Bio.18 = Mean monthly precipitation amount of the warmest quarter [mm/m2] within months 6 to 8.

```
##
## Family: binomial
## Link function: logit
##
## Formula:
## ba.85 ~ s(Bio.10, k = 3) + s(Bio.11, k = 3) + s(Bio.18, k = 3)
##
## Parametric coefficients:
##             Estimate Std. Error z value Pr(>|z|)
## (Intercept)  -1.4555     0.1395  -10.43   <2e-16 ***
## ---
## Signif. codes:  0 '***' 0.001 '**' 0.01 '*' 0.05 '.' 0.1 ' ' 1
##
## Approximate significance of smooth terms:
##             edf Ref.df Chi.sq p-value
## s(Bio.10) 1.994  2.000  216.1  <2e-16 ***
## s(Bio.11) 1.973  1.999  145.7  <2e-16 ***
## s(Bio.18) 1.000  1.000  182.8  <2e-16 ***
## ---
## Signif. codes:  0 '***' 0.001 '**' 0.01 '*' 0.05 '.' 0.1 ' ' 1
##
## R-sq.(adj) =  0.488   Deviance explained = 42.2%
## -REML = 711.24  Scale est. = 1         n = 1746
```

Evaluation parameter

Model performance was assessed using four statistical parameters: the area under the receiver operating characteristic curve (AUC), the true skill statistic (TSS), sensitivity (probability of the model to correctly predict a true presence) and specificity (probability of the model to correctly predict a true absence).

```
##     Species_name  AUC       TSS sensitivity specificity
## tp Juglans regia 0.90 0.6334479   0.8762887   0.7571592
```

Response curves and response maps

Response curves

Response curves (also known as effect curves) give an overview of the climatic niche of a species by relating the occurrence probability to corresponding climatic values. Predictor acronyms: Bio.10 = Mean temperature of warmest quarter [°C] within months 6 to 8, Bio.11 = Mean temperature of coldest quarter [°C] within months 12,1,2, Bio.12 = Annual precipitation sum [mm/m2], Bio.18 = Mean monthly precipitation amount of the warmest quarter [mm/m2] within months 6 to 8. Lines on the x-axis mark the upper and lower limit of the used presences (red), the mean (bold black) and the median (bold blue).

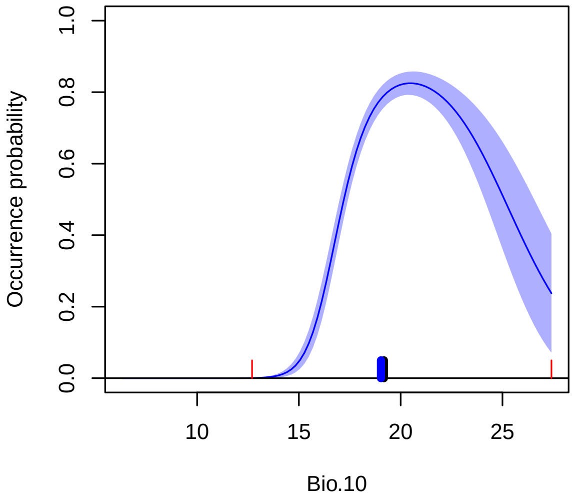

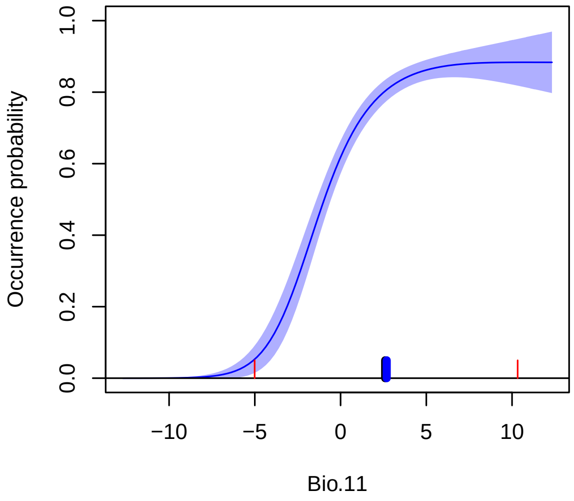

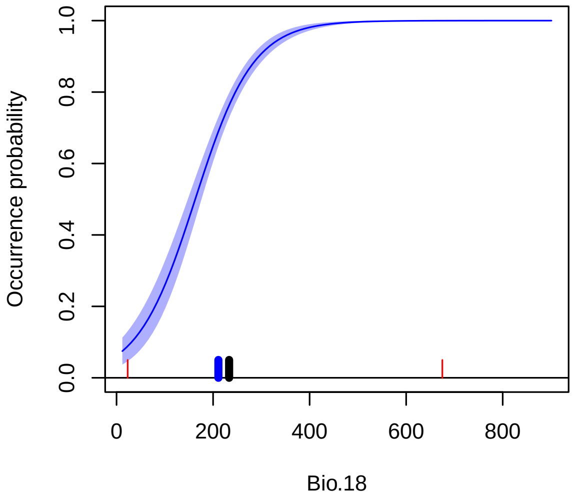

Response maps

Response maps (also referred to as partial effect maps). Each map represents how each predictor affects the occurrence probability. Predictor acronyms: Bio.10 = Mean temperature of warmest quarter [°C] within months 6 to 8, Bio.11 = Mean temperature of coldest quarter [°C] within months 12,1,2, Bio.12 = Annual precipitation sum [mm/m2], Bio.18 = Mean monthly precipitation amount of the warmest quarter [mm/m2] within months 6 to 8.

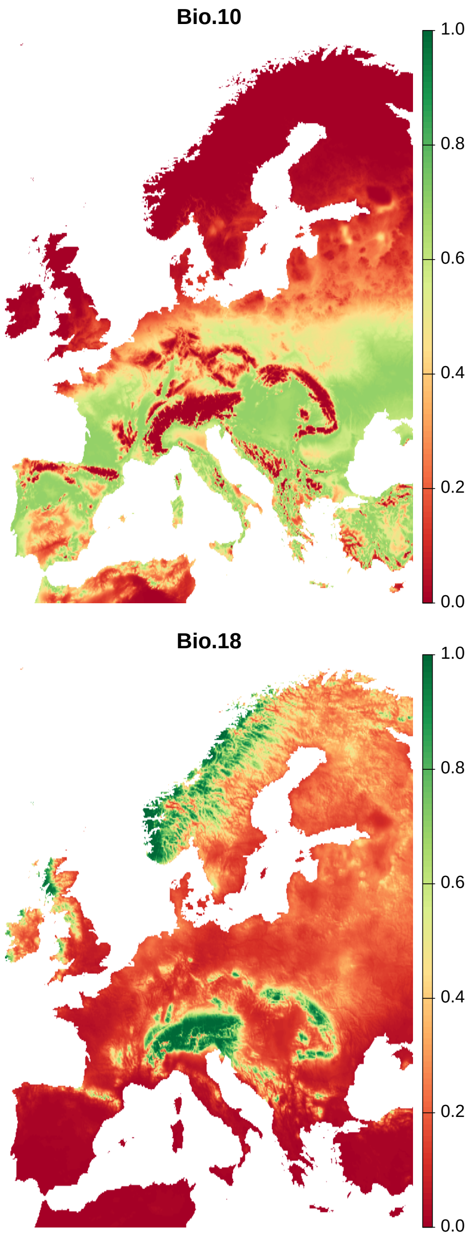

Model projections

Projection with plotted input data

Projection of species distribution model for reference period 1981-2010 over Europe. Occurrence probability ranges from 0 to 1 and is represented in dark red (low probability) to dark green (high probability). Input data used to calibrate the model is shown as presence points in magenta and absence points in black.

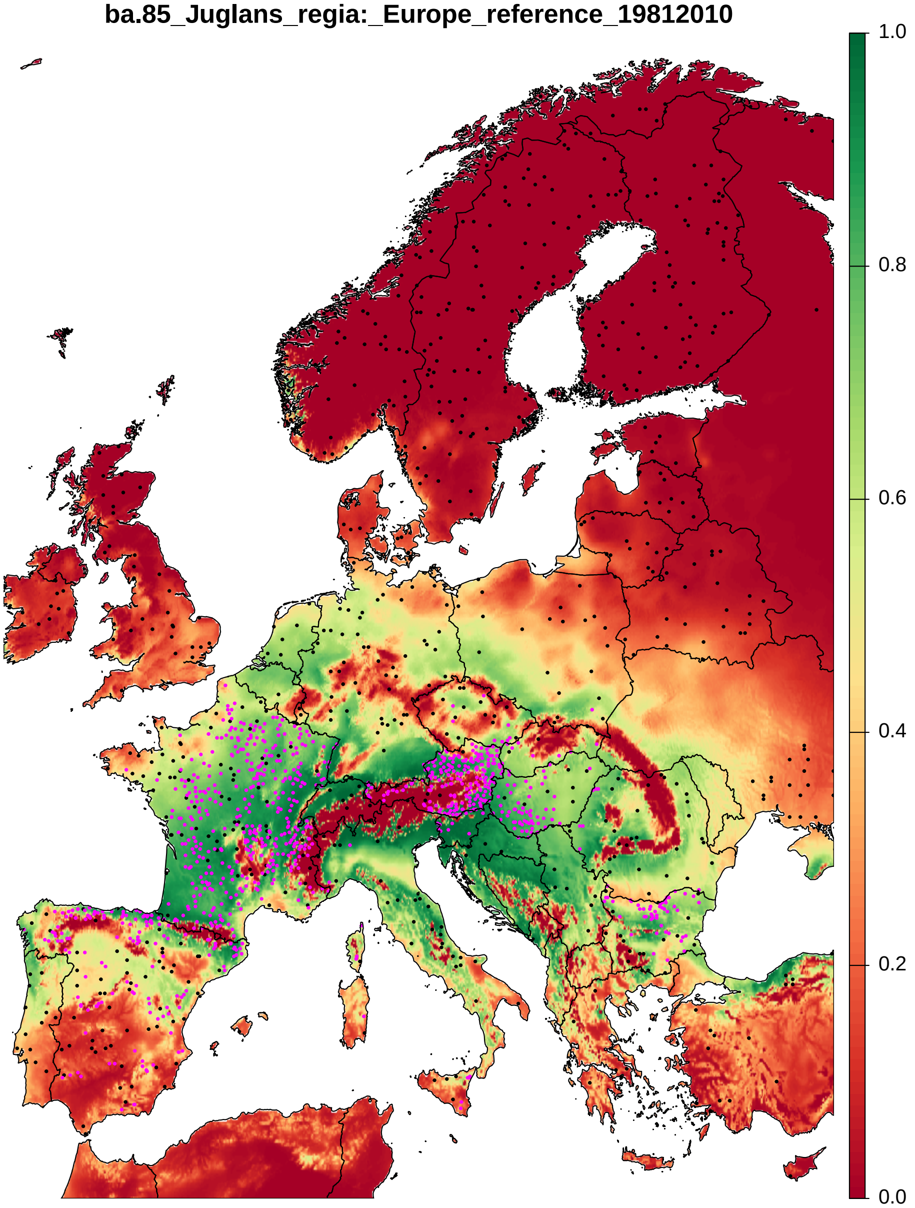

Projections

Projections of the species distribution models for reference period (1981-2010) and future scenarios RCP4.5 (2071-2100) and RCP8.5 (2071-2100) over Europe. Occurrence probabilities range from 0 to 1 and are represented from dark red (low probability) to dark green (high probability).

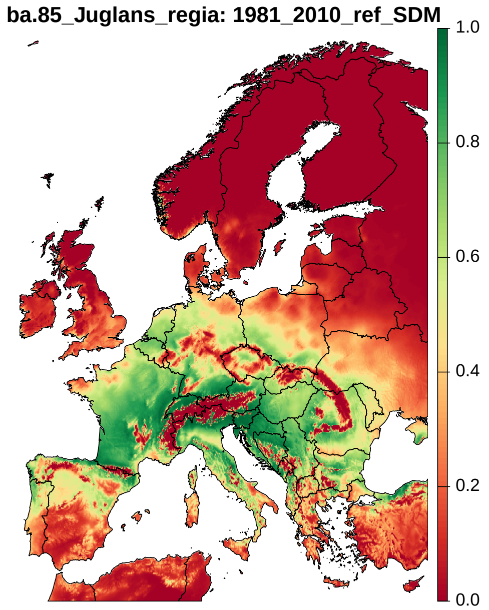

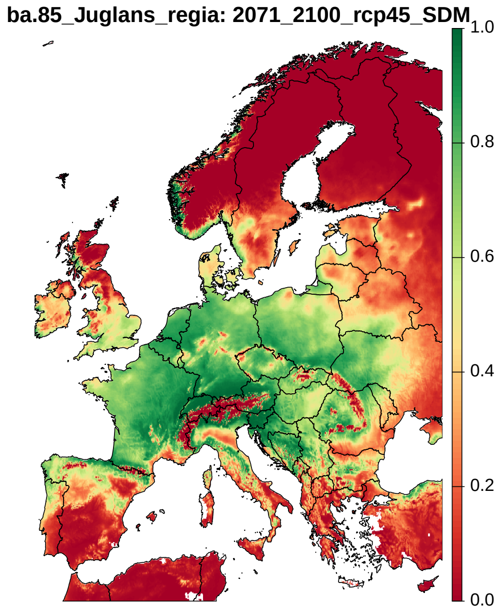

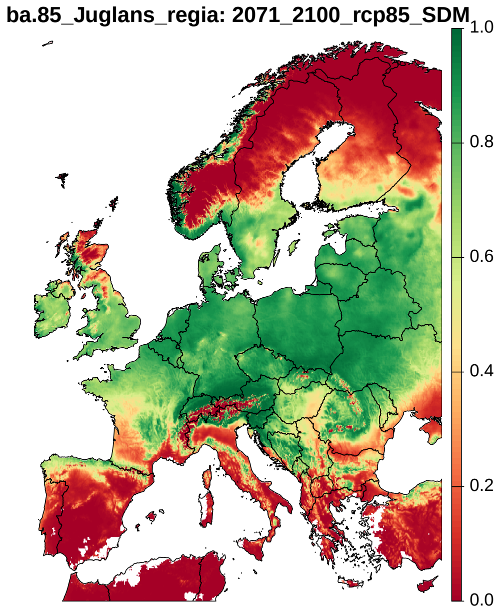

Climate risk classes

Maps of the climate risk classes. To estimate the distribution potential of each species as a mask for the SIMs, the continuous SDM outputs were categorized into three classes: low (yellow), medium (blue) and high climatic risk (red). The maps depict the risk classes in reference time (1981 to 2010), in climate scenario RCP4.5 (2071-2100) and RCP8.5 (2071-2100). To get an impression how well the thresholds fit to the data, presences (black) and absences (grey) were added on the reference map (top left). Refer to the legend and section “SDM thresholds” for the thresholds.

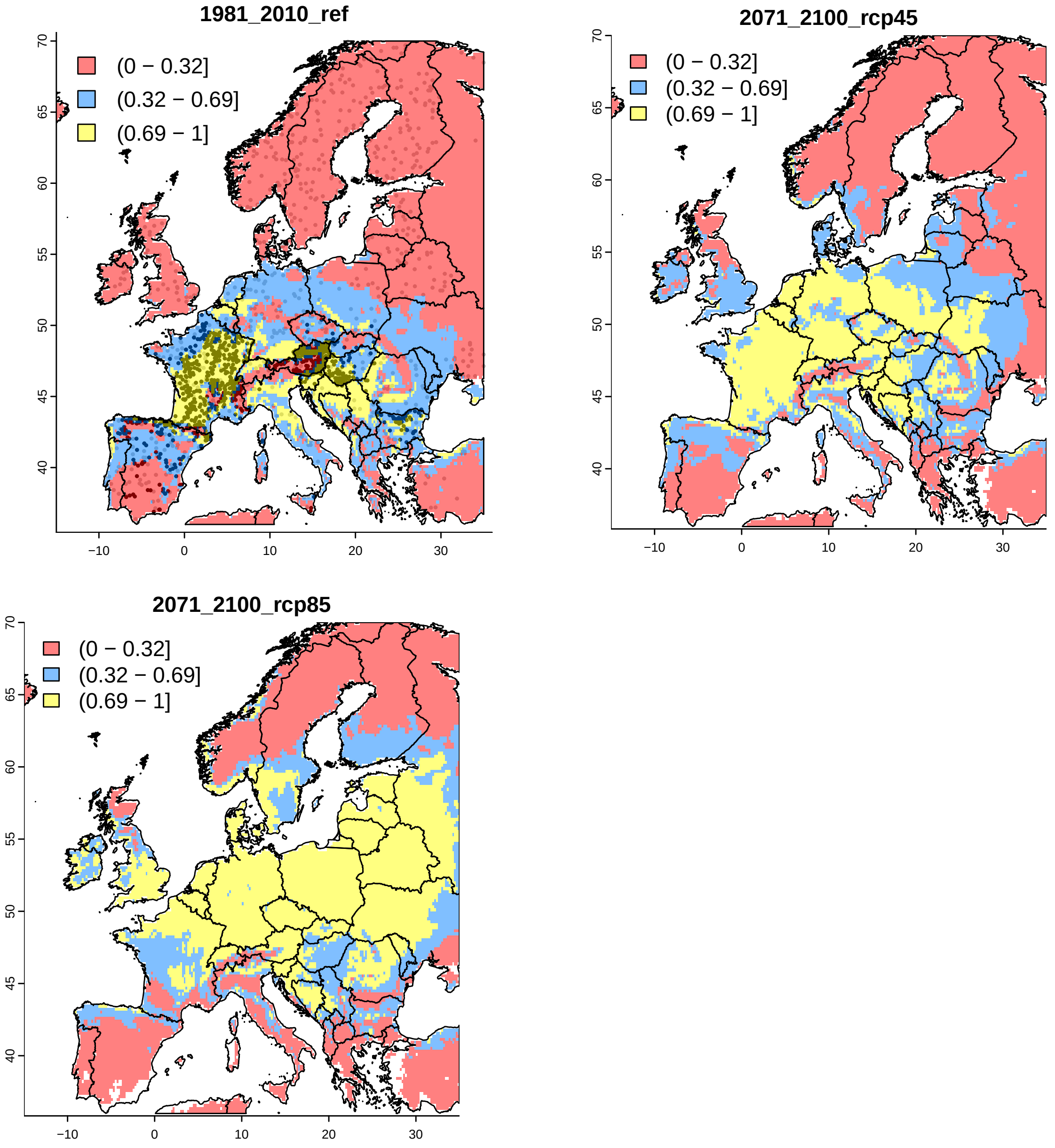

Larix decidua

Model statistics and evaluation

Summary

Predictor acronyms: Bio.10 = Mean temperature of warmest quarter [°C] within months 6 to 8, Bio.11 = Mean temperature of coldest quarter [°C] within months 12,1,2, Bio.12 = Annual precipitation sum [mm/m2], Bio.18 = Mean monthly precipitation amount of the warmest quarter [mm/m2] within months 6 to 8.

```
##
## Family: binomial
## Link function: logit
##
## Formula:
## ba.5 ~ s(Bio.10, k = 3) + s(Bio.11, k = 3) + s(Bio.18, k = 3)
##
## Parametric coefficients:
##             Estimate Std. Error z value Pr(>|z|)
## (Intercept) -0.31914    0.04155   -7.68 1.59e-14 ***
## ---
## Signif. codes:  0 '***' 0.001 '**' 0.01 '*' 0.05 '.' 0.1 ' ' 1
##
## Approximate significance of smooth terms:
##             edf Ref.df Chi.sq p-value
## s(Bio.10) 1.981  2.000  230.5  <2e-16 ***
## s(Bio.11) 1.996  2.000  405.0  <2e-16 ***
## s(Bio.18) 1.962  1.999  184.5  <2e-16 ***
## ---
## Signif. codes:  0 '***' 0.001 '**' 0.01 '*' 0.05 '.' 0.1 ' ' 1
##
## R-sq.(adj) =  0.352   Deviance explained = 28.5%
## -REML = 2441.8  Scale est. = 1         n = 4890
```

Evaluation parameter

Model performance was assessed using four statistical parameters: the area under the receiver operating characteristic curve (AUC), the true skill statistic (TSS), sensitivity (probability of the model to correctly predict a true presence) and specificity (probability of the model to correctly predict a true absence).

```
##     Species_name  AUC       TSS sensitivity specificity
## tp Larix decidua 0.83 0.5791411   0.9030675   0.6760736
```

Response curves and response maps

Response curves

Response curves (also known as effect curves) give an overview of the climatic niche of a species by relating the occurrence probability to corresponding climatic values. Predictor acronyms: Bio.10 = Mean temperature of warmest quarter [°C] within months 6 to 8, Bio.11 = Mean temperature of coldest quarter [°C] within months 12,1,2, Bio.12 = Annual precipitation sum [mm/m2], Bio.18 = Mean monthly precipitation amount of the warmest quarter [mm/m2] within months 6 to 8. Lines on the x-axis mark the upper and lower limit of the used presences (red), the mean (bold black) and the median (bold blue).

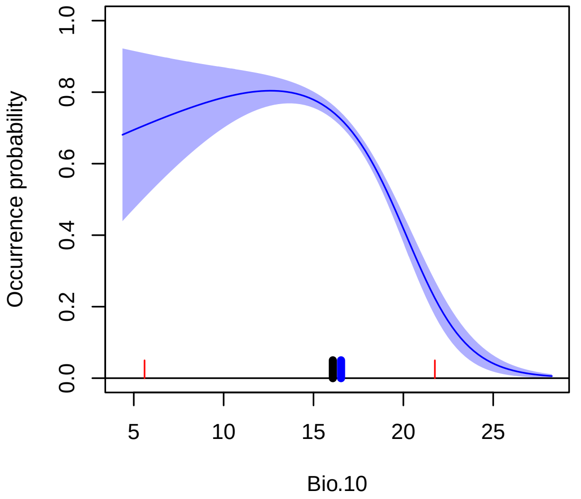

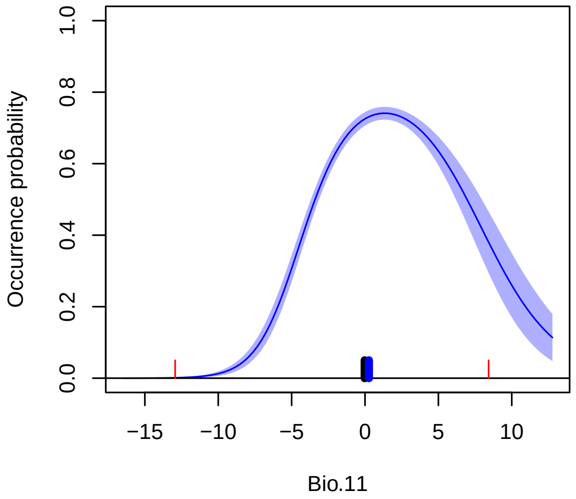

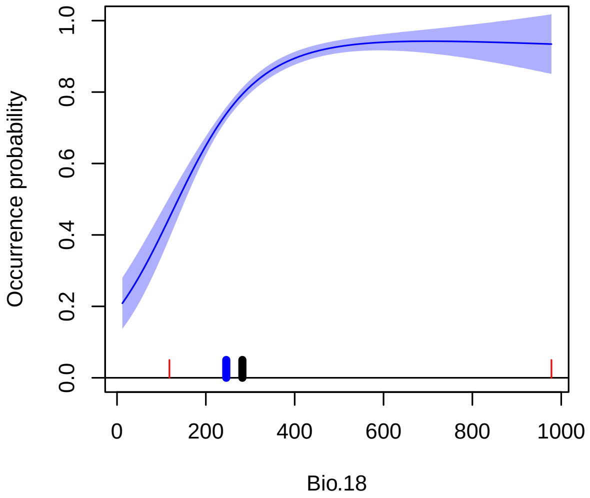

Response maps

Response maps (also referred to as partial effect maps). Each map represents how each predictor affects the occurrence probability. Predictor acronyms: Bio.10 = Mean temperature of warmest quarter [°C] within months 6 to 8, Bio.11 = Mean temperature of coldest quarter [°C] within months 12,1,2, Bio.12 = Annual precipitation sum [mm/m2], Bio.18 = Mean monthly precipitation amount of the warmest quarter [mm/m2] within months 6 to 8.

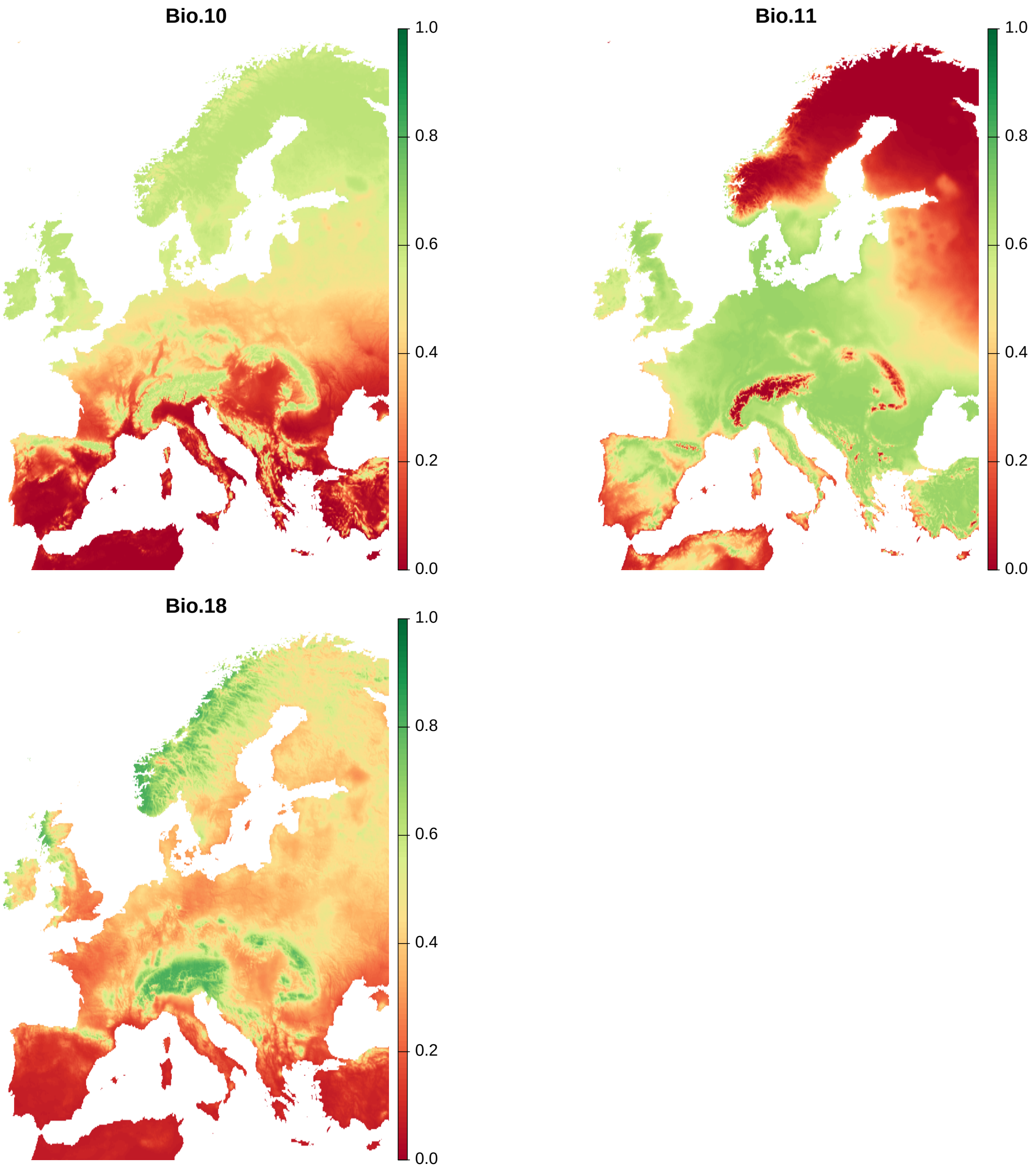

Model projections

Projection with plotted input data

Projection of species distribution model for reference period 1981-2010 over Europe. Occurrence probability ranges from 0 to 1 and is represented in dark red (low probability) to dark green (high probability). Input data used to calibrate the model is shown as presence points in magenta and absence points in black.

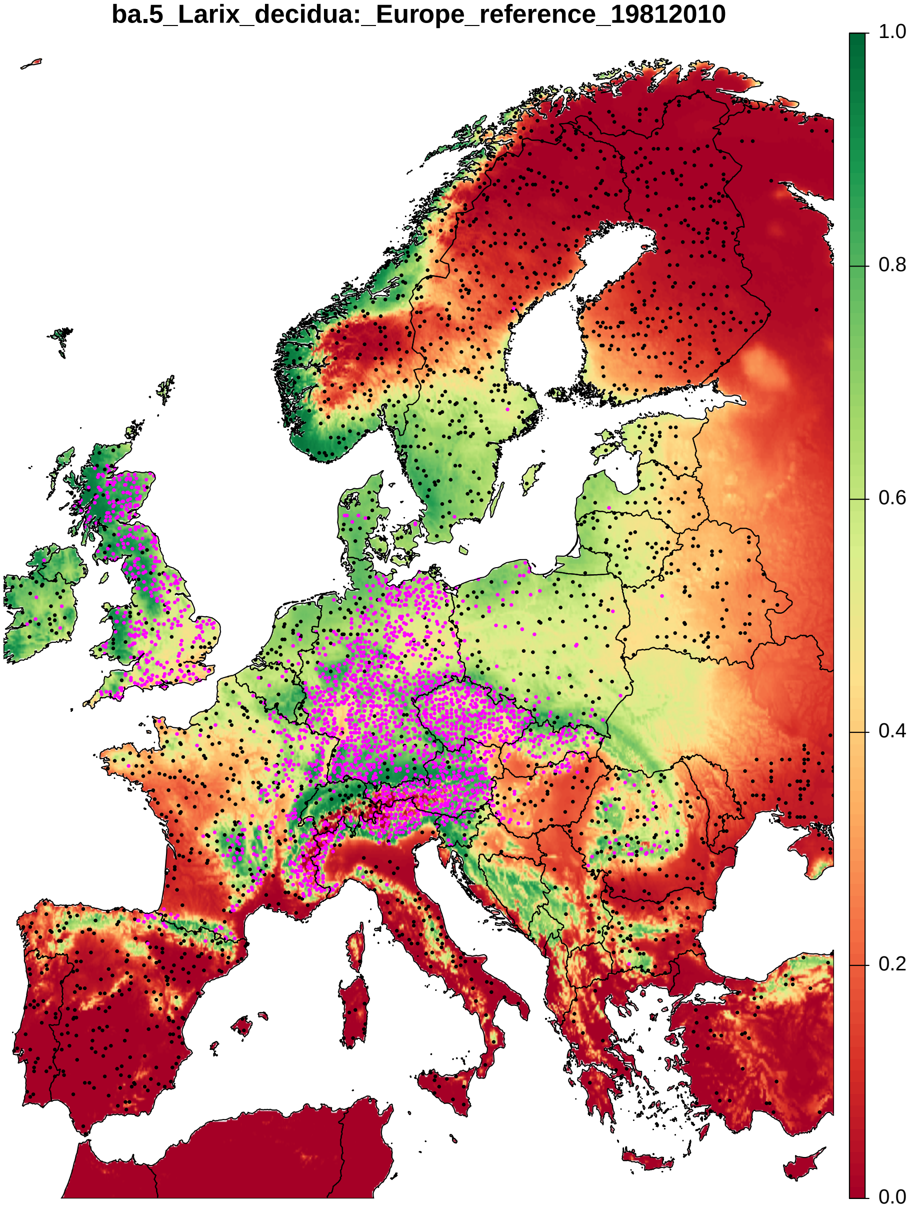

Projections

Projections of the species distribution models for reference period (1981-2010) and future scenarios RCP4.5 (2071-2100) and RCP8.5 (2071-2100) over Europe. Occurrence probabilities range from 0 to 1 and are represented from dark red (low probability) to dark green (high probability).

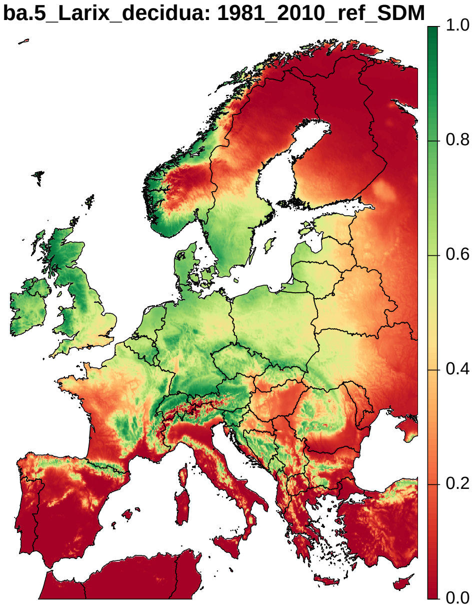

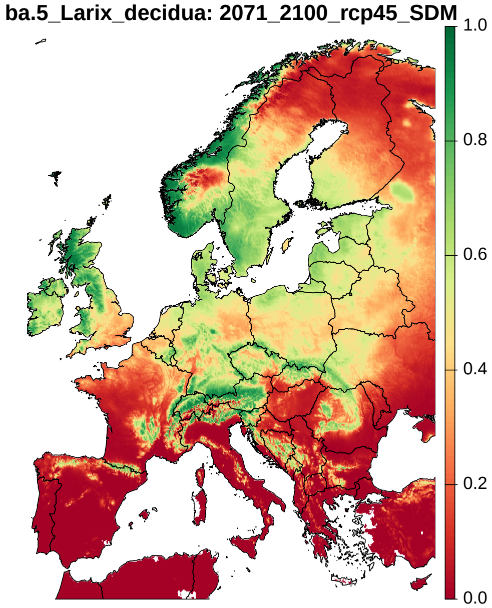

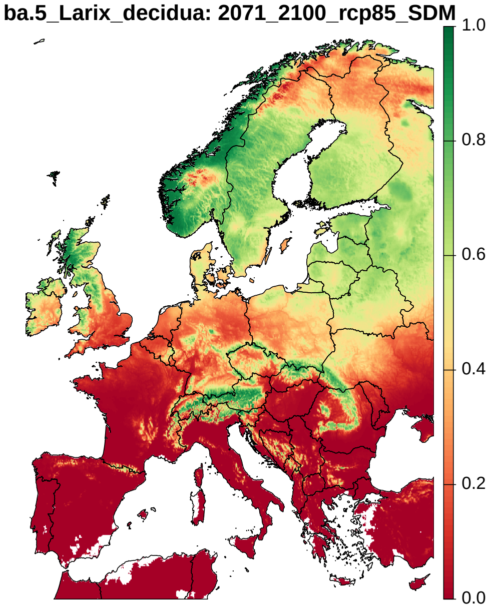

Climate risk classes

Maps of the climate risk classes. To estimate the distribution potential of each species as a mask for the SIMs, the continuous SDM outputs were categorized into three classes: low (yellow), medium (blue) and high climatic risk (red). The maps depict the risk classes in reference time (1981 to 2010), in climate scenario RCP4.5 (2071-2100) and RCP8.5 (2071-2100). To get an impression how well the thresholds fit to the data, presences (black) and absences (grey) were added on the reference map (top left). Refer to the legend and section “SDM thresholds” for the thresholds.

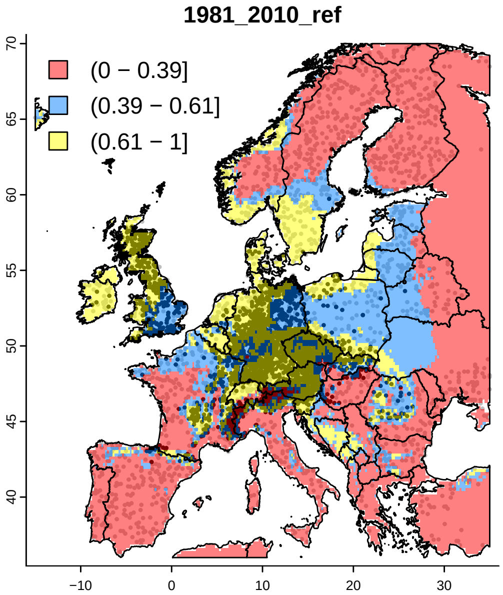

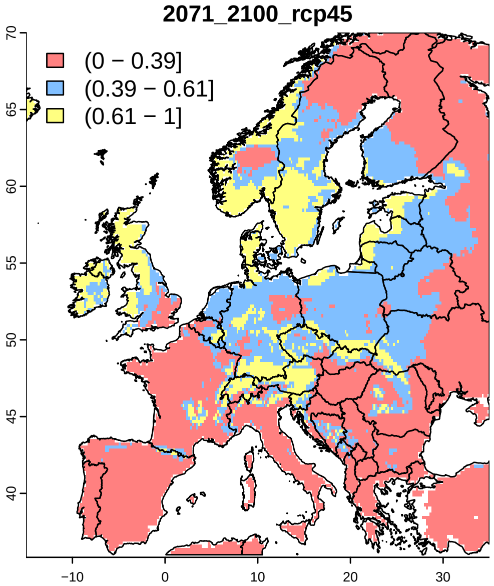

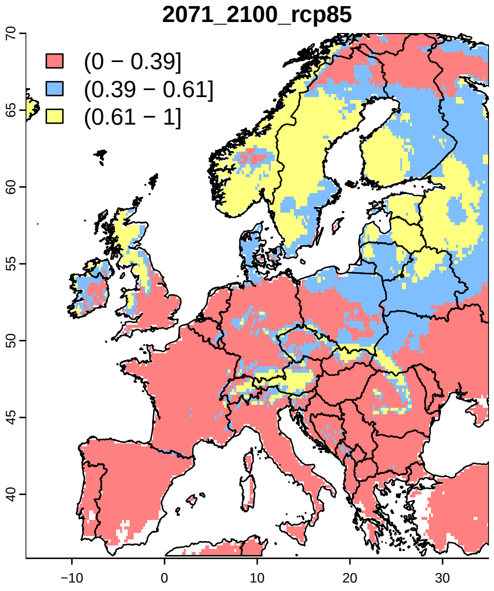

Picea abies

Model statistics and evaluation

Summary

Predictor acronyms: Bio.10 = Mean temperature of warmest quarter [°C] within months 6 to 8, Bio.11 = Mean temperature of coldest quarter [°C] within months 12,1,2, Bio.12 = Annual precipitation sum [mm/m2], Bio.18 = Mean monthly precipitation amount of the warmest quarter [mm/m2] within months 6 to 8.

```
##
## Family: binomial
## Link function: logit
##
## Formula:
## ba.1 ~ s(Bio.10, k = 3) + s(Bio.11, k = 3) + s(Bio.18, k = 3)
##
## Parametric coefficients:
##             Estimate Std. Error z value Pr(>|z|)
## (Intercept) -1.33133    0.03934  -33.84   <2e-16 ***
## ---
## Signif. codes:  0 '***' 0.001 '**' 0.01 '*' 0.05 '.' 0.1 ' ' 1
##
## Approximate significance of smooth terms:
##             edf Ref.df Chi.sq p-value
## s(Bio.10) 2.000      2 2703.5  <2e-16 ***
## s(Bio.11) 1.993      2  655.3  <2e-16 ***
## s(Bio.18) 1.996      2  648.2  <2e-16 ***
## ---
## Signif. codes:  0 '***' 0.001 '**' 0.01 '*' 0.05 '.' 0.1 ' ' 1
##
## R-sq.(adj) =   0.52   Deviance explained = 46.3%
## -REML = 7504.4  Scale est. = 1         n = 20094
```

Evaluation parameter

Model performance was assessed using four statistical parameters: the area under the receiver operating characteristic curve (AUC), the true skill statistic (TSS), sensitivity (probability of the model to correctly predict a true presence) and specificity (probability of the model to correctly predict a true absence).

```
##    Species_name  AUC       TSS sensitivity specificity
## tp  Picea abies 0.91 0.6534289   0.8988753   0.7545536
```

Response curves and response maps

Response curves

Response curves (also known as effect curves) give an overview of the climatic niche of a species by relating the occurrence probability to corresponding climatic values. Predictor acronyms: Bio.10 = Mean temperature of warmest quarter [°C] within months 6 to 8, Bio.11 = Mean temperature of coldest quarter [°C] within months 12,1,2, Bio.12 = Annual precipitation sum [mm/m2], Bio.18 = Mean monthly precipitation amount of the warmest quarter [mm/m2] within months 6 to 8. Lines on the x-axis mark the upper and lower limit of the used presences (red), the mean (bold black) and the median (bold blue).

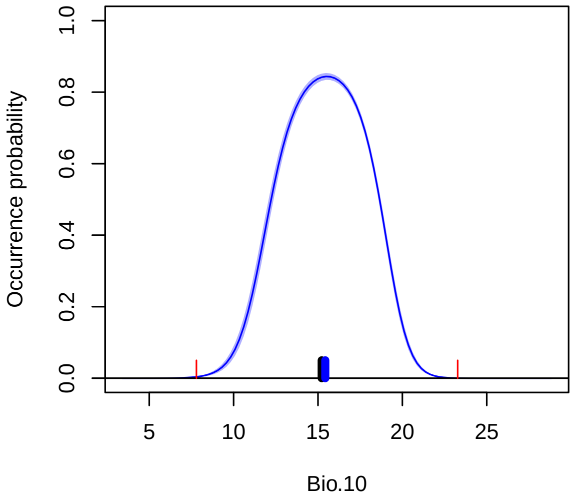

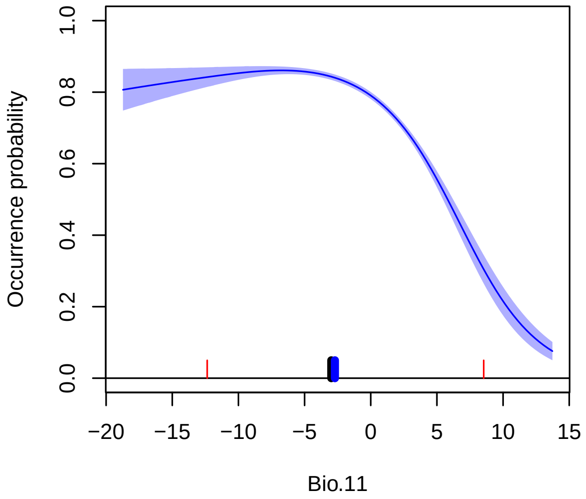

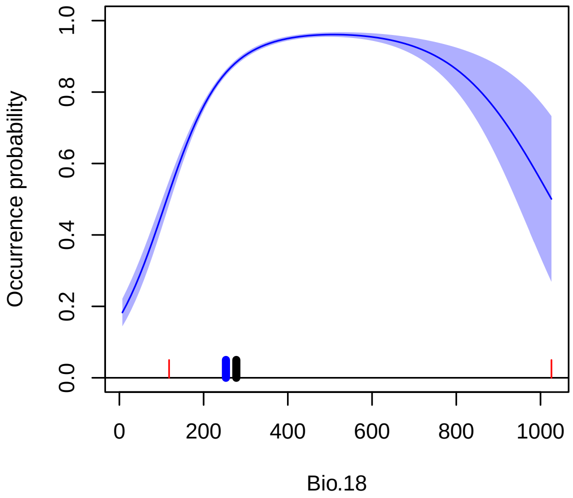

Response maps

Response maps (also referred to as partial effect maps). Each map represents how each predictor affects the occurrence probability. Predictor acronyms: Bio.10 = Mean temperature of warmest quarter [°C] within months 6 to 8, Bio.11 = Mean temperature of coldest quarter [°C] within months 12,1,2, Bio.12 = Annual precipitation sum [mm/m2], Bio.18 = Mean monthly precipitation amount of the warmest quarter [mm/m2] within months 6 to 8.

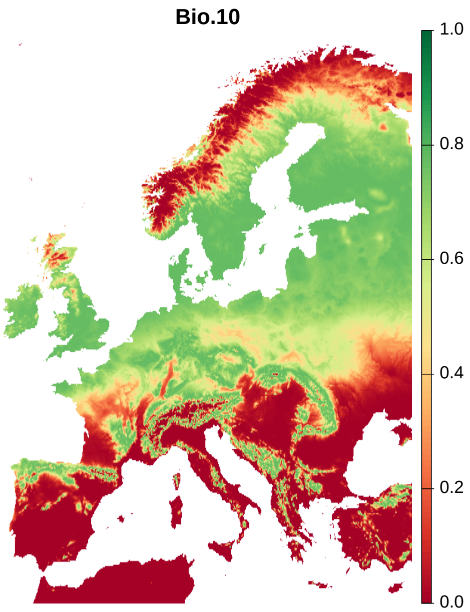

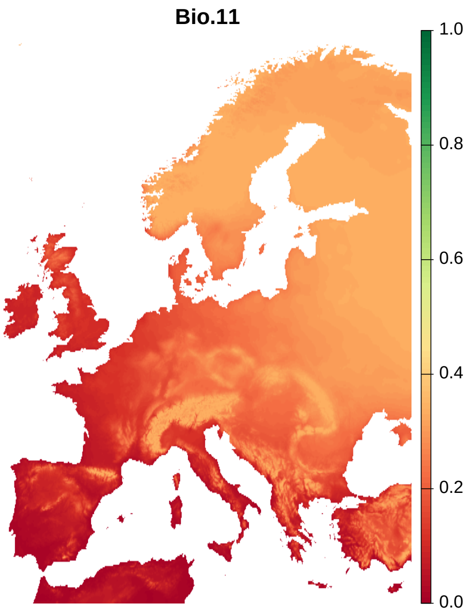

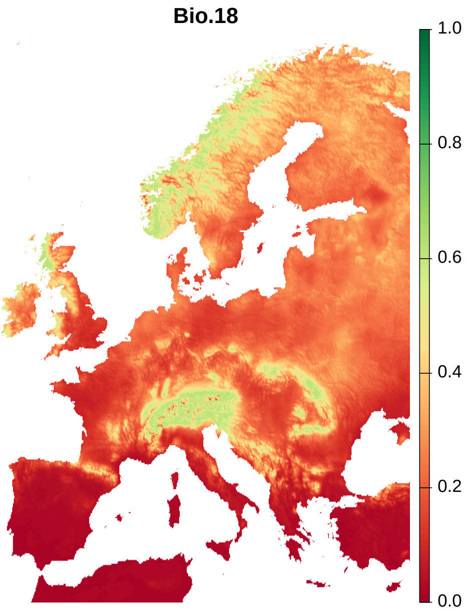

Model projections

Projection with plotted input data

Projection of species distribution model for reference period 1981-2010 over Europe. Occurrence probability ranges from 0 to 1 and is represented in dark red (low probability) to dark green (high probability). Input data used to calibrate the model is shown as presence points in magenta and absence points in black.

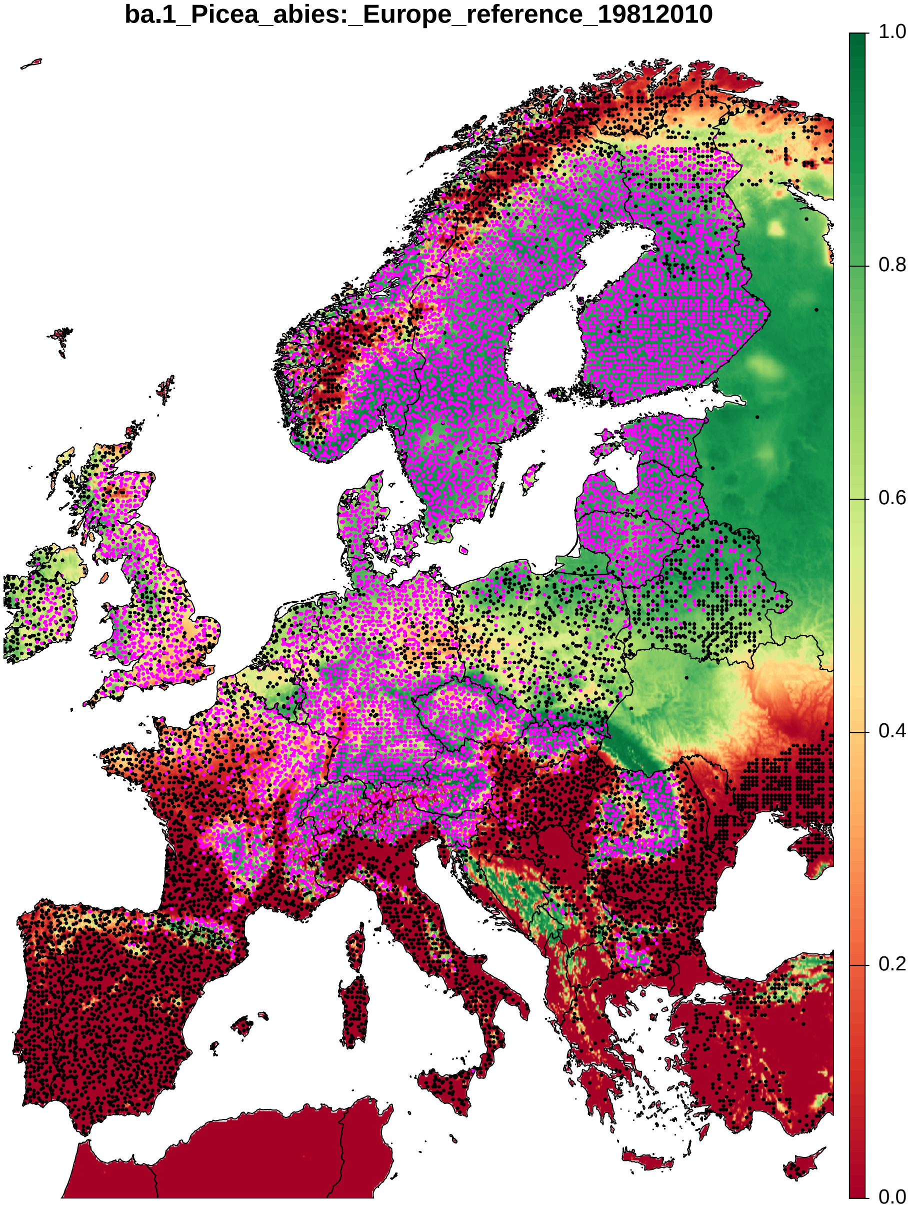

Projections

Projections of the species distribution models for reference period (1981-2010) and future scenarios RCP4.5 (2071-2100) and RCP8.5 (2071-2100) over Europe. Occurrence probabilities range from 0 to 1 and are represented from dark red (low probability) to dark green (high probability).

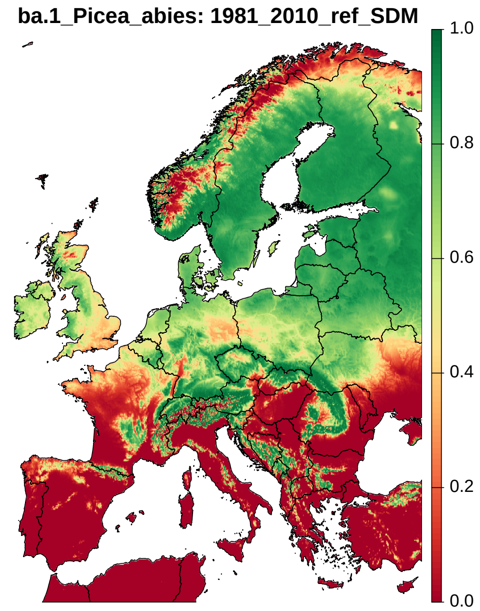

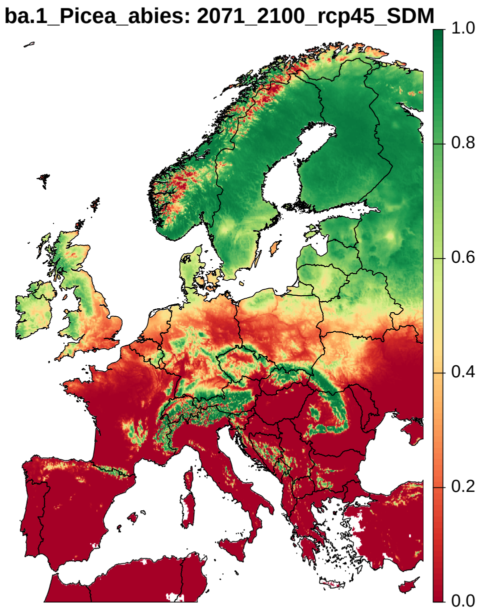

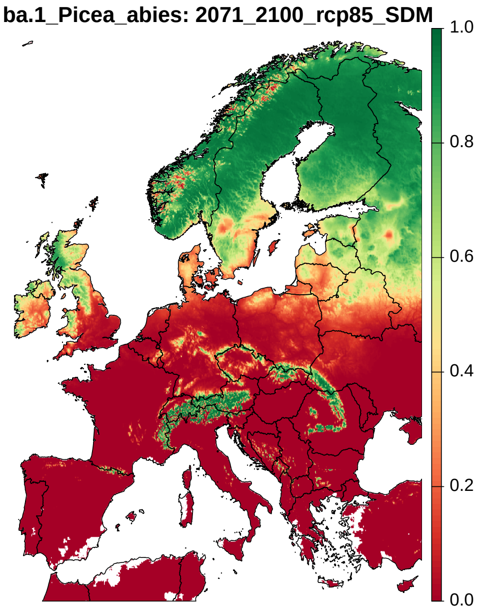

Climate risk classes

Maps of the climate risk classes. To estimate the distribution potential of each species as a mask for the SIMs, the continuous SDM outputs were categorized into three classes: low (yellow), medium (blue) and high climatic risk (red). The maps depict the risk classes in reference time (1981 to 2010), in climate scenario RCP4.5 (2071-2100) and RCP8.5 (2071-2100). To get an impression how well the thresholds fit to the data, presences (black) and absences (grey) were added on the reference map (top left). Refer to the legend and section “SDM thresholds” for the thresholds.

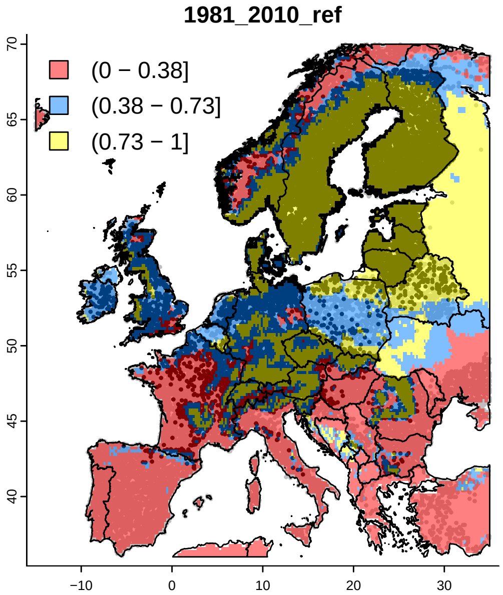

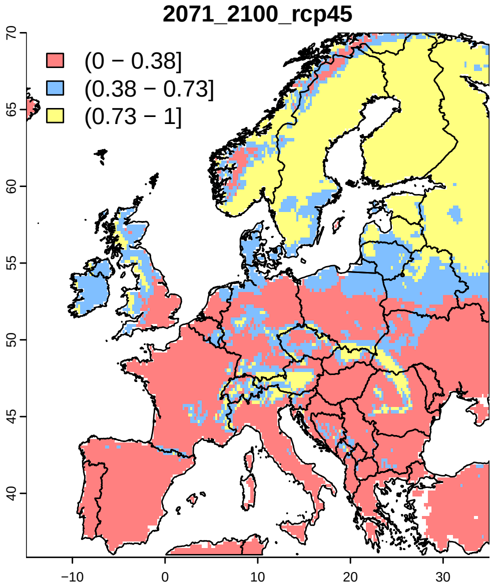

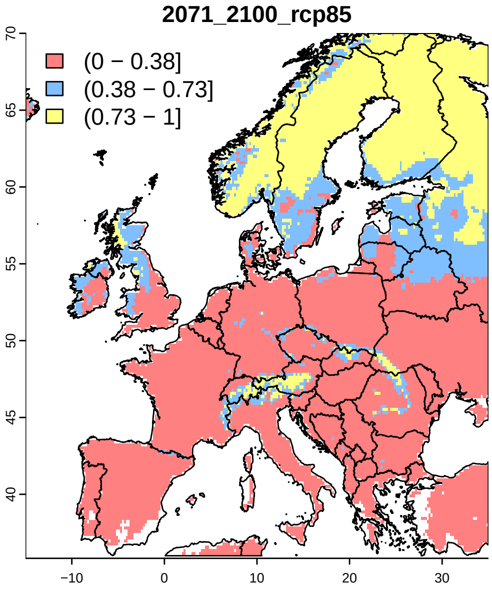

Pinus nigra

Model statistics and evaluation

Summary

Predictor acronyms: Bio.10 = Mean temperature of warmest quarter [°C] within months 6 to 8, Bio.11 = Mean temperature of coldest quarter [°C] within months 12,1,2, Bio.12 = Annual precipitation sum [mm/m2], Bio.18 = Mean monthly precipitation amount of the warmest quarter [mm/m2] within months 6 to 8.

```
##
## Family: binomial
## Link function: logit
##
## Formula:
## ba.4 ~ s(Bio.10, k = 3) + s(Bio.11, k = 3) + s(Bio.12, k = 3)
##
## Parametric coefficients:
##             Estimate Std. Error z value Pr(>|z|)
## (Intercept) -1.57010    0.09676  -16.23   <2e-16 ***
## ---
## Signif. codes:  0 '***' 0.001 '**' 0.01 '*' 0.05 '.' 0.1 ' ' 1
##
## Approximate significance of smooth terms:
##             edf Ref.df Chi.sq p-value
## s(Bio.10) 1.995  2.000 242.06  <2e-16 ***
## s(Bio.11) 1.996  2.000 288.52  <2e-16 ***
## s(Bio.12) 1.958  1.998  63.47  <2e-16 ***
## ---
## Signif. codes:  0 '***' 0.001 '**' 0.01 '*' 0.05 '.' 0.1 ' ' 1
##
## R-sq.(adj) =  0.436   Deviance explained = 36.3%
## -REML =   1751  Scale est. = 1         n = 3928
```

Evaluation parameter

Model performance was assessed using four statistical parameters: the area under the receiver operating characteristic curve (AUC), the true skill statistic (TSS), sensitivity (probability of the model to correctly predict a true presence) and specificity (probability of the model to correctly predict a true absence).

```
##    Species_name  AUC       TSS sensitivity specificity
## tp  Pinus nigra 0.87 0.6171079   0.9022403   0.7148676
```

Response curves and response maps

Response curves

Response curves (also known as effect curves) give an overview of the climatic niche of a species by relating the occurrence probability to corresponding climatic values. Predictor acronyms: Bio.10 = Mean temperature of warmest quarter [°C] within months 6 to 8, Bio.11 = Mean temperature of coldest quarter [°C] within months 12,1,2, Bio.12 = Annual precipitation sum [mm/m2], Bio.18 = Mean monthly precipitation amount of the warmest quarter [mm/m2] within months 6 to 8. Lines on the x-axis mark the upper and lower limit of the used presences (red), the mean (bold black) and the median (bold blue).

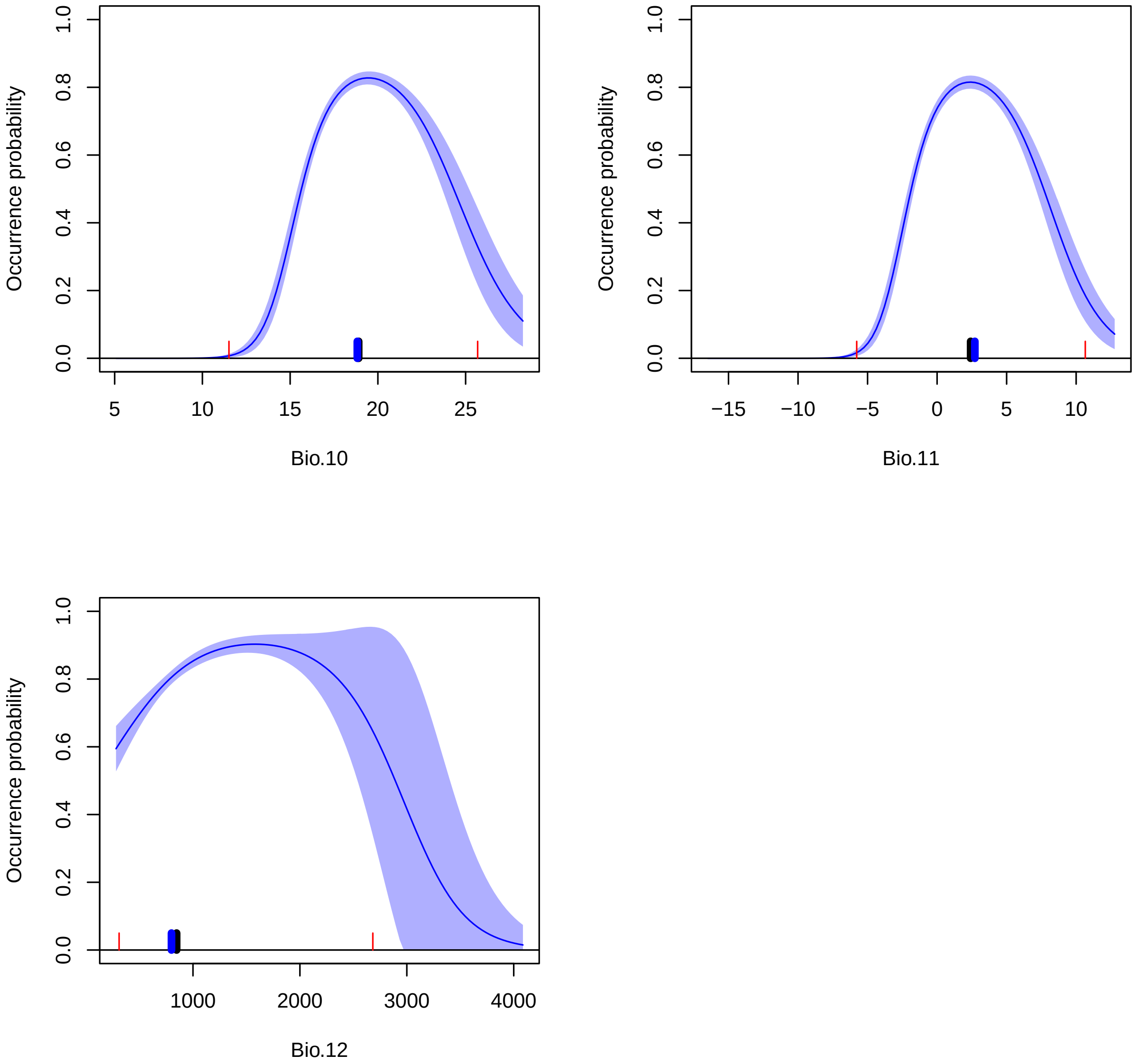

Response maps

Response maps (also referred to as partial effect maps). Each map represents how each predictor affects the occurrence probability. Predictor acronyms: Bio.10 = Mean temperature of warmest quarter [°C] within months 6 to 8, Bio.11 = Mean temperature of coldest quarter [°C] within months 12,1,2, Bio.12 = Annual precipitation sum [mm/m2], Bio.18 = Mean monthly precipitation amount of the warmest quarter [mm/m2] within months 6 to 8.

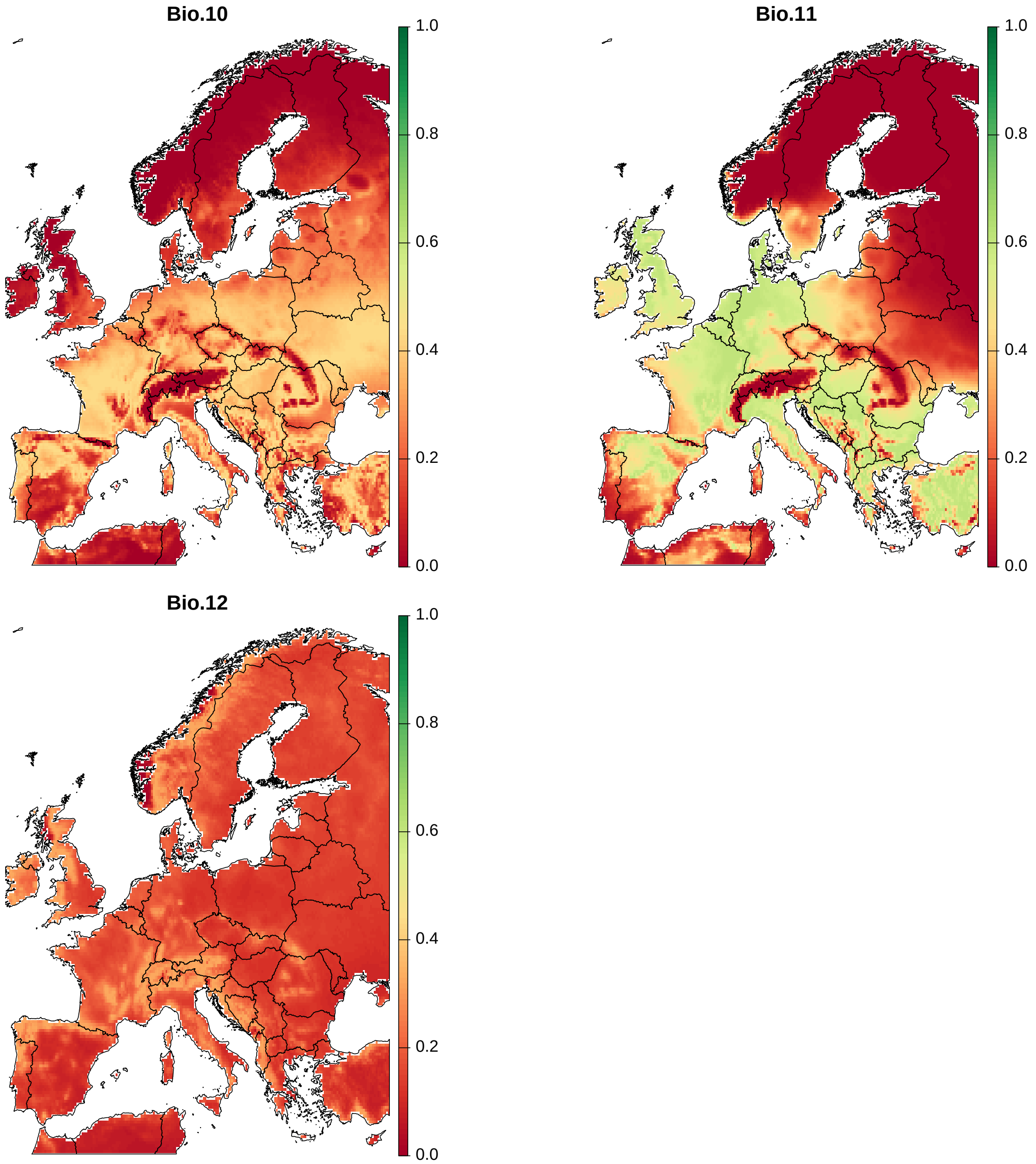

Model projections

Projection with plotted input data

Projection of species distribution model for reference period 1981-2010 over Europe. Occurrence probability ranges from 0 to 1 and is represented in dark red (low probability) to dark green (high probability). Input data used to calibrate the model is shown as presence points in magenta and absence points in black.

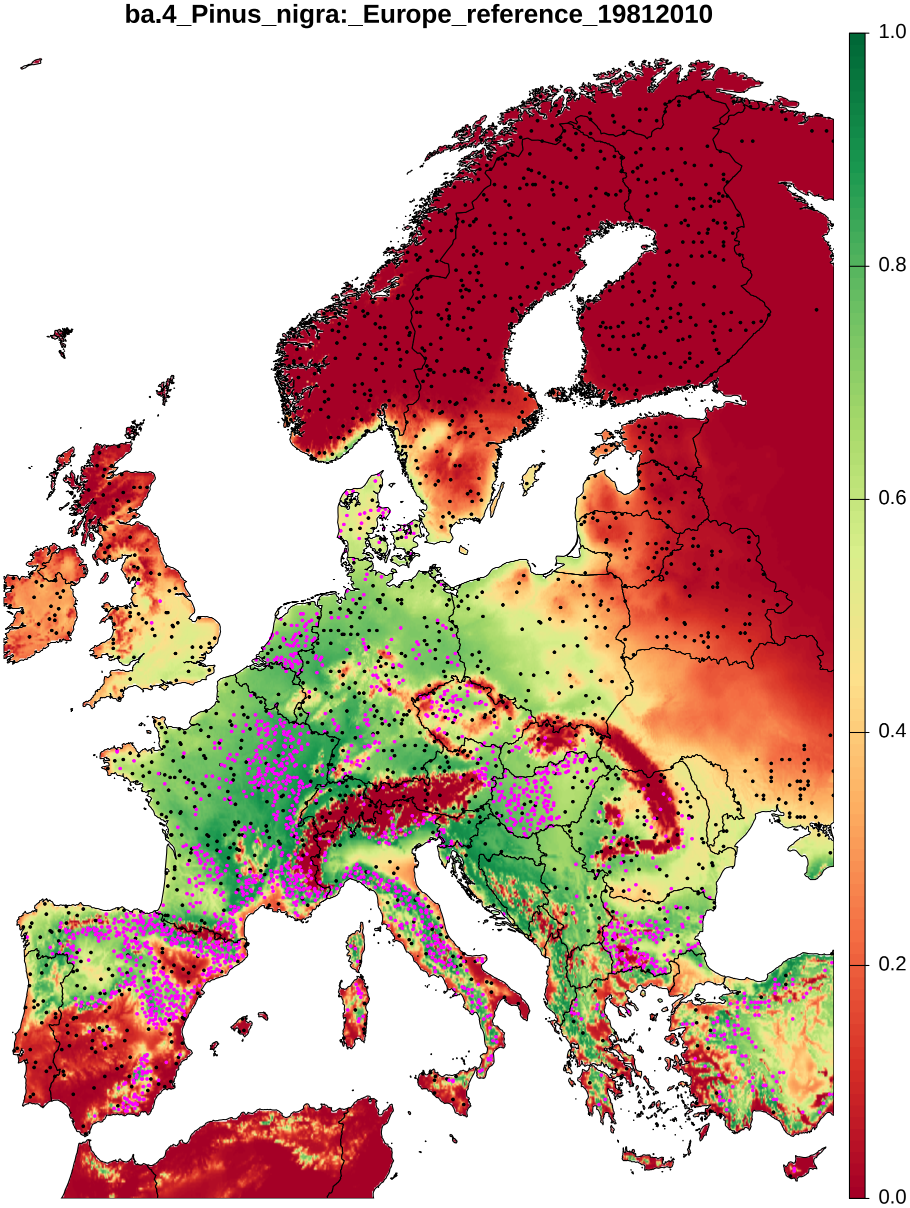

Projections

Projections of the species distribution models for reference period (1981-2010) and future scenarios RCP4.5 (2071-2100) and RCP8.5 (2071-2100) over Europe. Occurrence probabilities range from 0 to 1 and are represented from dark red (low probability) to dark green (high probability).

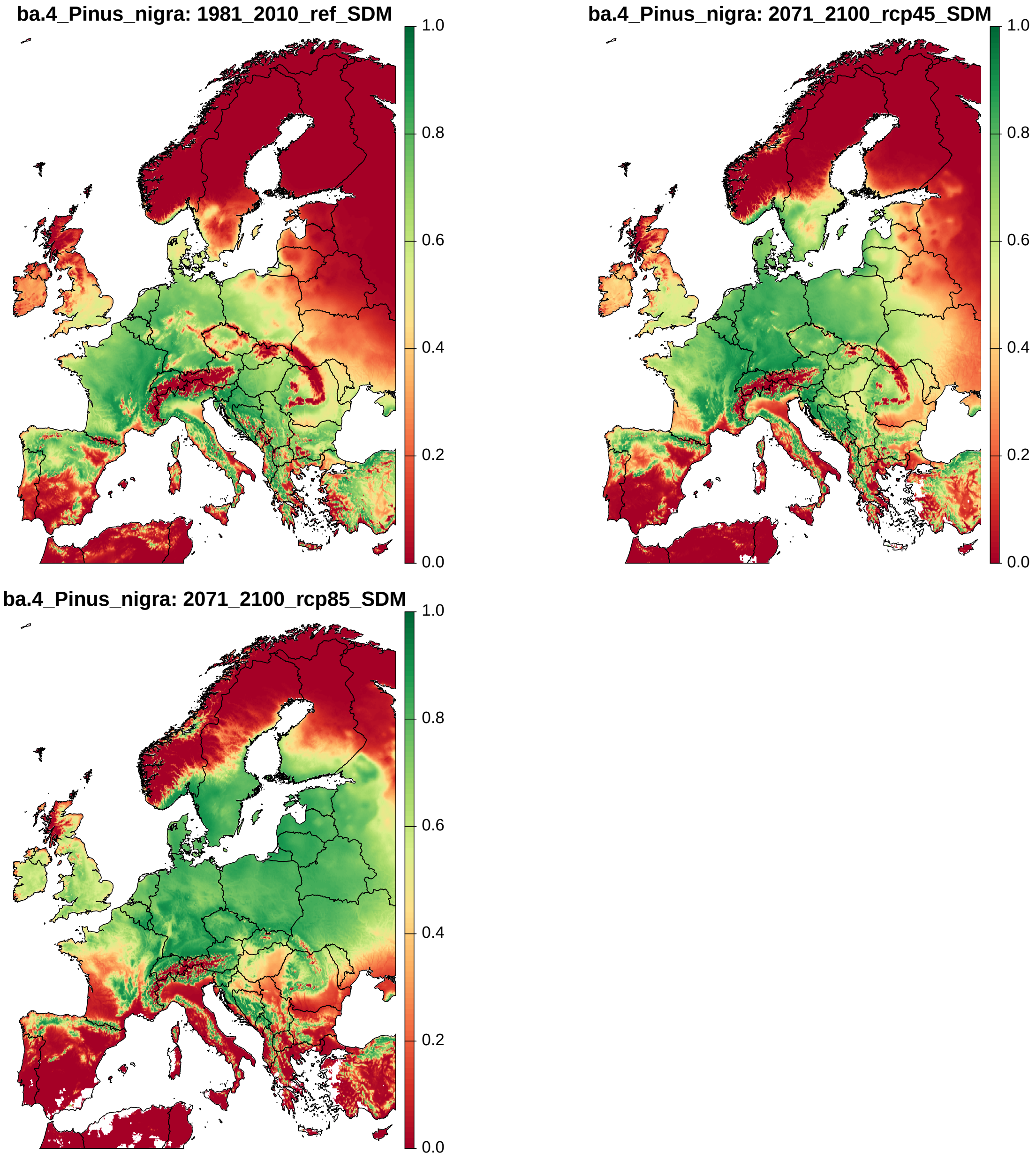

Climate risk classes

Maps of the climate risk classes. To estimate the distribution potential of each species as a mask for the SIMs, the continuous SDM outputs were categorized into three classes: low (yellow), medium (blue) and high climatic risk (red). The maps depict the risk classes in reference time (1981 to 2010), in climate scenario RCP4.5 (2071-2100) and RCP8.5 (2071-2100). To get an impression how well the thresholds fit to the data, presences (black) and absences (grey) were added on the reference map (top left). Refer to the legend and section "SDM thresholds" for the thresholds.

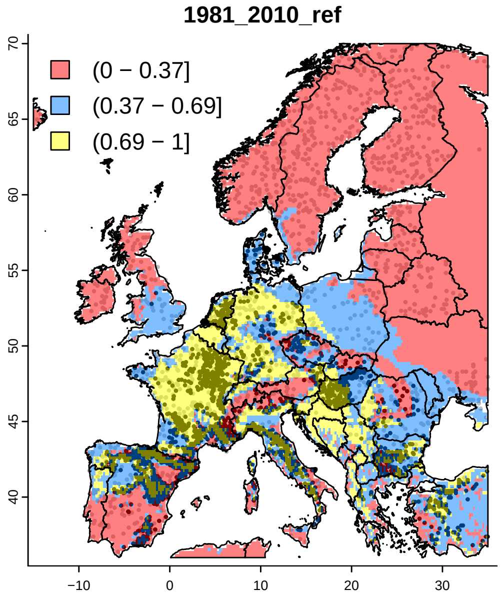

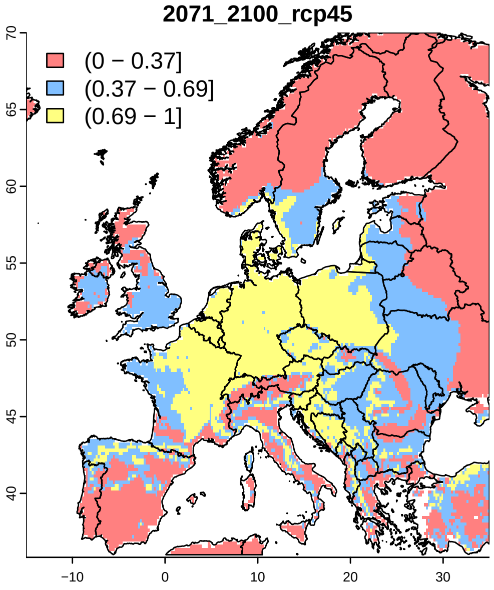

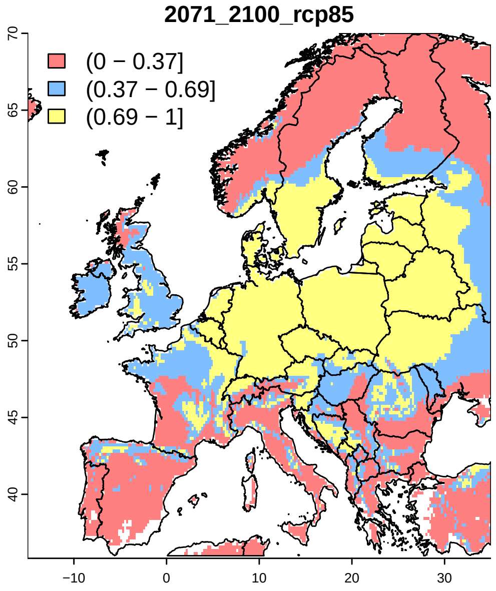

Pinus sylvestris

Model statistics and evaluation

Summary

Predictor acronyms: Bio.10 = Mean temperature of warmest quarter [°C] within months 6 to 8, Bio.11 = Mean temperature of coldest quarter [°C] within months 12,1,2, Bio.12 = Annual precipitation sum [mm/m2], Bio.18 = Mean monthly precipitation amount of the warmest quarter [mm/m2] within months 6 to 8.

```
##
## Family: binomial
## Link function: logit
##
## Formula:
## ba.3 ~ s(Bio.10, k = 3) + s(Bio.11, k = 3) + s(Bio.18, k = 3)
##
## Parametric coefficients:
##             Estimate Std. Error z value Pr(>|z|)
## (Intercept) -0.38991    0.02147  -18.16   <2e-16 ***
## ---
## Signif. codes:  0 '***' 0.001 '**' 0.01 '*' 0.05 '.' 0.1 ' ' 1
##
## Approximate significance of smooth terms:
##             edf Ref.df  Chi.sq p-value
## s(Bio.10) 2.000      2 2739.10  <2e-16 ***
## s(Bio.11) 1.988      2   85.09  <2e-16 ***
## s(Bio.18) 1.990      2   96.29  <2e-16 ***
## ---
## Signif. codes:  0 '***' 0.001 '**' 0.01 '*' 0.05 '.' 0.1 ' ' 1
##
## R-sq.(adj) =   0.34   Deviance explained = 28.5%
## -REML = 9589.1  Scale est. = 1         n = 19310
```

Evaluation parameter

Model performance was assessed using four statistical parameters: the area under the receiver operating characteristic curve (AUC), the true skill statistic (TSS), sensitivity (probability of the model to correctly predict a true presence) and specificity (probability of the model to correctly predict a true absence).

```
##        Species_name  AUC       TSS sensitivity specificity
## tp Pinus sylvestris 0.82 0.5245987   0.8524081   0.6721906
```

Response curves and response maps

Response curves

Response curves (also known as effect curves) give an overview of the climatic niche of a species by relating the occurrence probability to corresponding climatic values. Predictor acronyms: Bio.10 = Mean temperature of warmest quarter [°C] within months 6 to 8, Bio.11 = Mean temperature of coldest quarter [°C] within months 12,1,2, Bio.12 = Annual precipitation sum [mm/m2], Bio.18 = Mean monthly precipitation amount of the warmest quarter [mm/m2] within months 6 to 8. Lines on the x-axis mark the upper and lower limit of the used presences (red), the mean (bold black) and the median (bold blue).

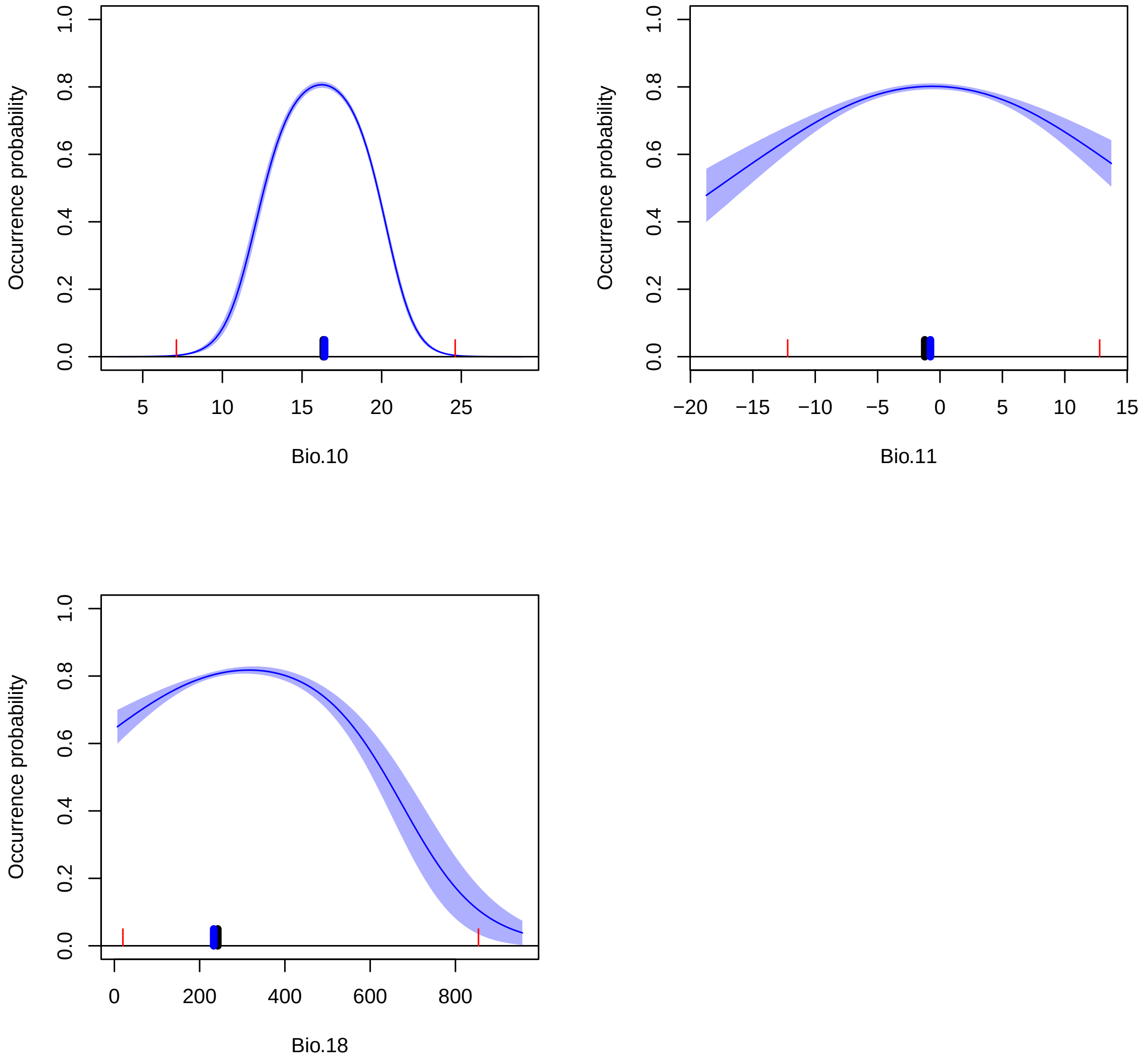

Response maps

Response maps (also referred to as partial effect maps). Each map represents how each predictor affects the occurrence probability. Predictor acronyms: Bio.10 = Mean temperature of warmest quarter [°C] within months 6 to 8, Bio.11 = Mean temperature of coldest quarter [°C] within months 12,1,2, Bio.12 = Annual precipitation sum [mm/m2], Bio.18 = Mean monthly precipitation amount of the warmest quarter [mm/m2] within months 6 to 8.

Bio.10

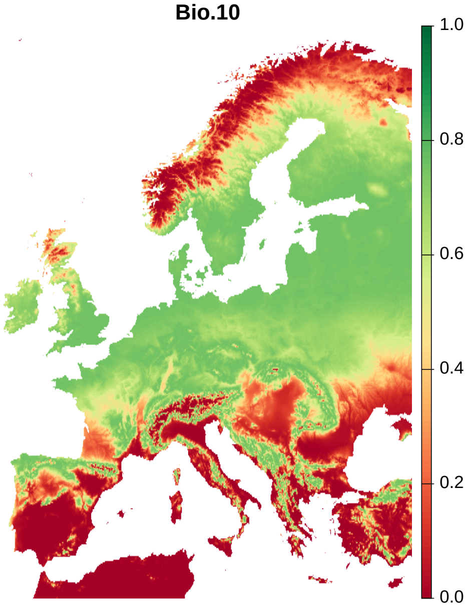

Bio.11

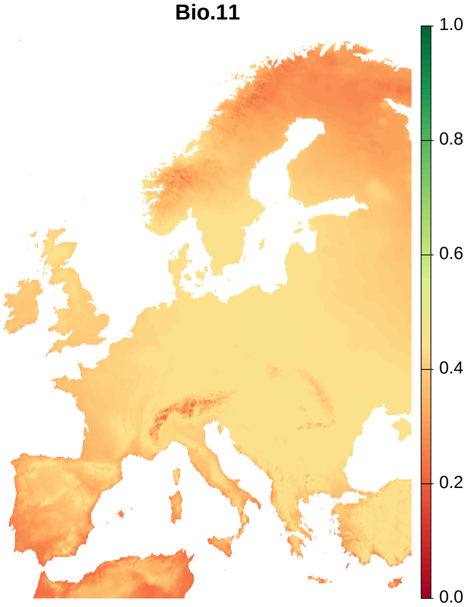

Bio.18

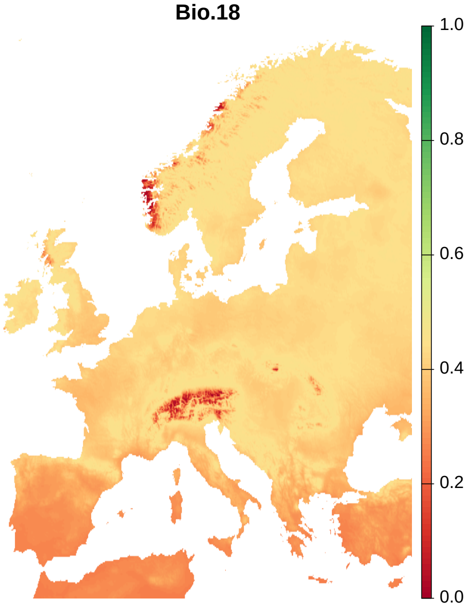

Model projections

Projection with plotted input data

Projection of species distribution model for reference period 1981-2010 over Europe. Occurrence probability ranges from 0 to 1 and is represented in dark red (low probability) to dark green (high probability). Input data used to calibrate the model is shown as presence points in magenta and absence points in black.

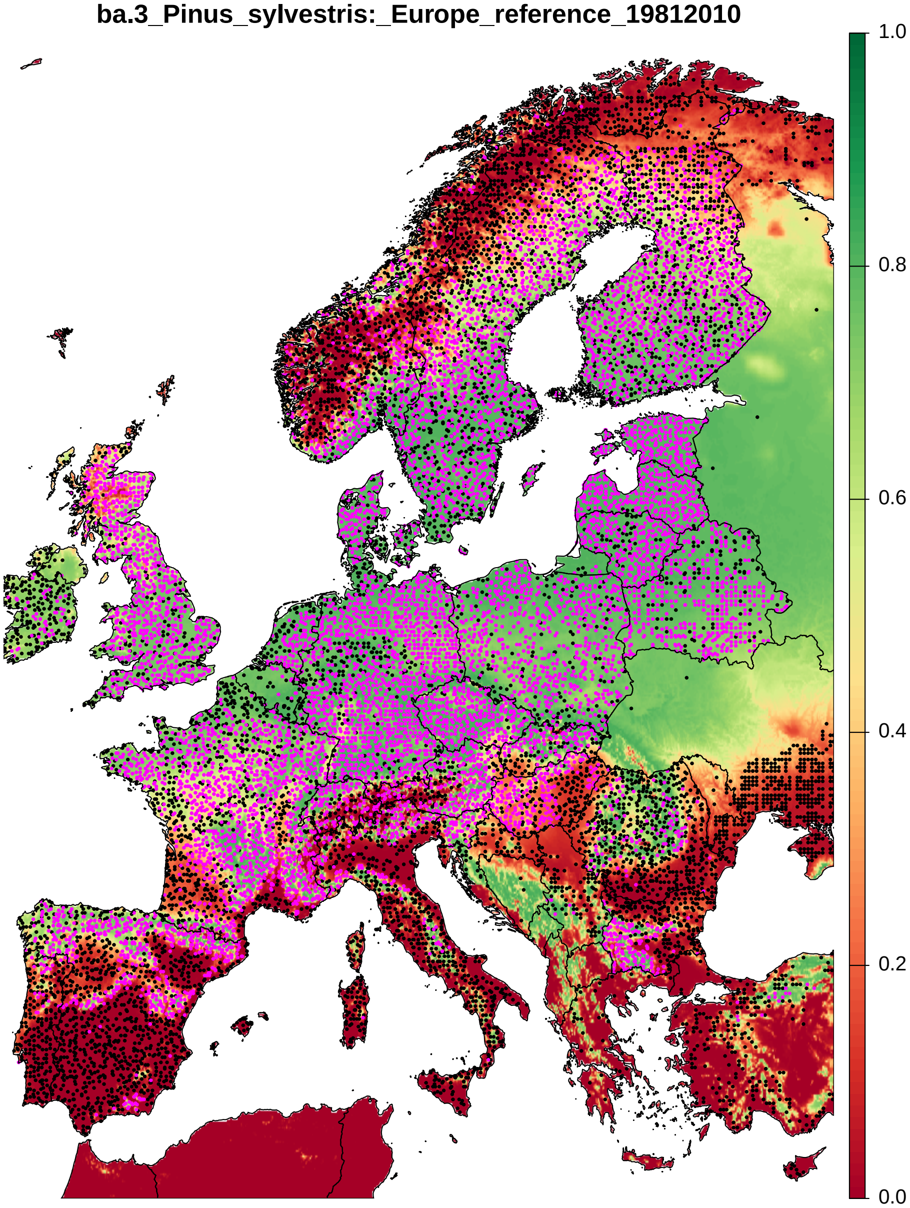

Projections

Projections of the species distribution models for reference period (1981-2010) and future scenarios RCP4.5 (2071-2100) and RCP8.5 (2071-2100) over Europe. Occurrence probabilities range from 0 to 1 and are represented from dark red (low probability) to dark green (high probability).

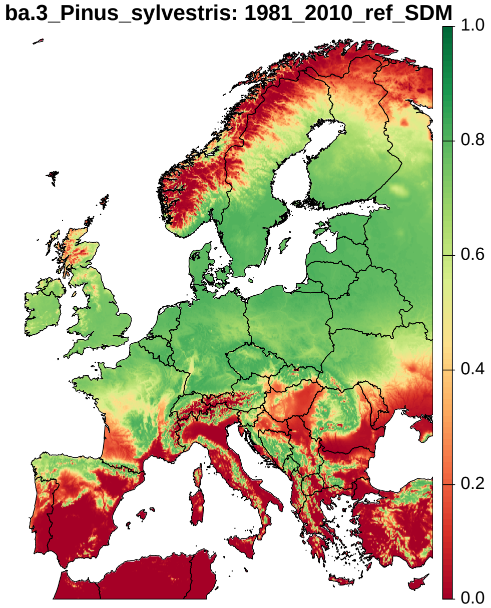

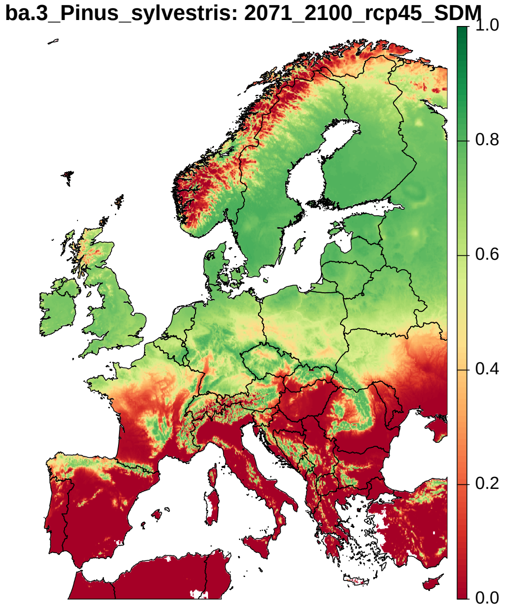

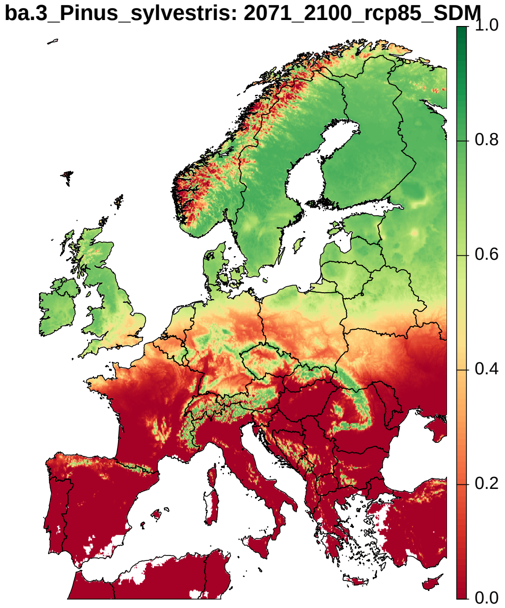

Climate risk classes

Maps of the climate risk classes. To estimate the distribution potential of each species as a mask for the SIMs, the continuous SDM outputs were categorized into three classes: low (yellow), medium (blue) and high climatic risk (red). The maps depict the risk classes in reference time (1981 to 2010), in climate scenario RCP4.5 (2071-2100) and RCP8.5 (2071-2100). To get an impression how well the thresholds fit to the data, presences (black) and absences (grey) were added on the reference map (top left). Refer to the legend and section “SDM thresholds” for the thresholds.

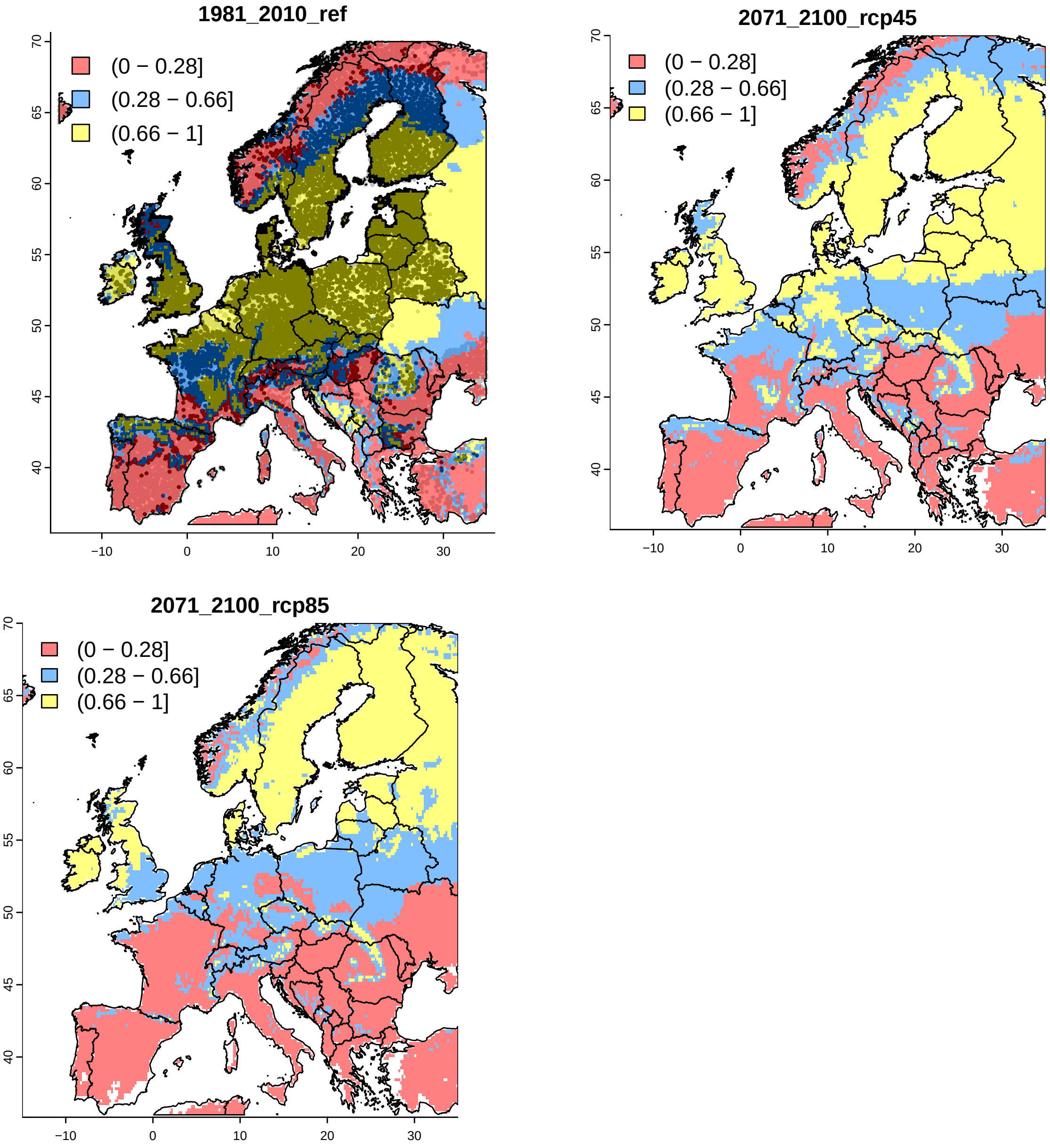

Prunus avium

Model statistics and evaluation

Summary

Predictor acronyms: Bio.10 = Mean temperature of warmest quarter [°C] within months 6 to 8, Bio.11 = Mean temperature of coldest quarter [°C] within months 12,1,2, Bio.12 = Annual precipitation sum [mm/m2], Bio.18 = Mean monthly precipitation amount of the warmest quarter [mm/m2] within months 6 to 8.

```
##
## Family: binomial
## Link function: logit
##
## Formula:
## ba.30 ~ s(Bio.10, k = 3) + s(Bio.11, k = 3) + s(Bio.18, k = 3)
##
## Parametric coefficients:
##             Estimate Std. Error z value Pr(>|z|)
## (Intercept) -1.22610    0.06217  -19.72   <2e-16 ***
## ---
## Signif. codes:  0 '***' 0.001 '**' 0.01 '*' 0.05 '.' 0.1 ' ' 1
##
## Approximate significance of smooth terms:
##             edf Ref.df Chi.sq p-value
## s(Bio.10) 1.998      2  645.9  <2e-16 ***
## s(Bio.11) 1.996      2  775.6  <2e-16 ***
## s(Bio.18) 1.988      2  482.9  <2e-16 ***
## ---
## Signif. codes:  0 '***' 0.001 '**' 0.01 '*' 0.05 '.' 0.1 ' ' 1
##
## R-sq.(adj) =  0.477   Deviance explained = 40.8%
## -REML = 3149.7  Scale est. = 1         n = 7636
```

Evaluation parameter

Model performance was assessed using four statistical parameters: the area under the receiver operating characteristic curve (AUC), the true skill statistic (TSS), sensitivity (probability of the model to correctly predict a true presence) and specificity (probability of the model to correctly predict a true absence).

```
##    Species_name  AUC       TSS sensitivity specificity
## tp Prunus avium 0.89 0.6346255   0.8962808   0.7383447
```

Response curves and response maps

Response curves

Response curves (also known as effect curves) give an overview of the climatic niche of a species by relating the occurrence probability to corresponding climatic values. Predictor acronyms: Bio.10 = Mean temperature of warmest quarter [°C] within months 6 to 8, Bio.11 = Mean temperature of coldest quarter [°C] within months 12,1,2, Bio.12 = Annual precipitation sum [mm/m2], Bio.18 = Mean monthly precipitation amount of the warmest quarter [mm/m2] within months 6 to 8. Lines on the x-axis mark the upper and lower limit of the used presences (red), the mean (bold black) and the median (bold blue).

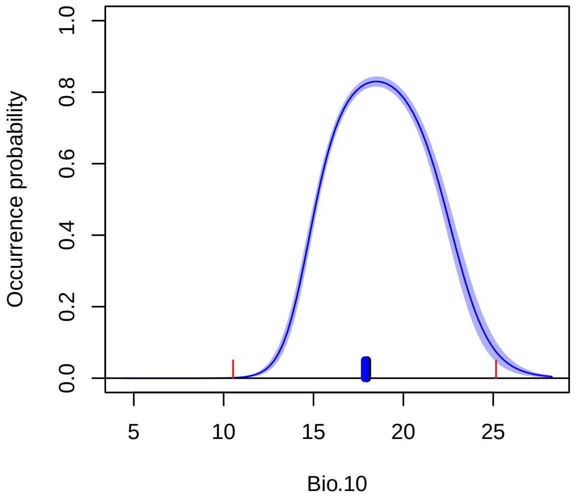

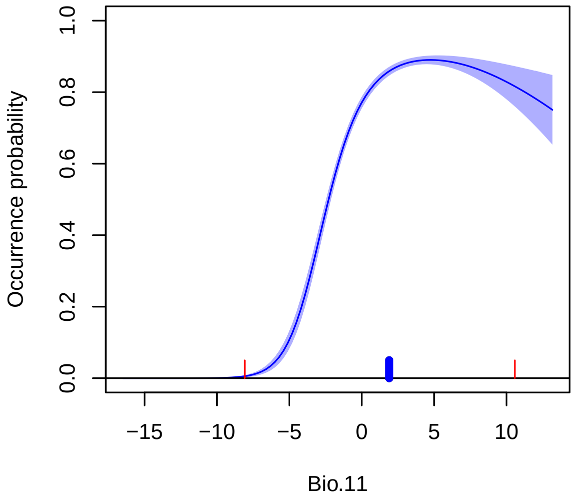

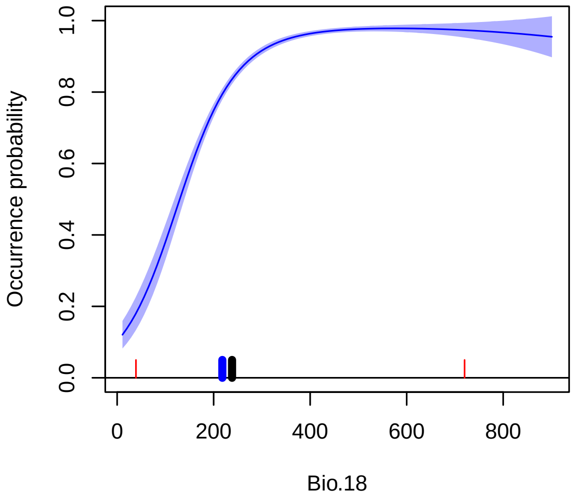

Response maps

Response maps (also referred to as partial effect maps). Each map represents how each predictor affects the occurrence probability. Predictor acronyms: Bio.10 = Mean temperature of warmest quarter [°C] within months 6 to 8, Bio.11 = Mean temperature of coldest quarter [°C] within months 12,1,2, Bio.12 = Annual precipitation sum [mm/m2], Bio.18 = Mean monthly precipitation amount of the warmest quarter [mm/m2] within months 6 to 8.

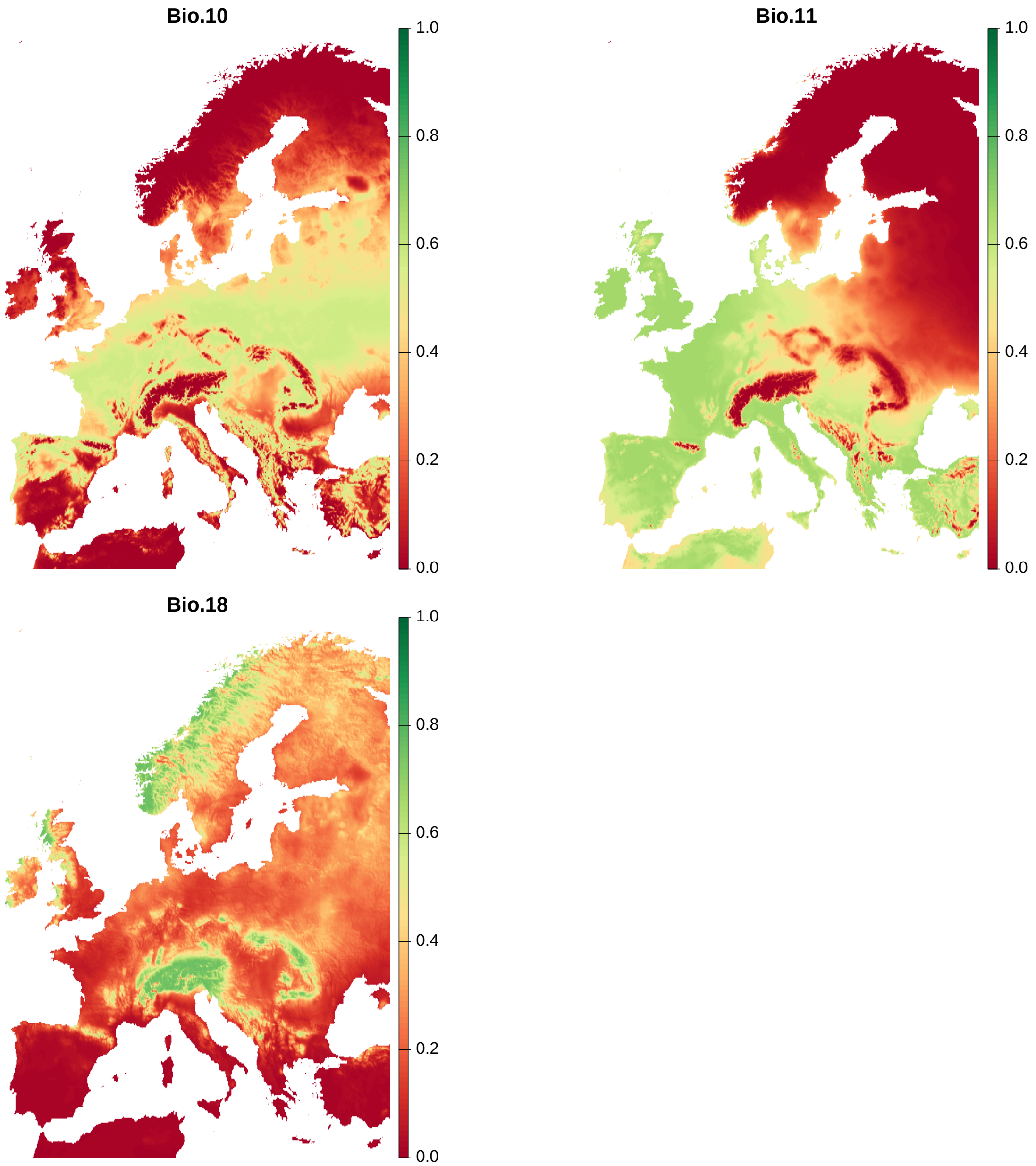

Model projections

Projection with plotted input data

Projection of species distribution model for reference period 1981-2010 over Europe. Occurrence probability ranges from 0 to 1 and is represented in dark red (low probability) to dark green (high probability). Input data used to calibrate the model is shown as presence points in magenta and absence points in black.

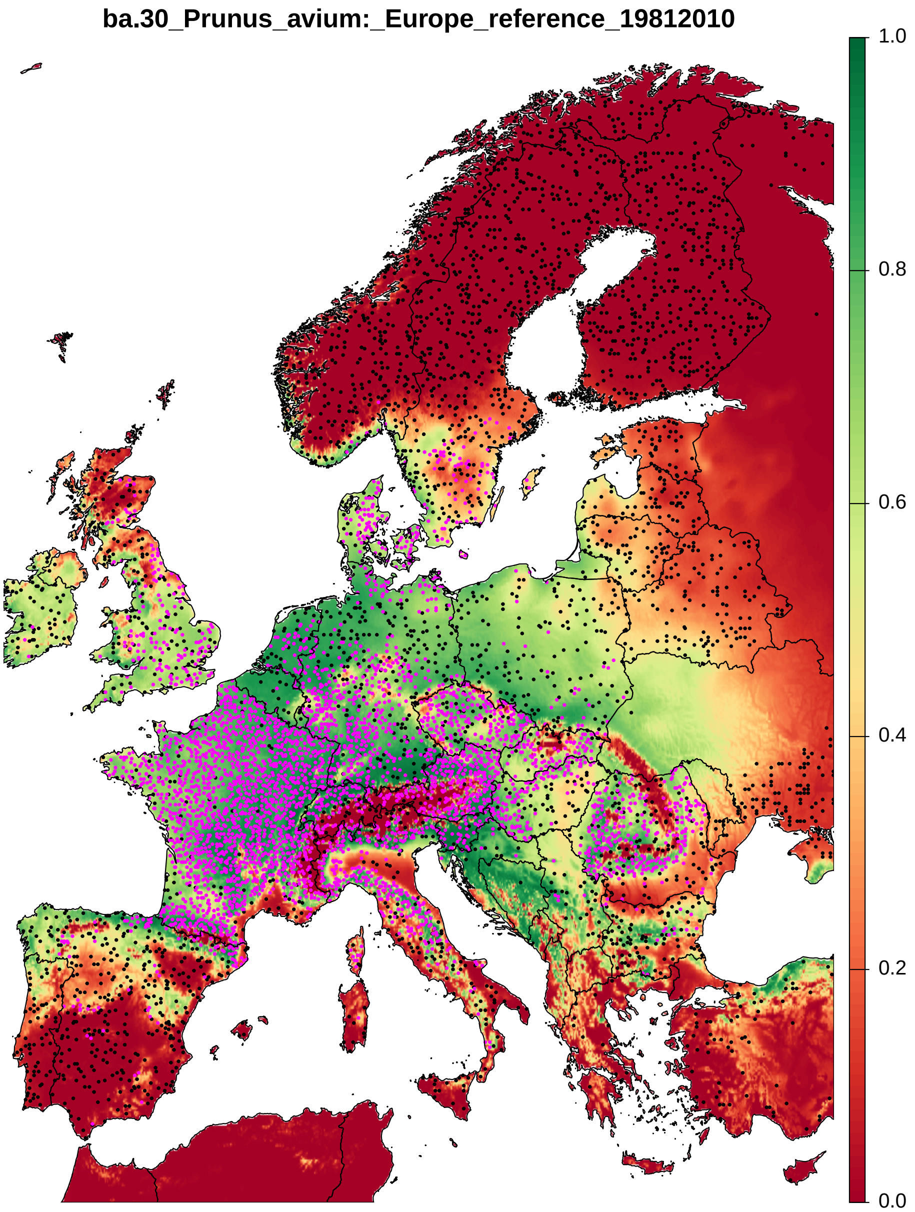

Projections

Projections of the species distribution models for reference period (1981-2010) and future scenarios RCP4.5 (2071-2100) and RCP8.5 (2071-2100) over Europe. Occurrence probabilities range from 0 to 1 and are represented from dark red (low probability) to dark green (high probability).

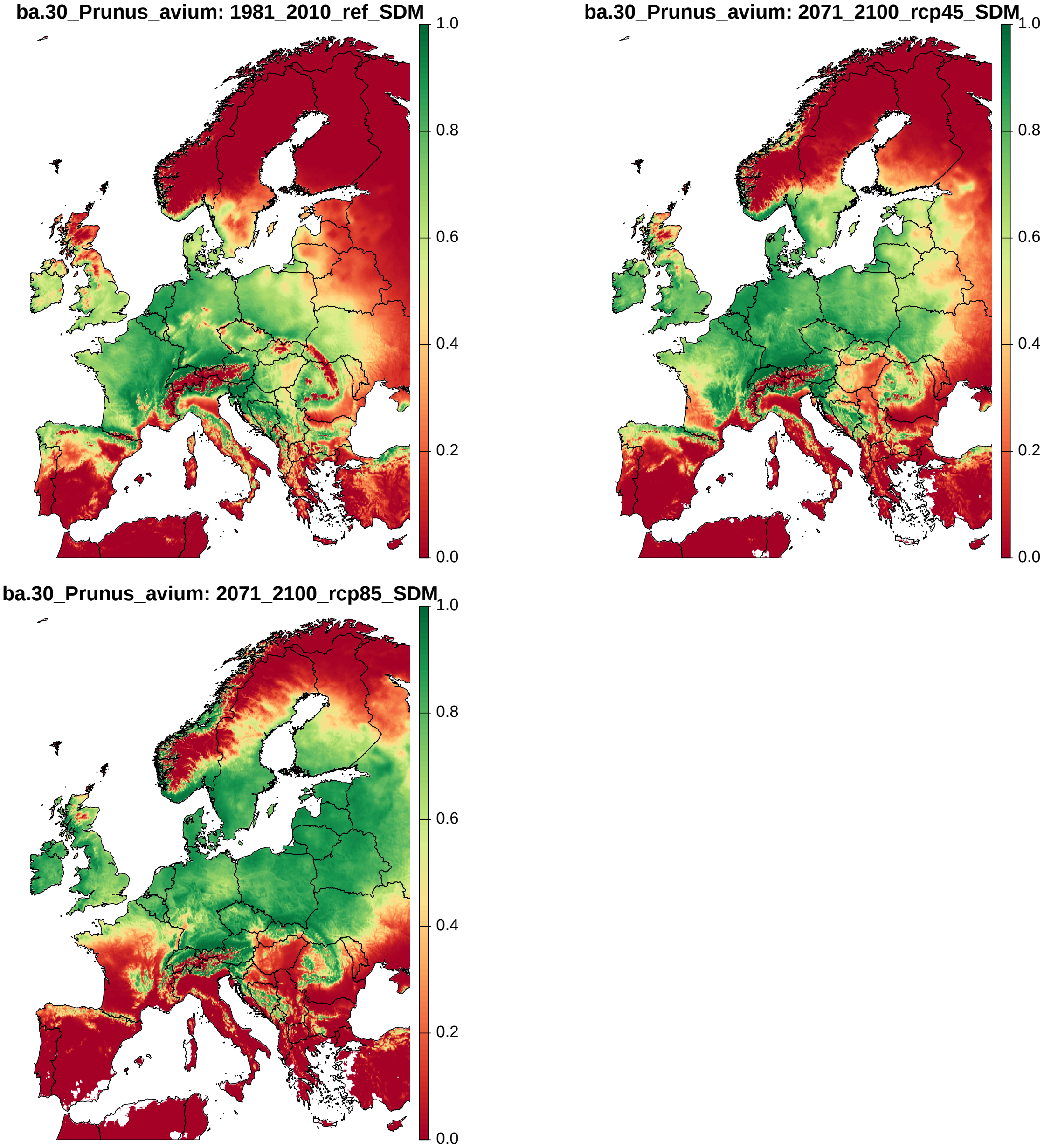

Climate risk classes

Maps of the climate risk classes. To estimate the distribution potential of each species as a mask for the SIMs, the continuous SDM outputs were categorized into three classes: low (yellow), medium (blue) and high climatic risk (red). The maps depict the risk classes in reference time (1981 to 2010), in climate scenario RCP4.5 (2071-2100) and RCP8.5 (2071-2100). To get an impression how well the thresholds fit to the data, presences (black) and absences (grey) were added on the reference map (top left). Refer to the legend and section “SDM thresholds” for the thresholds.

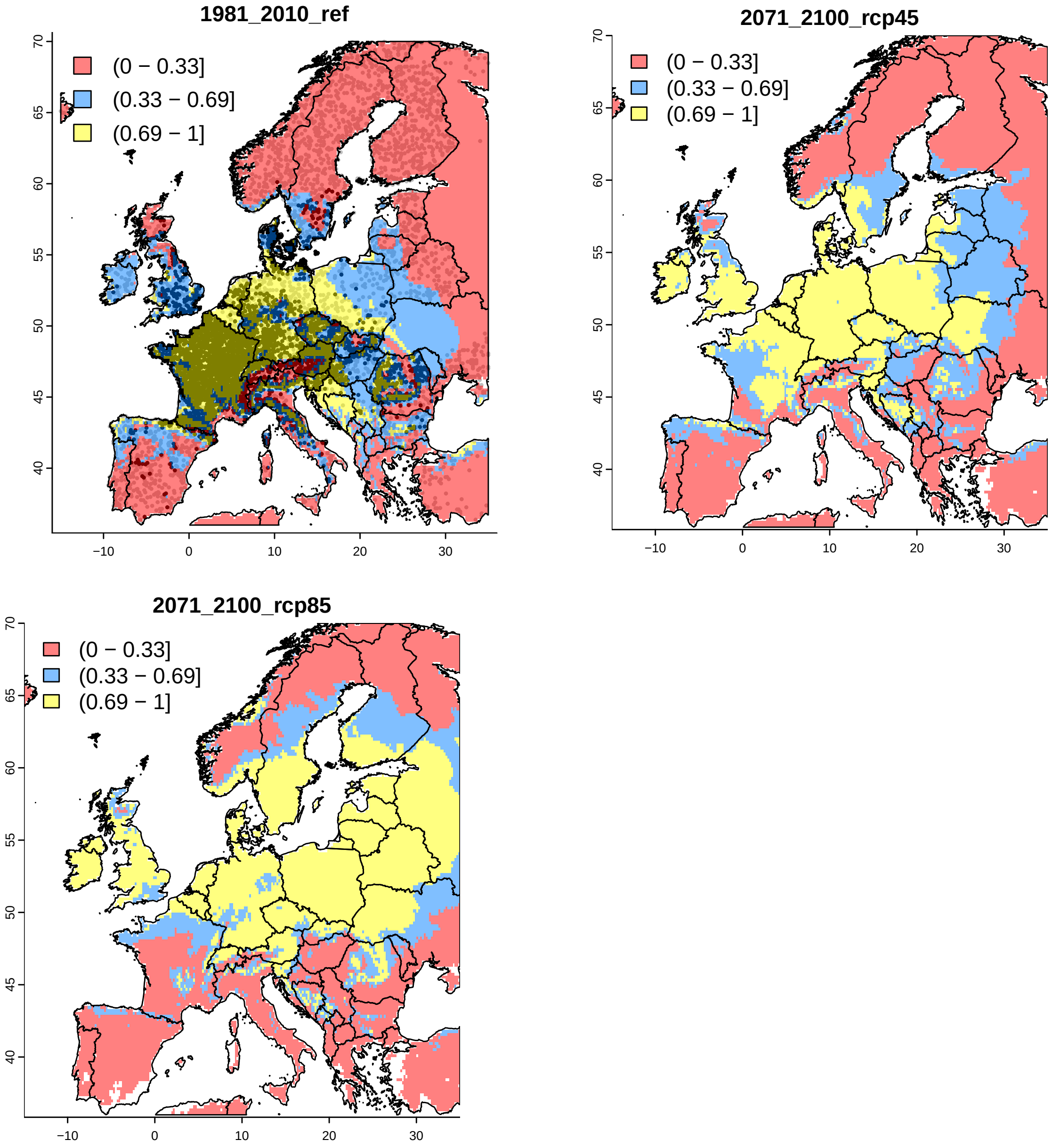

Pseudotsuga menziesii

Model statistics and evaluation

Summary

Predictor acronyms: Bio.10 = Mean temperature of warmest quarter [°C] within months 6 to 8, Bio.11 = Mean temperature of coldest quarter [°C] within months 12,1,2, Bio.12 = Annual precipitation sum [mm/m2], Bio.18 = Mean monthly precipitation amount of the warmest quarter [mm/m2] within months 6 to 8.

```
##
## Family: binomial
## Link function: logit
##
## Formula:
## ba.7 ~ s(Bio.10, k = 3) + s(Bio.11, k = 3) + s(Bio.18, k = 3)
##
## Parametric coefficients:
##             Estimate Std. Error z value Pr(>|z|)
## (Intercept)  -2.5701     0.1428  -17.99   <2e-16 ***
## ---
## Signif. codes:  0 '***' 0.001 '**' 0.01 '*' 0.05 '.' 0.1 ' ' 1
##
## Approximate significance of smooth terms:
##             edf Ref.df Chi.sq p-value
## s(Bio.10) 1.996      2  302.5  <2e-16 ***
## s(Bio.11) 1.996      2  620.8  <2e-16 ***
## s(Bio.18) 1.987      2  123.0  <2e-16 ***
## ---
## Signif. codes:  0 '***' 0.001 '**' 0.01 '*' 0.05 '.' 0.1 ' ' 1
##
## R-sq.(adj) =   0.58   Deviance explained = 51.2%
## -REML = 1631.6  Scale est. = 1         n = 4770
```

Evaluation parameter

Model performance was assessed using four statistical parameters: the area under the receiver operating characteristic curve (AUC), the true skill statistic (TSS), sensitivity (probability of the model to correctly predict a true presence) and specificity (probability of the model to correctly predict a true absence).

```
##             Species_name  AUC       TSS sensitivity specificity
## tp Pseudotsuga menziesii 0.92 0.7228512   0.9341719   0.7886792
```

Response curves and response maps

Response curves

Response curves (also known as effect curves) give an overview of the climatic niche of a species by relating the occurrence probability to corresponding climatic values. Predictor acronyms: Bio.10 = Mean temperature of warmest quarter [°C] within months 6 to 8, Bio.11 = Mean temperature of coldest quarter [°C] within months 12,1,2, Bio.12 = Annual precipitation sum [mm/m2], Bio.18 = Mean monthly precipitation amount of the warmest quarter [mm/m2] within months 6 to 8. Lines on the x-axis mark the upper and lower limit of the used presences (red), the mean (bold black) and the median (bold blue).

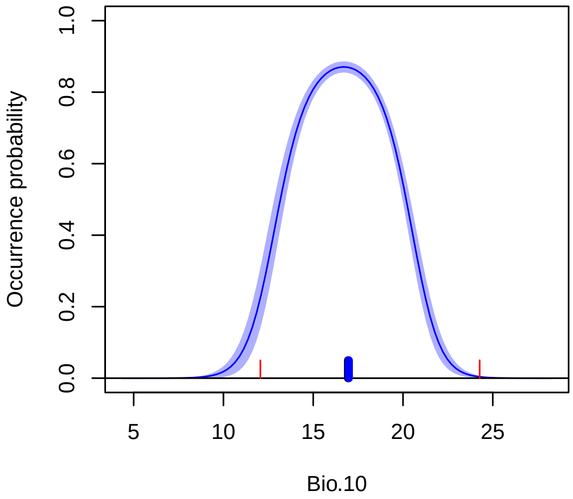

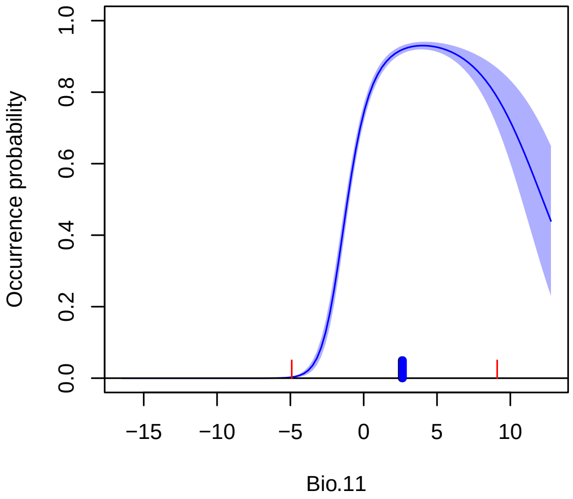

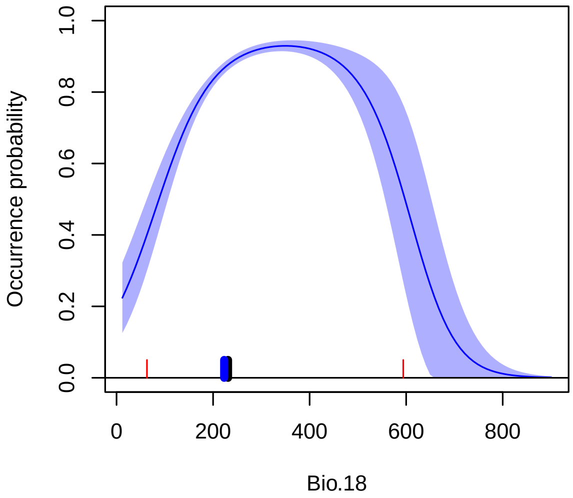

Response maps

Response maps (also referred to as partial effect maps). Each map represents how each predictor affects the occurrence probability. Predictor acronyms: Bio.10 = Mean temperature of warmest quarter [°C] within months 6 to 8, Bio.11 = Mean temperature of coldest quarter [°C] within months 12,1,2, Bio.12 = Annual precipitation sum [mm/m2], Bio.18 = Mean monthly precipitation amount of the warmest quarter [mm/m2] within months 6 to 8.

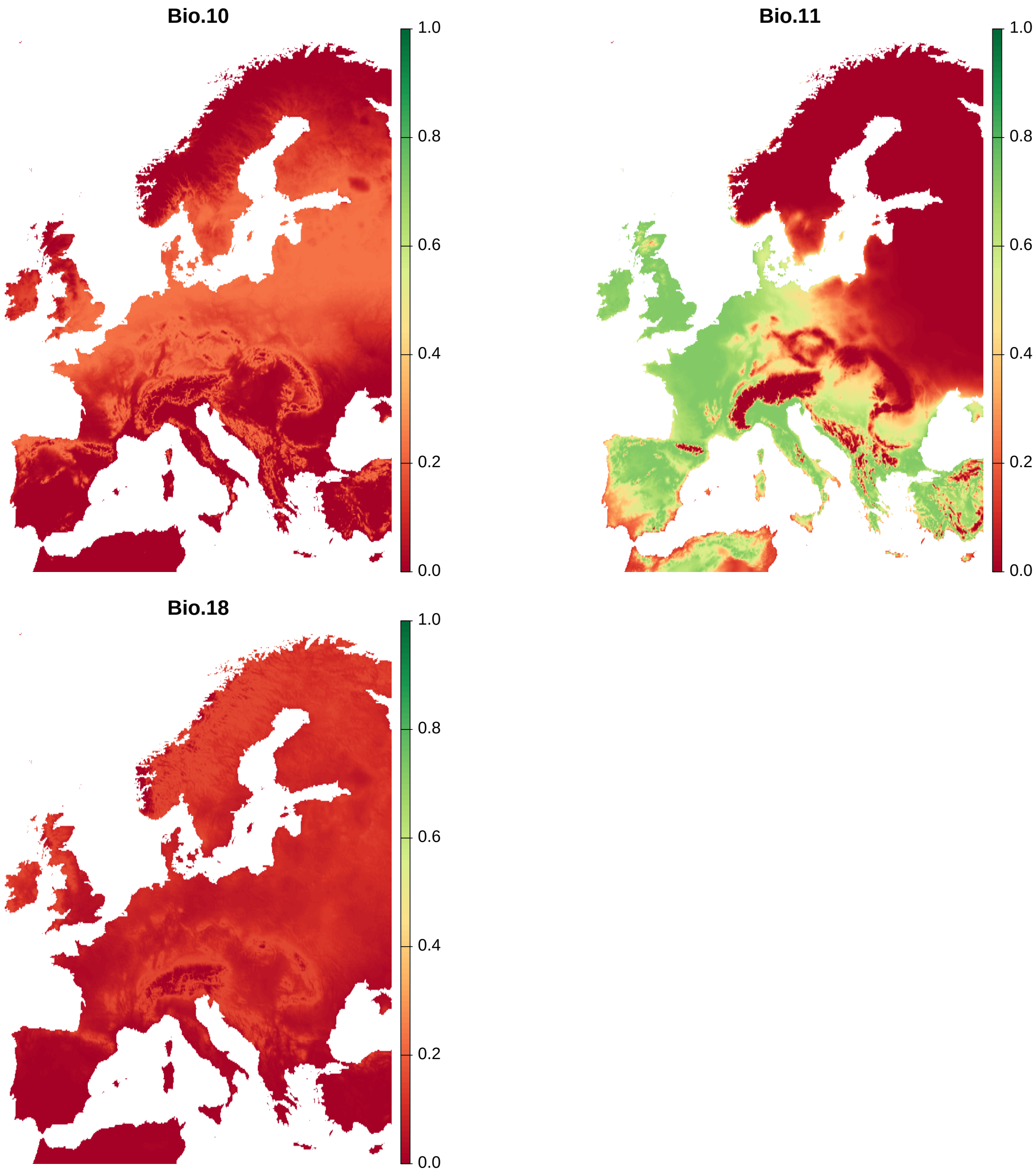

Model projections

Projection with plotted input data

Projection of species distribution model for reference period 1981-2010 over Europe. Occurrence probability ranges from 0 to 1 and is represented in dark red (low probability) to dark green (high probability). Input data used to calibrate the model is shown as presence points in magenta and absence points in black.

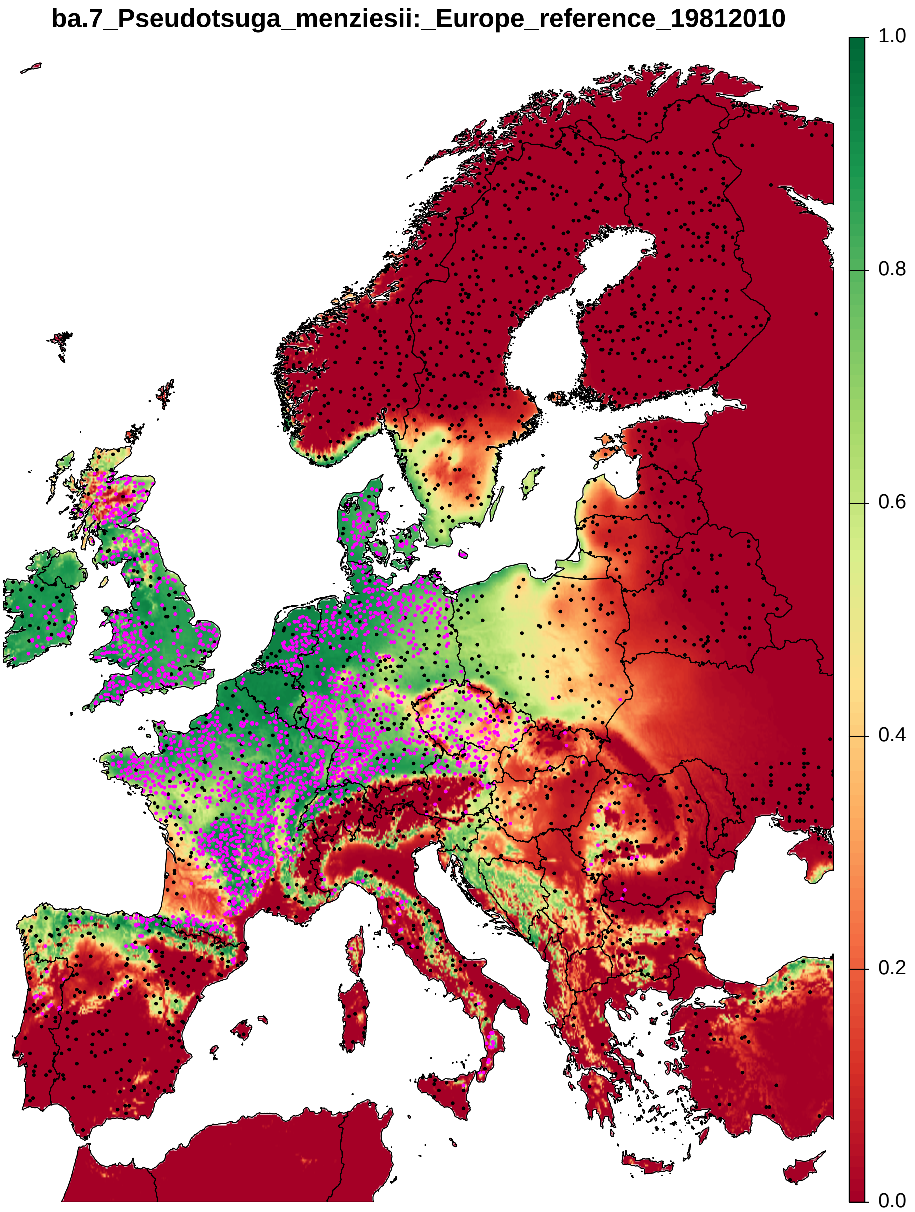

Projections

Projections of the species distribution models for reference period (1981-2010) and future scenarios RCP4.5 (2071-2100) and RCP8.5 (2071-2100) over Europe. Occurrence probabilities range from 0 to 1 and are represented from dark red (low probability) to dark green (high probability).

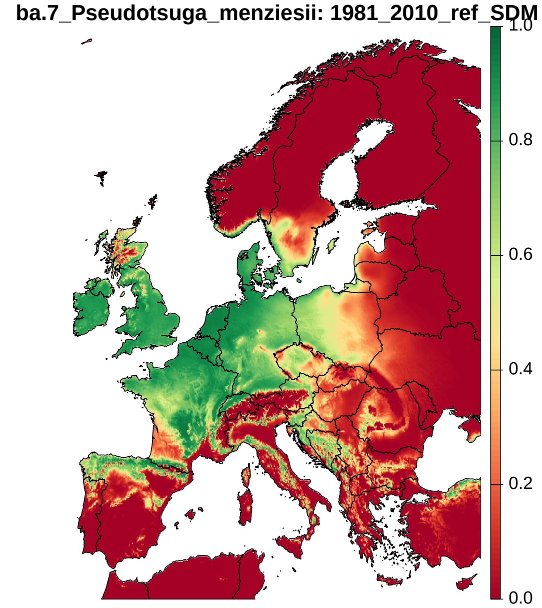

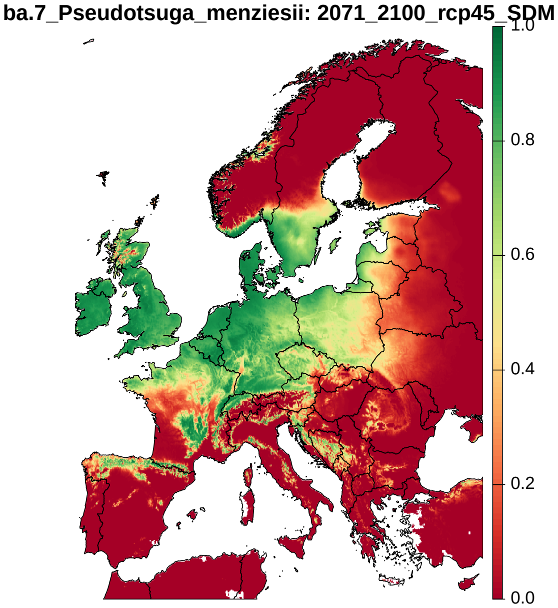

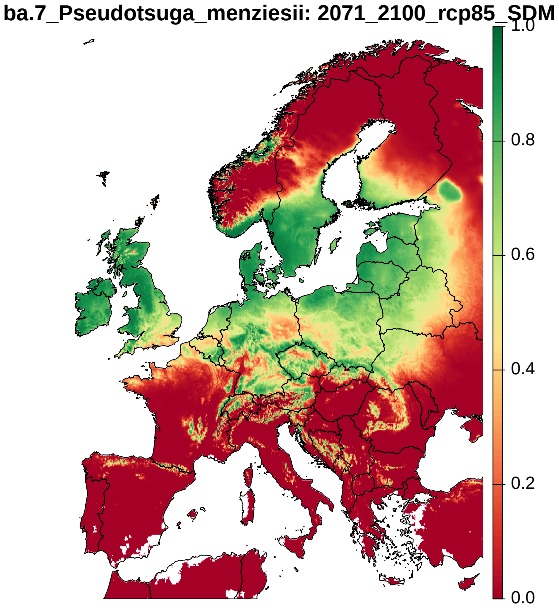

Climate risk classes

Maps of the climate risk classes. To estimate the distribution potential of each species as a mask for the SIMs, the continuous SDM outputs were categorized into three classes: low (yellow), medium (blue) and high climatic risk (red). The maps depict the risk classes in reference time (1981 to 2010), in climate scenario RCP4.5 (2071-2100) and RCP8.5 (2071-2100). To get an impression how well the thresholds fit to the data, presences (black) and absences (grey) were added on the reference map (top left). Refer to the legend and section "SDM thresholds" for the thresholds.

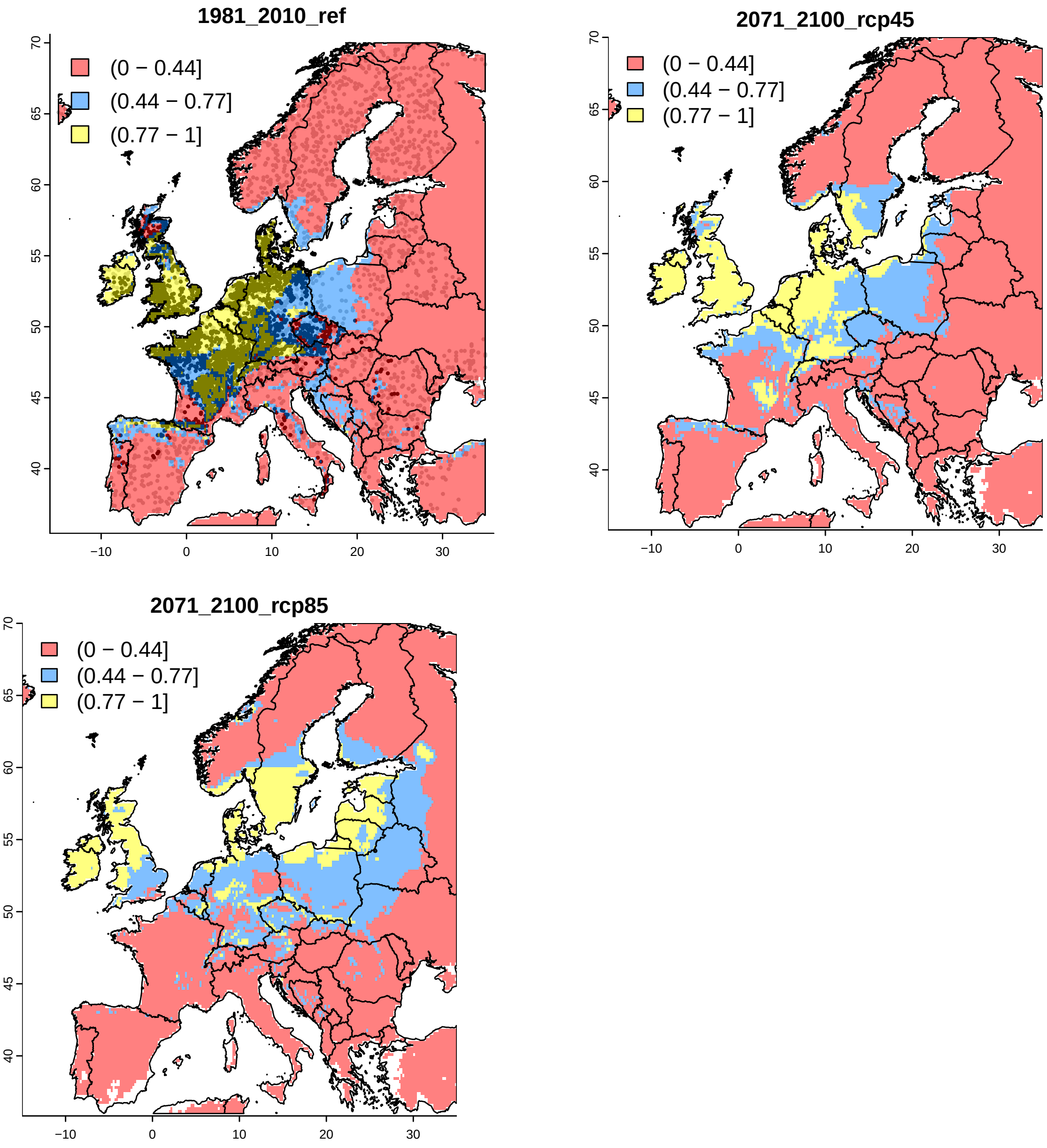

Pyrus pyraster

Model statistics and evaluation

Summary

Predictor acronyms: Bio.10 = Mean temperature of warmest quarter [°C] within months 6 to 8, Bio.11 = Mean temperature of coldest quarter [°C] within months 12,1,2, Bio.12 = Annual precipitation sum [mm/m2], Bio.18 = Mean monthly precipitation amount of the warmest quarter [mm/m2] within months 6 to 8.

```
##
## Family: binomial
## Link function: logit
##
## Formula:
## ba.29 ~ s(Bio.10, k = 3) + s(Bio.11, k = 3) + s(Bio.18, k = 3)
##
## Parametric coefficients:
##             Estimate Std. Error z value Pr(>|z|)
## (Intercept)  -1.5729     0.1488  -10.57   <2e-16 ***
## ---
## Signif. codes:  0 '***' 0.001 '**' 0.01 '*' 0.05 '.' 0.1 ' ' 1
##
## Approximate significance of smooth terms:
##             edf Ref.df Chi.sq p-value
## s(Bio.10) 1.993  2.000  173.8  <2e-16 ***
## s(Bio.11) 1.984  2.000  103.2  <2e-16 ***
## s(Bio.18) 1.638  1.869   83.5  <2e-16 ***
## ---
## Signif. codes:  0 '***' 0.001 '**' 0.01 '*' 0.05 '.' 0.1 ' ' 1
##
## R-sq.(adj) =  0.469   Deviance explained =   39%
## -REML = 683.04  Scale est. = 1         n = 1586
```

Evaluation parameter

Model performance was assessed using four statistical parameters: the area under the receiver operating characteristic curve (AUC), the true skill statistic (TSS), sensitivity (probability of the model to correctly predict a true presence) and specificity (probability of the model to correctly predict a true absence).

```
##      Species_name  AUC      TSS sensitivity specificity
## tp Pyrus pyraster 0.88 0.627995   0.8802018   0.7477932
```

Response curves and response maps

Response curves

Response curves (also known as effect curves) give an overview of the climatic niche of a species by relating the occurrence probability to corresponding climatic values. Predictor acronyms: Bio.10 = Mean temperature of warmest quarter [°C] within months 6 to 8, Bio.11 = Mean temperature of coldest quarter [°C] within months 12,1,2, Bio.12 = Annual precipitation sum [mm/m2], Bio.18 = Mean monthly precipitation amount of the warmest quarter [mm/m2] within months 6 to 8. Lines on the x-axis mark the upper and lower limit of the used presences (red), the mean (bold black) and the median (bold blue).

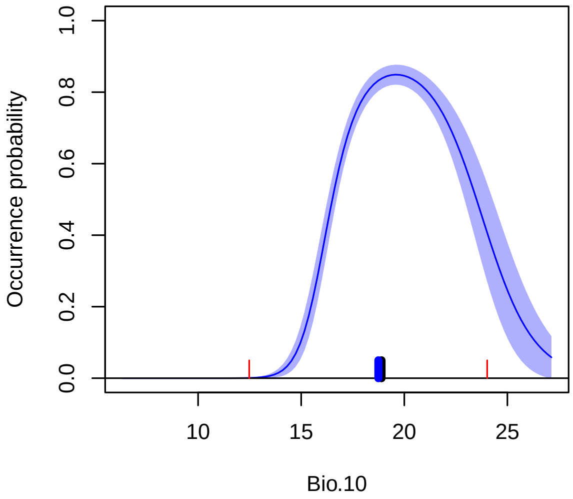

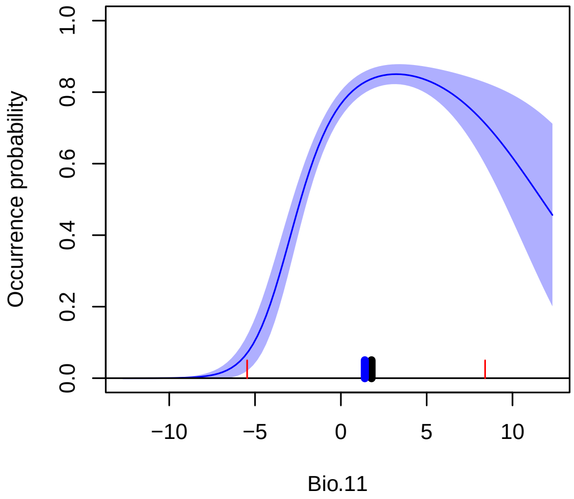

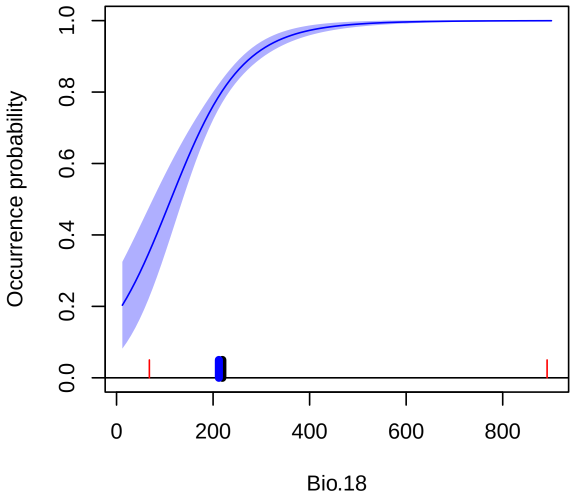

Response maps

Response maps (also referred to as partial effect maps). Each map represents how each predictor affects the occurrence probability. Predictor acronyms: Bio.10 = Mean temperature of warmest quarter [°C] within months 6 to 8, Bio.11 = Mean temperature of coldest quarter [°C] within months 12,1,2, Bio.12 = Annual precipitation sum [mm/m2], Bio.18 = Mean monthly precipitation amount of the warmest quarter [mm/m2] within months 6 to 8.

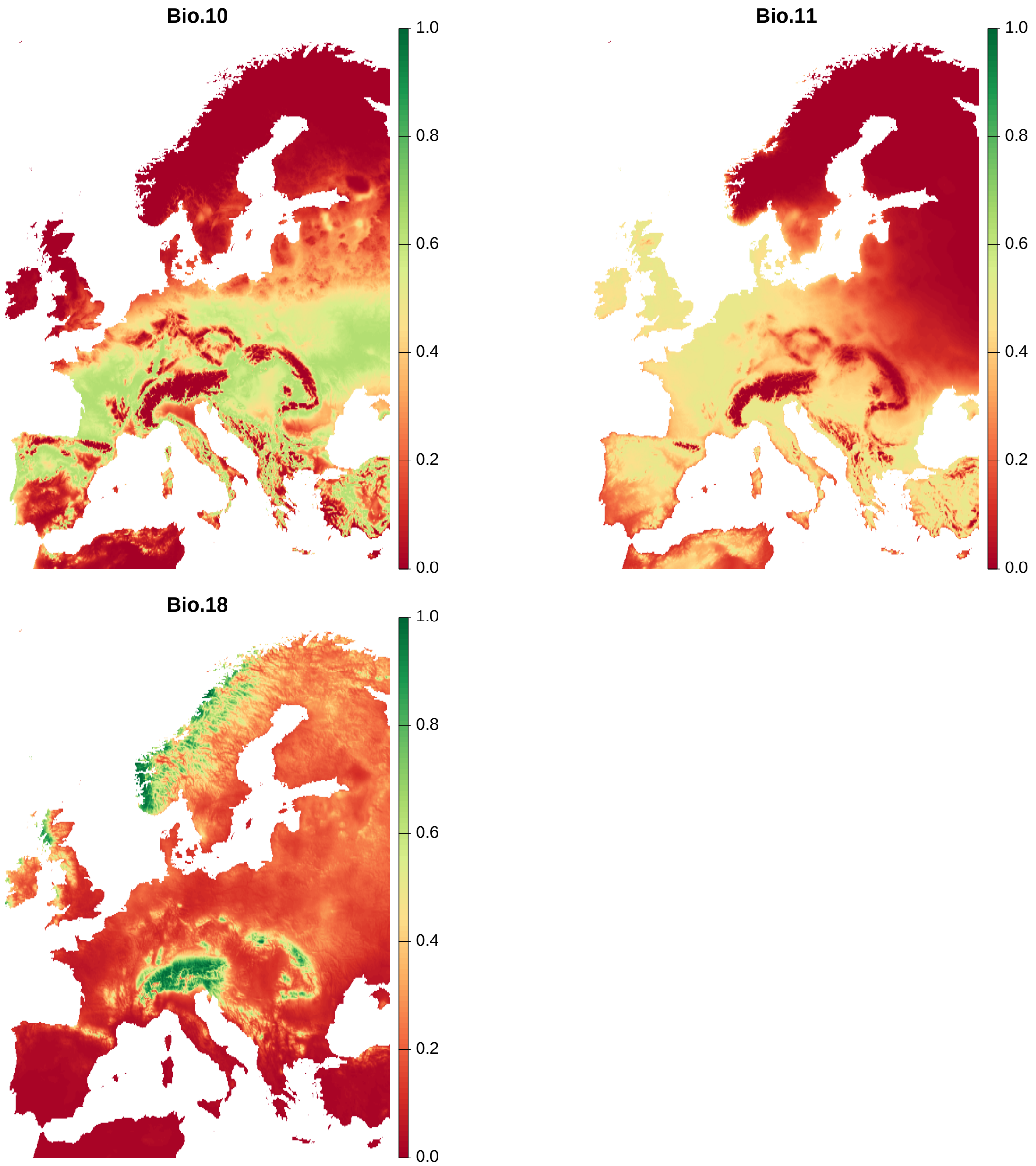

Model projections

Projection with plotted input data

Projection of species distribution model for reference period 1981-2010 over Europe. Occurrence probability ranges from 0 to 1 and is represented in dark red (low probability) to dark green (high probability). Input data used to calibrate the model is shown as presence points in magenta and absence points in black.

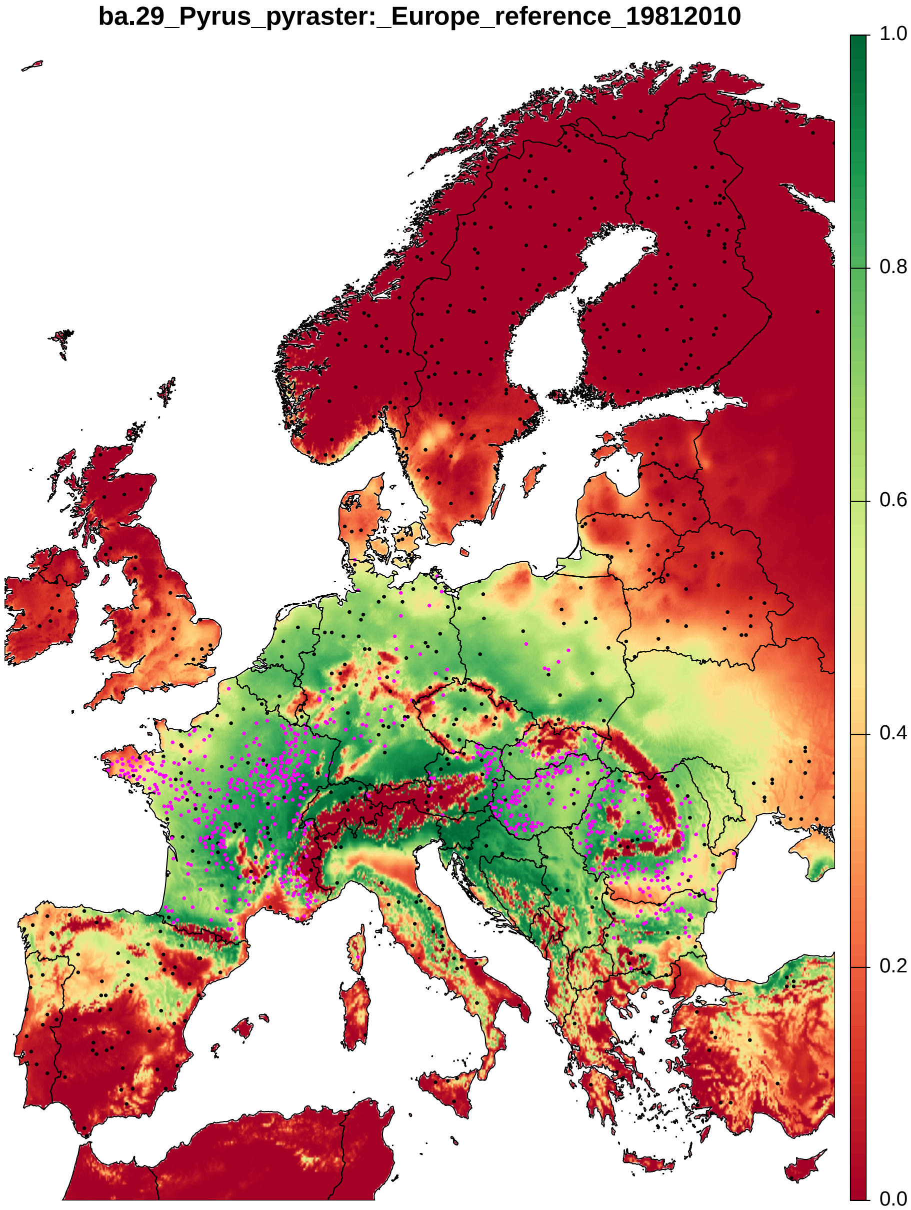

Projections

Projections of the species distribution models for reference period (1981-2010) and future scenarios RCP4.5 (2071-2100) and RCP8.5 (2071-2100) over Europe. Occurrence probabilities range from 0 to 1 and are represented from dark red (low probability) to dark green (high probability).

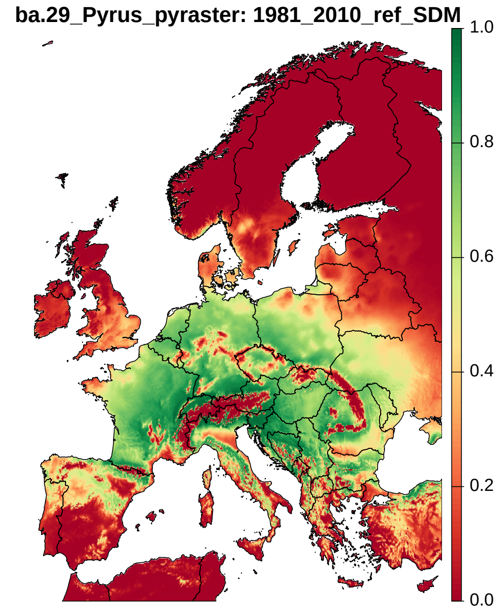

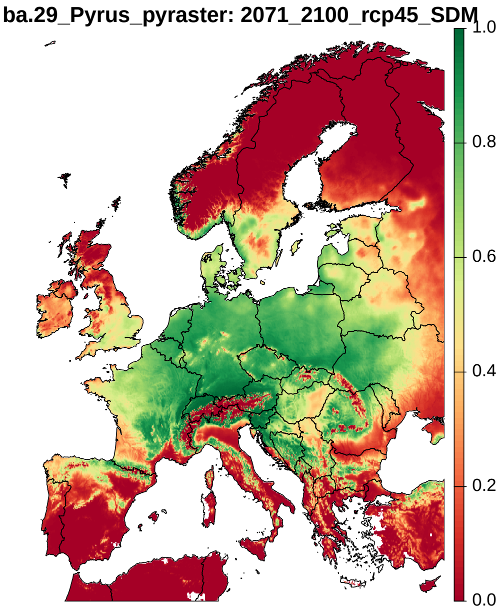

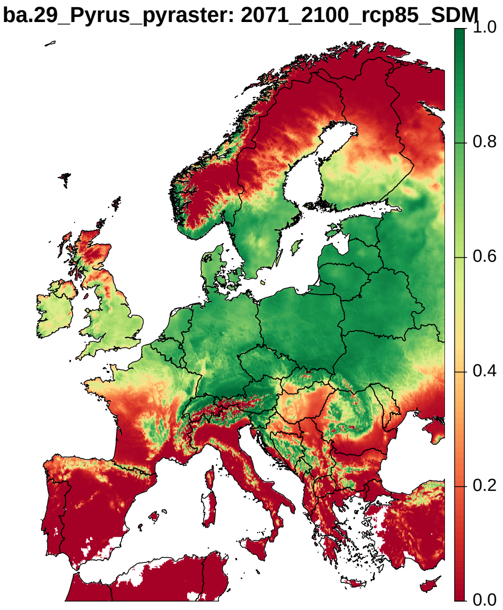

Climate risk classes

Maps of the climate risk classes. To estimate the distribution potential of each species as a mask for the SIMs, the continuous SDM outputs were categorized into three classes: low (yellow), medium (blue) and high climatic risk (red). The maps depict the risk classes in reference time (1981 to 2010), in climate scenario RCP4.5 (2071-2100) and RCP8.5 (2071-2100). To get an impression how well the thresholds fit to the data, presences (black) and absences (grey) were added on the reference map (top left). Refer to the legend and section “SDM thresholds” for the thresholds.

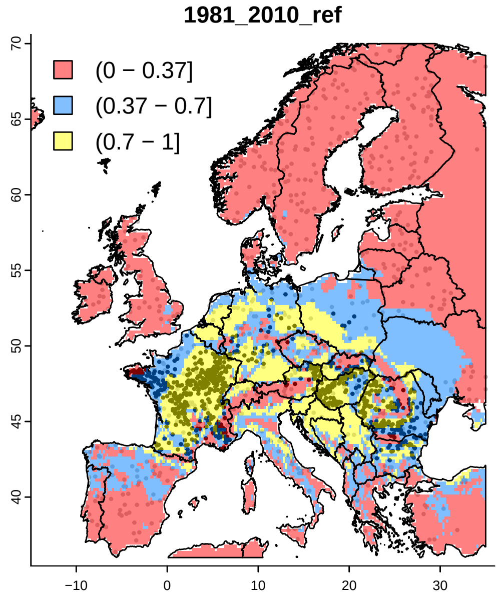

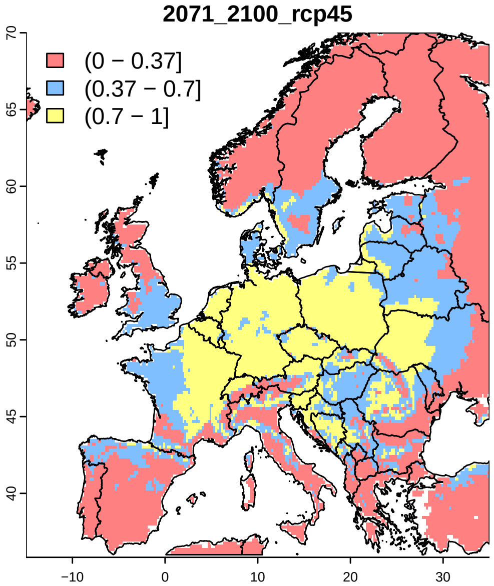

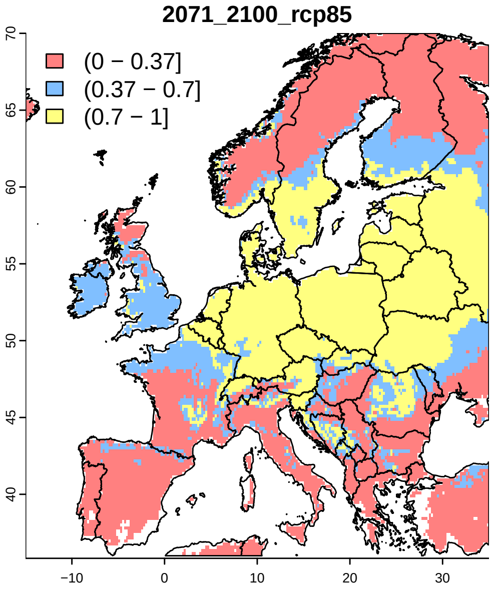

Quercus cerris

Model statistics and evaluation

Summary

Predictor acronyms: Bio.10 = Mean temperature of warmest quarter [°C] within months 6 to 8, Bio.11 = Mean temperature of coldest quarter [°C] within months 12,1,2, Bio.12 = Annual precipitation sum [mm/m2], Bio.18 = Mean monthly precipitation amount of the warmest quarter [mm/m2] within months 6 to 8.

```
##
## Family: binomial
## Link function: logit
##
## Formula:
## ba.14 ~ s(Bio.10, k = 3) + s(Bio.11, k = 3) + s(Bio.12, k = 3)
##
## Parametric coefficients:
##             Estimate Std. Error z value Pr(>|z|)
## (Intercept)  -2.6166     0.1707  -15.32   <2e-16 ***
## ---
## Signif. codes:  0 '***' 0.001 '**' 0.01 '*' 0.05 '.' 0.1 ' ' 1
##
## Approximate significance of smooth terms:
##             edf Ref.df Chi.sq p-value
## s(Bio.10) 1.993  2.000 342.83  <2e-16 ***
## s(Bio.11) 1.995  2.000 215.82  <2e-16 ***
## s(Bio.12) 1.971  1.999  80.46  <2e-16 ***
## ---
## Signif. codes:  0 '***' 0.001 '**' 0.01 '*' 0.05 '.' 0.1 ' ' 1
##
## R-sq.(adj) =  0.551   Deviance explained = 48.1%
## -REML = 994.03  Scale est. = 1         n = 2722
```

Evaluation parameter

Model performance was assessed using four statistical parameters: the area under the receiver operating characteristic curve (AUC), the true skill statistic (TSS), sensitivity (probability of the model to correctly predict a true presence) and specificity (probability of the model to correctly predict a true absence).

```
##      Species_name  AUC       TSS sensitivity specificity
## tp Quercus cerris 0.92 0.6877296   0.9110948   0.7766348
```

Response curves and response maps

Response curves

Response curves (also known as effect curves) give an overview of the climatic niche of a species by relating the occurrence probability to corresponding climatic values. Predictor acronyms: Bio.10 = Mean temperature of warmest quarter [°C] within months 6 to 8, Bio.11 = Mean temperature of coldest quarter [°C] within months 12,1,2, Bio.12 = Annual precipitation sum [mm/m2], Bio.18 = Mean monthly precipitation amount of the warmest quarter [mm/m2] within months 6 to 8. Lines on the x-axis mark the upper and lower limit of the used presences (red), the mean (bold black) and the median (bold blue).

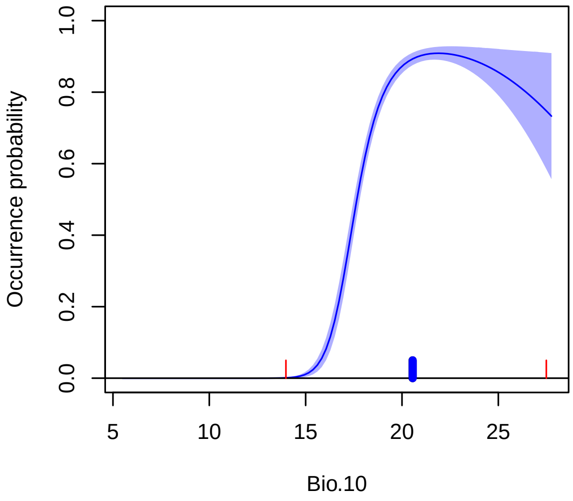

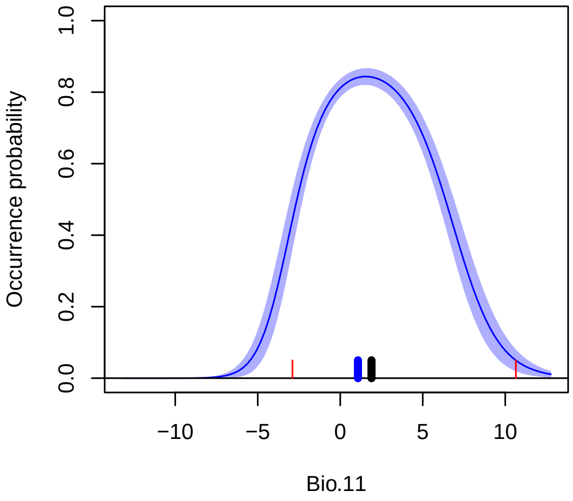

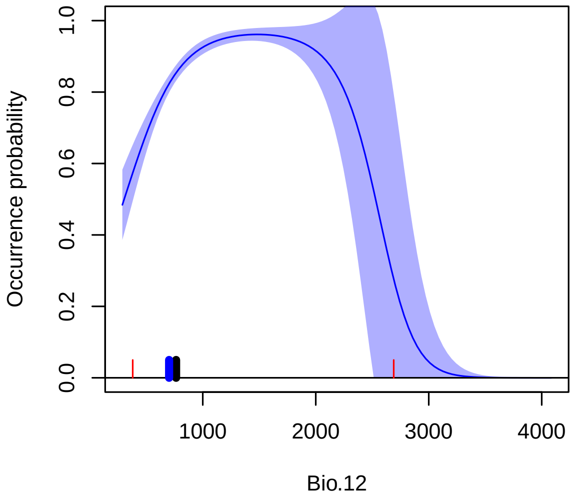

Response maps

Response maps (also referred to as partial effect maps). Each map represents how each predictor affects the occurrence probability. Predictor acronyms: Bio.10 = Mean temperature of warmest quarter [°C] within months 6 to 8, Bio.11 = Mean temperature of coldest quarter [°C] within months 12,1,2, Bio.12 = Annual precipitation sum [mm/m2], Bio.18 = Mean monthly precipitation amount of the warmest quarter [mm/m2] within months 6 to 8.

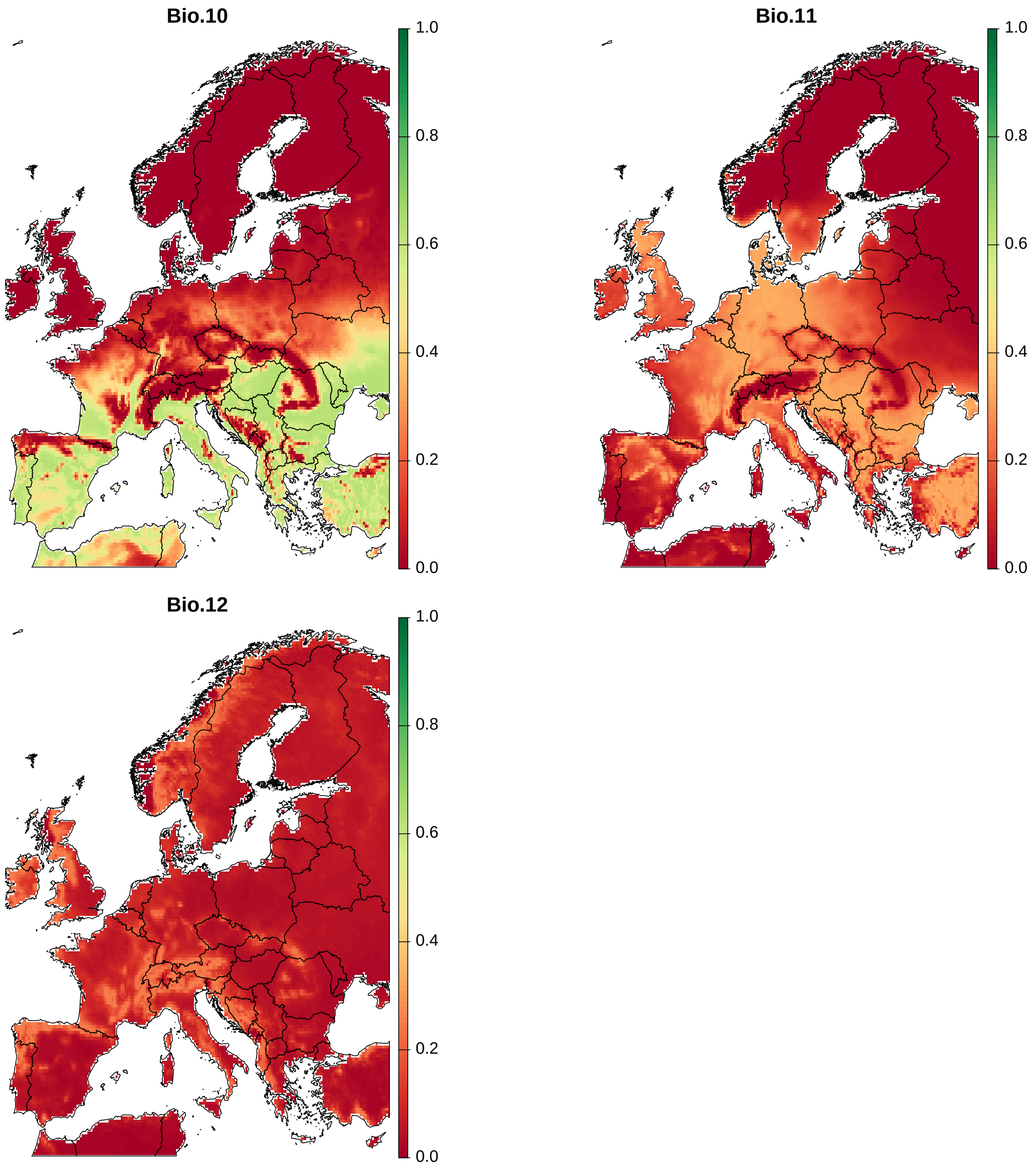

Model projections

Projection with plotted input data

Projection of species distribution model for reference period 1981-2010 over Europe. Occurrence probability ranges from 0 to 1 and is represented in dark red (low probability) to dark green (high probability). Input data used to calibrate the model is shown as presence points in magenta and absence points in black.

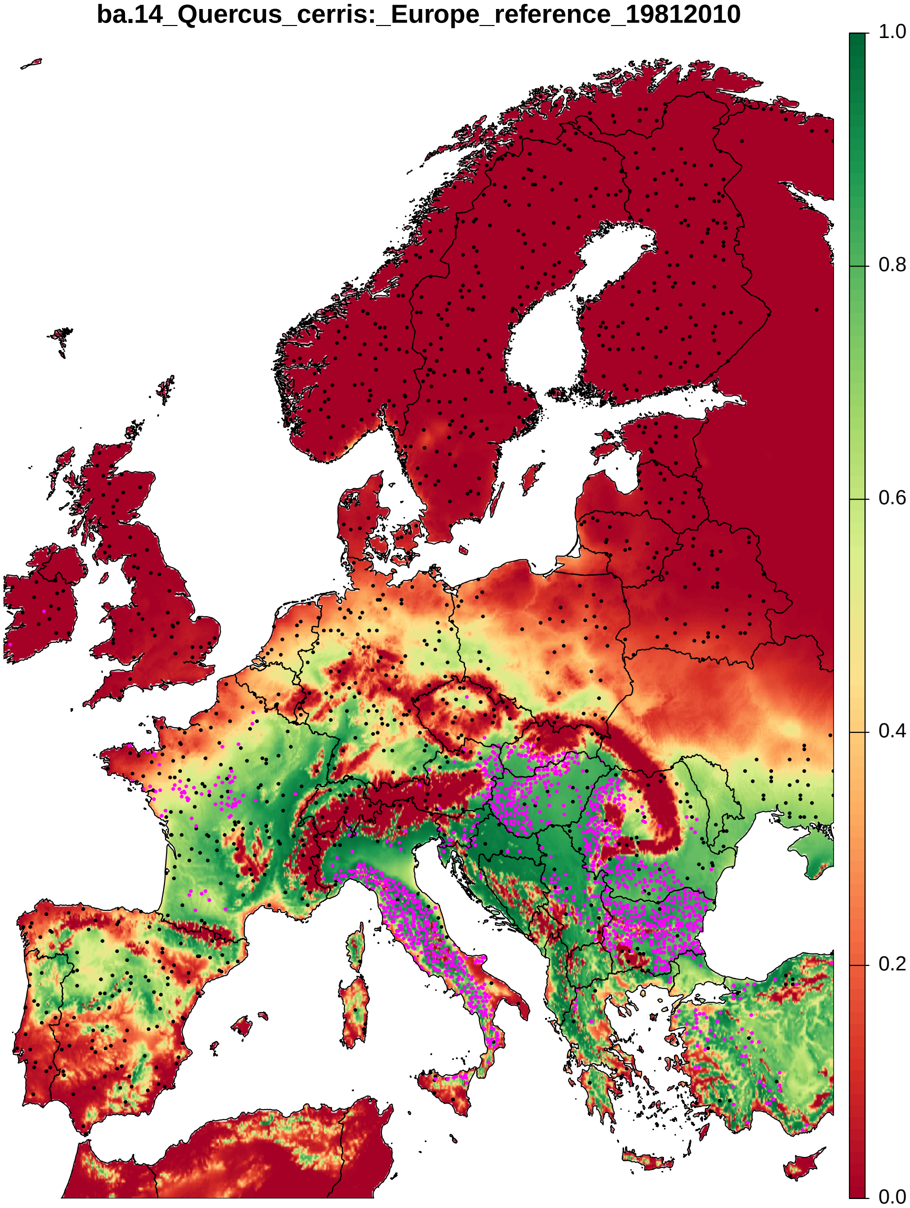

Projections

Projections of the species distribution models for reference period (1981-2010) and future scenarios RCP4.5 (2071-2100) and RCP8.5 (2071-2100) over Europe. Occurrence probabilities range from 0 to 1 and are represented from dark red (low probability) to dark green (high probability).

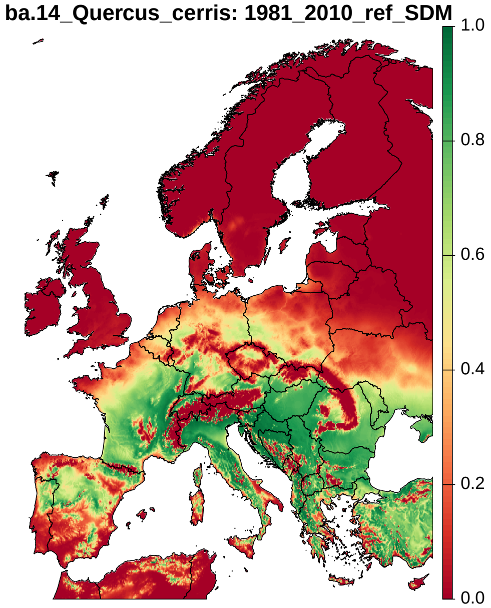

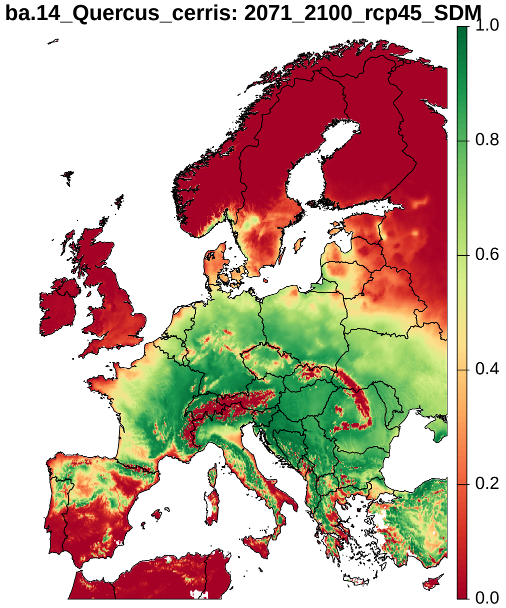

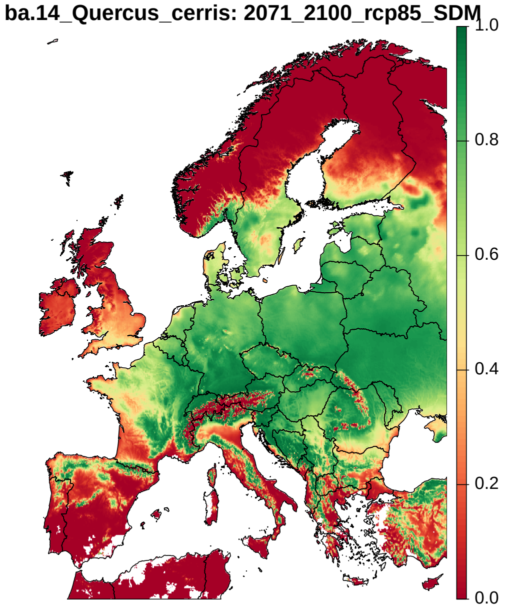

Climate risk classes

Maps of the climate risk classes. To estimate the distribution potential of each species as a mask for the SIMs, the continuous SDM outputs were categorized into three classes: low (yellow), medium (blue) and high climatic risk (red). The maps depict the risk classes in reference time (1981 to 2010), in climate scenario RCP4.5 (2071-2100) and RCP8.5 (2071-2100). To get an impression how well the thresholds fit to the data, presences (black) and absences (grey) were added on the reference map (top left). Refer to the legend and section “SDM thresholds” for the thresholds.

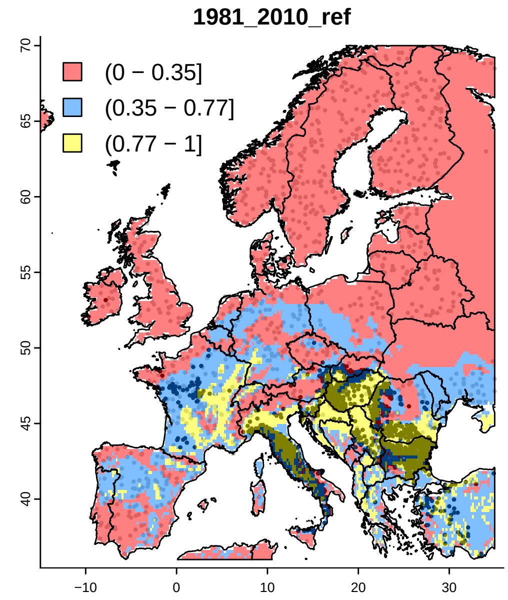

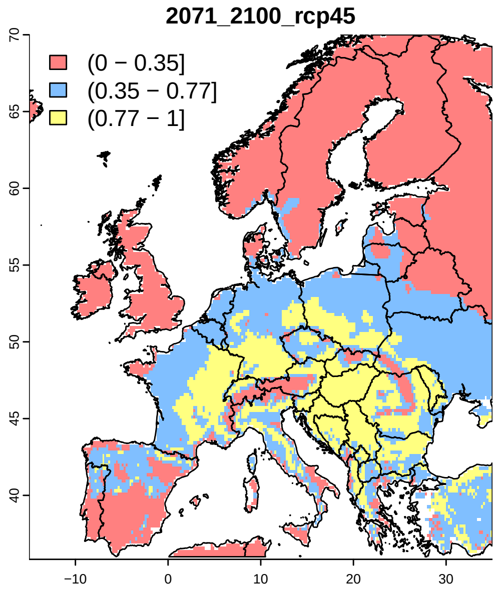

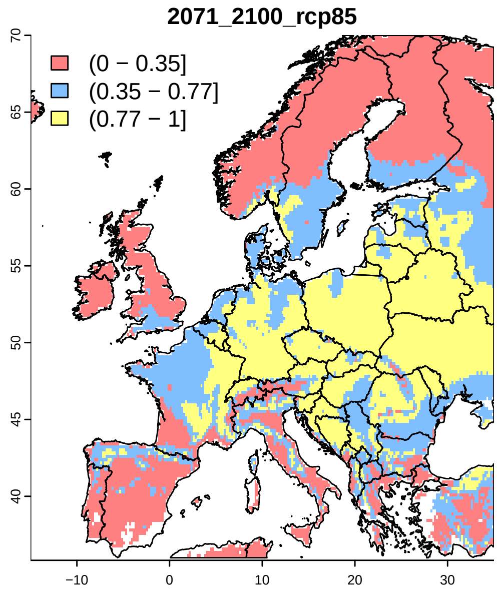

Quercus petraea

Model statistics and evaluation

Summary

Predictor acronyms: Bio.10 = Mean temperature of warmest quarter [°C] within months 6 to 8, Bio.11 = Mean temperature of coldest quarter [°C] within months 12,1,2, Bio.12 = Annual precipitation sum [mm/m2], Bio.18 = Mean monthly precipitation amount of the warmest quarter [mm/m2] within months 6 to 8.

```
##
## Family: binomial
## Link function: logit
##
## Formula:
## ba.11 ~ s(Bio.10, k = 3) + s(Bio.11, k = 3) + s(Bio.18, k = 3)
##
## Parametric coefficients:
##             Estimate Std. Error z value Pr(>|z|)
## (Intercept) -1.98723    0.07498   -26.5   <2e-16 ***
## ---
## Signif. codes:  0 '***' 0.001 '**' 0.01 '*' 0.05 '.' 0.1 ' ' 1
##
## Approximate significance of smooth terms:
##             edf Ref.df Chi.sq p-value
## s(Bio.10) 1.999      2  799.5  <2e-16 ***
## s(Bio.11) 1.998      2  890.8  <2e-16 ***
## s(Bio.18) 1.993      2  432.2  <2e-16 ***
## ---
## Signif. codes:  0 '***' 0.001 '**' 0.01 '*' 0.05 '.' 0.1 ' ' 1
##
## R-sq.(adj) =  0.535   Deviance explained =   46%
## -REML = 3294.2  Scale est. = 1         n = 8750
```

Evaluation parameter

Model performance was assessed using four statistical parameters: the area under the receiver operating characteristic curve (AUC), the true skill statistic (TSS), sensitivity (probability of the model to correctly predict a true presence) and specificity (probability of the model to correctly predict a true absence).

```
##       Species_name  AUC       TSS sensitivity specificity
## tp Quercus petraea 0.91 0.6882286   0.9051429   0.7830857
```

Response curves and response maps

Response curves

Response curves (also known as effect curves) give an overview of the climatic niche of a species by relating the occurrence probability to corresponding climatic values. Predictor acronyms: Bio.10 = Mean temperature of warmest quarter [°C] within months 6 to 8, Bio.11 = Mean temperature of coldest quarter [°C] within months 12,1,2, Bio.12 = Annual precipitation sum [mm/m2], Bio.18 = Mean monthly precipitation amount of the warmest quarter [mm/m2] within months 6 to 8. Lines on the x-axis mark the upper and lower limit of the used presences (red), the mean (bold black) and the median (bold blue).

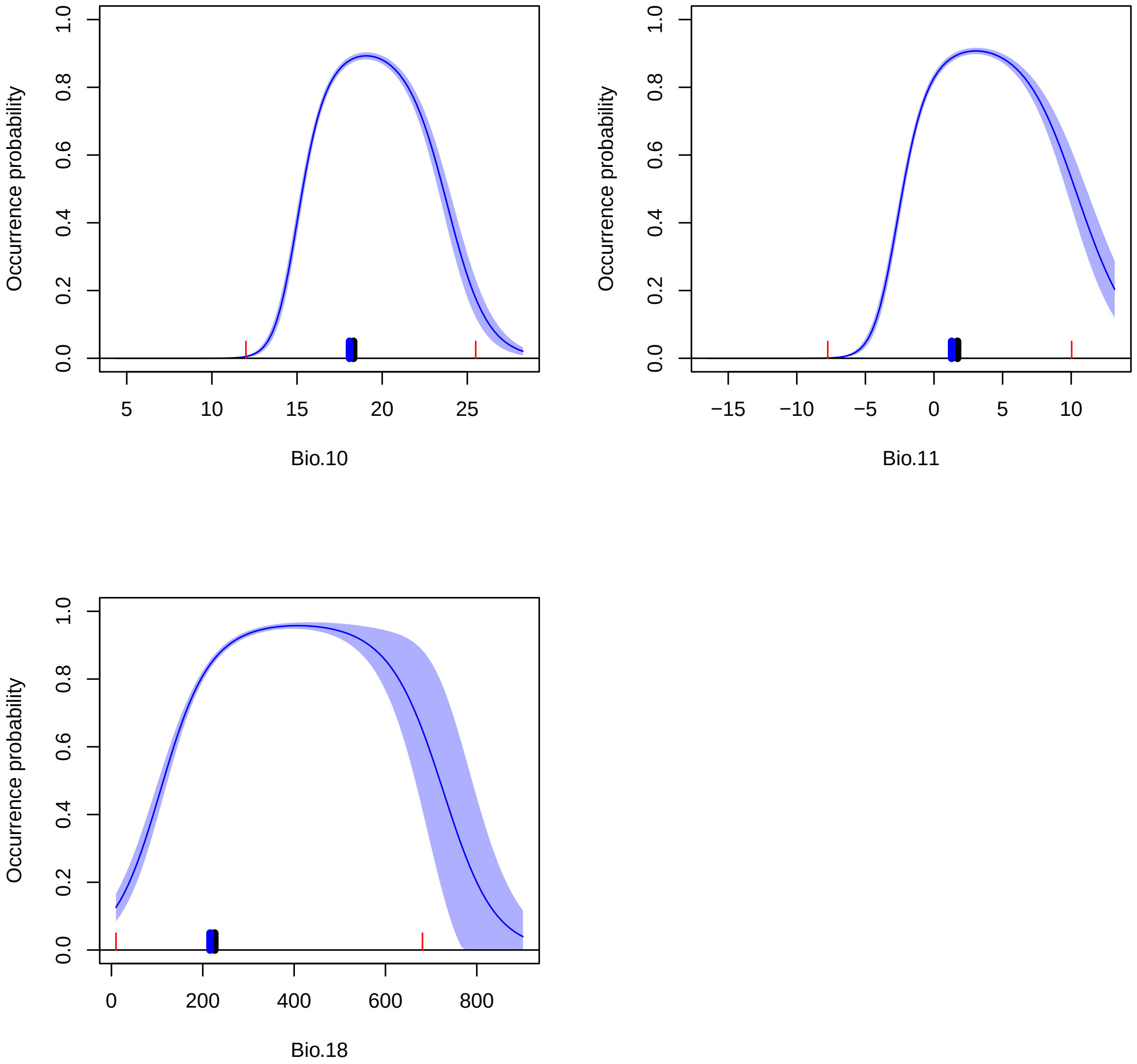

Response maps

Response maps (also referred to as partial effect maps). Each map represents how each predictor affects the occurrence probability. Predictor acronyms: Bio.10 = Mean temperature of warmest quarter [°C] within months 6 to 8, Bio.11 = Mean temperature of coldest quarter [°C] within months 12,1,2, Bio.12 = Annual precipitation sum [mm/m2], Bio.18 = Mean monthly precipitation amount of the warmest quarter [mm/m2] within months 6 to 8.

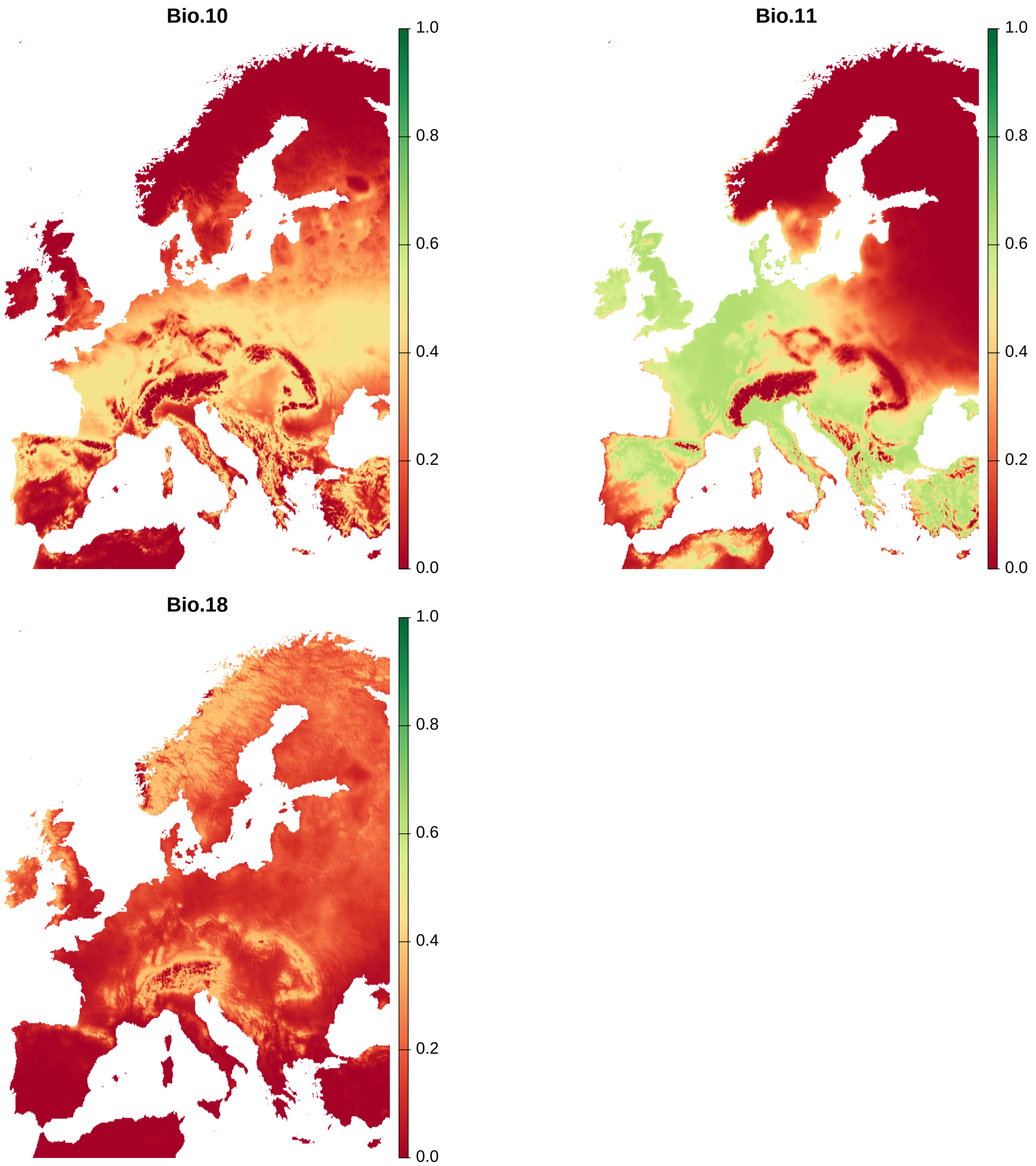

Model projections

Projection with plotted input data

Projection of species distribution model for reference period 1981-2010 over Europe. Occurrence probability ranges from 0 to 1 and is represented in dark red (low probability) to dark green (high probability). Input data used to calibrate the model is shown as presence points in magenta and absence points in black.

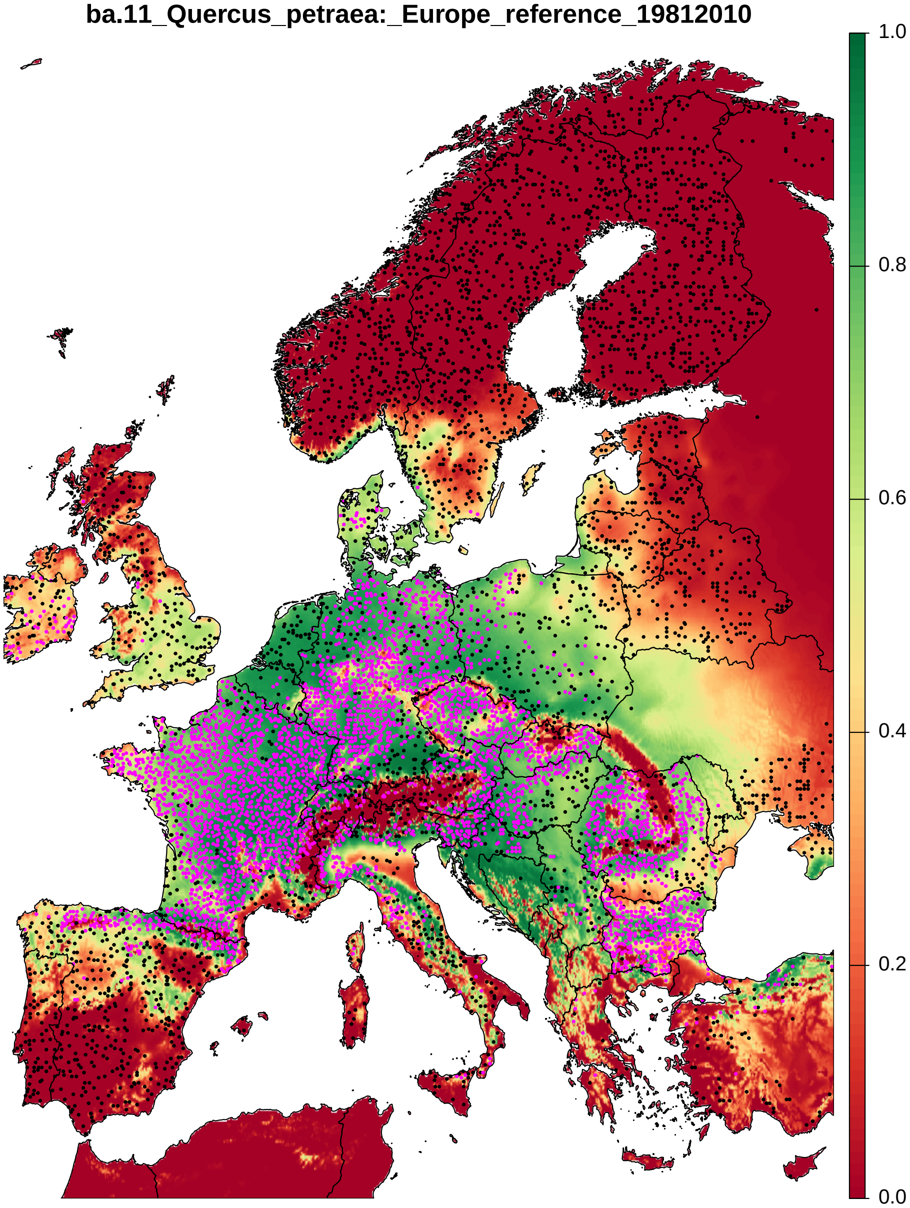

Projections

Projections of the species distribution models for reference period (1981-2010) and future scenarios RCP4.5 (2071-2100) and RCP8.5 (2071-2100) over Europe. Occurrence probabilities range from 0 to 1 and are represented from dark red (low probability) to dark green (high probability).

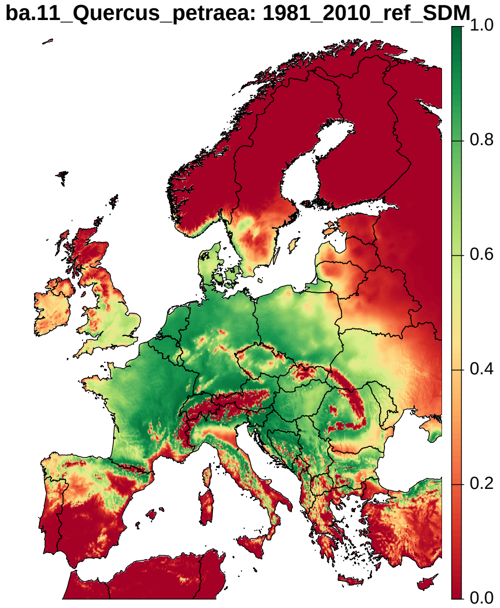

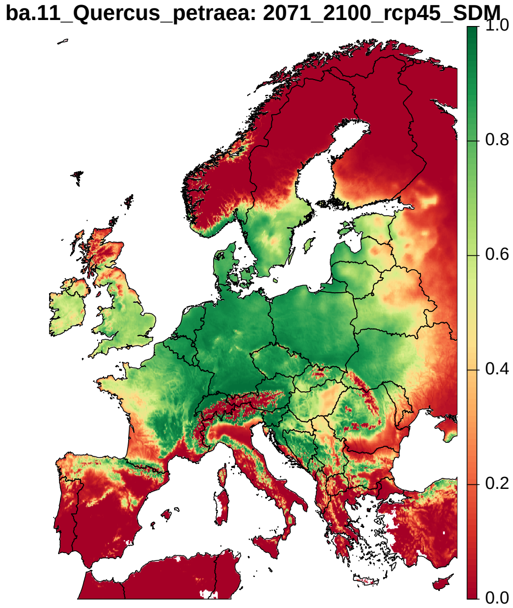

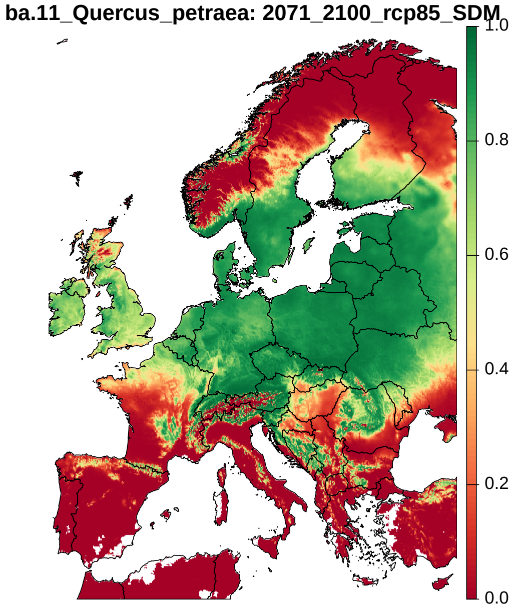

Climate risk classes

Maps of the climate risk classes. To estimate the distribution potential of each species as a mask for the SIMs, the continuous SDM outputs were categorized into three classes: low (yellow), medium (blue) and high climatic risk (red). The maps depict the risk classes in reference time (1981 to 2010), in climate scenario RCP4.5 (2071-2100) and RCP8.5 (2071-2100). To get an impression how well the thresholds fit to the data, presences (black) and absences (grey) were added on the reference map (top left). Refer to the legend and section "SDM thresholds" for the thresholds.

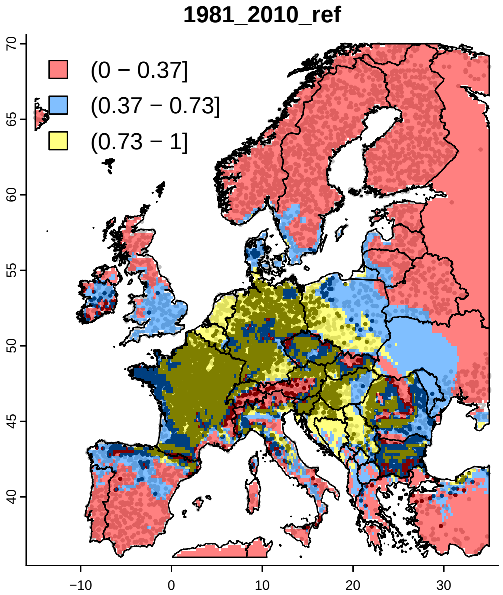

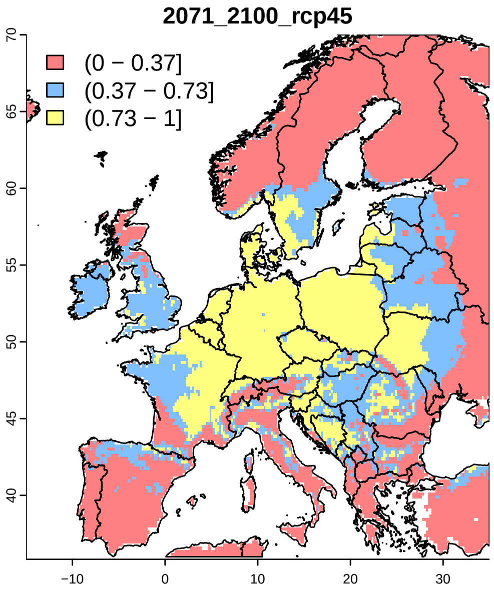

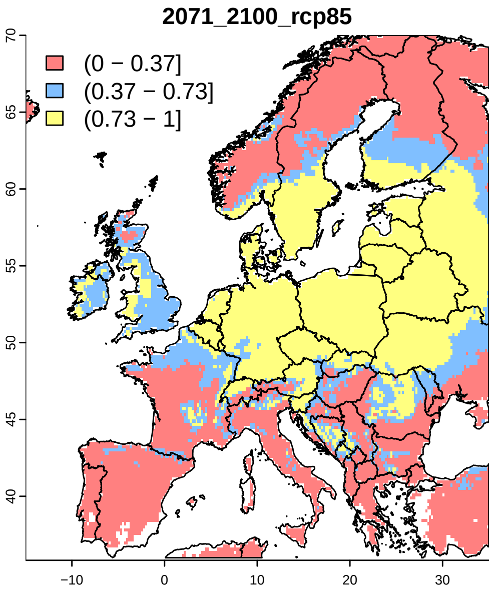

Quercus pubescens

Model statistics and evaluation

Summary

Predictor acronyms: Bio.10 = Mean temperature of warmest quarter [°C] within months 6 to 8, Bio.11 = Mean temperature of coldest quarter [°C] within months 12,1,2, Bio.12 = Annual precipitation sum [mm/m2], Bio.18 = Mean monthly precipitation amount of the warmest quarter [mm/m2] within months 6 to 8.

```
##
## Family: binomial
## Link function: logit
##
## Formula:
## ba.12 ~ s(Bio.10, k = 3) + s(Bio.11, k = 3) + s(Bio.12, k = 3)
##
## Parametric coefficients:
##             Estimate Std. Error z value Pr(>|z|)
## (Intercept)  -2.2093     0.1328  -16.63   <2e-16 ***
## ---
## Signif. codes:  0 '***' 0.001 '**' 0.01 '*' 0.05 '.' 0.1 ' ' 1
##
## Approximate significance of smooth terms:
##             edf Ref.df Chi.sq p-value
## s(Bio.10) 1.995      2  397.4  <2e-16 ***
## s(Bio.11) 1.993      2  165.1  <2e-16 ***
## s(Bio.12) 1.985      2  233.6  <2e-16 ***
## ---
## Signif. codes:  0 '***' 0.001 '**' 0.01 '*' 0.05 '.' 0.1 ' ' 1
##
## R-sq.(adj) =  0.566   Deviance explained = 48.2%
## -REML = 1380.6  Scale est. = 1         n = 3800
```

Evaluation parameter

Model performance was assessed using four statistical parameters: the area under the receiver operating characteristic curve (AUC), the true skill statistic (TSS), sensitivity (probability of the model to correctly predict a true presence) and specificity (probability of the model to correctly predict a true absence).

```
##          Species_name  AUC       TSS sensitivity specificity
## tp Quercus pubescens 0.92 0.7063158   0.9142105   0.7921053
```

Response curves and response maps

Response curves

Response curves (also known as effect curves) give an overview of the climatic niche of a species by relating the occurrence probability to corresponding climatic values. Predictor acronyms: Bio.10 = Mean temperature of warmest quarter [°C] within months 6 to 8, Bio.11 = Mean temperature of coldest quarter [°C] within months 12,1,2, Bio.12 = Annual precipitation sum [mm/m2], Bio.18 = Mean monthly precipitation amount of the warmest quarter [mm/m2] within months 6 to 8. Lines on the x-axis mark the upper and lower limit of the used presences (red), the mean (bold black) and the median (bold blue).

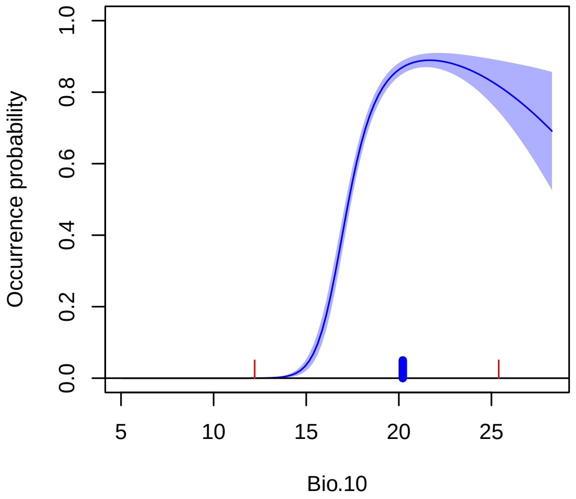

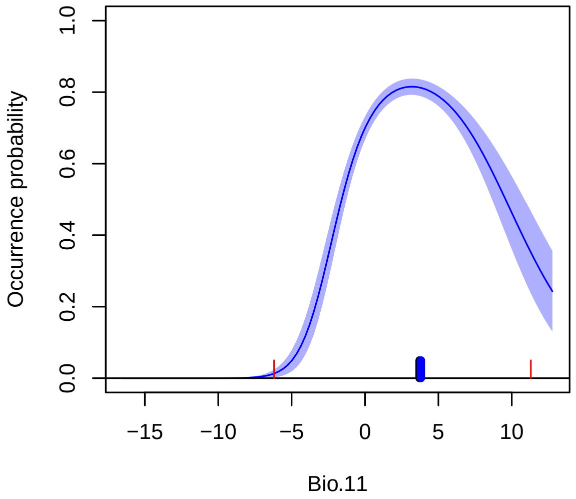

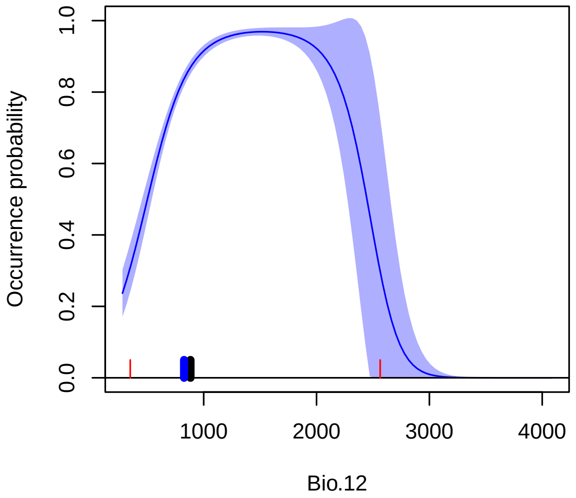

Response maps

Response maps (also referred to as partial effect maps). Each map represents how each predictor affects the occurrence probability. Predictor acronyms: Bio.10 = Mean temperature of warmest quarter [°C] within months 6 to 8, Bio.11 = Mean temperature of coldest quarter [°C] within months 12,1,2, Bio.12 = Annual precipitation sum [mm/m2], Bio.18 = Mean monthly precipitation amount of the warmest quarter [mm/m2] within months 6 to 8.

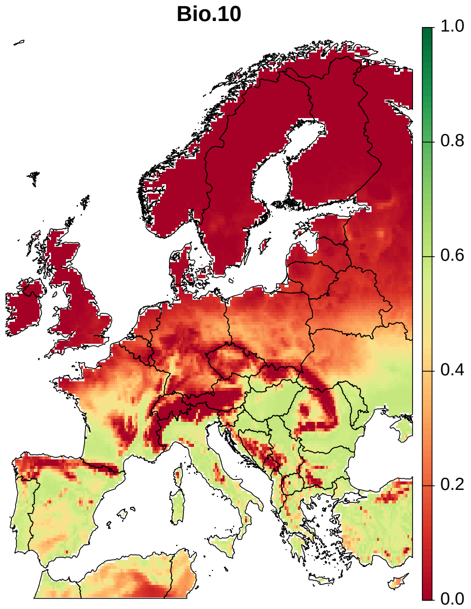

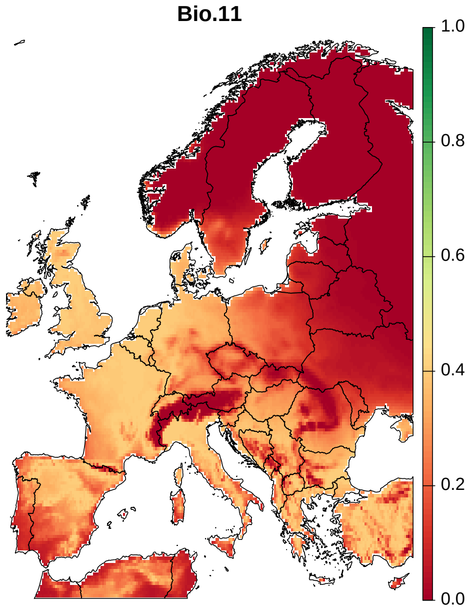

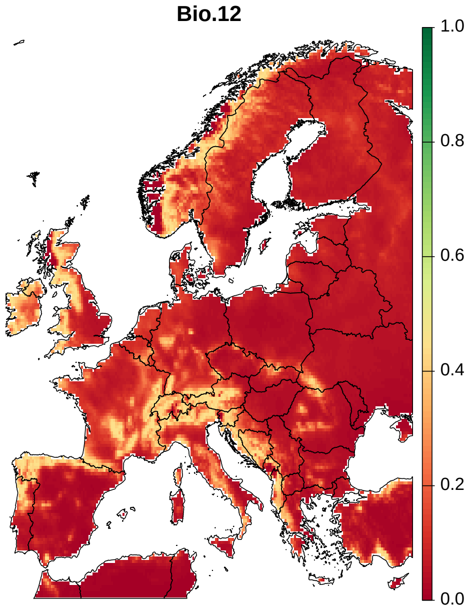

Model projections

Projection with plotted input data

Projection of species distribution model for reference period 1981-2010 over Europe. Occurrence probability ranges from 0 to 1 and is represented in dark red (low probability) to dark green (high probability). Input data used to calibrate the model is shown as presence points in magenta and absence points in black.

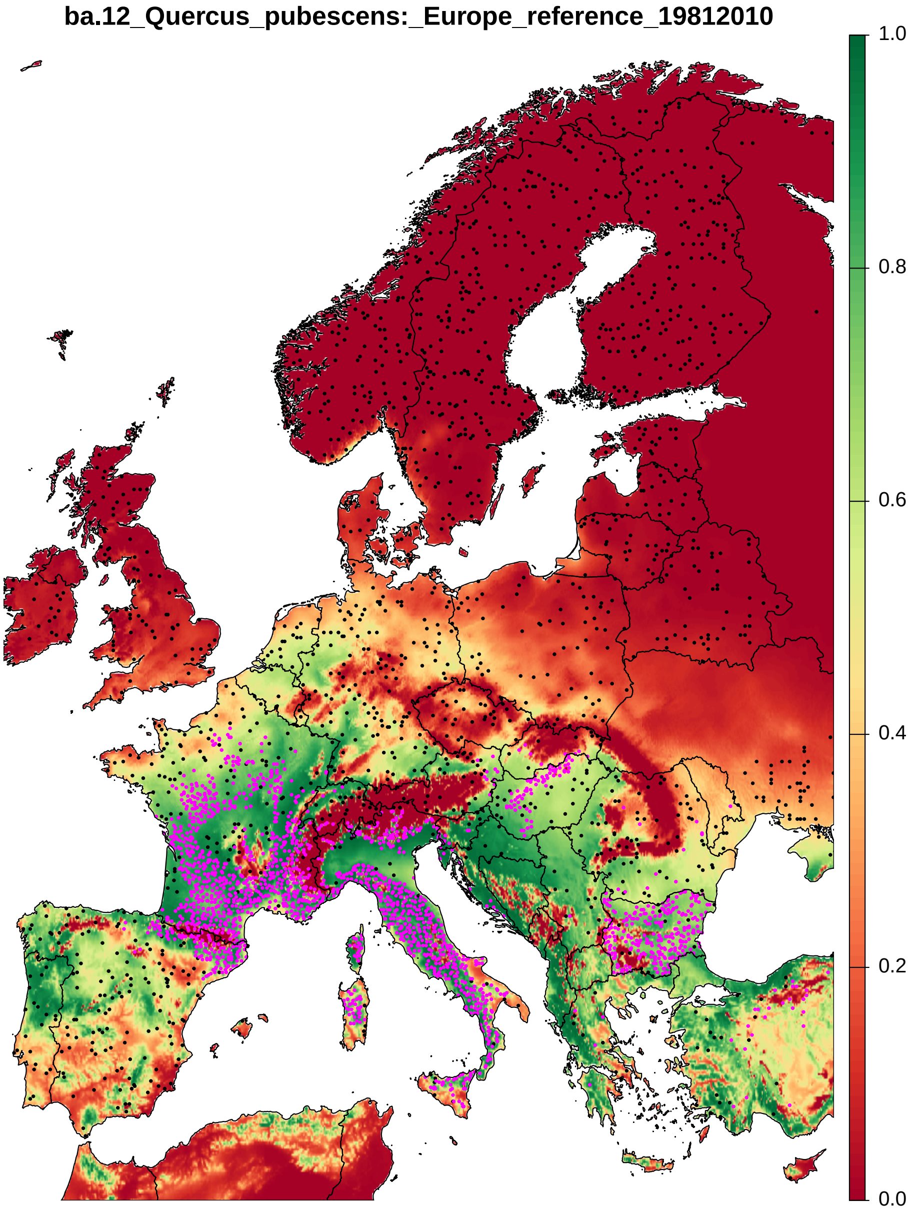

Projections

Projections of the species distribution models for reference period (1981-2010) and future scenarios RCP4.5 (2071-2100) and RCP8.5 (2071-2100) over Europe. Occurrence probabilities range from 0 to 1 and are represented from dark red (low probability) to dark green (high probability).

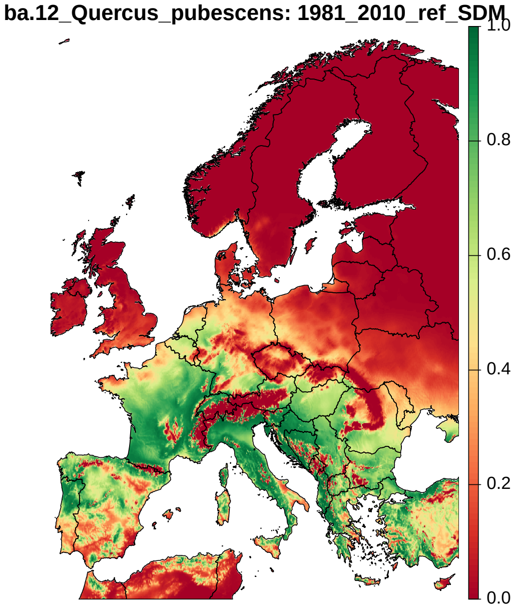

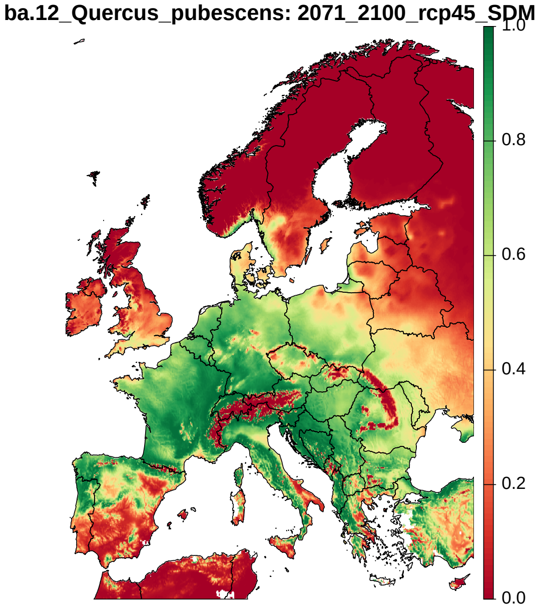

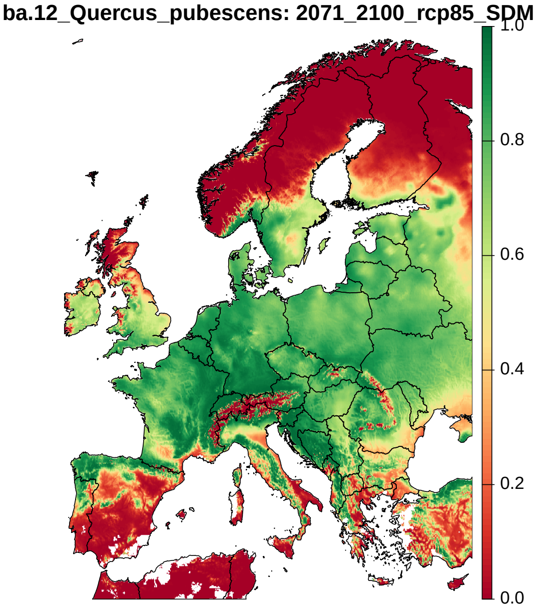

Climate risk classes

Maps of the climate risk classes. To estimate the distribution potential of each species as a mask for the SIMs, the continuous SDM outputs were categorized into three classes: low (yellow), medium (blue) and high climatic risk (red). The maps depict the risk classes in reference time (1981 to 2010), in climate scenario RCP4.5 (2071-2100) and RCP8.5 (2071-2100). To get an impression how well the thresholds fit to the data, presences (black) and absences (grey) were added on the reference map (top left). Refer to the legend and section "SDM thresholds" for the thresholds.

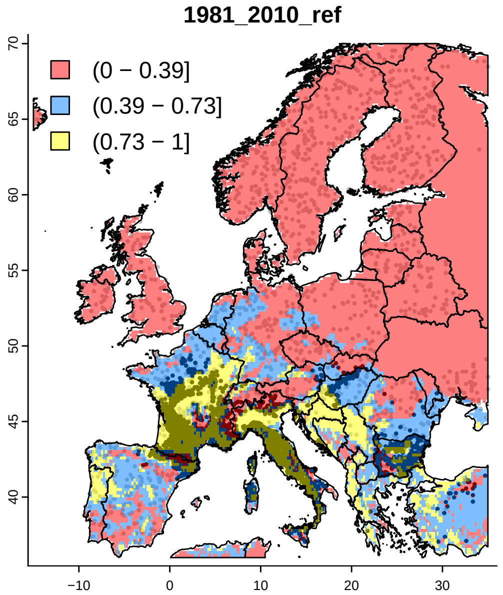

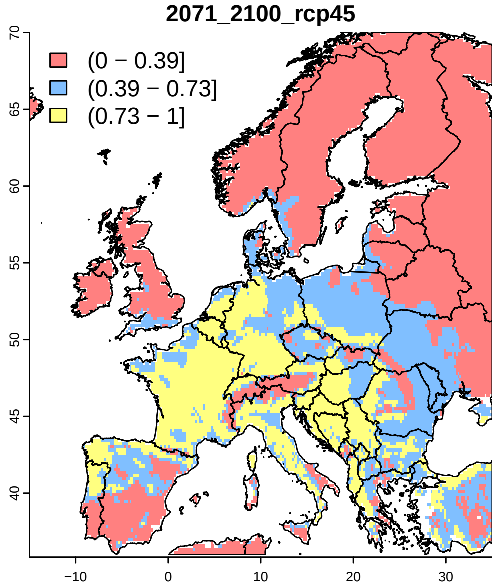

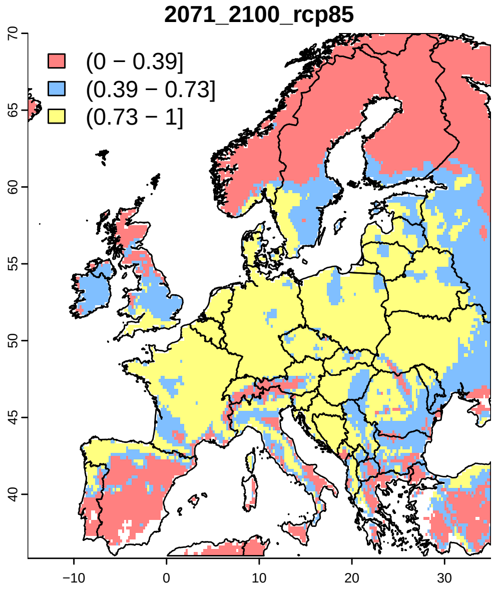

Quercus robur

Model statistics and evaluation

Summary

Predictor acronyms: Bio.10 = Mean temperature of warmest quarter [°C] within months 6 to 8, Bio.11 = Mean temperature of coldest quarter [°C] within months 12,1,2, Bio.12 = Annual precipitation sum [mm/m2], Bio.18 = Mean monthly precipitation amount of the warmest quarter [mm/m2] within months 6 to 8.

```
##
## Family: binomial
## Link function: logit
##
## Formula:
## ba.10 ~ s(Bio.10, k = 3) + s(Bio.11, k = 3) + s(Bio.18, k = 3)
##
## Parametric coefficients:
##             Estimate Std. Error z value Pr(>|z|)
## (Intercept) -1.17365    0.04672  -25.12   <2e-16 ***
## ---
## Signif. codes:  0 '***' 0.001 '**' 0.01 '*' 0.05 '.' 0.1 ' ' 1
##
## Approximate significance of smooth terms:
##             edf Ref.df Chi.sq p-value
## s(Bio.10) 1.999      2   1074  <2e-16 ***
## s(Bio.11) 1.997      2   1824  <2e-16 ***
## s(Bio.18) 1.997      2    590  <2e-16 ***
## ---
## Signif. codes:  0 '***' 0.001 '**' 0.01 '*' 0.05 '.' 0.1 ' ' 1
##
## R-sq.(adj) =  0.515   Deviance explained = 45.2%
## -REML =   4938  Scale est. = 1         n = 12938
```

Evaluation parameter

Model performance was assessed using four statistical parameters: the area under the receiver operating characteristic curve (AUC), the true skill statistic (TSS), sensitivity (probability of the model to correctly predict a true presence) and specificity (probability of the model to correctly predict a true absence).

```
##     Species_name  AUC       TSS sensitivity specificity
## tp Quercus robur 0.90 0.6563611   0.8828258   0.7735353
```

Response curves and response maps

Response curves

Response curves (also known as effect curves) give an overview of the climatic niche of a species by relating the occurrence probability to corresponding climatic values. Predictor acronyms: Bio.10 = Mean temperature of warmest quarter [°C] within months 6 to 8, Bio.11 = Mean temperature of coldest quarter [°C] within months 12,1,2, Bio.12 = Annual precipitation sum [mm/m2], Bio.18 = Mean monthly precipitation amount of the warmest quarter [mm/m2] within months 6 to 8. Lines on the x-axis mark the upper and lower limit of the used presences (red), the mean (bold black) and the median (bold blue).

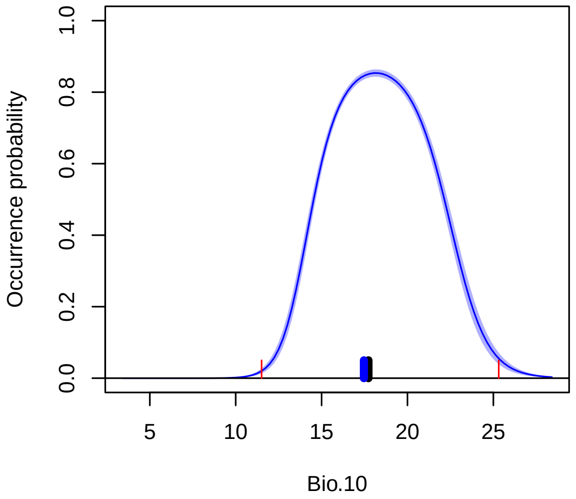

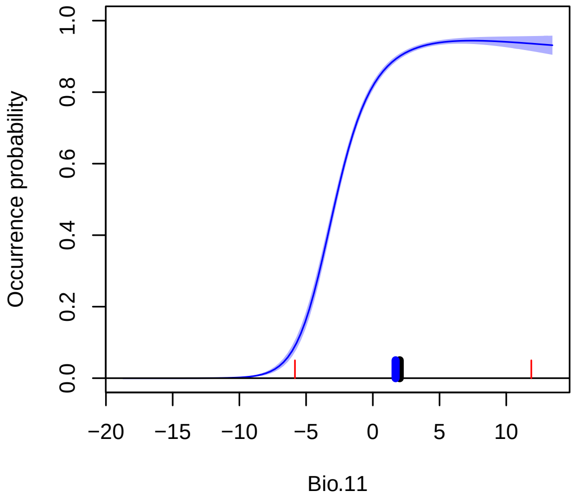

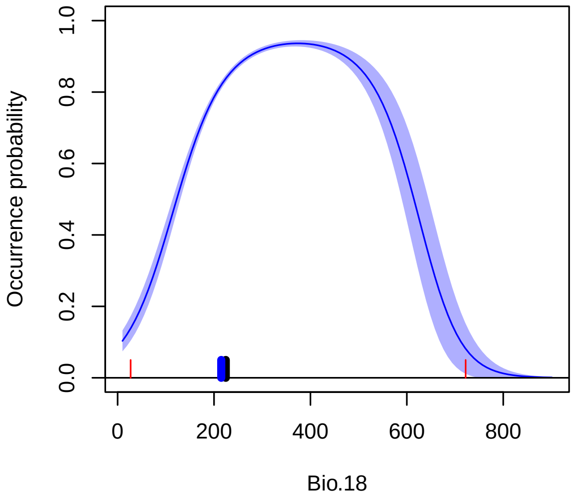

Response maps

Response maps (also referred to as partial effect maps). Each map represents how each predictor affects the occurrence probability. Predictor acronyms: Bio.10 = Mean temperature of warmest quarter [°C] within months 6 to 8, Bio.11 = Mean temperature of coldest quarter [°C] within months 12,1,2, Bio.12 = Annual precipitation sum [mm/m2], Bio.18 = Mean monthly precipitation amount of the warmest quarter [mm/m2] within months 6 to 8.

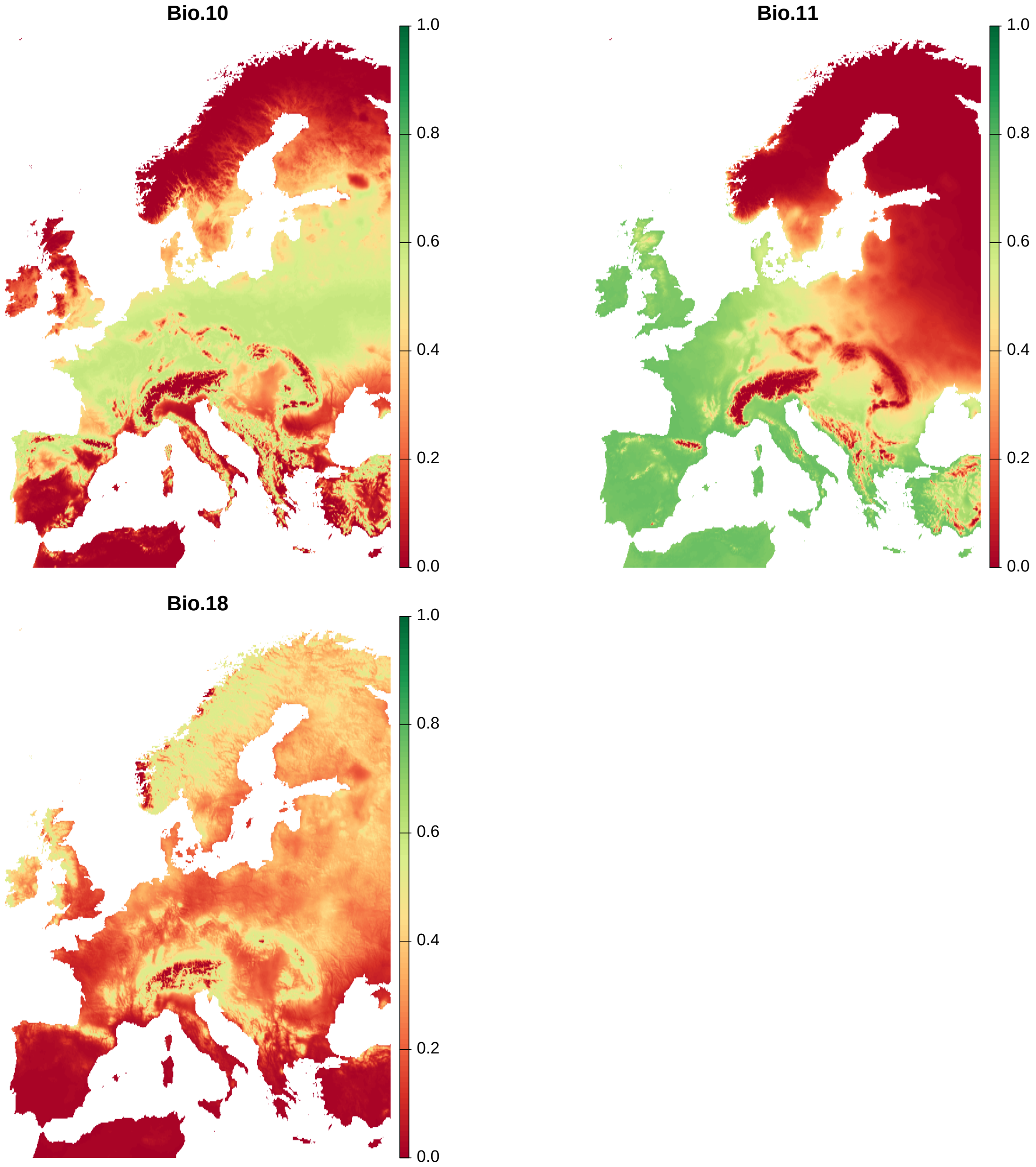

Model projections

Projection with plotted input data

Projection of species distribution model for reference period 1981-2010 over Europe. Occurrence probability ranges from 0 to 1 and is represented in dark red (low probability) to dark green (high probability). Input data used to calibrate the model is shown as presence points in magenta and absence points in black.

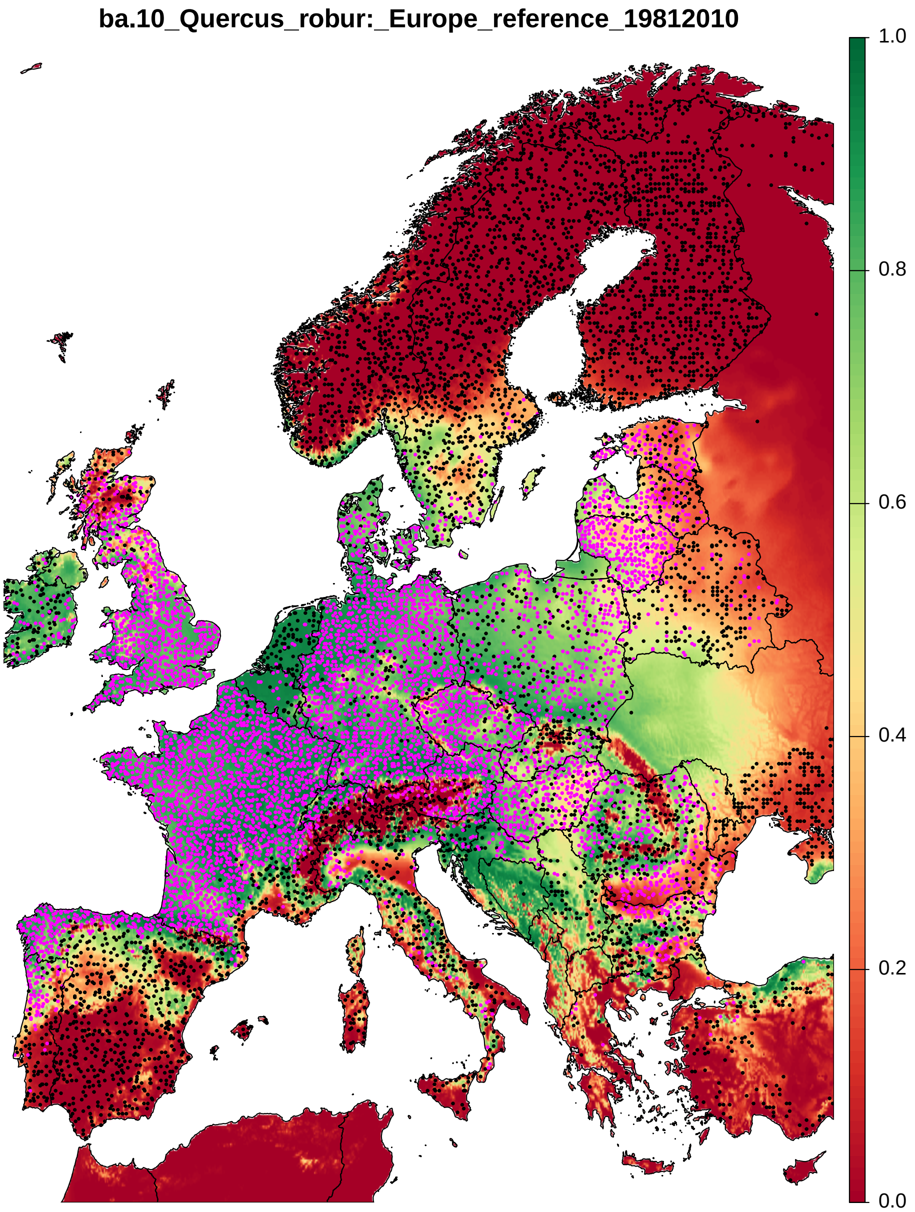

Projections

Projections of the species distribution models for reference period (1981-2010) and future scenarios RCP4.5 (2071-2100) and RCP8.5 (2071-2100) over Europe. Occurrence probabilities range from 0 to 1 and are represented from dark red (low probability) to dark green (high probability).

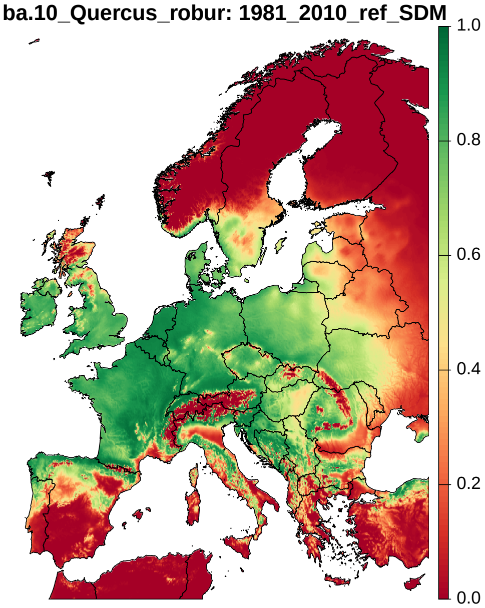

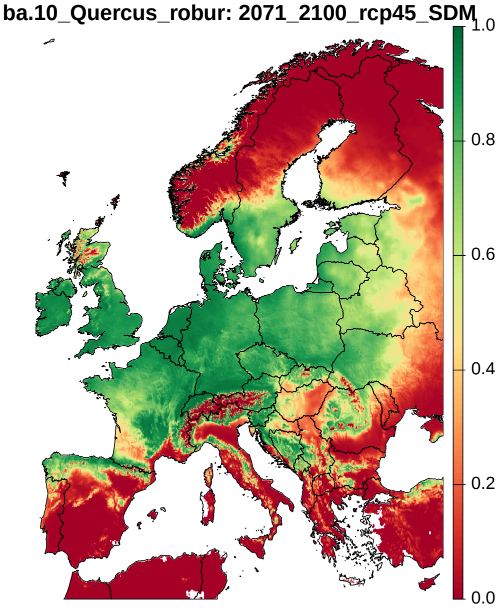

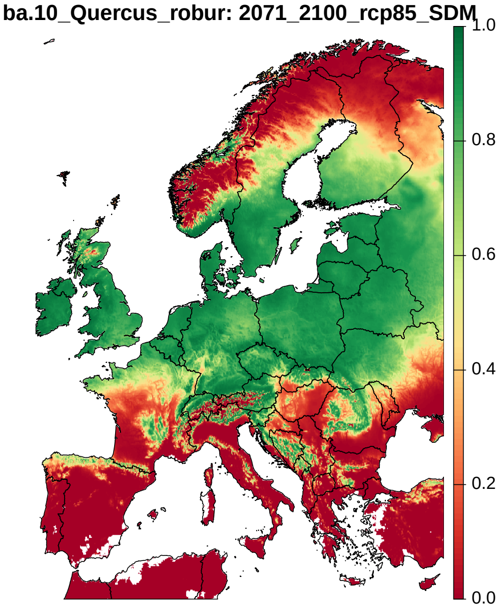

Climate risk classes

Maps of the climate risk classes. To estimate the distribution potential of each species as a mask for the SIMs, the continuous SDM outputs were categorized into three classes: low (yellow), medium (blue) and high climatic risk (red). The maps depict the risk classes in reference time (1981 to 2010), in climate scenario RCP4.5 (2071-2100) and RCP8.5 (2071-2100). To get an impression how well the thresholds fit to the data, presences (black) and absences (grey) were added on the reference map (top left). Refer to the legend and section “SDM thresholds” for the thresholds.

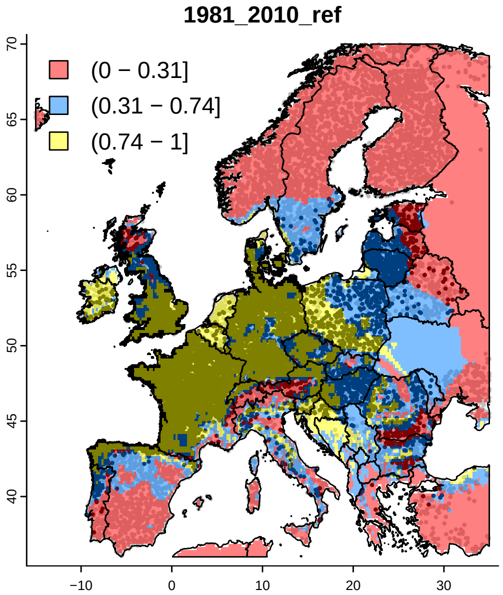

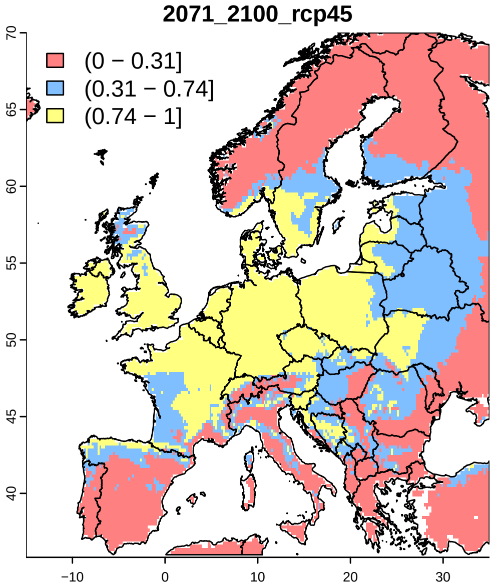

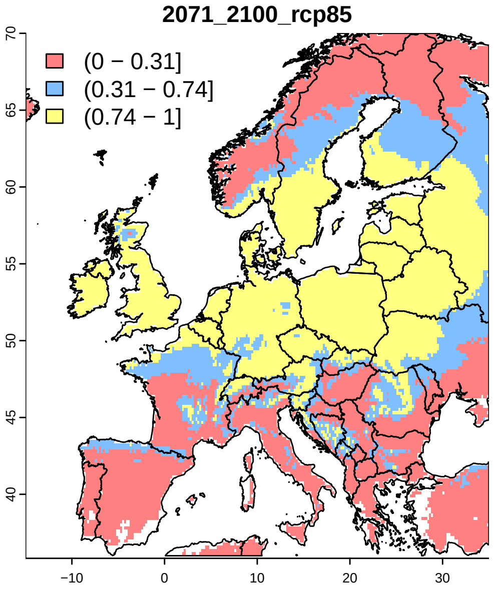

Quercus rubra

Model statistics and evaluation

Summary

Predictor acronyms: Bio.10 = Mean temperature of warmest quarter [°C] within months 6 to 8, Bio.11 = Mean temperature of coldest quarter [°C] within months 12,1,2, Bio.12 = Annual precipitation sum [mm/m2], Bio.18 = Mean monthly precipitation amount of the warmest quarter [mm/m2] within months 6 to 8.

```
##
## Family: binomial
## Link function: logit
##
## Formula:
## ba.13 ~ s(Bio.10, k = 3) + s(Bio.11, k = 3) + s(Bio.18, k = 3)
##
## Parametric coefficients:
##             Estimate Std. Error z value Pr(>|z|)
## (Intercept)  -2.4215     0.1651  -14.67   <2e-16 ***
## ---
## Signif. codes:  0 '***' 0.001 '**' 0.01 '*' 0.05 '.' 0.1 ' ' 1
##
## Approximate significance of smooth terms:
##             edf Ref.df Chi.sq p-value
## s(Bio.10) 1.995      2  210.3  <2e-16 ***
## s(Bio.11) 1.993      2  249.4  <2e-16 ***
## s(Bio.18) 1.986      2  114.5  <2e-16 ***
## ---
## Signif. codes:  0 '***' 0.001 '**' 0.01 '*' 0.05 '.' 0.1 ' ' 1
##
## R-sq.(adj) =  0.525   Deviance explained = 46.7%
## -REML = 981.51  Scale est. = 1         n = 2616
```

Evaluation parameter

Model performance was assessed using four statistical parameters: the area under the receiver operating characteristic curve (AUC), the true skill statistic (TSS), sensitivity (probability of the model to correctly predict a true presence) and specificity (probability of the model to correctly predict a true absence).

```
##     Species_name  AUC       TSS sensitivity specificity
## tp Quercus rubra 0.90 0.6796636    0.925841   0.7538226
```

Response curves and response maps

Response curves

Response curves (also known as effect curves) give an overview of the climatic niche of a species by relating the occurrence probability to corresponding climatic values. Predictor acronyms: Bio.10 = Mean temperature of warmest quarter [°C] within months 6 to 8, Bio.11 = Mean temperature of coldest quarter [°C] within months 12,1,2, Bio.12 = Annual precipitation sum [mm/m2], Bio.18 = Mean monthly precipitation amount of the warmest quarter [mm/m2] within months 6 to 8. Lines on the x-axis mark the upper and lower limit of the used presences (red), the mean (bold black) and the median (bold blue).

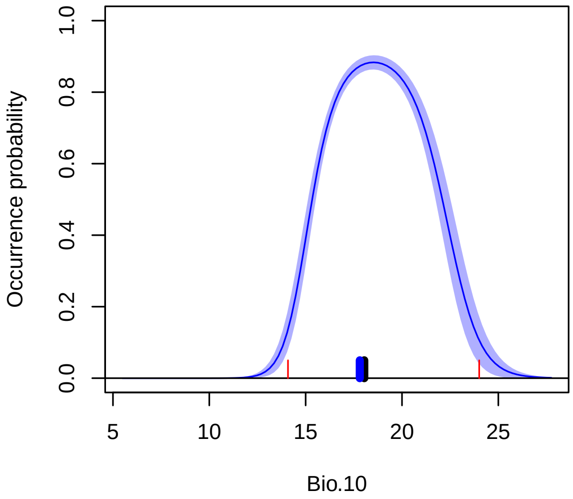

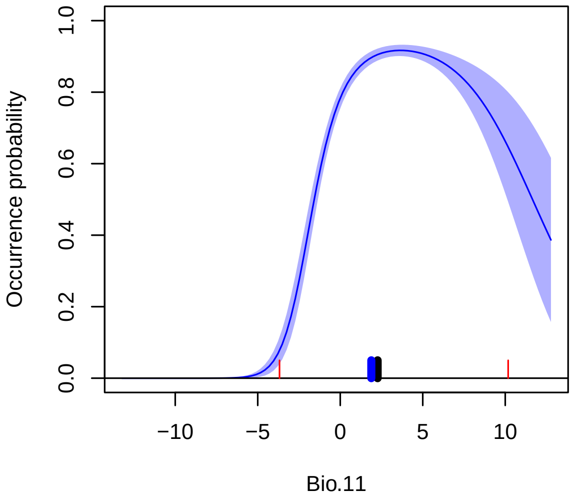

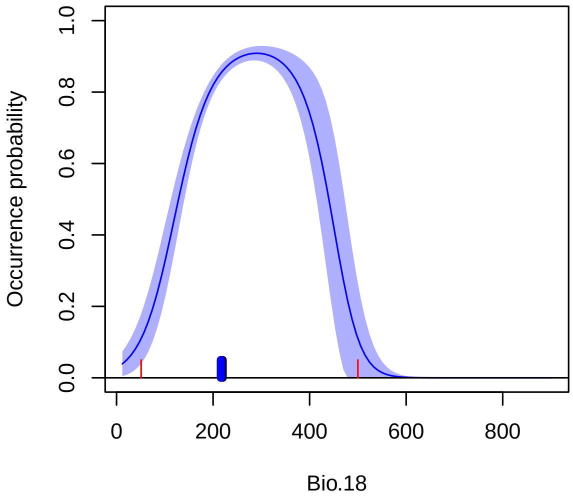

Response maps

Response maps (also referred to as partial effect maps). Each map represents how each predictor affects the occurrence probability. Predictor acronyms: Bio.10 = Mean temperature of warmest quarter [°C] within months 6 to 8, Bio.11 = Mean temperature of coldest quarter [°C] within months 12,1,2, Bio.12 = Annual precipitation sum [mm/m2], Bio.18 = Mean monthly precipitation amount of the warmest quarter [mm/m2] within months 6 to 8.

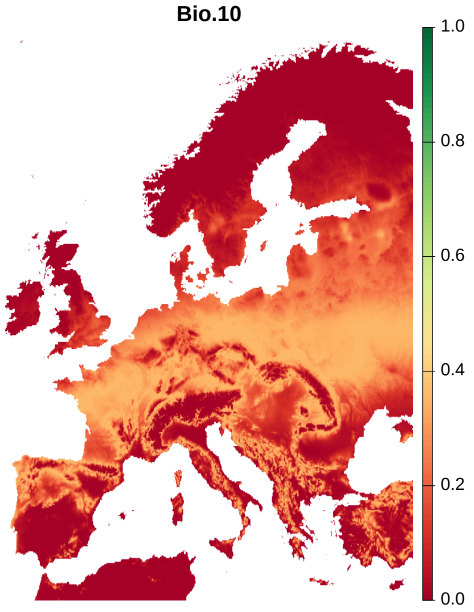

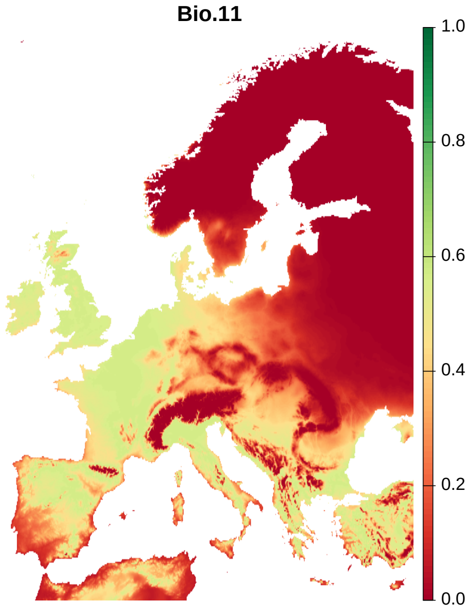

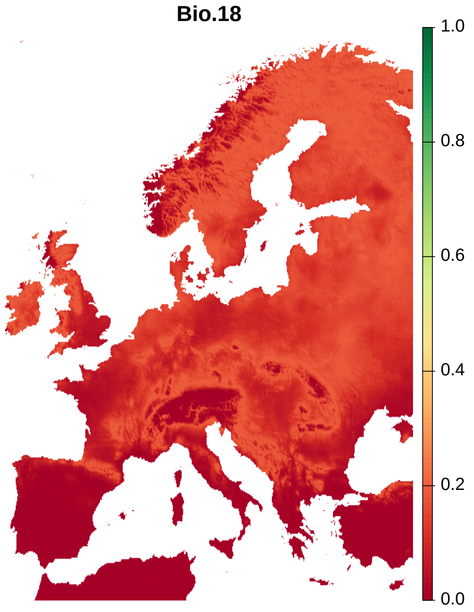

Model projections

Projection with plotted input data

Projection of species distribution model for reference period 1981-2010 over Europe. Occurrence probability ranges from 0 to 1 and is represented in dark red (low probability) to dark green (high probability). Input data used to calibrate the model is shown as presence points in magenta and absence points in black.

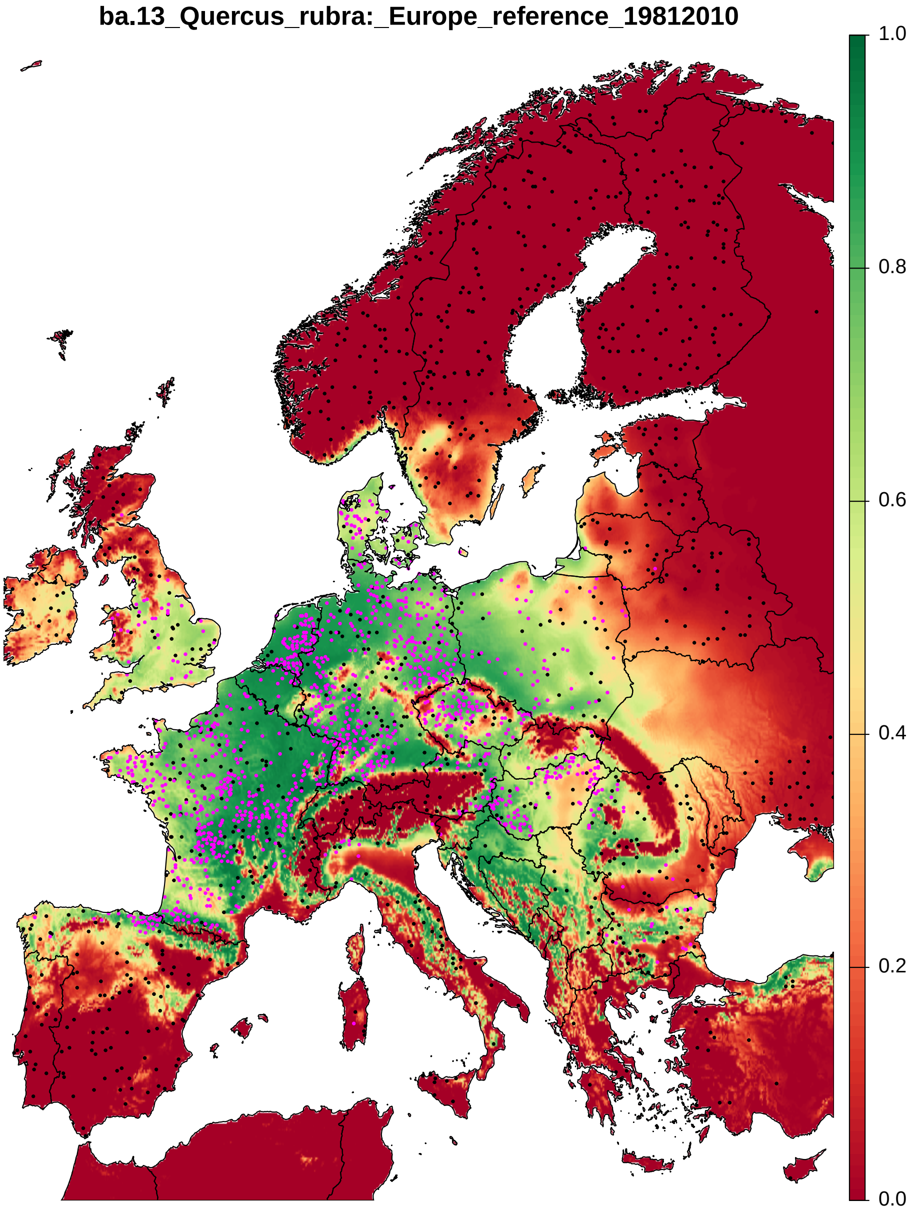

Projections

Projections of the species distribution models for reference period (1981-2010) and future scenarios RCP4.5 (2071-2100) and RCP8.5 (2071-2100) over Europe. Occurrence probabilities range from 0 to 1 and are represented from dark red (low probability) to dark green (high probability).

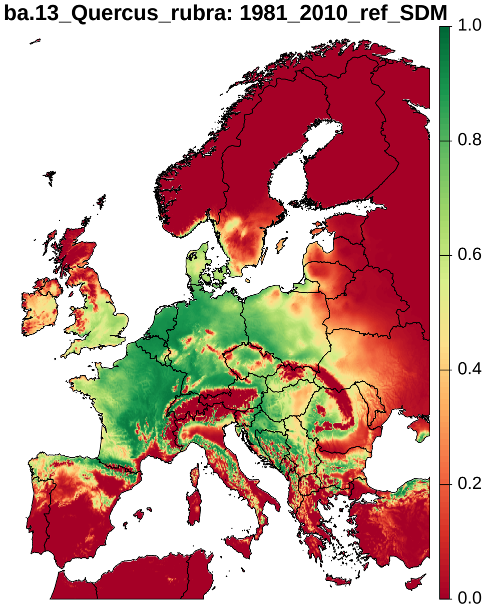

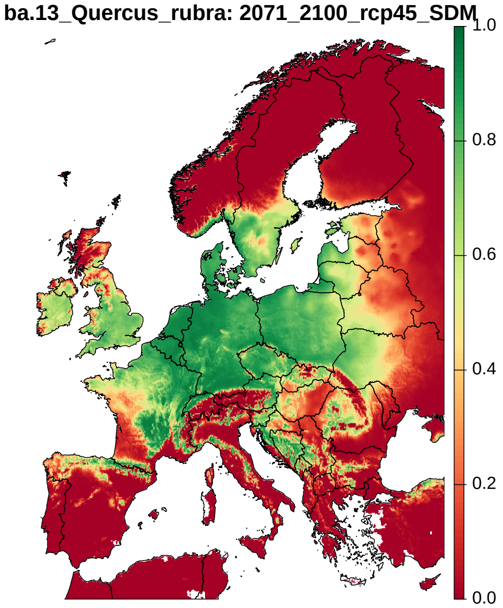

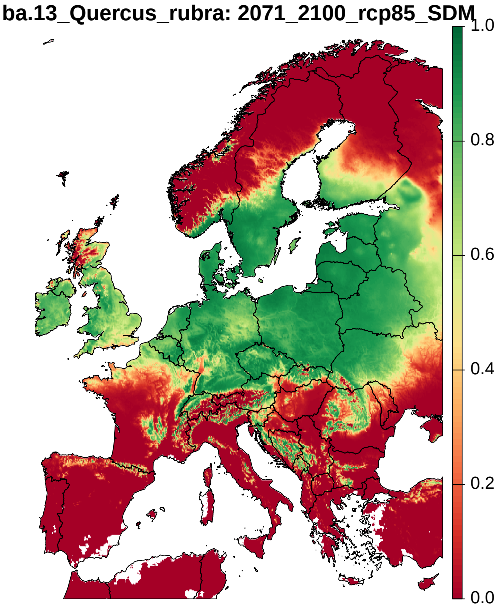

Climate risk classes

Maps of the climate risk classes. To estimate the distribution potential of each species as a mask for the SIMs, the continuous SDM outputs were categorized into three classes: low (yellow), medium (blue) and high climatic risk (red). The maps depict the risk classes in reference time (1981 to 2010), in climate scenario RCP4.5 (2071-2100) and RCP8.5 (2071-2100). To get an impression how well the thresholds fit to the data, presences (black) and absences (grey) were added on the reference map (top left). Refer to the legend and section “SDM thresholds” for the thresholds.

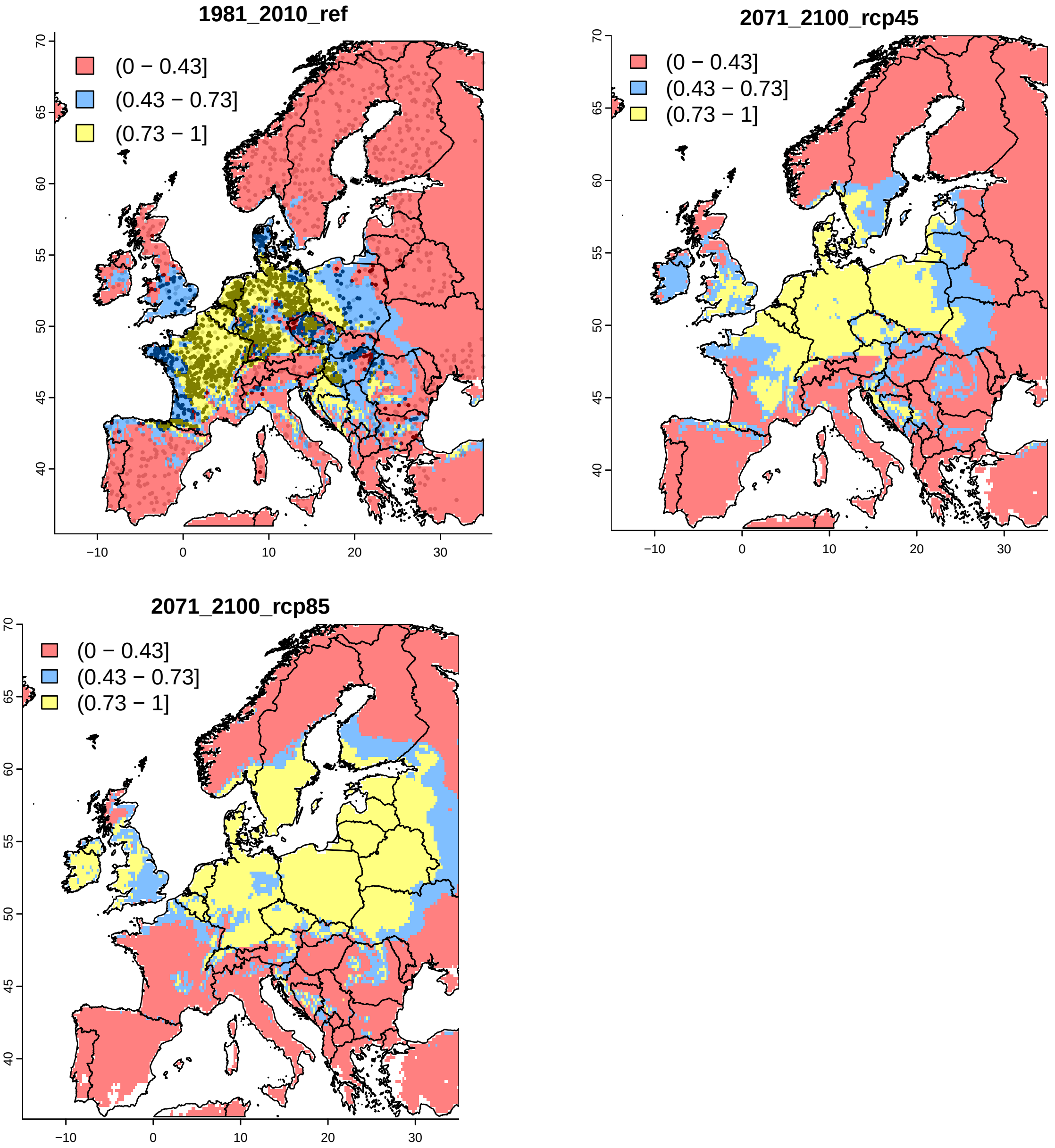

Robinia pseudoacacia

Model statistics and evaluation

Summary

Predictor acronyms: Bio.10 = Mean temperature of warmest quarter [°C] within months 6 to 8, Bio.11 = Mean temperature of coldest quarter [°C] within months 12,1,2, Bio.12 = Annual precipitation sum [mm/m2], Bio.18 = Mean monthly precipitation amount of the warmest quarter [mm/m2] within months 6 to 8.

```
##
## Family: binomial
## Link function: logit
##
## Formula:
## ba.32 ~ s(Bio.10, k = 3) + s(Bio.11, k = 3) + s(Bio.18, k = 3)
##
## Parametric coefficients:
##             Estimate Std. Error z value Pr(>|z|)
## (Intercept)  -3.8948     0.1667  -23.37   <2e-16 ***
## ---
## Signif. codes:  0 '***' 0.001 '**' 0.01 '*' 0.05 '.' 0.1 ' ' 1
##
## Approximate significance of smooth terms:
##             edf Ref.df Chi.sq p-value
## s(Bio.10) 1.997      2  706.9  <2e-16 ***
## s(Bio.11) 1.997      2  476.6  <2e-16 ***
## s(Bio.18) 1.993      2  335.0  <2e-16 ***
## ---
## Signif. codes:  0 '***' 0.001 '**' 0.01 '*' 0.05 '.' 0.1 ' ' 1
##
## R-sq.(adj) =  0.632   Deviance explained =   56%
## -REML = 1697.1  Scale est. = 1         n = 5508
```

Evaluation parameter

Model performance was assessed using four statistical parameters: the area under the receiver operating characteristic curve (AUC), the true skill statistic (TSS), sensitivity (probability of the model to correctly predict a true presence) and specificity (probability of the model to correctly predict a true absence).

```
##            Species_name  AUC       TSS sensitivity specificity
## tp Robinia pseudoacacia 0.94 0.7523602   0.9270153    0.825345
```

Response curves and response maps

Response curves

Response curves (also known as effect curves) give an overview of the climatic niche of a species by relating the occurrence probability to corresponding climatic values. Predictor acronyms: Bio.10 = Mean temperature of warmest quarter [°C] within months 6 to 8, Bio.11 = Mean temperature of coldest quarter [°C] within months 12,1,2, Bio.12 = Annual precipitation sum [mm/m2], Bio.18 = Mean monthly precipitation amount of the warmest quarter [mm/m2] within months 6 to 8. Lines on the x-axis mark the upper and lower limit of the used presences (red), the mean (bold black) and the median (bold blue).

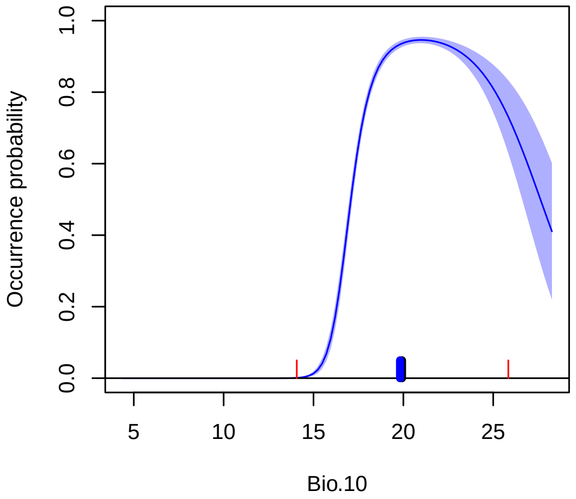

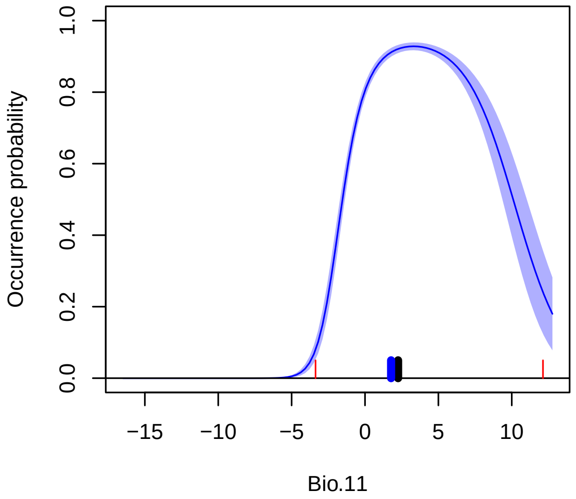

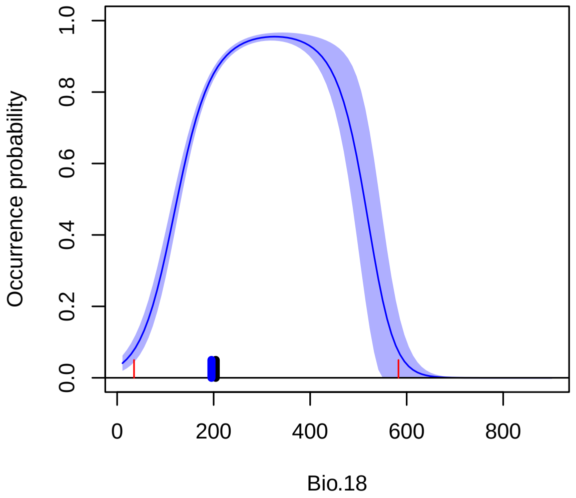

Response maps

Response maps (also referred to as partial effect maps). Each map represents how each predictor affects the occurrence probability. Predictor acronyms: Bio.10 = Mean temperature of warmest quarter [°C] within months 6 to 8, Bio.11 = Mean temperature of coldest quarter [°C] within months 12,1,2, Bio.12 = Annual precipitation sum [mm/m2], Bio.18 = Mean monthly precipitation amount of the warmest quarter [mm/m2] within months 6 to 8.

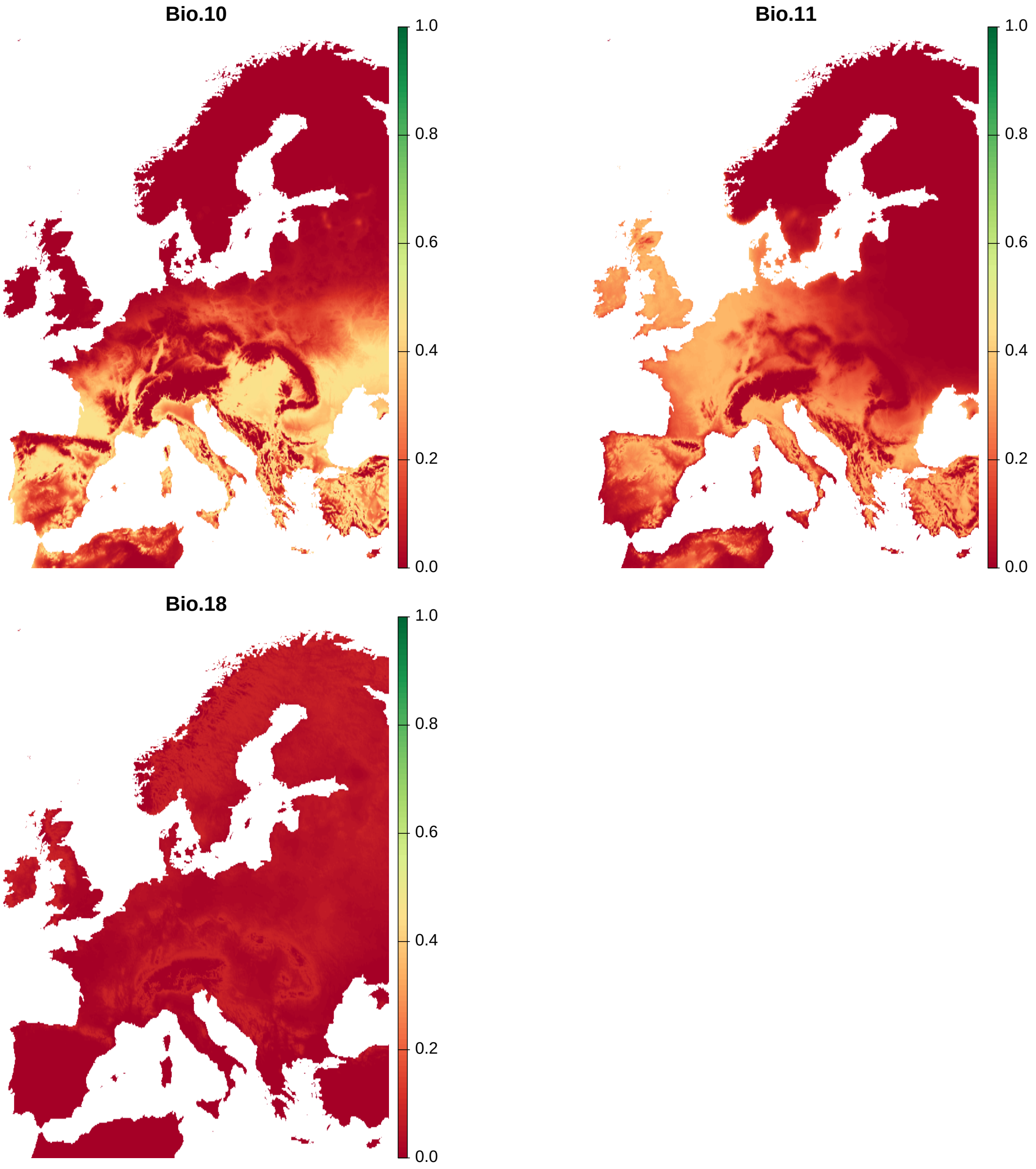

Model projections

Projection with plotted input data

Projection of species distribution model for reference period 1981-2010 over Europe. Occurrence probability ranges from 0 to 1 and is represented in dark red (low probability) to dark green (high probability). Input data used to calibrate the model is shown as presence points in magenta and absence points in black.

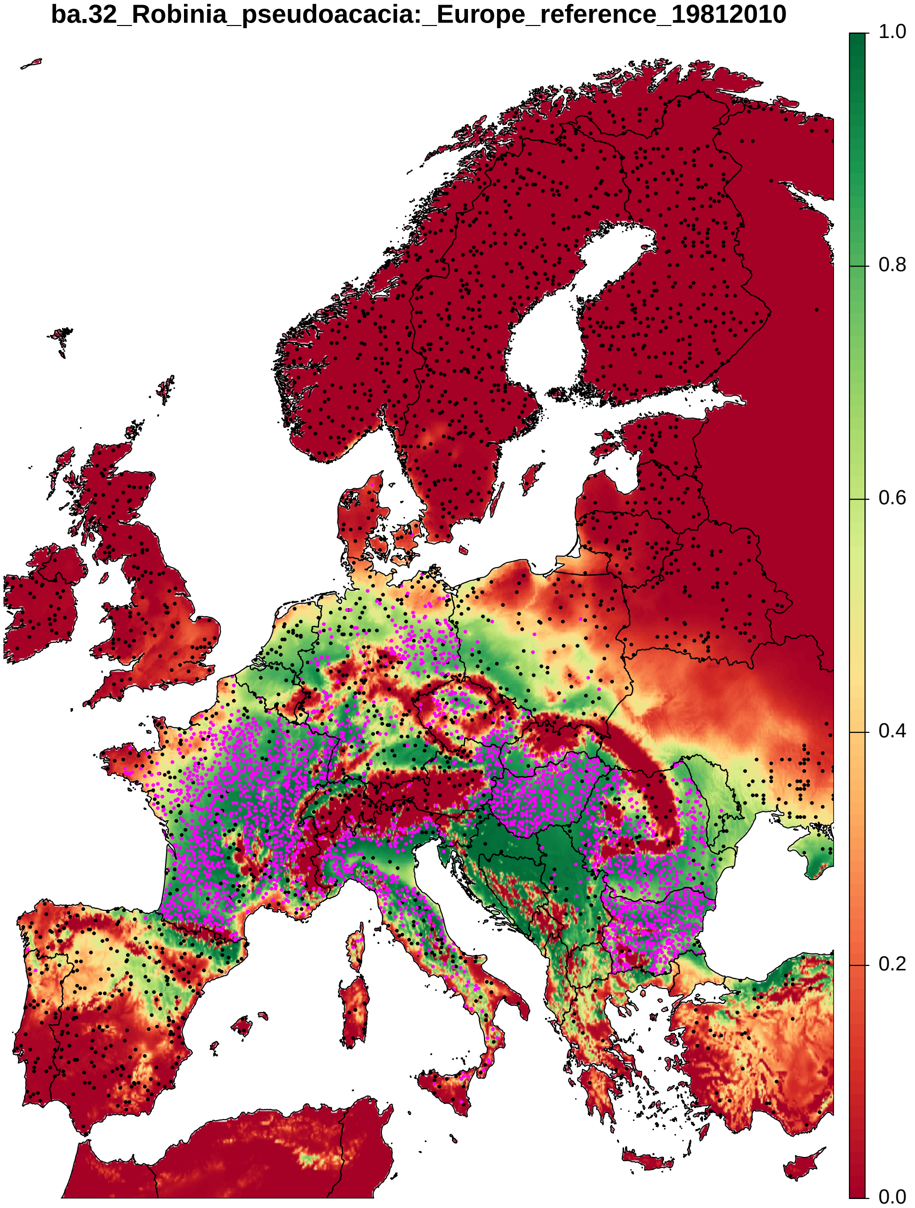

Projections

Projections of the species distribution models for reference period (1981-2010) and future scenarios RCP4.5 (2071-2100) and RCP8.5 (2071-2100) over Europe. Occurrence probabilities range from 0 to 1 and are represented from dark red (low probability) to dark green (high probability).

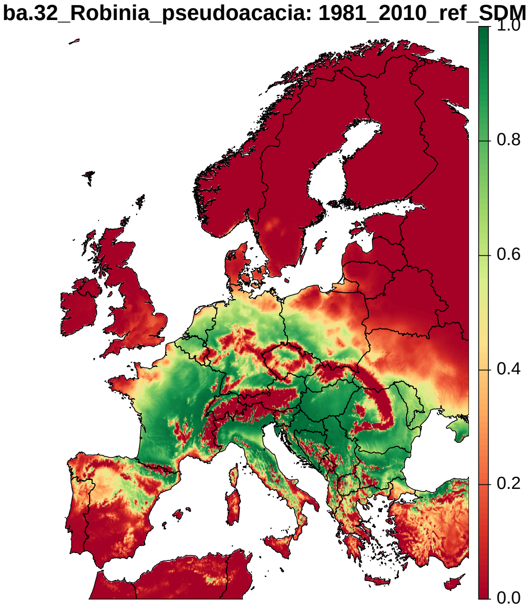

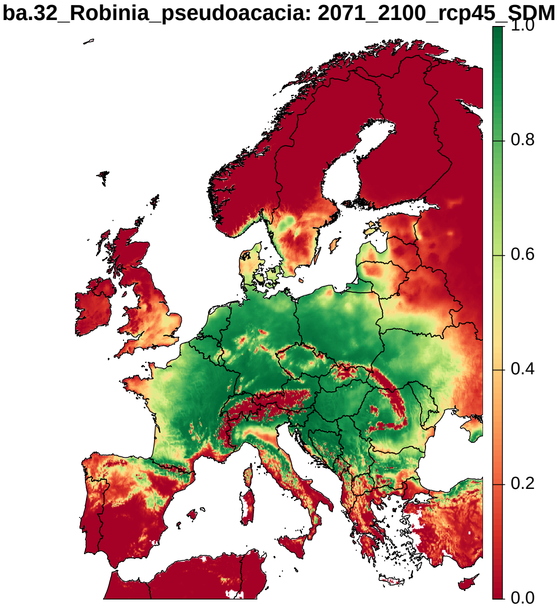

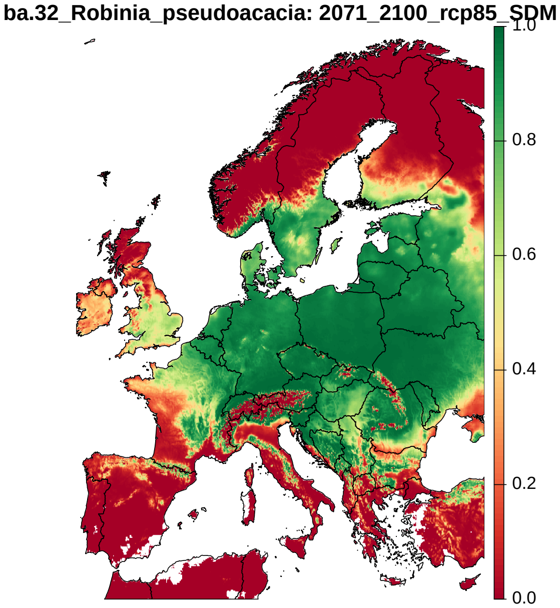

Climate risk classes

Maps of the climate risk classes. To estimate the distribution potential of each species as a mask for the SIMs, the continuous SDM outputs were categorized into three classes: low (yellow), medium (blue) and high climatic risk (red). The maps depict the risk classes in reference time (1981 to 2010), in climate scenario RCP4.5 (2071-2100) and RCP8.5 (2071-2100). To get an impression how well the thresholds fit to the data, presences (black) and absences (grey) were added on the reference map (top left). Refer to the legend and section "SDM thresholds" for the thresholds.

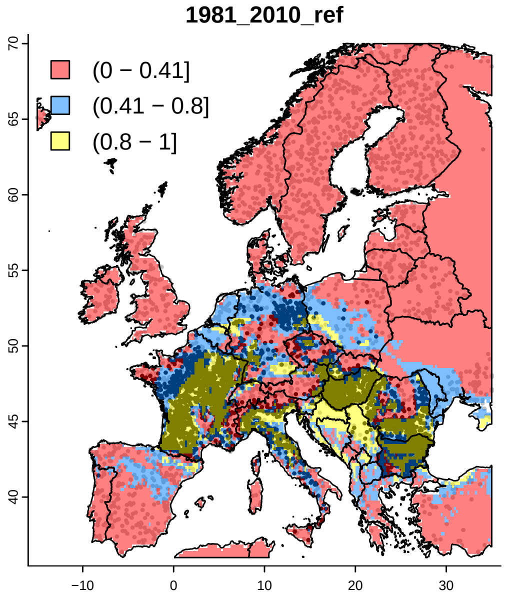

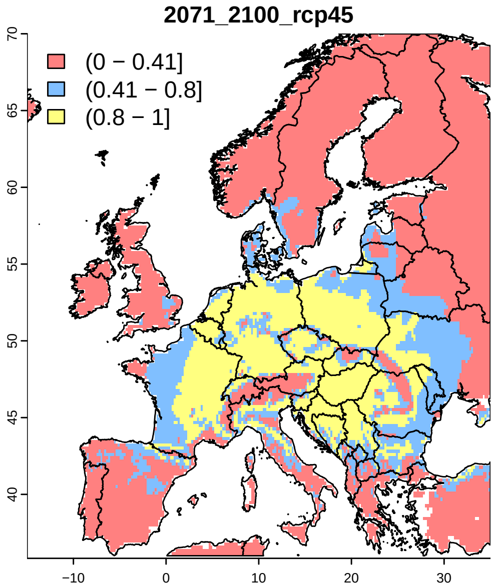

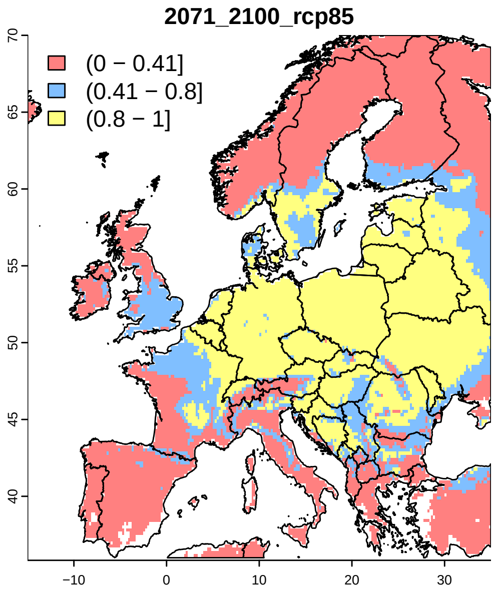

Sorbus aucuparia

Model statistics and evaluation

Summary

Predictor acronyms: Bio.10 = Mean temperature of warmest quarter [°C] within months 6 to 8, Bio.11 = Mean temperature of coldest quarter [°C] within months 12,1,2, Bio.12 = Annual precipitation sum [mm/m2], Bio.18 = Mean monthly precipitation amount of the warmest quarter [mm/m2] within months 6 to 8.

```
##
## Family: binomial
## Link function: logit
##
## Formula:
## ba.28 ~ s(Bio.10, k = 3) + s(Bio.11, k = 3) + s(Bio.18, k = 3)
##
## Parametric coefficients:
##             Estimate Std. Error z value Pr(>|z|)
## (Intercept) -0.50608    0.03727  -13.58   <2e-16 ***
## ---
## Signif. codes:  0 '***' 0.001 '**' 0.01 '*' 0.05 '.' 0.1 ' ' 1
##
## Approximate significance of smooth terms:
##             edf Ref.df Chi.sq p-value
## s(Bio.10) 1.995  2.000  773.0  <2e-16 ***
## s(Bio.11) 1.999  2.000  830.1  <2e-16 ***
## s(Bio.18) 1.725  1.924  110.0  <2e-16 ***
## ---
## Signif. codes:  0 '***' 0.001 '**' 0.01 '*' 0.05 '.' 0.1 ' ' 1
##
## R-sq.(adj) =  0.404   Deviance explained = 33.4%
## -REML = 4147.7  Scale est. = 1         n = 8952
```

Evaluation parameter

Model performance was assessed using four statistical parameters: the area under the receiver operating characteristic curve (AUC), the true skill statistic (TSS), sensitivity (probability of the model to correctly predict a true presence) and specificity (probability of the model to correctly predict a true absence).

```
##        Species_name  AUC       TSS sensitivity specificity
## tp Sorbus aucuparia 0.86 0.5592046   0.8608132   0.6983914
```

Response curves and response maps

Response curves

Response curves (also known as effect curves) give an overview of the climatic niche of a species by relating the occurrence probability to corresponding climatic values. Predictor acronyms: Bio.10 = Mean temperature of warmest quarter [°C] within months 6 to 8, Bio.11 = Mean temperature of coldest quarter [°C] within months 12,1,2, Bio.12 = Annual precipitation sum [mm/m2], Bio.18 = Mean monthly precipitation amount of the warmest quarter [mm/m2] within months 6 to 8. Lines on the x-axis mark the upper and lower limit of the used presences (red), the mean (bold black) and the median (bold blue).

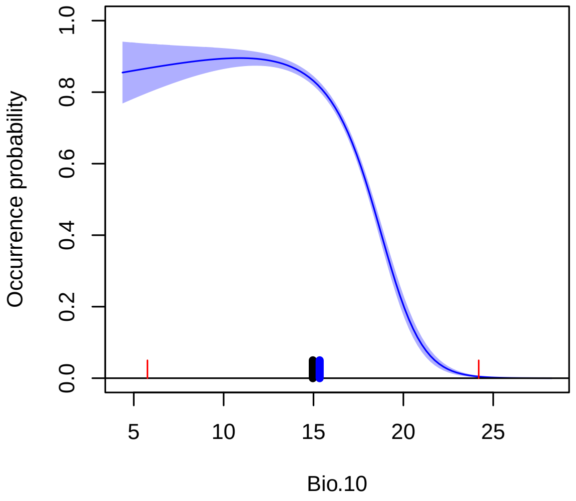

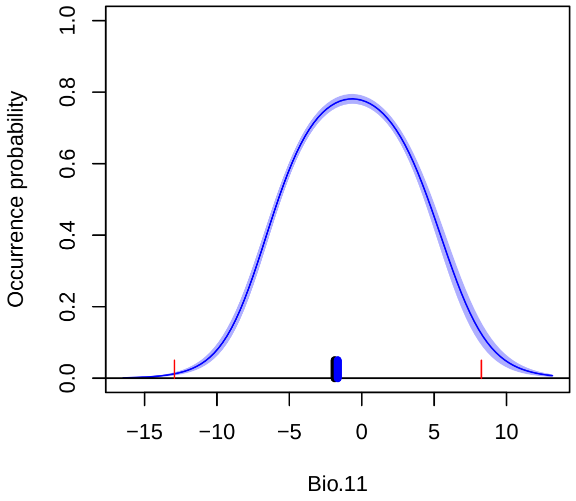

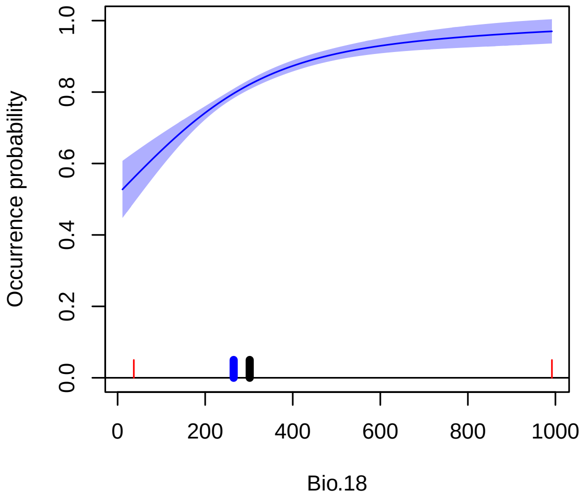

Response maps

Response maps (also referred to as partial effect maps). Each map represents how each predictor affects the occurrence probability. Predictor acronyms: Bio.10 = Mean temperature of warmest quarter [°C] within months 6 to 8, Bio.11 = Mean temperature of coldest quarter [°C] within months 12,1,2, Bio.12 = Annual precipitation sum [mm/m2], Bio.18 = Mean monthly precipitation amount of the warmest quarter [mm/m2] within months 6 to 8.

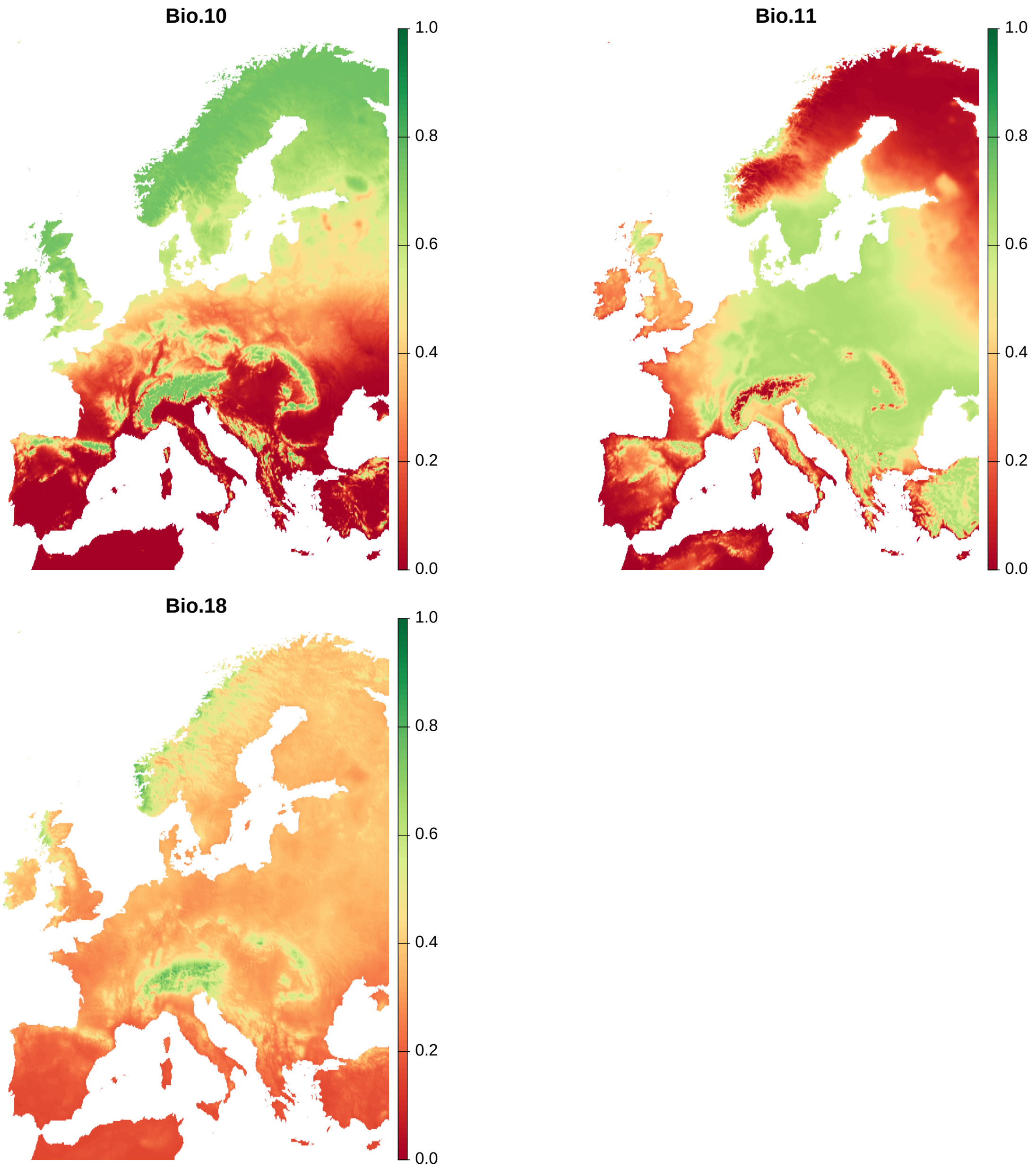

Model projections

Projection with plotted input data

Projection of species distribution model for reference period 1981-2010 over Europe. Occurrence probability ranges from 0 to 1 and is represented in dark red (low probability) to dark green (high probability). Input data used to calibrate the model is shown as presence points in magenta and absence points in black.

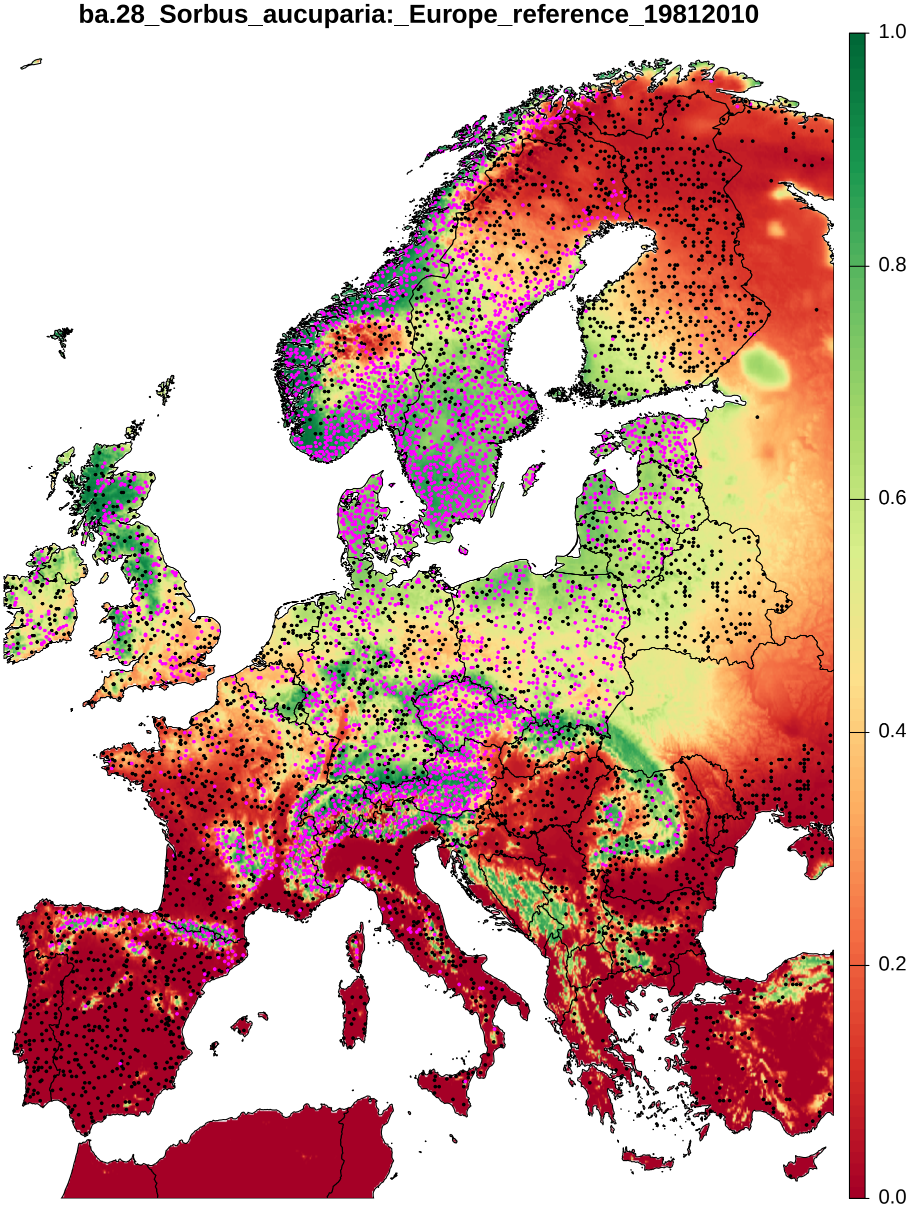

Projections

Projections of the species distribution models for reference period (1981-2010) and future scenarios RCP4.5 (2071-2100) and RCP8.5 (2071-2100) over Europe. Occurrence probabilities range from 0 to 1 and are represented from dark red (low probability) to dark green (high probability).

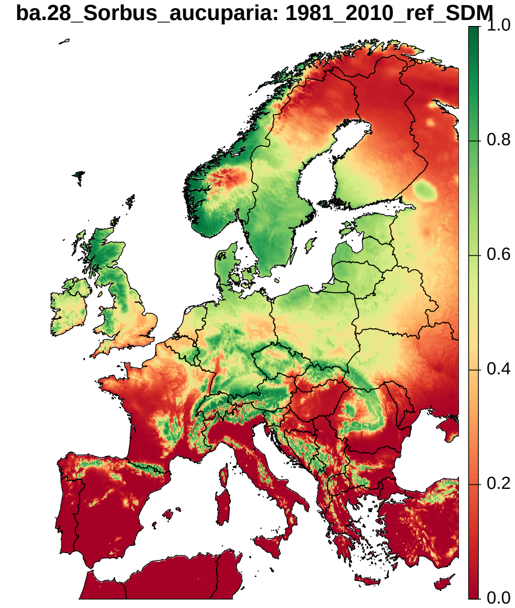

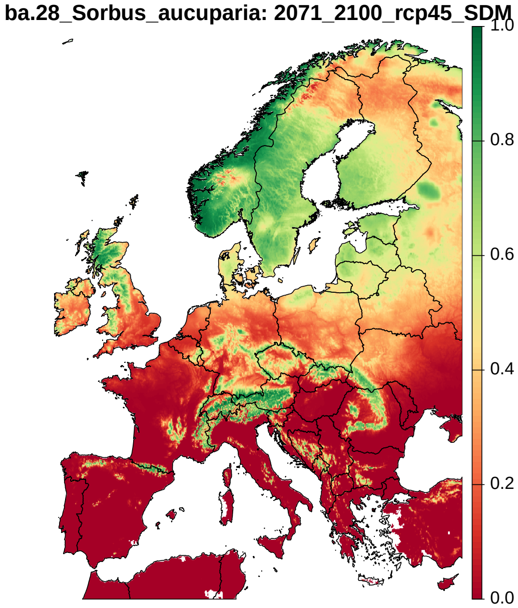

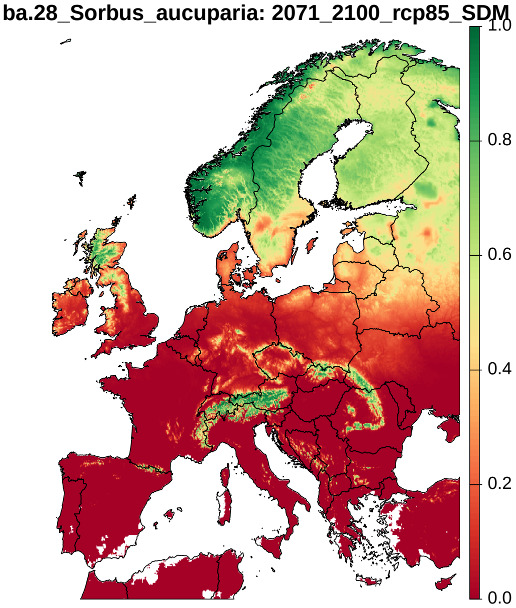

Climate risk classes

Maps of the climate risk classes. To estimate the distribution potential of each species as a mask for the SIMs, the continuous SDM outputs were categorized into three classes: low (yellow), medium (blue) and high climatic risk (red). The maps depict the risk classes in reference time (1981 to 2010), in climate scenario RCP4.5 (2071-2100) and RCP8.5 (2071-2100). To get an impression how well the thresholds fit to the data, presences (black) and absences (grey) were added on the reference map (top left). Refer to the legend and section "SDM thresholds" for the thresholds.

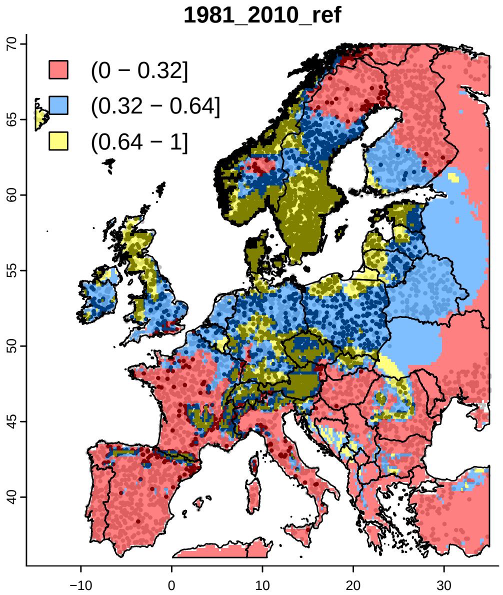

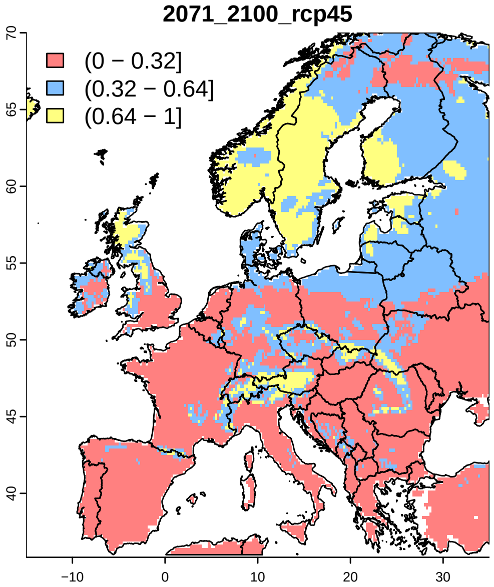

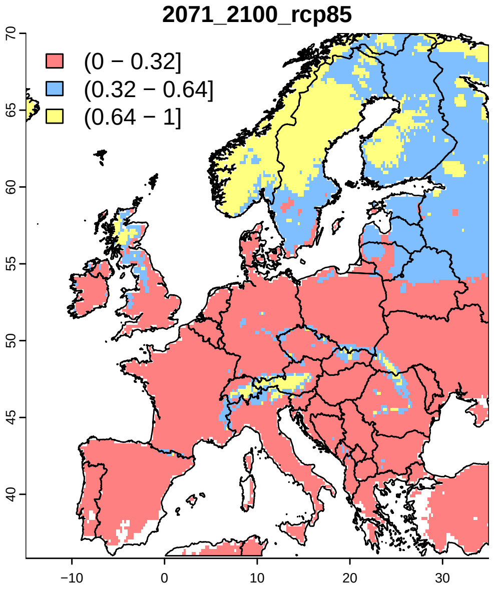

Sorbus torminalis

Model statistics and evaluation

Summary

Predictor acronyms: Bio.10 = Mean temperature of warmest quarter [°C] within months 6 to 8, Bio.11 = Mean temperature of coldest quarter [°C] within months 12,1,2, Bio.12 = Annual precipitation sum [mm/m2], Bio.18 = Mean monthly precipitation amount of the warmest quarter [mm/m2] within months 6 to 8.

```
##
## Family: binomial
## Link function: logit
##
## Formula:
## ba.26 ~ s(Bio.10, k = 3) + s(Bio.11, k = 3) + s(Bio.18, k = 3)
##
## Parametric coefficients:
##             Estimate Std. Error z value Pr(>|z|)
## (Intercept)  -2.0622     0.1514  -13.62   <2e-16 ***
## ---
## Signif. codes:  0 '***' 0.001 '**' 0.01 '*' 0.05 '.' 0.1 ' ' 1
##
## Approximate significance of smooth terms:
##             edf Ref.df Chi.sq p-value
## s(Bio.10) 1.996  2.000 275.78  <2e-16 ***
## s(Bio.11) 1.978  1.999 216.06  <2e-16 ***
## s(Bio.18) 1.751  1.938  86.41  <2e-16 ***
## ---
## Signif. codes:  0 '***' 0.001 '**' 0.01 '*' 0.05 '.' 0.1 ' ' 1
##
## R-sq.(adj) =  0.555   Deviance explained = 45.1%
## -REML = 985.21  Scale est. = 1         n = 2556
```

Evaluation parameter

Model performance was assessed using four statistical parameters: the area under the receiver operating characteristic curve (AUC), the true skill statistic (TSS), sensitivity (probability of the model to correctly predict a true presence) and specificity (probability of the model to correctly predict a true absence).

```
##         Species_name  AUC       TSS sensitivity specificity
## tp Sorbus torminalis 0.91 0.6932707   0.9201878   0.7730829
```

Response curves and response maps

Response curves

Response curves (also known as effect curves) give an overview of the climatic niche of a species by relating the occurrence probability to corresponding climatic values. Predictor acronyms: Bio.10 = Mean temperature of warmest quarter [°C] within months 6 to 8, Bio.11 = Mean temperature of coldest quarter [°C] within months 12,1,2, Bio.12 = Annual precipitation sum [mm/m2], Bio.18 = Mean monthly precipitation amount of the warmest quarter [mm/m2] within months 6 to 8. Lines on the x-axis mark the upper and lower limit of the used presences (red), the mean (bold black) and the median (bold blue).

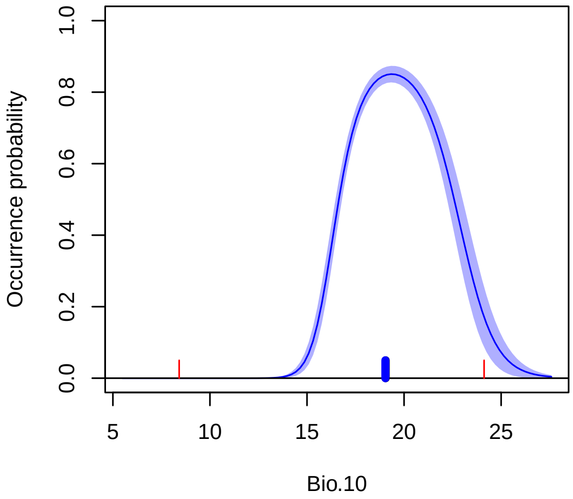

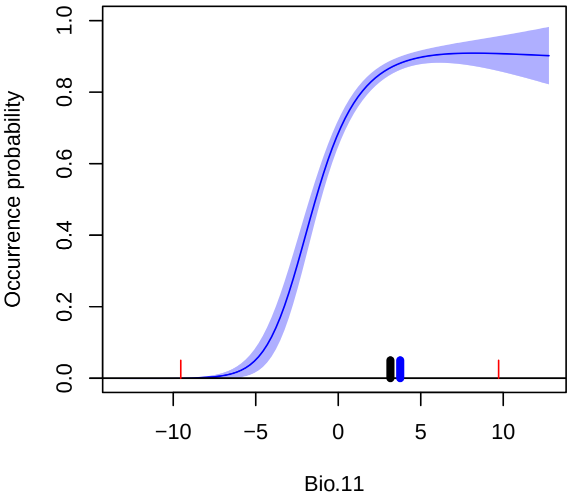

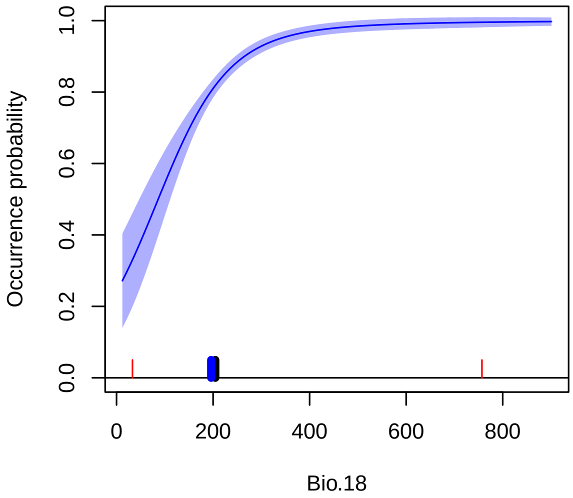

Response maps

Response maps (also referred to as partial effect maps). Each map represents how each predictor affects the occurrence probability. Predictor acronyms: Bio.10 = Mean temperature of warmest quarter [°C] within months 6 to 8, Bio.11 = Mean temperature of coldest quarter [°C] within months 12,1,2, Bio.12 = Annual precipitation sum [mm/m2], Bio.18 = Mean monthly precipitation amount of the warmest quarter [mm/m2] within months 6 to 8.

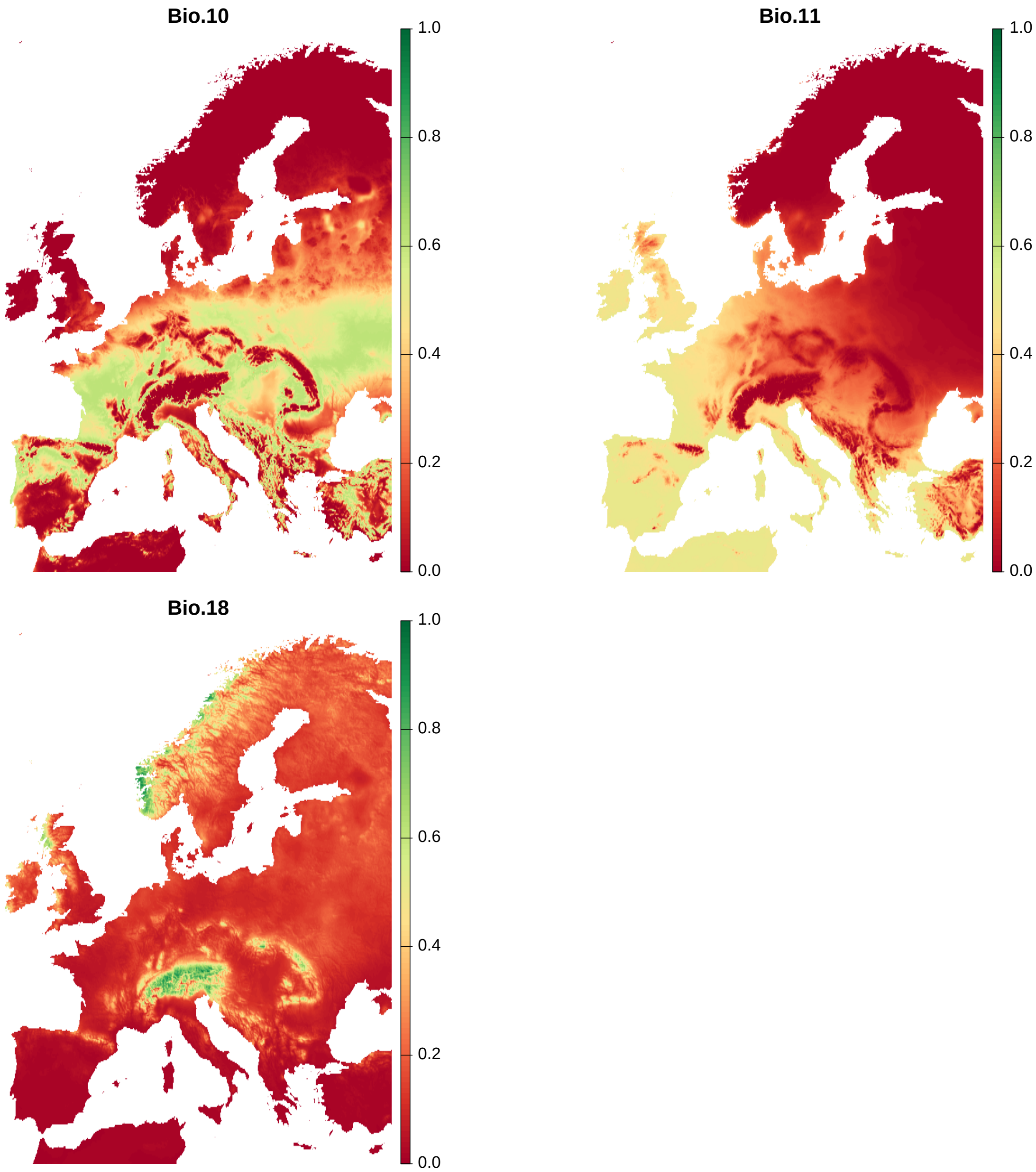

Model projections

Projection with plotted input data

Projection of species distribution model for reference period 1981-2010 over Europe. Occurrence probability ranges from 0 to 1 and is represented in dark red (low probability) to dark green (high probability). Input data used to calibrate the model is shown as presence points in magenta and absence points in black.

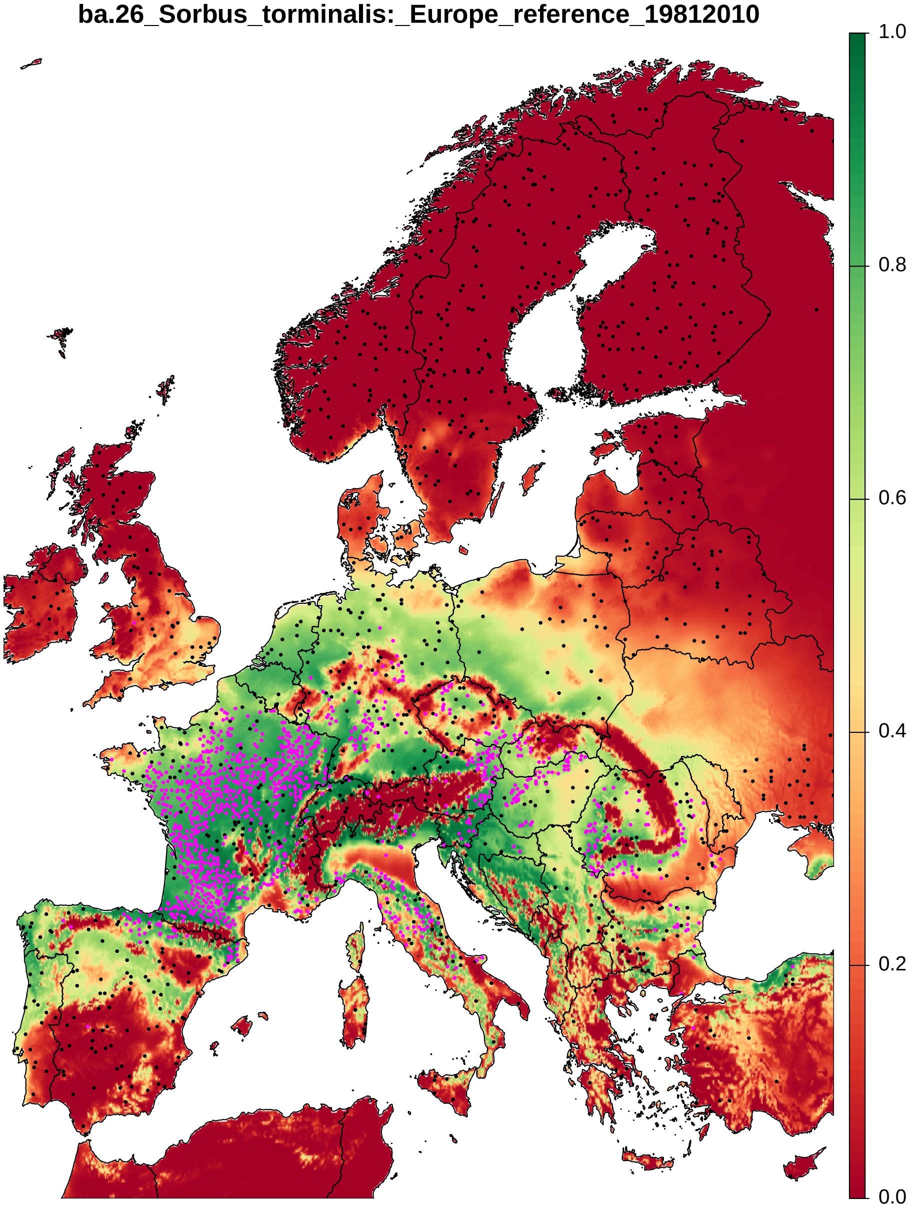

Projections

Projections of the species distribution models for reference period (1981-2010) and future scenarios RCP4.5 (2071-2100) and RCP8.5 (2071-2100) over Europe. Occurrence probabilities range from 0 to 1 and are represented from dark red (low probability) to dark green (high probability).

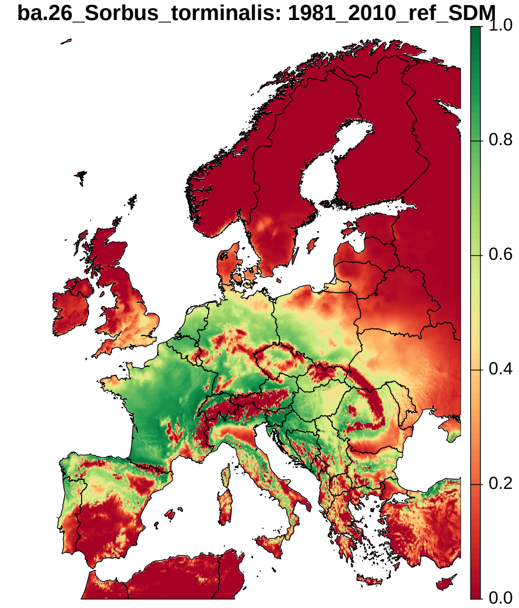

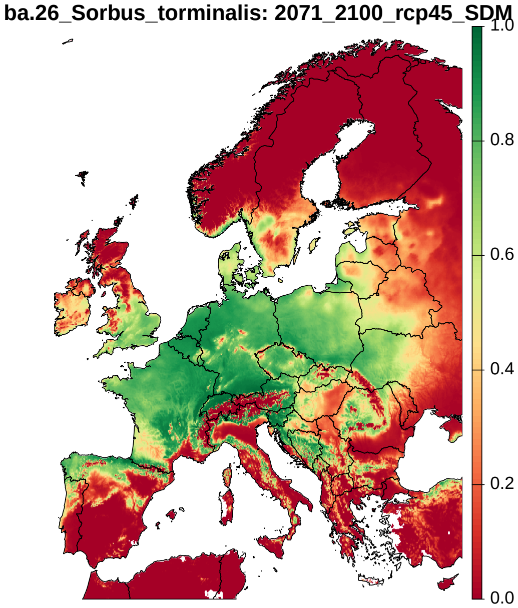

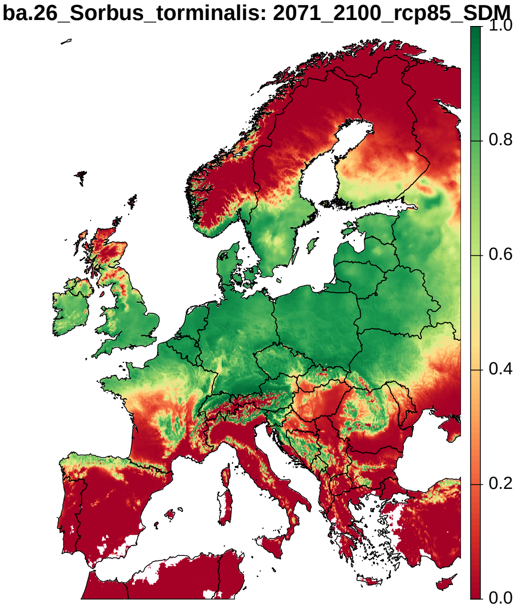

Climate risk classes

Maps of the climate risk classes. To estimate the distribution potential of each species as a mask for the SIMs, the continuous SDM outputs were categorized into three classes: low (yellow), medium (blue) and high climatic risk (red). The maps depict the risk classes in reference time (1981 to 2010), in climate scenario RCP4.5 (2071-2100) and RCP8.5 (2071-2100). To get an impression how well the thresholds fit to the data, presences (black) and absences (grey) were added on the reference map (top left). Refer to the legend and section "SDM thresholds" for the thresholds.

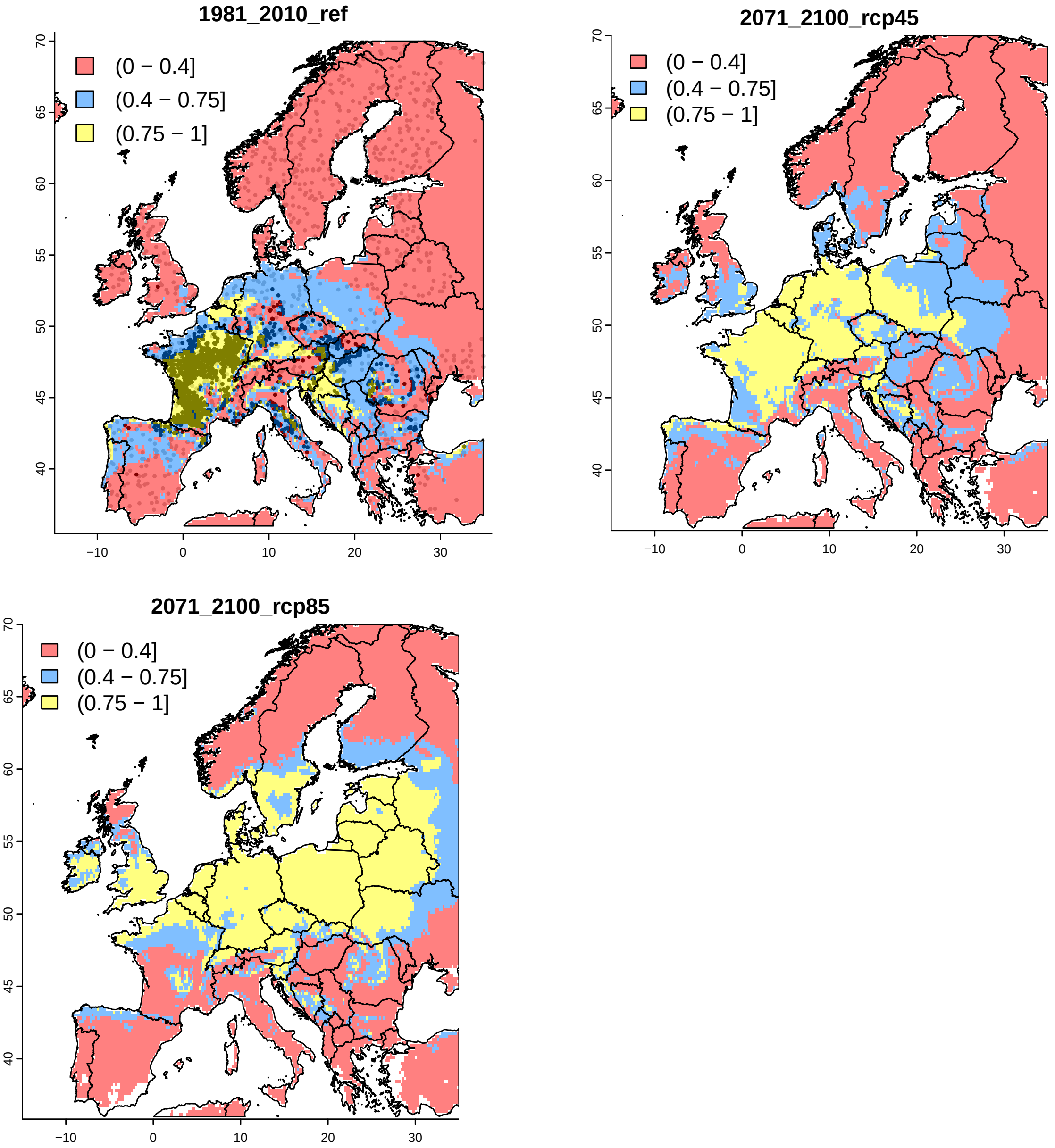

Tilia cordata

Model statistics and evaluation

Summary

Predictor acronyms: Bio.10 = Mean temperature of warmest quarter [°C] within months 6 to 8, Bio.11 = Mean temperature of coldest quarter [°C] within months 12,1,2, Bio.12 = Annual precipitation sum [mm/m2], Bio.18 = Mean monthly precipitation amount of the warmest quarter [mm/m2] within months 6 to 8.

```
##
## Family: binomial
## Link function: logit
##
## Formula:
## ba.20 ~ s(Bio.10, k = 3) + s(Bio.11, k = 3) + s(Bio.18, k = 3)
##
## Parametric coefficients:
##             Estimate Std. Error z value Pr(>|z|)
## (Intercept) -0.87796    0.07326  -11.98   <2e-16 ***
## ---
## Signif. codes:  0 '***' 0.001 '**' 0.01 '*' 0.05 '.' 0.1 ' ' 1
##
## Approximate significance of smooth terms:
##             edf Ref.df Chi.sq p-value
## s(Bio.10) 1.997      2  326.4  <2e-16 ***
## s(Bio.11) 1.989      2  108.5  <2e-16 ***
## s(Bio.18) 1.979      2  180.7  <2e-16 ***
## ---
## Signif. codes:  0 '***' 0.001 '**' 0.01 '*' 0.05 '.' 0.1 ' ' 1
##
## R-sq.(adj) =  0.416   Deviance explained = 36.2%
## -REML = 1614.8  Scale est. = 1         n = 3616
```

Evaluation parameter

Model performance was assessed using four statistical parameters: the area under the receiver operating characteristic curve (AUC), the true skill statistic (TSS), sensitivity (probability of the model to correctly predict a true presence) and specificity (probability of the model to correctly predict a true absence).

```
##     Species_name  AUC      TSS sensitivity specificity
## tp Tilia cordata 0.86 0.574115   0.8550885   0.7190265
```

Response curves and response maps

Response curves

Response curves (also known as effect curves) give an overview of the climatic niche of a species by relating the occurrence probability to corresponding climatic values. Predictor acronyms: Bio.10 = Mean temperature of warmest quarter [°C] within months 6 to 8, Bio.11 = Mean temperature of coldest quarter [°C] within months 12,1,2, Bio.12 = Annual precipitation sum [mm/m2], Bio.18 = Mean monthly precipitation amount of the warmest quarter [mm/m2] within months 6 to 8. Lines on the x-axis mark the upper and lower limit of the used presences (red), the mean (bold black) and the median (bold blue).

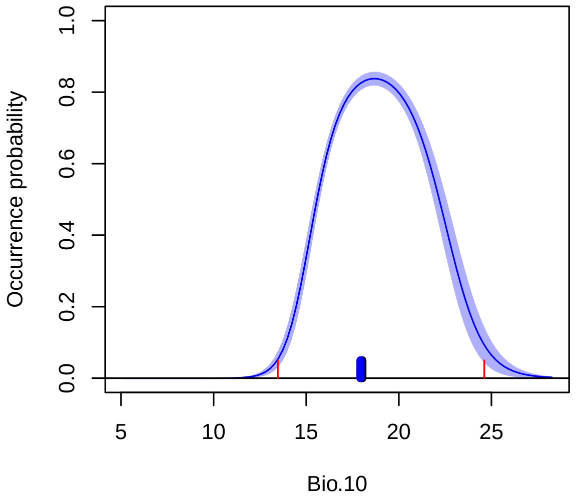

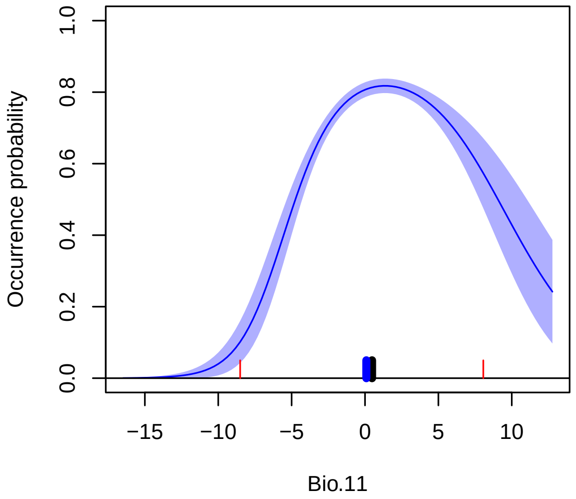

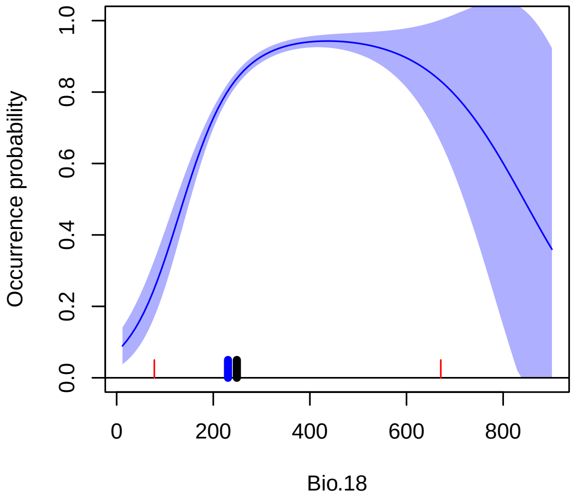

Response maps

Response maps (also referred to as partial effect maps). Each map represents how each predictor affects the occurrence probability. Predictor acronyms: Bio.10 = Mean temperature of warmest quarter [°C] within months 6 to 8, Bio.11 = Mean temperature of coldest quarter [°C] within months 12,1,2, Bio.12 = Annual precipitation sum [mm/m2], Bio.18 = Mean monthly precipitation amount of the warmest quarter [mm/m2] within months 6 to 8.

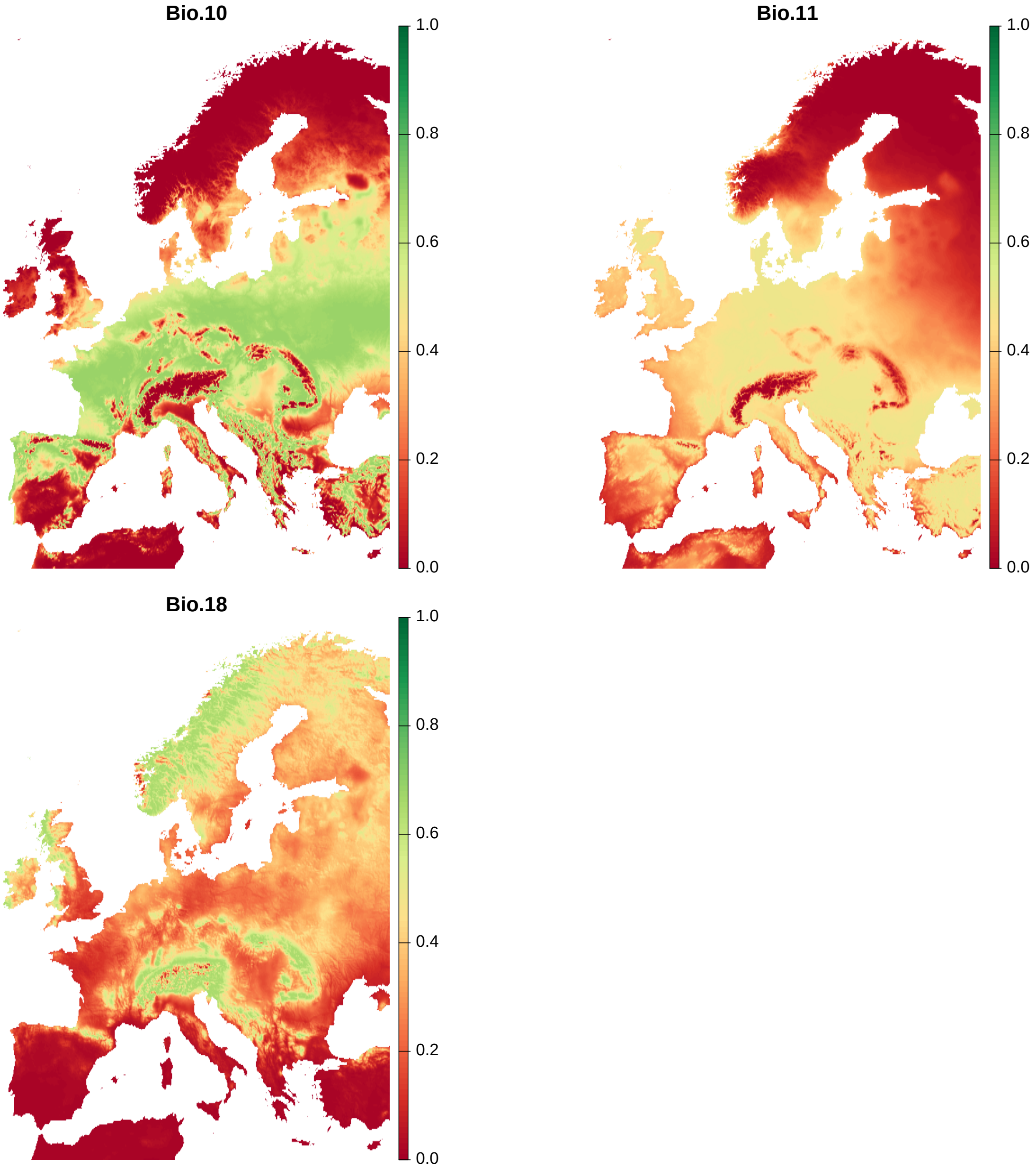

Model projections

Projection with plotted input data

Projection of species distribution model for reference period 1981-2010 over Europe. Occurrence probability ranges from 0 to 1 and is represented in dark red (low probability) to dark green (high probability). Input data used to calibrate the model is shown as presence points in magenta and absence points in black.

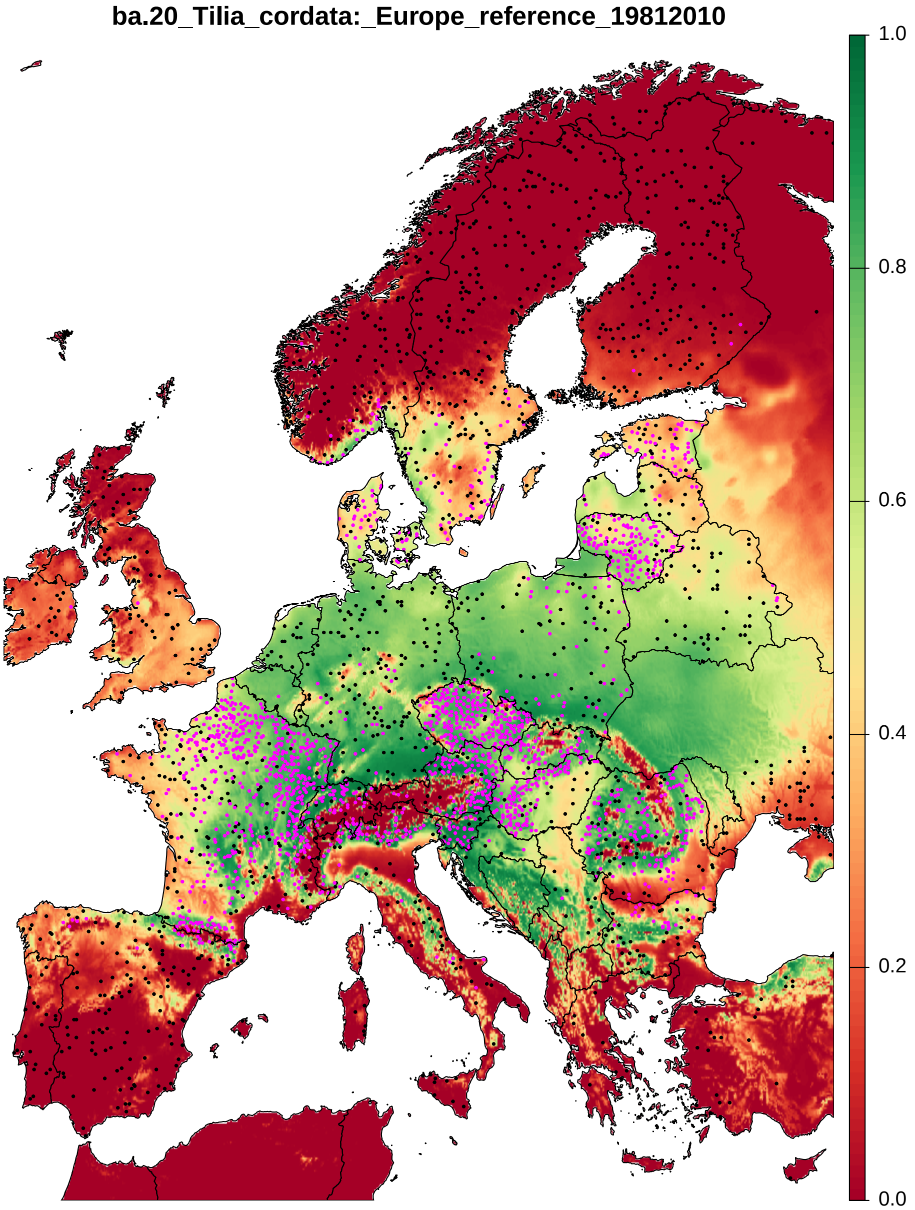

Projections

Projections of the species distribution models for reference period (1981-2010) and future scenarios RCP4.5 (2071-2100) and RCP8.5 (2071-2100) over Europe. Occurrence probabilities range from 0 to 1 and are represented from dark red (low probability) to dark green (high probability).

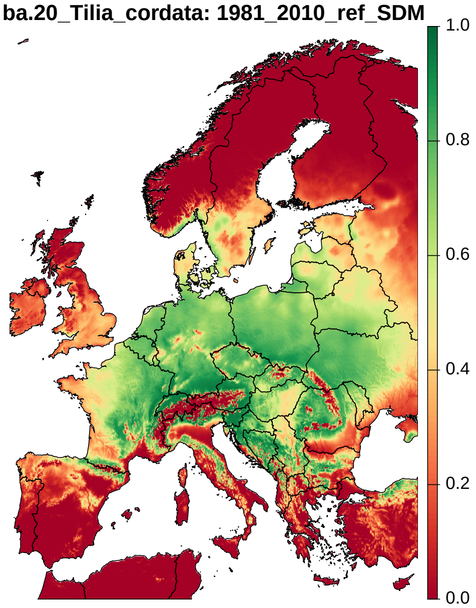

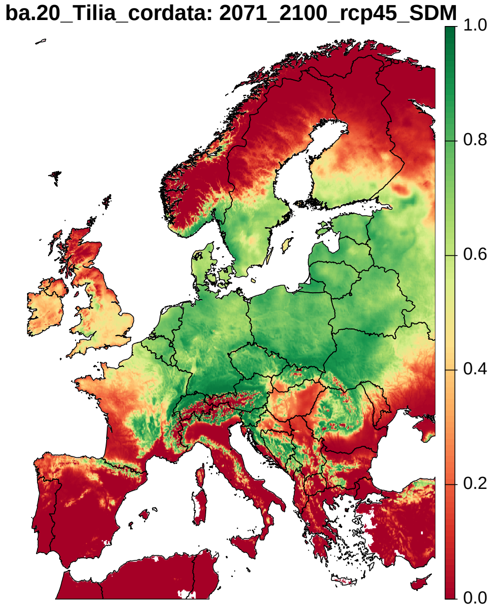

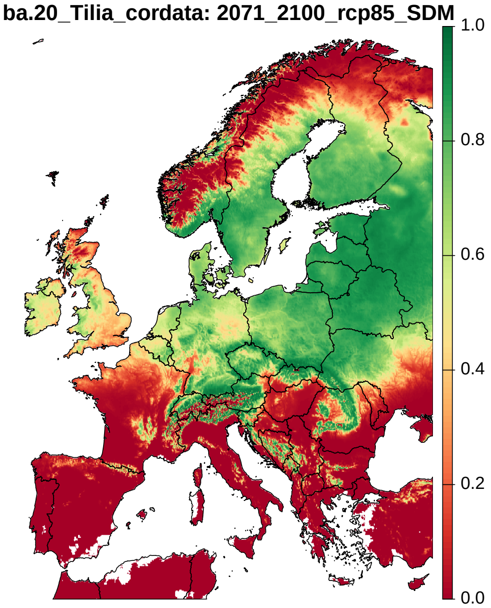

Climate risk classes

Maps of the climate risk classes. To estimate the distribution potential of each species as a mask for the SIMs, the continuous SDM outputs were categorized into three classes: low (yellow), medium (blue) and high climatic risk (red). The maps depict the risk classes in reference time (1981 to 2010), in climate scenario RCP4.5 (2071-2100) and RCP8.5 (2071-2100). To get an impression how well the thresholds fit to the data, presences (black) and absences (grey) were added on the reference map (top left). Refer to the legend and section "SDM thresholds" for the thresholds.

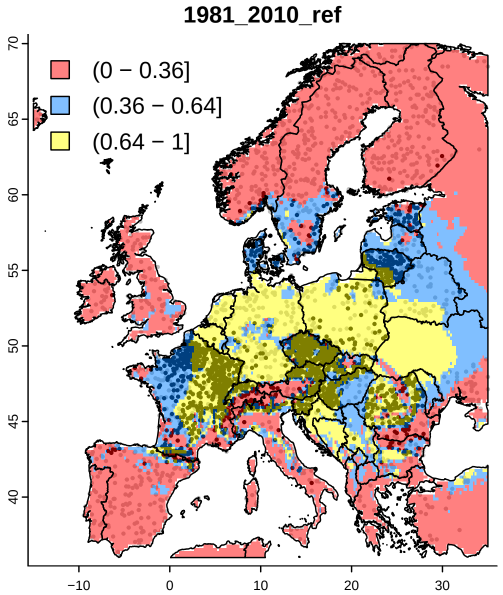

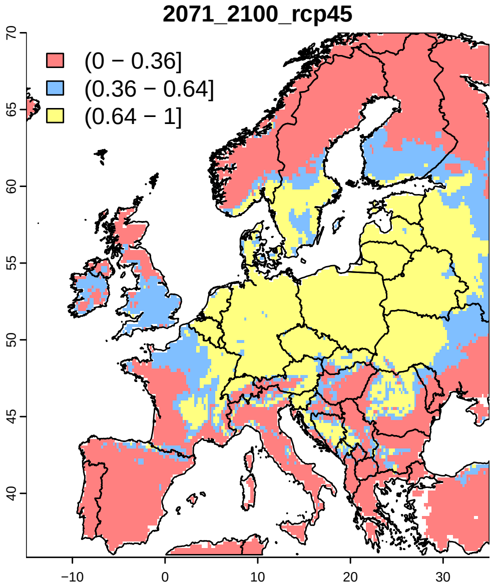

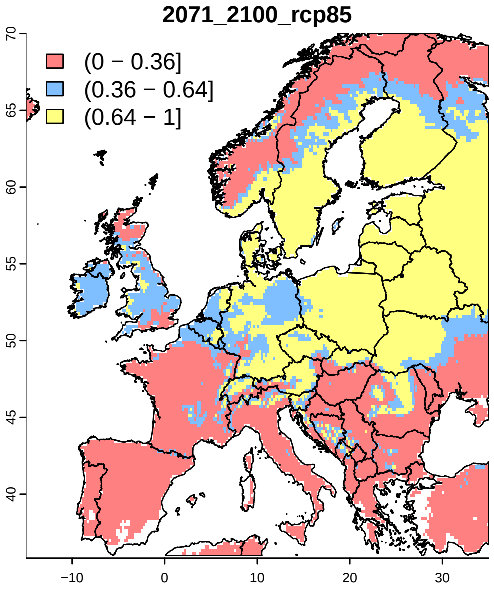

Ulmus laevis

Model statistics and evaluation

Summary

Predictor acronyms: Bio.10 = Mean temperature of warmest quarter [°C] within months 6 to 8, Bio.11 = Mean temperature of coldest quarter [°C] within months 12,1,2, Bio.12 = Annual precipitation sum [mm/m2], Bio.18 = Mean monthly precipitation amount of the warmest quarter [mm/m2] within months 6 to 8.

```
##
## Family: binomial
## Link function: logit
##
## Formula:
## ba.62 ~ s(Bio.10, k = 3) + s(Bio.11, k = 3) + s(Bio.18, k = 3)
##
## Parametric coefficients:
##             Estimate Std. Error z value Pr(>|z|)
## (Intercept)  -0.8946     0.1731  -5.167 2.37e-07 ***
## ---
## Signif. codes:  0 '***' 0.001 '**' 0.01 '*' 0.05 '.' 0.1 ' ' 1
##
## Approximate significance of smooth terms:
##             edf Ref.df Chi.sq p-value
## s(Bio.10) 1.947  1.997  102.4  <2e-16 ***
## s(Bio.11) 1.913  1.992   34.2  <2e-16 ***
## s(Bio.18) 1.937  1.996   51.6  <2e-16 ***
## ---
## Signif. codes:  0 '***' 0.001 '**' 0.01 '*' 0.05 '.' 0.1 ' ' 1
##
## R-sq.(adj) =  0.427   Deviance explained = 37.3%
## -REML = 351.49  Scale est. = 1         n = 786
```

Evaluation parameter

Model performance was assessed using four statistical parameters: the area under the receiver operating characteristic curve (AUC), the true skill statistic (TSS), sensitivity (probability of the model to correctly predict a true presence) and specificity (probability of the model to correctly predict a true absence).

```
##    Species_name  AUC       TSS sensitivity specificity
## tp Ulmus laevis 0.88 0.6412214   0.8753181   0.7659033
```

Response curves and response maps

Response curves

Response curves (also known as effect curves) give an overview of the climatic niche of a species by relating the occurrence probability to corresponding climatic values. Predictor acronyms: Bio.10 = Mean temperature of warmest quarter [°C] within months 6 to 8, Bio.11 = Mean temperature of coldest quarter [°C] within months 12,1,2, Bio.12 = Annual precipitation sum [mm/m2], Bio.18 = Mean monthly precipitation amount of the warmest quarter [mm/m2] within months 6 to 8. Lines on the x-axis mark the upper and lower limit of the used presences (red), the mean (bold black) and the median (bold blue).

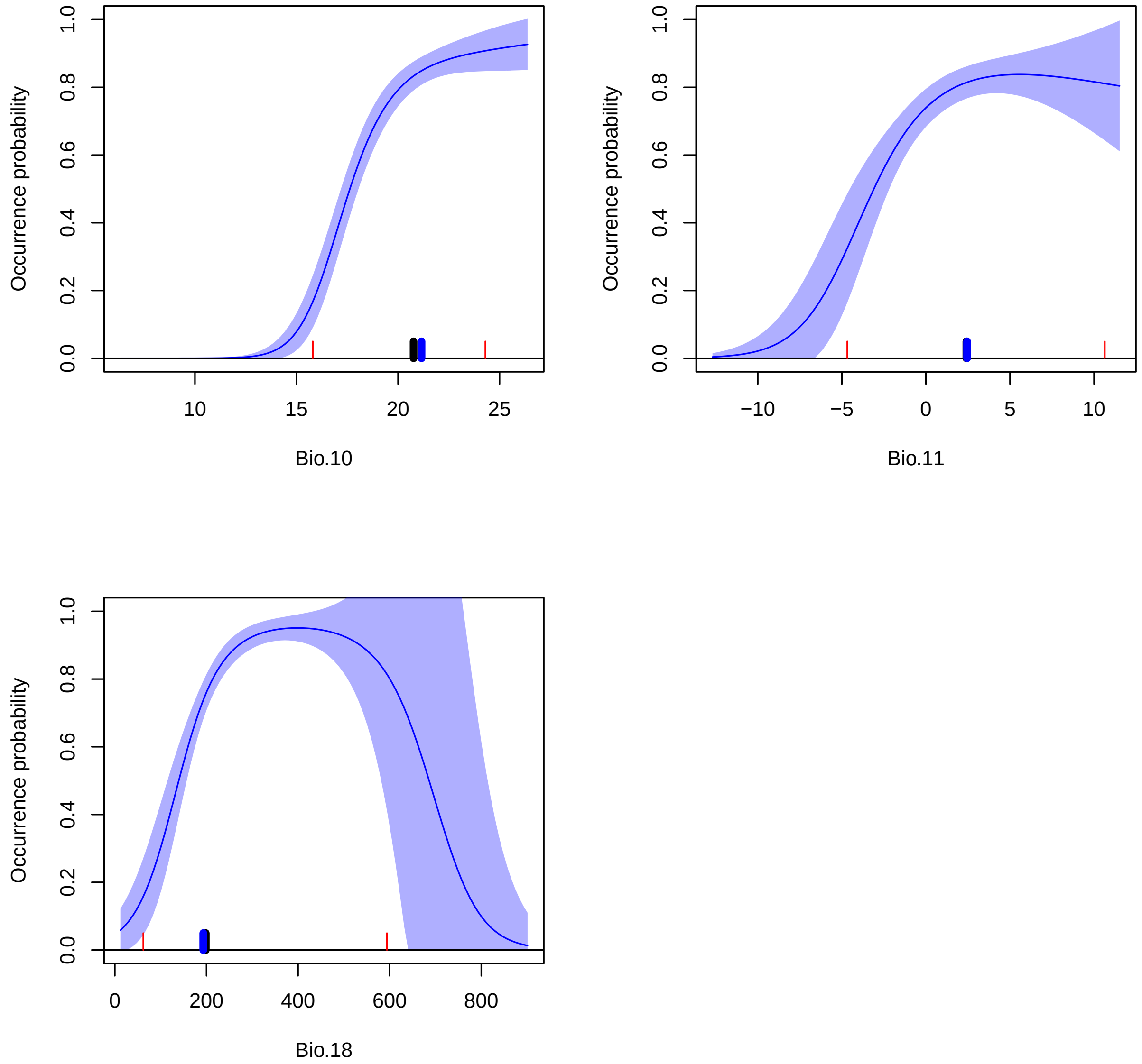

Response maps

Response maps (also referred to as partial effect maps). Each map represents how each predictor affects the occurrence probability. Predictor acronyms: Bio.10 = Mean temperature of warmest quarter [°C] within months 6 to 8, Bio.11 = Mean temperature of coldest quarter [°C] within months 12,1,2, Bio.12 = Annual precipitation sum [mm/m2], Bio.18 = Mean monthly precipitation amount of the warmest quarter [mm/m2] within months 6 to 8.

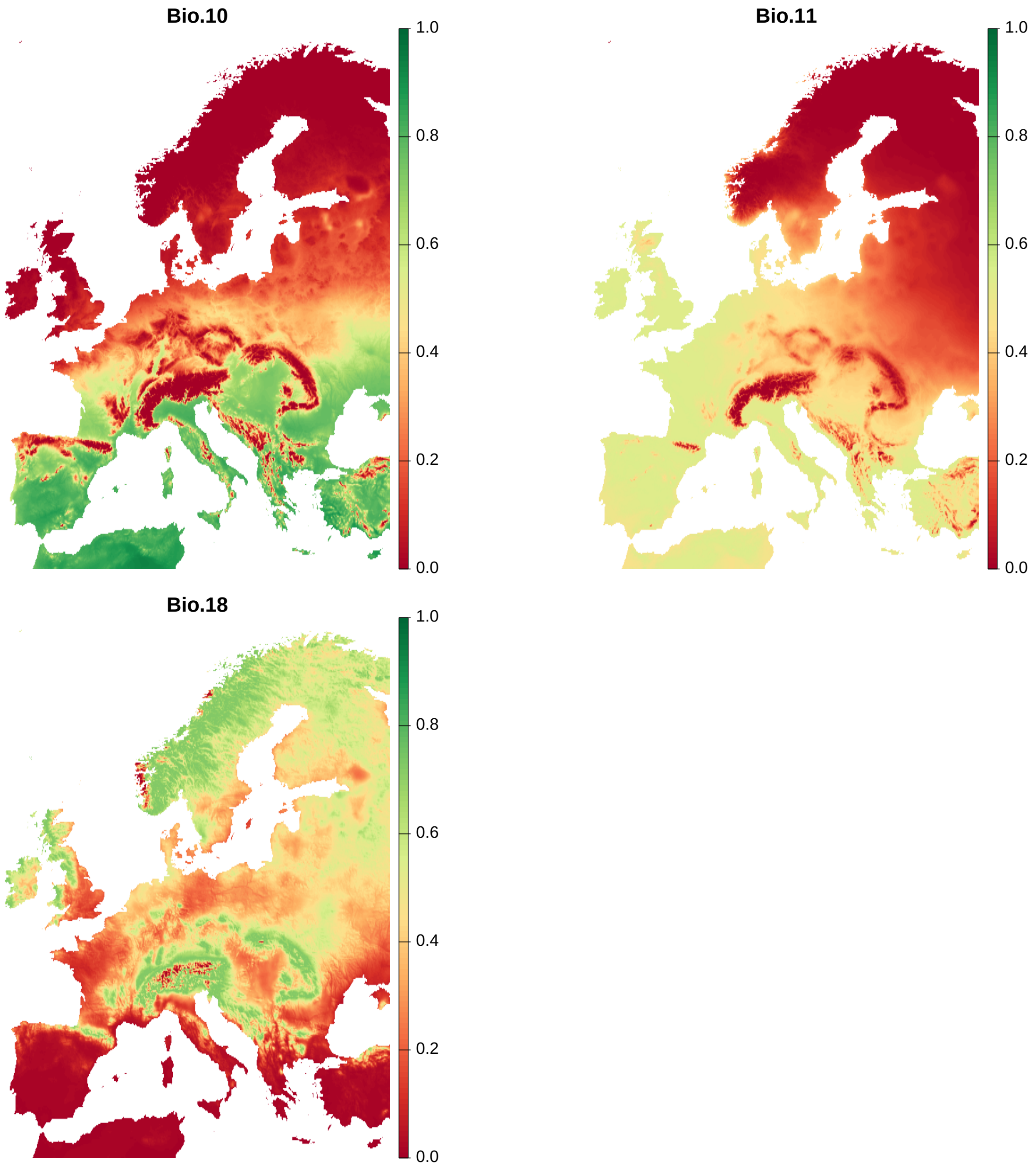

Model projections

Projection with plotted input data

Projection of species distribution model for reference period 1981-2010 over Europe. Occurrence probability ranges from 0 to 1 and is represented in dark red (low probability) to dark green (high probability). Input data used to calibrate the model is shown as presence points in magenta and absence points in black.

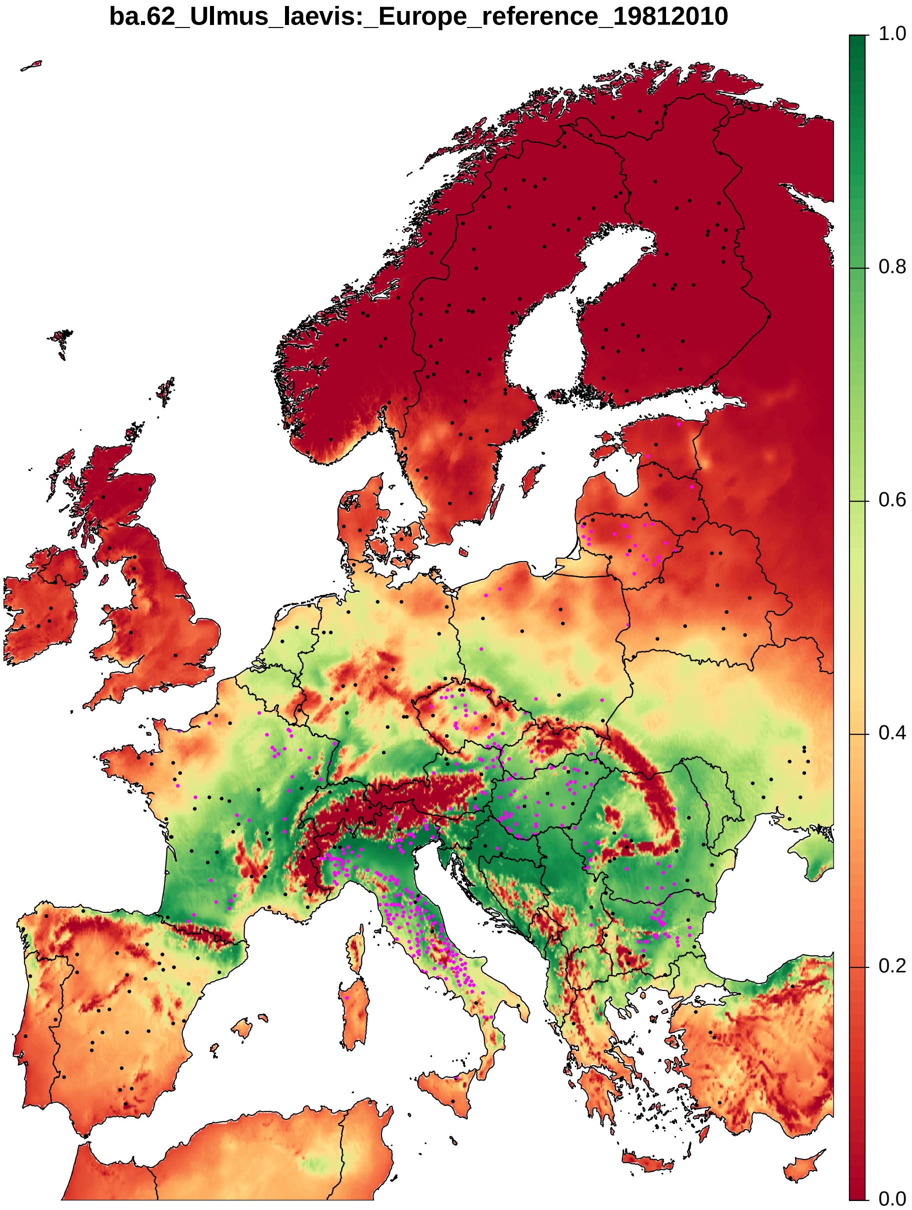

Projections

Projections of the species distribution models for reference period (1981-2010) and future scenarios RCP4.5 (2071-2100) and RCP8.5 (2071-2100) over Europe. Occurrence probabilities range from 0 to 1 and are represented from dark red (low probability) to dark green (high probability).

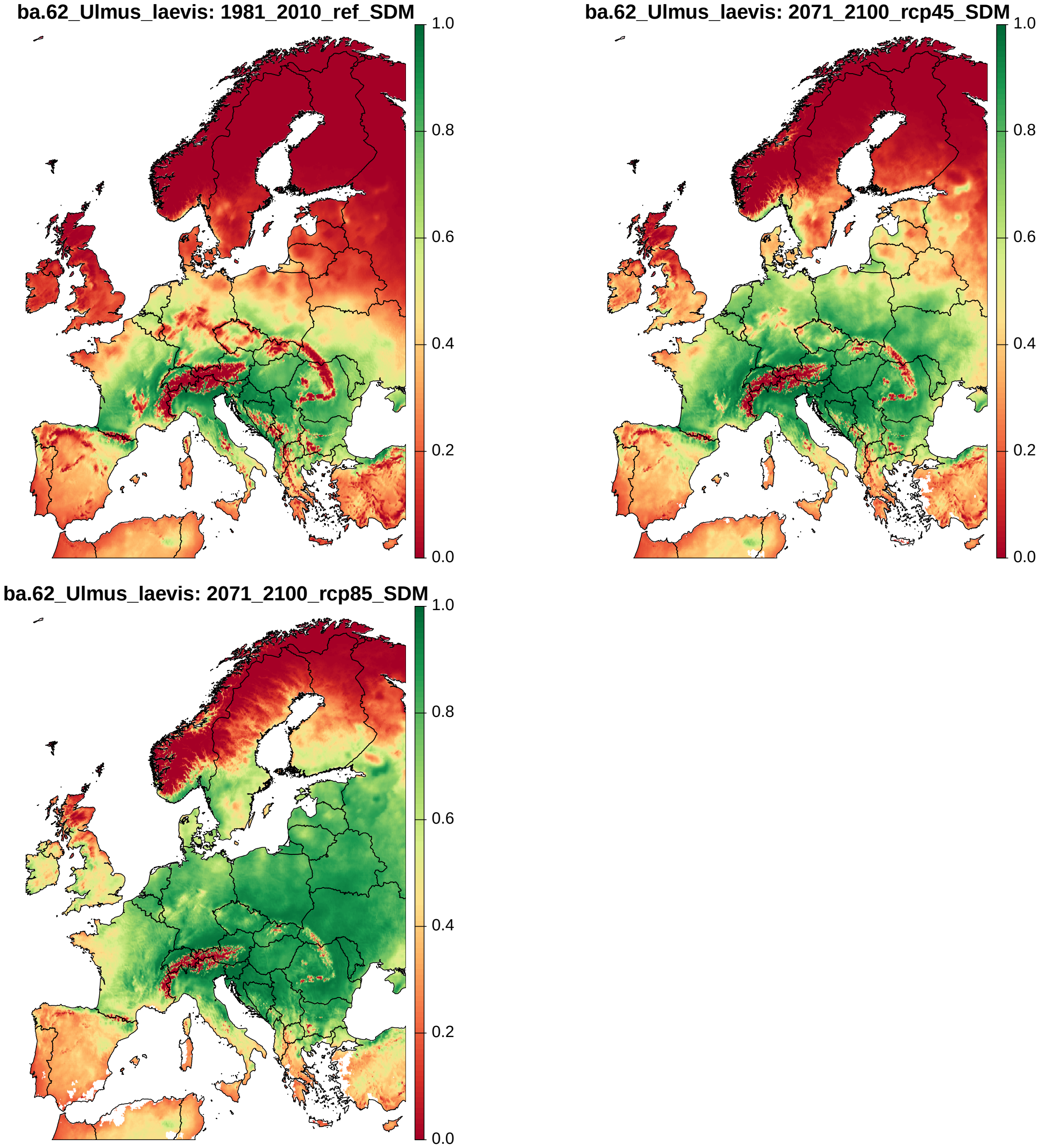

Climate risk classes

Maps of the climate risk classes. To estimate the distribution potential of each species as a mask for the SIMs, the continuous SDM outputs were categorized into three classes: low (yellow), medium (blue) and high climatic risk (red). The maps depict the risk classes in reference time (1981 to 2010), in climate scenario RCP4.5 (2071-2100) and RCP8.5 (2071-2100). To get an impression how well the thresholds fit to the data, presences (black) and absences (grey) were added on the reference map (top left). Refer to the legend and section "SDM thresholds" for the thresholds.

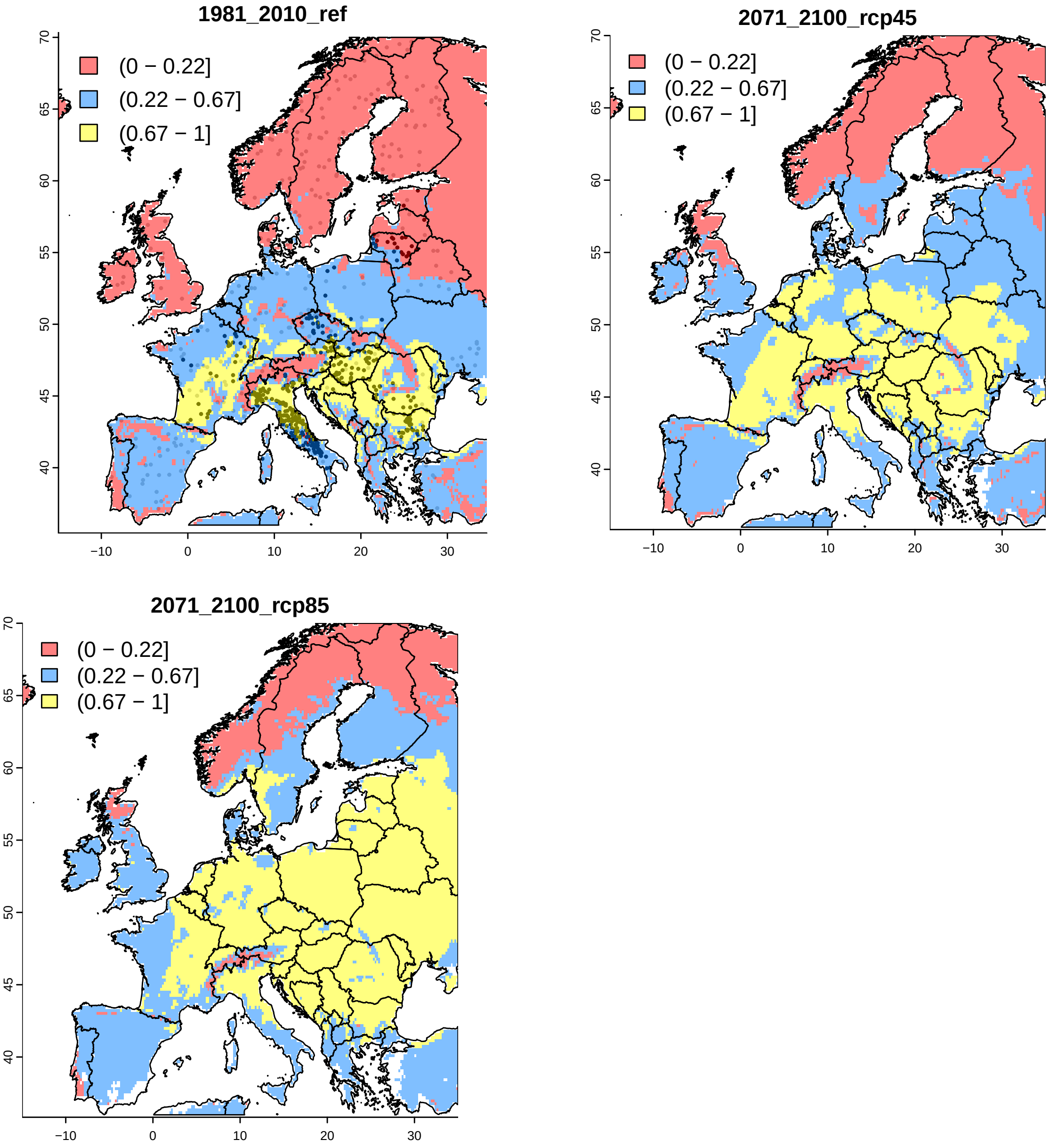

Supplement: Supplementary file 2 [file mmc2.pdf]
